# Total Synthesis of Benthol A: The Nominal and the Actual Natural Product

**DOI:** 10.1021/jacs.6c05583

**Published:** 2026-07-16

**Authors:** Guanghao Huang, Andrea Tomio, Thomas Varlet, Conny Wirtz, Alois Fürstner

**Affiliations:** Max-Planck-Institut für Kohlenforschung, 45470 Mülheim/Ruhr, Germany

## Abstract

The total synthesis of the dinoflagellate-derived “super-carbon-chain compound” benthol A rigorously confirmed what the analysis of one of the required building blocks had forecasted, namely that the configuration of the secondary −OH group at C40 had been misassigned by the isolation team as the only one of a total of 35 stereogenic centers decorating the backbone of this polyol/polyether derivative. This conclusion bears implications because the original assignment had been solely based on computational data, which had resulted in a remarkably high score of 99.93% that was ultimately misleading. The multiconvergent blueprint underlying the successful approach accounts for the fact that alongest linear sequence of 32 steps sufficed to reach this intricate marine natural product. The inherent flexibility should also empower future studies aiming at a detailed mapping of the pharmacophore of this compound endowed with significant antiplasmodial activity.

## Graphical abstract

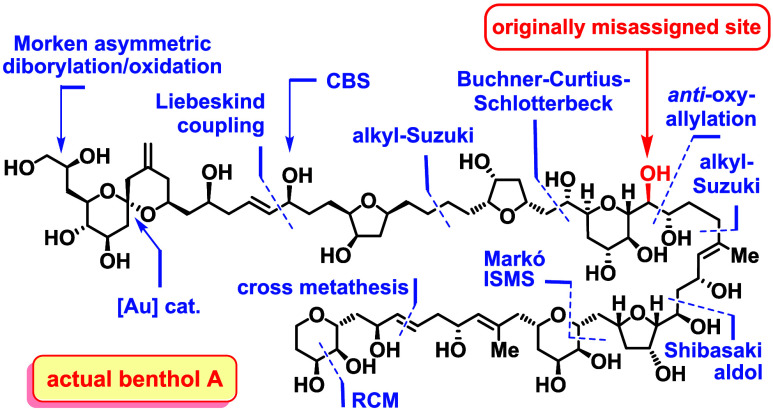

## Introduction

Dinoflagellates are known as a virtually inexhaustible source of new natural products with remarkable structures and prodigious biological properties.^1,2^ Even from this cornucopia, however, benthol A stands out in many regards.^3^ This compound consists of a linear backbone comprised of 72 C atoms, decorated with three one-carbon branches (one of which is an *exo*-methylene unit), 22 hydroxy groups, and eight fully saturated oxygen heterocycles; collectively, this functionality account for 35 stereogenic centers in addition to four stereogenic olefins. The polar and apolar substituents are distributed over the entire chain instead of forming hydrophilic and hydrophobic clusters, as observed in most other known “super-carbon-chain compounds” (SCCCs).^4–10^ In this respect, benthol A is reminiscent of palytoxin, although the latter is derived from an invertebrate rather than a dinoflagellate.^11^ The compound exhibits potent antiplasmodial as well as appreciable antiviral activity in vitro, whereas only modest cytotoxicity against human cancer cell lines was reported.^3^

In view of the molecular complexity and the limited amount of material available to the isolation team, the structure elucidation and determination of the relative and absolute configuration by Wu and co-workers was a veritable tour de force.^3^ It relied on extensive NMR spectroscopic investigations, thorough *J*-based configurational analysis, the careful examination of NOE interactions, as well as chemical derivatization (labeling, Mosher ester) and degradation experiments (ozonolysis, periodiate cleavage); in addition, DFT-based ^13^C NMR chemical-shift calculations complemented by a DP4+ statistical analysis^12^ of the data were used. Based on this evidence, benthol A was assigned structure **1** (Scheme 1).

**Scheme 1:**
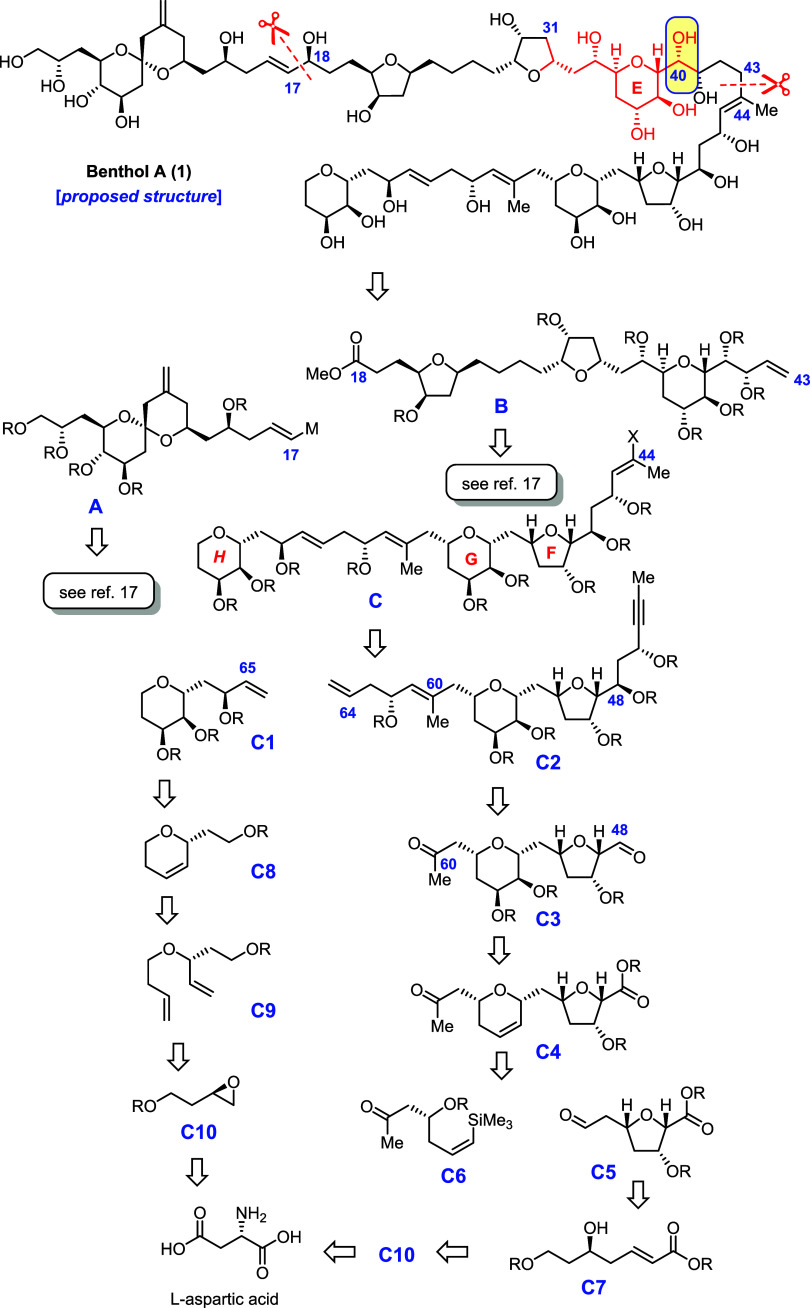
Proposed Structure of Benthol A, Global Retrosynthetic Analysis, and Detailed Analysis of Fragment **C**; the C31–C40 Region of Concern Is Color-Coded (Red) and the Presumed Site of Misassignment, as Identified in the Accompanying Paper, Highlighted in Yellow

Challenged by the molecular intricacy of benthol A and encouraged by its promising antimalaria activity,^13^ we embarked into a total synthesis of this alluring target as part of our long-term commitment to the study of dinoflagellate-derived natural products.^14–16^ To this end, a poly-convergent and inherently flexible blueprint was conceived based on three top fragments **A**, **B** and **C** of comparable size and complexity; their assembly should ultimately deliver the target compound (Scheme 1). As described in the accompanying paper,^17^ straightforward, productive and scalable routes to **A** and **B** were implemented. In parallel, these key building blocks were globally deprotected and derivatized as necessary for substructure authentication purposes by comparison of the spectral data of the synthetic samples with those of the corresponding sectors of benthol A and/or pertinent degradation products thereof. In so doing, confidence was gained that the spiroacetal fragment **A** corresponds perfectly to the “north-western” wing of **1**. For this excellent congruence, however, the non-negligible mis-match of the C31–C41 substructure of fragment **B** representing the tetrahydropyran E-ring of **1** and its immediate environment was all the more worrisome; the dissimilarity pertained to both the ^13^C NMR shifts as well as a series of ^3^ *J*_H,H_ coupling constants.

The pursuit of a target of this size that must be considered potentially misassigned for good reasons carries high risk of frustration.^18^ Therefore, we made every effort to clarify the issue before launching the final stages of the project. To this end, a number of modified building blocks was prepared and their spectral data carefully analyzed. This synthesis-driven approach ultimately provided convincing evidence that the spectral deviations along the 10 C-atom-long C31–C41 sector are almost certainly caused by a *single* mis-assigned stereogenic center.^17^ In this context, it is interesting to note that the isolation team had analyzed the configuration of the C40-OH group solely by computational means since massive signal overlap had prevented any use of pertinent NMR spectroscopic methods.^3^ All our evidence has let us propose with confidence that a single stereochemical “point mutation” at this very site is necessary in order to correct the structure of the target compound.^17^ This forecast was validated by the completion of the total syntheses of nominal (**1**) and actual (40-*epi*-**1**) benthol A outlined below.

## Results and Discussion

### Retrosynthesis of Segment **C**

The retrosynthetic analysis of fragment **C**, which had only briefly been addressed in the accompanying paper,^17^ is based on the incorporation of an alkenyl halide (or an equivalent thereof) at C44 of this building block. This functionality will serve the envisaged fragment coupling with the alkylboron reagent formed by hydroboration of the terminal double bond of the **B**- (or combined **AB**) segment via a B-alkyl-Suzuki reaction.^19–22^ Various ways to introduce the halide substituent were considered, of which we finally opted for a regio- and stereoselective hydrostannation of a methyl-capped alkyne followed by tin/halogen exchange. It was expected that an ordinary cross metathesis (CM)^23,24^ between the alkene **C1** as an adequate H-ring equivalent with the sterically unhindered alkene terminus of **C2** would kinetically outcompete an equally conceivable enyne metathesis^25^ with the triple bond on the other end of **C2**, provided the reaction is carried out under sufficiently mild conditions.^26–28^ Fragment **C1**, in turn, should be readily accessible from **C8**, which can be formed by ring closing metathesis (RCM) of diene **C9**.

Under the premise that the introduction of the terminal H-ring by CM leaves the alkyne on the other end of **C2** intact, this building block can be simplified by envisaging a two-directional chain extension strategy via olefination/asymmetric allylation on the “left” end and an asymmetric aldol reaction on the other terminus.^29^ While the exact timing of these events had to be defined during implementation, they lead back to a fragment of type **C3**, which we planned to assemble via an intramolecular silyl-modified Sakurai-type (ISMS) reaction^30–32^ of the alkenyl silane **C6** with aldehyde **C5** followed by dihydroxylation of the dihydropyran ring in **C4** thus formed. **C5** is a one-carbon homologue of the building block used twice en route to the central fragments **B** as well as **B′** (see accompanying paper)^17^ and can hence be obtained from **C7** by adapting the established route. It is also of note that **C7** and **C9** lead back to the same epoxide **C10** that is readily accessible from l-aspartic acid; this very same building block has already been used to make fragment **A** (see the accompanying paper).^17^ This triple convergency is favorable in terms of the overall step count and reduces the workload.

### The Forward Synthesis Route

l-Aspartic acid was transformed in three high-yielding steps on multigram scale into epoxide **2** by following a literature route.^33^ Opening of **2** on treatment with the sulfur ylide derived from [Me_3_S]I and *n*BuLi furnished the allylic alcohol **3** (Scheme 2).^34–36^ Since attempted etherification with 4-bromo-1-butene failed because of overly fast HBr elimination, alcohol **3** was subjected to O-alkylation with 1,4-dibromobutane under phase transfer conditions,^37^ and the resulting primary bromide was treated with *t*BuOK and catalytic amounts of 18-crown-6, again under biphasic conditions,^38^ to give diene **4** in readiness for RCM. As expected, this transformation proceeded smoothly with the aid of second-generation Grubbs ruthenium catalyst to give multigram quantities of the corresponding dihydropyran.^26^ The subsequent dihydroxylation with catalytic OsO_4_ and NMO as the terminal oxidant proceeded with outstanding diastereoselectivity to give diol **5** as the virtually only detectable isomer. This compound was easily elaborated into aldehyde **6**, which reacted with vinylmagnesium bromide under Cram-chelate control to afford the desired allylic alcohol **7** as the major product (dr ≈ 2.4:1).^39,40^ The minor isomer **8** could be separated and oxidized, and the resulting ketone was reduced with the (*R*)-CBS catalyst^41^ to give a second crop of **7** representing building block **C1**.

**Scheme 2:**
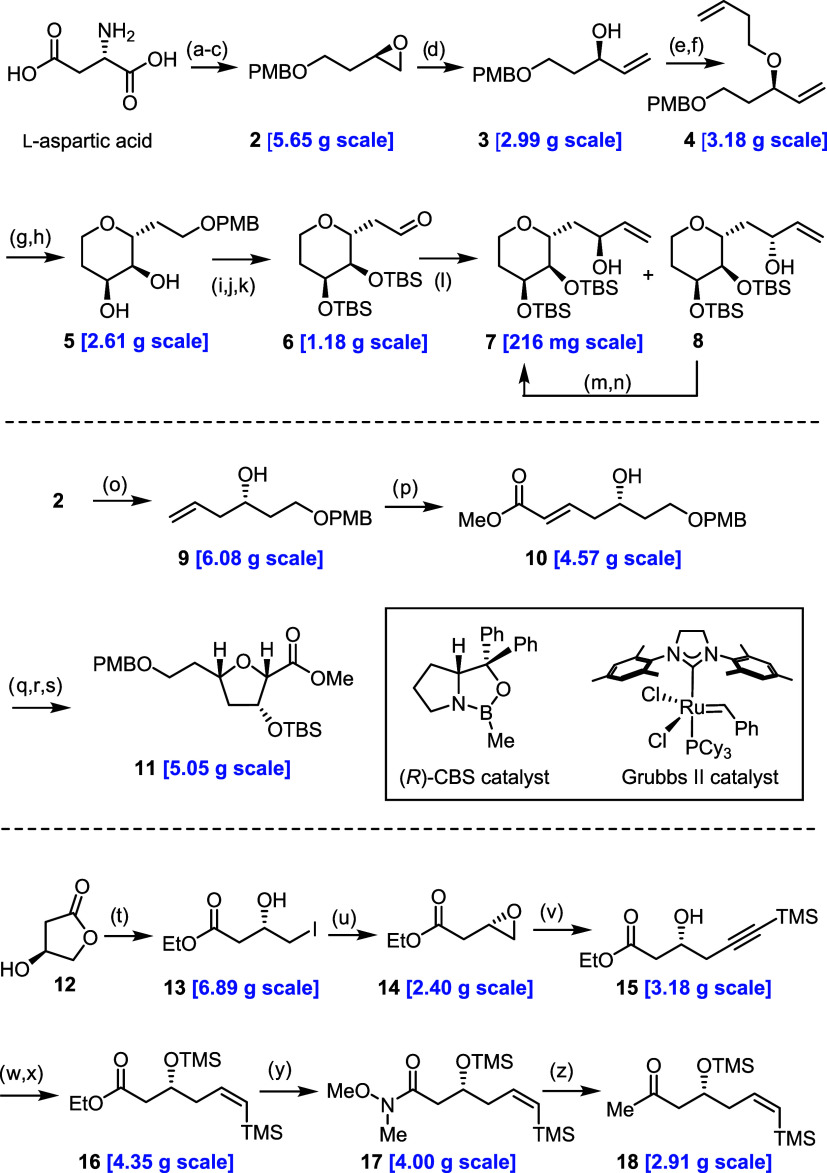
Forward Synthesis Route to Fragment **C** ^a^

a Reagents and Conditions: (a) NaNO_2_, KBr, H_2_SO_4_ cat., H_2_O, −5°C, 88% [13 g scale]; (b) BH_3_·THF, THF, −5°C to RT, 96% [5.39 g scale]; (c) NaH, THF/DMF, −5°C, then PMBBr, 92%; (d) [Me_3_S]­I, *n*BuLi, THF, 0 °C, 93%; (e) 1,4-dibromobutane, KOH, *n*Bu_4_NBr (10 mol %), toluene, RT, 92% [4.45 g scale]; (f) *t*BuOK, 18-crown-6 (15 mol %), toluene, 0 °C to RT, 92%; (g) Grubbs II catalyst (1 mol %), CH_2_Cl_2_, reflux, 95% [2.73 g scale]; (h) OsO_4_ (10 mol %), NMO, THF/H_2_O (3:1), RT, 84%; (i) TBSOTf, 2,6-lutidine, CH_2_Cl_2_, 0 °C, quant. [4.70 g scale]; (j) DDQ, CH_2_Cl_2_/H_2_O (95:5), RT, quant. [3.35 g scale]; (k) Dess-Martin periodinane, NaHCO_3_, CH_2_Cl_2_, RT, quant.; (l) vinylmagnesium bromide, THF, 0 °C, 61% (**7**) + 25% (**8**); (m) Dess-Martin periodinane, NaHCO_3_, CH_2_Cl_2_, RT; (n) (*R*)-CBS catalyst (20 mol %), BH_3_·SMe_2_, THF, −20 °C to 0 °C, 52% (over two steps [125 mg scale], dr = 18:1); (o) vinylmagnesium bromide, CuI (25 mol %), THF, −78 °C, 95%; (p) methyl acrylate, Grubbs II catalyst (2 × 1 mol %), CH_2_Cl_2_, reflux, 93%; (q) methanesulfonyl chloride, Et_3_N, CH_2_Cl_2_, 0 °C; (r) (DHQD)_2_PHAL (4 mol %), K_2_OsO_2_(OH)_4_ (2 mol %), K_3_Fe­(CN)_6_, MeSO_2_NH_2_, K_2_CO_3_, *t*BuOH, H_2_O, CH_2_Cl_2_, 0 °C to RT; (s) (i) 2,6-lutidine, 120°C; (ii) TBSCl, imidazole, DMAP cat., DMF, 0 °C to RT, 77% (over four steps, dr >20:1); (t) Me_3_SiI, EtOH, CH_2_Cl_2_, 0°C to RT, 89%; (u) Ag_2_O, MeCN, reflux, 92%; (v) trimethylsilylacetylene, *n*BuLi, Et_2_AlCl, toluene, −35°C to 0 °C, 91%; (w) Ni­(OAc)_2_·4H_2_O, NaBH_4_, H_2_ (1 atm), ethylenediamine, EtOH, RT, 86% [2.26 g scale]; (x) TMSCl, Et_3_N, CH_2_Cl_2_, 0 °C to RT, 96%; (y) MeNH­(OMe)·HCl, *i*PrMgCl, THF, −20°C, 88%; (z) MeMgBr, Et_2_O, 0 °C, 85%.

Epoxide **2** also served the preparation of a **C5** surrogate upon opening with vinylmagnesium bromide,^42^ followed by cross metathesis of the resulting homoallylic alcohol **9** with methyl acrylate,^23^ which afforded enoate **10** with excellent geometric purity (*E:Z* > 20:1). The derived mesylate was subjected to Sharpless asymmetric dihydroxylation^43^ and the resulting diol was exposed to 2,6-lutidine at high temperature to cause an intramolecular S_N_2 reaction resulting in ether ring formation. Traces of minor diastereomers were separated after TBS-protection of the remaining hydroxy group to give the desired building block **11** in pure form on multigram scale.

The alkenylsilane **18** needed for the envisaged intramolecular silyl-modified Sakurai (ISMS) reaction^30^ was prepared from commercial (*S*)-β-hydroxy-γ-butyrolactone **12**, which was first converted into epoxybutanoate **14** by ring cleavage with TMSI followed by oxirane formation on treatment with Ag_2_O.^44^ Exposure of **14** to metalated TMS-acetylene gave the literature-known alkyne derivative **15**,^45^ which underwent (*Z*)-selective semireduction on hydrogenation over freshly prepared P2-nickel.^46,47^ Equally uneventful was the elaboration of compound **16** thus formed into the methyl ketone **18**. Importantly, all steps of this route were nicely scalable.

The fusion of compounds **11** and **18** was achieved by an ISMS reaction promoted by TMSOTf in CH_2_Cl_2_ at low temperature (Scheme 3).^30^ The formation of the desired dihydropyran ring proceeded well but with modest diastereoselectivity (dr ≈ 3:1) and came along with partial cleavage of the TBS-ether; therefore, the crude product had to be reacted with TBSCl and imidazole in DMF. The catalytic dihydroxylation of the major product **20** proceeded smoothly, whereas the seemingly trivial persilylation of the resulting diol proved troublesome. Only the use of TBSOTf and 2,6-lutidine was effective, although the methyl ketone was partly transformed under these conditions into a mixture of silyl enol ethers; upon brief exposure of the crude material to aq. HCl, however, this functionality was cleaved and product **22** released in excellent yield.

**Scheme 3:**
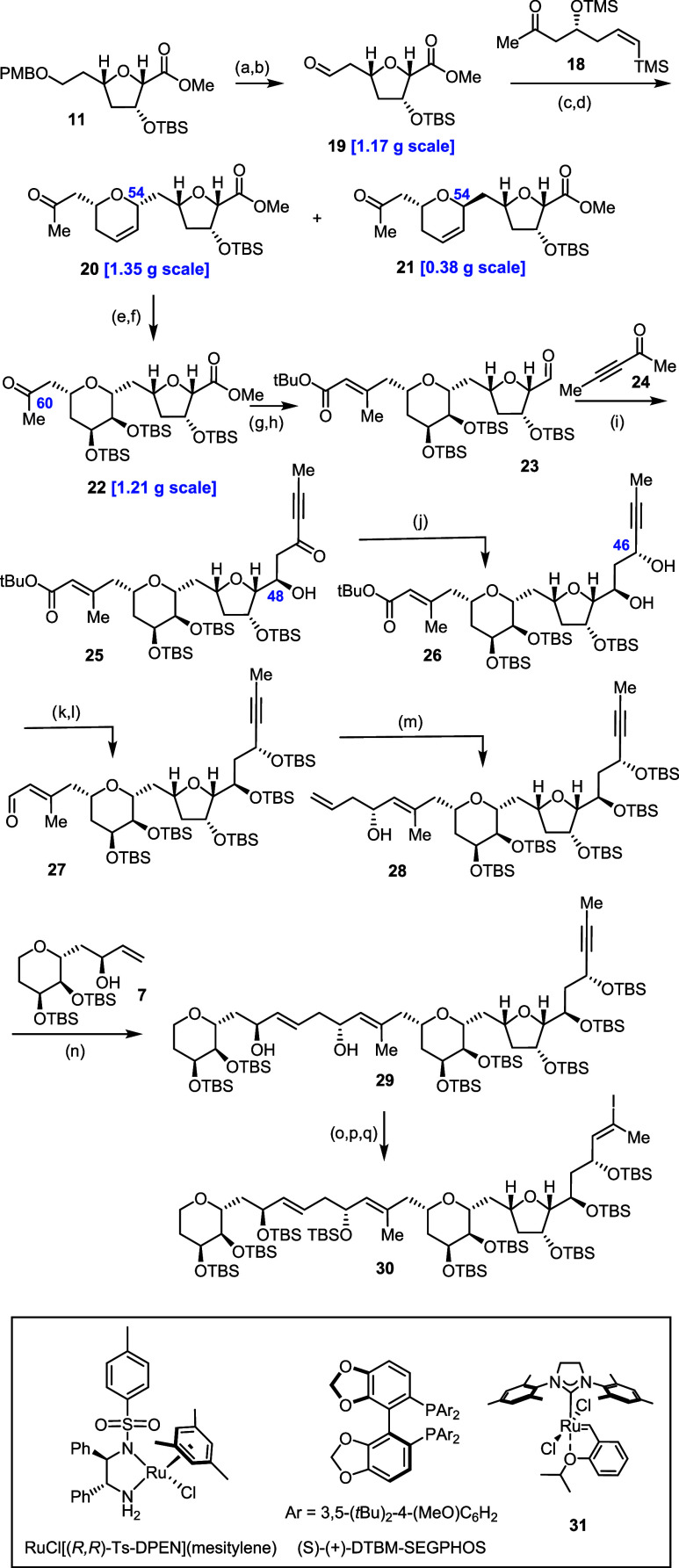
Coupling of Compounds **11** and **18** ^a^

a Reagents and Conditions: (a) DDQ, CH_2_Cl_2_/H_2_O, RT, quant. [1.57 g scale] (b) Dess-Martin periodinane, NaHCO_3_, CH_2_Cl_2_, 0 °C to RT, 77%; (c) **18**, TMSOTf, CH_2_Cl_2_, −78 °C; (d) TBSCl, imidazole, DMAP cat., DMF, 0 °C to RT, **20** (70%, over two steps) + **21** (20%); (e) OsO_4_ (5 mol %), NMO, THF/H_2_O, RT, 80% (dr = 9:1 [1.07 g scale]); (f) (i) TBSOTf, 2,6-lutidine, CH_2_Cl_2_, −78 °C to 0 °C; (ii) aq. HCl, RT, 98%; (g) *t*BuOOCCH_2_P­(O)­(OEt)_2_, NaH, LiBr, THF, 0 °C to RT, 68% (*E*-isomer [681 mg scale]) + 25% (*Z*-isomer); (h) Dibal-H, CH_2_Cl_2_, −95 °C; (i) **24**, CuBF_4_·(MeCN)_4_ (5 mol %), (*S*)-(+)-DTBM-SEGPHOS (5 mol %), F_3_CCH_2_OLi (5 mol %), CF_3_CH_2_OH (40 mol %), THF, −40°C, **25** (60% (83% brsm) over two steps, [474 mg scale]) + 48-*epi*-**25** (not shown; 12%); (j) RuCl­[(*R,R*)-Ts-DPEN]­(mesitylene) (5 mol %), HCOOH, Et_3_N, CH_2_Cl_2_, RT, **26** (68% [352 mg scale]) + 46-*epi*-**26** (16%); (k) TBSOTf, 2,6-lutidine, CH_2_Cl_2_, −78 °C, 98% [440 mg scale]; (l) (i) Dibal-H, toluene, −78 °C; (ii) Dess-Martin periodinane, NaHCO_3_, CH_2_Cl_2_, 0 °C to RT, 79% (over two steps) [322 mg scale]; (m) allylmagnesium bromide, (+)-Ipc_2_BOMe, Et_2_O, −95 °C, 82% [188 mg scale]; (n) **7**, **31** (5 mol %), CH_2_Cl_2_, RT, 73% [174 mg scale]; (o) TBSOTf, 2,6-lutidine, CH_2_Cl_2_, −78 °C, 95% [193 mg scale]; (p) *n*Bu_3_SnH, [(P­(*o*-tolyl)_3_)_2_PdCl_2_] (10 mol %), THF, RT; (q) I_2_, CH_2_Cl_2_, 0 °C, 82% (over two steps [89 mg scale]).

As mentioned above, the exact sequence of the projected two-directional chain extension reactions that had to follow at this point was not entirely clear at the outset. After some experimentation, we noticed that the olefination of the C60 carbonyl group had to precede the aldol chemistry intended to set the stereogenic center at C48 for stability reasons. Compound **22** was hence subjected to a high-yielding HWE reaction using NaH in combination with LiBr in THF as the optimal base.^48^ After separation of the resulting isomer mixture (*E:Z* = 2.7:1), the major product was treated with Dibal-H at low temperature, which left the *tert*-butyl ester of the enoate intact while reducing the methyl ester at the other terminus. The resulting crude aldehyde **23** was used in an asymmetric copper-catalyzed direct aldol reaction with pent-3-yne-2-one (**24**);^49^ with (*S*)-(+)-DTBM-SEGPHOS as the ligand and lithium 2,2,2-trifluoroethoxide as cocatalyst,^50^ this transformation furnished the desired product **25** in 60% isolated yield (83% brsm, dr ≈ 5:1) on 474 mg scale. This outcome was far superior to that observed in all attempts at Cram-chelate controlled addition of the lithium enolate derived from **24** as well as in a series of Mukaiyama-type aldol reactions.^51^ A minor complication was that the configuration of the newly set secondary −OH group at C48 could not be rigorously determined at this point; gratifyingly, any uncertainty was resolved after asymmetric reduction of the C46 carbonyl group by Noyori transfer hydrogenation with RuCl­[(*R,R*)-TsDPEN]­(mesitylene) as catalyst and HCOOH/Et_3_N as reductant.^52^ The resulting major product **26** was shown by the modified Mosher method to be (*R*)-configured at C46;^53^ the relative 1,3-*anti* configuration of the diol subunit could be deduced from the spectral data after conversion into the corresponding isopropylidene acetal (for details, see the Supporting Information).^54^

With a robust route to **26** in place, the yet missing attachment of the H-ring was tackled. After the two hydroxy groups had been capped with TBS groups, the *tert*-butyl ester was reduced with Dibal-H in toluene and the resulting primary −OH group oxidized with Dess-Martin periodinane.^55^ The resulting enal **27** succumbed to a reagent controlled asymmetric allylation on treatment with the allylborane reagent generated in situ from (+)-Ipc_2_B­(OMe) (derived from (−)-α-pinene)^56^ and allylmagnesium bromide.^57^ When performed at −95 °C in Et_2_O as the solvent, a single isomer was formed in high yield, the predicted (62*R*)-configuration of which was confirmed by modified Mosher ester analysis.^53^ This compound underwent cross metathesis with the terminal alkene derivative **7** in the presence of the ruthenium complex **31** as catalyst;^58^ as expected, the alkyne entity of **28** did not interfere at all when the reaction was performed at ambient temperature. Subsequent silylation of the two hydroxy groups of product **29** thus formed was necessary prior to palladium catalyzed hydrostannylation of the triple bond;^59^ the resulting stannane was swiftly converted into the targeted alkenyl iodide **30** as a fully functional building block of type **C** in readiness for the assembly of benthol A.

### Final Substructure Verification

In analogy to the attempted authentications described in the accompanying paper for the two other big building blocks **A** and **B**,^17^ the advanced compound **29** representing the entire “southern” hemisphere of benthol A was subjected to a similar test. As it carried only TBS ethers, exposure to excess TBAF in THF sufficed to obtain the polyol **32** suitable for comparison with the natural product (Scheme 4).

**Scheme 4:**
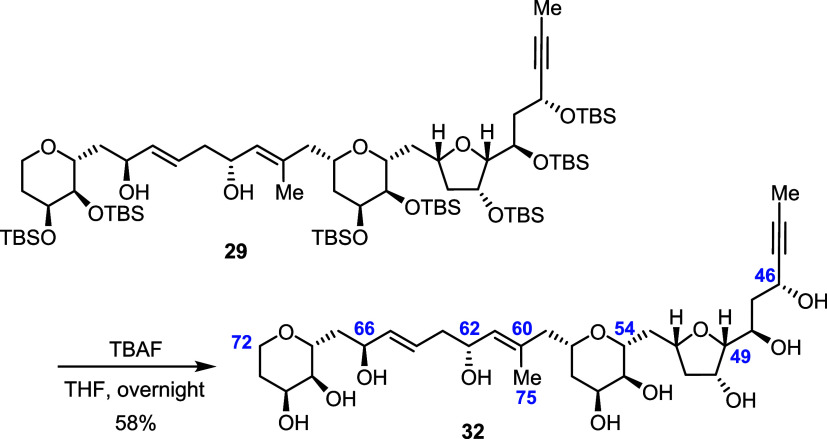
Preparation of Polyol Fragment **32** Representing the Southern Sector of Benthol A for Comparison with the Natural Product

It was gratifying to see that the ^13^C NMR shifts of compound **32** corresponded very well to those of benthol A (Figure 1).^3^ Termini apart, the shift differences (Δδ_C_) were ≤0.1 ppm for the entire range from C50 to C72/C75. Based on this congruence, we were confident that the entire southern perimeter is matching and that the C40-stereocenter is hence almost certainly the only site in benthol A that had been misassigned by the isolation team.

**Figure 1:**
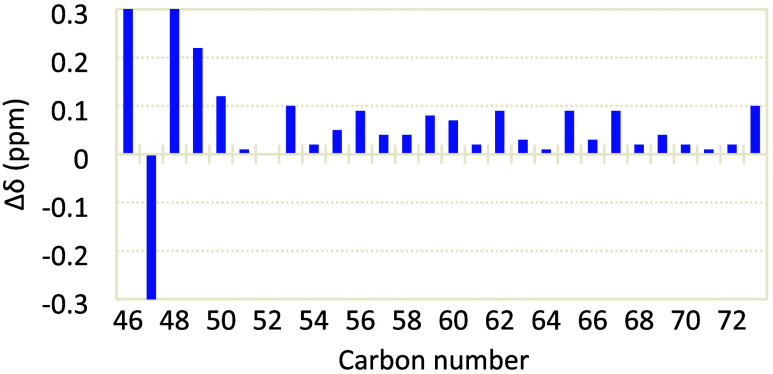
Graphical representation of the observed ^13^C NMR chemical shift differences (Δδ_C_, ppm; [D_4_]-MeOH) between the C46–C72/C75 sector of authentic benthol A and polyol **32**.

### Scouting the Endgame

With good amounts of all key building blocks in hand, we had to consider the best order in which to combine them en route to benthol A; the general retrosynthetic scheme had deliberately left this aspect open. Our initial preference for the sequence **A** + **B** → **AB** + **C** → benthol A reflected mostly the size of the fragments to be combined but disregarded a subtle but, in the end, critically important chemoselectivity aspect. Specifically, hydroboration of compound **33** representing the entire northern **AB** domain of benthol A (for the preparation of this compound, see the Supporting Information) with [9-H-9-BBN]_2_ followed by attempted B-alkyl-Suzuki coupling of the organoboron intermediate thus formed with the model alkenyl iodide **34** failed to afford the desired product (Scheme 5). Inspection of the crude material showed that the characteristic ^1^H NMR signals of the *exo*-methylene group branching off the C10 position on the B-ring were missing. This result suggested that a selective hydroboration of the terminal C42–C43 alkene did not take place under the chosen conditions; rather, the *exo*-methylene group on the B-ring seems to be as (or even more) reactive. A literature report corroborates this notion, although the data refer to very simple olefins only: thus, allyl acetate (as well as simple allyl halides) has been shown to react with [9-H-9-BBN]_2_ considerably more slowly than 2-methyl-1-pentene as a distantly related analogue of the *exo*-methylene group in **33**.^60^ Taken together, this evidence implied that the two bigger pieces **B** and **C** must be combined before the **A**-sector comprising the apparently noninnocent *exo*-olefin can be attached and the backbone of benthol A be completed.

**Scheme 5:**
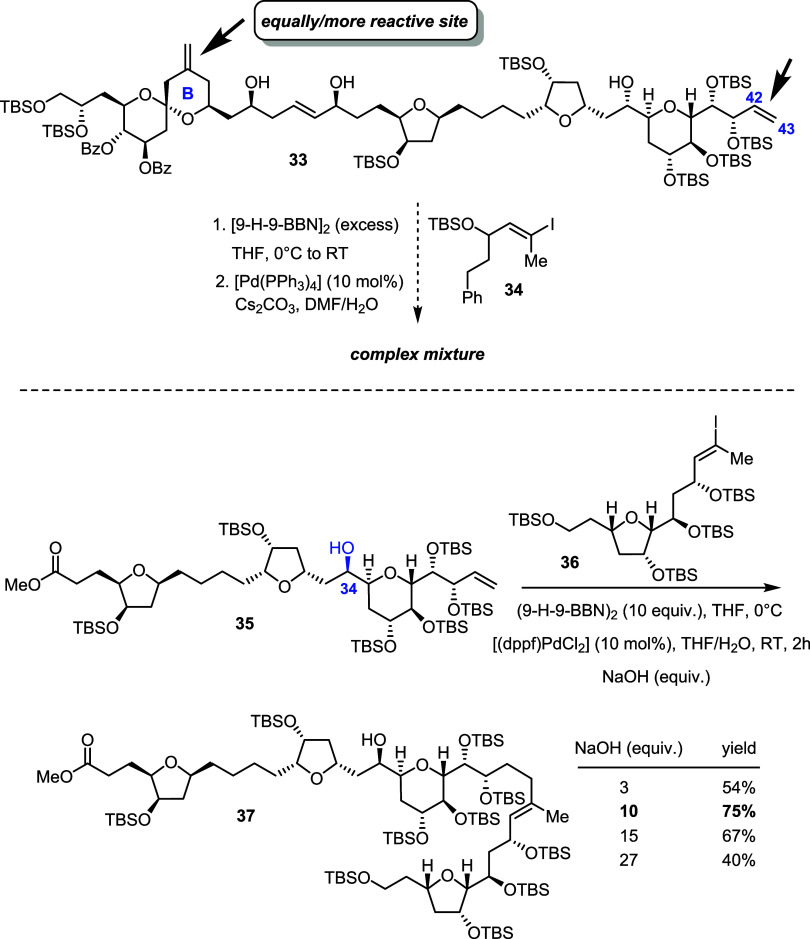
Intelligence Gathering

The conditions for the crucial B-alkyl-Suzuki reaction were also studied in some detail before the actual **B**/**C** merger was executed. Details apart, the results summarized in Scheme 5 are representative (Scheme 5, bottom; see also the Supporting Information). Thus, a large excess of [9-H-9-BBN]_2_ in THF as the solvent proved necessary to ensure quantitative hydroboration of the terminal alkene in **35** as the model compound (it is the otherwise useless C34 epimer of the actual fragment **B**/**B**′); unreacted borane was then destroyed upon addition of aqueous base, the choice of which proved to have a strong impact on the outcome of the reaction. Although Cs_2_CO_3_ in THF/DMF/H_2_O in combination with [Pd­(PPh_3_)_4_] has been used with considerable success in similarly advanced total syntheses projects and was therefore our first choice,^59,61^ the yield of product **37** was rather low even after long reaction times (≤42%, 20 h), which did not encourage the use of this recipe for the coupling of the precious fragments **B** and **C** themselves. In line with earlier advanced applications of B-alkyl-Suzuki couplings in our laboratory, which had taught us that the optimal base is strongly case-dependent (NaOMe, NaOAc, Ba­(OH)_2_, K_3_PO_4_),^62–66^ an assortment of candidates was screened and aqueous NaOH in combination with [(dppf)­PdCl_2_] identified as being most effective in the present setting, provided the equivalents of the base were carefully controlled.^36,67,68^

### Nominal and Actual Benthol A

For the acquired intelligence, we felt well prepared for the actual end game. It commenced with the hydroboration of compound 40-*epi*-**38** with excess [9-H-9-BBN]_2_ in THF followed by palladium catalyzed coupling of the alkylboron reagent thus formed with alkenyl iodide **30** under the optimized conditions outlined above to give compound 40-*epi*-**39** in 55% yield (66% brsm) (Scheme 6). The methyl ester was transformed into the corresponding arylthiol ester 40-*epi*-**40** on treatment with preformed [(*p*-tolylS)_3_Al] in toluene,^69^ which was instantly used in the next step without rigorous purification. The carbon skeleton of benthol A was completed by palladium-catalyzed, copper mediated Liebeskind coupling reaction with freshly prepared alkenyl stannane **41** ^17^ at slightly elevated temperature, which furnished enone 40-*epi*-**42** in good yield, provided that freshly prepared copper diphenylphosphinate was used as the promoter.^70^ The proclivity of the *exo*-methylene group decorating the B-ring to react with boron reagents surfaced again in the subsequent CBS-reduction of the carbonyl group,^41^ which had to be performed in fairly concentrated THF solution with catecholborane at low temperature instead of B_2_H_6_ as the terminal reducing agent in order to keep this olefin intact. All it took then was to subject the resulting product 40-*epi*-**43** to global deprotection, which proceeded smoothly in just two operations as only two benzoates and 17 TBS-ethers were present at this point. Thus, saponification of the esters with K_2_CO_3_ in THF/MeOH was followed by treatment of the resulting crude product with excess TBAF in THF. Upon attempted purification, however, the unreported yet exceptional acid-sensitivity of the final compound was noticed in that attempted regular flash chromatography entailed partial decomposition of the sample. Although no rigorous assignment of the resulting products was carried out, the spectral data suggested that the contact with silica gel (or, alternatively, Dowex resin) had sufficed to damage the allylic alcohol at C46, leading to the formation of the rearranged product **44** as well as of a diene tentatively assigned as **45**. Therefore, the workup had to be modified in that the crude product left after the TBAF deprotection was first extensively washed with *n*-hexane/CH_2_Cl_2_ (3:1) to remove most of the tetra-*n*-butyl ammonium salts before subjecting the residue to purification via reversed phase flash chromatography. This workflow furnished pure samples of compound 40-*epi*-**1** (containing traces of the C18 epimer formed in the CBS reduction step), which we had predicted to represent actual benthol A. Very much in line with our expectations, the NMR spectra of the synthetic material perfectly matched the reported data of this conspicuous marine natural product^3^ as one can see from both the tabular as well as the extensive visual comparisons contained in the Supporting Information. Figure 2 (top) shows, pars pro toto, the perfect congruence of the ^13^C NMR chemical shifts (Δδ_C_ ≪ 0.1 ppm).

**Figure 2:**
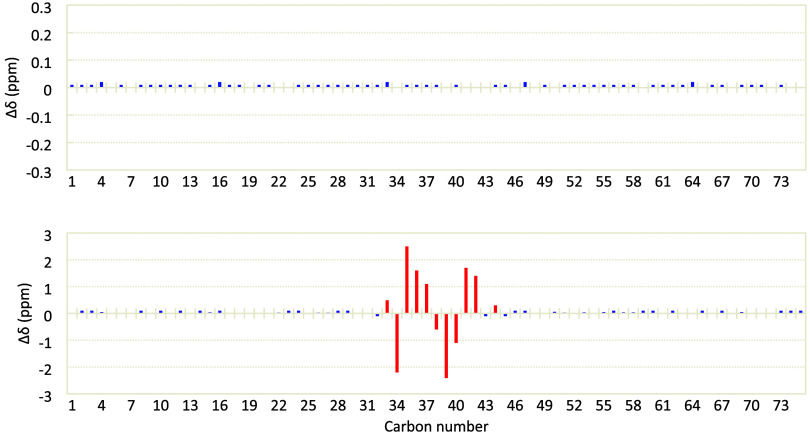
Graphical representation of the ^13^C NMR chemical shift differences (Δδ_C_, ppm; [D_4_]-MeOH) between authentic benthol A and synthetic 40-*epi*-**1** (top) as well as **1** (bottom)

**Scheme 6:**
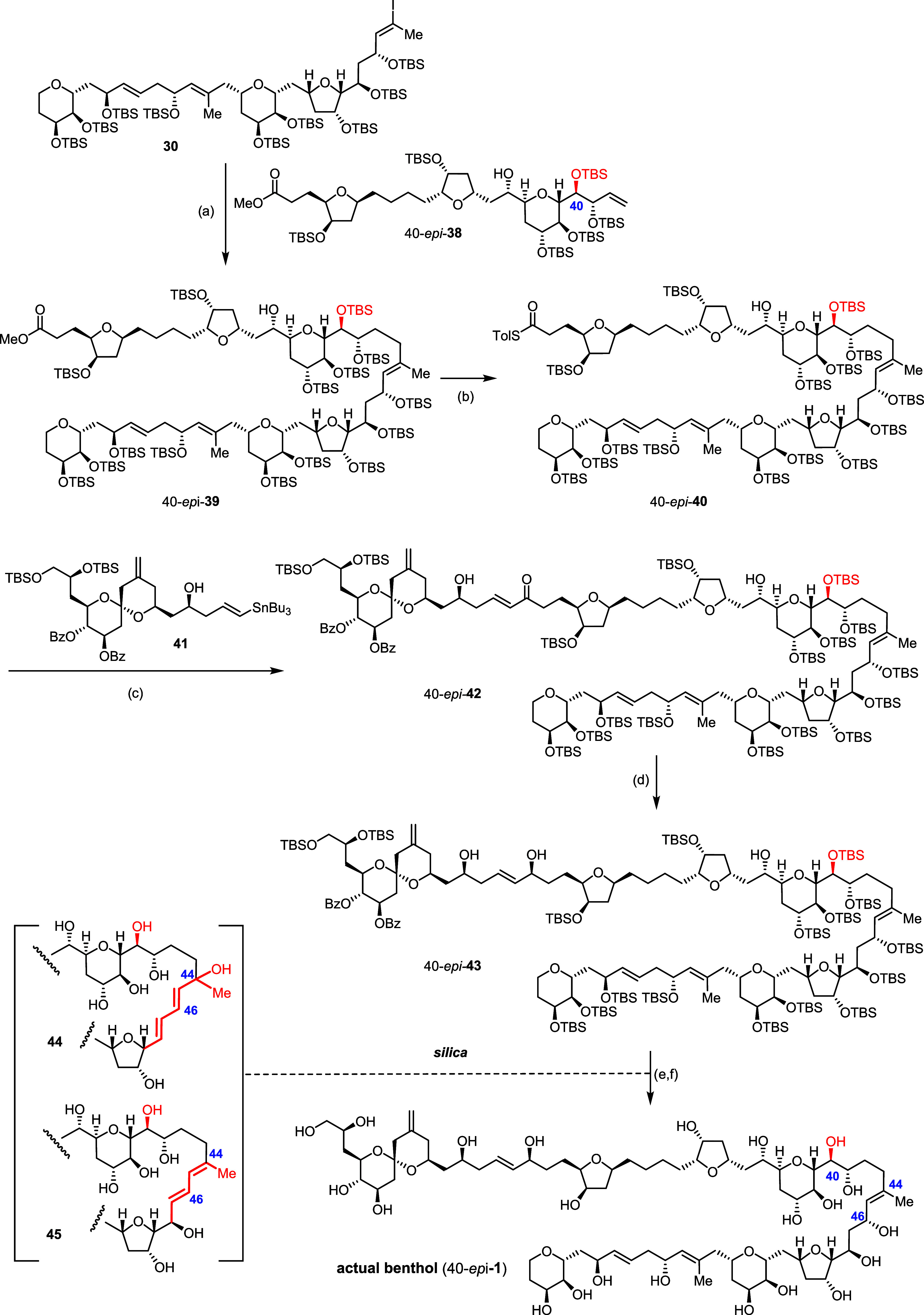
Synthesis of Actual Benthol A (40-*epi*-**1)** ^a^

a Reagents and Conditions: (a) (i) 40-*epi*-**38**, [9-H-9-BBN]_2_, THF, 0 °C to RT; (ii) **30**, [(dppf)­PdCl_2_] (10 mol %), NaOH (10 equiv), THF/H_2_O (5:1), RT, 55% (66% brsm); (b) (*p*-tolylS)_3_Al, toluene, RT, 77%; (c) [Pd_2_(dba)_3_]·CHCl_3_ (10 mol %), P­(2-furyl)_3_ (80 mol %), Ph_2_P­(O)­OCu, THF/hexanes (1:1), 50°C, 86%; (d) (*R*)-CBS catalyst, catecholborane, THF, −78 °C to −20 °C, 93%; (e) K_2_CO_3_, THF/MeOH, RT; (f) TBAF, THF, 0 °C to RT, 92% (over two steps).

To dispel any remaining doubts, the same sequence of reactions was applied to make nominal benthol A (**1**), starting off with the hydroboration of fragment **38** (Scheme 7). Once again, the final compound proved acid sensitive and hence mandated processing by the modified workup procedure outlined above. This technical issue notwithstanding, pure samples were obtained, the NMR spectra of which clearly digress from those of benthol A.^3^ Specifically, the ^13^C NMR chemical shifts of all C-atoms from C33 to C44 differ from those of the natural product, with deviations of up to 2.5 ppm (Figure 2, bottom).

**Scheme 7:**
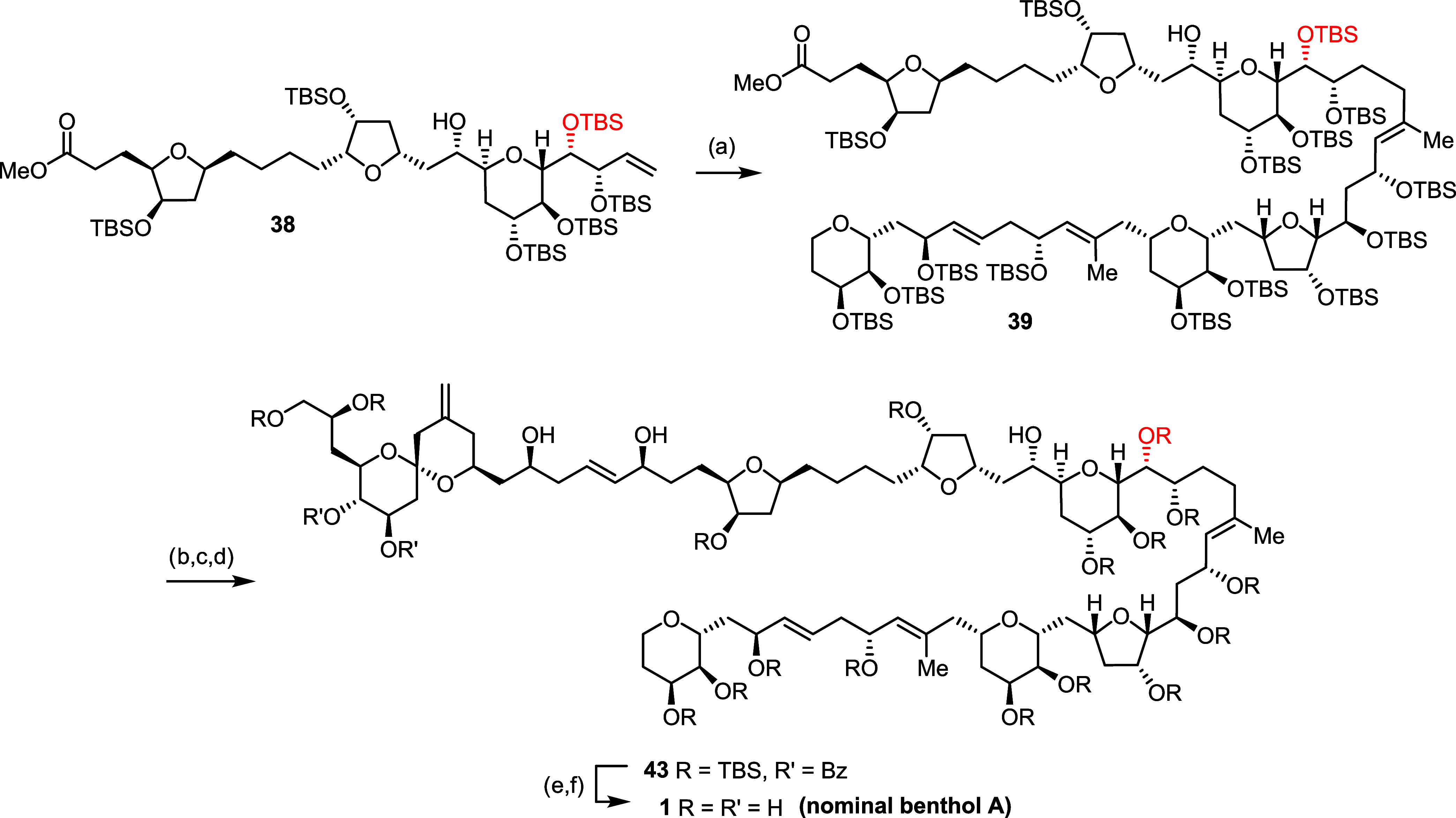
Synthesis of Nominal Benthol A (**1**)­^a^

a Reagents and Conditions: (a) (i) **38**, [9-H-9-BBN]_2_, THF, 0 °C to RT; (ii) **30**, [(dppf)­PdCl_2_] (10 mol %), NaOH (10 equiv), THF/H_2_O (5:1), RT, 55% (75% brsm); (b) (*p*-tolylS)_3_Al, toluene, RT, 89%; (c) **41**, [Pd_2_(dba)_3_]·CHCl_3_ (10 mol %), P­(2-furyl)_3_ (80 mol %), Ph_2_P­(O)­OCu, THF/hexanes (1:1), 50 °C, 84%; (d) (*R*)-CBS catalyst, catecholborane, THF, −78 °C to −20 °C, 70%; (e) K_2_CO_3_, THF/MeOH, RT; (f) TBAF, THF, 0°C to RT, 74% (over two steps).

## Conclusions

In summary, the first total synthesis of benthol A was achieved, providing access to this supercarbon-chain compound endowed with potent antiplasmodial and antiviral activity. The total synthesis comes along with a subtle structure revision, in that it is unambiguously shown that the configuration of the secondary −OH group at C40 had been misassigned by the isolation team. This revision bears implications because it corrects an assignment that had solely been based on computational data and a statistical analysis thereof, since massive signal overlap had prevented the use of any spectroscopic methods; moreover, the position in question could not be derivatized as Mosher ester either. In the present case, the computer-based assignment of the configuration of the C40 stereocenter had resulted in an exceptionally high score of 99.93%, which was, however, in the end misleading. This finding implies that the method still finds limitations, especially when applied to flexible compounds populating a very large conformational space; moreover, disregarding the MeOH solvent might have been misleading in case of a polyol that entertains numerous H-bonds with the protic medium.^71–74^

Gratifyingly, the misassignment did not come as an unpleasant surprise at the very end of this massive project, but had already been foreshadowed by the inspection of the key building blocks and the preparation of variants thereof for comparison. This detective work allowed us to predict with high confidence that only one of the 35 stereocenters adorning the backbone of benthol A needed revision and made it possible to localize the site of error with a high degree of certainty.

For the multiconvergent character, the total synthesis itself has a longest linear sequence (LLS) of 32 steps (starting from l-aspartic acid via compound **30** as fragment **C** surrogate to 40-*epi*-**1**), which is deemed rather short given the stereochemical splendor of this target; the overall yield (LLS) is ≈ 1.2% and the total step count is 93. To better assess these numbers, we refer to the synthetic efficiency index (SE_LLS_) introduced by Oishi, which is calculated by dividing the molecular weight of the compound (1506.83) by the number of steps in the LLS;^6^ in the present case, this results in a SE_LLS_ ≈ 47.1. For comparison: a ten-step synthesis of a hypothetical compound with a MW = 471 would give the same number. Particularly noteworthy transformations en route to nominal (**1**) as well as actual benthol A (40-*epi*-**1**) are (i) the use of a gold-catalyzed spiroacetalization, the regiochemical course of which could be controlled by proper choice of the neighboring protecting groups, (ii) the carboxylation of a fragile anomeric organolithium reagent, (iii) the remarkably efficient addition of densely functionalized but unstabilized diazo derivatives to equally elaborate aldehyde partners (Buchner-Curtius-Schlotterbeck reaction), (iv) a newly devised *anti*-oxyallylation reaction, (v) an advanced application of Morken’s platinum catalyzed asymmetric formal dihydroxylation of a terminal alkene, (vi) an enabling asymmetric copper-catalyzed direct Shibasaki aldol reaction of an acetylenic methyl ketone, (vii) the entirely chemoselective cross metathesis serving the attachment of the H-ring, whereby an equally conceivable enyne metathesis could be avoided, (viii) an intricate B-alkyl-Suzuki reaction for the coupling of the two biggest segments, and (ix) the Liebeskind coupling of highly elaborate thioester derivatives with an equally precious alkenylstannane as the final fragment coupling event, to name but a few. The synthetic endeavor has furnished enough material to enable a round of in-depth biological testing of benthol A and its C40-epimer; moreover, we plan to take further advantage of the flexibility of the chosen approach for mapping the pharmacophore encoding the significant antimalarial activity of this lead compound. Pertinent results along these lines will be disclosed in due course.

## Supplementary Material



## Notes

Experimental section including characterization data, and copies of spectra of new compounds (PDF)
