# Supplementary material for: Total Synthesis of Benthol A: The Nominal and the Actual Natural Product
Source: J Am Chem Soc. 2026 Jul 16;148(29):30731–43. doi: 10.1021/jacs.6c05583 (PMC13426268; doi:10.1021/jacs.6c05583)
Supplement: Supplementary file 1 [file ja6c05583_si_001.pdf]

SUPPORTING INFORMATION

Total Synthesis of Benthol A:
The Nominal and the Actual Natural Product

Guanghao Huang,[+] Andrea Tomio,[+] Thomas Varlet, Conny Wirtz, and Alois Fürstner*

Max-Planck-Institut für Kohlenforschung, 45470 Mülheim/Ruhr, Germany

E-Mail: fuerstner@kofo.mpg.de

[+] These authors contributed equally

Table of Contents

General Information	S2
The Sub-Fragments	S3
Assembly of Fragment C	S15
Final Substructure Verification	S31
Intelligence Gathering	S36
A/B-Fragment Coupling	S36
Model Studies on B/C-Fragment Coupling	S40
Completion of the Total Synthesis of Nominal Benthol A	S42
Completion of the Total Synthesis of Actual Benthol A	S55
Visual Comparison of the NMR Spectra of Authentic Benthol A with those of Synthetic 40-*epi*-**1**	S68
Copies of Spectra of New Compounds	S82
References	S215

General Information

Unless stated otherwise, all reactions were carried out in flame-dried glassware using anhydrous solvents under argon atmosphere.

The solvents were purified by distillation over the indicated drying agents and were transferred under argon: THF, Et_2O (Mg/anthracene); acetonitrile, 2,6-lutidine, CH_2Cl_2, 1,2-DCE, nitromethane (CaH_2); toluene (Na/K alloy); methanol (Mg, stored over MS 3 Å). DMSO, DMF, Et_3N, pentane, and pyridine were dried by an adsorption solvent purification system based on molecular sieves.

Thin layer chromatography (TLC): Macherey-Nagel pre-coated plates (normal phase: POLYGRAM®SIL/UV254; reversed phase: ALUGRAM®SILCN/UV254). Detection was achieved under UV-light (254 nm) and by staining with either acidic *para*-anisaldehyde, cerium ammonium molybdate, or basic $KMnO_4$ solution. Flash chromatography: Merck silica 60 (40–63 µm) (normal phase) or Carl Roth silica gel 60 cyano (35-70 µm) (reversed phase) with pre-distilled or HPLC grade solvents.

NMR: Spectra were recorded on Bruker AV 400, AV 500, AVIII 600, or AVneo 600 spectrometers in the indicated solvents; the 600 MHz spectrometers were equipped with Bruker BBO cryoprobes; chemical shifts (δ) are given in ppm relative to TMS, coupling constants (J) in Hz. All spectra were recorded at 25 °C, unless stated otherwise. The solvent signals were used as references and the chemical shifts converted to the TMS scale ($CDCl_3$: δ_C = 77.16 ppm; residual ^{1}H: δ_H = 7.26 ppm; [D_4]-MeOH: δ_C = 49.00 ppm, residual ^{1}H: δ_H = 3.31 ppm; CD_2Cl_2: δ_C = 53.84 ppm, residual ^{1}H: δ_H = 5.32 ppm; [D_6]-DMSO: δ_C = 39.52 ppm, residual ^{1}H: δ_H = 2.50 ppm). Multiplicities are indicated by the following abbreviations: s: singlet, d: doublet, t: triplet, q: quartet, hept: heptet, m: multiplet, br. s: broad singlet. ^{13}C NMR spectra were recorded in ^{1}H-decoupled manner and the values of the chemical shifts are rounded to one decimal point. Signal assignments were established using HSQC, HMBC, COSY, NOESY and other 2D experiments.

IR: Spectra were recorded on an Alpha Platinum ATR instrument (Bruker), wave numbers ($\tilde{\nu}$) in cm^{-1}.

MS (ESI-MS): Finnigan MAT 8200 (70 eV), ESI-MS: ESQ3000 (Bruker), accurate mass determinations: Bruker APEX III FTMS (7 T magnet) or Mat 95 (Finnigan).

Optical rotations ($[\alpha]_D^{20}$) were measured with an A-Krüss Optronic Model P8000-t polarimeter at a wavelength of 589 nm.

Molecular sieves were activated at 150 °C for 24 h under high vacuum (1×10^{-3} mbar) and stored under argon.

Unless stated otherwise, commercially available compounds (ABCR, Alfa Aesar, Aldrich, BLDPharm, TCI, Strem Chemicals, ChemPUR) were used as received. The preparation of multigram amounts of epoxide **2** follows a literature route[1] and is described in the accompanying paper.

The Sub-Fragments

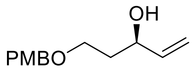

Compound 3. In a flame-dried flask, trimethylsulfonium iodide (5 eq., 72.03 mmol, 14.70 g) was suspended in THF (220 mL), The suspension was cooled to 0 °C (ice bath) before *n*-BuLi (1.6 M solution in hexanes, 40.5 mL, 64.82 mmol) was added dropwise over 10 min. After 10 min of vigorous stirring at 0 °C, a solution of epoxide **2** (1.0 eq., 14.40 mmol, 3.0 g) in THF (20 mL) was added dropwise and the resulting mixture was stirred at 0 °C for 1 h before the reaction was quenched with sat. aq. NH_4Cl (100 mL). The aqueous layer was extracted with CH_2Cl_2 (3 x 100 mL). The combined organic phases were dried over $MgSO_4$, filtered and concentrated under reduced pressure. The residue was purified by flash chromatography on silica (hexanes/EtOAc, 9:1 to 7:3) to afford the title compound was a colorless oil (2.99 g, 93%). $[\alpha]_D^{20}$ = –9.5 (c = 1.5, $CHCl_3$); ^{1}H NMR (400 MHz, $CDCl_3$): δ = 7.30 – 7.18 (m, 2H), 6.93 – 6.83 (m, 2H), 5.87 (ddd, *J* = 17.2, 10.4, 5.5 Hz, 1H), 5.27 (dt, *J* = 17.2, 1.6 Hz, 1H), 5.10 (dt, *J* = 10.4, 1.5 Hz, 1H), 4.45 (s, 2H), 4.33 (s, 1H), 3.81 (s, 3H), 3.69 (ddd, *J* = 9.3, 6.4, 4.7 Hz, 1H), 3.61 (ddd, *J* = 9.4, 7.2, 4.6 Hz, 1H), 2.93 – 2.81 (m, 1H), 1.94 – 1.73 (m, 2H) ppm; ^{13}C NMR (101 MHz, $CDCl_3$): δ = 159.4, 140.7, 130.2, 129.5 (2C), 114.5, 114.0 (2C), 73.1, 72.2, 68.2, 55.4, 36.4 ppm; IR (film) $\tilde{\nu}$ = 3428, 2938, 2862, 2838, 1612, 1513, 1247, 1091, 1033, 821 cm^{-1}; HRMS (EI): *m/z:* calcd. for $C_{13}H_{18}O_3$ [M^+]: 222.12505, found: 222.12492.

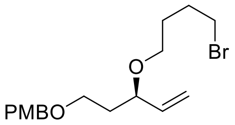

Compound S1. In a flame-dried flask, alcohol **3** (1.0 eq. 13.45 mmol, 2.99 g) was dissolved in toluene (135 mL). Tetrabutylammonium bromide (434 mg, 1.35 mmol, 10 mol%) and 1,4-dibromobutane (5 eq., 67.26 mmol, 8.0 mL) were added, followed by crushed KOH (2.26 g, 40.35 mmol). After 2 h, additional KOH (2.26 g, 40.35 mmol) was added and the mixture stirred overnight (14 h). At this point, a third batch of KOH (2.26 g, 40.35 mmol) was introduced and stirring continued for 2 h before yet another batch of KOH (2.26 g, 40.35 mmol) as well as additional 1,4-dibromobutane (1.60 mL, 13.45 mmol) were introduced. After additional 3 h, a final batch of KOH (2.26 g, 40.35 mmol) and more by 1,4-dibromobutane (3.20 mL, 26.90 mmol) were added. After stirring for an additional 3 h, TLC indicated complete conversion of the substrate. The mixture was cooled to 0 °C (ice bath) and water (100 mL) was added and the aqueous layer was separated and extracted with methyl *tert*-butyl ether (3 x 50 mL). The combined organic phases were washed with brine (100 mL) and dried over Na_2SO_4 before they were filtered and concentrated under reduced pressure. The residue was purified by flash chromatography on silica (hexanes/EtOAc, 100:0 to 4:1) to afford the title product as a colorless oil (4.45 g, 92%). $[\alpha]_D^{20}$ = +10.7 (c = 1.9, $CHCl_3$); ^{1}H NMR (400 MHz, $CDCl_3$): δ = 7.29 – 7.21 (m, 2H), 6.92 – 6.83 (m, 2H), 5.66 (ddd, *J* = 17.2, 10.3, 7.6 Hz, 1H),

5.23 – 5.12 (m, 2H), 4.42 (d, *J* = 1.3 Hz, 2H), 3.86 – 3.75 (m, 4H), 3.60 – 3.44 (m, 3H), 3.41 (t, *J* = 6.8 Hz, 2H), 3.25 (dt, *J* = 9.5, 6.3 Hz, 1H), 1.97 – 1.88 (m, 2H), 1.87 – 1.80 (m, 1H), 1.79 – 1.71 (m, 1H), 1.71 – 1.63 (m, 2H) ppm; ^{13}C NMR (101 MHz, $CDCl_3$): δ = 159.3, 139.1, 130.8, 129.5 (2C), 116.8, 113.9 (2C), 78.3, 72.8, 67.6, 66.3, 55.4, 35.9, 33.9, 29.9, 28.6 ppm; IR (film) $\tilde{\nu}$ = 3002, 2939, 2861, 1612, 1586, 1513, 1441, 1247, 1173, 1094, 822 cm^{-1}; HRMS (ESI): *m/z:* calcd. for $C_{17}H_{25}BrO_3Na$ $[M+Na]^+$: 379.08794, found: 379.08811.

Compound 4. In a flame-dried flask, 18-crown-6 (329 mg, 1.25 mmol, 10 mol%) was solubilized in

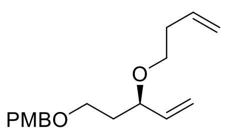

toluene (115 mL). A solution of bromoalkane **S1** (4.45 g, 12.45 mmol) in toluene (10 mL) was added and the solution cooled to 0 °C (ice bath). *tert*-BuOK (2.80 g, 24.91 mmol) was added in one portion and stirring continued at 0 °C for 10 min before removing the ice bath. Upon completion of the reaction (*ca*. 3.5 h), distilled water (100 mL) was added. The aqueous layer was extracted with methyl *tert*-butyl ether (3 x 50 mL), the combined organic phases were washed with brine (100 mL) and dried over Na_2SO_4 before being filtered and concentrated under reduced pressure. The residue was purified by flash chromatography on silica (hexanes/EtOAc, 100:0 to 95:5) to afford the title compound as a colorless oil (3.18 g, 92%). $[\alpha]_D^{20}$ = +17.7 (c = 1.7, $CHCl_3$); ^{1}H NMR (400 MHz, $CDCl_3$): δ = 7.31 – 7.21 (m, 2H), 6.92 – 6.85 (m, 2H), 5.81 (ddt, *J* = 17.0, 10.2, 6.7 Hz, 1H), 5.68 (ddd, *J* = 17.5, 10.3, 7.5 Hz, 1H), 5.23 – 5.12 (m, 2H), 5.11 – 4.99 (m, 2H), 4.42 (s, 2H), 3.87 – 3.78 (m, 4H), 3.61 – 3.45 (m, 3H), 3.29 (dt, *J* = 9.3, 6.9 Hz, 1H), 2.32 – 2.25 (m, 2H), 1.86 (ddt, *J* = 13.8, 8.0, 5.8 Hz, 1H), 1.75 (ddt, *J* = 13.6, 7.4, 5.7 Hz, 1H) ppm; ^{13}C NMR (101 MHz, $CDCl_3$): δ = 159.3, 139.1, 135.6, 130.8, 129.5 (2C), 116.7, 116.3, 113.9 (2C), 78.3, 72.8, 68.1, 66.4, 55.4, 35.9, 34.4 ppm; IR (film) $\tilde{\nu}$ = 3075, 3001, 2915, 2858, 1641, 1613, 1586, 1512, 1246, 1089, 1036, 920 cm^{-1}; HRMS (ESI): *m/z:* calcd. for $C_{17}H_{24}O_3Na$ $[M+Na]^+$: 299.16176, found: 299.16175.

Compound S2. A solution of Grubbs II catalyst (98 mg, 0.115 mmol, 1 mol%) in CH_2Cl_2 (570 mL) was

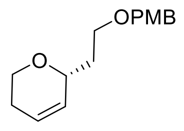

cooled to 0 °C (ice bath) and sparged with Ar for 15 min. This degassed solution was stirred at 40 °C (bath temperature) and a solution of diene **4** (3.18 g, 11.54 mmol) in CH_2Cl_2 (5 mL) was added dropwise over 5 min. Stirring was continued for 1 h at 40 °C before the solution was allowed to reach room temperature and all volatile materials were removed under reduced pressure. The residue was purified by flash chromatography on silica (hexanes/EtOAc, 100:0 to 4:1) to afford the title compound as a pale brown oil (2.73 g, 95%). $[\alpha]_D^{20}$ = +11.4 (c = 0.8, $CHCl_3$); ^{1}H NMR (400 MHz, $CDCl_3$): δ = 7.31 – 7.21 (m, 2H), 6.93 – 6.83 (m, 2H), 5.88 – 5.78 (m, 1H), 5.64 (ddt, *J* = 10.3, 2.4, 1.7 Hz, 1H), 4.44 (d, *J* = 1.9 Hz, 2H), 4.24 (tdd, *J* = 6.4, 3.2, 1.7 Hz, 1H), 3.94 (dddd, *J* = 11.3, 5.7, 2.7, 0.9 Hz, 1H), 3.80 (s, 3H), 3.70 – 3.51 (m, 3H), 2.31 – 2.19 (m, 1H), 1.99 – 1.88 (m, 1H), 1.86 – 1.77 (m, 2H) ppm; ^{13}C NMR (101 MHz, $CDCl_3$): δ = 159.3, 130.8, 130.5, 129.4 (2C), 124.9, 113.9 (2C),

72.9, 71.3, 66.8, 63.4, 55.4, 35.6, 25.5 ppm; IR (film) $\tilde{\nu}$ = 2918, 2853, 1613, 1513, 1463, 1247, 1181, 1084, 820 cm^{-1}; HRMS (EI): *m/z:* calcd. for $C_{15}H_{20}O_3$ [M$^+$]: 248.14070, found: 248.14054.

Compound 5. Dihydropyran **S2** (2.73 g, 11.0 mmol) was dissolved in THF (82 mL) and water (27 mL). *N*-Methylmorpholine N-oxide (3.86 g, 33.0 mmol) was added, followed by dropwise addition of a solution of osmium tetroxide (4% *w/w* in water, 7 mL, 1.1 mmol, 10 mol%). The flask was capped and stirring continued overnight (14 h). The reaction was quenched by addition of sat. aq. Na_2SO_3 solution. After vigorous stirring for 30 min, the solution was extracted with methyl *tert*-butyl ether (3 x 50 mL), the combined organic phases were washed with brine (50 mL) and dried over Na_2SO_4 before they were filtered and concentrated under reduced pressure. The residue was purified by flash chromatography (silica; hexanes/EtOAc, 1:1 to 1:4) to afford the title compound as a waxy solid (2.61 g, 84%). $[\alpha]_D^{20}$ = +34.0 (c = 1.0, $CHCl_3$); ^{1}H NMR (400 MHz, $CDCl_3$): δ = 7.28 – 7.21 (m, 2H), 6.91 – 6.86 (m, 2H), 4.48 (s, 2H), 4.43 (d, *J* = 3.0 Hz, 1H), 4.09 (q, *J* = 3.1 Hz, 1H), 3.80 (s, 3H), 3.78 – 3.70 (m, 1H), 3.69 – 3.61 (m, 2H), 3.59 – 3.49 (m, 2H), 3.32 (dt, *J* = 9.3, 3.0 Hz, 1H), 2.53 (s, 1H), 1.98 (dddd, *J* = 15.1, 9.5, 6.7, 3.8 Hz, 1H), 1.92 – 1.80 (m, 3H) ppm; ^{13}C NMR (101 MHz, $CDCl_3$): δ = 159.6, 129.7 (2C), 129.4, 114.1 (2C), 75.2, 73.2, 72.4, 67.3, 66.7, 62.0, 55.4, 35.0, 31.7 ppm; IR (film) $\tilde{\nu}$ = 3415, 2953, 2922, 2867, 1612, 1513, 1248, 1081, 1033, 820 cm^{-1}; HRMS (ESI): *m/z:* calcd. for $C_{15}H_{22}O_5Na$ [M+Na]$^+$: 305.13594, found: 305.13566.

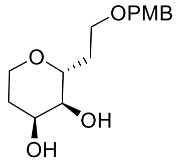

Compound S3. 2,6-Lutidine (3.22 mL, 27.63 mmol) was added at 0 °C (ice bath) to a solution of diol **5** (2.61 g, 9.21 mmol) in CH_2Cl_2 (90 mL), followed by the dropwise addition of TBSOTf (4.44 mL, 19.34 mmol). The solution was stirred at this temperature for 1 h before sat. aq. NH_4Cl (50 mL) was added. The aqueous layer was extracted with methyl *tert*-butyl ether (3 x 50 mL) and the combined organic phases were washed with brine (100 mL) and dried over anhydrous Na_2SO_4 before they were filtered and concentrated under reduced pressure. The residue was purified by flash chromatography on silica (hexanes/EtOAc, 100:0 to 95:5) to afford the title compound as a white solid (4.70 g, quant.). Mp: 71.3 – 71.8 °C; $[\alpha]_D^{20}$ = +13.3 (c = 9.8, $CHCl_3$); ^{1}H NMR (400 MHz, $CDCl_3$): δ = 7.29 – 7.22 (m, 2H), 6.91 – 6.82 (m, 2H), 4.44 (q, *J* = 11.6 Hz, 2H), 4.02 (dt, *J* = 4.3, 2.1 Hz, 1H), 3.80 (s, 3H), 3.80 – 3.70 (m, 2H), 3.64 – 3.52 (m, 3H), 3.29 (dd, *J* = 9.0, 2.4 Hz, 1H), 2.10 (dtd, *J* = 13.7, 7.9, 2.5 Hz, 1H), 1.79 (dddd, *J* = 14.1, 12.2, 5.1, 2.0 Hz, 1H), 1.68 – 1.60 (m, 1H), 1.48 (dddd, *J* = 13.7, 10.2, 7.2, 4.9 Hz, 1H), 0.91 (d, *J* = 2.8 Hz, 18H), 0.07 (dd, *J* = 6.7, 5.6 Hz, 12H) ppm; ^{13}C NMR (101 MHz, $CDCl_3$): δ = 159.1, 131.1, 129.3 (2C), 113.8 (2C), 74.7, 72.9, 72.4, 69.4, 67.3, 61.8, 55.4, 34.5, 32.4, 26.3 (3C), 26.0 (3C), 18.3, 18.2, –3.3, –4.1, –4.4, –4.6 ppm; IR (film) $\tilde{\nu}$ = 2952, 2928, 2885, 2856, 1613, 1513, 1462, 1360, 1247, 1088, 831 cm^{-1}; HRMS (ESI): *m/z:* calcd. for $C_{27}H_{50}O_5Si_2Na$ [M+Na]$^+$: 533.30890, found: 533.30920.

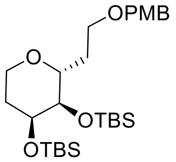

Compound S4. Distilled water (2 mL) was added to a solution of compound **S3** (4.33 g, 8.48 mmol) in

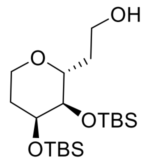

CH_2Cl_2 (40 mL). DDQ (2.31 g, 10.17 mmol) was added and the mixture stirred at room temperature in the dark (aluminum foil) for 1 h. Sat. aq. $NaHCO_3$ (200 mL) was added and, after 30 min of vigorous stirring, the aqueous layer was extracted with CH_2Cl_2 (3 x 50 mL). The combined organic phases were washed with brine (50 mL) and dried over Na_2SO_4 before they were filtered and concentrated under reduced pressure.

The residue was dissolved in MeOH (30 mL) and the solution transferred to a separatory funnel. Then sat. aq. $NaHSO_3$ solution (150 mL) was added, the mixture was shaken for approximately 30 s and then diluted with H_2O (150 mL). The mixture was extracted with EtOAc (150 mL), the combined organic layers were dried over Na_2SO_4 and concentrated under reduced pressure. The residue was purified by flash chromatography (silica; cyclohexane/EtOAc, 20:1 to 5:1) to give the desired product as a colorless oil (3.35 g, quant.). $[\alpha]_D^{20}$ = +61.9° (*c* = 1.84, $CHCl_3$). ^{1}H NMR (400 MHz, $CDCl_3$) δ 4.00 (dt, *J* = 4.3, 2.2 Hz, 1H), 3.88 (ddd, *J* = 10.0, 9.1, 2.7 Hz, 1H), 3.85 – 3.74 (m, 3H), 3.62 (ddd, *J* = 11.0, 5.1, 1.6 Hz, 1H), 3.31 (dd, *J* = 9.1, 2.3 Hz, 1H), 2.83 (br, 1H), 1.98 – 1.88 (m, 1H), 1.80 (dddd, *J* = 14.3, 12.5, 5.2, 2.0 Hz, 1H), 1.68 – 1.50 (m, 2H), 0.90 (s, 9H), 0.88 (s, 9H), 0.06 (s, 3H), 0.06 – 0.04 (m, 6H), 0.04 (s, 3H). ^{13}C NMR (101 MHz, $CDCl_3$) δ 77.1, 74.2, 69.0, 62.4, 61.8, 34.3, 34.1, 26.2, 26.0, 18.3, 18.2, –3.3, –4.1, –4.4, –4.6. IR (film) $\tilde{\nu}$ 3439, 2953, 2928, 2885, 2857, 1472, 1360, 1252, 1146, 1125, 1085, 1050, 955, 831, 772 cm^{-1}. HRMS (ESI) *m/z* calcd. for $C_{19}H_{42}O_4Si_2Na$ $[M+Na]^+$: 413.25138, found: 413.25139.

Compound 6. Dess-Martin periodinane (2.54 g, 6.00 mmol) was added to a solution of alcohol **S4** (1.17

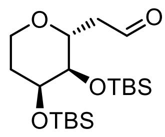

g, 3.00 mmol) and $NaHCO_3$ (1.00 g, 12.00 mmol) in CH_2Cl_2 (10 mL). The mixture was stirred at room temperature for 30 min before sat. aq. $Na_2S_2O_3$ solution (20 mL) and H_2O (20 mL) were introduced to quench the reaction. The organic layer was separated and the aqueous phase extracted with CH_2Cl_2 (3 x 20 mL). The combined organic layers were washed with sat. aq. $NaHCO_3$ solution (3 x 20 mL), dried over Na_2SO_4, and concentrated under reduced pressure. The residue was purified by flash chromatography (silica; hexane/EtOAc, 10:1) to give the title compound as a colorless oil (1.18 g, quant.). $[\alpha]_D^{20}$ = +43.6° (*c* = 1.69, $CHCl_3$). ^{1}H NMR (400 MHz, $CDCl_3$) δ 9.74 (dd, *J* = 3.7, 1.5 Hz, 1H), 4.25 (td, *J* = 9.7, 3.3 Hz, 1H), 4.04 (dt, *J* = 4.3, 2.2 Hz, 1H), 3.90 – 3.75 (m, 1H), 3.61 (ddd, *J* = 11.3, 5.2, 1.6 Hz, 1H), 3.33 (dd, *J* = 9.1, 2.4 Hz, 1H), 2.68 (ddd, *J* = 15.8, 3.3, 1.5 Hz, 1H), 2.31 (ddd, *J* = 16.0, 10.0, 3.7 Hz, 1H), 1.81 (dddd, *J* = 14.3, 12.5, 5.2, 2.0 Hz, 1H), 1.65 (dddd, *J* = 13.8, 3.9, 2.3, 1.4 Hz, 1H), 0.91 (s, 9H), 0.88 (s, 9H), 0.08 (s, 3H), 0.06 (s, 3H), 0.05 (s, 3H), 0.05 (s, 3H). ^{13}C NMR (101 MHz, $CDCl_3$) δ 202.3, 74.1, 71.4, 69.1, 61.9, 46.7, 34.4, 26.2, 26.0, 18.3, 18.1, –3.3, –4.1, –4.5, –4.7. IR (film) $\tilde{\nu}$ 2953, 2929, 2885, 2857, 2724, 1730, 1472, 1463, 1360, 1255, 1125, 1086, 953, 833, 773 cm^{-1}. HRMS (ESI) *m/z* calcd. for $C_{19}H_{40}O_4Si_2Na$ $[M+Na]^+$: 411.23573, found: 411.23629.

Compounds 7 and 8. Vinylmagnesium bromide (1.0 M in THF, 1.70 mL, 1.70 mmol) was added dropwise at 0 °C to a solution of aldehyde **6** (0.33 g, 0.85 mmol) in THF (10 mL). The mixture was stirred at this temperature for 2 h before sat. aq. NH_4Cl solution (30 mL) was carefully added. The mixture was then allowed to warm to room temperature and the mixture was extracted with *tert*-butyl methyl ether (3 x 20 mL). The combined organic layers were dried over Na_2SO_4 and concentrated under reduced pressure, and the residue was purified by flash chromatography (silica; hexane/EtOAc, 20:1 to 10:1) to give major diastereomer as a colorless oil (216.3 mg, 61%) and a second fraction comprised of the minor diastereomer as a colorless oil (88.7 mg, 25%).

Analytical and spectroscopic data of the major isomer **7**: $[\alpha]_D^{20}$ = +61.0° (*c* = 0.80, $CHCl_3$). ^{1}H NMR (400

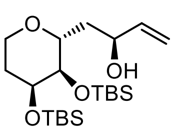

MHz, $CDCl_3$) δ 5.83 (ddd, *J* = 17.2, 10.5, 5.4 Hz, 1H), 5.24 (dd, *J* = 17.2, 1.6 Hz, 1H), 5.04 (dd, *J* = 10.5, 1.6 Hz, 1H), 4.43 – 4.25 (m, 1H), 3.99 (dt, *J* = 4.4, 2.2 Hz, 1H), 3.96 – 3.77 (m, 3H), 3.65 (ddd, *J* = 11.0, 5.2, 1.6 Hz, 1H), 3.27 (dd, *J* = 9.1, 2.3 Hz, 1H), 1.97 (ddd, *J* = 14.3, 2.4, 2.4 Hz, 1H), 1.86 – 1.74 (m, 1H), 1.68 – 1.58 (m, 1H), 1.39 (ddd, *J* = 14.3, 11.0, 9.7 Hz, 1H), 0.90 (s, 9H), 0.88 (s, 9H), 0.07 (s, 3H), 0.05 (s, 6H), 0.04 (s, 3H). ^{13}C NMR (101 MHz, $CDCl_3$) δ 140.9, 113.9, 77.1, 74.3, 73.3, 69.0, 61.8, 39.0, 34.2, 26.2, 26.0, 18.3, 18.2, –3.3, –4.1, –4.4, –4.6. IR (film) $\tilde{\nu}$ 3507, 2954, 2929, 2885, 2858, 1472, 1361, 1255, 1121, 1086, 960, 835, 773 cm^{-1}. HRMS (ESI) *m/z* calcd. for $C_{21}H_{44}O_4Si_2Na$ $[M+Na]^+$: 439.26703, found: 439.26721.

Analytical and spectroscopic data of the minor isomer **8**: $[\alpha]_D^{20}$ = +39.0° (*c* = 1.49, $CHCl_3$). ^{1}H NMR (400

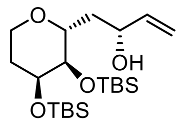

MHz, $CDCl_3$) δ 5.89 (ddd, *J* = 17.2, 10.5, 4.8 Hz, 1H), 5.29 (dt, *J* = 17.2, 1.8 Hz, 1H), 5.12 (dt, *J* = 10.5, 1.6 Hz, 1H), 4.39 (br, 1H), 4.08 – 3.98 (m, 2H), 3.79 (ddd, *J* = 12.4, 11.0, 2.2 Hz, 1H), 3.63 (ddd, *J* = 11.2, 5.1, 1.6 Hz, 1H), 3.36 (dd, *J* = 9.1, 2.4 Hz, 1H), 3.28 (d, *J* = 6.1 Hz, 1H), 1.88 (ddd, *J* = 14.4, 6.8, 2.9 Hz, 1H), 1.80 (dddd, *J* = 14.3, 12.4, 5.2, 2.0 Hz, 1H), 1.73 – 1.60 (m, 2H), 0.90 (s, 9H), 0.89 (s, 9H), 0.09 – 0.05 (m, 9H), 0.04 (s, 3H). ^{13}C NMR (101 MHz, $CDCl_3$) δ 140.9, 114.0, 73.8, 73.6, 70.4, 69.1, 61.7, 37.3, 34.3, 26.2, 26.0, 18.3, 18.2, –3.2, –4.0, –4.4, –4.6. IR (film) $\tilde{\nu}$ 3473, 2954, 2929, 2886, 2857, 1472, 1361, 1254, 1120, 1086, 965, 836, 773 cm^{-1}. HRMS (ESI) *m/z* calcd. for $C_{21}H_{44}O_4Si_2Na$ $[M+Na]^+$: 439.26703, found: 439.26706.

Mosher ester analysis of the major diastereomer 7: Starting from compound **7** and (*R*)-(–)-MTPA-Cl,

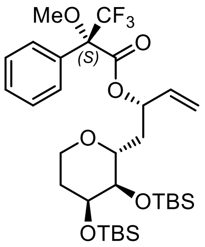

the esterification under standard conditions (CH_2Cl_2, pyridine, 0°C to RT) gave the corresponding (*S*)-(+)-MTPA ester as a colorless oil (6.36 mg, quant.). $[\alpha]_D^{20}$ = +12.3° (*c* = 0.64, $CHCl_3$). ^{1}H NMR (400 MHz, $CDCl_3$) δ 7.60 – 7.48 (m, 2H), 7.42 – 7.33 (m, 3H), 5.83 (ddd, *J* = 17.1, 10.3, 7.6 Hz, 1H), 5.67 (ddd, *J* = 9.9, 7.6, 4.7 Hz, 1H), 5.39 (dd, *J* = 17.2, 1.2 Hz, 1H), 5.30 (ddd, *J* = 10.3, 1.4, 0.7 Hz, 1H), 3.99 (dt, *J* = 4.3, 2.2 Hz, 1H), 3.76 – 3.63 (m, 2H), 3.58 (ddd, *J* = 11.2, 5.3, 1.5 Hz, 1H), 3.53 (q, *J* = 1.2 Hz, 3H), 3.27 (dd, *J* = 9.0, 2.3 Hz, 1H), 1.98 (ddd, *J* = 13.4, 9.9, 2.2 Hz, 1H), 1.84 – 1.73 (m, 1H), 1.71 – 1.58 (m, 2H), 0.88 (s,

9H), 0.86 (s, 9H), 0.05 (s, 3H), 0.03 (s, 6H), 0.00 (s, 3H). ^{13}C NMR (101 MHz, $CDCl_3$) δ 165.6, 134.8, 132.6, 129.6, 128.4, 127.6, 123.5 (q, J = 288.6 Hz), 119.5, 76.2, 74.4, 72.1, 69.2, 61.7, 55.5, 36.3, 34.4, 26.2, 26.0, 18.3, 18.2, −3.3, −4.1, −4.5, −4.7. IR (film) $\tilde{\nu}$ 2954, 2929, 2888, 2857, 1749, 1472, 1361, 1255, 1169, 1125, 1084, 961, 835, 774 cm^{-1}. HRMS (ESI) m/z calcd. for $C_{31}H_{51}F_3O_6Si_2Na$ $[M+Na]^+$: 655.30685, found: 655.30687.

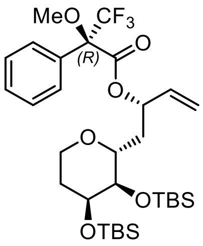

The analogous reaction of compound **7** with (*S*)-(+)-MTPA-Cl gave the corresponding (*R*)-(+)-MTPA ester as a colorless oil (5.45 mg, 90%). $[\alpha]_D^{20}$ = +62.0° (c = 0.55, $CHCl_3$). ^{1}H NMR (400 MHz, $CDCl_3$) δ 7.62 – 7.47 (m, 2H), 7.44 – 7.31 (m, 3H), 5.84 – 5.60 (m, 2H), 5.38 – 5.26 (m, 1H), 5.26 – 5.20 (m, 1H), 4.01 (dt, J = 4.3, 2.2 Hz, 1H), 3.79 – 3.65 (m, 2H), 3.60 (ddd, J = 11.2, 5.3, 1.5 Hz, 1H), 3.56 (q, J = 1.3 Hz, 3H), 3.29 (dd, J = 9.1, 2.3 Hz, 1H), 2.07 (ddd, J = 13.4, 9.3, 2.3 Hz, 1H), 1.84 – 1.67 (m, 2H), 1.62 (ddt, J = 13.8, 3.9, 2.3 Hz, 1H), 0.89 (s, 9H), 0.89 (s, 9H), 0.06 (s, 3H), 0.06 (s, 3H), 0.04 (s, 6H). ^{13}C NMR (101 MHz, $CDCl_3$) δ 165.5, 134.8, 132.7, 129.6, 128.4, 127.6, 123.5 (q, J = 288.1 Hz), 119.0, 76.1, 74.4, 72.4, 69.3, 61.7, 55.6, 36.7, 34.5, 26.2, 26.0, 18.3, 18.2, −3.3, −4.1, −4.5, −4.6. IR (film) $\tilde{\nu}$ 2954, 2929, 2895, 2857, 1749, 1472, 1361, 1256, 1169, 1126, 1084, 962, 835, 774 cm^{-1}. HRMS (ESI) m/z calcd. for $C_{31}H_{51}F_3O_6Si_2Na$ $[M+Na]^+$: 655.30685, found: 655.30694.

Table S1. Determination of absolute configuration of the chiral center at C66 of compound **7** via Mosher ester analysis.[2]

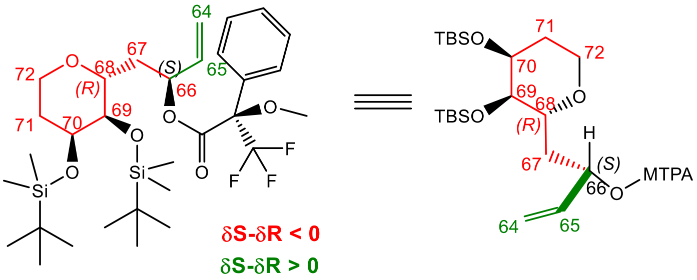

No.	$\delta_{H\ NMR}$ (*S*-ester) (ppm)	$\delta_{H\ NMR}$ (*R*-ester) (ppm)	$\Delta\delta$ (δ_S -δ_R, ppm)
64-*trans*	5.39	5.3	0.09
64-*cis*	5.3	5.23	0.07
65	5.83	5.74	0.09
66	5.67	5.66	0.01
67'	1.66	1.72	−0.06
67''	1.98	2.07	−0.09
68	3.7	3.74	−0.04
69	3.27	3.29	−0.02
70	3.99	4.01	−0.02
71'	1.60	1.62	−0.02
71''	1.78	1.80	−0.02
72'	3.68	3.71	−0.03
72''	3.58	3.60	−0.02

Compound 8a. Dess-Martin periodinane (180.5 mg, 0.43 mmol) was added to a solution of alcohol **8** (88.7 mg, 0.21 mmol) and $NaHCO_3$ (71.5 mg, 0.85 mmol) in CH_2Cl_2 (5 mL). The mixture was stirred at room temperature for 2 h before sat. aq. $Na_2S_2O_3$ solution (10 mL) and H_2O (10 mL) were introduced. The organic layer was separated and the aqueous phase extracted with CH_2Cl_2 (3 x 10 mL). The combined organic layers were washed with sat. aq. $NaHCO_3$ solution (3 x 20 mL), dried over Na_2SO_4, and concentrated under reduced pressure. The residue was purified by flash chromatography (silica; hexane/EtOAc, 10:1) to give the title compound as a colorless oil (89.2 mg, quant.). $[\alpha]_D^{20}$ = + 44.8° (*c* = 1.42, $CHCl_3$). ^1H NMR (400 MHz, $CDCl_3$) δ 6.37 (dd, *J* = 17.7, 10.6 Hz, 1H), 6.18 (dd, *J* = 17.7, 1.2 Hz, 1H), 5.80 (dd, *J* = 10.6, 1.1 Hz, 1H), 4.22 (ddd, *J* = 10.4, 9.1, 2.5 Hz, 1H), 4.03 (dt, *J* = 4.3, 2.2 Hz, 1H), 3.77 (ddd, *J* = 12.4, 11.2, 2.3 Hz, 1H), 3.57 (ddd, *J* = 11.3, 5.2, 1.6 Hz, 1H), 3.34 (dd, *J* = 9.2, 2.3 Hz, 1H), 2.90 (dd, *J* = 14.8, 2.5 Hz, 1H), 2.52 (dd, *J* = 14.9, 10.3 Hz, 1H), 1.79 (dddd, *J* = 14.3, 12.4, 5.2, 2.0 Hz, 1H), 1.62 (dddd, *J* = 13.8, 3.9, 2.3, 1.5 Hz, 1H), 0.90 (s, 9H), 0.89 (s, 9H), 0.07 (s, 9H), 0.04 (s, 3H). ^{13}C NMR (101 MHz, $CDCl_3$) δ 199.5, 136.8, 128.2, 74.2, 72.7, 69.2, 61.9, 43.1, 34.4, 26.1, 26.0, 18.3, 18.1, –3.3, –4.1, –4.5, –4.7. IR (film) $\tilde{\nu}$ 2954, 2929, 2886, 2857, 1688, 1617, 1472, 1360, 1255, 1120, 1085, 962, 835, 773 cm^{-1}. HRMS (ESI) *m/z* calcd. for $C_{21}H_{42}O_4Si_2Na$ $[M+Na]^+$: 437.25138, found: 437.25146.

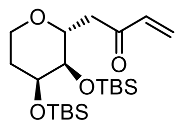

Compound 7 (by CBS reduction). (*R*)-(+)-2-Methyl-CBS-oxazaborolidine (31.8 mg, 0.11 mmol)[3] was added at –20 °C (bath temperature) to a solution of ketone **8a** (238.0 mg, 0.57 mmol) in THF (2.0 mL). The mixture was stirred at this temperature for 20 min before $BH_3 \cdot SMe_2$ (0.08 mL, 0.86 mmol) was added dropwise. The mixture was allowed to warm to 0 °C over 4 h before the reaction was quenched with MeOH (1.0 mL) and sat. aq. NH_4Cl solution (5 mL). The aqueous mixture was extracted with EtOAc (3 x 10 mL). The combined organic layers were washed with brine (20 mL), dried over Na_2SO_4, and concentrated under reduced pressure. The residue was purified by flash chromatography (silica; hexane/EtOAc, 20:1 to 10:1) to give allylic alcohol **7** as a colorless oil (125.2 mg, 52%, *dr* = 18:1 (^1H NMR)). See above for the analytical and spectral data.

Compound 9. A flame-dried flask was charged with CuI (1.29 g, 6.78 mmol) and THF (30 mL). The suspension was cooled to –78 °C (dry ice/acetone bath) before vinylmagnesium bromide (1.0 M in THF, 40.7 mL, 40.7 mmol) was added dropwise over 10 min. The resulting slurry was stirred for another 15 min before a solution of epoxide **2** (5.65 g, 27.13 mmol) in THF (10 mL)was slowly added. The resulting mixture was allowed to reach –50 °C over the course of 1 h at which temperature stirring was continued until TLC control indicated complete conversion of the substrate. The mixture was then cannulated into an ice-cold solution of sat. aq. NH_4Cl (100 mL) and the resulting mixture was vigorously stirred at room temperature until a clear blue aqueous phase and a colorless organic phase had formed. The aqueous layer was extracted with methyl *tert*-butyl ether

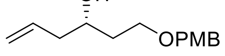

(3 x 50 mL), the combined organic phases were washed with brine (100 mL) and dried over Na_2SO_4 before being filtered and concentrated under reduced pressure. The resulting product was pure enough for direct use in the next step (6.08 g, 95%). $[\alpha]_D^{20}$ = −2.2 (c = 0.84, $CHCl_3$); ^1H NMR (400 MHz, $CDCl_3$): δ = 7.29 – 7.20 (m, 2H), 6.92 – 6.83 (m, 2H), 5.83 (ddt, *J* = 17.4, 10.4, 7.1 Hz, 1H), 5.15 – 5.04 (m, 2H), 4.45 (s, 2H), 3.90 – 3.82 (m, 1H), 3.80 (s, 3H), 3.73 – 3.66 (m, 1H), 3.65 – 3.58 (m, 1H), 2.91 – 2.86 (m, 1H), 2.24 (ddt, *J* = 7.3, 6.2, 1.3 Hz, 2H), 1.79 – 1.70 (m, 2H) ppm; ^{13}C NMR (101 MHz, $CDCl_3$): δ = 159.4, 135.0, 130.2, 129.5 (2C), 117.7, 114.0 (2C), 73.1, 70.6, 68.8, 55.4, 42.1, 36.0 ppm; IR (film) $\tilde{\nu}$ = 3442, 2937, 2861, 1613, 1513, 1441, 1247, 1089 cm^{-1}; HRMS (EI): *m/z:* calcd. for $C_{14}H_{20}O_3$ $[M^+]$: 236.140695, found: 236.140630.

Compound 10. An oven-dried Schlenk tube was charged with the homoallylic alcohol **9** (3.96 g, 16.76 mmol). The tube was evacuated and refilled with Ar before methyl acrylate (4.56 mL, 50.27 mmol) and degassed CH_2Cl_2 (40 mL) were added. The mixture was stirred at room temperature and Grubbs II catalyst (142.0 mg, 0.17 mmol) was introduced. Stirring was continued at 40 °C (bath temperature) for 2 h under a gentle flow of Ar before a second portion of Grubbs II catalyst (142.0 mg, 0.17 mmol) in degassed CH_2Cl_2 (5 mL) was added. The resulting mixture was stirred at 40 °C for another 1 h before it was allowed to reach ambient temperature. Celite was added and the suspension stirred for 10 min. The mixture was filtered through a plug of silica, rinsing with *tert*-butyl methyl ether. The combined filtrates were concentrated under reduced pressure and the residue was purified by flash chromatography (silica; hexanes/EtOAc, 3:1 to 1:1) to give the title compound as a greenish oil (4.57 g, 93% over two steps, *E/Z* > 20:1). $[\alpha]_D^{20}$ = –8.4 (c = 1.3, $CHCl_3$); ^1H NMR (400 MHz, $CDCl_3$): δ = 7.25 – 7.20 (m, 2H), 6.98 (dt, *J* = 15.7, 7.4 Hz, 1H), 6.92 – 6.83 (m, 2H), 5.89 (dt, *J* = 15.7, 1.5 Hz, 1H), 4.44 (s, 2H), 4.01 – 3.91 (m, 1H), 3.80 (s, 3H), 3.72 (s, 3H), 3.71 – 3.66 (m, 1H), 3.65 – 3.58 (m, 1H), 3.15 (d, *J* = 2.9 Hz, 1H), 2.46 – 2.29 (m, 2H), 1.84 – 1.68 (m, 2H) ppm; ^{13}C NMR (101 MHz, $CDCl_3$): δ = 166.9, 159.5, 145.7, 129.9, 129.5 (2C), 123.3, 114.0 (2C), 73.2, 70.5, 68.8, 55.4, 51.6, 40.2, 36.0 ppm; IR (film) $\tilde{\nu}$ = 3471, 2948, 2862, 1720, 1657, 1612, 1513, 1437, 1247, 1089, 1034 cm^{-1}; HRMS (ESI): *m/z:* calcd. for $C_{16}H_{22}O_5Na$ $[M+Na]^+$: 317.13594, found: 317.13576.

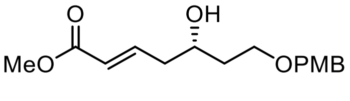

Compound 11. Triethylamine (4.33 mL, 31.05 mmol) and methanesulfonyl chloride (1.80 mL, 23.29 mmol) were added at 0 °C to a solution of compound **10** (4.57 g, 15.53 mmol) in CH_2Cl_2 (20 mL). Stirring was continued at 0 °C for 30 min before the reaction was quenched with water (50 mL). The organic layer was separated and the aqueous phase extracted with CH_2Cl_2 (3 x 20 mL). The combined organic layers were dried over $MgSO_4$ and concentrated under reduced pressure and the residue was purified by flash chromatography (silica; hexanes/EtOAc, 5:1 to 2:1) to give an inseparable mixture of the desired mesylate ester and thiirane by-product (5.59 g, ~94% calculated yield based on NMR) which was used in the next step without further purification.

A flask was charged with $K_3Fe(CN)_6$ (14.35 g, 43.58 mmol), K_2CO_3 (6.02 g, 43.58 mmol), $MeSO_2NH_2$ (2.76 g, 29.05 mmol), $(DHQD)_2PHAL$ (452.6 mg, 0.58 mmol), $K_2OsO_2(OH)_4$ (107.0 mg, 0.29 mmol), *t*BuOH (30 mL) and H_2O (30 mL). The solution was vigorously stirred for 15 min until a homogeneous phase had formed. The mixture was cooled to 0 °C and a solution of the crude mesylate (5.41 g, 14.53 mmol) in CH_2Cl_2 (6 mL) was added. Stirring was continued at 0 °C for 8 h and for another 12 h at room temperature. sat. aq. Na_2SO_3 solution was introduced and the mixture vigorously stirred for 1 h. The organic layer was separated and the aqueous phase extracted with CH_2Cl_2 (3 x 30 mL). The combined organic layers were washed with HCl (2 M, 30 mL) and brine (100 mL), dried over Na_2SO_4, and concentrated under reduced pressure to give a pale-yellow oil that was used in the next step without further purification.

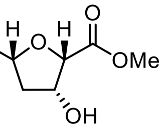

11a

2,6-Lutidine (30 mL) was added to this crude material and the resulting mixture was stirred at 120 °C (bath temperature) for 6 h. The mixture was concentrated under reduced pressure and the residue purified by flash chromatography (silica; CH_2Cl_2/EtOAc, 10:1 to 2:1) to give a mixture of diastereomeric ether derivatives as a pale-yellow oil (4.18 g), which was used in the next step without further purification. For analytical purposes, an aliquot was subjected to a second flash chromatographic purification (silica; CH_2Cl_2/EtOAc, 10:1 to 2:1). The major isomer analyzed as follows: $[\alpha]_D^{20}$ = −21.8 (c = 1.1, $CHCl_3$); ^1H NMR (400 MHz, $CDCl_3$): δ = 7.28 – 7.21 (m, 2H), 6.91 – 6.82 (m, 2H), 4.58 (dp, *J* = 6.2, 3.7 Hz, 1H), 4.43 (s, 2H), 4.34 (d, *J* = 4.7 Hz, 1H), 4.15 (dtd, *J* = 7.9, 6.7, 5.5 Hz, 1H), 3.79 (d, *J* = 1.6 Hz, 6H), 3.69 (ddd, *J* = 9.7, 6.5, 5.3 Hz, 1H), 3.60 (ddd, *J* = 9.7, 7.3, 5.2 Hz, 1H), 2.77 (d, *J* = 6.3 Hz, 1H), 2.39 (ddd, *J* = 13.5, 7.9, 6.6 Hz, 1H), 2.09 (dtd, *J* = 14.3, 6.6, 5.1 Hz, 1H), 1.96 (ddt, *J* = 14.3, 7.2, 5.5 Hz, 1H), 1.79 (ddd, *J* = 13.6, 6.9, 3.3 Hz, 1H) ppm; ^{13}C NMR (101 MHz, $CDCl_3$): δ = 170.3, 159.3, 130.4, 129.5, 113.9, 82.3, 76.6, 73.5, 72.8, 66.9, 55.4, 52.3, 39.9, 35.4 ppm; IR (film) $\tilde{\nu}$ = 3468, 2950, 2861, 1751, 1612, 1513, 1247, 1089 cm^{-1}; HRMS (EI): *m/z:* calcd. for $C_{16}H_{22}O_6$ $[M^+]$: 310.14109, found: 310.14115.

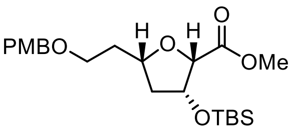

Imidazole (1.98 g, 29.05 mmol), DMAP (0.27 g, 2.18 mmol) and TBSCl (4.38 g, 29.05 mmol) were added at 0 °C to a solution of the crude product in DMF (15 mL). The mixture was stirred at room temperature for 16 h before the reaction was quenched with water (150 mL). The mixture was extracted with EtOAc (3 x 50 mL), the combined organic layers were washed with brine (100 mL), dried over Na_2SO_4, and concentrated under reduced pressure, and the residue was purified by flash chromatography (silica; hexanes/EtOAc, 20:1 to 5:1) to give the title compound as a pale-yellow oil (5.05 g, 77% over four steps, dr > 20:1). $[\alpha]_D^{20}$ = −14.5 (c = 1.0, $CHCl_3$); ^1H NMR (600 MHz, $CDCl_3$): δ = 7.28 – 7.23 (m, 2H), 6.88 – 6.85 (m, 2H), 4.60 (ddd, *J* = 6.4, 5.8, 4.9 Hz, 1H), 4.45 (d, *J* = 11.5 Hz, 1H), 4.41 (d, *J* = 11.5 Hz, 1H), 4.39 (d, *J* = 5.8 Hz, 1H), 4.18 (qd, *J* = 7.3, 5.2 Hz, 1H), 3.80 (s, 3H), 3.72 (s, 3H), 3.67 – 3.58 (m, 1H), 3.59 (dd, *J* = 9.5, 5.8 Hz, 1H), 2.24 (dt, *J* = 13.0, 6.4 Hz, 1H), 2.18 (ddt, *J* = 13.5, 7.9, 5.5 Hz, 1H), 1.92 (dddd, *J* = 13.9, 7.8, 6.0, 5.2 Hz, 1H),

1.75 (ddd, *J* = 12.4, 7.3, 4.9 Hz, 1H), 0.85 (s, 9H), 0.05 (s, 3H), 0.04 (s, 3H) ppm; ^{13}C NMR (151 MHz, $CDCl_3$): δ = 170.6, 159.3, 130.8, 129.4 (2C), 113.9 (2C), 82.0, 76.9, 74.0, 72.8, 67.3, 55.4, 51.8, 40.5, 36.1, 25.7 (3C), 18.0, −4.7, −5.1 ppm; IR (film) $\tilde{\nu}$ = 2951, 2931, 2888, 2857, 1766, 1613, 1513, 1248, 1095 cm^{-1}; HRMS (ESI): *m/z:* calcd. for $C_{22}H_{36}O_6SiNa$ $[M+Na]^+$: 447.21734, found: 447.21695.

Compound 13. (*S*)-3-Hydroxy-γ-butyrolactone (**12**) (3.06 g, 30 mmol) was dissolved in CH_2Cl_2 (100 mL).

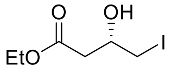

Ethanol (5.3 mL, 90 mmol) was added and the solution was cooled to 0 °C (ice bath) before TMSI (6.4 mL, 45 mmol) was introduced. The mixture was then stirred at room temperature overnight. The solution was poured into a aqueous solution of $Na_2S_2O_3$ (5% *w/w*, 50 mL), the organic phase was separated and the aqueous layer extracted with Et_2O (3 x 20 mL). The combined organic phases were washed with aq. $Na_2S_2O_3$ (5% *w/w*, 50 mL) and brine (50 mL) before they were dried over Na_2SO_4, filtered, and concentrated under reduced pressure (***Note***: *the product is volatile*). The residue was purified by flash chromatography on silica (pentane/methyl *tert*-butyl ether, 9/1 to 6/4) to afford the title compound as a pale yellow oil (6.89 g, 89%). $[\alpha]_D^{20}$ = −11.9 (c = 1.86, $CHCl_3$); ^1H NMR (400 MHz, $CDCl_3$): δ = 4.18 (q, *J* = 7.2 Hz, 2H), 3.99 (dtd, *J* = 9.9, 5.4, 2.6 Hz, 1H), 3.34 (dd, *J* = 10.3, 5.2 Hz, 1H), 3.29 (dd, *J* = 10.3, 5.7 Hz, 1H), 3.16 (d, *J* = 4.7 Hz, 1H), 2.67 (dd, *J* = 16.5, 4.2 Hz, 1H), 2.60 (dd, *J* = 16.5, 8.0 Hz, 1H), 1.28 (t, *J* = 7.1 Hz, 3H) ppm; ^{13}C NMR (101 MHz, $CDCl_3$): δ = 171.9, 67.6, 61.2, 40.8, 14.3, 12.1 ppm; IR (film) $\tilde{\nu}$ = 3438, 2981, 1722, 1445, 1399, 1373, 1293, 1182, 1031 cm^{-1}; HRMS (ESI): *m/z:* calcd. for $C_6H_{11}O_3INa$ $[M+Na]^+$: 280.96451, found: 280.96437. The data are consistent with the literature.[4]

Compound 14. Ag_2O (5.56 g, 24.0 mmol) was added in one portion to a solution of compound **13** (5.16

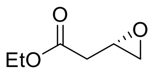

g, 20.0 mmol) in MeCN (80 mL), and the resulting mixture was vigorously stirred at 70 °C (bath temperature) for 24 h. The solution was allowed to reach room temperature and filtered through a plug of Celite, which was carefully rinsed with Et_2O. The combined filtrates were concentrated under reduced pressure (> 500 mbar; ***Note***: *the product is volatile*) to reduce the volume of the solvents. The sufficiently concentrated mixture was purified by flash chromatography on silica (pentane/Et_2O, 4:1) to afford the title compound as a pale yellow oil (2.40 g, 92%). $[\alpha]_D^{20}$ = −8.7 (c = 1.2, $CHCl_3$); ^1H NMR (400 MHz, $CDCl_3$): δ = 4.18 (q, *J* = 7.1 Hz, 2H), 3.28 (tdd, *J* = 6.1, 4.0, 2.6 Hz, 1H), 2.84 (dd, *J* = 4.9, 4.0 Hz, 1H), 2.64 – 2.47 (m, 3H), 1.28 (t, *J* = 7.1 Hz, 3H) ppm; ^{13}C NMR (101 MHz, $CDCl_3$): δ = 170.5, 61.0, 48.2, 46.9, 38.2, 14.3 ppm; IR (film) $\tilde{\nu}$ = 2985, 2933, 1731, 1409, 1324, 1263, 1181, 1028 cm^{-1}; HRMS (ESI): *m/z:* calcd. for $C_6H_{10}O_3Na$ $[M+Na]^+$: 153.05221, found: 153.05219. The data are consistent with the literature.[4]

Compound 15. *n*-BuLi (1.6 M solution in hexanes, 19.2 mL, 30.7 mmol,) was added dropwise to a solution of TMS-acetylene (4.40 mL, 30.7 mmol) in toluene (50 mL) at –35 °C. After 15 min, the dry ice bath was replaced by a ice/water bath and the reaction allowed to reach 0 °C. Et_2AlCl (1 M in hexanes, 30.7 mL, 30.7 mnol) was added dropwise and the mixture was vigorously stirred for 1 h at 0 °C. A solution of compound **14** (2.00 g, 15.40 mmol) in toluene (10 mL) was added and stirring continued for 2 h at 0 °C. For work up, sat. aq. NH_4Cl (5 mL) was added, followed by HCl (2 M, 50 mL). The solution was diluted with methyl *tert*-butyl ether (20 mL) and stirring continued for 30 min at room temperature before the aqueous phase was extracted with methyl *tert*-butyl ether (3 x 30 mL). The combined organic phases were washed with brine (50 mL) and dried over $MgSO_4$ before they were filtered and carefully concentrated under reduced pressure (***Note***: *the product is volatile*). The residue was purified by flash chromatography on silica (pentane/methyl *tert*-butyl ether, 4:1 to 7:3) to afford the title compound as a clear yellow oil (3.18 g, 91%). $[\alpha]_D^{20}$ = –29.4 (c = 1.4, $CHCl_3$); ^{1}H NMR (400 MHz, $CDCl_3$): δ = 4.24 – 4.11 (m, 3H), 3.02 (s, 1H), 2.67 (ddd, *J* = 16.6, 3.6, 0.8 Hz, 1H), 2.58 – 2.41 (m, 3H), 1.29 (td, *J* = 7.1, 1.0 Hz, 3H), 0.21 – 0.11 (m, 9H) ppm; ^{13}C NMR (101 MHz, $CDCl_3$): δ = 172.6, 102.3, 88.0, 66.7, 61.0, 40.3, 27.9, 14.3, 0.2 (3C) ppm; IR (film) $\tilde{\nu}$ = 3465, 2960, 2904, 2176, 1731, 1249, 1158, 839 cm^{-1}; HRMS (ESI): *m/z:* calcd. for $C_{11}H_{20}O_3SiNa$ $[M+Na]^+$: 251.10739, found: 251.10732. The data are consistent with those reported in the literature.[4]

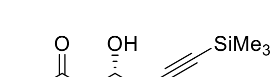

Compound S5. $NaBH_4$ (1.08 g, 28.5 mmol) was added in portions at 0 °C (bath temperature) to a suspension of $Ni(OAc)_2{\cdot}4H_2O$ (3.40 g, 13.7 mmol) in EtOH (20 mL). The resulting mixture was stirred at 0 °C for 20 min before degassed ethylenediamine (2.3 mL, 34.2 mmol) was added and stirring continued at room temperature for 20 min. The flask was purged with hydrogen gas for 3 min to replace the argon before a solution of alkyne **15** (2.28 g, 10.0 mmol) in EtOH (10 mL) was added. The mixture was stirred under hydrogen atmosphere (H_2 balloon) at room temperature for 20 h. The mixture was filtered through a plug of silica, rinsing with *tert*-butyl methyl ether. The combined filtrates were concentrated under reduced pressure and the residue was purified by flash chromatography (silica; pentane/*tert*-butyl methyl ether, 10:1 to 5:1) to the title compound as a colorless oil (2.26 g, 86%). $[\alpha]_D^{20}$ = –19.1 (c = 1.3, $CHCl_3$); ^{1}H NMR (400 MHz, $CDCl_3$): δ = 6.31 (dt, *J* = 14.4, 7.3 Hz, 1H), 5.68 (dt, *J* = 14.1, 1.4 Hz, 1H), 4.17 (q, *J* = 7.1 Hz, 2H), 4.09 (dtt, *J* = 9.9, 6.8, 3.1 Hz, 1H), 2.95 (d, *J* = 3.7 Hz, 1H), 2.53 (dd, *J* = 16.4, 3.3 Hz, 1H), 2.46 – 2.27 (m, 3H), 1.28 (t, *J* = 7.1 Hz, 3H), 0.13 (s, 9H) ppm; ^{13}C NMR (101 MHz, $CDCl_3$): δ = 173.0, 143.4, 133.0, 67.9, 60.9, 40.8, 40.2, 14.3, 0.3 (3C) ppm; IR (film) $\tilde{\nu}$ = 3457, 2955, 2901, 1733, 1607, 1373, 1248, 1152, 836 cm^{-1}; HRMS (ESI): *m/z:* calcd. for $C_{11}H_{22}O_3SiNa$ $[M+Na]^+$: 253.12304, found: 253.12302.

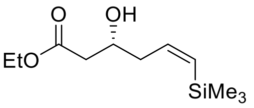

Compound 16. An oven-dried flask was charged with alcohol **S5** (3.46 g, 15.0 mmol) and CH_2Cl_2 (20.0 mL). The solution was stirred at 0 °C (bath temperature) when triethylamine (4.2 mL, 30.0 mmol) was added dropwise, followed by slow addition of TMSCl (3.0 mL, 22.5 mmol). Stirring was continued at room temperature until the alcohol was fully consumed (*ca.* 24 h). The reaction was quenched with sat. aq. $NaHCO_3$ (20 mL) and the aqueous phase extracted with CH_2Cl_2 (3 x 20 mL). The combined organic layers were dried over $MgSO_4$ and carefully concentrated under reduced pressure, and the residue was purified by flash chromatography (silica; pentane/*tert*-butyl methyl ether, 20:1) to give the title compound as a colorless liquid (4.35 g, 96%). $[\alpha]_D^{20}$ = –21.3° (*c* = 1.38, $CHCl_3$). ^1H NMR (400 MHz, $CDCl_3$) δ 6.28 (dt, *J* = 14.4, 7.4 Hz, 1H), 5.61 (dt, *J* = 14.2, 1.4 Hz, 1H), 4.20 (dq, *J* = 7.2, 6.0 Hz, 1H), 4.13 (qt, *J* = 7.1, 3.7 Hz, 2H), 2.44 – 2.41 (m, 2H), 2.33 (ddt, *J* = 7.6, 6.1, 1.6 Hz, 2H), 1.26 (t, *J* = 7.2 Hz, 3H), 0.11 (s, 9H), 0.10 (s, 9H). ^{13}C NMR (101 MHz, $CDCl_3$) δ 171.8, 144.0, 132.1, 69.5, 60.5, 42.9, 41.5, 14.4, 0.4, 0.3. IR (film) $\tilde{\nu}$ 2957, 2902, 1738, 1608, 1375, 1250, 1151, 1096, 839, 763 cm^{-1}. HRMS (ESI) *m/z* calcd. for $C_{14}H_{30}O_3Si_2Na$ $[M+Na]^+$: 325.16257, found: 325.16268.

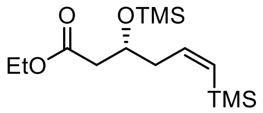

Compound 17. Isopropylmagnesium chloride (2.0 M in THF, 21.6 mL, 43.2 mmol) was added dropwise at –20 °C (bath temperature) to a solution of compound **16** (4.35 g, 14.4 mmol) and N,O-dimethylhydroxylamine hydrochloride (2.10 g, 21.6 mmol) in THF (30 mL). The mixture was stirred at –20 °C for 2 h before the reaction was quenched with sat. aq. NH_4Cl solution (30 mL). The mixture was allowed to reach room temperature before it was extracted with *tert*-butyl methyl ether (3 x 30 mL). The combined organic layers were dried over Na_2SO_4 and carefully concentrated under reduced pressure. The residue was purified by flash chromatography (silica; pentane/*tert*-butyl methyl ether, 10:1) to give the title compound as a pale-yellow liquid (4.00 g, 88%). $[\alpha]_D^{20}$ = –34.8° (*c* = 1.76, $CHCl_3$). ^1H NMR (400 MHz, $CDCl_3$) δ 6.32 (dt, *J* = 14.4, 7.3 Hz, 1H), 5.60 (dt, *J* = 14.2, 1.5 Hz, 1H), 4.29 (dtd, *J* = 8.0, 6.1, 4.7 Hz, 1H), 3.68 (s, 3H), 3.17 (s, 3H), 2.73 (dd, *J* = 14.3, 7.7 Hz, 1H), 2.41 (dd, *J* = 14.9, 4.9 Hz, 1H), 2.37 – 2.32 (m, 2H), 0.11 (s, 9H), 0.09 (s, 9H). ^{13}C NMR (101 MHz, $CDCl_3$) δ 172.5, 144.6, 131.7, 69.4, 61.4, 41.7, 39.8, 32.1, 0.4, 0.3. IR (film) $\tilde{\nu}$ 2955, 2900, 1664, 1607, 1414, 1385, 1248, 1088, 982, 836, 762, 689 cm^{-1}. HRMS (ESI) *m/z* calcd. for $C_{14}H_{31}NO_3Si_2Na$ $[M+Na]^+$: 340.17347, found: 340.17364.

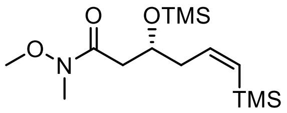

Compound 18. Methylmagnesium bromide (3.0 M in Et_2O, 6.3 mL, 18.9 mmol) was added dropwise at 0 °C (bath temperature) to a solution of Weinreb amide **17** (4.00 g, 12.6 mmol) in Et_2O (100 mL). The mixture was stirred at this temperature for 1 h before the reaction was quenched with sat. aq. NH_4Cl (30 mL). The mixture was allowed to reach room temperature before it was extracted with *tert*-butyl methyl ether (3 x 30 mL). The combined organic layers were dried over Na_2SO_4 and carefully concentrated under reduced pressure.

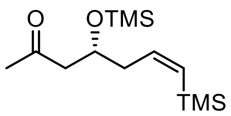

The residue was purified by flash chromatography (silica; pentane/*tert*-butyl methyl ether, 10:1) to give the title compound as a yellow liquid (2.91 g, 85%). $[\alpha]_D^{20}$ = –35.2° (*c* = 1.65, $CHCl_3$). ^{1}H NMR (400 MHz, $CDCl_3$) δ 6.26 (dt, *J* = 14.4, 7.3 Hz, 1H), 5.60 (dt, *J* = 14.2, 1.5 Hz, 1H), 4.22 (dtd, *J* = 7.9, 6.1, 4.5 Hz, 1H), 2.61 (dd, *J* = 15.4, 7.8 Hz, 1H), 2.46 (dd, *J* = 15.5, 4.4 Hz, 1H), 2.30 (ddt, *J* = 7.4, 6.2, 1.2 Hz, 2H), 2.14 (s, 3H), 0.11 (s, 9H), 0.09 (s, 9H). ^{13}C NMR (101 MHz, $CDCl_3$) δ 207.9, 144.1, 132.0, 69.0, 50.9, 41.5, 31.8, 0.4, 0.3. IR (film) $\tilde{\nu}$ 2956, 2899, 1720, 1607, 1415, 1358, 1248, 1085, 1014, 835, 762, 689 cm^{-1}. HRMS (ESI) *m/z* calcd. for $C_{13}H_{28}O_2Si_2Na$ [M+Na]$^+$: 295.15200, found: 295.15182.

Assembly of Fragment C

Alcohol S6. Distilled water (2.0 mL) was added to a solution of compound **11** (2.12 g, 5.00 mmol,) in

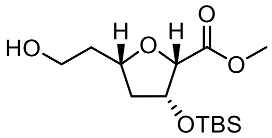

CH_2Cl_2 (40 mL), followed by DDQ (1.36 mg, 6.00 mmol). The mixture was stirred in the dark (wrapping the flask with aluminum foil) for 1 h before sat. aq. $NaHCO_3$ (100 mL) was added. The resulting mixture was vigorously stirred for 30 min before the aqueous phase was extracted with CH_2Cl_2 (3 x 30 mL). The combined organic phases were washed with brine (50 mL) and dried over Na_2SO_4 before being filtered and concentrated under reduced pressure. The residue was purified by flash chromatography (silica; hexanes/EtOAc 1:1) to afford the title compound as a colorless oil (1.57 g, quant.). $[\alpha]_D^{20}$ = −20.1 (c = 0.82, $CHCl_3$); ^{1}H NMR (400 MHz, $CDCl_3$): δ = 4.66 – 4.60 (m, 1H), 4.44 (dd, *J* = 5.4, 1.0 Hz, 1H), 4.38 – 4.27 (m, 1H), 3.89 – 3.76 (m, 2H), 3.74 (s, 3H), 3.06 – 3.01 (m, 1H), 2.26 (ddd, *J* = 13.2, 7.6, 5.9 Hz, 1H), 2.15 – 2.04 (m, 1H), 1.92 – 1.78 (m, 2H), 0.87 (s, 9H), 0.08 (s, 3H), 0.05 (s, 3H) ppm; ^{13}C NMR (101 MHz, $CDCl_3$): δ = 170.5, 82.6, 79.7, 74.0, 61.0, 52.0, 40.2, 37.5, 25.7 (3C), 18.0, −4.7, −5.2. ppm; IR (film) $\tilde{\nu}$ = 3456, 2952, 2930, 2887, 2857, 1754, 1438, 1255, 1208, 1098 cm^{-1}; HRMS (ESI): *m/z:* calcd. for $C_{14}H_{28}O_5SiNa$ [M+Na]$^+$: 327.15982, found: 327.15938.

Compound 19. Dess-Martin periodinane (4.24 g, 10.00 mmol) was slowly added at 0 °C (bath

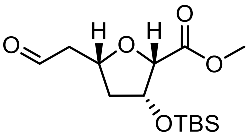

temperature) to a mixture of alcohol **S6** (1.52 g, 5.00 mmol) and $NaHCO_3$ (1.68 g, 20.00 mmol) in CH_2Cl_2 (20 mL). Stirring was continued at room temperature for 2 h before sat. aq. $Na_2S_2O_3$ solution (50 mL) and H_2O (50 mL) were introduced. The organic layer was separated and the aqueous phase was extracted with CH_2Cl_2 (3 x 20 mL). The combined organic layers were washed with sat. aq. $NaHCO_3$ solution (3 x 50 mL), dried over Na_2SO_4, and concentrated under reduced pressure. The residue was purified by flash chromatography (silica gel, hexane/EtOAc = 2:1) to give the desired product as a colorless oil (1.17 g, 77%). $[\alpha]_D^{20}$ = –16.5° (*c* = 2.86, $CHCl_3$). ^{1}H NMR (400 MHz, $CDCl_3$) δ 9.84 (t, *J* = 1.5 Hz, 1H), 4.63 (td, *J* = 5.8, 4.2 Hz, 1H), 4.55 (tt, *J* = 7.1, 6.0 Hz, 1H), 4.45 (d, *J* = 5.6 Hz, 1H), 3.71 (s, 3H), 3.13 (ddd, *J* = 17.4, 6.9, 1.5 Hz, 1H), 2.80 (ddd, *J* = 17.4, 6.0, 1.5 Hz, 1H), 2.35 (ddd, *J* = 13.2, 7.4, 6.0 Hz, 1H), 1.78 (ddd, *J* = 12.9, 5.9, 4.2 Hz, 1H),

0.84 (s, 9H), 0.05 (s, 3H), 0.04 (s, 3H). ^{13}C NMR (101 MHz, $CDCl_3$) δ 201.3, 170.4, 82.3, 74.4, 73.9, 51.9, 50.1, 40.2, 25.7, 17.9, –4.8, –5.2. IR (film) $\tilde{\nu}$ 2952, 2930, 2896, 2857, 1723, 1472, 1437, 1386, 1255, 1206, 1096, 1064, 937, 835, 777 cm^{-1}. HRMS (ESI) *m/z* calcd. for $C_{14}H_{26}O_5SiNa$ $[M+Na]^+$: 325.14417, found: 325.14432.

Compounds 20 and 21. An oven-dried Schlenk tube was charged with aldehyde **19** (1.42 g, 4.70 mmol), alkenylsilane **18** (1.54 g, 5.64 mmol) and CH_2Cl_2 (50 mL). The solution was stirred at –78 °C (bath temperature) while a solution of TMSOTf (0.17 mL, 0.94 mmol) in CH_2Cl_2 (3 mL) was added dropwise. Stirring was continued at –78 °C for 4 h and then at 0 °C for another 10 min before the reaction was quenched with sat. aq. $NaHCO_3$ solution (20 mL). The aqueous phase was extracted with CH_2Cl_2 (3 x 20 mL), the combined organic layers were washed with brine (50 mL), dried over Na_2SO_4, and concentrated under reduced pressure to give a crude residue that was used in the next step without further purification.

Imidazole (1.12 g, 16.43 mmol), DMAP (86.0 mg, 0.70 mmol) and TBSCl (2.12 g, 14.08 mmol) were added at 0 °C to a solution of the crude product in DMF (10 mL). The mixture was stirred at room temperature for 18 h before the reaction was quenched with brine (100 mL). The mixture was extracted with EtOAc (3 x 30 mL), the combined organic layers were washed with brine (50 mL), dried over Na_2SO_4 and concentrated under reduced pressure. The residue was purified by flash chromatography (silica; hexane/EtOAc, 3:1 to 2:1) to give compound **20** as a yellow oil (1.35 g, 70%) and a second fraction consisting of the *trans*-diastereomer as a yellow oil (0.38 g, 20%).

Analytical and spectroscopic data of the major cis-diastereomer **20**: $[\alpha]_D^{20}$ = –1.1° (*c* = 1.22, $CHCl_3$). ^{1}H

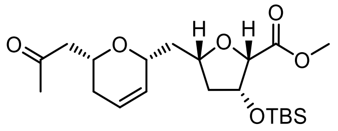

NMR (600 MHz, $CDCl_3$) δ 5.82 – 5.75 (m, 1H), 5.70 – 5.66 (m, 1H), 4.60 (td, *J* = 6.2, 5.0 Hz, 1H), 4.37 (d, *J* = 5.8 Hz, 1H), 4.28 – 4.22 (m, 1H), 4.21 – 4.15 (m, 1H), 4.02 – 3.95 (m, 1H), 3.71 (s, 3H), 2.69 (dd, *J* = 15.4, 8.2 Hz, 1H), 2.46 (dd, *J* = 15.5, 4.6 Hz, 1H), 2.23 (ddd, *J* = 12.9, 6.8, 6.3 Hz, 1H), 2.18 (s, 3H), 2.12 (ddd, *J* = 13.6, 8.0, 5.5 Hz, 1H), 1.98 (dtd, *J* = 6.6, 3.4, 1.9 Hz, 2H), 1.91 (ddd, *J* = 13.8, 8.0, 4.7 Hz, 1H), 1.79 (ddd, *J* = 12.6, 7.3, 5.0 Hz, 1H), 0.85 (s, 9H), 0.07 (s, 3H), 0.04 (s, 3H). ^{13}C NMR (151 MHz, $CDCl_3$) δ 207.5, 170.6, 130.0, 124.3, 81.9, 76.4, 74.1, 72.8, 70.7, 51.8, 49.9, 41.2, 40.1, 31.2, 30.9, 25.7, 18.0, –4.7, –5.1. IR (film) $\tilde{\nu}$ 2952, 2929, 2896, 2857, 1766, 1716, 1472, 1436, 1361, 1256, 1206, 1099, 938, 837, 778 cm^{-1}. HRMS (ESI) *m/z* calcd. for $C_{21}H_{36}O_6SiNa$ $[M+Na]^+$: 435.21734, found: 435.21786.

Analytical and spectroscopic data of the minor trans-diastereomer **21**: $[\alpha]_D^{20}$ = +26.1° (*c* = 0.75, $CHCl_3$).

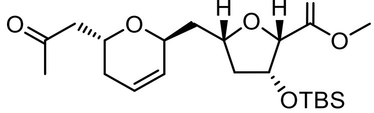

^{1}H NMR (600 MHz, $CDCl_3$) δ 5.79 – 5.75 (m, 1H), 5.74 – 5.70 (m, 1H), 4.61 (td, *J* = 6.4, 4.9 Hz, 1H), 4.52 – 4.45 (m, 1H), 4.40 (d, *J* = 5.9 Hz, 1H), 4.19 (dtd, *J* = 9.8, 7.1, 3.1 Hz, 1H), 4.12 (tt, *J* = 8.3, 3.9 Hz, 1H), 3.71 (s, 3H), 2.76 (dd, *J* = 15.8, 8.3 Hz, 1H), 2.47 (dd, *J* = 16.1, 4.4 Hz, 1H), 2.30 (ddd, *J* = 12.7, 6.8, 6.4

Hz, 1H), 2.18 (s, 3H), 2.01 – 1.92 (m, 3H), 1.91 – 1.85 (m, 1H), 1.72 (ddd, *J* = 12.6, 7.4, 5.0 Hz, 1H), 0.85 (s, 9H), 0.06 (s, 3H), 0.04 (s, 3H). ^{13}C NMR (151 MHz, $CDCl_3$) δ 207.4, 170.8, 130.3, 123.3, 82.1, 75.9, 74.1, 70.3, 64.0, 51.7, 49.1, 40.9, 39.6, 31.4, 30.4, 25.7, 18.0, –4.7, –5.1. IR (film) $\tilde{\nu}$ 2951, 2928, 2857, 1764, 1717, 1463, 1436, 1360, 1256, 1206, 1100, 938, 838, 778 cm^{-1}. HRMS (ESI) *m/z* calcd. for $C_{21}H_{36}O_6SiNa$ $[M+Na]^+$: 435.21734, found: 435.21767.

Compound S7. A dry flask was charged with *cis*-dihydropyran **20** (1.24 g, 3.00 mmol), THF (22.5 mL), H_2O (7.5 mL) and 4-methylmorpholine N-oxide (NMO, 0.70 g, 6.00 mmol). This mixture was stirred at room temperature while a solution of OsO_4 (4% *w/w* in water, 0.95 mL, 0.15 mmol) was added. Stirring was continued at room temperature for 24 h before the reaction was quenched with sat. aq. Na_2SO_3 solution (50 mL). The aqueous phase was extracted with EtOAc (3 x 30 mL), the combined organic layers were washed with brine (50 mL), dried over Na_2SO_4, and concentrated under reduced pressure. The residue was purified by flash chromatography (silica; hexane/EtOAc, 1:1 to 1:3) to give the title compound as a white solid (1.07 g, 80%, dr = 9:1 (^{1}H NMR)). $[\alpha]_D^{20}$ = –27.5° (*c* = 1.01, $CHCl_3$). ^{1}H NMR (600 MHz, C_6D_6) δ 5.26 (br, 1H), 4.63 (ddt, *J* = 10.5, 8.5, 2.0 Hz, 1H), 4.49 – 4.40 (m, 1H), 4.26 (q, *J* = 3.0 Hz, 1H), 4.21 (d, *J* = 4.8 Hz, 1H), 4.12 (dd, *J* = 10.0, 2.9 Hz, 1H), 4.08 – 4.03 (m, 2H), 3.31 (s, 3H), 2.72 (br, 1H), 2.58 (ddd, *J* = 15.2, 10.5, 3.4 Hz, 1H), 2.35 (dd, *J* = 15.0, 9.1 Hz, 1H), 1.96 (dd, *J* = 15.3, 4.0 Hz, 1H), 1.84 (ddd, *J* = 13.8, 3.3, 2.2 Hz, 1H), 1.78 (s, 3H), 1.71 (ddd, *J* = 15.1, 3.6, 1.7 Hz, 1H), 1.60 (ddd, *J* = 13.4, 8.5, 5.0 Hz, 1H), 1.35 – 1.29 (m, 2H), 0.84 (s, 9H), –0.12 (s, 3H), –0.13 (s, 3H). ^{13}C NMR (151 MHz, C_6D_6) δ 204.7, 171.7, 83.5, 76.9, 74.3, 73.9, 70.0, 69.0, 68.2, 51.6, 49.9, 41.2, 38.8, 38.0, 29.7, 25.7, 17.9, –4.8, –5.5. IR (film) $\tilde{\nu}$ 3460, 2953, 2927, 2857, 1748, 1714, 1463, 1438, 1361, 1256, 1216, 1095, 941, 837, 779 cm^{-1}. HRMS (ESI) *m/z* calcd. for $C_{21}H_{38}O_8SiNa$ $[M+Na]^+$: 469.22282, found: 469.22309.

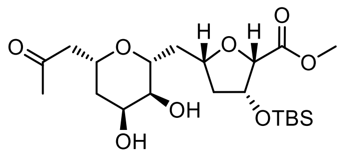

Compound 22. An oven-dried flask was charged with diol **S7** (0.82 g, 1.84 mmol) and CH_2Cl_2 (10 mL). The solution was stirred at –78 °C (bath temperature) while 2,6-lutidine (1.07 mL, 9.18 mmol) was added, followed by slow addition of TBSOTf (2.10 mL, 9.18 mmol). The resulting mixture was stirred at 0 °C for 2 h before the reaction was quenched with sat. aq. $NaHCO_3$ solution (20 mL). The mixture was extracted with CH_2Cl_2 (3 x 20 mL) and the combined organic layers were washed with brine (20 mL).

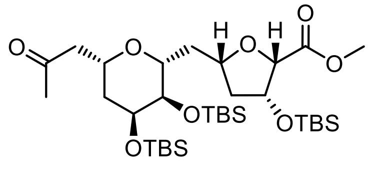

HCl (1.0 M, 20 mL) was added to the organic phase and the mixture was stirred vigorously at room temperature for 2 h. The organic layer was separated, washed with brine (20 mL), dried over Na_2SO_4, and concentrated under reduced pressure. The residue was purified by flash chromatography (silica; pentane/EtOAc, 5:1 to 2:1) to give the title compound as a colorless oil (1.21 g, 98%). $[\alpha]_D^{20}$ = +52.4° (*c* = 1.13, $CHCl_3$). ^{1}H NMR (400 MHz, $CDCl_3$) δ 4.59 (q, *J* = 6.1 Hz, 1H), 4.33 (d, *J* = 6.1 Hz, 1H), 4.17 (dddd,

J = 10.6, 8.4, 4.8, 2.2 Hz, 1H), 4.08 (dddd, J = 10.0, 8.5, 6.5, 4.4 Hz, 1H), 3.98 (dt, J = 4.2, 2.1 Hz, 1H), 3.76 – 3.66 (m, 4H), 3.27 (dd, J = 9.0, 2.3 Hz, 1H), 2.58 (dd, J = 15.2, 8.4 Hz, 1H), 2.32 (dd, J = 15.2, 4.8 Hz, 1H), 2.25 (dt, J = 12.8, 6.5 Hz, 1H), 2.13 (s, 3H), 2.05 (ddd, J = 12.8, 9.9, 2.7 Hz, 1H), 1.85 (ddd, J = 13.3, 10.9, 4.4 Hz, 1H), 1.75 – 1.64 (m, 2H), 1.48 (ddd, J = 13.4, 11.3, 2.0 Hz, 1H), 0.90 (s, 9H), 0.90 (s, 9H), 0.84 (s, 9H), 0.08 (s, 3H), 0.06 (s, 3H), 0.06 (s, 6H), 0.05 (s, 3H), 0.03 (s, 3H). ^{13}C NMR (101 MHz, $CDCl_3$) δ 207.3, 170.5, 81.8, 77.0, 74.4, 74.0, 73.4, 69.6, 68.2, 51.7, 49.3, 40.0, 39.8, 37.9, 31.1, 26.3, 26.0, 25.8, 18.3, 18.2, 18.1, –3.3, –4.0, –4.4, –4.5, –4.8, –5.1. IR (film) $\tilde{\nu}$ 2952, 2929, 2887, 2857, 1739, 1719, 1472, 1463, 1360, 1254, 1128, 1098, 952, 836, 776 cm^{-1}. HRMS (ESI) *m/z* calcd. for $C_{33}H_{66}O_8Si_3Na$ [M+Na]$^+$: 697.39578, found: 697.39620.

Compound S8. A solution of *tert*-butyl diethylphosphonoacetate (3.29 g, 13.03 mmol) in THF (13 mL) was added dropwise at 0 °C (bath temperature) to a stirred suspension of NaH (0.25 g, 10.43 mmol) in THF (13 mL). After stirring at 0 °C for 10 min and for another 30 min at room temperature, LiBr (1.13 g, 13.03 mmol) was added and stirring continued at room temperature for 5 min. The suspension was cooled to 0 °C (bath temperature) before a solution of ketone **22** (0.88 g, 1.30 mmol) in THF (13 mL) was added dropwise. The mixture was stirred at 0 °C for 10 min and then at room temperature for 15 h. The reaction was quenched with sat. aq. NH_4Cl solution (30 mL) and the aqueous phase extracted with EtOAc (3 x 30 mL). The combined organic layers were washed with brine (30 mL), dried over Na_2SO_4, and concentrated under reduced pressure. The residue was purified by flash chromatography (silica; hexane/EtOAc, 20:1 to 10:1) to give *E*-isomer as a pale-yellow oil (0.68 g, 68%) and a second fraction comprised of the corresponding *Z*-isomer as a pale-yellow oil (0.25 g, 25%).

Analytical and spectroscopic data of the major isomer **(*E*)-S8**: $[\alpha]_D^{20}$ = +45.4° (*c* = 0.89, $CHCl_3$). ^{1}H NMR

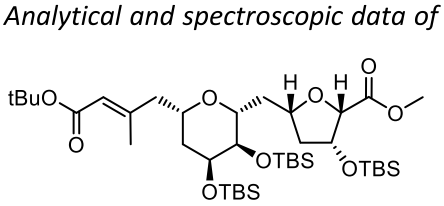

(600 MHz, $CDCl_3$) δ 5.59 – 5.52 (m, 1H), 4.65 – 4.56 (m, 1H), 4.34 (d, J = 6.1 Hz, 1H), 4.16 (dddd, J = 10.3, 8.3, 6.6, 4.4 Hz, 1H), 4.00 (dt, J = 4.2, 2.1 Hz, 1H), 3.91 (dddd, J = 11.0, 8.0, 5.1, 2.1 Hz, 1H), 3.73 – 3.65 (m, 4H), 3.29 (dd, J = 9.0, 2.4 Hz, 1H), 2.29 – 2.20 (m, 2H), 2.10 (d, J = 1.4 Hz, 3H), 2.09 – 2.03 (m, 2H), 1.93 – 1.86 (m, 1H), 1.72 – 1.65 (m, 2H), 1.50 – 1.44 (m, 10H), 0.91 (s, 9H), 0.90 (s, 9H), 0.85 (s, 9H), 0.09 (s, 3H), 0.07 (s, 3H), 0.06 (s, 3H), 0.06 (s, 3H), 0.04 (s, 6H). ^{13}C NMR (151 MHz, $CDCl_3$) δ 170.5, 166.3, 154.9, 119.3, 81.9, 79.6, 77.1, 74.7, 74.1, 73.4, 69.7, 69.4, 51.7, 46.6, 40.1, 39.6, 38.0, 28.4, 26.3, 26.0, 25.8, 19.1, 18.3, 18.3, 18.1, –3.3, –4.0, –4.4, –4.4, –4.7, –5.1. IR (film) $\tilde{\nu}$ 2952, 2929, 2886, 2857, 1740, 1711, 1649, 1472, 1463, 1364, 1253, 1137, 1099, 966, 836, 775 cm^{-1}. HRMS (ESI) *m/z* calcd. for $C_{39}H_{76}O_9Si_3Na$ [M+Na]$^+$: 795.46893, found: 795.46897.

Analytical and spectroscopic data of the minor isomer ***(Z)*-S8**: $[\alpha]_D^{20}$ = +16.4° (*c* = 0.92, $CHCl_3$). ^1H NMR

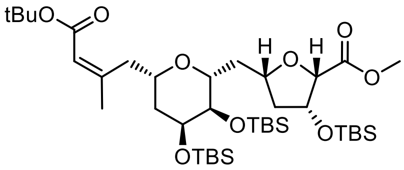

(600 MHz, $CDCl_3$) δ 5.65 – 5.58 (m, 1H), 4.61 (dt, *J* = 6.6, 6.0 Hz, 1H), 4.35 (d, *J* = 6.2 Hz, 1H), 4.14 (dddd, *J* = 10.7, 8.7, 6.4, 4.4 Hz, 1H), 3.99 (dt, *J* = 4.3, 2.2 Hz, 1H), 3.94 – 3.88 (m, 1H), 3.70 (s, 3H), 3.70 – 3.65 (m, 1H), 3.30 (dd, *J* = 9.0, 2.4 Hz, 1H), 2.92 (ddd, *J* = 12.7, 4.4, 1.0 Hz, 1H), 2.48 (dd, *J* = 12.7, 8.4 Hz, 1H), 2.22 (dt, *J* = 12.9, 6.5 Hz, 1H), 2.05 (ddd, *J* = 13.1, 10.2, 2.8 Hz, 1H), 1.92 – 1.88 (m, 1H), 1.87 (d, *J* = 1.4 Hz, 3H), 1.75 (ddd, *J* = 13.6, 4.0, 2.1 Hz, 1H), 1.70 (ddd, *J* = 12.5, 8.7, 5.9 Hz, 1H), 1.56 – 1.52 (m, 1H), 1.45 (s, 9H), 0.91 (s, 9H), 0.89 (s, 9H), 0.86 (s, 9H), 0.09 (s, 3H), 0.07 (s, 3H), 0.06 (s, 6H), 0.06 (s, 3H), 0.04 (s, 3H). ^{13}C NMR (151 MHz, $CDCl_3$) δ 170.6, 165.8, 156.7, 118.9, 81.7, 79.5, 77.2, 74.7, 74.1, 73.2, 71.6, 69.9, 51.7, 40.2, 39.7, 38.6, 37.9, 28.4, 26.5, 26.4, 26.0, 25.8, 18.3, 18.3, 18.1, –3.3, –4.0, –4.4, –4.5, –4.7, –5.0. IR (film) $\tilde{\nu}$ 2952, 2929, 2886, 2857, 1741, 1707, 1645, 1472, 1463, 1364, 1253, 1141, 1099, 959, 836, 775 cm^{-1}. HRMS (ESI) *m/z* calcd. for $C_{39}H_{76}O_9Si_3Na$ $[M+Na]^+$: 795.46893, found: 795.46914.

3-Pentyn-2-one (24). An oven-dried Schlenk tube was charged with $AlCl_3$ (13.33 g, 100.0 mmol) and CH_2Cl_2 (100 mL). The mixture was cooled to 0 °C (bath temperature) before a solution of 1-trimethylsilylpropyne (14.8 mL, 100.0 mmol) and acetyl chloride (7.1 mL, 100.0 mmol) in CH_2Cl_2 (20 mL) was added over the course of 1.5 h. The resulting suspension was allowed to reach room temperature to give a reddish-brown mixture, which was carefully poured into ice/water. The organic layer was separated and the aqueous phase was extracted with CH_2Cl_2 (20 mL). The combined organic layers were washed with water (20 mL), dried over Na_2SO_4, and concentrated under reduced pressure to give a greenish-black liquid. Upon distillation at 180 mbar, the fraction boiling between 64 °C and 70 °C was collected. This product was carefully re-distilled at 210 mbar to give 3-pentyn-2-one as a colorless liquid (5.07 g, 62%). bp = 81-85 °C. ^1H NMR (400 MHz, $CDCl_3$) δ 2.30 (s, 3H), 2.01 (s, 3H). ^{13}C NMR (101 MHz, $CDCl_3$) δ 185.0, 89.9, 80.8, 32.8, 4.1. The spectral data match the literature.[5]

Compound 25. *Preparation of a Stock Solution of CF_3CH_2OLi:* An oven-dried Schlenk tube was charged with CF_3CH_2OH (10.0 mg, 0.10 mmol) and THF (1.0 mL). The solution was stirred at –40 °C (bath temperature) when *n*BuLi (1.6 M in hexanes, 62.5 µL, 0.10 mmol) was added dropwise. The mixture was stirred at room temperature for 1 h to give the required CF_3CH_2OLi stock solution (0.10 M in THF).

To an oven-dried flask was charged with ester ***(E)*-S8** (790.0 mg, 1.02 mmol) and CH_2Cl_2 (10 mL). The solution was stirred at –95 °C (acetone/liquid nitrogen bath) for 5 min before DIBAL-H (1.0 M in hexane, 1.50 mL, 1.50 mmol) was slowly added. After stirred at –95 °C for 1 h, the reaction was quenched with sat. Rochelle salt solution (20 mL). The resulting mixture was stirred vigorously at room temperature for 1 h before the aqueous phase was extracted with CH_2Cl_2 (3 x 20 mL). The combined organic layers

were dried over $MgSO_4$ and concentrated under reduced pressure to give the crude aldehyde **23** (810.0 mg) which was used in the next step without further purification.

An oven-dried Schlenk tube was charged with (*S*)-(+)-DTBM-SEGPHOS (56.5 mg, 48.0 µmol), $CuBF_4 \cdot 4CH_3CN$ (15.1 mg, 48.0 µmol) and THF (0.2 mL). The mixture was stirred at room temperature for 20 min before CF_3CH_2OH (38.4 mg, 0.38 mmol) and ynone **24** (118.0 mg, 1.44 mmol) were added. The mixture was stirred at –40 °C (bath temperature) while an aliquot of the CF_3CH_2OLi stock solution (0.10 M in THF, 0.48 mL, 48.0 µmol) was added, followed by the slow addition of a solution of the crude aldehyde **23** (760 mg) in THF (1.2 mL). The mixture was stirred at –40 °C for 60 h before the reaction was quenched with sat. aq. NH_4Cl solution (10 mL). The mixture was then allowed to warm to room temperature and the resulting mixture was extracted with *tert*-butyl methyl ether (3 x 10 mL). The combined organic layers were dried over Na_2SO_4 and concentrated under reduced pressure, and the residue was purified by flash chromatography (silica; hexane/EtOAc, 10:1 to 5:1) to give the major diastereomer as a pale-yellow oil (474.1 mg, 60% over two steps, 83 % brsm), the minor diastereomer as a pale-yellow oil (94.0 mg, 12% over two steps, 16 % brsm), as well as recovered starting material (209.0 mg, 28%).

Analytical and spectroscopic data of the major isomer **25**: $[\alpha]_D^{20}$ = +30.6° (*c* = 2.90, $CHCl_3$). ^{1}H NMR (400 MHz, $CDCl_3$) δ 5.56 (q, *J* = 1.3 Hz, 1H), 4.52 (dt, *J* = 6.7, 5.5 Hz, 1H), 4.43 – 4.35 (m, 1H), 4.04 – 3.95 (m, 2H), 3.94 – 3.87 (m, 1H), 3.73 – 3.63 (m, 1H), 3.55 (dd, *J* = 7.3, 5.6 Hz, 1H), 3.26 (dd, *J* = 9.1, 2.3 Hz, 1H), 3.11 (d, *J* = 4.2 Hz, 1H), 2.97 (dd, *J* = 16.7, 3.0 Hz, 1H), 2.73 (dd, *J* = 16.8, 9.4 Hz, 1H), 2.30 – 2.23 (m, 2H), 2.10 (d, *J* = 1.3 Hz, 3H), 2.06 (ddd, *J* = 14.1, 5.4, 1.0 Hz, 1H), 2.00 (s, 3H), 1.81 – 1.75 (m, 2H), 1.70 – 1.65 (m, 1H), 1.55 – 1.50 (m, 1H), 1.47 – 1.42 (m, 10H), 0.90 (s, 18H), 0.89 (s, 9H), 0.10 (s, 3H), 0.10 (s, 3H), 0.07 (s, 3H), 0.06 (s, 3H), 0.05 (s, 3H), 0.03 (s, 3H). ^{13}C NMR (101 MHz, $CDCl_3$) δ 187.4, 166.3, 154.8, 119.3, 90.4, 82.4, 80.6, 79.7, 75.3, 74.5, 73.8, 73.3, 69.6, 69.4, 67.5, 49.5, 46.6, 40.6, 40.0, 38.3, 28.4, 26.3, 26.0, 26.0, 19.1, 18.3, 18.2, 18.1, 4.3, –3.3, –4.0, –4.3, –4.5, –4.5, –5.0. IR (film) ṽ 3506, 2953, 2929, 2886, 2857, 2222, 1711, 1672, 1472, 1462, 1364, 1252, 1135, 1085, 968, 835, 774 cm^{-1}. HRMS (ESI) *m/z* calcd. for $C_{43}H_{80}O_9Si_3Na$ [M+Na]$^+$: 847.50024, found: 847.50072.

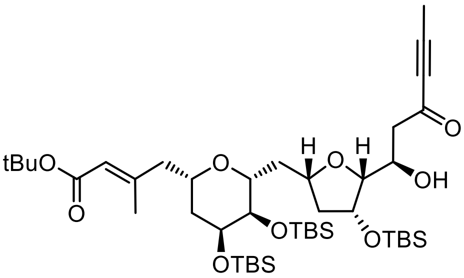

Analytical and spectroscopic data of the minor isomer **S9***:* $[\alpha]_D^{20}$ = +36.0° (*c* = 1.24, $CHCl_3$). ^1H NMR (400

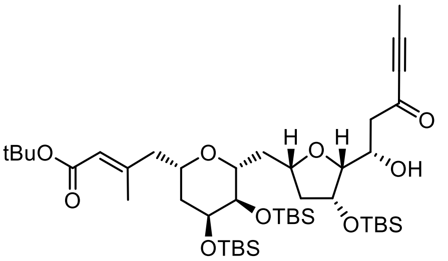

MHz, $CDCl_3$) δ 5.56 (q, *J* = 1.2 Hz, 1H), 4.53 – 4.38 (m, 2H), 4.01 – 3.88 (m, 3H), 3.70 (td, *J* = 9.8, 3.1 Hz, 1H), 3.55 (dd, *J* = 5.6, 4.2 Hz, 1H), 3.27 (dd, *J* = 9.1, 2.3 Hz, 1H), 3.20 (d, *J* = 2.4 Hz, 1H), 2.80 (d, *J* = 6.5 Hz, 2H), 2.35 – 2.22 (m, 2H), 2.10 (d, *J* = 1.3 Hz, 3H), 2.09 – 2.03 (m, 1H), 2.00 (s, 3H), 1.91 – 1.77 (m, 2H), 1.67 (ddd, *J* = 13.6, 4.1, 2.1 Hz, 1H), 1.53 (ddd, *J* = 12.8, 8.7, 5.1 Hz, 1H), 1.49 – 1.42 (m, 10H), 0.90 (s, 9H), 0.89 – 0.88 (m, 18H), 0.10 (s, 3H), 0.09 (s, 3H), 0.07 (s, 3H), 0.06 (s, 3H), 0.05 (s, 3H), 0.03 (s, 3H). ^{13}C NMR (101 MHz, $CDCl_3$) δ 186.3, 166.3, 154.9, 119.3, 90.3, 82.4, 80.5, 79.6, 75.3, 74.4, 74.4, 73.5, 69.6, 69.4, 67.1, 49.1, 46.6, 41.7, 39.9, 38.0, 28.4, 26.3, 26.0, 25.9, 19.1, 18.3, 18.2, 18.0, 4.2, –3.3, –4.0, –4.3, –4.5 (two peaks), –5.0. IR (film) $\tilde{\nu}$ 3520, 2953, 2929, 2887, 2857, 2218, 1711, 1675, 1650, 1472, 1463, 1364, 1253, 1136, 1085, 972, 836, 775 cm^{-1}. HRMS (ESI) *m/z* calcd. for $C_{43}H_{80}O_9Si_3Na$ $[M+Na]^+$: 847.50024, found: 847.50056.

Compound 26. An oven-dried flask was charged with RuCl[(*R*,*R*)-TsDPEN](mesitylene) (19.3 mg, 31.1 µmol) and CH_2Cl_2 (5 mL). The mixture was stirred at room temperature while HCOOH (0.23 mL, 6.22 mmol) and Et_3N (0.35 mL, 2.50 mmol) were added sequentially, followed by a solution of ynone **25** (513.0 mg, 0.62 mmol) in CH_2Cl_2 (15 mL). The solution was stirred at room temperature for 19 h before the reaction was quenched with sat. aq. $NaHCO_3$ solution (10 mL). The mixture was extracted with CH_2Cl_2 (3 x 20 mL), the combined organic layers were washed with brine (10 mL), dried over Na_2SO_4, and concentrated under reduced pressure. The residue was purified by flash chromatography (silica; hexane/EtOAc, 5:1) to give the major diastereomer as a pale-yellow oil (352.0 mg, 68%), along with a second fraction comprising the minor diastereomer as a yellow oil (81.2 mg, 16%).

Analytical and spectroscopic data of the major isomer **26**: $[\alpha]_D^{20}$ = +30.7° (*c* = 0.61, $CHCl_3$). ^1H NMR (400

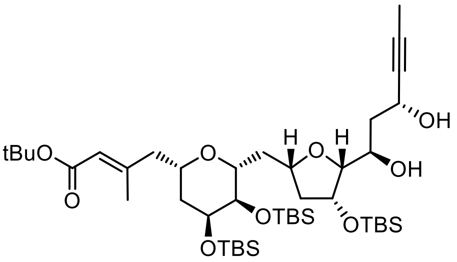

MHz, $CDCl_3$) δ 5.56 (q, *J* = 1.1 Hz, 1H), 4.71 – 4.53 (m, 2H), 4.33 (td, *J* = 8.4, 3.7 Hz, 1H), 4.01 – 3.87 (m, 3H), 3.74 – 3.41 (m, 4H), 3.26 (dd, *J* = 9.1, 2.3 Hz, 1H), 2.32 – 2.23 (m, 2H), 2.10 (d, *J* = 1.4 Hz, 3H), 2.09 – 2.03 (m, 1H), 2.03 – 1.89 (m, 2H), 1.82 (d, *J* = 2.2 Hz, 3H), 1.81 – 1.75 (m, 2H), 1.67 (ddd, *J* = 13.6, 4.1, 2.1 Hz, 1H), 1.57 – 1.50 (m, 1H), 1.46 (s, 10H), 0.90 (s, 9H), 0.90 (s, 9H), 0.89 (s, 9H), 0.12 (s, 3H), 0.11 (s, 3H), 0.07 (s, 3H), 0.06 (s, 3H), 0.05 (s, 3H), 0.03 (s, 3H). ^{13}C NMR (101 MHz, $CDCl_3$) δ 166.3, 154.8, 119.3, 81.9, 80.7, 80.3, 79.7, 75.4, 74.5, 74.4, 73.4, 69.8, 69.6, 69.4, 61.3, 46.6, 40.6, 40.1, 40.0, 38.2, 28.4, 26.3, 26.0, 25.9, 19.0, 18.3, 18.2, 18.0, 3.8, –3.3, –4.0, –4.1, –4.5 (two peaks), –5.0. IR (film) $\tilde{\nu}$ 3462, 2953, 2928, 2857, 1712, 1647, 1472, 1463, 1365, 1253, 1137, 1084, 952, 835, 775 cm^{-1}. HRMS (ESI) *m/z* calcd. for $C_{43}H_{82}O_9Si_3Na$ $[M+Na]^+$: 849.51588, found: 849.51567.

Analytical and spectroscopic data of the minor isomer **S10**: $[\alpha]_D^{20}$ = +34.9° (*c* = 0.57, $CHCl_3$). ^1H NMR (400

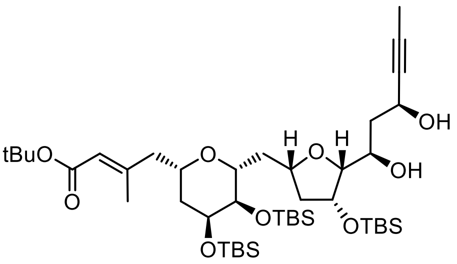

MHz, $CDCl_3$) δ 5.56 (q, *J* = 1.3 Hz, 1H), 4.63 (ddd, *J* = 9.0, 3.5, 2.0 Hz, 1H), 4.58 (dd, *J* = 6.3, 6.3 Hz, 1H), 4.08 (ddd, *J* = 9.6, 7.7, 3.3 Hz, 1H), 4.03 – 3.87 (m, 3H), 3.78 – 3.43 (m, 4H), 3.26 (dd, *J* = 9.1, 2.3 Hz, 1H), 2.32 – 2.23 (m, 2H), 2.13 – 2.03 (m, 5H), 1.95 – 1.81 (m, 4H), 1.78 (dd, *J* = 7.7, 6.1 Hz, 2H), 1.68 (ddd, *J* = 13.6, 4.1, 2.1 Hz, 1H), 1.56 – 1.44 (m, 11H), 0.92 – 0.88 (m, 27H), 0.13 (s, 3H), 0.12 (s, 3H), 0.07 (s, 3H), 0.07 (s, 3H), 0.05 (s, 3H), 0.04 (s, 3H). ^{13}C NMR (101 MHz, $CDCl_3$) δ 166.3, 154.8, 119.3, 82.1, 80.4, 80.2, 79.7, 75.4, 74.5, 74.4, 73.3, 71.6, 69.6, 69.4, 62.7, 46.6, 41.5, 40.5, 40.0, 38.4, 28.4, 26.3, 26.0, 25.9, 19.1, 18.3, 18.2, 18.0, 3.8, –3.3, –4.0, –4.1, –4.5, –4.5, –5.0. IR (film) $\tilde{\nu}$ 3447, 2953, 2928, 2887, 2857, 1712, 1649, 1472, 1463, 1365, 1253, 1137, 1085, 950, 836, 775 cm^{-1}. HRMS (ESI) *m/z* calcd. for $C_{43}H_{82}O_9Si_3Na$ $[M+Na]^+$: 849.51588, found: 849.51576.

Mosher ester analysis of the major diastereomer 26. Reaction of **26** with (*S*)-(+)-MTPA-Cl under

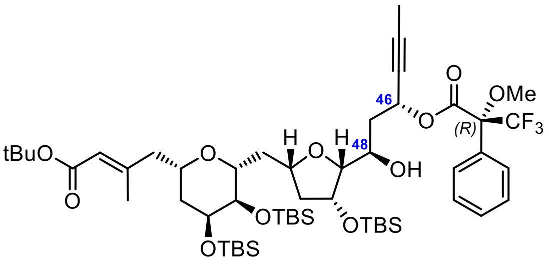

standard conditions (CH_2Cl_2, pyridine, 0°C to RT) furnished the corresponding (*R*)-(+)-MTPA ester as a colorless oil (1.13 mg, 60%). $[\alpha]_D^{20}$ = +61.8° (*c* = 0.11, $CHCl_3$). ^1H NMR (600 MHz, $CDCl_3$) δ 7.60 – 7.56 (m, 2H), 7.40 – 7.37 (m, 3H), 5.79 (ddt, *J* = 7.9, 3.5, 2.1 Hz, 1H), 5.56 (q, *J* = 1.2 Hz, 1H), 4.51 (q, *J* = 6.2 Hz, 1H), 4.01 – 3.96 (m, 1H), 3.94 – 3.87 (m, 2H), 3.80 – 3.74 (m, 1H), 3.66 (td, *J* = 9.4, 3.6 Hz, 1H), 3.57 (s, 3H), 3.44 (dd, *J* = 7.5, 5.9 Hz, 1H), 3.24 (dd, *J* = 9.1, 2.4 Hz, 1H), 2.99 (br, 1H), 2.34 – 2.25 (m, 2H), 2.24 – 2.19 (m, 1H), 2.11 (d, *J* = 1.4 Hz, 3H), 2.06 (dd, *J* = 14.0, 5.0 Hz, 1H), 1.91 – 1.84 (m, 4H), 1.75 – 1.71 (m, 2H), 1.67 (ddd, *J* = 13.4, 4.1, 2.0 Hz, 1H), 1.47 – 1.44 (m, 10H), 1.41 – 1.38 (m, 1H), 0.90 (s, 9H), 0.88 (s, 9H), 0.86 (s, 9H), 0.10 – 0.08 (m, 6H), 0.06 (s, 3H), 0.04 (s, 3H), 0.03 (s, 3H), 0.03 (s, 3H). ^{13}C NMR (151 MHz, $CDCl_3$) δ 166.3, 165.9, 154.9, 132.8, 129.6, 128.5, 127.7, 119.3, 82.8, 82.4, 79.7, 76.3, 75.1, 74.4, 74.4, 73.4, 69.6, 69.4, 66.9, 63.8, 55.5, 46.6, 40.7, 40.0, 39.3, 38.1, 28.4, 26.2, 26.0, 25.9, 19.0, 18.3, 18.2, 18.0, 3.7, –3.3, –4.0, –4.2, –4.5, –4.5, –5.0. ***Note****: the ^{13}C NMR signals of the CF_3 group and the adjacent quaternary carbon have not been detected.* IR (film) $\tilde{\nu}$ 3506, 2954, 2925, 2855, 1754, 1712, 1650, 1462, 1365, 1258, 1130, 1084, 1018, 836, 802, 777 cm^{-1}. HRMS (ESI) *m/z* calcd. for $C_{53}H_{89}F_3O_{11}Si_3Na$ $[M+Na]^+$: 1065.55570, found: 1065.55612.

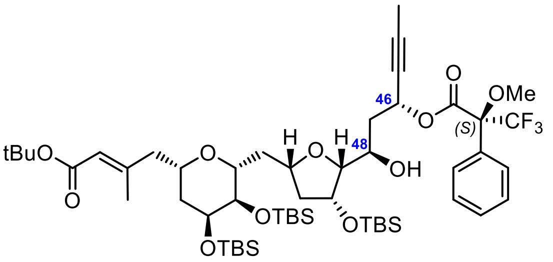

The analogous reaction of **26** with (*R*)-(–)-MTPA-Cl gave (*S*)-(+)-MTPA ester as a colorless oil (1.26 mg, 67%). $[\alpha]_D^{20}$ = +43.3° (*c* = 0.12, $CHCl_3$). ^1H NMR (600 MHz, $CDCl_3$) δ 7.57 – 7.53 (m, 2H), 7.40 – 7.36 (m, 3H), 5.81 – 5.72 (m, 1H), 5.56 (q, *J* = 1.1 Hz, 1H), 4.58 – 4.49 (m, 1H), 4.01 – 3.96 (m, 1H), 3.95 – 3.88 (m, 3H), 3.69 – 3.64 (m, 1H), 3.57 (s, 3H), 3.47 (dd, *J* = 7.4, 5.7 Hz, 1H), 3.26 (dd, *J* = 9.1, 2.4 Hz, 1H), 3.02 (br, 1H), 2.32 (ddd, *J* = 14.7, 10.1, 3.0 Hz, 1H), 2.29 – 2.24 (m, 2H), 2.10 (d, *J* = 1.4 Hz, 3H), 2.05 (dd, *J* = 14.0, 4.9 Hz, 1H), 1.91 (ddd, *J* = 14.4, 10.2, 3.6 Hz, 1H), 1.80 (d, *J* = 2.2 Hz, 3H), 1.78 – 1.75 (m, 2H), 1.67 (ddd, *J* = 13.4, 4.1, 2.0 Hz, 1H), 1.47 (s, 9H), 1.47 – 1.42 (m, 2H), 0.90 (s, 9H), 0.88 (s, 9H), 0.87 (s, 9H), 0.09 (s, 3H), 0.09 (s, 3H), 0.07 (s, 3H), 0.06 (s, 3H), 0.05 (s, 3H), 0.04 (s, 3H). ^{13}C NMR (151 MHz, $CDCl_3$) δ 166.3, 165.9, 154.9, 132.5, 129.6, 128.3, 127.7, 119.3, 82.8, 82.7, 79.7, 76.1, 75.3, 74.4, 74.3, 73.4, 69.6, 69.4, 67.0, 64.1, 55.8, 46.6, 41.1, 40.0, 39.3, 38.1, 28.4, 26.2, 26.0, 25.9, 19.1, 18.3, 18.2, 18.0, 3.7, –3.3, –3.9, –4.2, –4.5, –4.5, –5.0. ***Note**: the ^{13}C NMR signals of the CF_3 group and the adjacent quaternary carbon have not been detected.* IR (film) $\tilde{\nu}$ 3367, 2955, 2926, 2855, 1751, 1713, 1653, 1464, 1365, 1259, 1128, 1091, 1018, 836, 801, 777 cm^{-1}. HRMS (ESI) *m/z* calcd. for $C_{53}H_{89}F_3O_{11}Si_3Na$ $[M+Na]^+$: 1065.55570, found: 1065.55555.

Table S2. Determination of absolute configuration of the stereogenic center at C46 of compound **26** set by Noyori-transfer hydrogenation via Mosher ester analysis.[2]

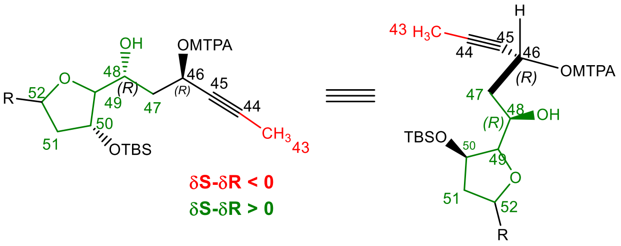

No.	$\delta_{H\ NMR}$ (*S*-ester) (ppm)	$\delta_{H\ NMR}$ (*R*-ester) (ppm)	$\Delta\delta$ (δ_S -δ_R, ppm)
43	1.80	1.84	–0.04
46	5.77	5.79	–0.02
47a	1.91	1.88	0.03
47b	2.32	2.31	0.01
48	3.92	3.77	0.15
48-OH	3.03	2.99	0.04
49	3.47	3.44	0.03
50	4.54	4.51	0.03
51a	1.44	1.39	0.05
51b	2.27	2.21	0.06
52	3.92	3.90	0.02

Isopropylidene Acetal S11. PPTS (0.1 mg, 0.4 µmol) was added to a solution of 1,3-diol **26** (2.0 mg, 2.4 µmol) in 2,2-dimethoxy propane (0.2 mL). The mixture was stirred at room temperature for 48 h before the reaction was quenched with sat. aq. $NaHCO_3$ (5 mL). The mixture was extracted with CH_2Cl_2 (3 x 5 mL), the combined extracts were washed with brine (10 mL), dried over Na_2SO_4, concentrated under reduced pressure, and the residue was purified by flash chromatography (silica; cyclohexane/EtOAc, 20:1 to 10:1) to give the title compound as a yellow oil (2.10 mg, quant.).

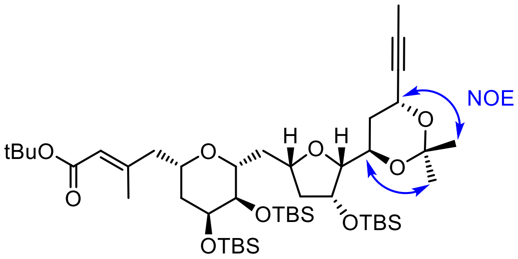

$[\alpha]_D^{20}$ = +24.5° (*c* = 0.29, CH_2Cl_2). ^1H NMR (600 MHz, CD_2Cl_2) δ 5.57 – 5.52 (m, 1H), 4.63 (tq, *J* = 4.8, 2.4 Hz, 1H), 4.40 (ddd, *J* = 6.7, 5.2, 3.9 Hz, 1H), 4.29 (dt, *J* = 9.8, 4.4 Hz, 1H), 4.02 (dt, *J* = 4.4, 2.3 Hz, 1H), 3.97 – 3.90 (m, 2H), 3.72 (ddd, *J* = 10.5, 9.1, 2.9 Hz, 1H), 3.55 (dd, *J* = 4.9, 4.9 Hz, 1H), 3.31 (dd, *J* = 9.0, 2.4 Hz, 1H), 2.29 – 2.24 (m, 2H), 2.12 – 2.06 (m, 5H), 1.88 – 1.81 (m, 5H), 1.77 (ddd, *J* = 13.2, 10.7, 4.5 Hz, 1H), 1.69 (ddd, *J* = 13.5, 4.1, 2.0 Hz, 1H), 1.56 (s, 3H), 1.50 – 1.43 (m, 11H), 1.30 (s, 3H), 0.93 – 0.90 (m, 27H), 0.10 (s, 3H), 0.09 – 0.08 (m, 9H), 0.07 (s, 3H), 0.06 (s, 3H). ^{13}C NMR (151 MHz, CD_2Cl_2) δ 166.3, 155.2, 119.4, 99.9, 84.7, 81.7, 80.3, 79.6, 75.4, 74.9, 73.6, 73.1, 70.0, 69.7, 65.3, 60.0, 46.7, 41.8, 40.2, 38.4, 33.6, 29.1, 28.4, 26.4, 26.2, 26.1, 24.3, 19.1, 18.5, 18.4, 18.4, 3.7, –3.3, –4.0, –4.4, –4.5, –4.6, –4.7. IR (film) $\tilde{\nu}$ 2951, 2927, 2856, 1713, 1651, 1463, 1368, 1254, 1136, 1095, 836, 775 cm^{-1}. HRMS (ESI) *m/z* calcd. for $C_{46}H_{86}O_9Si_3Na$ $[M+Na]^+$: 889.54718, found: 889.54645.

Isopropylidene Acetal S12. Prepared analogously starting from the minor isomer **S10**; yellow oil (2.10 mg, quant.).

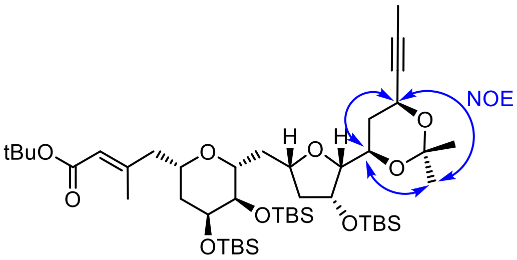

$[\alpha]_D^{20}$ = +43.8° (*c* = 0.21, CH_2Cl_2). ^1H NMR (600 MHz, CD_2Cl_2) δ 5.55 (q, *J* = 1.2 Hz, 1H), 4.62 – 4.57 (m, 1H), 4.35 (ddd, *J* = 6.4, 4.5, 3.0 Hz, 1H), 4.12 (ddd, *J* = 11.5, 6.4, 2.5 Hz, 1H), 4.02 (dt, *J* = 4.3, 2.1 Hz, 1H), 3.97 – 3.89 (m, 2H), 3.72 (ddd, *J* = 10.4, 8.9, 3.0 Hz, 1H), 3.36 (dd, *J* = 6.4, 4.5 Hz, 1H), 3.30 (dd, *J* = 9.0, 2.4 Hz, 1H), 2.30 – 2.23 (m, 2H), 2.10 – 2.05 (m, 4H), 1.87 (dt, *J* = 13.4, 2.6 Hz, 1H), 1.84 – 1.79 (m, 4H), 1.74 (ddd, *J* = 13.2, 10.4, 4.7 Hz, 1H), 1.71 – 1.63 (m, 2H), 1.50 – 1.44 (m, 11H), 1.42 (d, *J* = 0.6 Hz, 3H), 1.33 (d, *J* = 0.6 Hz, 3H), 0.91 (s, 9H), 0.91 (s, 9H), 0.89 (s, 9H), 0.09 (s, 3H), 0.08 (s, 3H), 0.07 (s, 3H), 0.06 (s, 3H), 0.05 (s, 3H), 0.05 (s, 3H). ^{13}C NMR (151 MHz, CD_2Cl_2) δ 166.3, 155.2, 119.3, 99.0, 85.3, 80.6, 79.6, 78.9, 75.5, 75.0, 73.6, 72.5, 70.1, 69.7, 67.1, 60.6, 46.7, 41.7, 40.2, 38.7, 35.6, 30.2, 28.4, 26.4, 26.2, 26.1, 20.0, 19.1, 18.5, 18.4, 18.4, 3.6, –3.3, –3.9, –4.5, –4.5, –4.5, –4.6, –4.7. IR (film) $\tilde{\nu}$ 2953, 2926, 2855, 1713, 1650, 1462, 1378, 1366, 1256, 1137, 1096, 836, 774 cm^{-1}. HRMS (ESI) *m/z* calcd. for $C_{46}H_{86}O_9Si_3Na$ $[M+Na]^+$: 889.54718, found: 889.54751.

Compound S13. An oven-dried flask was charged with 1,3-diol **26** (352.0 mg, 0.43 mmol) and CH_2Cl_2 (10 mL) and the resulting solution stirred at –78 °C (bath temperature) while 2,6-lutidine (0.25 mL, 2.13 mmol) was added, followed by slow addition of TBSOTf (0.24 mL, 1.06 mmol). Stirring was continued at –78 °C for 3 h before the reaction was quenched with sat. aq. $NaHCO_3$ solution (20 mL). The mixture was extracted with CH_2Cl_2 (3 x 20 mL), the combined organic layers were washed with brine (20 mL), dried over Na_2SO_4, and concentrated under reduced pressure. The residue was purified by flash chromatography (silica; hexane/EtOAc, 20:1 to 10:1) to give the title compound as a colorless oil (440.0 mg, 98%). $[\alpha]_D^{20}$ = +33.7° (*c* = 0.82, $CHCl_3$). ^1H NMR (400 MHz, $CDCl_3$) δ 5.57 (q, *J* = 1.3 Hz, 1H), 4.61 – 4.51 (m, 1H), 4.34 (ddd, *J* = 6.3, 4.6, 3.2 Hz, 1H), 4.21 (dt, *J* = 7.2, 5.2 Hz, 1H), 4.03 – 3.96 (m, 1H), 3.96 – 3.84 (m, 2H), 3.77 – 3.64 (m, 1H), 3.49 (t, *J* = 4.6 Hz, 1H), 3.28 (dd, *J* = 9.0, 2.4 Hz, 1H), 2.26 (ddd, *J* = 14.3, 8.2, 1.1 Hz, 1H), 2.21 – 2.13 (m, 1H), 2.11 (d, *J* = 1.3 Hz, 3H), 2.10 – 2.03 (m, 2H), 1.92 (ddd, *J* = 13.3, 7.2, 5.9 Hz, 1H), 1.85 – 1.80 (m, 2H), 1.78 (d, *J* = 2.2 Hz, 3H), 1.67 (ddd, *J* = 13.4, 4.1, 2.0 Hz, 1H), 1.54 – 1.45 (m, 11H), 0.90 (s, 9H), 0.89 (s, 9H), 0.89 (s, 9H), 0.89 (s, 9H), 0.86 (s, 9H), 0.12 (s, 3H), 0.09 (s, 3H), 0.08 – 0.05 (m, 18H), 0.05 (s, 3H), 0.04 (s, 3H). ^{13}C NMR (101 MHz, $CDCl_3$) δ 166.4, 155.0, 119.1, 86.6, 82.2, 79.6, 79.6, 74.9, 74.5, 73.4, 72.8, 69.8, 69.3, 68.2, 60.6, 46.5, 43.9, 40.6, 40.2, 37.9, 28.5, 26.4, 26.3, 26.3 (two peaks), 26.0, 19.1, 18.5, 18.4, 18.3, 18.3, 18.3, 3.8, –3.3, –3.3, –3.4, –3.8, –4.0, –4.1, –4.3, –4.4, –4.5, –4.5. IR (film) $\tilde{\nu}$ 2953, 2928, 2888, 2856, 1713, 1649, 1472, 1463, 1362, 1252, 1134, 1084, 960, 834, 773 cm^{-1}. HRMS (ESI) *m/z* calcd. for $C_{55}H_{110}O_9Si_5Na$ $[M+Na]^+$: 1077.68884, found: 1077.68879.

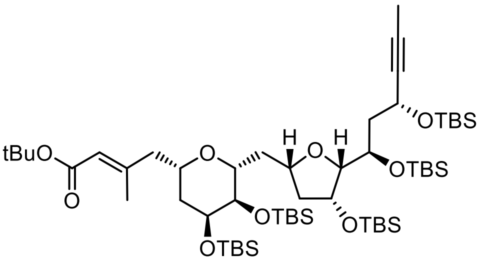

Compound 27. An oven-dried flask was charged with ester **S13** (440.0 mg, 0.42 mmol) and toluene (10.0 mL). This resulting solution was stirred at –78 °C (bath temperature) for 5 min before the slow addition of DIBAL-H (1.0 M in toluene, 0.63 mL, 0.63 mmol). After stirring at –78 °C for 1 h, the reaction was quenched with MeOH (1 mL), followed by sat. Rochelle salt solution (10 mL). The resulting mixture was stirred vigorously at room temperature for 1 h before the aqueous phase was extracted with EtOAc (3 x 20 mL). The combined organic layers were dried over Na_2SO_4 and concentrated under reduced pressure to give a mixture of alcohol and aldehyde that was used in the next step without further purification.

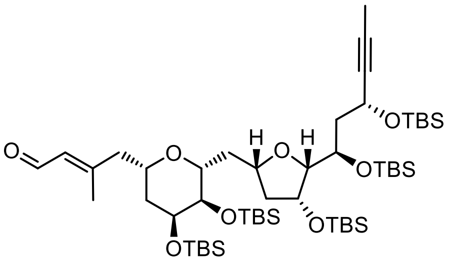

Dess-Martin periodinane (353.5 mg, 0.83 mmol) was added at 0 °C to a suspension of the crude product and $NaHCO_3$ (140.0 mg, 1.67 mmol) in CH_2Cl_2 (10 mL). The resulting mixture was stirred at room temperature for 2 h before sat. aq. $Na_2S_2O_3$ solution (10 mL) and H_2O (10 mL) were added. The organic

layer was separated and the aqueous phase extracted with CH_2Cl_2 (3 x 20 mL). The combined organic layers were washed with sat. aq. $NaHCO_3$ solution (3 x 20 mL), dried over Na_2SO_4, and concentrated under reduced pressure. The residue was purified by flash chromatography (silica; hexane/EtOAc, 20:1 to 10:1) to give the title compound as a colorless oil (322.1 mg, 79% over two steps). $[\alpha]_D^{20}$ = +36.6° (*c* = 0.61, $CHCl_3$). ^{1}H NMR (400 MHz, $CDCl_3$) δ 10.00 (d, *J* = 8.0 Hz, 1H), 5.87 (dq, *J* = 8.0, 1.2 Hz, 1H), 4.56 (ddd, *J* = 8.1, 5.4, 2.1 Hz, 1H), 4.36 (ddd, *J* = 6.5, 4.9, 3.7 Hz, 1H), 4.20 (dt, *J* = 7.6, 4.8 Hz, 1H), 4.04 – 3.91 (m, 2H), 3.91 – 3.81 (m, 1H), 3.72 (td, *J* = 9.8, 3.7 Hz, 1H), 3.53 (t, *J* = 4.6 Hz, 1H), 3.28 (dd, *J* = 9.0, 2.3 Hz, 1H), 2.38 – 2.27 (m, 1H), 2.23 – 2.11 (m, 5H), 2.07 (ddd, *J* = 13.3, 8.2, 4.9 Hz, 1H), 1.96 – 1.76 (m, 6H), 1.67 (ddd, *J* = 13.4, 4.1, 2.2 Hz, 1H), 1.57 – 1.45 (m, 2H), 0.90 (s, 9H), 0.90 (s, 9H), 0.89 (s, 9H), 0.89 (s, 9H), 0.87 (s, 9H), 0.12 (s, 3H), 0.09 (s, 3H), 0.09 – 0.06 (m, 15H), 0.06 (s, 3H), 0.05 (s, 3H), 0.04 (s, 3H). ^{13}C NMR (101 MHz, $CDCl_3$) δ 191.2, 161.2, 128.9, 86.3, 82.2, 79.7, 74.7, 74.4, 73.5, 72.9, 69.7, 69.6, 68.4, 60.6, 46.3, 43.6, 41.0, 40.3, 38.0, 26.4, 26.3, 26.3, 26.3, 26.0, 18.5, 18.4, 18.3, 18.3, 18.3, 18.3, 3.8, –3.2, –3.3, –3.4, –3.9, –4.0, –4.0, –4.3, –4.4, –4.4, –4.5. IR (film) $\tilde{\nu}$ 2955, 2929, 2889, 2857, 1678, 1472, 1463, 1361, 1254, 1087, 966, 836, 775 cm^{-1}. HRMS (ESI) *m/z* calcd. for $C_{51}H_{102}O_8Si_5Na$ $[M+Na]^+$: 1005.63132, found: 1005.63084.

Compound 28. To an oven-dried flask was charged with (+)-Ipc_2BOMe (*freshly prepared*;[6] 169.8 mg, 0.54 mmol) and Et_2O (4 mL). The solution was stirred at –78 °C (bath temperature) while allylmagnesium bromide (1.0 M in Et_2O, 0.44 mL, 0.44 mmol) was added dropwise. Stirring was continued at –78 °C for 15 min and at room temperature for another 1 h. The mixture was then cooled to at –95 °C (bath temperature) before a solution of aldehyde **27** (220.0 mg, 0.22 mmol) in Et_2O (4 mL) was added dropwise. After stirring at –95 °C for 2 h, water (4 mL) was added followed by $NaBO_3{\cdot}4H_2O$ (206.4 mg, 1.34 mmol). The mixture was vigorously stirred at room temperature for 2 h before it was diluted with *tert*-butyl methyl ether (10 mL). The aqueous phase was extracted with *tert*-butyl methyl ether (3 x 20 mL), and the combined organic layers were washed with brine (20 mL), dried over Na_2SO_4, and concentrated under reduced pressure. The borane-derived by-products were removed by Kugelrohr distillation (< 0.1 mbar, 70 °C) before the residue was submitted to flash chromatography (silica; hexane/EtOAc = 10:1) to give the desired product as a colorless oil (188.0 mg, 82%). $[\alpha]_D^{20}$ = +36.0° (*c* = 0.85, $CHCl_3$). ^{1}H NMR (400 MHz, $CDCl_3$) δ 5.87 – 5.72 (m, 1H), 5.22 (dq, *J* = 8.6, 1.2 Hz, 1H), 5.17 – 5.08 (m, 2H), 4.61 – 4.51 (m, 1H), 4.44 – 4.33 (m, 2H), 4.20 (dt, *J* = 7.6, 4.9 Hz, 1H), 4.02 – 3.96 (m, 1H), 3.94 – 3.83 (m, 2H), 3.74 – 3.65 (m, 1H), 3.51 (dd, *J* = 4.6, 4.6 Hz, 1H), 3.29 (dd, *J* = 9.0, 2.4 Hz, 1H), 2.33 – 2.23 (m, 2H), 2.21 – 2.11 (m, 2H), 2.06 (ddd, *J* = 13.4, 8.2, 5.1 Hz, 1H), 1.98 (ddd, *J* = 13.9, 5.1, 1.1 Hz, 1H), 1.92 (ddd, *J* = 13.6, 7.6, 5.5 Hz, 1H), 1.86

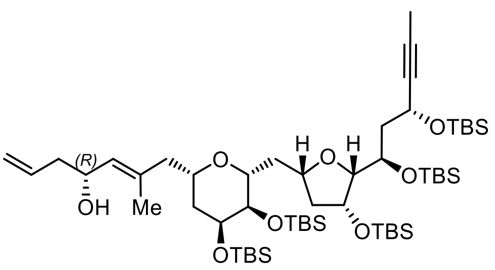

– 1.80 (m, 2H), 1.78 (d, J = 2.2 Hz, 3H), 1.69 – 1.64 (m, 4H), 1.53 – 1.41 (m, 3H), 0.90 (s, 9H), 0.90 (s, 9H), 0.89 (s, 18H), 0.86 (s, 9H), 0.12 (s, 3H), 0.09 (s, 3H), 0.08 – 0.05 (m, 18H), 0.05 (s, 3H), 0.04 (s, 3H). ^{13}C NMR (101 MHz, $CDCl_3$) δ 135.9, 134.5, 129.3, 118.2, 86.3, 82.2, 79.6, 74.9, 74.5, 73.4, 72.9, 69.8, 69.4, 68.4, 67.8, 60.6, 45.6, 43.6, 42.3, 41.1, 40.2, 37.9, 26.4, 26.3, 26.3, 26.2, 26.0, 18.5, 18.4, 18.3, 18.3, 18.3, 16.9, 3.8, –3.3, –3.3, –3.4, –3.9 (two peaks), –4.0, –4.3, –4.4 (two peaks), –4.5. IR (film) ṽ 3441, 2953, 2928, 2888, 2856, 1670, 1472, 1361, 1253, 1128, 1085, 962, 835, 774 cm^{-1}. HRMS (ESI) *m/z* calcd. for $C_{54}H_{108}O_8Si_5Na$ [M+Na]$^+$: 1047.67827, found: 1047.67929.

(*S*)-Mosher ester derived from compound 28. Reaction of **28** with (*R*)-(–)-MTPA-Cl under standard conditions (CH_2Cl_2, pyridine, 0°C to RT) furnished the corresponding (*S*)-(+)-MTPA ester as a colorless oil (1.30 mg, quant.). $[\alpha]_D^{20}$ = +10.0° (*c* = 0.13, $CHCl_3$). ^{1}H NMR (600 MHz, $CDCl_3$) δ 7.51 – 7.48 (m, 2H), 7.40 – 7.36 (m, 3H), 5.75 – 5.68 (m, 2H), 5.14 – 5.07 (m, 3H), 4.56 (ddd, J = 7.9, 5.5, 2.0 Hz, 1H), 4.35 (dt, J = 6.5, 4.8 Hz, 1H), 4.20 (dt, J = 7.6, 4.9 Hz, 1H), 4.00 – 3.96 (m, 1H), 3.91 – 3.84 (m, 2H), 3.70 (td, J = 10.1, 3.2 Hz, 1H), 3.52 – 3.51 (m, 3H), 3.50 (dd, J = 4.6, 4.6 Hz, 1H), 3.27 (dd, J = 9.0, 2.3 Hz, 1H), 2.50 – 2.44 (m, 1H), 2.38 – 2.33 (m, 1H), 2.20 – 2.13 (m, 2H), 2.06 (ddd, J = 13.2, 8.1, 5.0 Hz, 1H), 1.98 (dd, J = 14.1, 4.9 Hz, 1H), 1.95 – 1.90 (m, 1H), 1.84 – 1.79 (m, 2H), 1.78 (d, J = 2.1 Hz, 3H), 1.76 (d, J = 1.4 Hz, 3H), 1.64 (ddd, J = 13.5, 4.2, 2.0 Hz, 1H), 1.49 (ddd, J = 12.9, 7.9, 3.7 Hz, 1H), 1.42 (ddd, J = 13.3, 11.4, 1.9 Hz, 1H), 0.90 (s, 9H), 0.90 (s, 9H), 0.89 (s, 9H), 0.89 (s, 9H), 0.86 (s, 9H), 0.12 (s, 3H), 0.09 (s, 3H), 0.08 (s, 3H), 0.07 (s, 6H), 0.06 (s, 6H), 0.05 (s, 3H), 0.05 (s, 3H), 0.04 (s, 3H). ^{13}C NMR (151 MHz, $CDCl_3$) δ 165.9, 139.4, 133.0, 132.5, 129.6, 128.4, 127.7, 124.0, 118.6, 86.4, 82.2, 79.7, 74.9, 74.5, 73.8, 73.3, 72.9, 69.8, 69.3, 68.3, 60.6, 55.6, 45.8, 43.7, 41.1, 40.1, 39.2, 38.0, 26.4, 26.3, 26.3, 26.3, 26.0, 18.5, 18.4, 18.3, 18.3 (two peaks), 17.0, 3.8, –3.3, –3.3, –3.4, –3.9, –4.0, –4.0, –4.3, –4.4, –4.5, –4.5. ***Note**: the ^{13}C signals of the CF_3 group and the adjacent quaternary carbon have not been detected.* IR (film) ṽ 2953, 2925, 2855, 1748, 1659, 1632, 1464, 1253, 1127, 1085, 964, 836, 776 cm^{-1}. HRMS (ESI) *m/z* calcd. for $C_{64}H_{115}F_3O_{10}Si_5Na$ [M+Na]$^+$: 1263.71809, found: 1263.71943.

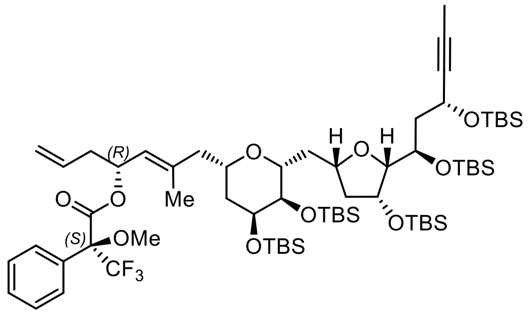

(*R*)-Mosher ester derived from compound 28. The analogous reaction of **28** with (*S*)-(+)-MTPA-Cl gave the corresponding (*R*)-(+)-MTPA ester as a colorless oil (1.42 mg, quant.). $[\alpha]_D^{20}$ = +22.1° (*c* = 0.14, $CHCl_3$). ^{1}H NMR (600 MHz, $CDCl_3$) δ 7.54 – 7.51 (m, 2H), 7.39 – 7.36 (m, 3H), 5.74 (ddd, J = 9.4, 7.4, 5.8 Hz, 1H), 5.61 (dddd, J = 16.9, 10.3, 7.4, 6.5 Hz, 1H), 5.25 – 5.18 (m, 1H), 5.05 – 4.99 (m, 2H), 4.56 (ddd, J = 8.0, 5.4, 2.1 Hz, 1H), 4.34

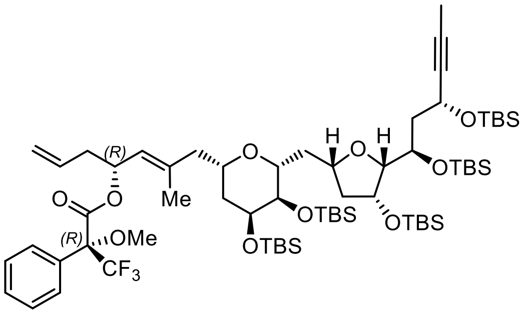

(ddd, *J* = 6.6, 4.8, 3.5 Hz, 1H), 4.20 (dt, *J* = 7.6, 4.9 Hz, 1H), 3.96 – 3.93 (m, 1H), 3.92 – 3.84 (m, 2H), 3.72 – 3.67 (m, 1H), 3.54 – 3.53 (m, 3H), 3.50 (dd, *J* = 4.6, 4.6 Hz, 1H), 3.27 (dd, *J* = 9.0, 2.3 Hz, 1H), 2.44 – 2.38 (m, 1H), 2.33 – 2.28 (m, 1H), 2.25 – 2.20 (m, 1H), 2.17 – 2.12 (m, 1H), 2.06 (ddd, *J* = 13.3, 8.1, 5.0 Hz, 1H), 2.02 – 1.98 (m, 1H), 1.92 (ddd, *J* = 13.7, 7.6, 5.5 Hz, 1H), 1.86 – 1.79 (m, 2H), 1.78 (d, *J* = 2.1 Hz, 3H), 1.77 (d, *J* = 1.4 Hz, 3H), 1.60 (ddd, *J* = 13.4, 4.0, 2.0 Hz, 1H), 1.49 (ddd, *J* = 13.0, 7.7, 3.8 Hz, 1H), 1.40 (ddd, *J* = 13.4, 11.4, 2.0 Hz, 1H), 0.90 (s, 9H), 0.89 (s, 9H), 0.89 (s, 9H), 0.89 (s, 9H), 0.86 (s, 9H), 0.12 (s, 3H), 0.10 (s, 3H), 0.07 (s, 6H), 0.07 (s, 3H), 0.06 (s, 3H), 0.05 (s, 3H), 0.05 (s, 3H), 0.04 (s, 3H), 0.00 (s, 3H). ^{13}C NMR (151 MHz, $CDCl_3$) δ 165.8, 139.7, 132.7, 132.7, 129.6, 128.4, 127.5, 124.0, 118.5, 86.4, 82.2, 79.7, 74.9, 74.5, 73.5, 73.3, 72.9, 69.8, 69.3, 68.3, 60.6, 55.5, 45.8, 43.7, 41.0, 40.1, 39.2, 38.0, 26.4, 26.3, 26.3, 26.2, 26.0, 18.5, 18.4, 18.3 (three peaks), 17.1, 3.8, –3.3, –3.3, –3.4, –3.9, –4.0 (two peaks), –4.3, –4.4, –4.5, –4.5. ***Note****: the ^{13}C signals of the CF_3 group and the adjacent quaternary carbon have not been detected.* IR (film) ṽ 2954, 2925, 2854, 1746, 1658, 1632, 1462, 1254, 1126, 1096, 957, 836, 775 cm^{-1}. HRMS (ESI) *m/z* calcd. for $C_{64}H_{115}F_3O_{10}Si_5Na$ $[M+Na]^+$: 1263.71809, found: 1263.71893.

Table S3. Determination of absolute configuration of the C62 stereogenic center of compound **28** via Mosher ester analysis.[2]

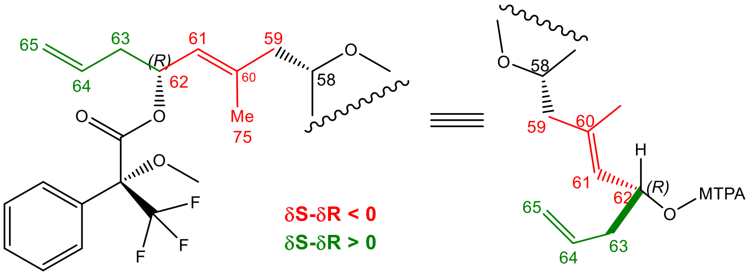

No.	$\delta_{H\ NMR}$ (*S*-ester) (ppm)	$\delta_{H\ NMR}$ (*R*-ester) (ppm)	*Δδ* (δ_S -δ_R, ppm)
75	1.76	1.77	–0.01
59'	2.18	2.23	–0.05
59''	1.98	2.00	–0.02
61	5.10	5.23	–0.13
62	5.72	5.74	–0.02
63'	2.47	2.41	0.06
63''	2.35	2.3	0.05
64	5.72	5.61	0.11
65-*trans*	5.11	5.02	0.09
65-*cis*	5.09	5.01	0.08

Compound 29. An oven-dried Schlenk tube was charged with compound **28** (173.1 mg, 168.7 µmol), allylic alcohol **7** (211.0 mg, 506.2 µmol) and degassed CH_2Cl_2 (18 mL). The mixture was stirred at room temperature before a solution of catalyst **31** (5.3 mg, 8.4 µmol) in degassed CH_2Cl_2 (6 mL) was added. The resulting mixture was stirred at room temperature under a gentle flow of Ar for 24 h. Celite was then added and the suspension stirred at room temperature for 10 min. The mixture was filtered through a plug of silica, which was carefully rinsed with *tert*-butyl methyl ether. The combined filtrates were concentrated under reduced pressure and the residue purified by flash chromatography (silica; hexane/EtOAc, 10:1 to 5:1) to give the title compound as a yellow oil (174.2 mg, 73%). $[\alpha]_D^{20}$ = +47.1° (*c* = 0.65, $CHCl_3$). ^1H NMR (400 MHz, $CDCl_3$) δ 5.67 (dt, *J* = 15.7, 7.0 Hz, 1H), 5.58 (dd, *J* = 15.5, 5.8 Hz, 1H), 5.23 – 5.13 (m, 1H), 4.59 – 4.50 (m, 1H), 4.40 – 4.28 (m, 3H), 4.20 (dt, *J* = 7.6, 4.7 Hz, 1H), 4.01 – 3.96 (m, 2H), 3.95 – 3.80 (m, 5H), 3.70 (td, *J* = 9.1, 4.8 Hz, 1H), 3.67 – 3.61 (m, 1H), 3.53 (dd, *J* = 4.6, 4.6 Hz, 1H), 3.30 – 3.24 (m, 2H), 2.26 – 2.13 (m, 4H), 2.07 (ddd, *J* = 13.4, 8.3, 4.9 Hz, 1H), 1.99 – 1.88 (m, 3H), 1.85 – 1.75 (m, 6H), 1.71 – 1.59 (m, 6H), 1.49 (ddd, *J* = 12.3, 8.0, 3.9 Hz, 1H), 1.45 – 1.35 (m, 2H), 0.91 (s, 9H), 0.90 – 0.87 (m, 45H), 0.86 (s, 9H), 0.11 (s, 3H), 0.09 (s, 3H), 0.08 – 0.04 (m, 33H), 0.02 (s, 3H). ^{13}C NMR (101 MHz, $CDCl_3$) δ 136.6, 135.7, 129.4, 126.1, 86.2, 82.2, 79.6, 77.0, 74.9, 74.5, 74.3, 73.3, 73.0, 72.9, 69.8, 69.8, 68.9, 68.5, 67.9, 61.8, 60.6, 45.6, 43.5, 41.1, 40.8, 40.0, 39.3, 37.9, 34.1, 26.4, 26.3, 26.3, 26.3, 26.2, 26.0 (two peaks), 18.5, 18.4, 18.3, 18.3, 18.3, 18.3, 18.2, 17.2, 3.8, –3.3, –3.3, –3.4, –3.4, –3.9, –4.0, –4.0, –4.1, –4.3, –4.4, –4.5, –4.5, –4.5 (two peaks). IR (film) $\tilde{\nu}$ 3424, 2953, 2928, 2893, 2856, 1669, 1472, 1361, 1254, 1085, 961, 835, 774 cm^{-1}. HRMS (ESI) *m/z* calcd. for $C_{73}H_{148}O_{12}Si_7Na$ $[M+Na]^+$: 1435.92479, found: 1435.92392.

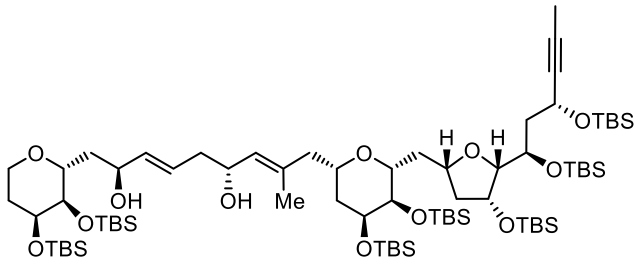

Compound S14. A solution of compound **29** (174.2 mg, 123.1 µmol) in CH_2Cl_2 (5 mL) was stirred at –78 °C (bath temperature) while 2,6-lutidine (72.0 µL, 615.7 µmol) was added, followed by slow addition of TBSOTf (71.0 µL, 308.0 µmol). Stirring was continued at this temperature for 2 h before sat. aq. $NaHCO_3$ solution (10 mL) was introduced. The aqueous phase was extracted with CH_2Cl_2 (3 x 10 mL), the combined organic layers were washed with brine (10 mL), dried over Na_2SO_4, and concentrated under reduced pressure. The residue was purified by flash chromatography (silica; hexane/EtOAc = 20:1) to give the title compound as a colorless oil (193.0 mg, 95%). $[\alpha]_D^{20}$ = +28.1° (*c* = 0.69, $CHCl_3$). ^1H NMR (400 MHz, $CDCl_3$) δ 5.61 – 5.49 (m, 1H), 5.42 (dd, *J* = 15.5, 6.8 Hz, 1H), 5.14 (d, *J* = 8.6 Hz, 1H), 4.63 – 4.52 (m, 1H), 4.41 – 4.34 (m,

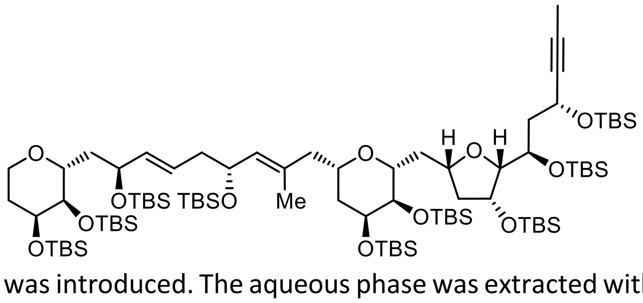

1H), 4.31 (ddd, J = 8.6, 7.2, 5.6 Hz, 1H), 4.27 – 4.18 (m, 2H), 4.02 – 3.96 (m, 2H), 3.94 – 3.80 (m, 2H), 3.75 – 3.65 (m, 3H), 3.59 – 3.52 (m, 2H), 3.31 – 3.24 (m, 2H), 2.29 – 2.14 (m, 3H), 2.12 – 2.03 (m, 2H), 1.99 – 1.89 (m, 2H), 1.87 – 1.81 (m, 2H), 1.80 – 1.74 (m, 5H), 1.70 – 1.58 (m, 5H), 1.52 – 1.38 (m, 3H), 0.90 – 0.87 (m, 81H), 0.12 (s, 3H), 0.10 (s, 3H), 0.09 – 0.03 (m, 42H), 0.01 (s, 3H), 0.00 (s, 3H). ^{13}C NMR (101 MHz, $CDCl_3$) δ 135.1, 132.6, 131.5, 126.8, 86.3, 82.2, 79.6, 74.8, 74.7, 74.6, 73.2, 73.0, 72.9, 71.4, 71.1, 70.1, 69.8, 69.5, 68.5, 61.7, 60.6, 45.9, 43.6, 41.9, 41.4, 41.1, 40.2, 38.0, 34.6, 26.4, 26.3 (two peaks), 26.3, 26.3, 26.1 (two peaks), 26.1, 26.0, 18.5, 18.5, 18.3, 18.3 (five peaks), 18.3, 17.4, 3.8, –3.2, –3.3, –3.3, –3.4, –3.8, –3.8, –3.9, –4.0, –4.0, –4.1, –4.3, –4.4, –4.4, –4.5, –4.5 (two peaks), –4.5 (two peaks). IR (film) $\tilde{\nu}$ 2954, 2928, 2887, 2857, 1472, 1463, 1361, 1253, 1084, 967, 835, 774 cm^{-1}. HRMS (ESI) m/z calcd. for $C_{85}H_{176}O_{12}Si_9Na$ [M+Na]$^+$: 1664.09774, found: 1664.09776.

Compound 30. An oven-dried Schlenk tube was charged with alkyne **S14** (100.0 mg, 60.9 µmol), [(P(o-tolyl)$_3$)$_2$PdCl$_2$] (4.8 mg, 6.1 µmol) and THF (2 mL). The mixture was stirred at room temperature while a solution of nBu$_3$SnH (81.0 µL, 304.3 µmol) in THF (2 mL) was added dropwise via syringe pump over the course of 2 h. Once the addition was complete, stirring was continued for an additional 10 min before the mixture was filtered through a pad of Celite. The filtrate was evaporated under reduced pressure and the residue purified by flash chromatography (silica; hexane/EtOAc, 200:1 to 50:1) to afford the targeted alkenylstannane as a colorless oil (97.3 mg, 83%). The obtained compound was immediately used in the next reaction because of its instability.

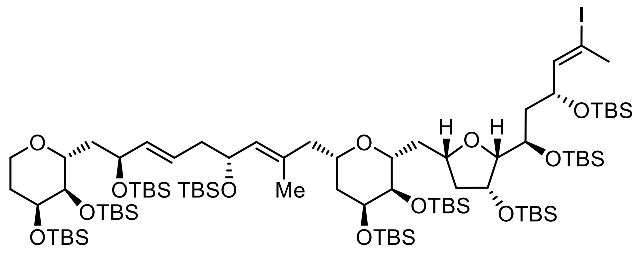

A solution of iodine (0.02 M in CH_2Cl_2, prepared by dissolving 25.4 mg of I_2 in 5 mL of CH_2Cl_2) was added dropwise to a solution of the alkenylstannane in CH_2Cl_2 (5 mL) at 0 °C. Once the purple color persisted, the addition was stopped immediately and stirring was continued at 0 °C in the dark for an additional 10 min. The reaction was quenched with sat. aq. $Na_2S_2O_3$ solution (10 mL), the aqueous phase was extracted with CH_2Cl_2 (3 x 10 mL), and the combined organic layers were washed with brine (10 mL), dried over Na_2SO_4 and concentrated under reduced pressure. The residue was purified by flash chromatography (silica; hexane/EtOAc, 100:1 to 50:1) to give the title compound as a colorless oil (88.8 mg, 82%). $[\alpha]_D^{20}$ = +31.6° (c = 0.70, CH_2Cl_2). ^{1}H NMR (400 MHz, C_6D_6) δ 6.46 (dq, J = 9.3, 1.5 Hz, 1H), 6.02 – 5.90 (m, 1H), 5.81 (dd, J = 15.5, 6.6 Hz, 1H), 5.41 (d, J = 8.5 Hz, 1H), 4.94 (ddd, J = 9.3, 7.8, 5.6 Hz, 1H), 4.70 (ddd, J = 10.0, 6.5, 4.0 Hz, 1H), 4.56 (ddd, J = 8.6, 7.2, 5.5 Hz, 1H), 4.45 – 4.35 (m, 2H), 4.31 – 4.21 (m, 1H), 4.14 – 4.06 (m, 1H), 4.03 – 3.94 (m, 3H), 3.91 (dt, J = 4.3, 2.2 Hz, 1H), 3.88 – 3.79 (m, 1H), 3.69 (dd, J = 4.7, 4.7 Hz, 1H), 3.63 – 3.56 (m, 1H), 3.38 – 3.30 (m, 2H), 2.60 – 2.47 (m, 4H), 2.45 – 2.29 (m, 3H), 2.25 (ddd, J = 13.4, 7.9, 5.3 Hz, 1H), 2.20 – 2.02 (m, 5H), 1.91 (ddd, J = 13.3, 10.7, 4.3 Hz, 1H), 1.78

(d, J = 1.4 Hz, 3H), 1.75 – 1.64 (m, 2H), 1.64 – 1.53 (m, 1H), 1.45 – 1.33 (m, 2H), 1.09 (s, 9H), 1.08 – 1.06 (m, 18H), 1.06 – 1.02 (m, 45H), 1.00 (s, 9H), 0.28 (s, 3H), 0.27 (s, 3H), 0.25 – 0.23 (m, 6H), 0.23 – 0.19 (m, 12H), 0.19 – 0.17 (m, 6H), 0.15 (s, 3H), 0.14 (s, 3H), 0.13 (s, 3H), 0.11 (s, 3H), 0.10 (s, 3H), 0.06 (s, 3H), 0.05 – 0.02 (m, 6H). ^{13}C NMR (101 MHz, C_6D_6) δ 146.3, 135.7, 132.8, 132.1, 127.1, 95.1, 87.1, 75.2, 75.2, 75.1, 73.5, 73.4, 73.0, 71.7, 71.1, 70.6, 70.2, 69.9, 68.5, 68.4, 61.7, 46.3, 43.1, 42.4, 42.2, 42.1, 40.4, 38.7, 34.8, 28.8, 26.6 (two peaks), 26.5, 26.5, 26.4, 26.3, 26.3, 26.3, 26.3, 18.7, 18.6, 18.6 (four peaks), 18.5, 18.5, 18.4, 17.7, –2.7, –3.0, –3.2, –3.3, –3.5, –3.7, –3.8, –3.9, –3.9, –4.0, –4.1, –4.1, –4.2, –4.3, –4.3, –4.4, –4.4, –4.5. IR (film) ṽ 2953, 2928, 2893, 2856, 1733, 1639, 1472, 1463, 1361, 1253, 1080, 968, 834, 774 cm^{-1}. HRMS (ESI) *m/z* calcd. for $C_{85}H_{177}IO_{12}Si_9Na$ [M+Na]$^+$: 1792.01004, found: 1792.01036.

Final Substructure Verification

Compound 32. TBAF (1.0 M in THF, 85.0 µL, 85.0 µmol) was added dropwise to a solution of compound **29** (6.0 mg, 4.24 µmol) in THF (1.0 mL). The resulting mixture was stirred at room temperature for 12 h before $CaCO_3$ (30 mg), DOWEX 50WX8-400 (90 mg), and MeOH (1.0 mL) were added. The suspension was stirred at room temperature for 1 h, all insoluble materials were filtered off through a pad of Celite, thoroughly rinsing with MeOH. The combined filtrates were concentrated under reduced pressure and the residue was purified by flash chromatography (silica; CH_2Cl_2/MeOH = 4:1 to 2:1) to give a slightly impure product that was submitted to HPLC separation (YMC-Triart C18, MeOH/H_2O = 2:3) to provide the desired product as a colorless oil (1.50 mg, 58%). $[\alpha]_D^{20}$ = +18.0° (c = 0.15, MeOH). ^{1}H NMR (600 MHz, [D$_4$]-MeOH) δ 5.69 – 5.62 (m, 1H), 5.51 (ddt, J = 15.3, 7.3, 1.2 Hz, 1H), 5.22 (dq, J = 8.8, 1.2 Hz, 1H), 4.57 – 4.52 (m, 1H), 4.46 – 4.42 (m, 1H), 4.37 (dt, J = 8.7, 6.5 Hz, 1H), 4.28 – 4.23 (m, 1H), 4.16 – 4.08 (m, 2H), 4.03 – 3.98 (m, 2H), 3.90 – 3.84 (m, 1H), 3.70 (td, J = 11.8, 2.3 Hz, 1H), 3.64 – 3.55 (m, 3H), 3.41 (dd, J = 7.4, 4.3 Hz, 1H), 3.28 (dd, J = 9.7, 3.1 Hz, 1H), 3.23 (dd, J = 9.4, 3.1 Hz, 1H), 2.37 (ddd, J = 13.3, 7.6, 6.6 Hz, 1H), 2.30 – 2.24 (m, 1H), 2.22 – 2.16 (m, 2H), 2.06 – 1.94 (m, 4H), 1.91 – 1.72 (m, 8H), 1.71 (d, J = 1.4 Hz, 3H), 1.66 – 1.60 (m, 2H), 1.52 (ddd, J = 14.0, 11.5, 2.7 Hz, 1H). ^{13}C NMR (151 MHz, [D$_4$]-MeOH) δ 136.2, 135.8, 130.9, 129.1, 85.9, 81.9, 80.6, 76.5, 75.0, 74.3, 73.4, 73.3, 73.1, 71.8, 70.9, 69.1, 68.8, 68.1, 67.9, 62.6, 59.7, 46.5, 43.8, 41.9, 41.7, 40.9, 39.6, 39.5, 33.8, 17.4, 3.1. IR (film) ṽ 3354, 2923, 2851, 1658, 1633, 1450, 1411, 1105, 1016 cm^{-1}. HRMS (ESI) *m/z* calcd. for $C_{31}H_{50}O_{12}Na$ [M+Na]$^+$: 637.31945, found: 637.31965.

Table S4. Analysis of the NMR data of the polyol fragment **32**; benthol A numbering scheme as shown in the Insert

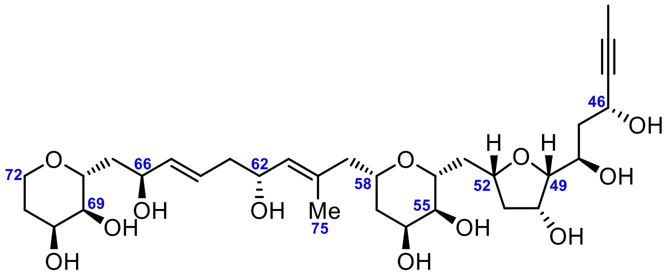

Atom	*δ* (ppm)	*J* (Hz)	COSY	HSQC	HMBC	NOESY
**43 C**	3.13			43		
**H3**	1.81	2.10(46)	46	43	44, 45, 46, 47	
**44 C**	80.55				43	
**45 C**	81.91				43, 46, 47a, 47b	
**46 C**	59.7			46	43, 47a, 47b, 48	
**H**	4.55	2.10(43), 3.40(47a), 9.40(47b)	43, 47a, 47b	46	45	47a, 47b, 48, 49'
**47 C**	43.84			47a, 47b	43, 48, 49'	
**Ha**	1.78	3.40(46), 14.30(47b), 9.80(48)	46, 47b, 48	47	45, 46, 48, 49	46, 49'
**Hb**	2.03	14.30(47a), 9.40(46), 2.90(48)	46, 47a, 48	47	45, 46	46, 49'
**48 C**	67.85			48	47a, 49'	
**H**	4.14	7.50(49'), 9.80(47a), 2.90(47b)	47a, 47b, 49'	48	46, 47, 49, 50	46, 49'
**49 C**	85.88			49'	47a, 48, 50, 51b	
**49' H**	3.41	4.30(50), 7.50(48)	48, 50	49	47, 48, 50	46, 47a, 47b, 48, 50, 51b, 52'
**50 C**	73.37			50	48, 49', 51a, 51b	
**H**	4.44	4.30(49'), 3.10(51a), 6.60(51b)	49', 51a, 51b	50	49, 52	49', 51b, 52'
**51 C**	41.72			51a, 51b	53a, 53b	
**Ha**	1.64	3.10(50), 7.10(52'), 13.30(51b)	50, 51b, 52'	51	50, 52, 53	53b, 54
**Hb**	2.37	13.30(51a), 6.60(50), 7.50(52')	50, 51a, 52'	51	49, 50, 52, 53	49', 50, 52'
**52 C**	76.5			52'	50, 51a, 51b, 53a, 53b, 54	
**52' H**	4.11	7.10(51a), 7.50(51b), 5.10(53a), 8.40(53b)	51a, 51b, 53a, 53b	52	54	49', 50, 51b, 53a, 54, 55
**53 C**	39.51			53a, 53b	51a, 51b, 54, 55	
**Ha**	1.88	9.00(54), 5.10(52'), 13.80(53b)	52', 53b, 54	53	51, 52, 54, 55	52', 55
**Hb**	1.99	13.80(53a), 2.80(54), 8.40(52')	52', 53a, 54	53	51, 52, 54, 55	51a, 54, 55
**54 C**	74.28			54	52', 53a, 53b, 55, 56	
**H**	3.6	9.70(55), 9.00(53a), 2.80(53b)	53a, 53b, 55	54	52, 53, 55, 56, 58	51a, 52', 53b, 58

**55 C**	73.1			55	53a, 53b, 54, 56, 57b	
**H**	3.28	9.70(54), 3.10(56)	54, 56	55	53, 54, 56, 57	52', 53a, 53b, 56, 57a
**56 C**	68.81			56	54, 55, 57b	
**H**	4.01	3.10(55)	55, 57a, 57b	56	54, 55, 58	55, 57a, 57b
**57 C**	39.59			57a, 57b	55, 59a, 59b	
**Ha**	1.52	14.10(57b),	56, 57b, 58	57	58, 59	55, 56, 59b
**Hb**	1.82	14.10(57a)	56, 57a, 58	57	55, 56	56, 58, 59a
**58 C**	70.92			58	54, 56, 57a, 59a, 59b	
**H**	3.87		57a, 57b, 59a, 59b	58		54, 57b, 59a, 59b, 61, 75
**59 C**	46.52			59a, 59b	57a, 61, 75	
**Ha**	2.04	13.90(59b)	58, 59b, 61	59	57, 58, 60, 61, 75	57b, 58, 61, 75
**Hb**	2.18	13.90(59a)	58, 59a, 61	59	57, 58, 60, 61, 75	57a, 58, 61, 75
**60 C**	135.83				59a, 59b, 62, 75	
**61 C**	130.88			61	59a, 59b, 62, 63a, 63b, 75	
**H**	5.22	1.38(75), 8.50(62)	59a, 59b, 62, 75	61	59, 63, 75	58, 59a, 59b, 63a, 63b
**62 C**	69.11			62	63a, 63b, 64	
**H**	4.37	6.50(63a), 6.50(63b), 8.50(61)	61, 63a, 63b	62	60, 61, 63, 64	64, 75
**63 C**	41.87			63a, 63b	61, 62, 64, 65	
**Ha**	2.2	6.10(64), 13.90(63b), 6.50(62)	62, 63b, 64, 65	63	61, 62, 64, 65	61, 65
**Hb**	2.27	7.50(64), 13.90(63a), 6.50(62)	62, 63a, 64, 65	63	61, 62, 64, 65	61, 65
**64 C**	129.09			64	62, 63a, 63b, 65, 66	
**H**	5.66	15.20(65), 6.10(63a), 7.50(63b)	63a, 63b, 65, 66	64	62, 63, 65, 66	62, 66
**65 C**	136.21			65	63a, 63b, 64, 66, 67a, 67b	
**H**	5.51	15.20(64), 7.30(66)	63a, 63b, 64, 66	65	63, 64, 66, 67	63a, 63b, 66, 67b, 68
**66 C**	71.77			66	64, 65, 67a, 67b, 68	
**H**	4.25	7.30(65), 5.70(67a), 8.10(67b)	64, 65, 67a, 67b	66	64, 65, 67, 68	64, 65, 67a, 68
**67 C**	40.91			67a, 67b	65, 66, 68, 69	
**Ha**	1.63	5.70(66), 9.80(68), 13.90(67b)	66, 67b, 68	67	65, 66, 68, 69	66, 69
**Hb**	1.96	8.10(66), 13.90(67a), 2.60(68)	66, 67a, 68	67	65, 66, 68, 69	65, 68, 69
**68 C**	74.98			68	66, 67a, 67b, 69, 70, 72a, 72b	
**H**	3.57	9.50(69), 9.80(67a), 2.60(67b)	67a, 67b, 69	68	66, 67, 69	65, 66, 67b, 72b
**69 C**	73.29			69	67a, 67b, 68, 70, 71a	
**H**	3.23	9.50(68), 3.10(70)	68, 70	69	67, 68, 70	67a, 67b, 70, 71b
**70 C**	68.08			70	69, 72a, 72b	
**H**	4	3.10(69)	69, 71a, 71b	70	68, 69, 72	69, 71a, 71b
**71 C**	33.79			71a, 71b	72a	
**Ha**	1.75		70, 71b, 72a, 72b	71	69	70, 72a, 72b

**Hb**	1.84		70, 71a, 72a, 72b	71	72	69, 70, 72a
**72 C**	62.58			72a, 72b	70, 71b	
**Ha**	3.61		71a, 71b, 72b	72	68, 70, 71	71a, 71b
**Hb**	3.69		71a, 71b, 72a	72	68, 70	68, 71a
**75 C**	17.4			75	59a, 59b, 61	
**H3**	1.71	1.38(61)	61	75	59, 60, 61	58, 59a, 59b, 62

Table S5. Comparison of pertinent NMR data of authentic benthol A[7] and those of the unprotected polyol fragment **32**.

Atom	δ (ppm, ^{1}H-NMR in CD_3OD)		Δδ	δ (ppm, ^{13}C-NMR in CD_3OD)		Δδ
	benthol A	**32**		benthol A	**32**	
**46**	4.67	4.55	0.12	66.2	59.7	6.5
**47a**	1.58	1.78	-0.20	43.3	43.84	-0.54
**47b**	1.88	2.03	-0.15			
**48**	4.10	4.14	-0.04	68.2	67.85	0.35
**49**	3.42	3.41	0.01	86.1	85.88	0.22
**50**	4.45	4.44	0.01	73.49	73.37	0.12
**51a**	1.64	1.64	0.00	41.73	41.72	0.01
**51b**	2.36	2.37	-0.01			
**52**	4.11	4.11	0.00	76.5	76.5	0.00
**53a**	1.88	1.88	0.00	39.61	39.51	0.10
**53b**	1.99	1.99	0.00			
**54**	3.59	3.60	-0.01	74.3	74.28	0.02
**55**	3.27	3.28	-0.01	73.15	73.1	0.05
**56**	4.01	4.01	0.00	68.9	68.81	0.09
**57a**	1.51	1.52	-0.01	39.63	39.59	0.04
**57b**	1.82	1.82	0.00			
**58**	3.86	3.87	-0.01	70.96	70.92	0.04
**59a**	2.04	2.04	0.00	46.6	46.52	0.08
**59b**	2.18	2.18	0.00			
**60**	-	-	-	135.9	135.83	0.07
**61**	5.21	5.22	-0.01	130.9	130.88	0.02
**62**	4.37	4.37	0.00	69.2	69.11	0.09
**63a**	2.20	2.20	0.00	41.9	41.87	0.03
**63b**	2.26	2.27	-0.01			
**64**	5.65	5.66	-0.01	129.1	129.09	0.01
**65**	5.50	5.51	-0.01	136.3	136.21	0.09
**66**	4.25	4.25	0.00	71.8	71.77	0.03
**67a**	1.63	1.63	0.00	41.0	40.91	0.09
**67b**	1.96	1.96	0.00			
**68**	3.56	3.57	-0.01	75.0	74.98	0.02
**69**	3.22	3.23	-0.01	73.33	73.29	0.04
**70**	4.00	4.00	0.00	68.1	68.08	0.02
**71a**	1.74	1.75	-0.01	33.8	33.79	0.01
**71b**	1.83	1.84	-0.01			
**72a**	3.61	3.61	0.00	62.6	62.58	0.02
**72b**	3.69	3.69	0.00			
**75**	1.70	1.74	-0.04	17.5	17.4	0.10

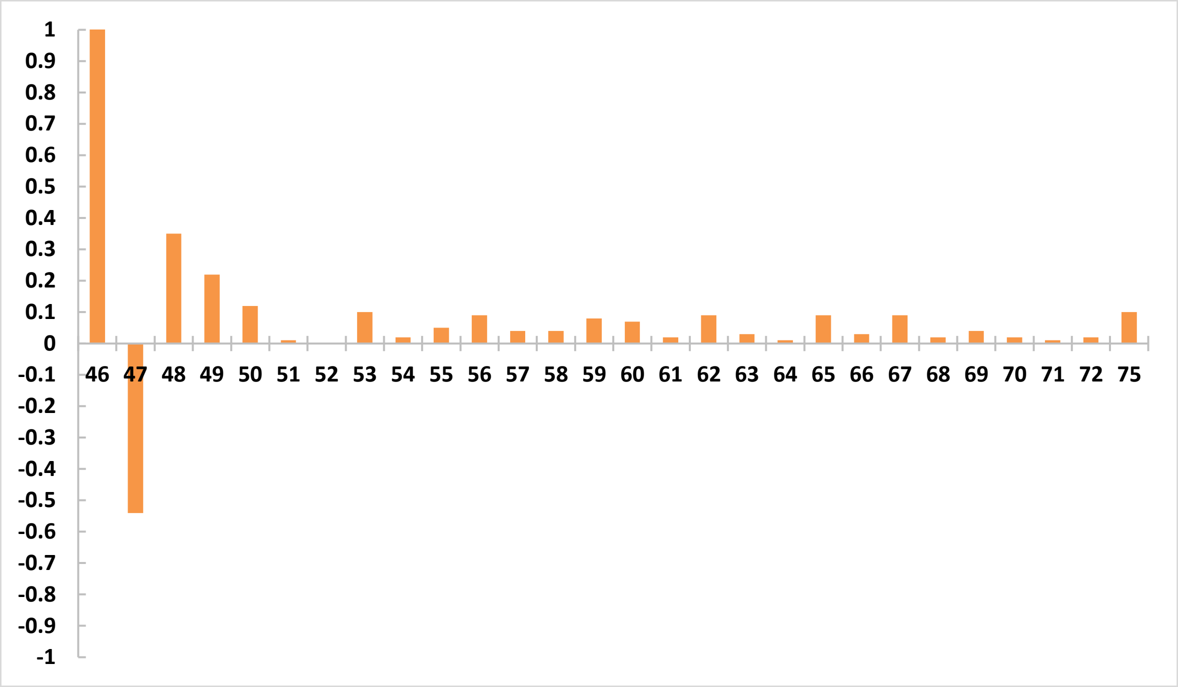

Figure S1. Graphical representation of the ^{13}C NMR shift differences ($\Delta\delta_C$) between the pertinent section of authentic benthol A[7] and the unprotected polyol fragment **32**.

Intelligence Gathering

A/B-Fragment Coupling

Compound S15. In a flame-dried Schlenk flask, 4-methylthiophenol (90 mg, 0.72 mmol) was dissolved in toluene (0.5 mL), before trimethylaluminium (2 M in hexanes, 80 µL, 0.16 mmol) was added at ambient temperature. The mixture was stirred until gas-evolution had ceased before it was stirred at reflux temperature for 1 h. The resulting mixture was cooled to room temperature before a solution of methyl ester **38** (100 mg, 0.080 mmol) in toluene (0.5 mL) was added. The mixture was stirred for *exactly* 15 min and the reaction quenched by addition of sat. aq. Rochelle salt (2 mL) and *tert*-butyl methyl ether (2 mL). The mixture was stirred vigorously for 30 min until two clear layers had formed. The aqueous phase was extracted with pentane (2 × 3 mL), the combined organic layers were washed with brine (2 mL), dried over $MgSO_4$, filtered and concentrated. Purification of the residue by flash chromatography on silica (*n*-pentane/*tert*-butyl methyl ether, 95:5, then 85:15) afforded the title compound as a yellow oil (93 mg, 86%). $[\alpha]_D^{20}$ = −20.8° (c = 0.83 in $CHCl_3$); ^{1}H NMR (600 MHz, $CDCl_3$): δ = 7.30 – 7.27 (m, 2H), 7.22 – 7.18 (m, 2H), 6.04 (ddd, J = 17.0, 10.5, 6.2 Hz, 1H), 5.22 (dt, J = 17.4, 1.6 Hz, 1H), 5.16 (dt, J = 10.5, 1.6 Hz, 1H), 4.35 (dd, J = 10.1, 1.9 Hz, 1H), 4.29 – 4.22 (m, 2H), 4.19 (ddd, J = 6.6, 4.1, 2.7 Hz, 1H), 4.06 – 3.97 (m, 1H), 3.87 (q, J = 3.0 Hz, 1H), 3.78 (d, J = 3.0 Hz, 1H), 3.75 (p, J = 7.0 Hz, 1H), 3.71 – 3.60 (m, 4H), 3.51 (ddd, J = 7.1, 6.0, 4.1 Hz, 1H), 3.09 (s, 1H), 2.84 (dt, J = 15.2, 7.5 Hz, 1H), 2.76 (dt, J = 15.8, 7.7 Hz, 1H), 2.46 – 2.31 (m, 4H), 2.23 (dt, J = 13.3, 6.8 Hz, 1H), 2.00 – 1.91 (m, 2H), 1.91 – 1.83 (m, 1H), 1.81 – 1.73 (m, 1H), 1.72 – 1.60 (m, 2H), 1.60 – 1.44 (m, 5H), 1.44 – 1.36 (m, 3H), 1.31 (m, 2H, *overlap with grease*), 0.91 (s, 9H), 0.90 (s, 9H), 0.89 (s, 9H), 0.89 (s, 18H), 0.87 (s, 9H), 0.12 (s, 3H), 0.10 (s, 3H), 0.09 – 0.07 (m, 12H), 0.06 – 0.03 ppm (m, 18H); ^{13}C NMR (151 MHz, $CDCl_3$): δ = 198.2, 139.6, 138.7, 134.6, 130.1, 124.7, 115.9, 83.3, 81.3, 79.2, 77.8, 76.9 (*by HSQC*), 75.2, 73.4, 73.4, 73.0, 72.2, 70.2, 69.5, 67.4, 43.2, 41.8, 40.6, 39.2, 36.7, 29.6, 29.6, 26.8, 26.4, 26.3, 26.2, 26.0, 26.0, 25.9, 25.9, 21.5, 18.7, 18.5, 18.3, 18.3, 18.2, 18.1, −2.7, −3.6, −3.6, −3.7, −4.3, −4.4, −4.4, −4.6, −4.8, −4.8, −4.9, −4.9 ppm; IR (film) $\tilde{\nu}$ = 2952, 2928, 2896, 2856, 1472, 1463, 1361, 1253, 1074, 1005, 962, 939, 874, 833, 806, 773, 710, 667, 469, 446 cm^{-1}; HRMS (ESI): *m/z*: calcd. for $C_{69}H_{134}O_{11}Si_6SNa$ $[M+Na]^+$: 1361.81548; found: 1361.81499.

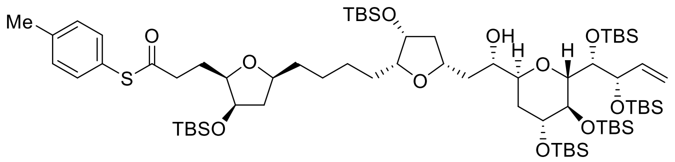

Compound S16. *Catalyst Stock Solution*: A flame-dried Schlenk flask was charged with $Pd_2(dba)_3 \cdot CHCl_3$ (15.8 mg, 15.3 µmol), tris-(2-furyl)-phosphine (28.8 mg, 122 µmol) and THF (1.8 mL). The mixture was sparged with argon for 30 s and sonicated until a homogeneous yellow solution had formed.

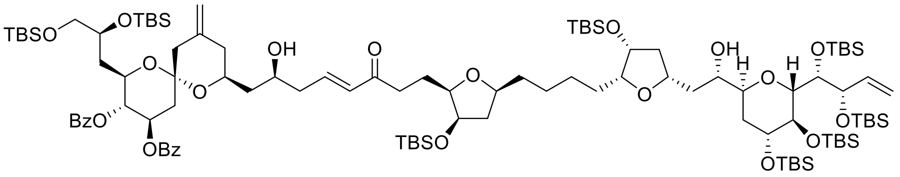

A flame-dried pressure Schlenk flask was charged with thioester **S15** (22.5 mg, 16.8 µmol) and copper diphenylphosphinate (14.1 mg, 50.4 µmol; see below for the preparation). An aliquot of the catalyst stock solution (0.2 mL, corresponding to 20 mol% palladium) was added, immediately followed by a solution of alkenylstannane **41** (25.0 mg, 23.1 mmol; see the accompanying paper) in hexanes (0.1 mL +0.1 mL rinse). The pressure-Schlenk flask was sealed and placed into a pre-heated oil-bath (50 °C bath temperature) for 1 h. The mixture was diluted with *n*-pentane (2 mL) and filtered through a plug of Celite and the filtrates were concentrated in vacuo. Purification of the residue by flash chromatography on silica (*n*-pentane/*tert*-butyl methyl ether, 100:0 to 70:30) afforded the title compound as a pale-brown oil (27.4 mg, 81%). $[\alpha]_D^{20}$ = +1.0° (c = 0.70 in $CHCl_3$); ^{1}H NMR (600 MHz, $CDCl_3$): δ = 7.96 – 7.94 (m, 2H), 7.91 – 7.89 (m, 2H), 7.51 – 7.45 (m, 2H), 7.39 – 7.32 (m, 4H), 6.94 (dt, *J* = 15.9, 7.4 Hz, 1H), 6.22 (dt, J = 15.8, 1.4 Hz, 1H), 6.04 (ddd, *J* = 17.1, 10.5, 6.2 Hz, 1H), 5.63 (ddd, *J* = 11.5, 9.6, 5.3 Hz, 1H), 5.24 – 5.19 (m, 2H), 5.15 (dt, *J* = 10.6, 1.5 Hz, 1H), 4.83 (q, *J* = 1.9 Hz, 1H), 4.79 (q, *J* = 1.8 Hz, 1H), 4.35 (dd, *J* = 10.1, 1.9 Hz, 1H), 4.27 (dd, *J* = 6.3, 1.7 Hz, 1H), 4.24 (ddd, *J* = 6.4, 4.5, 3.5 Hz, 1H), 4.21 – 4.16 (m, 2H), 4.03 – 3.94 (m, 3H), 3.91 (dddd, *J* = 8.4, 6.5, 3.7, 1.9 Hz, 1H), 3.87 (q, *J* = 3.0 Hz, 1H), 3.78 (d, *J* = 3.1 Hz, 1H), 3.73 (pent, *J* = 6.9 Hz, 1H), 3.70 – 3.58 (m, 5H), 3.55 (dd, *J* = 10.7, 3.7 Hz, 1H), 3.50 (ddd, *J* = 7.2, 6.0, 4.1 Hz, 1H), 3.09 (br s, 1H), 2.93 (br s, 1H), 2.79 – 2.67 (m, 2H), 2.52 (dd, *J* = 12.7, 5.3 Hz, 1H), 2.48 – 2.43 (m, 2H), 2.39 – 2.33 (m, 2H), 2.29 – 2.18 (m, 3H), 2.14 (t, *J* = 12.4 Hz, 1H), 1.95 – 1.81 (m, 5H), 1.77 (ddd, *J* = 13.4, 7.0, 2.2 Hz, 1H), 1.71 – 1.59 (m, 5H), 1.55 – 1.47 (m, 5H), 1.43 – 1.36 (m, 3H), 1.33 – 1.29 (m, 2H), 0.91 (s, 9H), 0.90 (s, 9H), 0.89 (s, 9H), 0.89 (s, 9H), 0.88 (s, 9H), 0.87 (s, 9H), 0.83 (s, 9H), 0.80 (s, 9H), 0.12 (s, 3H), 0.10 (s, 3H), 0.09 – 0.07 (m, 12H), 0.06 (s, 3H), 0.06 (s, 3H), 0.06 (s, 3H), 0.05 (s, 3H), 0.04 (s, 3H), 0.04 (s, 3H), 0.03 (s, 3H), 0.00 (s, 3H), 0.00 (s, 3H), −0.01 (s, 3H), −0.05 ppm (s, 3H); ^{13}C NMR (151 MHz, $CDCl_3$): δ = 200.1, 166.2, 165.8, 142.7, 140.1, 138.7, 133.4, 133.1, 132.9, 130.0, 129.9, 129.8, 129.5, 128.5, 128.5, 115.9, 111.4, 98.5, 83.3, 81.9, 79.2, 77.8, 76.9 (*by HSQC*), 75.2, 73.6, 73.4, 73.4, 73.0, 72.2, 71.0, 70.7, 70.2, 69.5, 68.6, 68.0, 67.4, 67.4, 66.6, 43.5, 43.2, 42.4, 41.8, 41.4, 40.8, 39.3, 39.2, 37.2, 36.8, 36.6, 29.5 (*two peaks*), 26.8, 26.8, 26.4, 26.3, 26.2, 26.2, 26.1, 26.0, 26.0, 25.9, 24.3, 18.7, 18.6, 18.5, 18.4, 18.3, 18.2 (*two peaks*), 18.1, −2.7, −3.6, −3.6, −3.7, −4.3, −4.4(*two peaks*), −4.4 (*two peaks*), −4.6, −4.7, −4.8, −4.8, −4.8, −4.9, −5.1, −5.2 ppm; IR (film) $\tilde{\nu}$ = 2953, 2929, 2895, 2856, 1729, 1472, 1463, 1277, 1253, 1175, 1070, 1028, 1005, 961, 939, 834, 811, 775, 711 cm^{-1}; HRMS (ESI): *m/z*: calcd. for $C_{106}H_{192}O_{20}Si_8Na$ $[M+Na]^+$: 2032.20535; found: 2032.20777.

Compound 33. An oven-dried jacketed Schlenk flask was charged with enone **S16** (28.2 mg, 14 µmol) and toluene (1 mL). The solution was cooled to −78 °C (bath temperature) before a solution of the (*R*)-Me-CBS catalyst (11.7 mg, 42 µmol) in toluene (0.2 mL) was added dropwise with the aid of a Hamilton syringe. The mixture was stirred for 30 min before catecholborane (1 M in THF, 80 µL, 80 µmol) was added drop-wise. The mixture was stirred at −78 °C for 30 min and then at −40 °C for another 2.5 h. MeOH (0.5 mL) was added dropwise before the mixture was warmed to ambient temperature. The mixture was diluted with *n*-pentane (20 mL) and then washed with H_2O (5 × 1 mL), aq. citric acid (5% *w/w*, 1 mL), and brine (1 mL). The combined aqueous layers were extracted with pentane (2 × 10 mL). The combined organic phases were dried over Na_2SO_4, filtered, and concentrated. Purification of the residue by flash chromatography on silica (CH_2Cl_2/*tert*-butyl methyl ether, 100:0 to 92.5:7.5) afforded the title compound as a colorless oil which solidified in the freezer (27 mg, 95%). $[\alpha]_D^{20}$ = −8.1° (c = 0.97 in $CHCl_3$); ***Note:*** *Compound* **33** *was obtained as an inseparable mixture of C18-epimers (dr ≈4:1). The NMR data of the major epimer were extracted from the spectra of the mixture.*

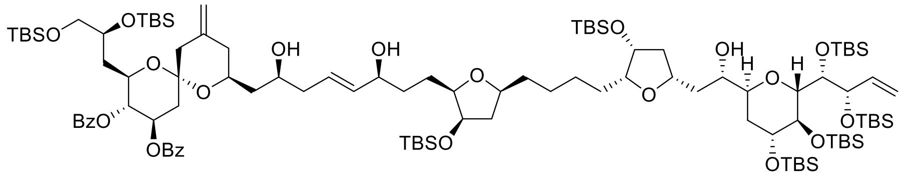

^{1}H NMR (600 MHz, $CDCl_3$): *δ* = 7.96 – 7.94 (m, 2H), 7.91 – 7.88 (m, 2H), 7.51 – 7.45 (m, 2H), 7.38 – 7.31 (m, 4H), 6.04 (ddd, *J* = 17.1, 10.6, 6.2 Hz, 1H), 5.78 – 5.71 (m, 1H), 5.67 – 5.60 (m, 2H), 5.24 – 5.19 (m, 2H), 5.15 (dt, *J* = 10.6, 1.5 Hz, 1H), 4.82 (q, *J* = 2.0 Hz, 1H), 4.78 – 4.76 (m, 1H), 4.35 (dd, *J* = 10.1, 1.9 Hz, 1H), 4.29 – 4.26 (m, 1H), 4.22 – 4.17 (m, 3H), 4.07 – 3.89 (m, 5H), 3.87 (q, *J* = 3.1 Hz, 1H), 3.81 – 3.72 (m, 2H), 3.70 – 3.62 (m, 3H), 3.61 – 3.56 (m, 3H), 3.50 (ddd, *J* = 7.2, 5.9, 4.1 Hz, 1H), 3.09 (br s, 1H), 2.60 (d, *J* = 3.9 Hz, 1H), 2.51 (ddd, *J* = 12.7, 5.3, 3.9 Hz, 1H), 2.42 – 2.33 (m, 3H), 2.31 – 2.20 (m, 5H), 2.11 (t, *J* = 12.5 Hz, 1H), 1.93 – 1.75 (m, 4H), 1.74 – 1.61 (m, 9H), 1.58 – 1.47 (m, 5H), 1.44 – 1.37 (m, 3H), 1.34 – 1.28 (m, 2H), 0.91 (s, 9H), 0.90 (s, 9H), 0.90 – 0.89 (m, 18H), 0.88 (s, 9H), 0.87 (s, 9H), 0.83 – 0.81 (m, 18H), 0.12 (s, 3H), 0.10 (s, 3H), 0.09 – 0.07 (m, 12H), 0.05 (s, 6H), 0.05 (s, 3H), 0.04 – 0.04 (m, 6H), 0.03 (s, 3H), 0.01 (s, 3H), 0.00 – −0.01 (m, 6H), −0.05 ppm (s, 3H); ^{13}C NMR (151 MHz, $CDCl_3$): *δ* = 166.2, 165.9, 140.4, 138.7, 136.8, 133.3, 133.1, 130.0, 129.9, 129.8, 129.6, 128.4 (*two peaks*), 126.8, 115.9, 111.2, 98.3, 83.3, 83.2, 79.2, 78.0, 77.0 (*by HSQC*), 75.2, 73.7, 73.4, 73.4, 73.0, 72.6, 72.2, 70.8, 70.8, 70.2, 69.5, 68.8, 67.7, 67.4, 67.4, 67.3, 43.6, 43.2, 42.3, 41.9, 41.3, 40.8, 39.6, 39.2, 36.7, 36.6, 34.5, 29.5, 29.5, 26.8, 26.8, 26.4, 26.3, 26.2, 26.2, 26.1, 26.0, 26.0, 25.9, 25.6, 18.7, 18.5, 18.5, 18.4, 18.3, 18.2 (*two peaks*), 18.1, −2.7, −3.6, −3.6, −3.7, −4.3, −4.3, −4.4, −4.4, −4.6, −4.7, −4.8, −4.8, −4.8, −4.9, −5.1, −5.1 ppm;IR (film) $\tilde{\nu}$ = 2952, 2929, 2895, 2856, 1729, 1472, 1463, 1361, 1277, 1254, 1175, 1071, 1028, 1005, 939, 834, 811, 775, 710, 671 cm^{-1}; HRMS (ESI): *m/z*: calcd. for $C_{106}H_{194}O_{20}Si_8Na$ [M+Na]$^+$: 2034.22100; found: 2034.22214.

All attempts at subjecting this compound to hydroboration with (9-H-9-BBN)$_2$ followed by cross coupling of the organoborane intermediates formed with alkenyl iodides such as **34** resulted in complex mixtures. The signals attributed to the C10=C73 methylene group could not be detected by NMR inspection of the crude materials.

Model Studies on B/C-Fragment Coupling

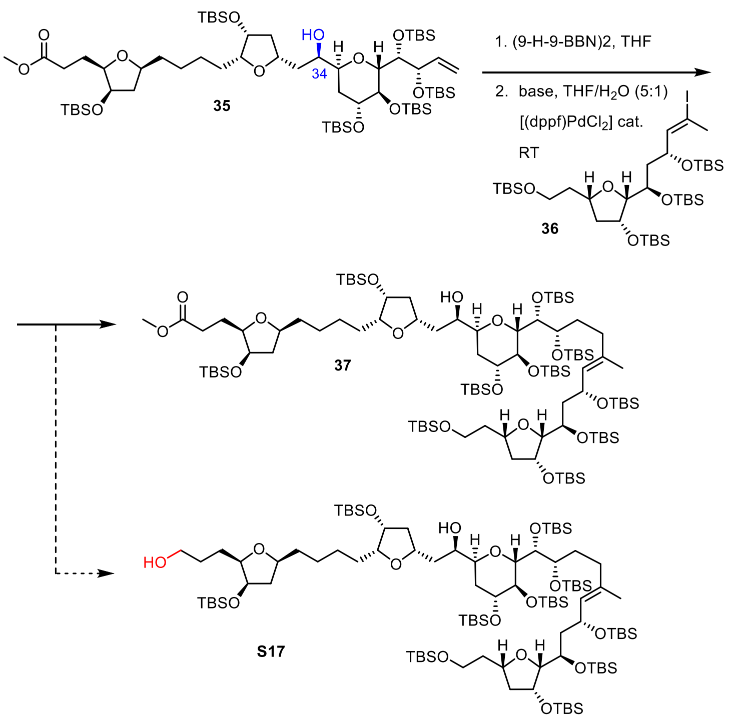

Scheme S1. Model study for the projected coupling of fragments **B** and **C**

Table S6. Optimization of the alkyl-Suzuki reaction meant to serve the coupling of fragments **B** and **C** using compound **35** (epimeric at C34) as the model reaction partner

##	**35**	$[9\text{-H-9-BBN}]_2$	T (°C)	t (h)	[Pd]	Base	t (h)	Yield
1	2 eq.	10 eq.	0 to RT	1.5	20 mol%[a]	Cs_2CO_3 (21 eq.)	2[b]	trace
2							20[b]	42%
3	1.2 eq.	3 eq.	60	1	10 mol%	NaOH (3 eq.)	2	47%
4	1.2 eq.	6 eq.	60	1	10 mol%	8 eq.	3	31% + **S17**
5	2 eq.	10 eq.	0 to RT	1.5	10 mol%	27 eq.	2	40%
6						22 eq.	2	63%
7						22 eq.	20	15%
8						15 eq.	2	67%
9						10 eq.	2	75%
10						3 eq.	2	54%

[a] with $[Pd(PPh_3)_4]$ as catalyst; [b] in $THF/DMF/H_2O$

Compound 37 (Table S6, entry 9). An oven-dried Schlenk tube was charged with 9-BBN-dimer (7.3 mg, 30.0 µmol) and THF (0.1 mL). The solution was cooled at 0 °C (bath temperature) before a solution of alkene **35** (7.5 mg, 6.0 µmol) in THF (0.2 mL) was added dropwise. The mixture was stirred at room temperature for 1.5 h. The resulting solution of the corresponding alkyl-9-BBN derivative was used directly in the cross-coupling reaction.

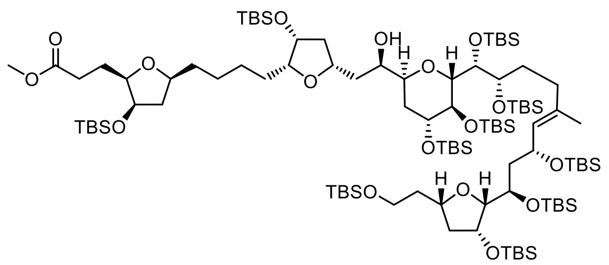

NaOH solution (0.30 M in water, 0.1 mL, 30.0 µmol) was added to the solution containing the alkyl-9-BBN species and the resulting mixture was stirred vigorously at room temperature for 30 min. Alkenyl iodide **36** (2.5 mg, 3.0 µmol), THF (0.2 mL) and Pd(dppf)Cl_2 (0.2 mg, 0.3 µmol) were then added and stirring was continued at room temperature for 2 h. The mixture was diluted with sat. aq. NH_4Cl solution (10 mL) and then extracted with EtOAc (3 x 10 mL). The combined organic layers were washed with brine (10 mL), dried over Na_2SO_4, and concentrated under reduced pressure. The residue was purified by flash chromatography (silica; hexane/EtOAc, 10:1) to give the title compound as a pale-yellow oil (4.41 mg, 75%). $[\alpha]_D^{20}$ = –2.1° (*c* = 1.73, CH_2Cl_2). ^{1}H NMR (400 MHz, CD_2Cl_2) δ 5.18 – 5.11 (m, 1H), 4.65 (td, *J* = 9.3, 3.7 Hz, 1H), 4.52 – 4.41 (m, 1H), 4.38 (ddd, *J* = 6.3, 4.7, 3.5 Hz, 1H), 4.24 (ddd, *J* = 6.5, 4.7, 3.6 Hz, 1H), 4.22 – 4.14 (m, 2H), 4.03 – 3.77 (m, 5H), 3.75 – 3.66 (m, 4H), 3.65 – 3.41 (m, 8H), 3.24 (s, 1H), 2.49 – 2.29 (m, 3H), 2.28 – 2.19 (m, 2H), 1.99 – 1.80 (m, 8H), 1.77 – 1.70 (m, 2H), 1.69 – 1.56 (m, 10H), 1.53 – 1.43 (m, 4H), 1.43 – 1.36 (m, 2H), 1.34 – 1.28 (m, 2H), 0.93 – 0.87 (m, 90H), 0.16 (s, 3H), 0.13 (s, 3H), 0.11 – 0.05 (m, 42H), 0.04 (s, 6H), 0.03 (s, 3H), 0.01 (s, 3H). ^{13}C NMR (101 MHz, CD_2Cl_2) δ 174.4, 134.2, 130.1, 86.5, 84.2, 81.8, 77.9, 77.7, 74.5, 74.4, 73.7, 73.4, 73.2, 71.5, 70.3, 69.5, 68.6, 67.8, 67.4, 60.9, 51.6, 43.0, 42.6, 42.1, 39.6, 39.4, 37.0, 36.7, 31.2, 29.9, 28.9, 27.0, 26.9, 26.5, 26.4, 26.4, 26.4, 26.1, 26.1, 26.0 (two peaks), 25.7, 18.7, 18.7, 18.6, 18.5, 18.5, 18.4, 18.4, 18.4, 18.3, 17.3, –2.8, –3.2, –3.3, –3.9 (two peaks), –4.1, –4.2, –4.3, –4.4, –4.4, –4.4, –4.7, –5.0 (two peaks), –5.2, –5.2. ***Note**: Some carbon signals could not be detected because of overlapping or extremely weak intensities.* IR (film) $\tilde{\nu}$ 3357, 2953, 2927, 2855, 1743, 1649, 1471, 1462, 1361, 1252, 1079, 1005, 938, 833, 773 cm^{-1}. HRMS (ESI) *m/z* calcd. for $C_{99}H_{208}O_{17}Si_{10}Na$ $[M+Na]^+$: 1972.29964, found: 1972.30002.

Completion of the Total Synthesis of Nominal Benthol A

Compound 39. An oven-dried Schlenk tube was charged with 9-BBN-dimer (44.0 mg, 180.0 µmol) and THF (0.2 mL). The solution was cooled at 0 °C (bath temperature) before a solution of alkene **38** (45.0 mg, 36.0 µmol) in THF (0.8 mL) was added dropwise. The mixture was stirred at room temperature for 1.5 h. The resulting solution of the corresponding alkyl-9-BBN derivative was directly used in the cross-coupling reaction.

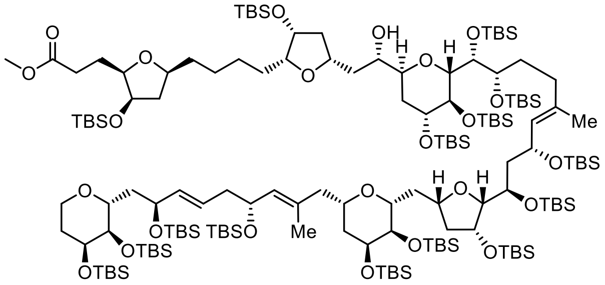

NaOH (0.45 M in water, 0.4 mL, 180.0 µmol) was added to the solution of the alkyl-9-BBN reagent and the resulting mixture was stirred vigorously at room temperature for 30 min. Alkenyl iodide **30** (31.9 mg, 18.0 µmol), THF (1.0 mL) and Pd(dppf)Cl_2 (1.3 mg, 1.8 µmol) were then added and stirring continued for 6 h. The mixture was diluted with sat. aq. NH_4Cl solution (10 mL) and extracted with EtOAc (3 x 10 mL). The combined organic layers were washed with brine (10 mL), dried over Na_2SO_4, and concentrated under reduced pressure. Purification of the residue by flash chromatography (silica; toluene/*tert*-butyl methyl ether, 10:1) gave the title compound as a pale-yellow oil (28.7 mg, 55%; 75% brsm) as well as a second fraction consisting of recovered alkenyl iodide (8.6 mg, 27%). $[\alpha]_D^{20}$ = +9.9° (*c* = 0.98, CH_2Cl_2). ^{1}H NMR (600 MHz, C_6D_6) δ 6.02 – 5.92 (m, 1H), 5.82 (ddt, *J* = 15.4, 6.7, 1.3 Hz, 1H), 5.52 (d, *J* = 8.7 Hz, 1H), 5.44 – 5.38 (m, 1H), 5.08 (dd, *J* = 7.4, 6.8 Hz, 1H), 4.80 (br, 1H), 4.71 (ddd, *J* = 9.8, 6.3, 3.3 Hz, 1H), 4.58 (ddd, *J* = 8.5, 7.0, 5.5 Hz, 1H), 4.47 (dd, *J* = 10.6, 5.5 Hz, 1H), 4.43 (ddd, *J* = 6.9, 4.3, 2.8 Hz, 1H), 4.36 – 4.16 (m, 4H), 4.14 – 3.82 (m, 12H), 3.69 – 3.63 (m, 1H), 3.63 – 3.53 (m, 3H), 3.42 (br, 1H), 3.39 (dd, *J* = 9.0, 2.3 Hz, 1H), 3.37 – 3.33 (m, 4H), 3.23 (br, 1H), 2.59 – 2.50 (m, 3H), 2.45 – 2.33 (m, 3H), 2.28 (br, 1H), 2.26 – 2.20 (m, 3H), 2.19 – 2.08 (m, 7H), 2.05 – 1.99 (m, 2H), 1.97 – 1.89 (m, 6H), 1.86 – 1.82 (m, 1H), 1.80 (d, *J* = 1.4 Hz, 3H), 1.77 – 1.71 (m, 3H), 1.70 – 1.63 (m, 3H), 1.62 – 1.53 (m, 4H), 1.45 – 1.34 (m, 7H), 1.20 – 1.08 (m, 81H), 1.07 – 1.02 (m, 36H), 1.01 (s, 9H), 0.97 (s, 9H), 0.45 – 0.28 (m, 24H), 0.26 (s, 3H), 0.25 (s, 3H), 0.24 (s, 3H), 0.24 (s, 3H), 0.23 (s, 3H), 0.22 (s, 3H), 0.20 (s, 3H), 0.19 (s, 3H), 0.19 – 0.16 (m, 9H), 0.16 – 0.14 (m, 6H), 0.12 (s, 6H), 0.11 – 0.09 (m, 6H), 0.06 (s, 3H), 0.05 – 0.04 (m, 6H), 0.03 (s, 3H), 0.02 (s, 3H). ^{13}C NMR (151 MHz, C_6D_6) δ 173.6, 135.7, 134.8, 133.0, 132.1, 129.8, 127.1, 87.8, 83.6, 81.6, 78.1, 77.6, 75.3, 75.3, 75.2, 75.1, 73.9, 73.8, 73.4 (two peaks), 73.0, 71.7, 71.2, 70.7, 70.6, 70.3, 69.9, 68.5, 68.0, 67.7, 61.7, 51.0, 46.3, 44.8, 42.6, 42.4, 42.3, 42.1, 41.9, 40.5, 38.6, 37.1, 34.8, 31.1, 30.1, 27.2, 27.1, 26.7, 26.7, 26.6, 26.6, 26.5, 26.5, 26.3, 26.3, 26.3, 26.3, 26.2, 26.1, 25.9, 18.8, 18.6, 18.6, 18.5, 18.4, 18.4, 18.3, 18.3, 18.0, 17.7, –1.3, –2.4, –2.5, –2.9, –3.2, –3.3, –3.3, –3.5, –3.6, –3.7, –3.7, –3.8, –3.9, –4.0, –4.0, –4.2, –4.2, –4.3, –4.3, –4.3, –4.4, –4.4, –4.5, –4.7, –4.9, –4.9. ***Note****: Some carbon resonances could not be observed because of signal overlap or extremely*

weak intensities. IR (film) $\tilde{\nu}$ 3530, 2953, 2928, 2894, 2856, 1743, 1472, 1463, 1361, 1253, 1082, 1005, 940, 834, 774 cm^{-1}. HRMS (ESI) *m/z* calcd. for $C_{148}H_{308}O_{24}Si_{15}Na_2$ $[M+2Na]^{2+}$: 1467.96020, found: 1467.95895.

Tris(*p*-tolylthio)aluminum.[8] An oven-dried Schlenk tube was charged with 4-methylbenzenethiol (81.0 mg, 652.1 µmol) and toluene (1.0 mL). The mixture was stirred at room temperature while trimethyl aluminum (2.0 M in hexane, 72.0 µL, 144.0 µmol) was added dropwise. After stirring at room temperature for 10 min, the solution was warmed to 120 °C (bath temperature) for 1 h. After reaching room temperature, the mixture formed a wet white solid. This material was used in the next step.

Compound 40. An oven-dried Schlenk tube was charged with methyl ester **39** (20.0 mg, 6.9 µmol) and

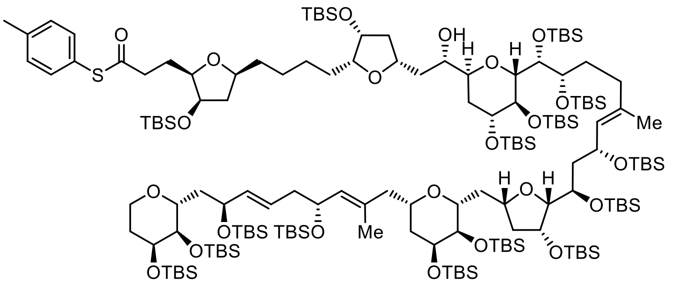

toluene (0.5 mL). The solution was stirred vigorously at room temperature while freshly prepared tris(*p*-tolylthio)aluminum was added in small portions until the starting methyl ester was fully consumed (ca. 30 min). Once TLC indicated complete conversion, the addition of tris(*p*-tolylthio)aluminum was stopped and the reaction was immediately quenched with sat. Rochelle salt solution (5 mL). The resulting mixture was stirred vigorously at room temperature for 30 min and then extracted with EtOAc (3 x 10 mL). The combined organic layers were dried over Na_2SO_4 and concentrated under reduced pressure, and the residue was purified by flash chromatography (silica; hexane/EtOAc, 20:1 to 10:1) to give the title compound as a colorless oil (18.4 mg, 89%). $[\alpha]_D^{20}$ = +10.8° (*c* = 1.43, CH_2Cl_2). ^1H NMR (600 MHz, C_6D_6) δ 7.38 – 7.32 (m, 2H), 6.88 (m, 2H), 6.02 – 5.93 (m, 1H), 5.83 (ddt, *J* = 15.3, 6.6, 1.3 Hz, 1H), 5.53 (d, *J* = 8.7 Hz, 1H), 5.45 – 5.39 (m, 1H), 5.08 (dd, *J* = 7.1, 6.5 Hz, 1H), 4.81 (br, 1H), 4.71 (ddd, *J* = 10.7, 7.0, 4.3 Hz, 1H), 4.58 (ddd, *J* = 8.5, 7.0, 5.6 Hz, 1H), 4.47 (dd, *J* = 6.2, 6.2 Hz, 1H), 4.43 (ddd, *J* = 7.0, 4.2, 2.9 Hz, 1H), 4.35 – 4.16 (m, 4H), 4.15 – 3.82 (m, 12H), 3.69 – 3.64 (m, 1H), 3.61 (ddd, *J* = 11.0, 5.2, 1.8 Hz, 1H), 3.59 – 3.52 (m, 2H), 3.44 (br, 1H), 3.39 (dd, *J* = 9.1, 2.4 Hz, 1H), 3.35 (dd, *J* = 8.9, 2.4 Hz, 1H), 3.23 (br, 1H), 2.92 – 2.78 (m, 2H), 2.60 – 2.52 (m, 1H), 2.46 – 2.33 (m, 3H), 2.29 (br, 1H), 2.27 – 2.21 (m, 3H), 2.20 – 2.05 (m, 9H), 1.99 (s, 3H), 1.97 – 1.89 (m, 6H), 1.88 – 1.84 (m, 1H), 1.80 (d, *J* = 1.4 Hz, 3H), 1.79 – 1.72 (m, 3H), 1.72 – 1.63 (m, 3H), 1.62 – 1.54 (m, 4H), 1.48 – 1.33 (m, 7H), 1.22 – 1.07 (m, 81H), 1.07 – 1.02 (m, 36H), 1.01 (s, 9H), 0.97 (s, 9H), 0.44 – 0.29 (m, 24H), 0.26 (s, 3H), 0.25 (s, 3H), 0.25 (s, 3H), 0.24 (s, 3H), 0.24 (s, 3H), 0.23 (s, 3H), 0.21 (s, 3H), 0.20 (s, 3H), 0.19 – 0.16 (m, 9H), 0.16 – 0.14 (m, 6H), 0.14 – 0.12 (m, 6H), 0.12 – 0.09 (m, 6H), 0.07 (s, 3H), 0.06 – 0.03 (m, 6H), 0.03 (s, 3H), 0.02 (s, 3H). ^{13}C NMR (151 MHz, C_6D_6) δ 196.6, 139.2, 135.7, 135.0, 134.9, 133.0, 132.1, 130.1, 129.8, 127.1, 125.5, 87.8, 83.6, 81.3, 78.0, 77.7, 75.3, 75.3, 75.2, 75.1, 73.8, 73.4 (two peaks), 73.0, 71.7, 71.2, 70.7, 70.6, 70.3, 69.9, 68.5, 67.9, 67.7,

61.7, 46.3, 44.8, 42.6, 42.4, 42.3, 42.1, 41.9, 40.9, 40.5, 38.6, 37.1, 34.8, 30.1, 27.2, 27.1, 26.7, 26.7, 26.6, 26.6, 26.5, 26.5, 26.3, 26.3, 26.3, 26.3, 26.2, 26.1, 21.1, 18.8, 18.7, 18.6, 18.5, 18.5, 18.4, 18.3, 18.3, 18.0, 17.7, −1.3, −2.3, −2.5, −2.9, −3.2, −3.3, −3.3, −3.5, −3.6, −3.7, −3.7, −3.8, −3.9, −4.0, −4.0, −4.1, −4.2, −4.3, −4.3, −4.3, −4.4, −4.4, −4.7, −4.8, −4.9. ***Note**: Some carbon resonances could not be observed because of signal overlap or extremely weak intensities.* IR (film) $\tilde{\nu}$ 3511, 2953, 2928, 2893, 2857, 1713, 1472, 1462, 1361, 1254, 1082, 1005, 957, 940, 835, 774 cm^{-1}. HRMS (ESI) *m/z* calcd. for $C_{154}H_{312}O_{23}SSi_{15}Na_2$ $[M+2Na]^{2+}$: 1513.96443, found: 1513.96371.

Copper(I) diphenylphosphinate (CuDPP).[9] An oven-dried Schlenk tube equipped with a Dean-Stark trap was charged with diphenylphosphinic acid (1.31 g, 6.0 mmol), Cu_2O (0.43 g , 3.0 mmol) and degassed toluene (40 mL). The mixture was stirred at reflux temperature with constant removal of water for 20 h. The mixture was cooled to room temperature and the suspension filtered under Argon. The filter residue was washed with degassed toluene (10 mL), Et_2O (5 ml) and hexanes (10 mL) and then dried under reduced pressure to give copper(I) diphenylphosphinate as a light-tan solid that was used in the coupling without further purification.

Compound 42. *Catalyst Stock Solution:* An oven-dried Schlenk tube was charged with $Pd_2(dba)_3{\cdot}CHCl_3$ (1.5 mg, 1.4 µmol), P(2-furyl)$_3$ (2.7 mg, 11.2 µmol) and degassed THF (600 µL). The mixture was stirred at room temperature until a homogeneous pale yellow solution had formed (ca. 3 min) which was used in the cross coupling step.

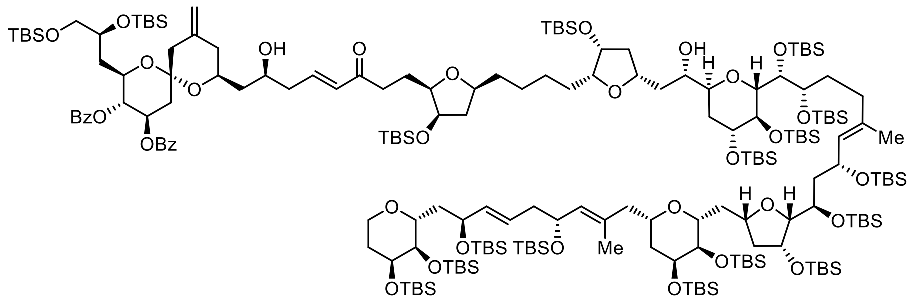

An oven-dried Schlenk tube was charged with thioester **40** (14.3 mg, 4.8 µmol), CuDPP (4.0 mg, 14.4 µmol) and THF (50 µL). An aliquot of the Pd catalyst solution (200 µL) was added, followed by the quick addition of a solution of alkenylstannane **41** (7.8 mg, 7.2 µmol; see the accompanying paper) in hexanes (250 µL). The mixture was stirred at 50 °C (bath temperature) for 7 h before it was allowed to reach room temperature. The mixture was filtered through a short pad of Celite, thoroughly rinsing with *tert*-butyl methyl ether (5 mL). The combined filtrates were concentrated under reduced pressure and the residue was purified by flash chromatography (silica; CH_2Cl_2/acetonitrile, 20:1 to 10:1) to give the title compound as a colorless oil (14.7 mg, 84%). $[\alpha]_D^{20}$ = +13.2° (*c* = 1.47, CH_2Cl_2). ^{1}H NMR (600 MHz, C_6D_6) δ 8.14 – 8.10 (m, 4H), 7.05 – 6.97 (m, 4H), 6.96 – 6.93 (m, 2H), 6.88 (dt, *J* = 15.9, 7.3 Hz, 1H), 6.16 (dt, *J* = 15.8, 1.4 Hz, 1H), 6.07 (ddd, *J* = 11.5, 9.7, 5.3 Hz, 1H), 6.00 – 5.92 (m, 1H), 5.82 (ddt, *J* = 15.4, 6.6, 1.3 Hz, 1H), 5.55 (dd, *J* = 9.8, 9.8

Hz, 1H), 5.52 (d, *J* = 8.8 Hz, 1H), 5.44 – 5.39 (m, 1H), 5.08 (dd, *J* = 14.7, 6.7 Hz, 1H), 4.89 (dd, *J* = 2.0, 2.0 Hz, 1H), 4.86 (dd, *J* = 2.0, 2.0 Hz, 1H), 4.80 (br, 1H), 4.70 (ddd, *J* = 9.8, 6.3, 3.8 Hz, 1H), 4.58 (ddd, *J* = 8.6, 7.1, 5.6 Hz, 1H), 4.46 (dd, *J* = 11.1, 4.9 Hz, 1H), 4.42 (ddd, *J* = 4.2, 4.2, 2.6 Hz, 1H), 4.35 – 4.06 (m, 11H), 4.05 – 3.96 (m, 6H), 3.95 – 3.90 (m, 2H), 3.89 – 3.81 (m, 3H), 3.79 (dd, *J* = 10.8, 2.9 Hz, 1H), 3.72 – 3.63 (m, 2H), 3.60 (ddd, *J* = 11.1, 5.2, 1.8 Hz, 1H), 3.57 (br, 1H), 3.42 (br, 1H), 3.38 (dd, *J* = 9.0, 2.4 Hz, 1H), 3.35 (dd, *J* = 9.0, 2.4 Hz, 1H), 3.23 (br, 1H), 2.81 – 2.68 (m, 2H), 2.56 (dt, *J* = 12.6, 6.2 Hz, 1H), 2.45 – 2.40 (m, 2H), 2.40 – 2.32 (m, 2H), 2.32 – 2.26 (m, 2H), 2.25 – 2.19 (m, 5H), 2.19 – 2.13 (m, 5H), 2.13 – 2.03 (m, 7H), 2.02 – 1.97 (m, 3H), 1.96 – 1.94 (m, 3H), 1.94 – 1.88 (m, 3H), 1.86 – 1.82 (m, 2H), 1.80 (d, *J* = 1.4 Hz, 3H), 1.77 – 1.71 (m, 2H), 1.71 – 1.63 (m, 3H), 1.62 – 1.52 (m, 6H), 1.50 – 1.47 (m, 2H), 1.45 – 1.39 (m, 5H), 1.20 – 1.08 (m, 81H), 1.07 – 1.03 (m, 27H), 1.03 (s, 9H), 1.00 (s, 9H), 1.00 – 0.99 (m, 18H), 0.97 (s, 9H), 0.49 – 0.32 (m, 18H), 0.32 – 0.30 (m, 6H), 0.26 – 0.22 (m, 18H), 0.21 – 0.17 (m, 21H), 0.15 – 0.14 (m, 6H), 0.13 – 0.11 (m, 9H), 0.11 – 0.09 (m, 9H), 0.09 (s, 3H), 0.07 – 0.05 (m, 6H), 0.05 – 0.03 (m, 6H). ^{13}C NMR (151 MHz, C_6D_6) δ 198.6, 166.3, 165.8, 142.0, 141.0, 135.7, 134.8, 133.3, 133.2, 133.1, 132.9, 132.0, 130.4, 130.2, 130.0, 130.0, 129.8, 128.7, 128.6, 127.1, 111.0, 98.5, 87.8, 83.6, 82.1, 78.0, 77.7, 75.3, 75.2, 75.1, 75.0, 74.2, 73.9, 73.8, 73.4 (two peaks), 72.9, 72.2, 71.7, 71.5, 71.4, 71.2, 71.0, 70.7, 70.6, 70.2, 69.9, 69.8, 69.1, 68.5, 68.2, 68.1, 67.9, 67.7, 67.6, 66.7, 61.7, 46.3, 44.8, 43.6, 43.0, 42.6, 42.4, 42.3, 42.1, 41.8, 41.4, 40.8, 40.4, 40.0, 38.6, 38.0, 37.4, 37.3, 37.2, 36.5, 34.8, 30.1, 29.3, 27.3, 27.2, 26.7, 26.7, 26.6, 26.6, 26.5, 26.5, 26.4, 26.4, 26.3, 26.3, 26.3, 26.3, 26.3, 26.2, 26.1, 26.1, 24.7, 18.8, 18.7, 18.6, 18.6, 18.6, 18.6, 18.5, 18.5, 18.5, 18.4, 18.4, 18.3, 18.0, 17.7, –1.3, –2.3, –2.5, –2.9, –3.2, –3.3, –3.3, –3.5, –3.6, –3.7, –3.7, –3.8, –4.0, –4.0, –4.1, –4.1, –4.1, –4.2, –4.2, –4.3, –4.3, –4.3, –4.4, –4.4, –4.4, –4.5, –4.8, –4.9, –4.9, –5.0. ***Note**: Some carbon resonances could not be observed because of signal overlap or extremely weak intensities.* IR (film) $\tilde{\nu}$ 3524, 2954, 2927, 2856, 1731, 1675, 1632, 1462, 1361, 1255, 1084, 1006, 951, 835, 774 cm^{-1}. HRMS (ESI) *m/z* calcd. for $C_{191}H_{370}O_{32}Si_{17}Cs$ $[M+Cs]^+$: 3785.24518, found: 3785.24232.

Compound 43. *Preparation of the CBS-Catalyst:* An oven-dried Schlenk tube was charged with (*R*)-(+)-2-methyl-CBS-oxazaborolidine (3.6 mg, 11.3 µmol) and toluene (45.0 µL). The mixture was sonicated for 20 min to give a white suspension that was used for the reduction.

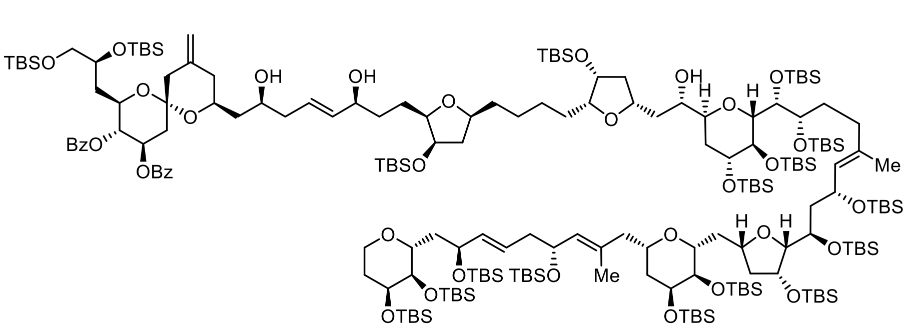

This solution containing the (*R*)-(+)-2-methyl-CBS-oxazaborolidine (0.25 M in toluene, 45.0 µL, 11.3 µmol) was added at –78 °C (bath temperature) to a solution of ketone **42** (13.8 mg, 3.8 µmol) in toluene (100.0 µL). The mixture was

stirred at this temperature for 30 min before catecholborane (1.0 M in THF, 22.6 µL, 22.6 µmol) was added dropwise. The mixture was allowed to slowly warm to –20 °C over the course of 4 h. The reaction was quenched with MeOH (0.5 mL), the mixture was diluted with sat. aq. NH_4Cl solution (5 mL) and then extracted with EtOAc (3 x 10 mL). The combined organic layers were washed with sat. aq. Na_2CO_3 solution until the aqueous layer becomes colorless, and then with brine (20 mL). The organic phase was dried over Na_2SO_4 and concentrated under reduced pressure, and the residue was purified by flash chromatography (silica; hexane/EtOAc, 10:1 to 3:1) to give the title compound as a colorless oil (9.73 mg, 70%). $[\alpha]_D^{20}$ = +12.9° (*c* = 1.42, CH_2Cl_2). ^1H NMR (600 MHz, C_6D_6) δ 8.16 – 8.07 (m, 4H), 7.03 – 6.92 (m, 6H), 6.11 (ddd, *J* = 11.5, 9.7, 5.3 Hz, 1H), 5.97 (dddd, *J* = 15.3, 8.3, 5.9, 1.1 Hz, 1H), 5.82 (ddt, *J* = 15.3, 6.6, 1.3 Hz, 1H), 5.74 – 5.63 (m, 2H), 5.58 (dd, *J* = 9.8, 9.8 Hz, 1H), 5.52 (d, *J* = 8.8 Hz, 1H), 5.45 – 5.40 (m, 1H), 5.08 (dd, *J* = 14.8, 6.7 Hz, 1H), 4.91 (dd, *J* = 2.1, 2.1 Hz, 1H), 4.88 (dd, *J* = 2.0, 2.0 Hz, 1H), 4.81 (br, 1H), 4.71 (ddd, *J* = 9.8, 6.1, 3.6 Hz, 1H), 4.58 (ddd, *J* = 8.6, 7.0, 5.6 Hz, 1H), 4.47 (dd, *J* = 11.0, 5.5 Hz, 1H), 4.45 – 4.40 (m, 1H), 4.37 – 4.15 (m, 8H), 4.15 – 4.04 (m, 5H), 4.04 – 3.92 (m, 6H), 3.92 – 3.78 (m, 5H), 3.74 – 3.67 (m, 1H), 3.63 – 3.59 (m, 1H), 3.57 (br, 1H), 3.52 (dt, *J* = 8.4, 4.1 Hz, 1H), 3.43 (br, 1H), 3.39 (dd, *J* = 9.0, 2.4 Hz, 1H), 3.35 (dd, *J* = 9.0, 2.4 Hz, 1H), 3.30 – 3.19 (m, 1H), 2.60 – 2.52 (m, 1H), 2.50 – 2.32 (m, 5H), 2.32 – 2.07 (m, 18H), 2.06 – 2.02 (m, 2H), 2.01 – 1.89 (m, 10H), 1.87 – 1.81 (m, 4H), 1.80 (d, *J* = 1.4 Hz, 3H), 1.79 – 1.73 (m, 2H), 1.70 – 1.63 (m, 3H), 1.62 – 1.51 (m, 6H), 1.48 (ddd, *J* = 13.0, 7.1, 3.1 Hz, 1H), 1.45 – 1.39 (m, 4H), 1.20 – 1.08 (m, 81H), 1.08 – 1.04 (m, 27H), 1.04 – 1.03 (m, 9H), 1.02 – 1.02 (m, 9H), 1.02 – 1.00 (m, 18H), 0.98 (s, 9H), 0.47 – 0.32 (m, 18H), 0.32 – 0.30 (m, 6H), 0.27 – 0.21 (m, 24H), 0.21 – 0.17 (m, 15H), 0.16 – 0.14 (m, 9H), 0.14 – 0.12 (m, 9H), 0.11 – 0.09 (m, 6H), 0.09 – 0.05 (m, 9H), 0.05 – 0.03 (m, 6H). ^{13}C NMR (151 MHz, C_6D_6) δ 166.3, 166.0, 141.3, 138.0, 135.7, 134.8, 133.1, 133.0, 133.0, 132.1, 130.4, 130.2, 130.1, 130.0, 129.8, 128.6, 128.6, 127.1, 126.5, 110.9, 98.4, 87.9, 83.6, 83.4, 78.0, 77.8, 75.3, 75.2, 75.1, 75.0, 74.3, 74.0, 73.7, 73.4 (two peaks), 72.9, 72.7, 72.2, 71.7, 71.4, 71.2 (two peaks), 71.1, 70.7, 70.6, 70.2, 69.9, 69.1, 68.5, 68.4, 67.9, 67.8, 67.7, 67.7, 67.3, 61.7, 46.3, 44.8, 43.7, 43.0, 42.6, 42.4, 42.4, 42.1, 41.8, 40.9, 40.4, 40.2, 38.6, 38.0, 37.3, 37.2, 36.6, 35.1, 34.8, 30.0, 29.3, 27.2 (two peaks), 26.7, 26.7, 26.6, 26.6, 26.5, 26.5, 26.4, 26.3, 26.3, 26.3, 26.3, 26.2, 26.2, 26.1, 18.8, 18.7, 18.7, 18.6, 18.6, 18.6, 18.5, 18.5, 18.5, 18.4, 18.3, 18.0, 17.7, –1.3, –2.3, –2.5, –2.9, –3.2, –3.3, –3.3, –3.5, –3.6, –3.7, –3.7, –3.8, –4.0, –4.0, –4.1, –4.1, –4.1, –4.2, –4.3, –4.3, –4.3, –4.4, –4.5, –4.8, –4.8, –4.9. ***Note****: Some carbon resonances could not be observed because of signal overlap or extremely weak intensities.* IR (film) $\tilde{\nu}$ 3515, 2953, 2927, 2855, 1730, 1675, 1462, 1361, 1253, 1083, 1005, 967, 834, 774 cm^{-1}. HRMS (ESI) *m/z* calcd. for $C_{191}H_{372}O_{32}Si_{17}Na_2$ $[M+Na]^{2+}$: 1850.16719, found: 1850.16762.

Nominal Benthol A (1). A dry Schlenk flask was charged with compound **43** (11.5 mg, 3.14 µmol), K_2CO_3 (4.3 mg, 31.4 µmol), THF (1.0 mL) and MeOH (1.0 mL). The mixture was stirred at room temperature for 21 h before all volatile materials were removed under reduced pressure. The residue was diluted with brine (10 mL) and extracted with EtOAc (3 x 10 mL). The combined organic layers were dried over Na_2SO_4 and concentrated under reduced pressure to give a residue that was used in the next step without further purification.

TBAF (1.0 M in THF, 107.0 µL, 107.0 µmol) was added dropwise at 0 °C (bath temperature) to a solution of the crude product in THF (1.0 mL). The mixture was stirred at room temperature for 24 h before it was diluted with EtOAc (10 mL). The resulting mixture was concentrated under reduced pressure. A mixed solvent (hexanes/ CH_2Cl_2, 3:1, 10 mL) was added to the oily residue and the resulting suspension was filtered through a plug of cotton; the solid material was kept for further processing. The cotton was rinsed with the mixed solvent (3 x 2 mL) and the organic filtrates were discarded. The cotton was then rinsed with MeOH (3 x 2 mL). The MeOH filtrates were mixed with the solid material precipitated from the mixed solvent and the combined solution was then concentrated under reduced pressure. The washing procedure was repeated until all TBAF by-product was removed. The highly polar residue was purified by flash chromatography (silica gel 60 cyano, H_2O/MeOH = 9:1) to give the title compound as a white solid (3.51 mg, 74% over two steps). $[\alpha]_D^{25}$ = +20.6° (*c* = 0.35, MeOH). ^{1}H NMR (600 MHz, [D$_4$]-MeOH) δ 5.73 – 5.61 (m, 2H), 5.58 – 5.48 (m, 2H), 5.30 (dq, *J* = 8.7, 1.3 Hz, 1H), 5.22 (dq, *J* = 8.5, 1.2 Hz, 1H), 4.81 (s, 1H), 4.72 (s, 1H), 4.68 (td, *J* = 9.3, 3.4 Hz, 1H), 4.45 (ddd, *J* = 6.8, 4.2, 2.9 Hz, 1H), 4.37 (dt, *J* = 8.7, 6.4 Hz, 1H), 4.28 – 4.23 (m, 1H), 4.21 – 4.17 (m, 2H), 4.14 – 4.09 (m, 2H), 4.08 – 4.03 (m, 2H), 4.02 – 3.99 (m, 2H), 3.99 – 3.96 (m, 1H), 3.93 – 3.82 (m, 8H), 3.78 – 3.72 (m, 1H), 3.72 – 3.67 (m, 2H), 3.65 – 3.64 (m, 1H), 3.64 – 3.58 (m, 4H), 3.58 – 3.52 (m, 3H), 3.49 (d, *J* = 5.4 Hz, 2H), 3.42 (dd, *J* = 7.4, 4.2 Hz, 1H), 3.28 (dd, *J* = 9.8, 3.1 Hz, 1H), 3.23 (dd, *J* = 9.5, 3.0 Hz, 1H)., 3.02 (dd, *J* = 9.2, 9.2 Hz, 1H), 2.45 – 2.33 (m, 3H), 2.29 – 2.14 (m, 10H), 2.13 – 2.07 (m, 2H), 2.06 – 2.02 (m, 2H), 2.02 – 1.94 (m, 3H), 1.91 – 1.86 (m, 2H), 1.86 – 1.78 (m, 3H), 1.78 – 1.74 (m, 2H), 1.74 – 1.72 (m, 4H), 1.72 – 1.70 (m, 4H), 1.69 – 1.61 (m, 11H), 1.60 – 1.55 (m, 3H), 1.54 – 1.46 (m, 7H), 1.45 – 1.38 (m, 2H). ^{13}C NMR (151 MHz, [D$_4$]-MeOH) δ 143.0, 137.8, 137.0, 136.2, 135.8, 130.9, 129.7, 129.1, 128.7, 111.1, 99.7, 86.1, 84.6, 84.5, 79.0, 77.3, 76.5, 75.6, 75.5, 75.3, 75.0, 74.3, 74.2, 73.8, 73.5, 73.4, 73.4, 73.3 (two peaks), 73.2, 73.1, 72.9, 71.8, 70.9, 70.8, 70.1, 70.0, 69.3, 69.1, 68.8, 68.6, 68.2, 68.2, 68.1, 67.0, 66.1, 62.6, 46.5, 44.9, 44.0, 43.8, 43.2, 42.7, 42.6, 42.3, 41.9, 41.7, 41.2, 41.2, 40.9, 39.6, 39.6, 37.4, 36.9, 36.3, 35.1, 33.8, 32.6, 32.1, 30.1, 27.7, 27.6, 26.2, 17.4, 16.7. IR (film) $\tilde{\nu}$ 3362, 2922, 2853, 1659, 1633, 1423, 1384, 1315, 1072, 980, 894 cm^{-1}. HRMS (ESI) *m*/*z* calcd. for $C_{75}H_{126}O_{30}Na$ [M+Na]$^+$: 1529.82261, found: 1529.82335.

Table S7. Analysis of the NMR data of synthetic **1** corresponding to nominal benthol A; numbering scheme as shown in the Insert

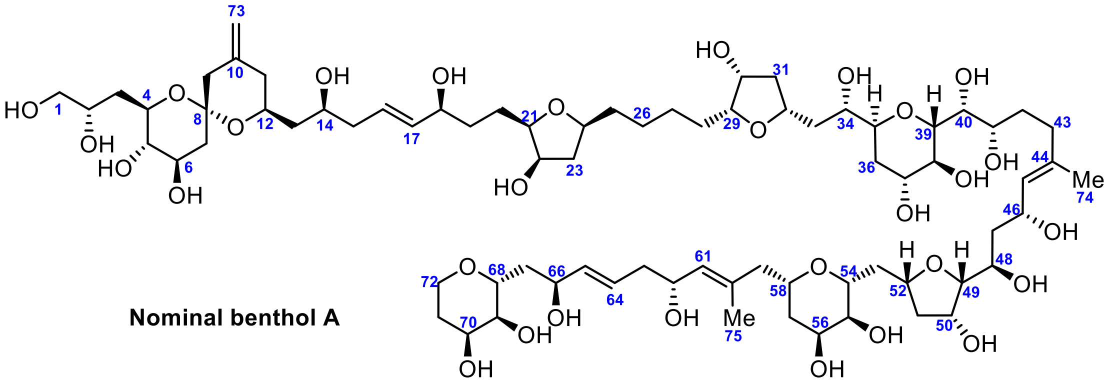

Nominal benthol A

Atom	*δ* (ppm)	*J* (Hz)	COSY	HSQC	HMBC	ROESY
**1 C**	66.95			1	2, 3a, 3b	
**H2**	3.49		2	1	2, 3	3b
**2 C**	72.92			2	1, 3a, 3b, 4	
**H**	3.91		1, 3a, 3b	2	1, 4	4
**3 C**	36.33			3a, 3b	1, 4, 5	
**Ha**	1.46	14.40(3b), 10.10(4)	2, 3b, 4	3	1, 2, 4, 5	5
**Hb**	2.16	2.50(4), 14.40(3a)	2, 3a, 4	3	1, 2, 4	1, 4
**4 C**	73.49			4	2, 3a, 3b, 5	
**H**	3.68	9.20(5), 2.50(3b), 10.10(3a)	3a, 3b, 5	4	2, 3, 5, 6, 8	2, 3b, 6, 12
**5 C**	77.28			5	3a, 4, 6, 7ax, 7eq	
**H**	3.02	9.20(4), 9.20(6)	4, 6	5	3, 4, 6, 7	3a, 7ax
**6 C**	70.10			6	4, 5, 7ax, 7eq	
**H**	3.84	9.20(5), 11.60(7ax), 5.10(7eq)	5, 7ax, 7eq	6	5, 7	4
**7 C**	43.95			7ax, 7eq	5, 6, 9	
**Hax**	1.52	11.60(6), 12.90(7eq)	6, 7eq	7	5, 6, 8, 9	5
**Heq**	2.10	5.10(6), 12.90(7ax)	6, 7ax	7	5, 6, 8	
**8 C**	99.72				4, 7ax, 7eq, 9	
**9 C**	44.85			9	7ax, 11b, 73', 73''	
**H2**	2.25		73', 73''	9	7, 8, 10, 11, 73	
**10 C**	143.01				9, 11a, 11b, 73'	
**11 C**	41.18			11a, 11b	9, 13b, 73', 73''	
**Ha**	1.99		11b, 12, 73', 73''	11	10, 12, 13, 73	
**Hb**	2.22		11a, 12	11	9, 10, 12, 73	73''
**12 C**	68.60			12	11a, 11b, 13a, 13b, 14	
**H**	3.87		11a, 11b, 13a, 13b	12		4
**13 C**	43.76			13a, 13b	11a, 14, 15	
**Ha**	1.49		12, 14	13	12, 14	
**Hb**	1.67		12, 14	13	11, 12	
**14 C**	68.19			14	13a, 15, 16	
**H**	3.91		13a, 13b, 15	14	12, 13, 16	16
**15 C**	42.62			15	16, 17	
**H2**	2.22		14, 16, 17	15	13, 14, 16, 17	
**16 C**	128.72			16	14, 15, 17, 18	

**H**	5.69		15, 17, 18	16	14, 15, 17, 18	14, 18
**17 C**	136.98			17	15, 16, 18, 19	
**H**	5.55		15, 16, 18	17	15, 16, 18, 19	
**18 C**	73.79			18	16, 17, 19, 20a, 20b	
**H**	4.05		16, 17, 19	18	16, 17, 19, 20	16
**19 C**	35.07			19	17, 18, 20a, 20b, 21	
**H2**	1.63		18, 20a, 20b	19	17, 18, 20	
**20 C**	26.16			20a, 20b	18, 19, 21	
**Ha**	1.65		19, 20b, 21	20	18, 19, 21	
**Hb**	1.73		19, 20a, 21	20	18, 19, 21	
**21 C**	84.48			21	20a, 20b, 22, 23a, 23b	
**H**	3.53		20a, 20b, 22	21	19, 20, 22	22, 24
**22 C**	73.19			22	21, 23a, 23b	
**H**	4.19		21, 23a, 23b	22	21, 24	21, 23b
**23 C**	42.33			23a, 23b		
**Ha**	1.49	13.50(23b)	22, 23b, 24	23	21, 22	
**Hb**	2.38	13.50(23a)	22, 23a, 24	23	21, 22, 24, 25	22, 24
**24 C**	79.03			24	22, 23b, 26b	
**H**	3.75		23a, 23b, 25a, 25b	24	26	21, 23b
**25 C**	37.36			25a, 25b	23b, 26b, 27b	
**Ha**	1.57		24, 25b, 26a, 26b	25	27	
**Hb**	1.72		24, 25a, 26a, 26b	25	27	
**26 C**	27.65			26a, 26b	24, 27a, 27b, 28	
**Ha**	1.40		25a, 25b, 26b, 27a, 27b	26	27	
**Hb**	1.49		25a, 25b, 26a, 27a, 27b	26	24, 25, 27	
**27 C**	27.61			27a, 27b	25a, 25b, 26a, 26b, 28, 29	
**Ha**	1.42		26a, 26b, 27b, 28	27	26	
**Hb**	1.48		26a, 26b, 27a, 28	27	25, 26	
**28 C**	30.08			28	29	
**H2**	1.65		27a, 27b, 29	28	26, 27	
**29 C**	84.61			29	30, 31a, 31b	
**H**	3.55		28, 30	29	27, 28, 30	30, 32
**30 C**	73.28			30	29, 31a, 31b	
**H**	4.19		29, 31a, 31b	30	29, 32	29, 31b
**31 C**	42.74			31a, 31b	33a, 33b	
**Ha**	1.56	13.50(31b)	30, 31b, 32	31	29, 30, 32, 33	
**Hb**	2.42	13.50(31a)	30, 31a, 32	31	29, 30, 32, 33	30, 32
**32 C**	75.45			32	30, 31a, 31b, 33a, 33b, 34	
**H**	4.05		31a, 31b, 33a, 33b	32	34	29, 31b
**33 C**	41.24			33a, 33b	31a, 31b, 34, 35'	
**Ha**	1.62		32, 33b, 34	33	31, 32, 35	
**Hb**	1.81		32, 33a, 34	33	31, 32, 35	
**34 C**	69.31			34	32, 35', 36ax, 36eq	

**H**	3.98		33a, 33b, 35'	34	32, 33, 35	37, 39'
**35 C**	75.25			35'	33a, 33b, 34, 36ax, 36eq, 37, 39'	
**35' H**	3.63		34, 36ax, 36eq	35	33, 34, 36, 37, 39	
**36 C**	32.62			36ax, 36eq	35', 38	
**Hax**	1.64		35', 36eq, 37	36	34, 35, 37, 38	
**Heq**	2.04		35', 36ax, 37	36	34, 35, 37, 38	
**37 C**	70.03			37	35', 36ax, 36eq, 38, 39'	
**H**	3.85	6.60(38)	36ax, 36eq, 38	37	35, 38, 39	34
**38 C**	73.40			38	36ax, 36eq, 37, 39', 40	
**H**	3.60	6.60(37), 6.60(39')	37, 39'	38	36, 37, 39, 40	
**39 C**	75.63			39'	35', 37, 38, 40	
**39' H**	3.65	6.60(38), 7.80(40)	38, 40	39	35, 37, 38, 40, 41	34, 41
**40 C**	74.23			40	38, 39', 42b	
**H**	3.90	1.40(41), 7.80(39')	39', 41	40	38, 39, 41, 42	41, 42a
**41 C**	70.84			41	39', 40, 42b, 43a, 43b	
**H**	3.83	1.40(40)	40, 42a, 42b	41	42, 43	39', 40, 42a, 45
**42 C**	32.11			42a, 42b	40, 41, 43a, 43b	
**Ha**	1.66		41, 42b, 43a, 43b	42	43	40, 41
**Hb**	1.75		41, 42a, 43a, 43b	42	40, 41, 43	
**43 C**	36.92			43a, 43b	41, 42a, 42b, 45, 74	
**Ha**	2.10		42a, 42b, 43b	43	41, 42, 44, 45, 74	
**Hb**	2.17		42a, 42b, 43a	43	41, 42, 44, 45, 74	
**44 C**	137.78				43a, 43b, 46, 74	
**45 C**	129.74			45	43a, 43b, 46, 47a, 47b, 74	
**H**	5.30	9.00(46), 1.30(74)	46, 74	45	43, 74	41
**46 C**	66.05			46	47b	
**H**	4.68	9.00(45)	45, 47a, 47b	46	44, 45, 47, 48	47a, 49'
**47 C**	43.2			47a, 47b	46, 49', 74	
**Ha**	1.57	14.40(47b)	46, 47b	47	45, 48, 49	46, 49'
**Hb**	1.88	14.40(47a)	46, 47a	47	45, 46, 48, 49	49'
**48 C**	68.23			48	46, 47a, 47b, 49'	
**H**	4.11		47a, 47b, 49’	48	46, 47, 49	
**49 C**	86.06			49'	47a, 47b, 50, 51a, 51b	
**49' H**	3.42	4.20(50)	50, 48	49	47, 48, 50	46, 47a, 47b, 50, 51b, 52'
**50 C**	73.43			50	49', 51a, 51b, 52'	
**H**	4.45	4.20(49')	49', 51a, 51b	50	49, 52	49', 51b, 52'
**51 C**	41.70			51a, 51b	53a, 53b	
**Ha**	1.65	13.40(51b)	50, 51b, 52'	51	49, 50, 52, 53	
**Hb**	2.37	13.40(51a)	50, 51a, 52'	51	49, 50, 52, 53	49', 50, 52'

**52 C**	76.49			52'	50, 51a, 51b, 53a, 53b, 54	
**52' H**	4.11		51a, 51b, 53a, 53b	52	50, 54	49', 50, 51b, 54
**53 C**	39.57			53a, 53b	51a, 51b, 55	
**Ha**	1.89	8.80(54), 13.80(53b)	52', 53b, 54	53	51, 52, 54, 55	55
**Hb**	1.99	13.80(53a), 3.00(54)	52', 53a, 54	53	51, 52, 54, 55	54, 55
**54 C**	74.28			54	52', 53a, 53b, 55, 56	
**H**	3.60	8.80(53a), 9.60(55), 3.00(53b)	53a, 53b, 55	54	52, 55, 56, 58	52', 53b
**55 C**	73.10			55	53a, 53b, 54, 56, 57ax, 57eq	
**H**	3.28	3.10(56), 9.60(54)	54, 56	55	53, 54, 56, 57	53a, 53b, 56, 57ax
**56 C**	68.81			56	54, 55, 57eq	
**H**	4.01	3.10(55)	55, 57ax, 57eq	56	54, 55, 57, 58	55
**57 C**	39.59			57ax, 57eq	55, 56, 59a, 59b	
**Hax**	1.52		56, 57eq, 58	57	55, 58	55
**Heq**	1.82		56, 57ax, 58	57	55, 56	
**58 C**	70.92			58	54, 56, 57ax, 59a, 59b	
**H**	3.86		57ax, 57eq, 59a, 59b	58		
**59 C**	46.54			59a, 59b	61, 75	
**Ha**	2.04		58, 59b, 75	59	57, 58, 60, 61, 75	
**Hb**	2.18		58, 59a	59	57, 58, 60, 61, 75	
**60 C**	135.82				59a, 59b, 62, 75	
**61 C**	130.89			61	59a, 59b, 62, 63a, 63b, 75	
**H**	5.22	8.70(62)	62, 75	61	59, 63, 75	
**62 C**	69.10			62	63a, 63b, 64, 75	
**H**	4.37	8.70(61)	61, 63a, 63b	62	60, 61, 63, 64	75
**63 C**	41.89			63a, 63b	61, 62, 64, 65, 75	
**Ha**	2.20	7.10(64), 14.00(63b)	62, 63b, 64, 65	63	61, 62, 64, 65	
**Hb**	2.27	14.00(63a), 7.10(64)	62, 63a, 64, 65	63	61, 62, 64, 65	
**64 C**	129.08			64	62, 63a, 63b, 65, 66	
**H**	5.65	15.30(65), 7.10(63a), 7.10(63b)	63a, 63b, 65, 66	64	62, 63, 65, 66	66
**65 C**	136.22			65	63a, 63b, 64, 66, 67a, 67b	
**H**	5.51	15.30(64), 7.40(66)	63a, 63b, 64, 66	65	63, 64, 66, 67	68
**66 C**	71.78			66	64, 65, 67a, 67b, 68	
**H**	4.25	7.40(65), 7.90(67b), 5.70(67a)	64, 65, 67a, 67b	66	64, 65, 67, 68	64, 67a, 68
**67 C**	40.92			67a, 67b	65, 66, 68, 69	
**Ha**	1.63	5.70(66), 13.90(67b), 9.70(68)	66, 67b, 68	67	65, 66, 68, 69	66, 69
**Hb**	1.96	7.90(66), 2.30(68), 13.90(67a)	66, 67a, 68	67	65, 66, 68, 69	68, 69
**68 C**	74.98			68	66, 67a, 67b, 69, 70, 72a, 72b	

**H**	3.57	9.50(69), 2.30(67b), 9.70(67a)	67a, 67b, 69	68	66, 67, 69, 70, 72	65, 66, 67b
**69 C**	73.28			69	67a, 67b, 68, 70, 71a	
**H**	3.23	9.50(68), 3.00(70)	68, 70	69	67, 68, 70	67a, 67b, 70, 71b
**70 C**	68.08			70	68, 69, 71a, 72a, 72b	
**H**	4.00	3.00(69)	69, 71a, 71b	70	68, 69, 71, 72	69
**71 C**	33.80			71a, 71b	70, 72a	
**Ha**	1.75		70, 71b, 72a, 72b	71	69, 70	
**Hb**	1.84		70, 71a, 72a, 72b	71	72	69
**72 C**	62.59			72a, 72b	68, 70, 71b	
**Ha**	3.61		71a, 71b, 72b	72	68, 70, 71	
**Hb**	3.69		71a, 71b, 72a	72	68, 70	
**73 C**	111.13			73', 73''	9, 11a, 11b	
**H'**	4.72		9, 11a, 73''	73	9, 10, 11	
**H''**	4.81		9, 11a, 73'	73	9, 11	11b
**74 C**	16.70			74	43a, 43b, 45	
**H3**	1.73	1.30(45)	45	74	43, 44, 45, 47	
**75 C**	17.43			75	59a, 59b, 61	
**H3**	1.71		59a, 61	75	59, 60, 61, 62, 63	62

Table S8. Comparison of NMR data of authentic benthol A and synthetic nominal benthol A (**1**)

Atom	δ (ppm, ^{1}H-NMR in CD_3OD)		Δδ	δ (ppm, ^{13}C-NMR in CD_3OD)		Δδ
	benthol A	**1**		benthol A	**1**	
**1a**	3.48	3.49	-0.01	67.0	67.0	0
**1b**	3.48	3.49	-0.01			
**2**	3.90	3.91	-0.01	73.0	72.9	0.1
**3a**	1.46	1.46	0	36.4	36.3	0.1
**3b**	2.15	2.16	-0.01			
**4**	3.67	3.68	-0.01	73.54	73.49	0.05
**5**	3.01	3.02	-0.01	77.3	77.3	0
**6**	3.83	3.84	-0.01	70.1	70.1	0
**7ax**	1.50	1.52	-0.02	44.0	44.0	0
**7eq**	2.10	2.10	0			
**8**	-	-	-	99.8	99.7	0.1
**9a**	2.24	2.25	-0.01	44.9	44.9	0
**9b**	2.24	2.25	-0.01			
**10**	-	-	-	143.1	143.0	0.1
**11a**	1.98	1.99	-0.01	41.2	41.2	0
**11b**	2.22	2.22	0			
**12**	3.87	3.87	0	68.7	68.6	0.1
**13a**	1.49[a]	1.49	0	43.8	43.8	0
**13b**	1.67	1.67	0			
**14**	3.90	3.91	-0.01	68.3	68.2	0.1
**15a**	2.21	2.22	-0.01	42.66	42.62	0.04
**15b**	2.21	2.22	-0.01			
**16**	5.69	5.69	0	128.8	128.7	0.1
**17**	5.54	5.55	-0.01	137.0	137.0	0
**18**	4.05	4.05	0	73.8	73.8	0
**19a**	1.63	1.63	0	35.1	35.1	0
**19b**	1.63	1.63	0			

**20a**	1.64	1.65	-0.01	26.2	26.2	0
**20b**	1.71	1.73	-0.02			
**21**	3.53	3.53	0	84.5	84.5	0
**22**	4.18	4.19	-0.01	73.22	73.19	0.03
**23a**	1.49	1.49	0	42.4	42.3	0.1
**23b**	2.38	2.38	0			
**24**	3.75	3.75	0	79.1	79.0	0.1
**25a**	1.56	1.57	-0.01	37.4	37.4	0
**25b**	1.71	1.72	-0.01			
**26a**	1.38	1.40	-0.02	27.68	27.65	0.03
**26b**	1.48	1.49	-0.01			
**27a**	1.40	1.42	-0.02	27.64	27.61	0.03
**27b**	1.48	1.48	0			
**28a**	1.64	1.65	-0.01	30.2	30.1	0.1
**28b**	1.64	1.65	-0.01			
**29**	3.56	3.55	0.01	84.7	84.6	0.1
**30**	4.18	4.19	-0.01	73.3	73.3	0
**31a**	1.55	1.56	-0.01	42.7	42.7	0
**31b**	2.42	2.42	0			
**32**	4.08	4.05	0.03	75.4	75.5	-0.1
**33a**	1.52	1.62	-0.1	41.73	41.24	0.49
**33b**	1.82	1.81	0.01			
**34**	4.18	3.98	0.2	67.1	69.3	-2.2
**35**	3.62	3.63	-0.01	77.8	75.3	2.5
**36ax**	1.72	1.64	0.08	34.2	32.6	1.6
**36eq**	2.03	2.04	-0.01			
**37**	3.76	3.85	-0.09	71.1	70.0	1.1
**38**	3.49	3.60	-0.11	72.8	73.4	-0.6
**39**	3.69	3.65	0.04	73.22	75.63	-2.41
**40**	3.62	3.90	-0.28	73.1	74.2	-1.1
**41**	3.63	3.83	-0.2	72.5	70.8	1.7
**42a**	1.50	1.66	-0.16	33.5	32.1	1.4
**42b**	1.99	1.75	0.24			
**43a**	2.11	2.10	0.01	36.8	36.9	-0.1
**43b**	2.27	2.17	0.1			
**44**	-	-	-	138.1	137.8	0.3
**45**	5.29	5.30	-0.01	129.6	129.7	-0.1
**46**	4.67	4.68	-0.01	66.2	66.1	0.1
**47a**	1.58	1.57	0.01	43.3	43.2	0.1
**47b**	1.88	1.88	0			
**48**	4.10	4.11	-0.01	68.2	68.2	0
**49**	3.42	3.42	0	86.1	86.1	0
**50**	4.45	4.45	0	73.49	73.43	0.06
**51a**	1.64	1.65	-0.01	41.73	41.70	0.03
**51b**	2.36	2.37	-0.01			
**52**	4.11	4.11	0	76.5	76.5	0
**53a**	1.88	1.89	-0.01	39.61	39.57	0.04
**53b**	1.99	1.99	0			
**54**	3.59	3.60	-0.01	74.3	74.3	0
**55**	3.27	3.28	-0.01	73.15	73.10	0.05
**56**	4.01	4.01	0	68.9	68.8	0.1
**57ax**	1.51	1.52	-0.01	39.63	39.59	0.04
**57eq**	1.82	1.82	0			
**58**	3.86	3.86	0	70.96	70.92	0.04
**59a**	2.04	2.04	0	46.6	46.5	0.1
**59b**	2.18	2.18	0			
**60**	-	-	-	135.9	135.8	0.1
**61**	5.21	5.22	-0.01	130.9	130.9	0
**62**	4.37	4.37	0	69.2	69.1	0.1

**63a**	2.20	2.20	0	41.9	41.9	0
**63b**	2.26	2.27	-0.01			
**64**	5.65	5.65	0	129.1	129.1	0
**65**	5.50	5.51	-0.01	136.3	136.2	0.1
**66**	4.25	4.25	0	71.8	71.8	0
**67a**	1.63	1.63	0	41.0	40.9	0.1
**67b**	1.96	1.96	0			
**68**	3.56	3.57	-0.01	75.0	75.0	0
**69**	3.22	3.23	-0.01	73.33	73.28	0.05
**70**	4.00	4.00	0	68.1	68.1	0
**71a**	1.74	1.75	-0.01	33.8	33.8	0
**71b**	1.83	1.84	-0.01			
**72a**	3.61	3.61	0	62.6	62.6	0
**72b**	3.69	3.69	0			
**73a**	4.72	4.72	0	111.2	111.1	0.1
**73b**	4.81	4.81	0			
**74**	1.72	1.73	-0.01	16.8	16.7	0.1
**75**	1.70	1.71	-0.01	17.5	17.4	0.1

[a] The chemical shift of H-13a was extracted from the copy of the ^{1}H NMR spectrum (figure S15) of natural benthol A contained in the supporting information provided by the isolation team.[7]

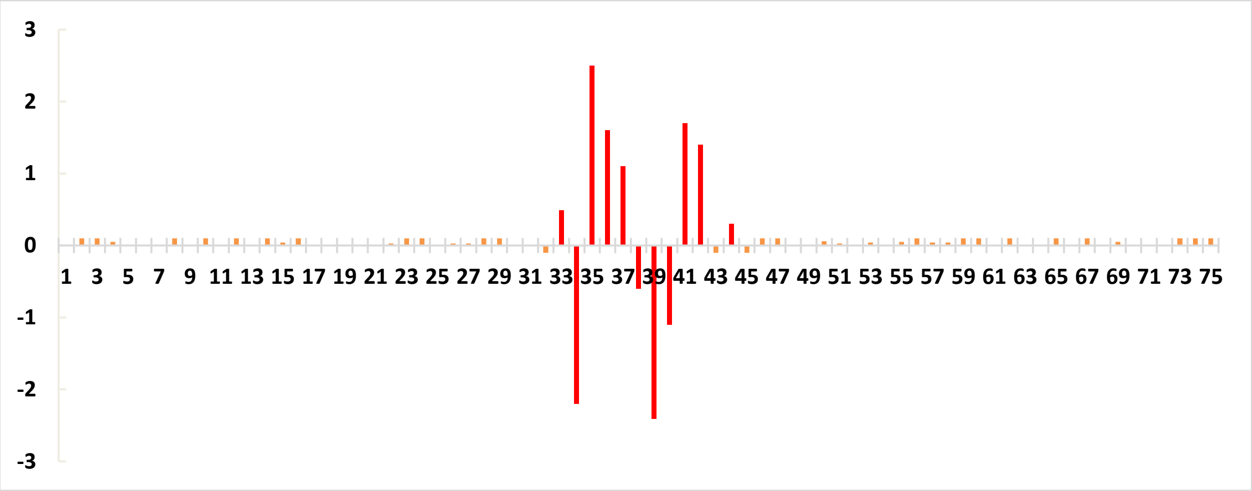

Figure S2. Graphical representation of the ^{13}C NMR shift differences ($\Delta\delta_C$) between authentic benthol A and synthetic **1**.

Completion of the Total Synthesis of Actual Benthol A

Compound 40-*epi*-39. An oven-dried Schlenk tube was charged with 9-BBN-dimer (55.0 mg, 225.0 µmol) and THF (0.2 mL). The solution was stirred at 0 °C (bath temperature) while a solution of alkene **40-*epi*-38** (56.2 mg, 45.0 µmol) in THF (0.8 mL) was added dropwise. The mixture was stirred at room temperature for 1.5 h. The resulting solution of the corresponding alkyl-9-BBN derivative was directly used in the cross-coupling reaction.

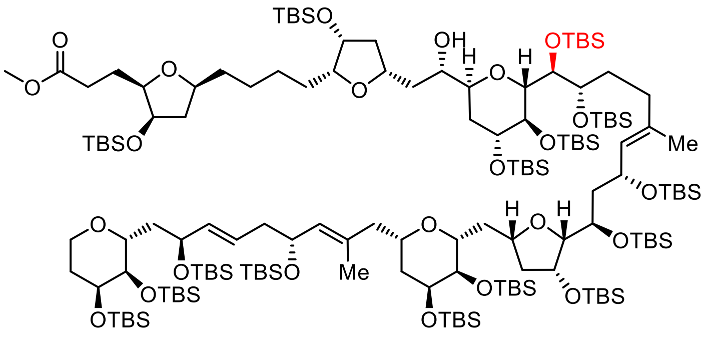

NaOH solution (0.50 M in water, 0.45 mL, 225.0 µmol) was added to the solution of the alkyl-9-BBN derivative at room temperature and the resulting mixture was stirred vigorously for 30 min. Alkenyl iodide **30** (40.0 mg, 22.5 µmol), THF (1.0 mL) and Pd(dppf)Cl_2 (1.65 mg, 2.25 µmol) were then successively added and the resulting solution stirred at room temperature for 2 h. The mixture was diluted with sat. aq. NH_4Cl solution (10 mL) and then extracted with EtOAc (3 x 10 mL). The combined organic layers were washed with brine (10 mL), dried over Na_2SO_4, and concentrated under reduced pressure. The residue was purified by flash chromatography (silica; toluene/*tert*-butyl methyl ether, 10:1) to give the title compound as a pale-yellow oil (35.8 mg, 55%, 66% brsm) as well as a fraction consisting of recovered alkenyl iodide **30** (6.7 mg, 17%). $[\alpha]_D^{20}$ = +12.8° (*c* = 1.02, CH_2Cl_2). ^{1}H NMR (600 MHz, C_6D_6) δ 5.97 (dddd, *J* = 15.3, 8.3, 5.9, 1.1 Hz, 1H), 5.82 (ddt, *J* = 15.3, 6.7, 1.3 Hz, 1H), 5.58 – 5.51 (m, 1H), 5.45 – 5.39 (m, 1H), 5.09 (ddd, *J* = 9.1, 7.5, 6.1 Hz, 1H), 4.71 (ddd, *J* = 9.9, 6.7, 4.0 Hz, 1H), 4.58 (ddd, *J* = 8.5, 7.1, 5.5 Hz, 1H), 4.48 (dd, *J* = 6.1, 6.0 Hz, 1H), 4.44 (ddd, *J* = 7.0, 4.3, 2.9 Hz, 1H), 4.29 (ddd, *J* = 8.1, 6.7, 6.7 Hz, 1H), 4.27 – 4.23 (m, 2H), 4.23 – 4.18 (m, 2H), 4.15 – 4.08 (m, 2H), 4.04 – 3.96 (m, 5H), 3.94 – 3.90 (m, 3H), 3.87 – 3.82 (m, 2H), 3.71 (ddd, *J* = 7.4, 7.3, 4.9 Hz, 1H), 3.64 – 3.58 (m, 3H), 3.47 (ddd, *J* = 7.7, 5.4, 3.9 Hz, 1H), 3.38 (dd, *J* = 9.0, 2.3 Hz, 1H), 3.36 (s, 3H), 3.35 (dd, *J* = 8.9, 2.5 Hz, 1H), 3.01 (s, 1H), 2.60 – 2.47 (m, 4H), 2.45 – 2.31 (m, 5H), 2.26 – 2.13 (m, 5H), 2.12 – 2.06 (m, 3H), 2.06 – 2.00 (m, 3H), 2.00 – 1.97 (m, 1H), 1.96 (d, *J* = 1.3 Hz, 3H), 1.92 (ddd, *J* = 13.4, 10.7, 4.3 Hz, 1H), 1.88 – 1.82 (m, 4H), 1.82 – 1.77 (m, 4H), 1.74 (ddd, *J* = 13.5, 3.9, 2.1 Hz, 1H), 1.71 – 1.66 (m, 2H), 1.64 – 1.57 (m, 5H), 1.49 – 1.43 (m, 4H), 1.41 – 1.37 (m, 1H), 1.17 (s, 9H), 1.16 (s, 9H), 1.13 (s, 9H), 1.11 (s, 9H), 1.11 (s, 9H), 1.10 (s, 9H), 1.09 (s, 9H), 1.09 – 1.09 (m, 18H), 1.08 (s, 9H), 1.06 (s, 9H), 1.05 (s, 9H), 1.01 (s, 9H), 1.00 (s, 9H), 0.97 (s, 9H), 0.40 – 0.37 (m, 6H), 0.37 – 0.35 (m, 6H), 0.35 – 0.33 (m, 6H), 0.33 – 0.31 (m, 9H), 0.28 (s, 3H), 0.26 – 0.22 (m, 15H), 0.21 – 0.17 (m, 15H), 0.16 – 0.14 (m, 6H), 0.11 (s, 3H), 0.09 (s, 3H), 0.06 – 0.03 (m, 18H). ^{13}C NMR (151 MHz, C_6D_6) δ 173.6, 135.7, 135.3, 133.0, 132.1, 130.2, 127.1, 87.8, 83.5, 81.7, 77.6, 75.3, 75.2, 75.0, 74.8, 74.7, 74.5, 74.2, 73.8, 73.8, 73.4, 73.4, 73.4, 73.3,

73.0, 72.6, 71.7, 71.2, 70.6, 70.3, 69.9, 69.8, 68.8, 67.8, 61.7, 51.0, 46.3, 44.7, 42.6 (two peaks), 42.4, 42.4, 42.1, 40.5, 40.2, 38.7, 37.3, 34.8, 33.2, 31.8, 31.8, 31.1, 30.3, 27.3, 27.2, 27.2, 26.7, 26.7, 26.6 (two peaks), 26.6, 26.5, 26.5, 26.4, 26.3, 26.3, 26.3, 26.3, 26.1, 26.1, 25.9, 19.5, 18.8, 18.7, 18.7, 18.6, 18.6 (three peaks), 18.5, 18.5 (two peaks), 18.5, 18.4, 18.4, 18.3, 18.0, 17.7, –1.1, –2.2, –2.3, –2.7 (two peaks), –2.8, –3.3, –3.3 (two peaks), –3.5, –3.6, –3.6, –3.8 (two peaks), –3.8, –4.0, –4.0, –4.0, –4.1, –4.2, –4.3, –4.3, –4.3, –4.3, –4.3, –4.4, –4.4, –4.5, –4.9, –4.9. IR (film) $\tilde{\nu}$ 2953, 2928, 2894, 2856, 1743, 1472, 1462, 1361, 1252, 1082, 1005, 940, 833, 773 cm^{-1}. HRMS (ESI) *m/z* calcd. for $C_{148}H_{308}O_{24}Si_{15}Na$ $[M+Na]^+$: 2912.93118, found: 2912.93051.

Compound 40-*epi*-40. An oven-dried Schlenk tube was charged with methyl ester **40-*epi*-39** (15.3 mg, 5.3 µmol) and toluene (0.5 mL). The solution was vigorously stirred at room temperature while freshly prepared tris(*p*-tolylthio)aluminum in solid form was added in small portions until the starting methyl ester was fully consumed (ca. 30 min, TLC control). At this point, the reaction was immediately quenched with sat. Rochelle salt solution (5 mL). The resulting mixture was stirred vigorously at room temperature for 30 min before the aqueous phase was extracted with EtOAc (3 x 10 mL). The combined organic layers were dried over Na_2SO_4 and concentrated under reduced pressure, and the residue was purified by flash chromatography (silica, hexane/EtOAc, 20:1 to 10:1) to give the title compound as a pale-yellow oil (12.2 mg, 77%). $[\alpha]_D^{20}$ = +14.2° (*c* = 0.88, CH_2Cl_2). ^1H NMR (600 MHz, C_6D_6) δ 7.37 – 7.33 (m, 2H), 6.90 – 6.87 (m, 2H), 5.97 (dddd, *J* = 15.3, 8.3, 5.9, 1.0 Hz, 1H), 5.83 (ddt, *J* = 15.4, 6.7, 1.3 Hz, 1H), 5.57 – 5.52 (m, 1H), 5.42 (ddd, *J* = 8.5, 1.2, 1.2 Hz, 1H), 5.14 – 5.05 (m, 1H), 4.71 (ddd, *J* = 10.0, 6.4, 3.5 Hz, 1H), 4.59 (ddd, *J* = 8.5, 7.1, 5.5 Hz, 1H), 4.48 (dd, *J* = 6.2, 6.0 Hz, 1H), 4.44 (ddd, *J* = 7.0, 4.3, 2.9 Hz, 1H), 4.33 – 4.28 (m, 1H), 4.28 – 4.18 (m, 4H), 4.15 – 4.08 (m, 2H), 4.05 – 3.97 (m, 4H), 3.97 – 3.91 (m, 4H), 3.88 – 3.82 (m, 2H), 3.71 (ddd, *J* = 7.3, 7.2, 5.0 Hz, 1H), 3.65 – 3.58 (m, 2H), 3.56 (dt, *J* = 8.8, 4.5 Hz, 1H), 3.48 (ddd, *J* = 7.6, 5.4, 3.9 Hz, 1H), 3.39 (dd, *J* = 9.0, 2.3 Hz, 1H), 3.35 (dd, *J* = 9.0, 2.4 Hz, 1H), 3.01 (s, 1H), 2.92 – 2.80 (m, 2H), 2.60 – 2.54 (m, 1H), 2.51 (td, *J* = 13.8, 4.6 Hz, 1H), 2.46 – 2.30 (m, 5H), 2.26 – 2.13 (m, 5H), 2.13 – 2.06 (m, 4H), 2.06 – 2.01 (m, 2H), 2.00 – 1.95 (m, 7H), 1.94 – 1.90 (m, 1H), 1.90 – 1.87 (m, 1H), 1.87 – 1.83 (m, 3H), 1.83 – 1.77 (m, 4H), 1.74 (ddd, *J* = 13.5, 4.2, 2.1 Hz, 1H), 1.72 – 1.66 (m, 2H), 1.65 – 1.56 (m, 5H), 1.49 – 1.42 (m, 4H), 1.41 – 1.37 (m, 1H), 1.17 (s, 9H), 1.16 (s, 9H), 1.13 (s, 9H), 1.12 (s, 9H), 1.11 (s, 9H), 1.10 (s, 9H), 1.10 (s, 9H), 1.10 – 1.08 (m, 27H), 1.06 (s, 9H), 1.05 (s, 9H), 1.02 – 0.99 (m, 18H), 0.97 (s, 9H), 0.39 (s, 6H), 0.38 – 0.36 (m, 6H), 0.35 (s, 3H), 0.34 (s, 3H), 0.33 – 0.32 (m, 6H), 0.32 (s, 3H), 0.29 (s, 3H), 0.26 (s, 3H), 0.25 (s, 3H), 0.24 – 0.23 (m, 6H), 0.23 (s, 3H), 0.20 (s, 3H),

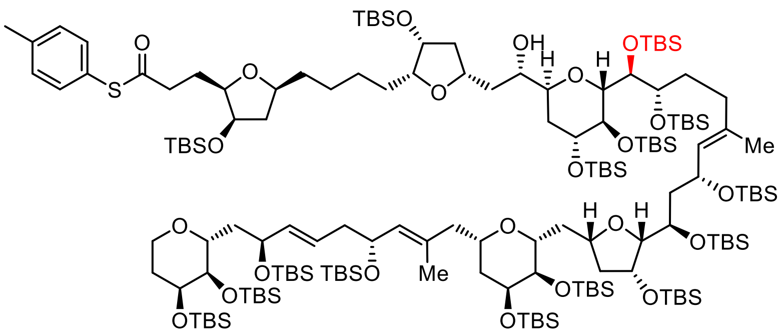

0.20 – 0.19 (m, 6H), 0.19 – 0.17 (m, 6H), 0.16 – 0.14 (m, 6H), 0.11 (s, 3H), 0.09 (s, 3H), 0.06 (s, 3H), 0.05 (s, 3H), 0.05 – 0.04 (m, 6H), 0.03 (s, 3H), 0.02 (s, 3H). ^{13}C NMR (151 MHz, C_6D_6) δ 196.5, 139.3, 135.7, 135.3, 135.0, 133.0, 132.1, 130.2, 130.1, 127.1, 125.5, 87.8, 83.5, 81.4, 77.7, 75.3, 75.2, 75.1, 74.8, 74.7, 74.5, 74.2, 73.8, 73.8, 73.4, 73.4 (two peaks), 73.3, 73.0, 72.6, 71.7, 71.2, 70.6, 70.3, 69.9, 69.8, 68.8, 67.8, 61.7, 46.3, 44.7, 42.6 (two peaks), 42.4, 42.3, 42.1, 40.9, 40.5, 40.2, 38.7, 37.2, 34.8, 33.2, 31.8, 31.8, 30.3, 27.3, 27.2, 27.2, 26.7, 26.7, 26.6 (two peaks), 26.6, 26.5, 26.5, 26.5, 26.3, 26.3, 26.3 (two peaks), 26.3, 26.2, 26.1, 21.1, 19.6, 18.8, 18.7, 18.7, 18.6, 18.6 (three peaks), 18.5 (two peaks), 18.5, 18.5, 18.4, 18.4, 18.3, 18.0, 17.7, –1.1, –2.2, –2.3, –2.7 (two peaks), –2.8, –3.3, –3.3 (two peaks), –3.5, –3.6, –3.6, –3.8, –3.8, –3.8, –4.0, –4.0, –4.0, –4.1, –4.2, –4.3, –4.3, –4.3 (two peaks), –4.3, –4.4, –4.4, –4.5, –4.9, –4.9. IR (film) $\tilde{\nu}$ 2952, 2928, 2894, 2856, 1712, 1472, 1463, 1361, 1252, 1084, 1005, 968, 940, 833, 773 cm^{-1}. HRMS (ESI) *m/z* calcd. for $C_{154}H_{312}O_{23}SSi_{15}Na$ $[M+Na]^+$: 3004.93964, found: 3004.94087.

Compound 40-*epi*-42. *Preparation of the Catalyst Stock Solution:* An oven-dried Schlenk tube was charged with $Pd_2(dba)_3{\cdot}CHCl_3$ (1.2 mg, 1.2 µmol), $P(2\text{-furyl})_3$ (2.2 mg, 9.6 µmol) and degassed THF (400 µL). The mixture was stirred at room temperature until a homogeneous solution had formed (ca. 3 min), which was used in the Liebeskind coupling reaction.

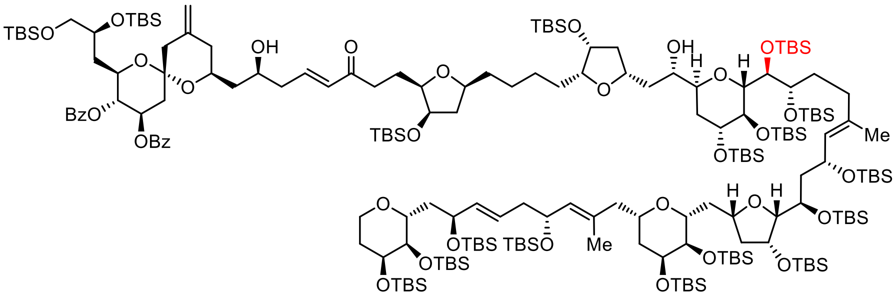

An oven-dried Schlenk tube was charged with thioester **40-*epi*-40** (18.0 mg, 6.0 µmol), copper diphenylphosphinate (5.1 mg, 18.0 µmol) and THF (50 µL). The mixture was stirred at room temperature while an aliquot of the Pd catalyst solution (200 µL) was added, followed by the quick addition of a solution of alkenylstannane **41** (9.8 mg, 9.0 µmol; see the accompanying paper) in hexanes (250 µL). The mixture was stirred at 50 °C (bath temperature) for 3 h, cooled to room temperature and filtered through a short pad of Celite, carefully rinsing with *tert*-butyl methyl ether (5 mL). The combined filtrates were concentrated under reduced pressure and the residue purified by flash chromatography (silica; CH_2Cl_2/acetonitrile, 20:1 to 10:1) to give the title compound as a pale-yellow oil (19.0 mg, 86%). $[\alpha]_D^{20}$ = +18.2° (*c* = 1.02, CH_2Cl_2). ^1H NMR (600 MHz, C_6D_6) δ 8.14 – 8.11 (m, 4H), 7.05 – 6.98 (m, 4H), 6.96 – 6.93 (m, 2H), 6.89 (dt, *J* = 15.9, 7.3 Hz, 1H), 6.16 (dt, *J* = 15.8, 1.4 Hz, 1H), 6.07 (ddd, *J* = 11.5, 9.7, 5.3 Hz, 1H), 6.00 – 5.94 (m, 1H), 5.82 (ddt, *J* = 15.4, 6.7, 1.3 Hz, 1H), 5.59 – 5.51 (m, 2H), 5.44 – 5.39 (m, 1H), 5.14 – 5.04 (m, 1H), 4.90 (dd, *J* = 2.0, 2.0 Hz, 1H), 4.86 (dd, *J* = 2.0, 2.0 Hz, 1H), 4.71 (ddd, *J* = 9.8, 6.3, 4.0 Hz, 1H), 4.58 (ddd, *J* = 8.6, 7.0, 5.5 Hz, 1H), 4.48 (dd,

J = 6.0, 5.9 Hz, 1H), 4.44 (ddd, *J* = 7.0, 4.3, 2.9 Hz, 1H), 4.35 – 4.23 (m, 4H), 4.22 – 4.18 (m, 2H), 4.18 – 4.15 (m, 1H), 4.14 – 4.08 (m, 3H), 4.07 – 3.96 (m, 6H), 3.95 – 3.90 (m, 3H), 3.87 – 3.82 (m, 3H), 3.79 (dd, *J* = 10.7, 2.9 Hz, 1H), 3.77 – 3.72 (m, 1H), 3.68 (dt, *J* = 8.0, 4.7 Hz, 1H), 3.65 – 3.57 (m, 2H), 3.47 (ddd, *J* = 7.5, 5.5, 3.8 Hz, 1H), 3.39 (dd, *J* = 9.1, 2.4 Hz, 1H), 3.35 (dd, *J* = 9.0, 2.4 Hz, 1H), 3.01 (s, 1H), 2.81 – 2.68 (m, 2H), 2.56 (dt, *J* = 13.5, 6.5 Hz, 1H), 2.51 (td, *J* = 13.9, 4.4 Hz, 1H), 2.46 – 2.31 (m, 6H), 2.29 (d, *J* = 4.6 Hz, 1H), 2.26 – 2.13 (m, 8H), 2.13 – 2.07 (m, 4H), 2.07 – 1.99 (m, 6H), 1.96 (d, *J* = 1.3 Hz, 3H), 1.95 – 1.83 (m, 7H), 1.83 – 1.76 (m, 4H), 1.74 (ddd, *J* = 13.5, 4.0, 2.0 Hz, 1H), 1.72 – 1.64 (m, 4H), 1.64 – 1.57 (m, 3H), 1.57 – 1.49 (m, 3H), 1.48 – 1.40 (m, 4H), 1.40 – 1.36 (m, 1H), 1.17 (s, 9H), 1.16 (s, 9H), 1.13 (s, 9H), 1.12 – 1.10 (m, 18H), 1.10 – 1.07 (m, 45H), 1.06 (s, 9H), 1.05 (s, 9H), 1.02 – 0.99 (m, 36H), 0.98 (s, 9H), 0.40 – 0.37 (m, 6H), 0.37 – 0.35 (m, 6H), 0.35 (s, 3H), 0.34 (s, 3H), 0.33 – 0.32 (m, 6H), 0.32 (s, 3H), 0.28 (s, 3H), 0.27 – 0.22 (m, 15H), 0.21 – 0.17 (m, 21H), 0.16 – 0.13 (m, 9H), 0.12 – 0.10 (m, 6H), 0.10 – 0.09 (m, 6H), 0.07 (s, 3H), 0.06 (s, 3H), 0.05 – 0.03 (m, 9H). ^{13}C NMR (151 MHz, C_6D_6) δ 198.6, 166.3, 165.8, 142.0, 141.0, 135.7, 135.3, 133.2, 133.2, 133.1, 133.0, 132.1, 130.5, 130.2 (two peaks), 130.1, 130.0, 128.7, 128.6, 127.1, 111.0, 98.5, 87.8, 83.6, 82.2, 77.7, 75.3, 75.2, 75.1, 74.9, 74.7, 74.4, 74.3 (two peaks), 74.0, 73.8, 73.4 (two peaks), 73.4, 73.3, 73.0, 72.6, 71.7, 71.5, 71.2, 71.0, 70.6, 70.3, 69.9, 69.8, 68.8, 68.3, 68.1, 67.8, 67.6, 66.7, 61.7, 46.3, 44.7, 43.6, 43.0, 42.6 (two peaks), 42.4, 42.4, 42.1, 41.4, 40.9, 40.5, 40.2, 40.0, 38.7, 37.5, 37.4, 37.2, 34.8, 33.2, 31.8, 31.8, 30.3, 27.3, 27.3, 27.2, 26.7, 26.7, 26.6 (two peaks), 26.6, 26.5, 26.5, 26.5, 26.4, 26.4, 26.3, 26.3, 26.3, 26.3, 26.2, 26.2, 24.7, 19.6, 18.8, 18.7 (two peaks), 18.7, 18.6, 18.6, 18.6 (three peaks), 18.5 (three peaks), 18.5, 18.4, 18.4, 18.4, 18.0, 17.7, –1.1, –2.2, –2.3, –2.7 (two peaks), –2.8, –3.3, –3.3 (two peaks), –3.5, –3.6, –3.6, –3.8, –3.8, –3.8, –4.0, –4.0, –4.0, –4.1, –4.1, –4.2, –4.3, –4.3 (two peaks), –4.3, –4.3, –4.4, –4.4, –4.4, –4.5, –4.8, –4.9, –4.9, –4.9. IR (film) $\tilde{\nu}$ 3536, 2953, 2929, 2895, 2856, 1729, 1677, 1472, 1361, 1254, 1093, 1005, 940, 835, 774 cm^{-1}. HRMS (ESI) *m/z* calcd. for $C_{191}H_{370}O_{32}Si_{17}Na_2$ $[M+Na]^{2+}$: 1849.15936, found: 1849.15909.

Compound 40-*epi*-43. *Preparation of the CBS-catalyst:* An oven-dried Schlenk tube was charged with (*R*)-(+)-2-methyl-CBS-oxazaborolidine (4.3 mg, 15.6 µmol) and toluene (62.0 µL). The solution was sonicated for 20 min to give a white suspension that was used for the reduction.

A Schlenk flask was charged with compound **40-*epi*-42** (19.0 mg, 5.2 µmol) and toluene (200.0 µL). The resulting solution was stirred at –78 °C (bath temperature) while the above mixture comprising the (*R*)-(+)-2-methyl-CBS-oxazaborolidine (0.25 M in toluene, 62.0 µL, 15.6 µmol) was added. Stirring was continued at –78 °C for 30 min before catecholborane (1.0 M in THF, 31.2 µL, 31.2 µmol) was added dropwise. The mixture was allowed to warm slowly to –20 °C over the course of 4 h. The reaction was

quenched with MeOH (0.5 mL), the mixture was diluted with sat. aq. NH_4Cl solution (5 mL) and extracted with EtOAc (3 x 10 mL). The combined organic layers were washed with sat. aq. Na_2CO_3 solution until the aqueous layer became colorless, followed by washing with brine (20 mL); it was dried over Na_2SO_4 and concentrated under reduced pressure, and the residue was purified by flash chromatography (silica; hexane/EtOAc, 10:1 to 3:1) to give the title compound as a pale-yellow oil (17.7

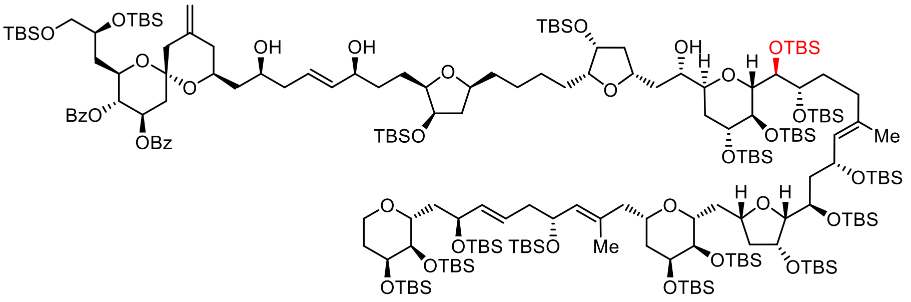

mg, 93%). $[\alpha]_D^{20} = +17.2°$ (c = 0.88, CH_2Cl_2). ^1H NMR (600 MHz, C_6D_6) δ 8.15 – 8.07 (m, 4H), 7.03 – 6.92 (m, 6H), 6.11 (ddd, J = 11.5, 9.7, 5.3 Hz, 1H), 5.97 (dddd, J = 15.3, 8.3, 5.8, 1.1 Hz, 1H), 5.83 (ddt, J = 15.3, 6.7, 1.3 Hz, 1H), 5.74 – 5.64 (m, 2H), 5.59 (dd, J = 9.8, 9.8 Hz, 1H), 5.56 – 5.52 (m, 1H), 5.44 – 5.40 (m, 1H), 5.12 – 5.06 (m, 1H), 4.91 (dd, J = 2.1, 2.1 Hz, 1H), 4.88 (dd, J = 1.7, 1.7 Hz, 1H), 4.71 (ddd, J = 9.8, 6.3, 3.5 Hz, 1H), 4.59 (ddd, J = 8.5, 7.0, 5.5 Hz, 1H), 4.48 (dd, J = 6.1, 6.0 Hz, 1H), 4.44 (ddd, J = 7.0, 4.3, 2.8 Hz, 1H), 4.36 – 4.28 (m, 2H), 4.28 – 4.23 (m, 2H), 4.23 – 4.14 (m, 5H), 4.14 – 4.09 (m, 2H), 4.07 (ddt, J = 11.9, 9.4, 2.6 Hz, 1H), 4.05 – 3.95 (m, 5H), 3.94 – 3.90 (m, 3H), 3.90 – 3.82 (m, 4H), 3.78 – 3.71 (m, 1H), 3.67 – 3.58 (m, 2H), 3.57 – 3.51 (m, 1H), 3.47 (ddd, J = 7.6, 5.4, 3.8 Hz, 1H), 3.39 (dd, J = 9.0, 2.3 Hz, 1H), 3.35 (dd, J = 8.9, 2.3 Hz, 1H), 3.06 – 3.01 (m, 1H), 2.60 – 2.54 (m, 1H), 2.50 (dd, J = 13.5, 4.2 Hz, 1H), 2.47 – 2.32 (m, 6H), 2.29 (ddd, J = 14.4, 9.2, 1.6 Hz, 1H), 2.26 – 2.13 (m, 9H), 2.12 – 2.08 (m, 3H), 2.08 – 1.95 (m, 10H), 1.95 – 1.90 (m, 2H), 1.90 – 1.83 (m, 6H), 1.83 – 1.77 (m, 5H), 1.74 (ddd, J = 13.5, 4.0, 2.0 Hz, 1H), 1.72 – 1.58 (m, 8H), 1.58 – 1.49 (m, 3H), 1.49 – 1.44 (m, 2H), 1.44 – 1.41 (m, 1H), 1.41 – 1.37 (m, 1H), 1.19 – 1.15 (m, 18H), 1.13 (s, 9H), 1.13 – 1.11 (m, 18H), 1.11 – 1.06 (m, 45H), 1.06 – 1.03 (m, 18H), 1.03 – 1.01 (m, 18H), 1.01 – 0.99 (m, 18H), 0.98 (s, 9H), 0.40 – 0.38 (m, 6H), 0.38 – 0.33 (m, 12H), 0.33 – 0.30 (m, 9H), 0.29 (s, 3H), 0.28 – 0.21 (m, 21H), 0.21 – 0.17 (m, 15H), 0.17 – 0.14 (m, 9H), 0.13 (s, 3H), 0.11 (s, 3H), 0.10 – 0.07 (m, 6H), 0.07 – 0.06 (m, 6H), 0.06 – 0.03 (m, 9H). ^{13}C NMR (151 MHz, C_6D_6) δ 166.3, 166.0, 141.2, 138.0, 135.7, 135.3, 133.1, 133.0, 133.0, 132.1, 130.5, 130.2, 130.2, 130.2, 130.0, 128.6, 128.6, 127.1, 126.5, 110.9, 98.4, 87.8, 83.6, 83.5, 77.9, 75.3, 75.2, 75.1, 74.8, 74.7, 74.4, 74.4, 74.3, 74.0, 73.8, 73.4, 73.4 (two peaks), 73.3, 73.0, 72.7, 72.6, 71.7, 71.3, 71.2, 71.1, 70.6, 70.3, 69.9, 69.9, 68.8, 68.4, 67.8, 67.8 (two peaks), 67.3, 61.7, 46.3, 44.7, 43.7, 43.0, 42.6 (two peaks), 42.5, 42.4, 42.1, 41.8, 40.9, 40.5, 40.2, 40.2, 38.7, 37.3, 37.3, 35.1, 34.8, 33.2, 31.8 (two peaks), 30.2, 27.3, 27.3, 27.2, 26.7, 26.7, 26.6 (two peaks), 26.6, 26.5, 26.5 (two peaks), 26.4 (two peaks), 26.4, 26.3, 26.3, 26.3, 26.3, 26.2 (two peaks), 19.6, 18.8, 18.7 (two peaks), 18.7, 18.6, 18.6, 18.6 (three peaks), 18.5 (two peaks), 18.5, 18.5, 18.4 (two peaks), 18.4, 18.0, 17.7, −1.1, −2.2, −2.3, −2.7 (two peaks), −2.8, −3.3, −3.3 (two peaks), −3.5, −3.6, −3.6, −3.8, −3.8, −3.8, −4.0, −4.0, −4.0, −4.1 (two peaks), −4.2, −4.3, −4.3 (two peaks), −4.3

(two peaks), –4.3, –4.4, –4.4, –4.5, –4.8, –4.8, –4.9 (two peaks). IR (film) $\tilde{\nu}$ 3446, 2953, 2928, 2896, 2856, 1730, 1603, 1472, 1361, 1253, 1088, 1005, 940, 834, 774 cm^{-1}. HRMS (ESI) *m*/*z* calcd. for $C_{191}H_{372}O_{32}Si_{17}Na_2$ $[M+Na]^{2+}$: 1850.16719, found: 1850.16710.

Actual Benthol A (40-*epi*-1, Revised Structure). A Schlenk flask was charged with compound **40-*epi*-43** (17.7 mg, 4.84 µmol), K_2CO_3 (6.7 mg, 48.4 µmol), THF (1.0 mL) and MeOH (1.0 mL). The mixture was stirred at room temperature for 21 h before all volatile materials were removed under reduced pressure. The residue was diluted with brine (10 mL) and the aqueous phase extracted with EtOAc (3 x 10 mL). The combined organic layers were dried over Na_2SO_4 and concentrated under reduced pressure to give a crude product that was used in the next step without further purification.

TBAF (1.0 M in THF, 165.0 µL, 165.0 µmol) was added dropwise at 0 °C to a solution of the crude product in THF (1.0 mL). The resulting mixture was stirred at room temperature for 24 h before it was diluted with EtOAc (10 mL). The mixture was concentrated under reduced pressure. A mixture of hexanes/ CH_2Cl_2 (3:1, 10 mL) was added to the oily residue and the resulting suspension was filtered through a plug of cotton; the solid material was kept for further processing. The cotton plug was rinsed with the same mixed solvent (3 x 2 mL) and the organic filtrates were discarded. The cotton was then rinsed with MeOH (3 x 2 mL). The MeOH filtrates were mixed with the solid that had been insoluble in the mixed solvent and the resulting solution was concentrated under reduced pressure. The washing procedure was repeated until all TBAF by-product was removed. Finally, the remaining highly polar residue was purified by flash chromatography (silica gel 60 cyano, H_2O/MeOH = 9:1) to give the title compound as a white solid (6.68 mg, 92% over two steps). $[\alpha]_D^{25}$ = +22.5° (*c* = 0.48, MeOH) [Lit. $[\alpha]_D^{25}$ = +60.0° (*c* = 0.10, MeOH)].[7] ^{1}H NMR (600 MHz, [D$_4$]-MeOH) δ 5.73 – 5.61 (m, 2H), 5.58 – 5.48 (m, 2H), 5.30 (dq, *J* = 8.6, 1.3 Hz, 1H), 5.22 (dq, *J* = 8.7, 1.2 Hz, 1H), 4.81 (s, 1H), 4.72 (s, 1H), 4.67 (td, *J* = 9.1, 3.6 Hz, 1H), 4.45 (ddd, *J* = 6.9, 4.3, 3.0 Hz, 1H), 4.37 (dt, *J* = 8.6, 6.4 Hz, 1H), 4.28 – 4.22 (m, 1H), 4.22 – 4.15 (m, 3H), 4.15 – 4.03 (m, 4H), 4.03 – 3.99 (m, 2H), 3.93 – 3.81 (m, 5H), 3.79 – 3.73 (m, 2H), 3.72 – 3.66 (m, 3H), 3.66 – 3.52 (m, 8H), 3.51 – 3.47 (m, 3H), 3.42 (dd, *J* = 7.4, 4.2 Hz, 1H), 3.28 (dd, *J* = 9.7, 3.1 Hz, 1H), 3.23 (dd, *J* = 9.4, 3.1 Hz, 1H), 3.02 (dd, *J* = 9.3, 9.3 Hz, 1H), 2.45 – 2.33 (m, 3H), 2.30 – 2.24 (m, 4H), 2.24 – 2.14 (m, 6H), 2.13 – 2.07 (m, 2H), 2.07 – 1.93 (m, 6H), 1.92 – 1.86 (m, 2H), 1.86 – 1.81 (m, 3H), 1.77 – 1.69 (m, 10H), 1.69 – 1.61 (m, 8H), 1.61 – 1.55 (m, 3H), 1.54 – 1.45 (m, 9H), 1.44 – 1.37 (m, 2H). ^{13}C NMR (151 MHz, [D$_4$]-MeOH) δ 143.0, 138.1, 137.0, 136.2, 135.8, 130.9, 129.5, 129.1, 128.7, 111.1, 99.7, 86.1, 84.6, 84.5, 79.0, 77.7, 77.3, 76.5, 75.3, 75.0, 74.3, 73.8, 73.5, 73.5, 73.3, 73.3, 73.2 (two peaks), 73.1, 73.1, 72.9, 72.7, 72.4, 71.8, 71.0, 70.9, 70.1, 69.1, 68.8, 68.6, 68.2, 68.2, 68.1, 67.1, 66.9, 66.1, 62.6, 46.5, 44.8, 44.0, 43.8, 43.2, 42.7, 42.6, 42.3, 41.9, 41.7, 41.7, 41.2, 40.9, 39.6, 39.6, 37.4, 36.8, 36.3, 35.1, 34.1, 33.8, 33.4, 30.1, 27.6, 27.6, 26.2, 17.4, 16.8. IR (film) $\tilde{\nu}$ 3361, 2923, 1656, 1426,

1069, 1024, 983, 892 cm^{-1}. HRMS (ESI) *m/z* calcd. for $C_{75}H_{126}O_{30}Na$ $[M+Na]^+$: 1529.82261, found: 1529.82266.

Table S9. Analysis of the NMR data of synthetic benthol A (**40-*epi*-1**, revised structure); numbering scheme as shown in the Insert

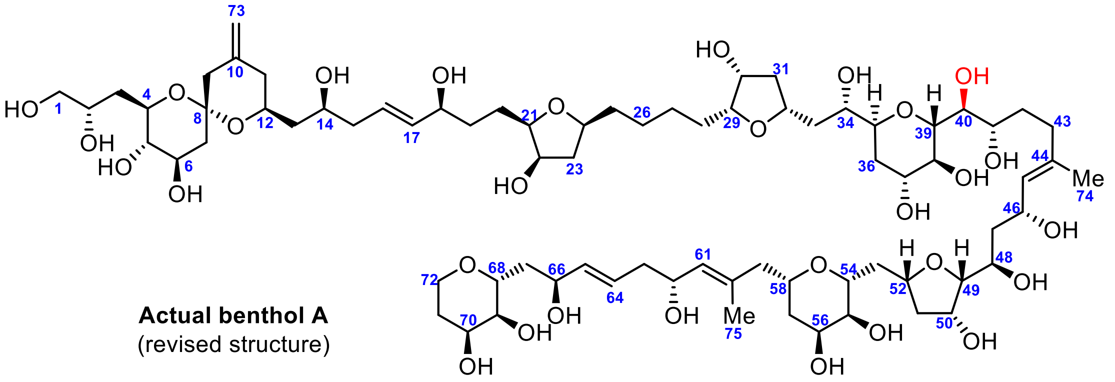

Actual benthol A
(revised structure)

Atom	*δ* (ppm)	*J* (Hz)	COSY	HSQC	HMBC	ROESY
**1 C**	66.94			1	2, 3a, 3b	
**H2**	3.49	5.30(2)	2	1	2, 3	3a, 3b
**2 C**	72.92			2	1, 3a, 3b, 4	
**H**	3.91	5.30(1)	1, 3a, 3b	2	1, 3, 4	4
**3 C**	36.33			3a, 3b	1, 2, 4, 5	
**Ha**	1.47	10.10(4), 14.40(3b)	2, 3b, 4	3	1, 2, 4, 5	1, 5
**Hb**	2.16	14.40(3a), 2.50(4)	2, 3a, 4	3	1, 2, 4, 5	1, 4
**4 C**	73.48			4	2, 3a, 3b, 5, 6	
**H**	3.68	9.20(5), 10.10(3a), 2.50(3b)	3a, 3b, 5	4	2, 3, 5, 6, 8	2, 3b, 6, 12
**5 C**	77.27			5	3a, 3b, 4, 6, 7ax, 7eq	
**H**	3.02	9.20(4), 9.20(6)	4, 6, 7eq	5	3, 4, 6, 7	3a, 7ax
**6 C**	70.10			6	4, 5, 7ax, 7eq	
**H**	3.84	9.20(5), 11.60(7ax), 5.16(7eq)	5, 7ax, 7eq	6	4, 5, 7	4
**7 C**	43.95			7ax, 7eq	5, 6, 9	
**Hax**	1.52	11.60(6), 12.90(7eq)	6, 7eq	7	5, 6, 8, 9	5
**Heq**	2.10	5.16(6), 12.90(7ax)	5, 6, 7ax	7	5, 6, 8	
**8 C**	99.71				4, 7ax, 7eq, 9	
**9 C**	44.84			9	7ax, 11b, 73', 73''	
**H2**	2.25		73', 73''	9	7, 8, 10, 11, 73	73'
**10 C**	143.00				9, 11a, 11b, 73', 73''	
**11 C**	41.18			11a, 11b	9, 13a, 13b, 73', 73''	
**Ha**	1.99		11b, 12, 73', 73''	11	10, 12, 13, 73	
**Hb**	2.22		11a, 12	11	9, 10, 12, 73	73''
**12 C**	68.60			12	11a, 11b, 13a, 13b, 14	
**H**	3.87		11a, 11b, 13a, 13b	12		4
**13 C**	43.75			13a, 13b	11a, 14, 15	
**Ha**	1.49		12, 14	13	11, 12, 14, 15	
**Hb**	1.67		12, 14	13	11, 12, 15	
**14 C**	68.19			14	13a, 15, 16	

**H**	3.91		13a, 13b, 15	14	12, 13, 16	16
**15 C**	42.61			15	13a, 13b, 16, 17	
**H2**	2.22	7.20(16)	14, 16, 17	15	13, 14, 16, 17	17
**16 C**	128.71			16	14, 15, 17, 18	
**H**	5.69	7.20(15), 15.30(17)	15, 17, 18	16	14, 15, 17, 18	14, 18
**17 C**	136.98			17	15, 16, 18, 19	
**H**	5.55	15.30(16)	15, 16, 18	17	15, 16, 18, 19	15
**18 C**	73.78			18	16, 17, 19, 20a, 20b	
**H**	4.05		16, 17, 19	18	16, 17, 19, 20	16
**19 C**	35.06			19	17, 18, 20a, 20b, 21	
**H2**	1.63		18, 20a, 20b	19	17, 18, 20	
**20 C**	26.15			20a, 20b	18, 19, 21	
**Ha**	1.64		19, 20b, 21	20	18, 19, 21	
**Hb**	1.72		19, 20a, 21	20	18, 19, 21	
**21 C**	84.47			21	20a, 20b, 22, 23a, 23b	
**H**	3.54		20a, 20b, 22	21	19, 20, 22	22, 23b, 24
**22 C**	73.18			22	21, 23a, 23b	
**H**	4.19		21, 23a, 23b	22	21, 24	21, 23b
**23 C**	42.31			23a, 23b		
**Ha**	1.50		22, 23b, 24	23	21, 22	
**Hb**	2.38		22, 23a, 24	23	21, 22, 24, 25	21, 22, 24
**24 C**	79.01			24	22, 23b, 26b	
**H**	3.76		23a, 23b, 25a, 25b	24	26	21, 23b
**25 C**	37.35			25a, 25b	23b, 26b, 27b	
**Ha**	1.57		24, 25b, 26a, 26b	25	27	
**Hb**	1.72		24, 25a, 26a, 26b	25	27	
**26 C**	27.63			26a, 26b	24, 27a, 27b, 28	
**Ha**	1.40		25a, 25b, 26b, 27a, 27b	26	27, 28	
**Hb**	1.49		25a, 25b, 26a, 27a, 27b	26	24, 25, 27	
**27 C**	27.59			27a, 27b	25a, 25b, 26a, 26b, 28, 29	
**Ha**	1.42		26a, 26b, 27b, 28	27	26	
**Hb**	1.49		26a, 26b, 27a, 28	27	25, 26	
**28 C**	30.12			28	26a, 29	
**H2**	1.65		27a, 27b, 29	28	26, 27, 29	
**29 C**	84.64			29	28, 30, 31a, 31b	
**H**	3.56		28, 30	29	27, 28, 30, 31	30, 32
**30 C**	73.25			30	29, 31a, 31b	
**H**	4.19		29, 31a, 31b	30	29, 32	29, 31b
**31 C**	42.65			31a, 31b	29, 33a, 33b	
**Ha**	1.55	13.50(31b)	30, 31b, 32	31	29, 30, 32, 33	
**Hb**	2.42	13.50(31a)	30, 31a, 32	31	29, 30, 32, 33	30, 32
**32 C**	75.34			32	30, 31a, 31b, 33a, 33b, 34	
**H**	4.08		31a, 31b, 33a, 33b	32	34	29, 31b, 34
**33 C**	41.67			33a, 33b	31a, 31b, 34, 35'	
**Ha**	1.52		32, 33b, 34	33	31, 32, 34, 35	
**Hb**	1.83		32, 33a, 34	33	31, 32, 35	
**34 C**	67.1			34	32, 33a, 35', 36ax	
**H**	4.18		33a, 33b, 35'	34	32, 33, 35, 36	32, 37, 39'
**35 C**	77.71			35'	33a, 33b, 34, 36ax, 36eq, 37, 39'	
**35' H**	3.62		34, 36ax, 36eq	35	33, 34, 36, 37, 39	36eq

**36 C**	34.11			36ax, 36eq	34, 35', 37, 38	
**Hax**	1.71	13.40(36eq), 11.40(37)	35', 36eq, 37	36	34, 35, 37, 38	38
**Heq**	2.03	5.10(37), 13.40(36ax)	35', 36ax, 37	36	35, 37, 38	35'
**37 C**	71.04			37	35', 36ax, 36eq, 38, 39'	
**H**	3.76	5.10(36eq), 11.40(36ax)	36ax, 36eq, 38	37	35, 36, 38, 39	34, 39'
**38 C**	72.73			38	36ax, 36eq, 37, 39', 40	
**H**	3.49	9.60(39')	37, 39'	38	36, 37, 39, 40	36ax
**39 C**	73.18			39'	35', 37, 38, 40	
**39' H**	3.70	9.60(38), 1.30(40)	38, 40	39	35, 37, 38	34, 37, 41
**40 C**	73.05			40	38, 41, 42b	
**H**	3.63	1.30(39')	39', 41	40	38, 39, 41, 42	41, 42a
**41 C**	72.43			41	40, 42a, 42b, 43a, 43b	
**H**	3.63		40, 42a, 42b	41	40, 42, 43	39', 40, 42a, 45
**42 C**	33.41			42a, 42b	40, 41, 43a, 43b	
**Ha**	1.51		41, 42b, 43a, 43b	42	41, 43, 44	40, 41
**Hb**	2.00		41, 42a, 43a, 43b	42	40, 41, 43	
**43 C**	36.76			43a, 43b	41, 42a, 42b, 45, 74	
**Ha**	2.11		42a, 42b, 43b, 45	43	41, 42, 44, 45, 74	
**Hb**	2.27		42a, 42b, 43a, 45, 74	43	41, 42, 44, 45, 74	
**44 C**	138.07				42a, 43a, 43b, 46, 74	
**45 C**	129.53			45	43a, 43b, 46, 47a, 47b, 74	
**H**	5.30	8.70(46), 1.30(74)	43a, 43b, 46, 74	45	43, 47, 74	41, 47b
**46 C**	66.11			46	47a, 47b, 48	
**H**	4.67	8.70(45)	45, 47a, 47b, 74	46	44, 45, 47, 48	47a, 48, 49', 74
**47 C**	43.23			47a, 47b	45, 46, 48, 49', 74	
**Ha**	1.58	14.30(47b)	46, 47b, 48	47	45, 46, 48, 49	46, 49'
**Hb**	1.89	14.30(47a)	46, 47a, 48	47	45, 46, 48, 49	45, 48, 49'
**48 C**	68.24			48	46, 47a, 47b, 49'	
**H**	4.10	7.30(49')	47a, 47b, 49'	48	46, 47, 49	46, 47b
**49 C**	86.08			49'	47a, 47b, 48, 50, 51a, 51b, 52'	
**49' H**	3.42	4.20(50), 7.30(48)	48, 50	49	47, 48, 50, 52	46, 47a, 47b, 50, 51b, 52'
**50 C**	73.44			50	49', 51a, 51b, 52'	
**H**	4.45	4.20(49')	49', 51a, 51b	50	49, 52	49', 51b, 52'
**51 C**	41.68			51a, 51b	53a, 53b	
**Ha**	1.65	13.30(51b)	50, 51b, 52'	51	49, 50, 52, 53	
**Hb**	2.36	13.30(51a)	50, 51a, 52'	51	49, 50, 52, 53	49', 50, 52'
**52 C**	76.49			52'	49', 50, 51a, 51b, 53a, 53b, 54	
**52' H**	4.11		51a, 51b, 53a, 53b	52	49, 50, 54	49', 50, 51b, 54
**53 C**	39.56			53a, 53b	51a, 51b, 54, 55	
**Ha**	1.88	8.80(54), 13.80(53b)	52', 53b, 54	53	51, 52, 54, 55	55
**Hb**	1.99	13.80(53a), 3.00(54)	52', 53a, 54	53	51, 52, 54, 55	54
**54 C**	74.28			54	52', 53a, 53b, 55, 56, 58	
**H**	3.60	8.80(53a), 9.70(55), 3.00(53b)	53a, 53b, 55	54	52, 53, 55, 56, 58	52', 53b, 58

**55 C**	73.10			55	53a, 53b, 54, 56, 57ax, 57eq	
**H**	3.28	9.70(54), 3.10(56)	54, 56	55	53, 54, 56, 57	53a, 56, 57ax
**56 C**	68.81			56	54, 55, 57eq	
**H**	4.01	3.10(55)	55, 57ax, 57eq	56	54, 55, 57, 58	55
**57 C**	39.58			57ax, 57eq	55, 56, 59a, 59b	
**Hax**	1.52		56, 57eq, 58	57	55, 58, 59	55
**Heq**	1.82		56, 57ax, 58	57	55, 56	58
**58 C**	70.91			58	54, 56, 57ax, 59a, 59b	
**H**	3.86		57ax, 57eq, 59a, 59b	58	54, 60	54, 57eq, 75
**59 C**	46.54			59a, 59b	57ax, 61, 75	
**Ha**	2.04		58, 59b, 61, 75	59	57, 58, 60, 61, 75	
**Hb**	2.18		58, 59a, 61, 75	59	57, 58, 60, 61, 75	
**60 C**	135.82				58, 59a, 59b, 62, 75	
**61 C**	130.88			61	59a, 59b, 62, 63a, 63b, 75	
**H**	5.22	1.36(75), 8.66(62)	59a, 59b, 62, 75	61	59, 63, 75	
**62 C**	69.10			62	63a, 63b, 64, 75	
**H**	4.37	8.66(61)	61, 63a, 63b, 75	62	60, 61, 63, 64	75
**63 C**	41.89			63a, 63b	61, 62, 64, 65, 75	
**Ha**	2.20	7.10(64), 14.00(63b)	62, 63b, 64, 65	63	61, 62, 64, 65	
**Hb**	2.27	14.00(63a), 7.10(64)	62, 63a, 64, 65	63	61, 62, 64, 65	
**64 C**	129.08			64	62, 63a, 63b, 65, 66	
**H**	5.66	15.30(65), 7.10(63a), 7.10(63b)	63a, 63b, 65, 66	64	62, 63, 65, 66	66
**65 C**	136.22			65	63a, 63b, 64, 66, 67a, 67b	
**H**	5.51	15.30(64), 7.40(66)	63a, 63b, 64, 66	65	63, 64, 66, 67	68
**66 C**	71.77			66	64, 65, 67a, 67b, 68	
**H**	4.25	7.40(65), 5.70(67a), 8.00(67b)	64, 65, 67a, 67b	66	64, 65, 67, 68	64, 67a, 68
**67 C**	40.91			67a, 67b	65, 66, 68, 69	
**Ha**	1.63	9.70(68), 5.70(66), 13.90(67b)	66, 67b, 68	67	65, 66, 68, 69	66, 69
**Hb**	1.96	8.00(66), 13.90(67a), 2.60(68)	66, 67a, 68	67	65, 66, 68, 69	68, 69
**68 C**	74.97			68	66, 67a, 67b, 69, 70, 72a, 72b	
**H**	3.57	9.50(69), 9.70(67a), 2.60(67b)	67a, 67b, 69	68	66, 67, 69, 70, 72	65, 66, 67b, 72b
**69 C**	73.28			69	67a, 67b, 68, 70, 71a	
**H**	3.23	9.50(68), 3.00(70)	68, 70	69	67, 68, 70	67a, 67b, 70, 71b
**70 C**	68.07			70	68, 69, 71a, 72a, 72b	
**H**	4.00	3.00(69)	69, 71a, 71b	70	68, 69, 71, 72	69
**71 C**	33.79			71a, 71b	70, 72a, 72b	
**Ha**	1.75		70, 71b, 72a, 72b	71	69, 70	
**Hb**	1.84		70, 71a, 72a, 72b	71	72	69
**72 C**	62.59			72a, 72b	68, 70, 71b	
**Ha**	3.61		71a, 71b, 72b	72	68, 70, 71	
**Hb**	3.69		71a, 71b, 72a	72	68, 70, 71	68
**73 C**	111.13			73', 73''	9, 11a, 11b	
**H'**	4.72		9, 11a, 73''	73	9, 10, 11	9
**H''**	4.81		9, 11a, 73'	73	9, 10, 11	11b

**74 C**	16.80			74	43a, 43b, 45	
**H3**	1.73	1.30(45)	43b, 45, 46	74	43, 44, 45, 47	46
**75 C**	17.42			75	59a, 59b, 61	
**H3**	1.71	1.36(61)	59a, 59b, 61, 62	75	59, 60, 61, 62, 63	58, 62

Table S10. Comparison of NMR data of authentic benthol A[7] and synthetic **40-*epi*-1** (revised structure)

Atom	δ (ppm, ^{1}H NMR in CD_3OD)		Δδ	δ (ppm, ^{13}C NMR in CD_3OD)[b]		Δδ
	benthol A	**40-*epi*-1**		benthol A	**40-*epi*-1**	
**1a**	3.48	3.49	-0.01	66.99	66.98	0.01
**1b**	3.48	3.49	-0.01			
**2**	3.90	3.91	-0.01	72.97	72.96	0.01
**3a**	1.46	1.47	-0.01	36.38	36.37	0.01
**3b**	2.15	2.16	-0.01			
**4**	3.67	3.68	-0.01	73.54	73.52	0.02
**5**	3.01	3.02	-0.01	77.31	77.31	0
**6**	3.83	3.84	-0.01	70.14	70.13	0.01
**7ax**	1.50	1.52	-0.02	43.99	43.99	0
**7eq**	2.10	2.10	0			
**8**	-	-	-	99.76	99.75	0.01
**9a**	2.24	2.25	-0.01	44.89	44.88	0.01
**9b**	2.24	2.25	-0.01			
**10**	-	-	-	143.05	143.04	0.01
**11a**	1.98	1.99	-0.01	41.23	41.22	0.01
**11b**	2.22	2.22	0			
**12**	3.87	3.87	0	68.65	68.64	0.01
**13a**	1.49[a]	1.49	0	43.80	43.79	0.01
**13b**	1.67	1.67	0			
**14**	3.90	3.91	-0.01	68.23[c]	68.23	0
**15a**	2.21	2.22	-0.01	42.66	42.65	0.01
**15b**	2.21	2.22	-0.01			
**16**	5.69	5.69	0	128.77	128.75	0.02
**17**	5.54	5.55	-0.01	137.03	137.02	0.01
**18**	4.05	4.05	0	73.83	73.82	0.01
**19a**	1.63	1.63	0	35.10	35.10	0
**19b**	1.63	1.63	0			
**20a**	1.64	1.64	0	26.20	26.19	0.01
**20b**	1.71	1.72	-0.01			
**21**	3.53	3.54	-0.01	84.52	84.51	0.01
**22**	4.18	4.19	-0.01	73.22	73.22	0
**23a**	1.49	1.50	-0.01	42.35	42.35	0
**23b**	2.38	2.38	0			
**24**	3.75	3.76	-0.01	79.06	79.05	0.01
**25a**	1.56	1.57	-0.01	37.40	37.39	0.01
**25b**	1.71	1.72	-0.01			
**26a**	1.38	1.40	-0.02	27.68	27.67	0.01
**26b**	1.48	1.49	-0.01			
**27a**	1.40	1.42	-0.02	27.64	27.63	0.01
**27b**	1.48	1.49	-0.01			
**28a**	1.64	1.65	-0.01	30.17	30.16	0.01
**28b**	1.64	1.65	-0.01			
**29**	3.56	3.56	0	84.69	84.68	0.01
**30**	4.18	4.19	-0.01	73.30	73.29	0.01
**31a**	1.55	1.55	0	42.70	42.69	0.01
**31b**	2.42	2.42	0			

**32**	4.08	4.08	0	75.39	75.38	0.01
**33a**	1.52	1.52	0	41.73	41.71	0.02
**33b**	1.82	1.83	-0.01			
**34**	4.18	4.18	0	67.14	67.14	0
**35**	3.62	3.62	0	77.77	77.76	0.01
**36ax**	1.72	1.71	0.01	34.16	34.15	0.01
**36eq**	2.03	2.03	0			
**37**	3.76	3.76	0	71.09	71.08	0.01
**38**	3.49	3.49	0	72.78	72.77	0.01
**39**	3.69	3.70	-0.01	73.22	73.22	0
**40**	3.62	3.63	-0.01	73.10	73.09	0.01
**41**	3.63	3.63	0	72.47	72.47	0
**42a**	1.50	1.51	-0.01	33.45	33.45	0
**42b**	1.99	2.00	-0.01			
**43a**	2.11	2.11	0	36.80	36.80	0
**43b**	2.27	2.27	0			
**44**	-	-	-	138.12	138.11	0.01
**45**	5.29	5.30	-0.01	129.58	129.57	0.01
**46**	4.67	4.67	0	66.15	66.15	0
**47a**	1.58	1.58	0	43.29	43.27	0.02
**47b**	1.88	1.89	-0.01			
**48**	4.10	4.10	0	68.28[c]	68.28	0
**49**	3.42	3.42	0	86.13	86.12	0.01
**50**	4.45	4.45	0	73.49	73.49	0
**51a**	1.64	1.65	-0.01	41.73	41.72	0.01
**51b**	2.36	2.36	0			
**52**	4.11	4.11	0	76.54	76.53	0.01
**53a**	1.88	1.88	0	39.61	39.60	0.01
**53b**	1.99	1.99	0			
**54**	3.59	3.60	-0.01	74.33	74.32	0.01
**55**	3.27	3.28	-0.01	73.15	73.14	0.01
**56**	4.01	4.01	0	68.86	68.85	0.01
**57ax**	1.51	1.52	-0.01	39.63	39.62	0.01
**57eq**	1.82	1.82	0			
**58**	3.86	3.86	0	70.96	70.95	0.01
**59a**	2.04	2.04	0	46.58	46.58	0
**59b**	2.18	2.18	0			
**60**	-	-	-	135.86	135.85	0.01
**61**	5.21	5.22	-0.01	130.93	130.92	0.01
**62**	4.37	4.37	0	69.15	69.14	0.01
**63a**	2.20	2.20	0	41.94	41.93	0.01
**63b**	2.26	2.27	-0.01			
**64**	5.65	5.66	-0.01	129.14	129.12	0.02
**65**	5.50	5.51	-0.01	136.26	136.26	0
**66**	4.25	4.25	0	71.82	71.81	0.01
**67a**	1.63	1.63	0	40.96	40.95	0.01
**67b**	1.96	1.96	0			
**68**	3.56	3.57	-0.01	75.01	75.01	0
**69**	3.22	3.23	-0.01	73.33	73.32	0.01
**70**	4.00	4.00	0	68.12	68.11	0.01
**71a**	1.74	1.75	-0.01	33.84	33.83	0.01
**71b**	1.83	1.84	-0.01			
**72a**	3.61	3.61	0	62.63	62.63	0
**72b**	3.69	3.69	0			
**73a**	4.72	4.72	0	111.18	111.17	0.01
**73b**	4.81	4.81	0			
**74**	1.72	1.73	-0.01	16.84	16.84	0
**75**	1.70	1.71	-0.01	17.46	17.46	0

[a] The chemical shift of H-13a was deduced from the ^{1}H NMR copy (figure S15) of natural benthol A in the supporting information provided by the isolation team because it was not present in the text.[7]

[b] Given the ^{13}C NMR chemical shifts of natural benthol A were referenced to [D$_3$]-MeOH $\delta_C \equiv$ 49.04 ppm, therefore the ^{13}C NMR chemical shifts of our synthetic revised benthol A were also referenced to this shift value in order to ensure optimal comparison (note that this signal is otherwise referenced to 49.00 ppm throughout this paper, see General Information)

[c] The isolation team misassigned the chemical shifts of C14 and C48. The correct data is shown in the table.

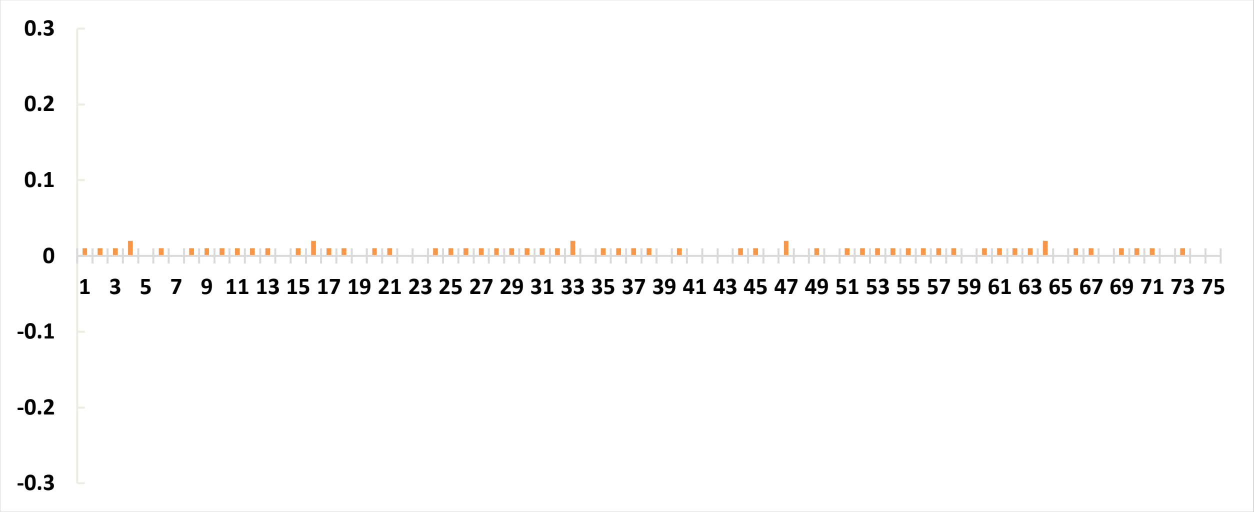

Figure S3. Graphical representation of the ^{13}C NMR shift differences ($\Delta\delta_C$) between authentic benthol A and synthetic **40-*epi*-1**. *Note*: for the sake of comparison, the spectra of our synthetic sample were referenced to [D$_3$]-MeOH (49.04 ppm) as the shift value used by to the isolation team (note that this signal is otherwise referenced to 49.00 ppm throughout this paper, see General Information)

Visual Comparison of the NMR Spectra of Authentic Benthol A (contained in Supporting information of Ref.[7]) with those of Synthetic 40-*epi*-1

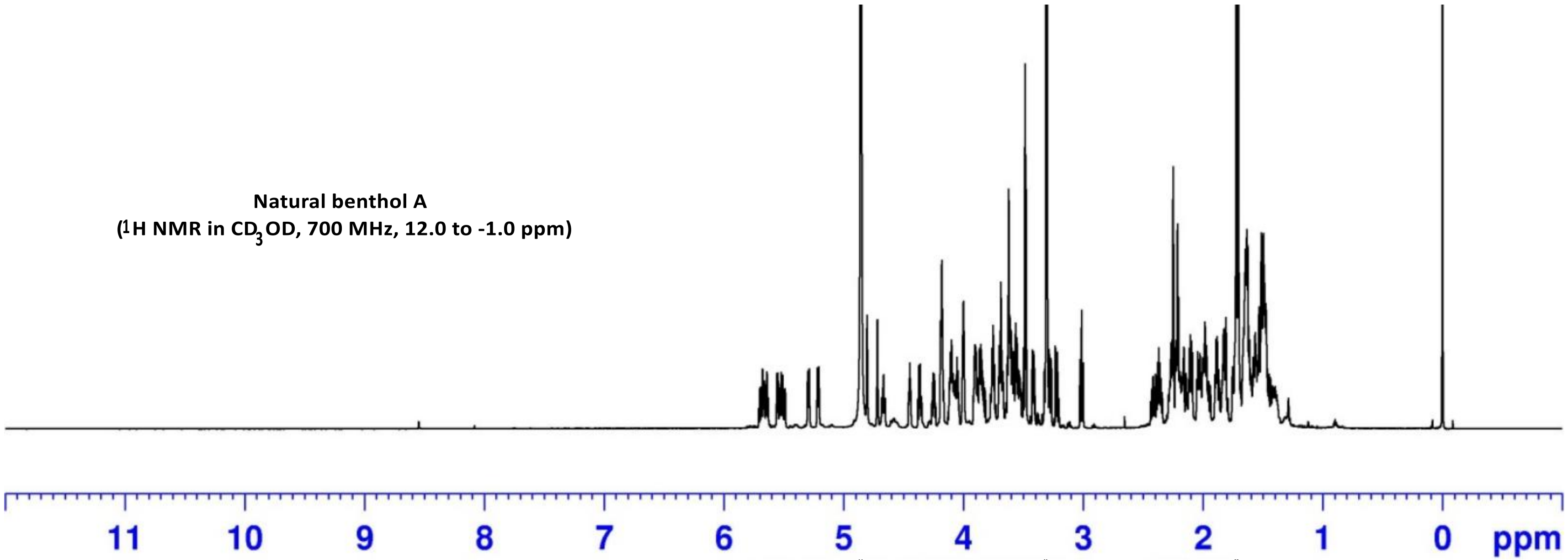

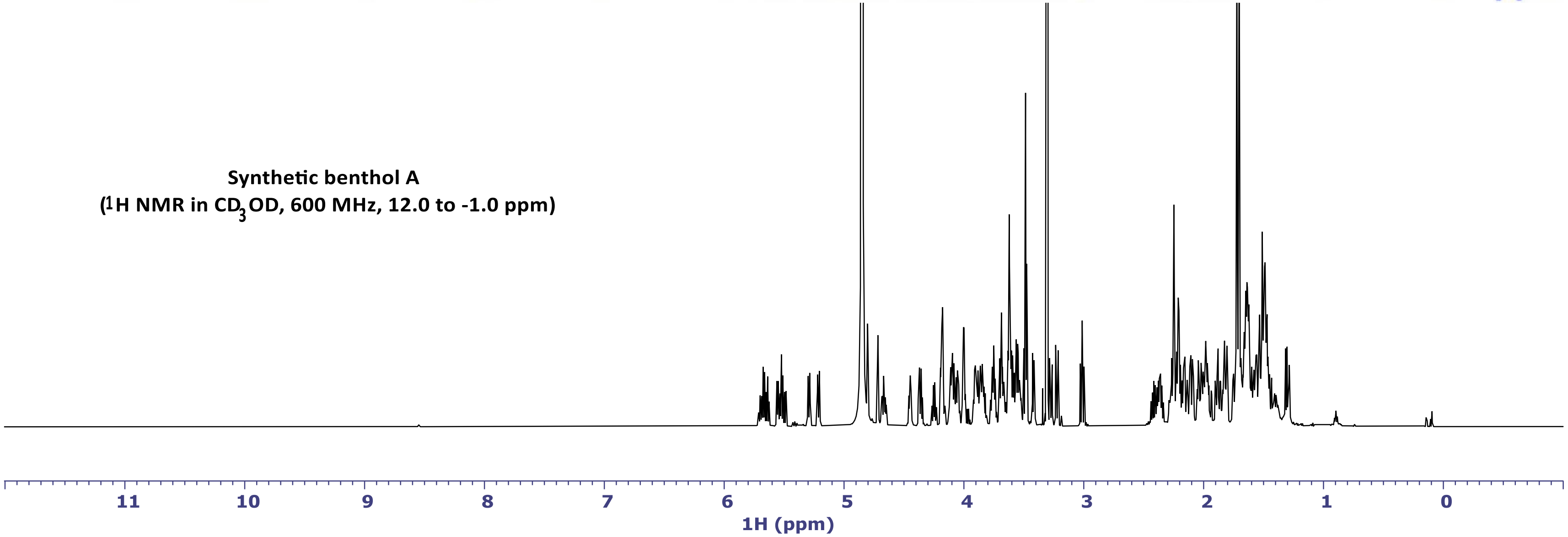

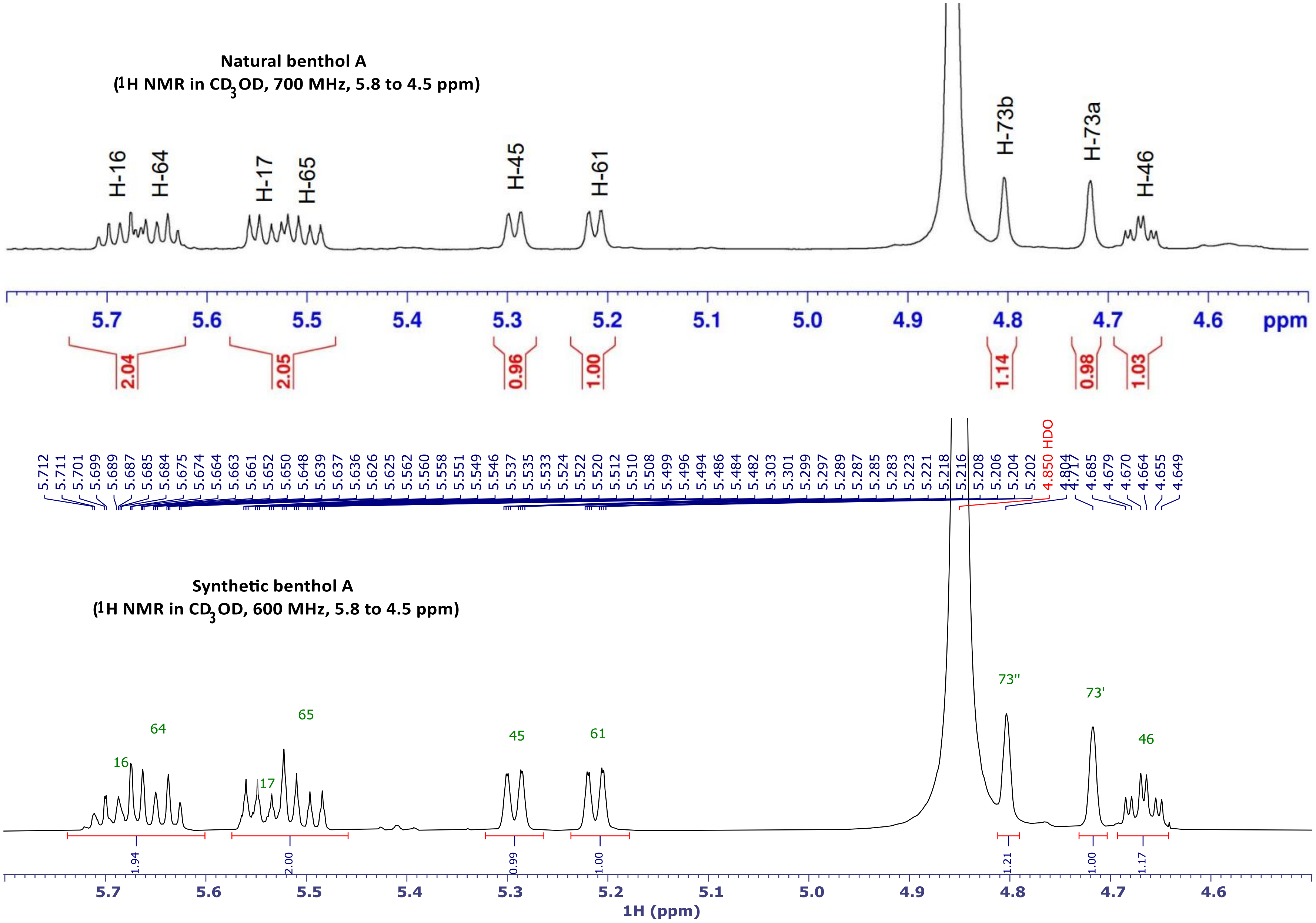
Natural benthol A
(1H NMR in CD3OD, 700 MHz, 5.8 to 4.5 ppm)
H-16
H-64
H-17
H-65
H-45
H-61
H-73b
H-73a
H-46
5.7
5.6
5.5
5.4
5.3
5.2
5.1
5.0
4.9
4.8
4.7
4.6
ppm
2.04
2.05
0.96
1.00
1.14
0.98
1.03
5.712 5.711 5.701 5.699 5.689 5.687 5.685 5.684 5.675 5.674 5.664 5.663 5.661 5.652 5.650 5.648 5.639 5.637 5.636 5.626 5.625 5.562 5.560 5.558 5.551 5.549 5.546 5.537 5.535 5.533 5.524 5.522 5.520 5.512 5.510 5.508 5.499 5.496 5.494 5.486 5.484 5.482 5.303 5.301 5.299 5.297 5.289 5.287 5.285 5.283 5.223 5.221 5.218 5.216 5.208 5.206 5.204 5.202 4.850 HDO 4.804 4.717 4.685 4.679 4.670 4.664 4.655 4.649
Synthetic benthol A
(1H NMR in CD3OD, 600 MHz, 5.8 to 4.5 ppm)
16
64
17
65
45
61
73''
73'
46
1.94
2.00
0.99
1.00
1.21
1.00
1.17
5.7
5.6
5.5
5.4
5.3
5.2
5.1
5.0
4.9
4.8
4.7
4.6
1H (ppm)

Natural benthol A
(^{1}H NMR in CD_3OD, 700 MHz, 4.5 to 3.8 ppm)

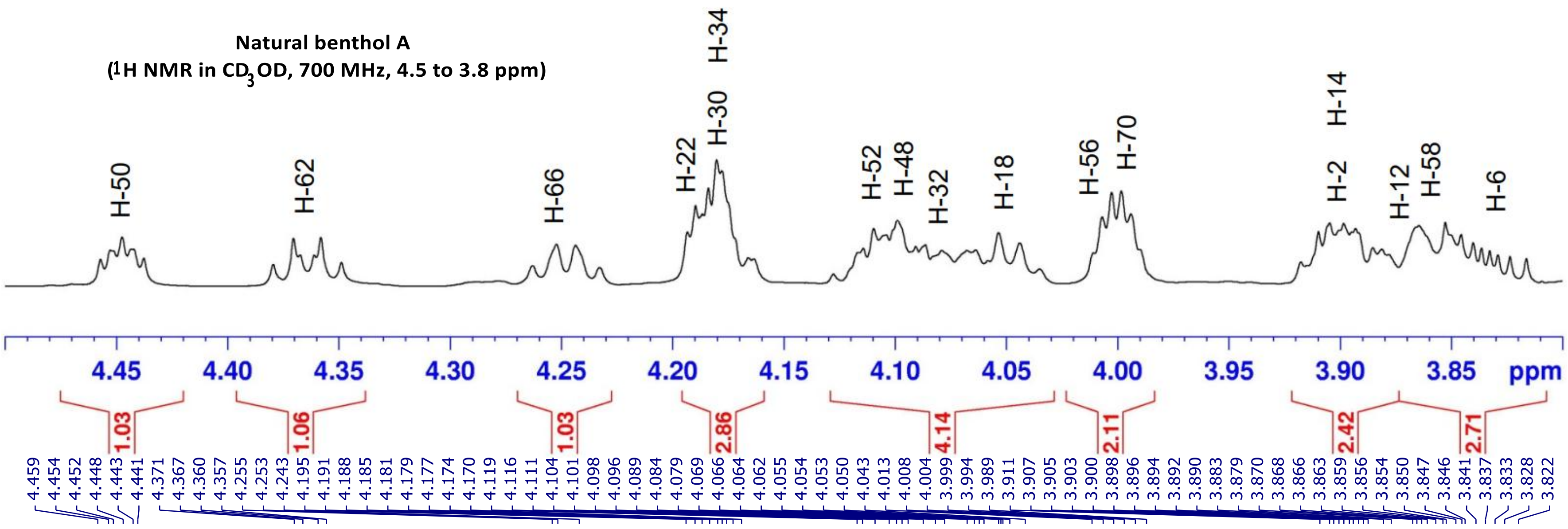

Synthetic benthol A
(^{1}H NMR in CD_3OD, 600 MHz, 4.5 to 3.8 ppm)

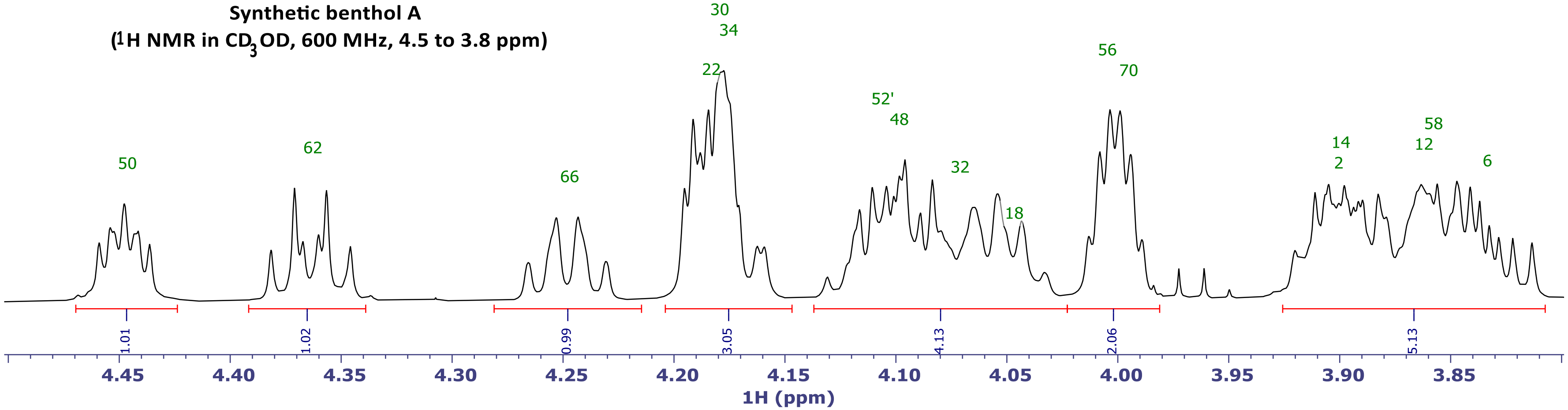

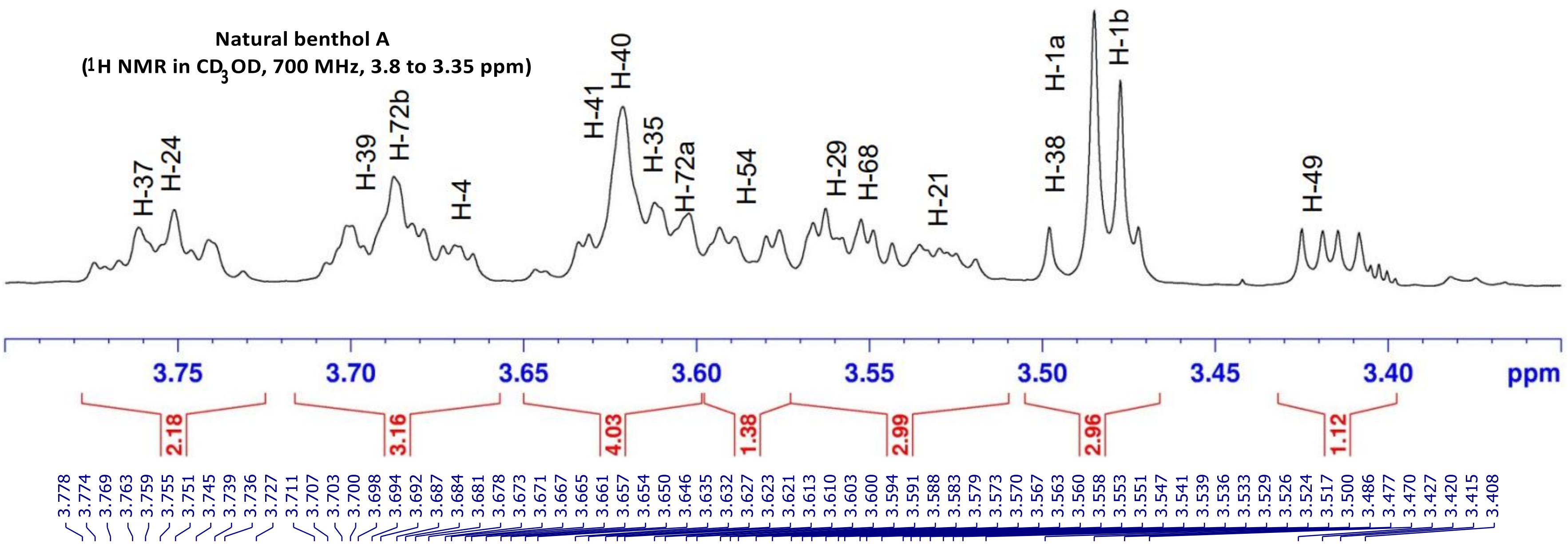
Natural benthol A
(1H NMR in CD3OD, 700 MHz, 3.8 to 3.35 ppm)
H-37
H-24
H-39
H-72b
H-4
H-41
H-40
H-35
H-72a
H-54
H-29
H-68
H-21
H-38
H-1a
H-1b
H-49
3.75
3.70
3.65
3.60
3.55
3.50
3.45
3.40
ppm
2.18
3.16
4.03
1.38
2.99
2.96
1.12
3.778 3.774 3.769 3.763 3.759 3.755 3.751 3.745 3.739 3.736 3.727 3.711 3.707 3.703 3.700 3.698 3.694 3.692 3.687 3.684 3.681 3.678 3.673 3.671 3.667 3.665 3.661 3.657 3.654 3.650 3.646 3.635 3.632 3.627 3.623 3.621 3.613 3.610 3.603 3.600 3.594 3.591 3.588 3.583 3.579 3.573 3.570 3.567 3.563 3.560 3.558 3.553 3.551 3.547 3.541 3.539 3.536 3.533 3.529 3.526 3.524 3.517 3.500 3.486 3.477 3.470 3.427 3.420 3.415 3.408

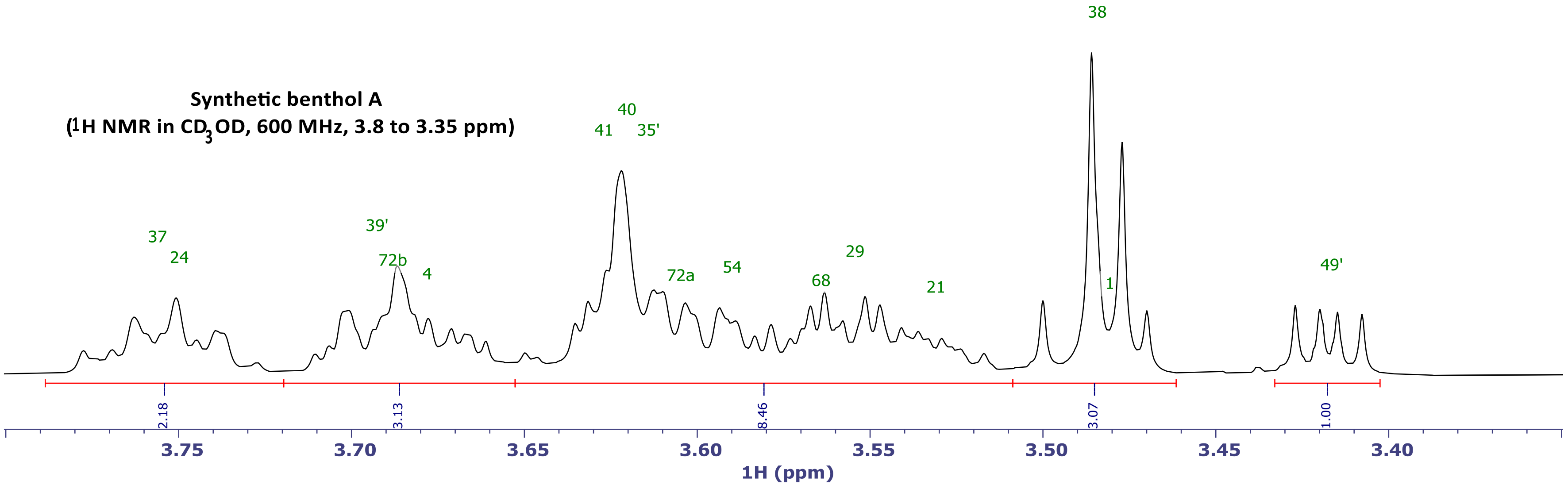
Synthetic benthol A
(1H NMR in CD3OD, 600 MHz, 3.8 to 3.35 ppm)
38
40
41
35'
39'
37
24
72b
4
72a
54
29
68
21
1
49'
2.18
3.13
8.46
3.07
1.00
3.75
3.70
3.65
3.60
3.55
3.50
3.45
3.40
1H (ppm)

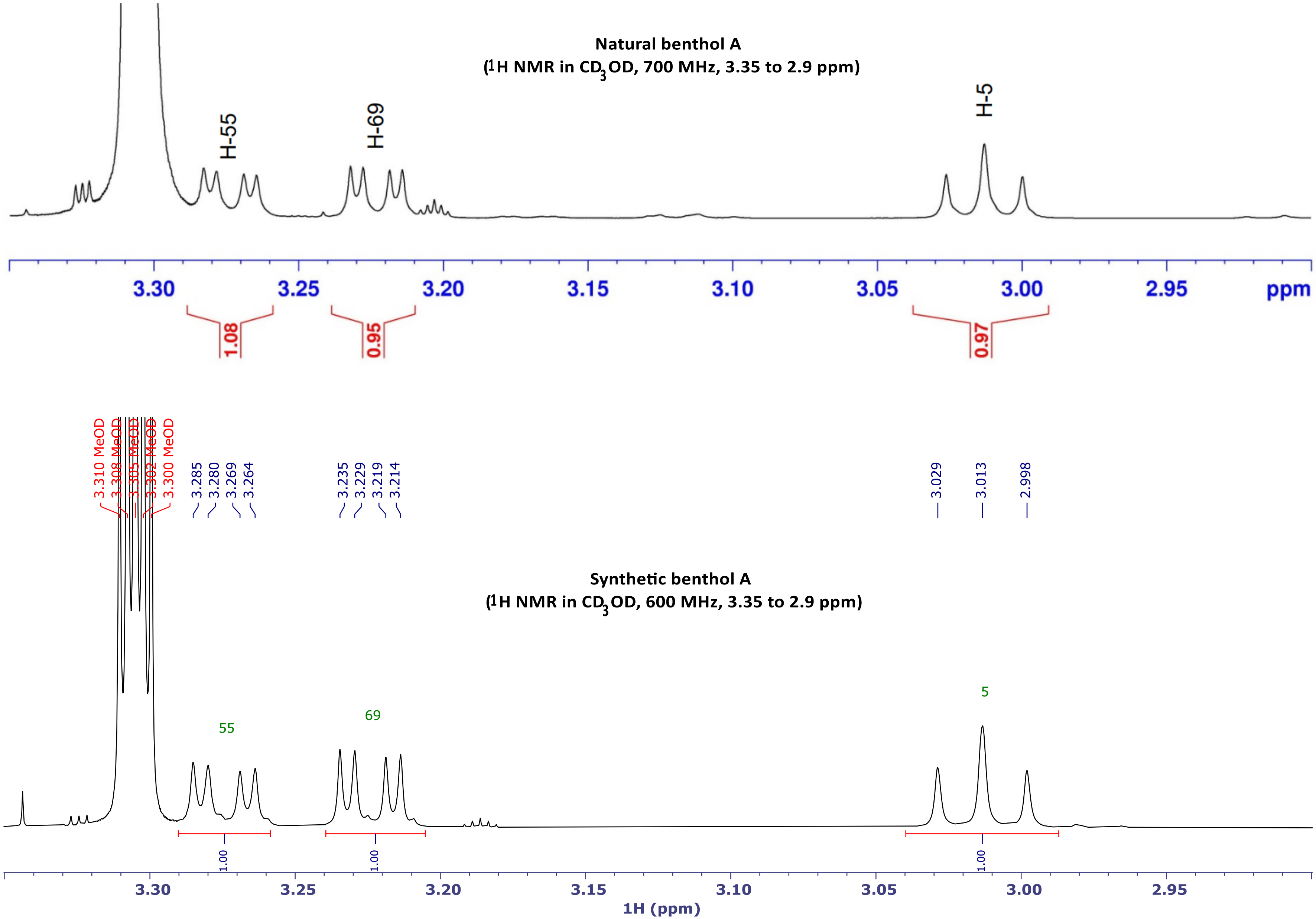
Natural benthol A
(1H NMR in CD3OD, 700 MHz, 3.35 to 2.9 ppm)
H-55
H-69
H-5
3.30
3.25
3.20
3.15
3.10
3.05
3.00
2.95
ppm
1.08
0.95
0.97
3.310 MeOD
3.308 MeOD
3.305 MeOD
3.302 MeOD
3.300 MeOD
3.285
3.280
3.269
3.264
3.235
3.229
3.219
3.214
3.029
3.013
2.998
Synthetic benthol A
(1H NMR in CD3OD, 600 MHz, 3.35 to 2.9 ppm)
55
69
5
1.00
1.00
1.00
3.30
3.25
3.20
3.15
3.10
3.05
3.00
2.95
1H (ppm)

Natural benthol A
(^{1}H NMR in CD_3OD, 700 MHz, 2.5 to 1.77 ppm)

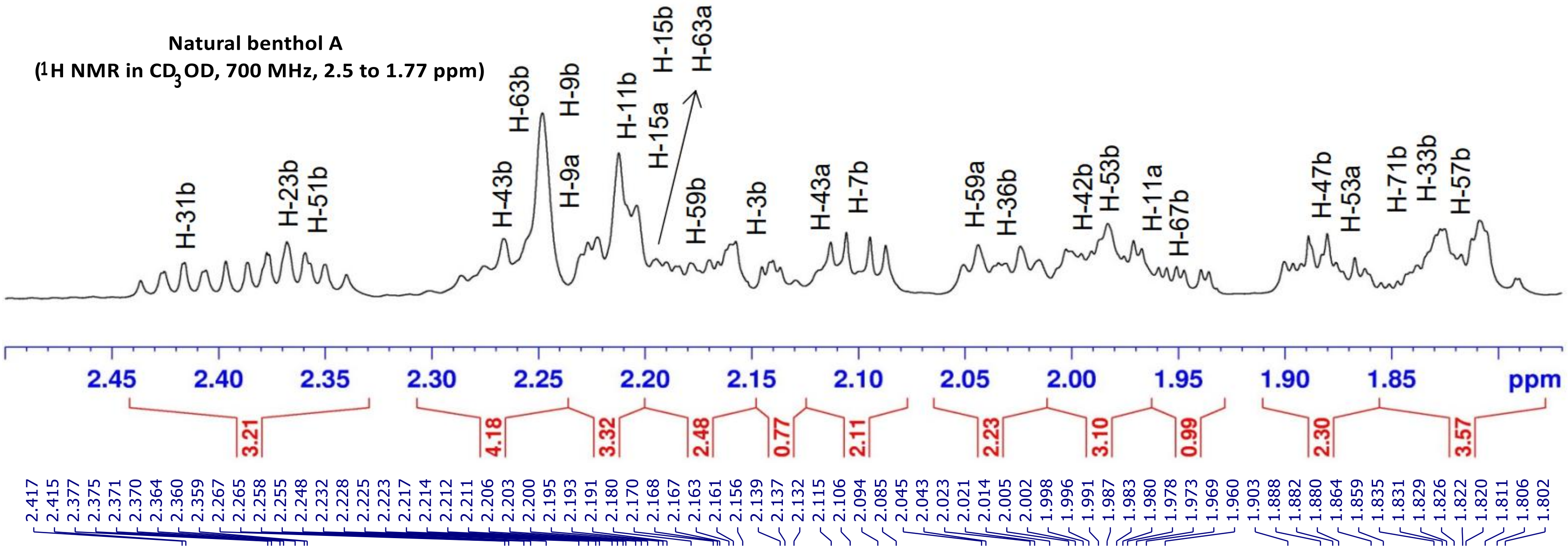

Synthetic benthol A
(^{1}H NMR in CD_3OD, 600 MHz, 2.5 to 1.77 ppm)

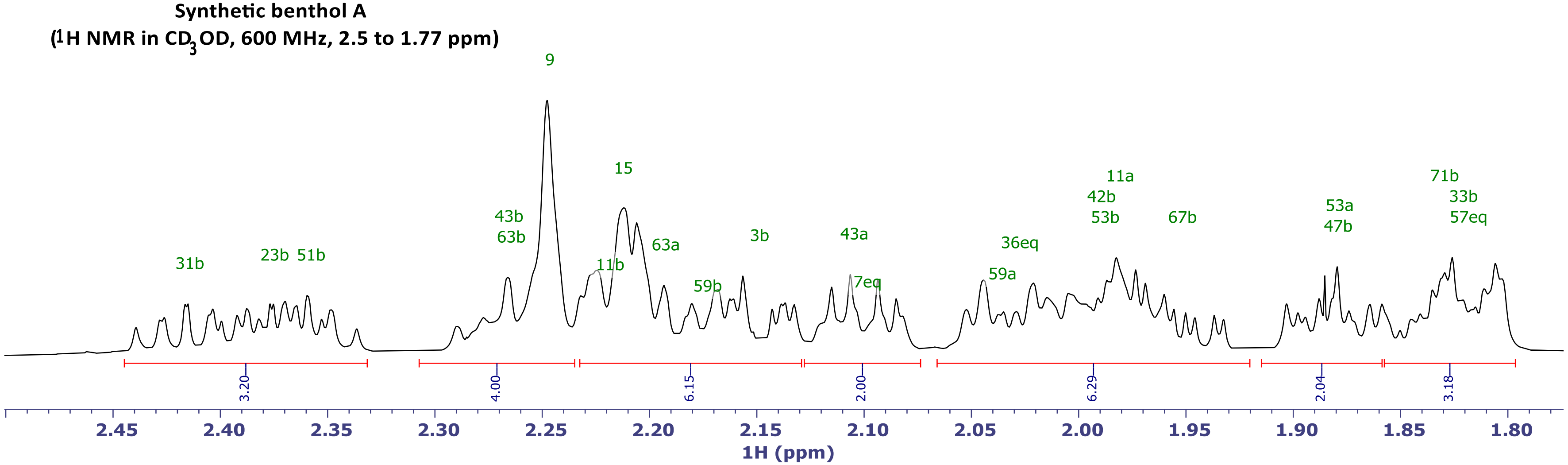

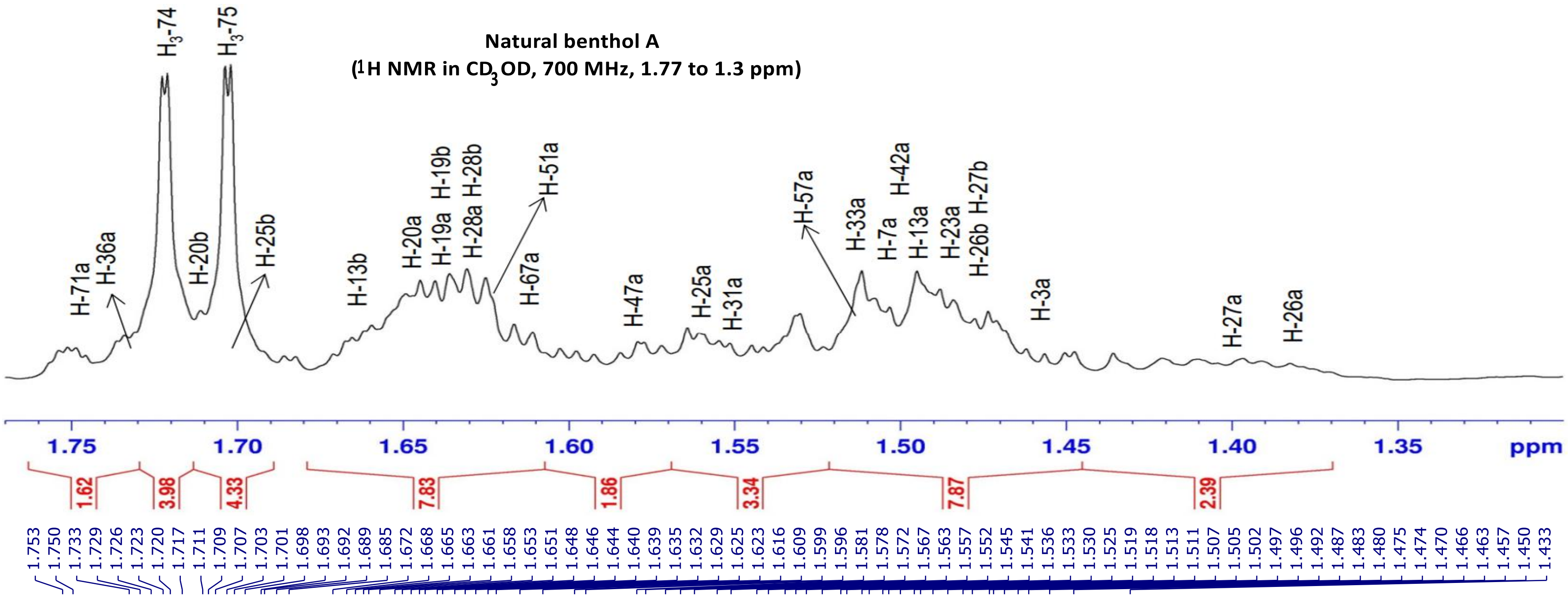
Natural benthol A
(1H NMR in CD3OD, 700 MHz, 1.77 to 1.3 ppm)
H3-74
H3-75
H-71a
H-36a
H-20b
H-25b
H-13b
H-20a
H-19a
H-19b
H-28a
H-28b
H-51a
H-67a
H-47a
H-25a
H-31a
H-57a
H-33a
H-7a
H-42a
H-13a
H-23a
H-26b
H-27b
H-3a
H-27a
H-26a
ppm
1.62
3.98
4.33
7.83
1.86
3.34
7.87
2.39

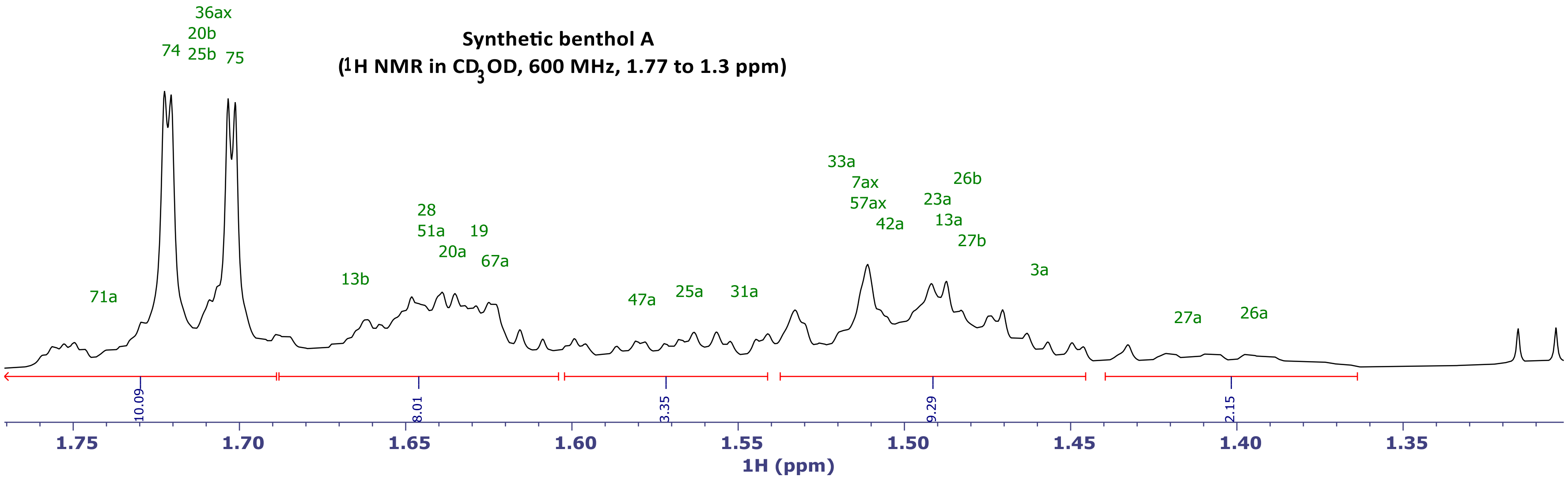
Synthetic benthol A
(1H NMR in CD3OD, 600 MHz, 1.77 to 1.3 ppm)
36ax
20b
74
25b
75
71a
13b
28
51a
19
20a
67a
47a
25a
31a
33a
7ax
57ax
42a
26b
23a
13a
27b
3a
27a
26a
10.09
8.01
3.35
9.29
2.15
1H (ppm)

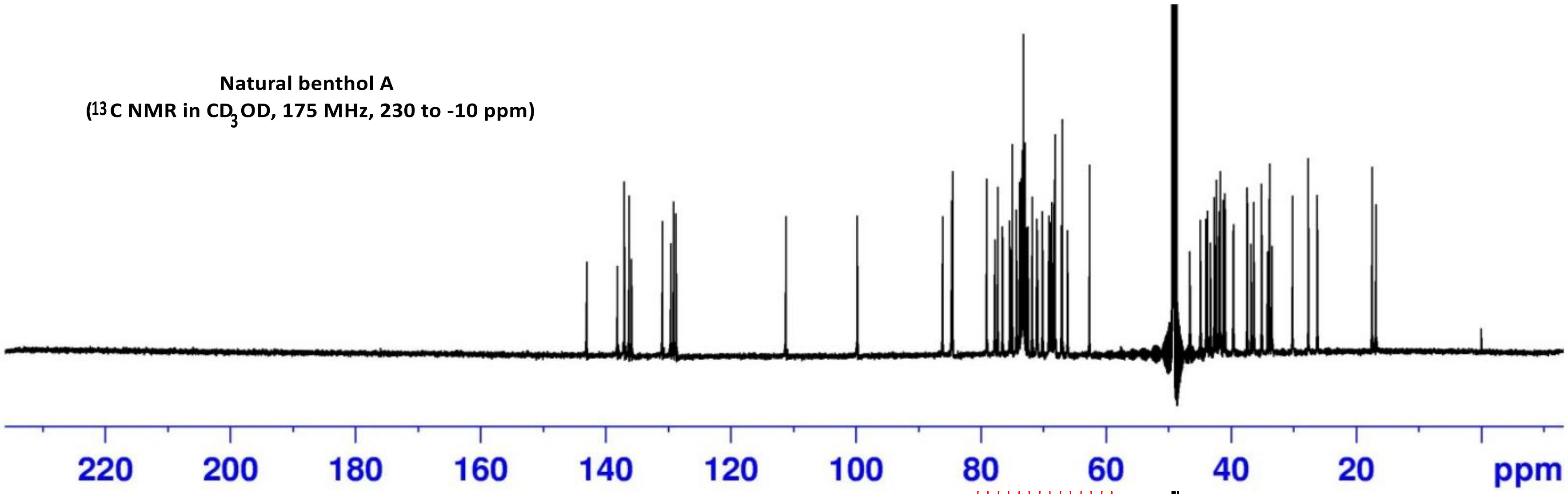

Natural benthol A
(13C NMR in CD3OD, 175 MHz, 230 to -10 ppm)
220
200
180
160
140
120
100
80
60
40
20
ppm

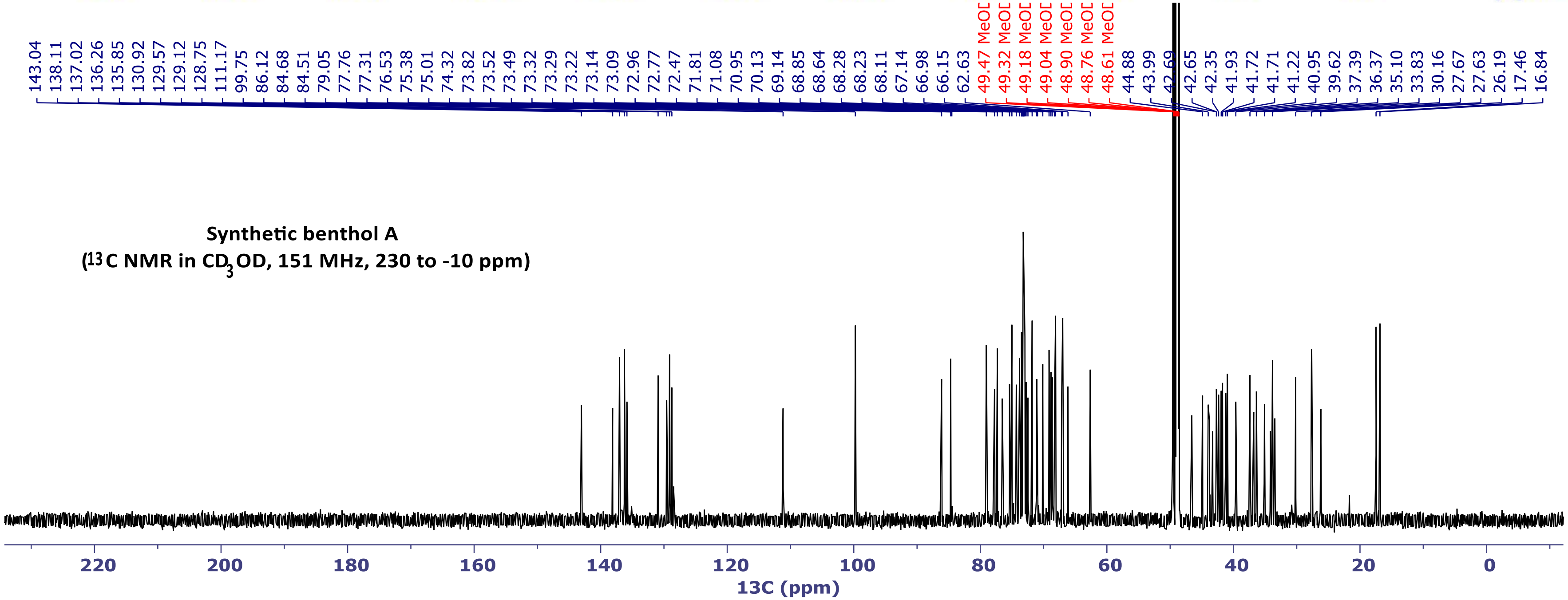

143.04
138.11
137.02
136.26
135.85
130.92
129.57
129.12
128.75
111.17
99.75
86.12
84.68
84.51
79.05
77.76
77.31
76.53
75.38
75.01
74.32
73.82
73.52
73.49
73.32
73.29
73.22
73.14
73.09
72.96
72.77
72.47
71.81
71.08
70.95
70.13
69.14
68.85
68.64
68.28
68.23
68.11
67.14
66.98
66.15
62.63
49.47 MeOD
49.32 MeOD
49.18 MeOD
49.04 MeOD
48.90 MeOD
48.76 MeOD
48.61 MeOD
44.88
43.99
42.69
42.65
42.35
41.93
41.72
41.71
41.22
40.95
39.62
37.39
36.37
35.10
33.83
30.16
27.67
27.63
26.19
17.46
16.84
Synthetic benthol A
(13C NMR in CD3OD, 151 MHz, 230 to -10 ppm)
220
200
180
160
140
120
100
80
60
40
20
0
13C (ppm)

Natural benthol A
(^{13}C NMR in CD_3OD, 175 MHz, 150 to 80 ppm)

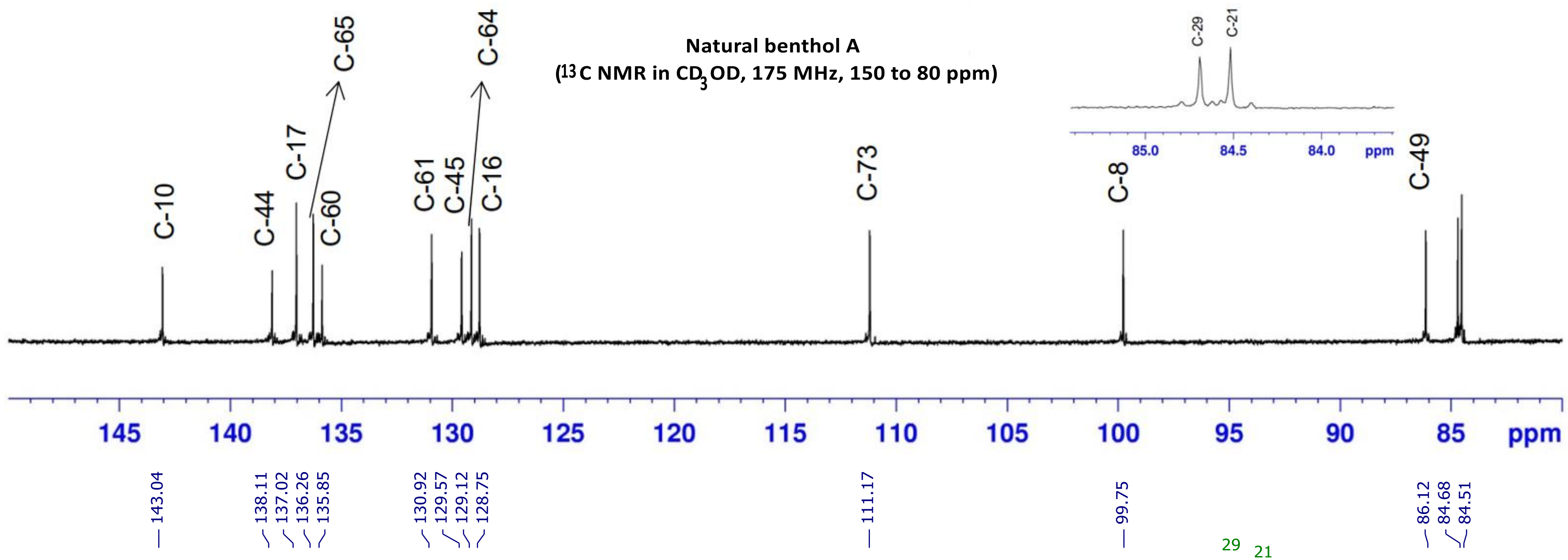

Synthetic benthol A
(^{13}C NMR in CD_3OD, 151 MHz, 150 to 80 ppm)

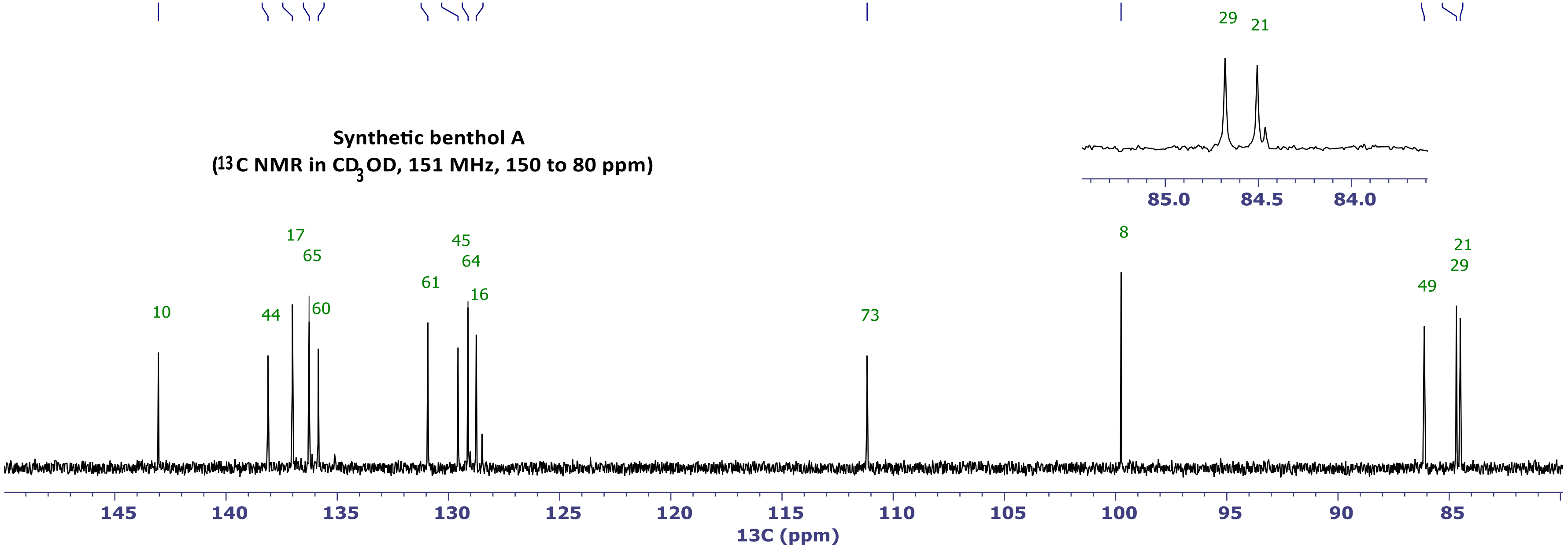

Natural benthol A
(^{13}C NMR in CD_3OD, 175 MHz, 80 to 73.6 ppm)

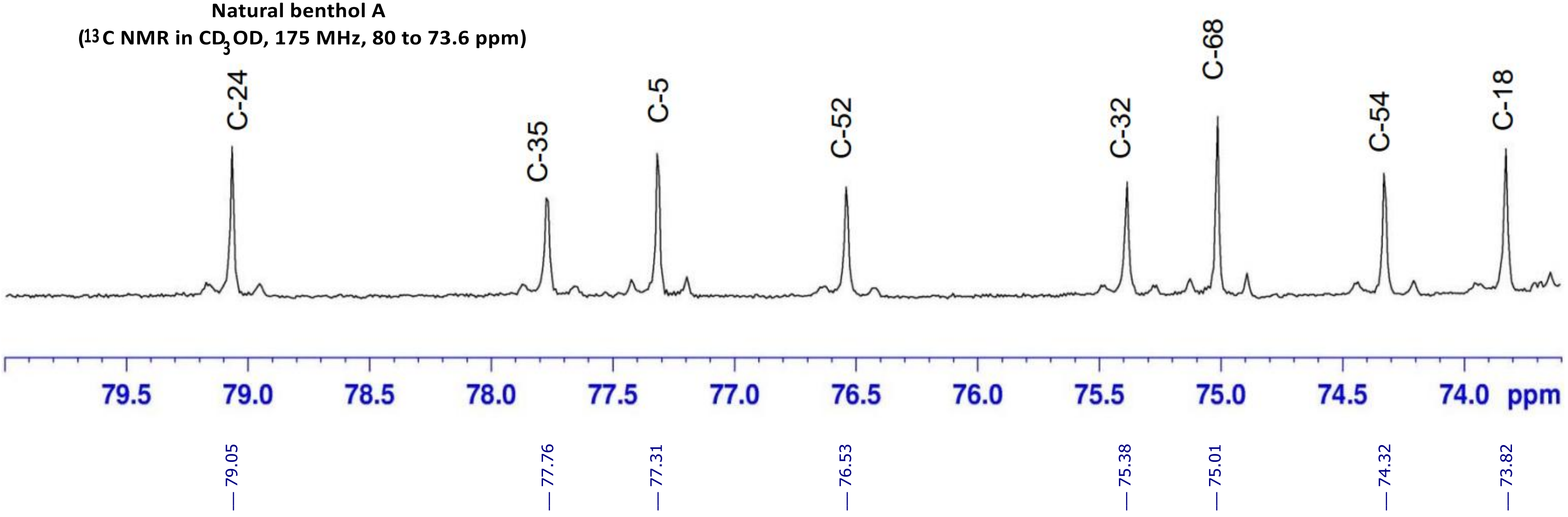

Synthetic benthol A
(^{13}C NMR in CD_3OD, 151 MHz, 80 to73.6 ppm)

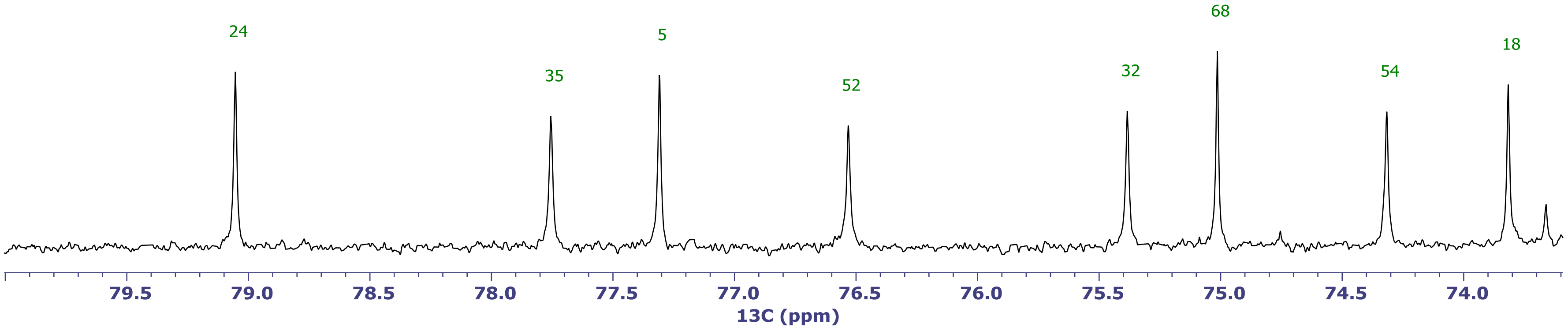

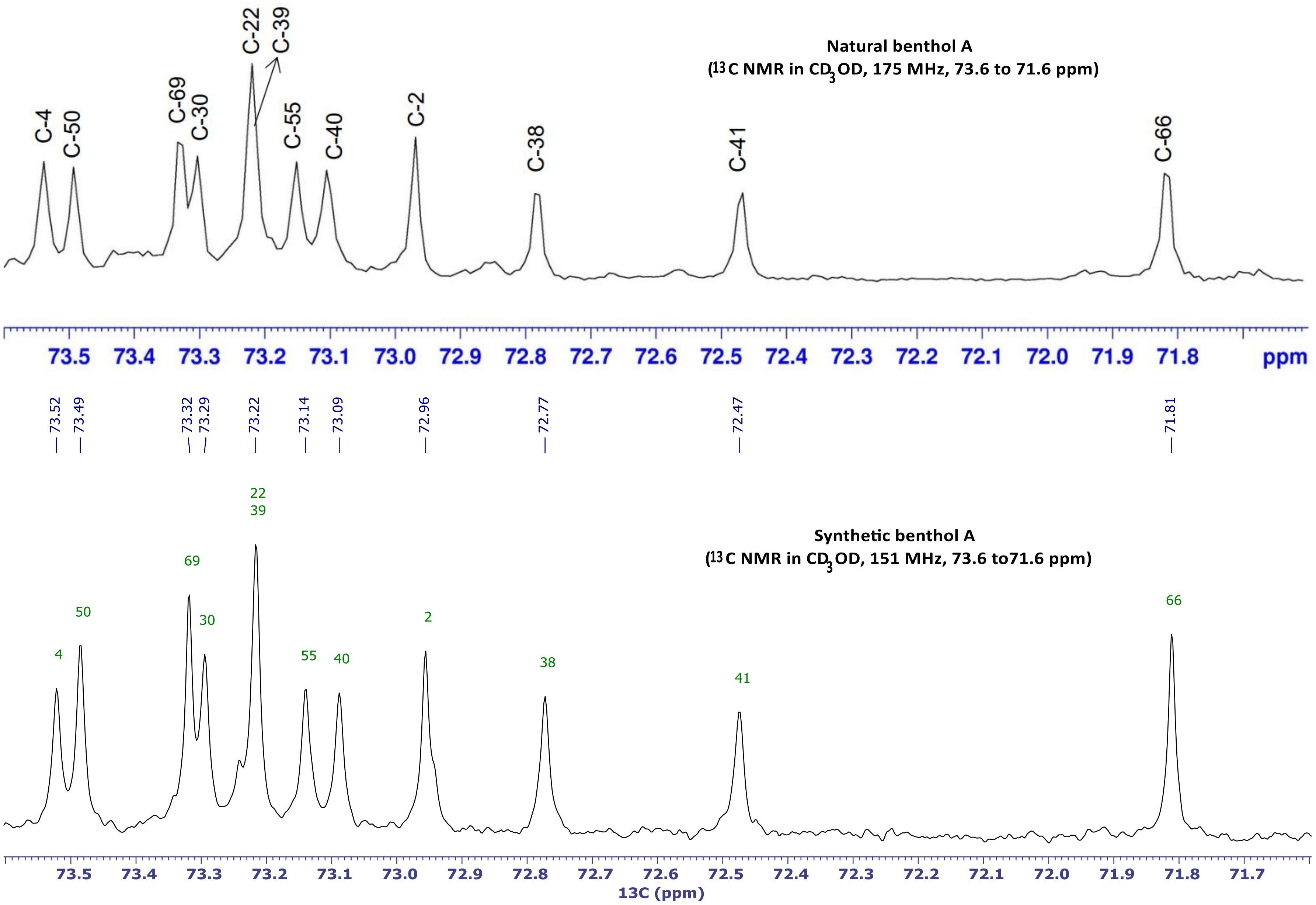
Natural benthol A
(^{13}C NMR in CD_3OD, 175 MHz, 73.6 to 71.6 ppm)
C-4
C-50
C-69
C-30
C-22
C-39
C-55
C-40
C-2
C-38
C-41
C-66
73.5 73.4 73.3 73.2 73.1 73.0 72.9 72.8 72.7 72.6 72.5 72.4 72.3 72.2 72.1 72.0 71.9 71.8 ppm
73.52
73.49
73.32
73.29
73.22
73.14
73.09
72.96
72.77
72.47
71.81
Synthetic benthol A
(^{13}C NMR in CD_3OD, 151 MHz, 73.6 to71.6 ppm)
4
50
69
30
22
39
55
40
2
38
41
66
73.5 73.4 73.3 73.2 73.1 73.0 72.9 72.8 72.7 72.6 72.5 72.4 72.3 72.2 72.1 72.0 71.9 71.8 71.7
13C (ppm)

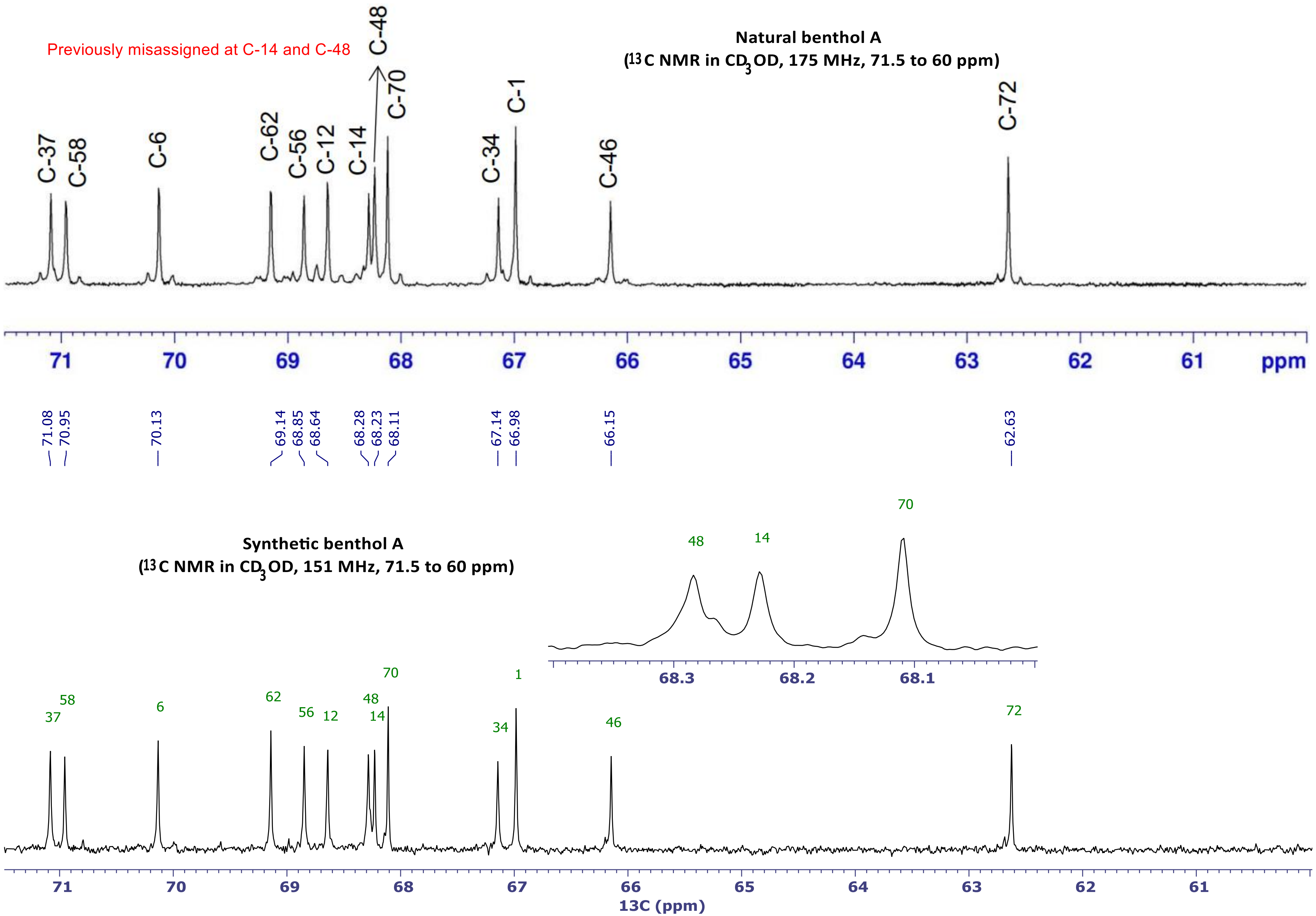
Previously misassigned at C-14 and C-48
Natural benthol A
(13C NMR in CD3OD, 175 MHz, 71.5 to 60 ppm)
C-37
C-58
C-6
C-62
C-56
C-12
C-14
C-48
C-70
C-34
C-1
C-46
C-72
71
70
69
68
67
66
65
64
63
62
61
ppm
71.08
70.95
70.13
69.14
68.85
68.64
68.28
68.23
68.11
67.14
66.98
66.15
62.63
Synthetic benthol A
(13C NMR in CD3OD, 151 MHz, 71.5 to 60 ppm)
48
14
70
68.3
68.2
68.1
37
58
6
62
56
12
48
14
70
34
1
46
72
71
70
69
68
67
66
65
64
63
62
61
13C (ppm)

Natural benthol A
(^{13}C NMR in CD_3OD, 175 MHz, 47.5 to 39 ppm)

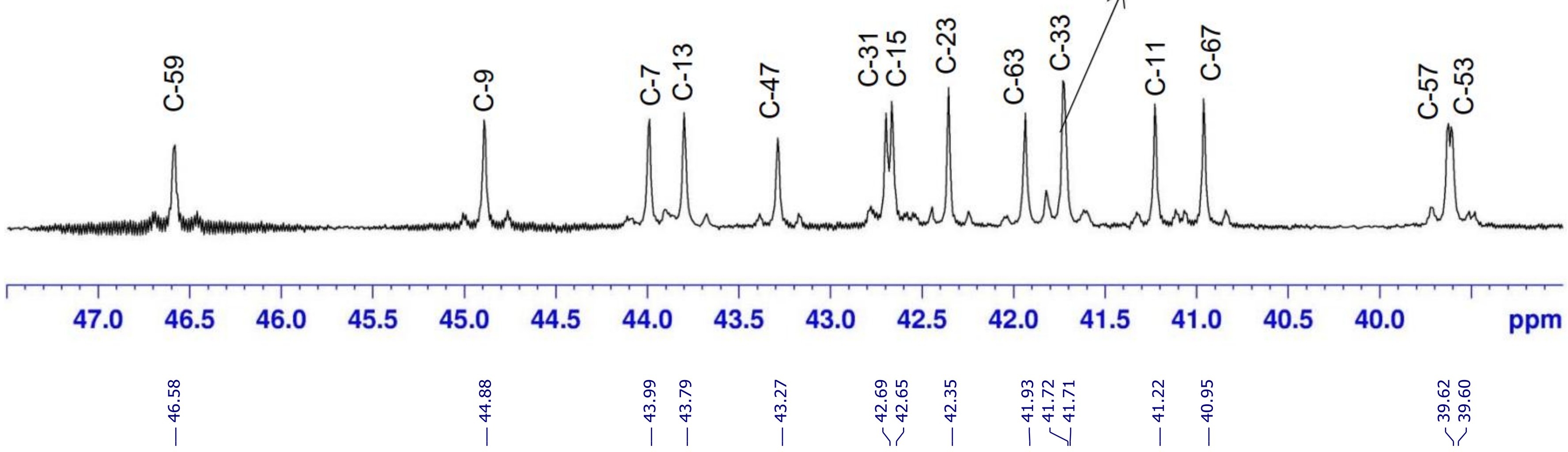

Synthetic benthol A
(^{13}C NMR in CD_3OD, 151 MHz, 47.5 to 39 ppm)

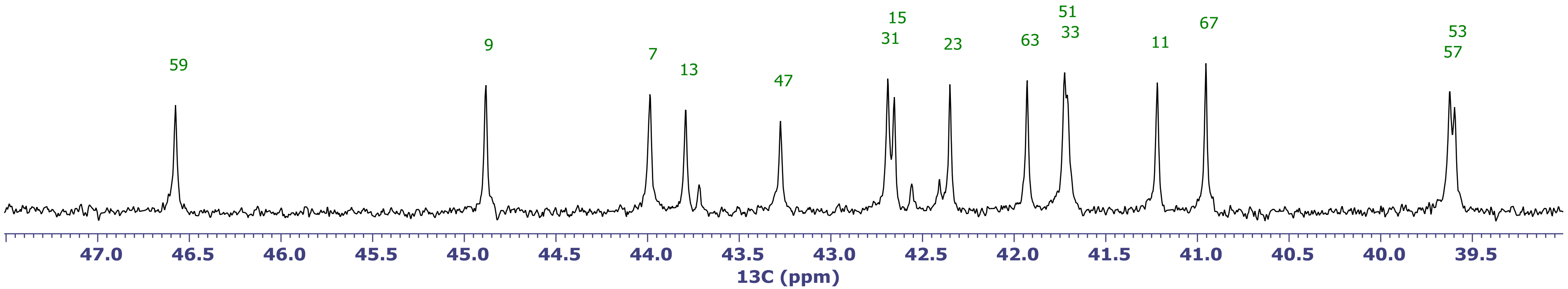

Natural benthol A
(^{13}C NMR in CD_3OD, 175 MHz, 39 to 15 ppm)

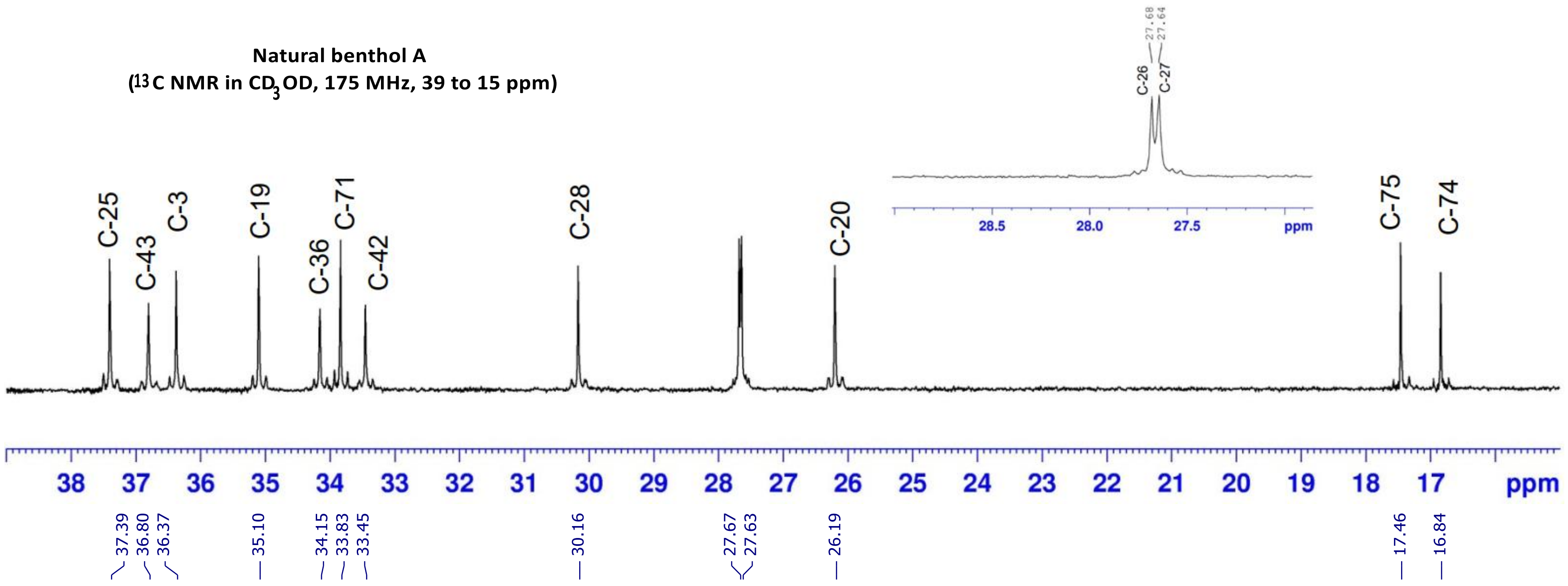

Synthetic benthol A
(^{13}C NMR in CD_3OD, 151 MHz, 39 to 15 ppm)

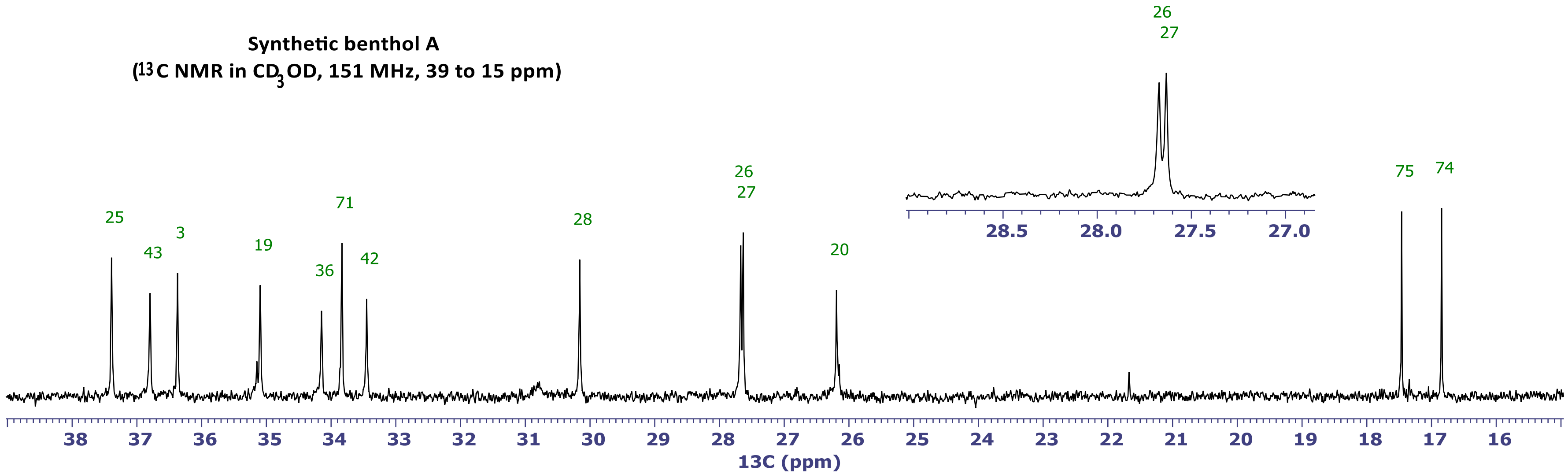

Copies of Spectra of New Compounds

Compound 3: ^{1}H NMR ($CDCl_3$, 400 MHz)

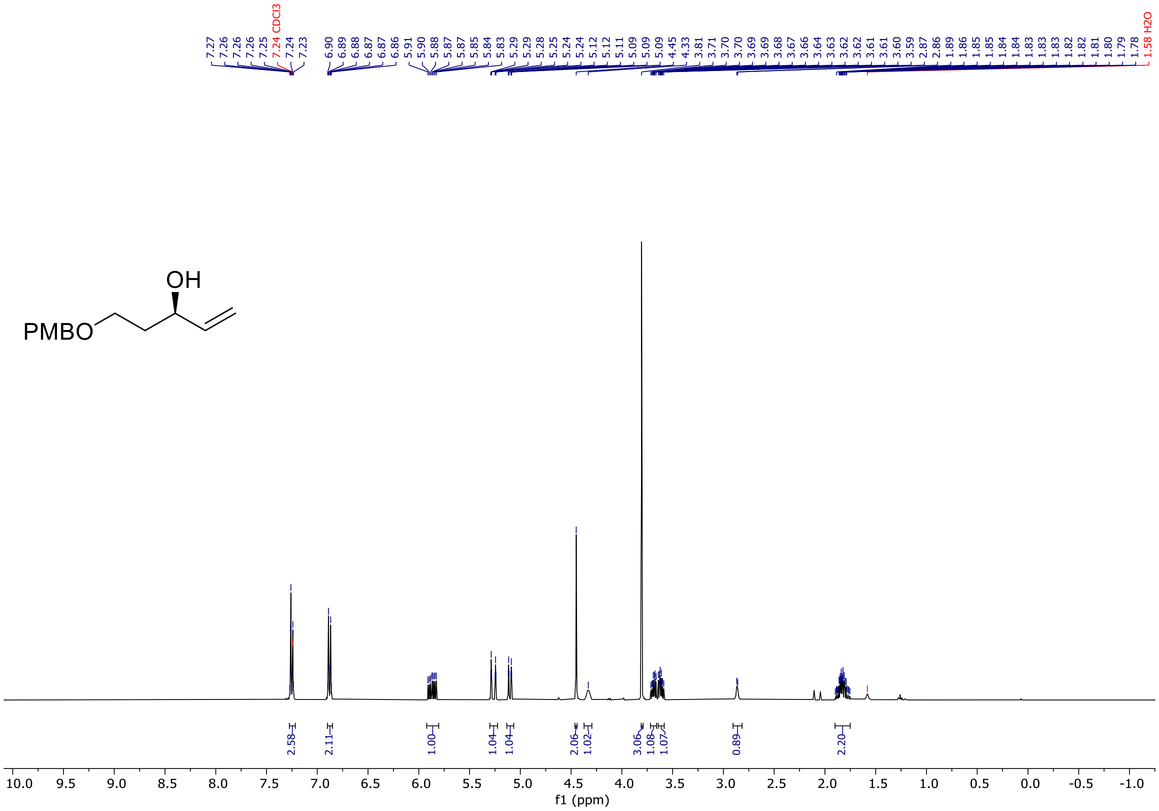

^{13}C NMR ($CDCl_3$, 101 MHz)

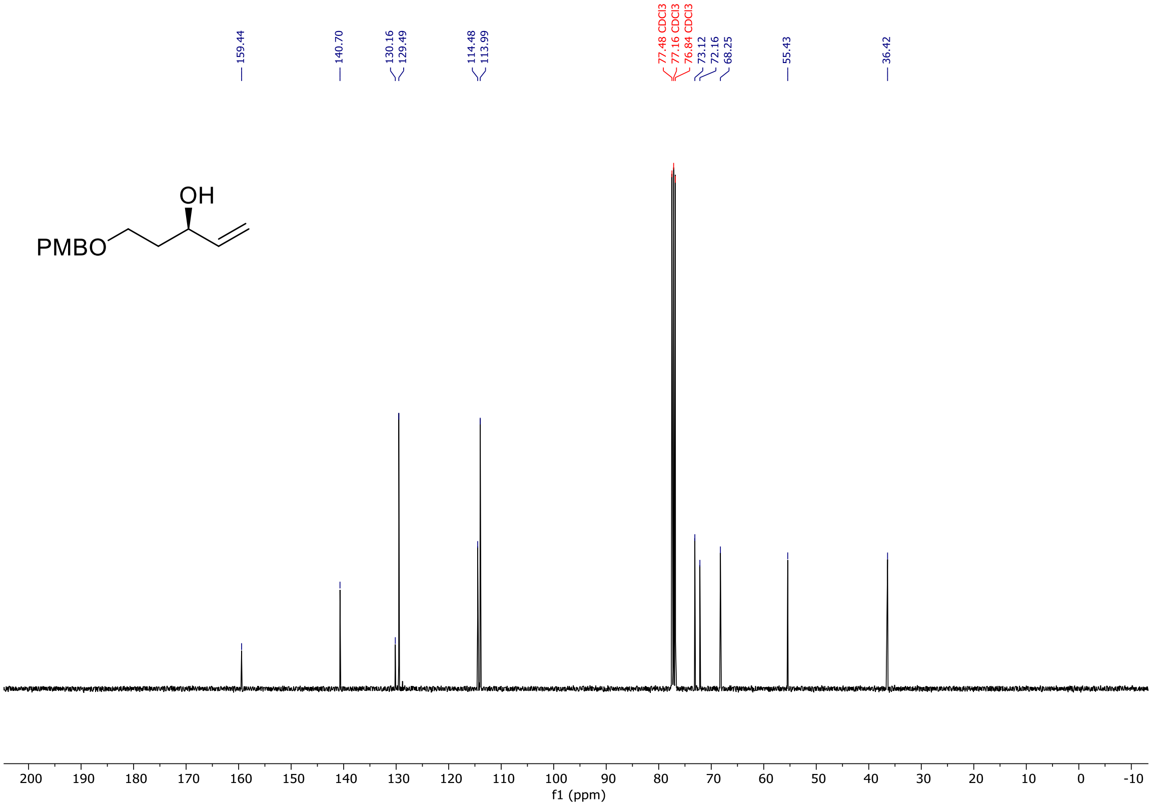

Compound S1: ^{1}H NMR ($CDCl_3$, 400 MHz)

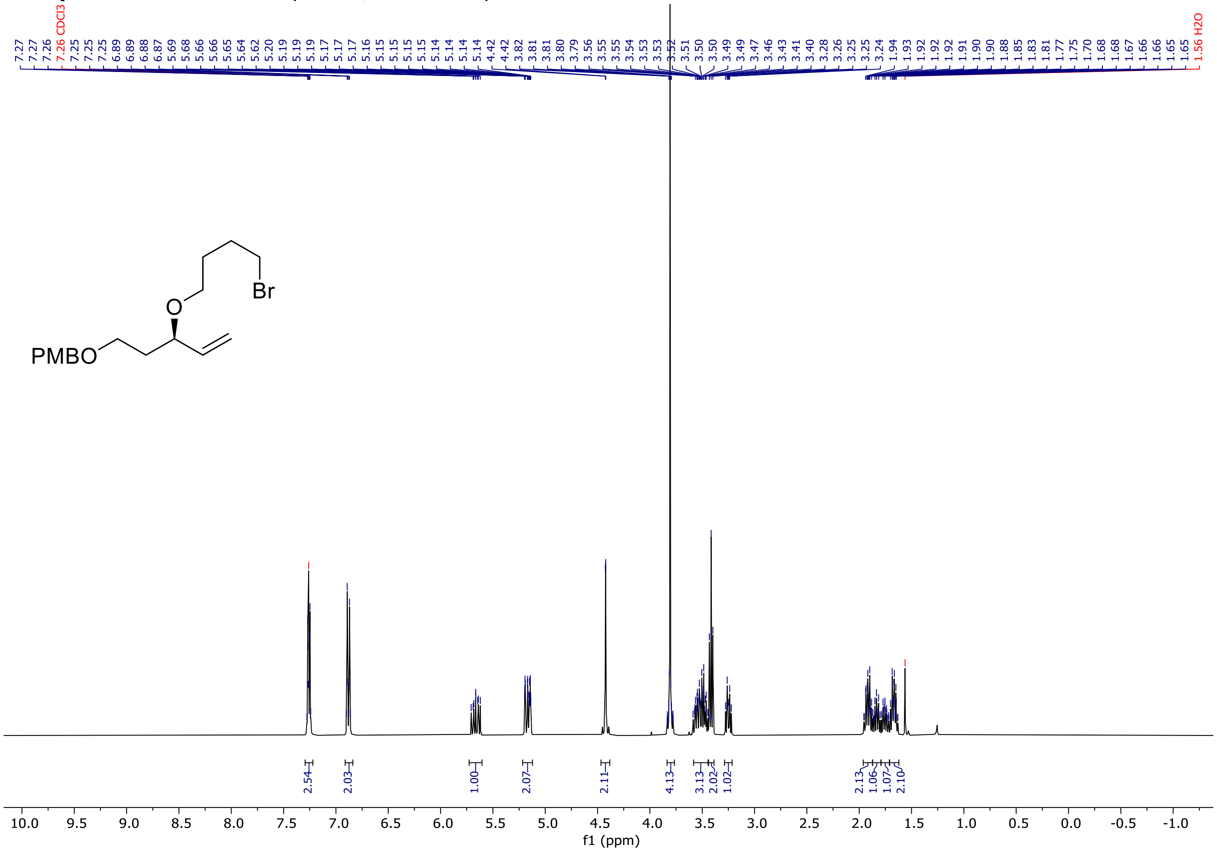

^{13}C NMR ($CDCl_3$, 101 MHz)

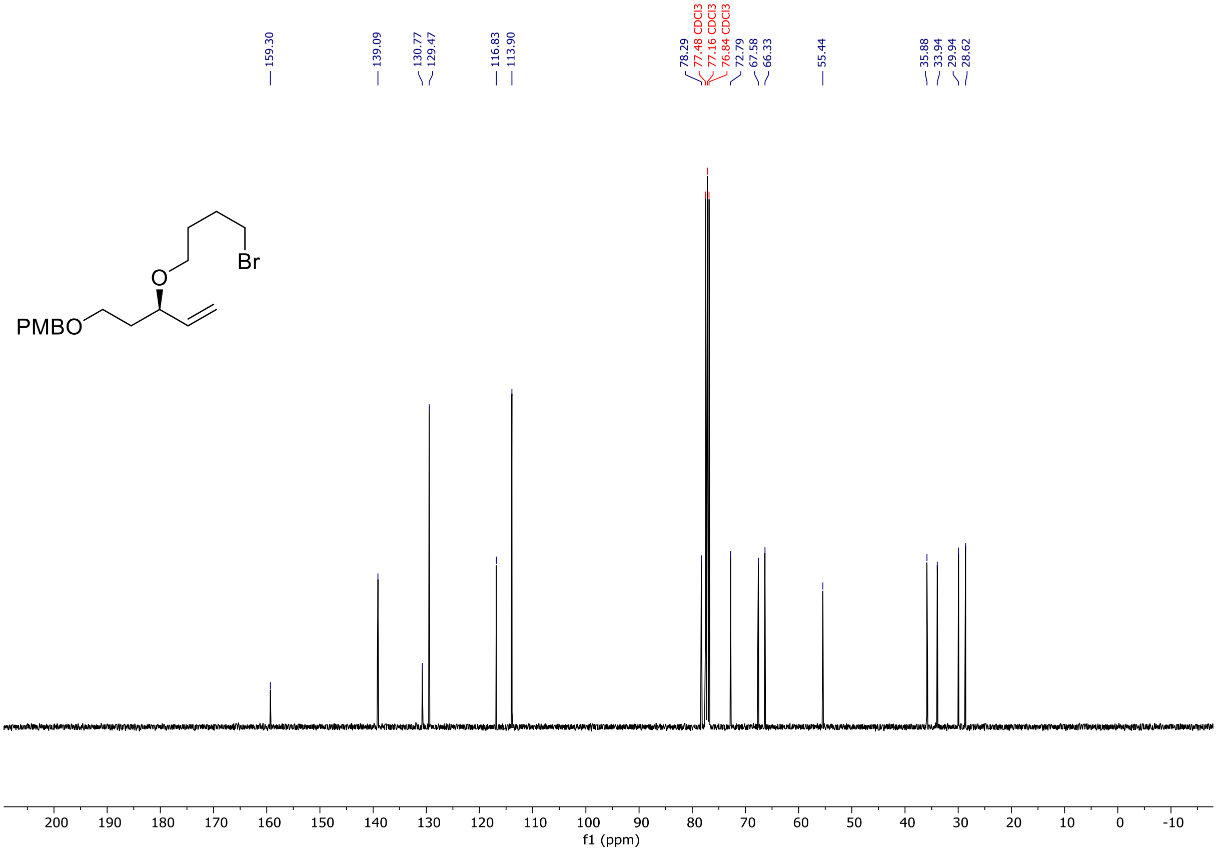

Compound 4: ^{1}H NMR ($CDCl_3$, 400 MHz)

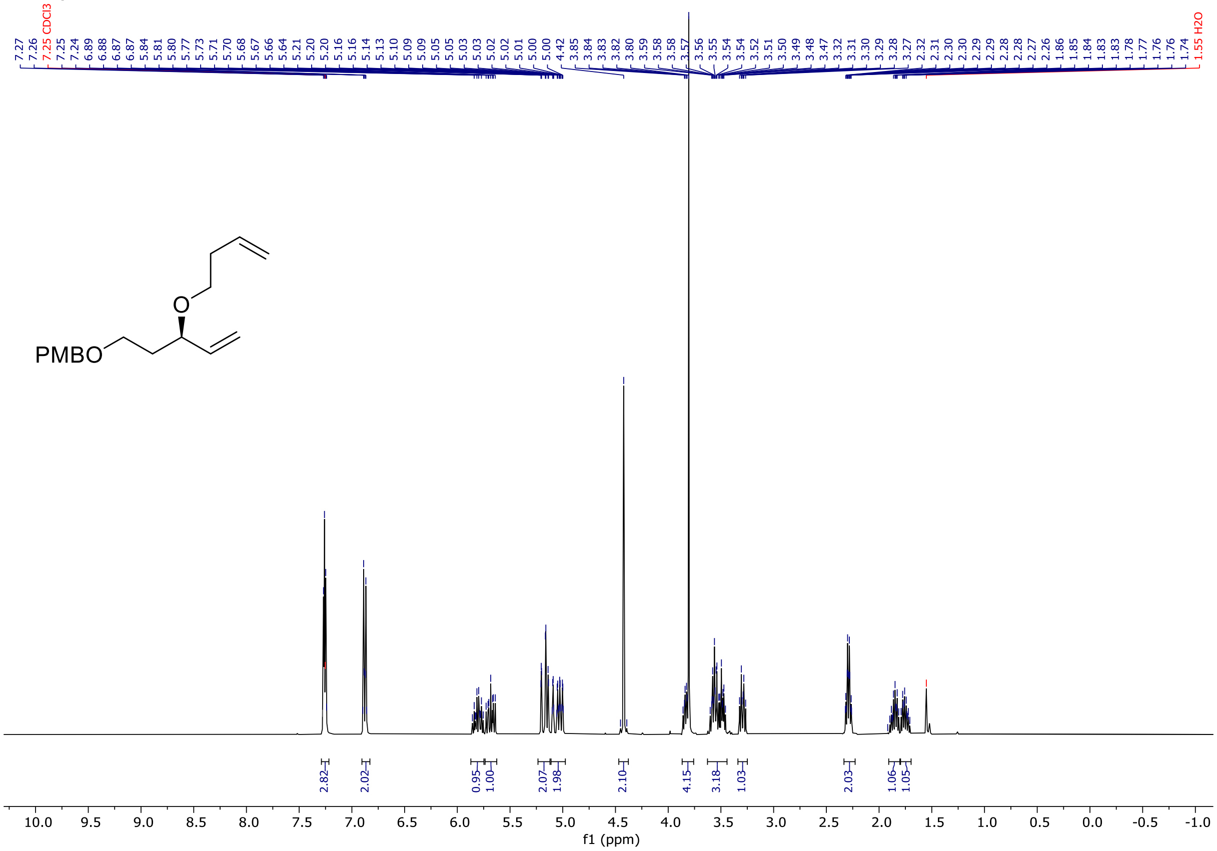

^{13}C NMR ($CDCl_3$, 101 MHz)

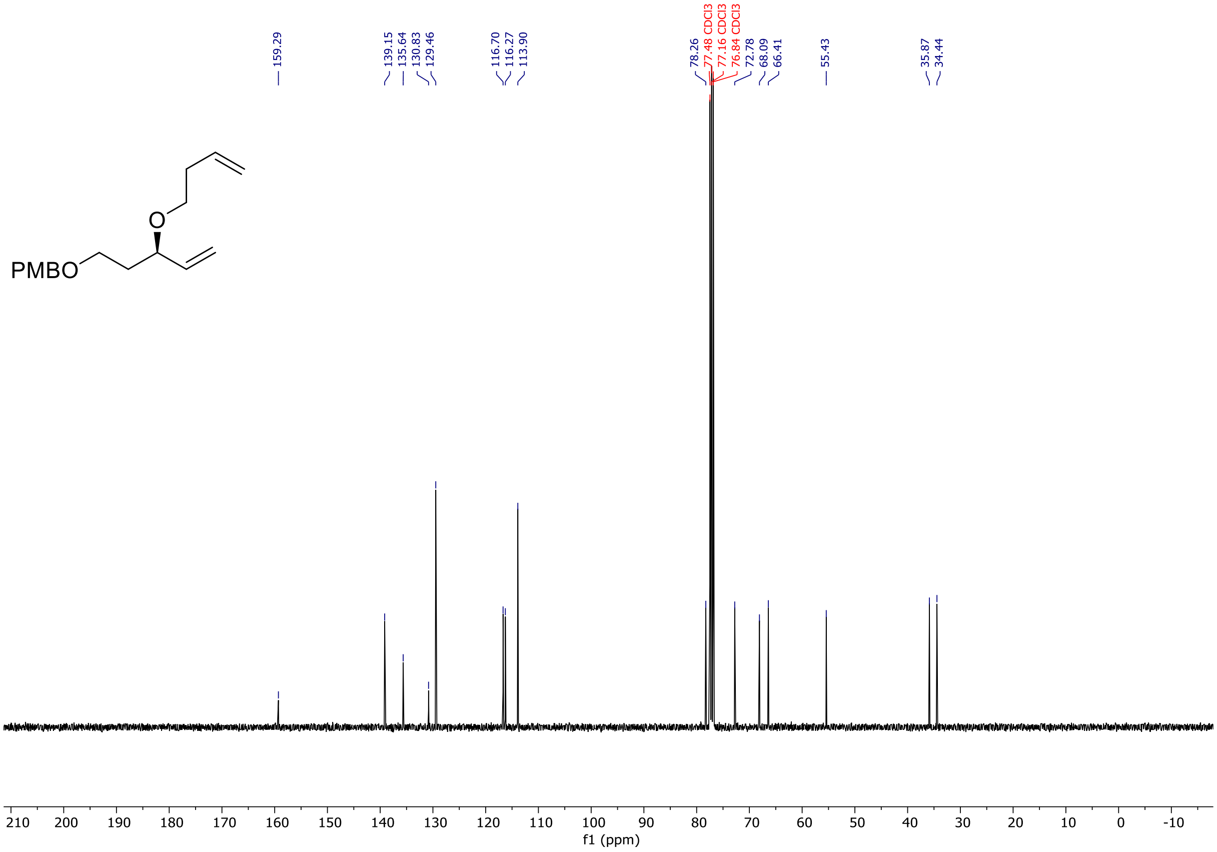

Compound S2: ^{1}H NMR ($CDCl_3$, 400 MHz)

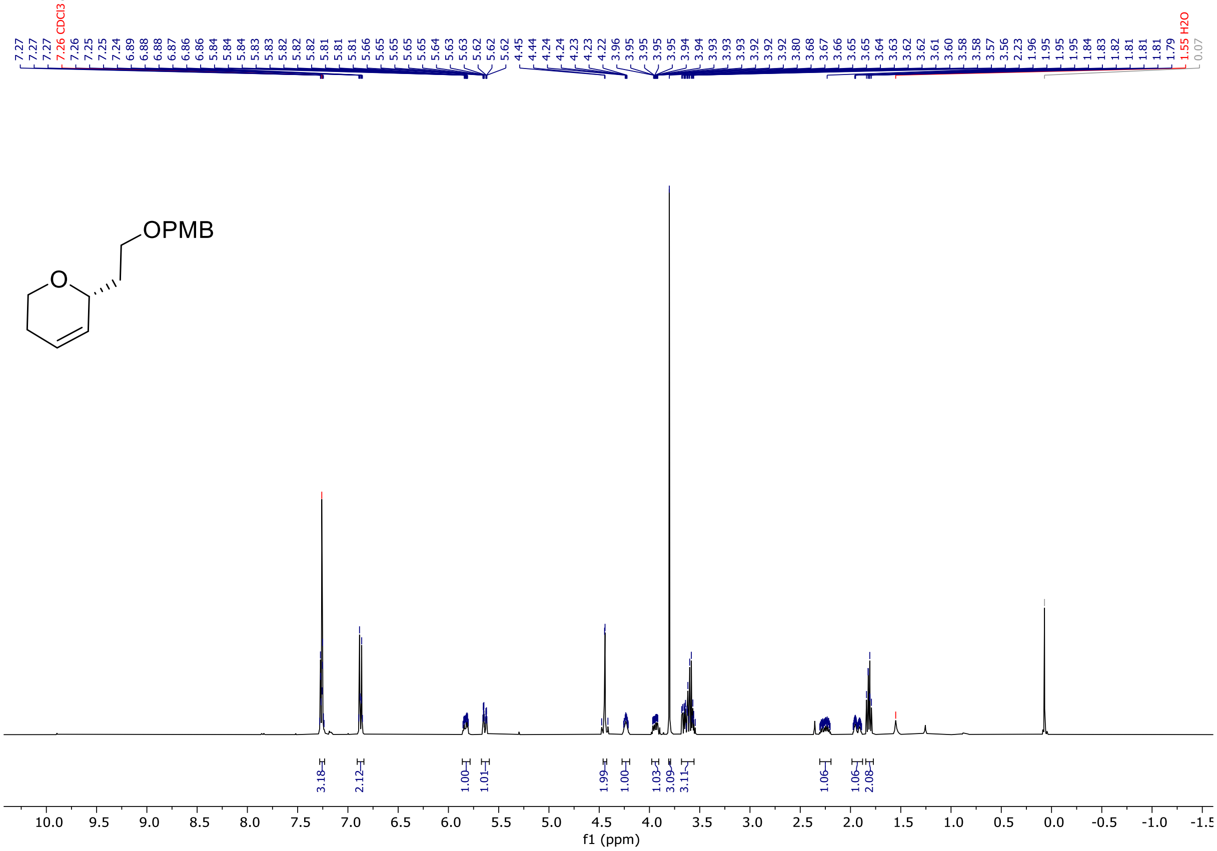

^{13}C NMR ($CDCl_3$, 101 MHz)

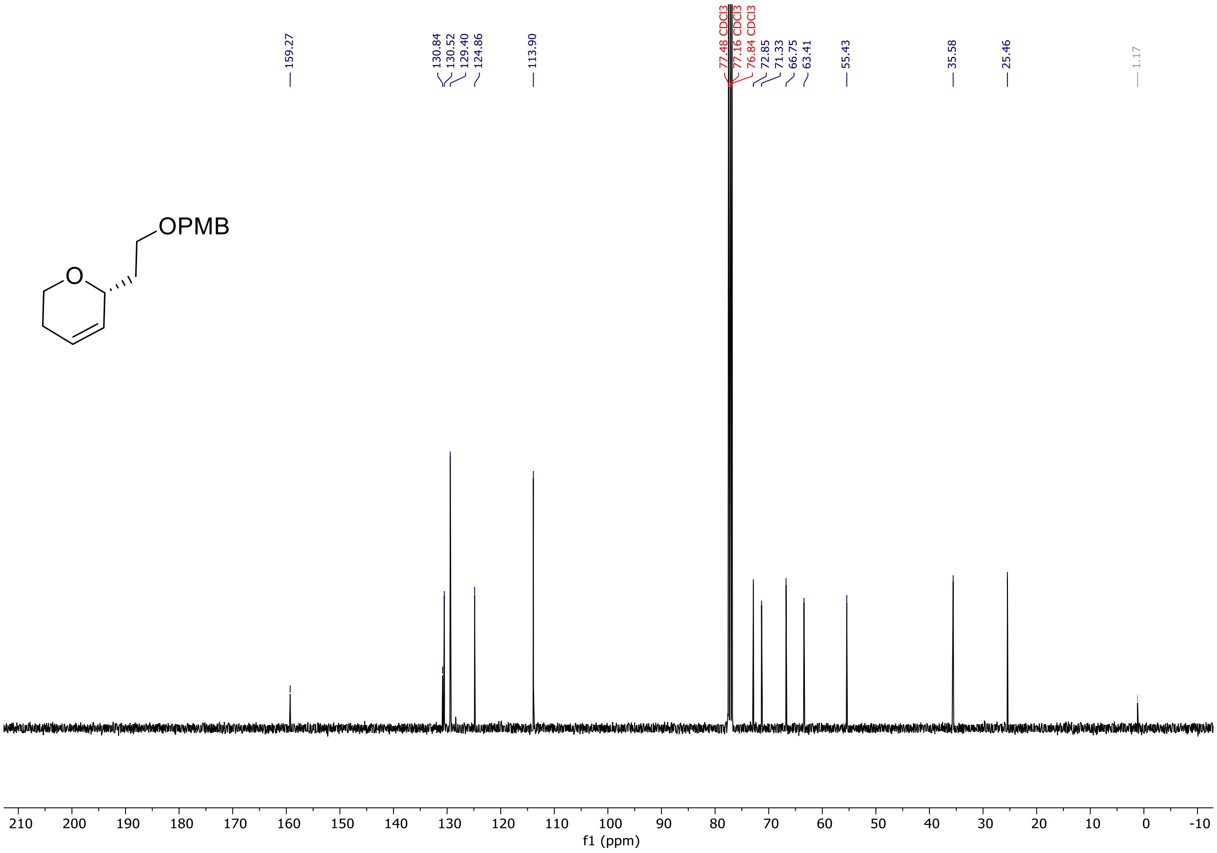

Compound 5: ^{1}H NMR ($CDCl_3$, 400 MHz)

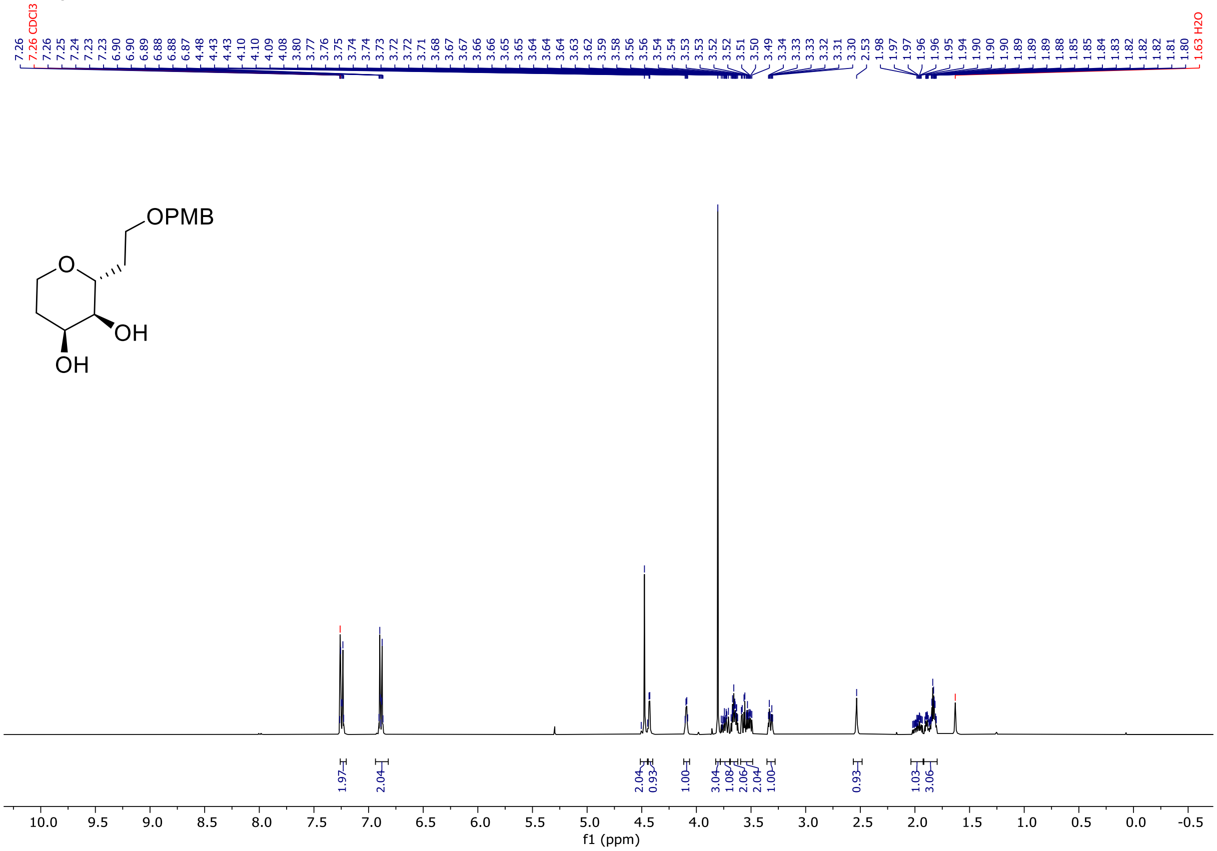

^{13}C NMR ($CDCl_3$, 101 MHz)

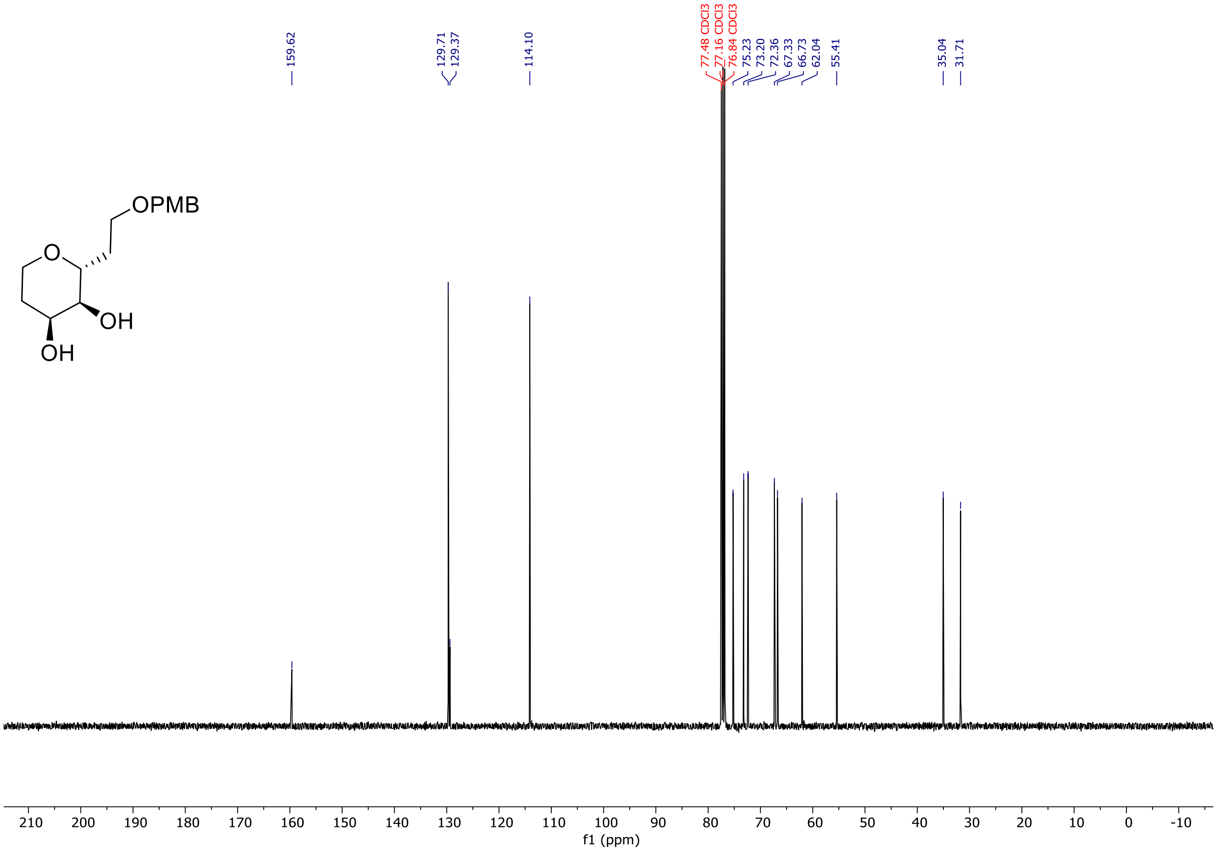

Compound S3: ^{1}H NMR ($CDCl_3$, 400 MHz)

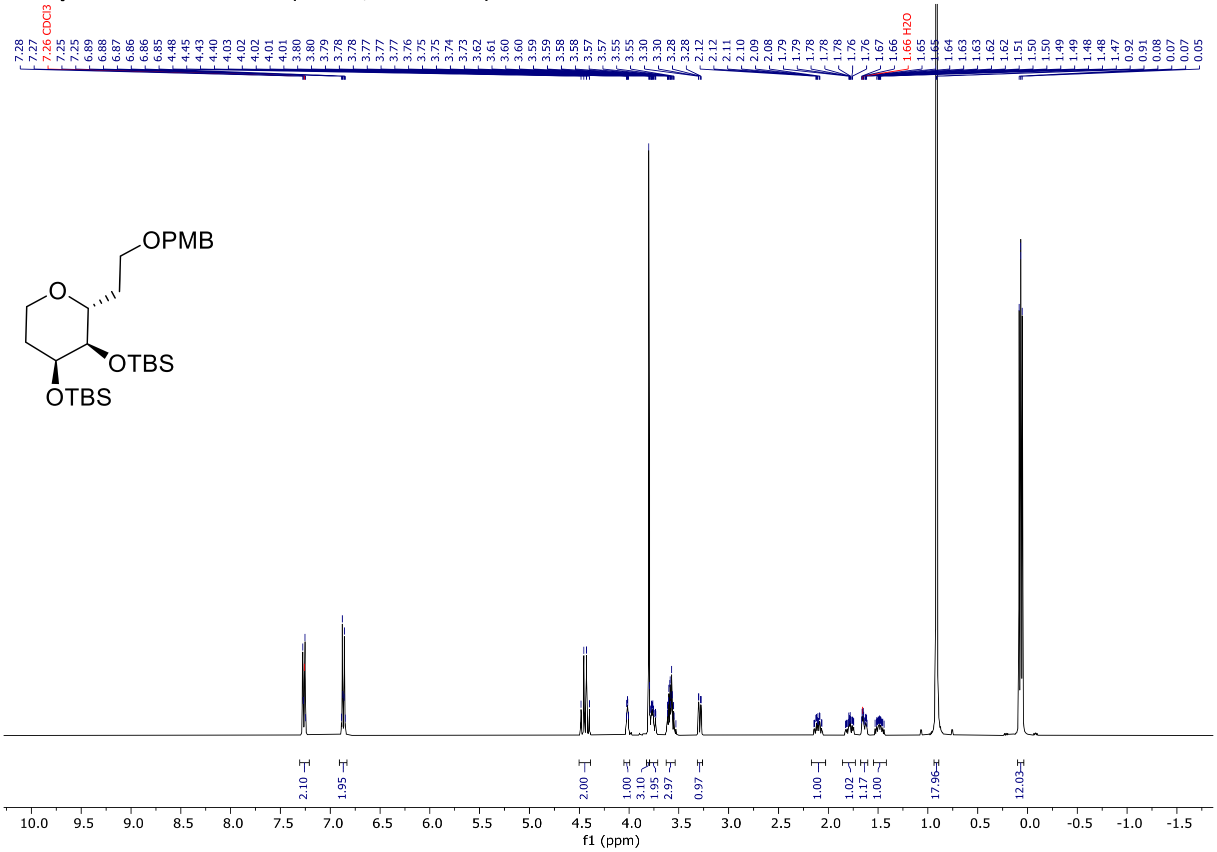

^{13}C NMR ($CDCl_3$, 101 MHz)

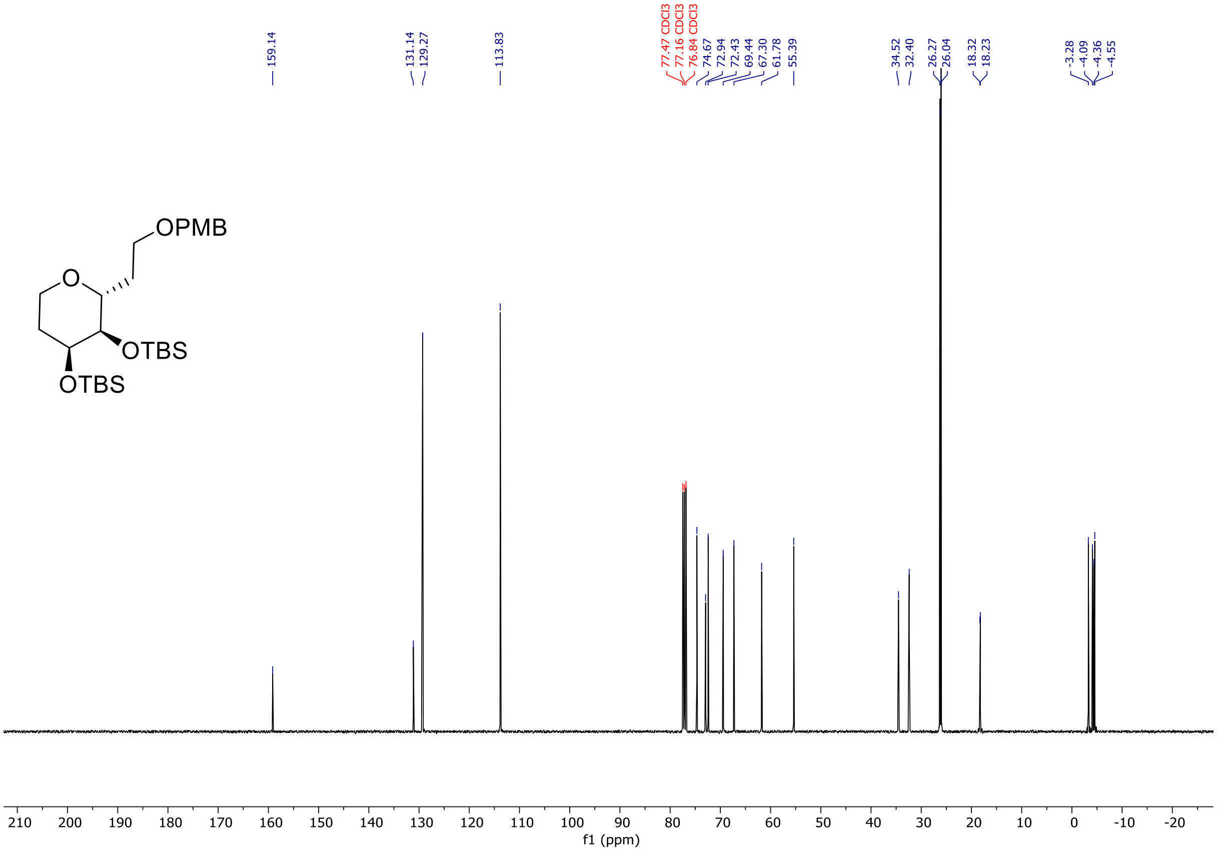

Compound S4: ^{1}H NMR (400 MHz, $CDCl_3$)

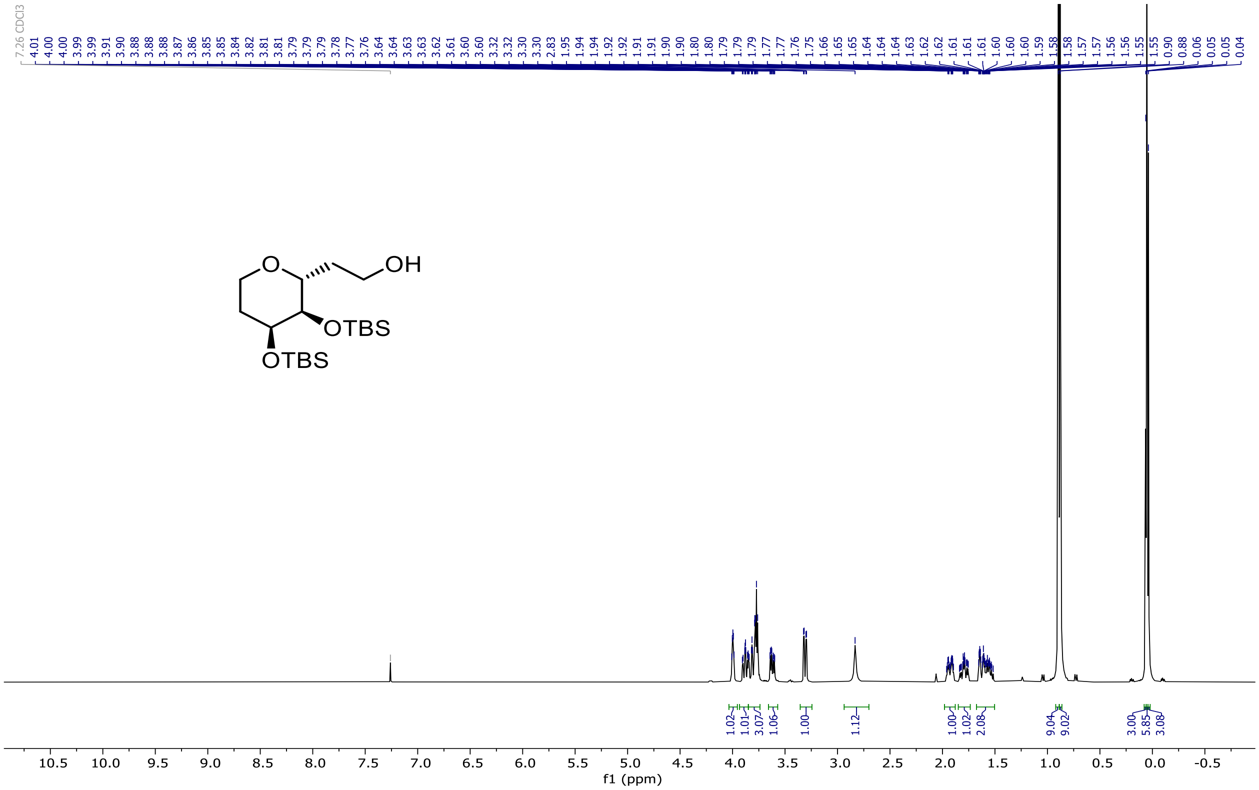

^{13}C NMR (101 MHz, $CDCl_3$)

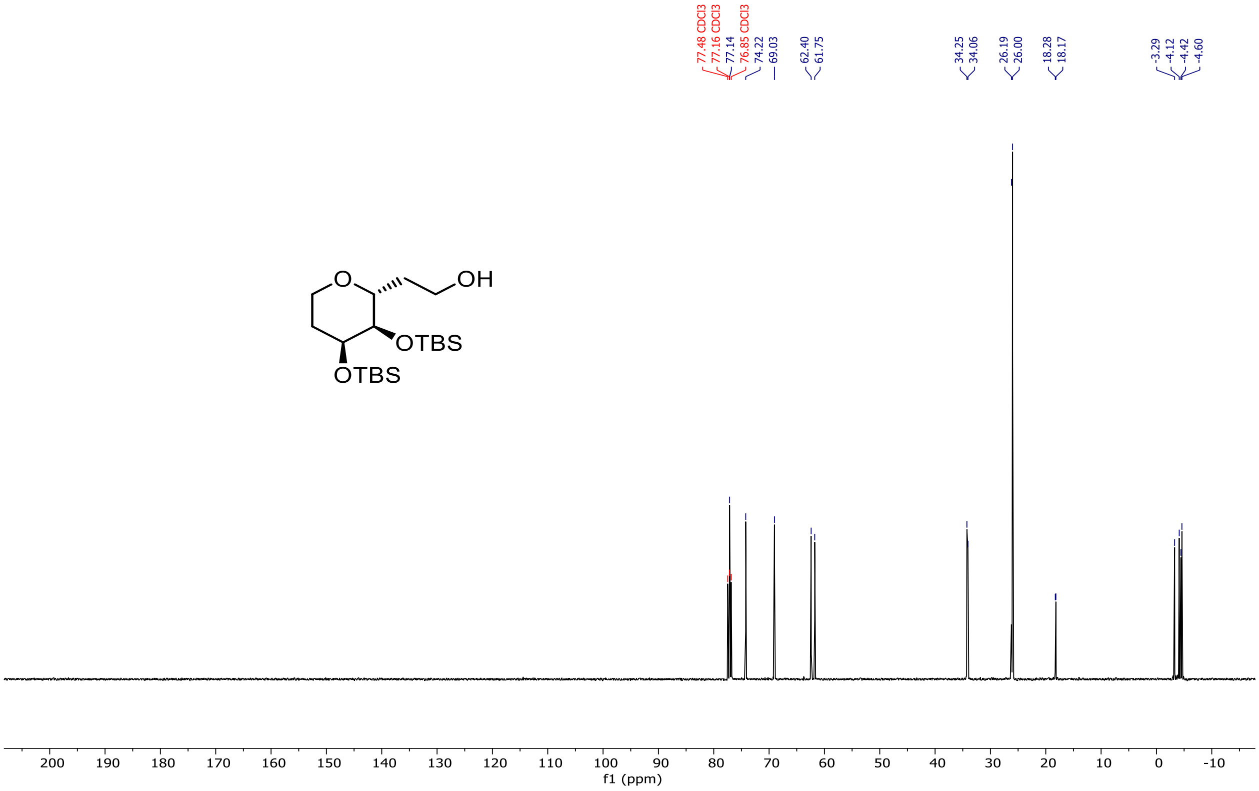

Compound 6: ^{1}H NMR (400 MHz, $CDCl_3$)

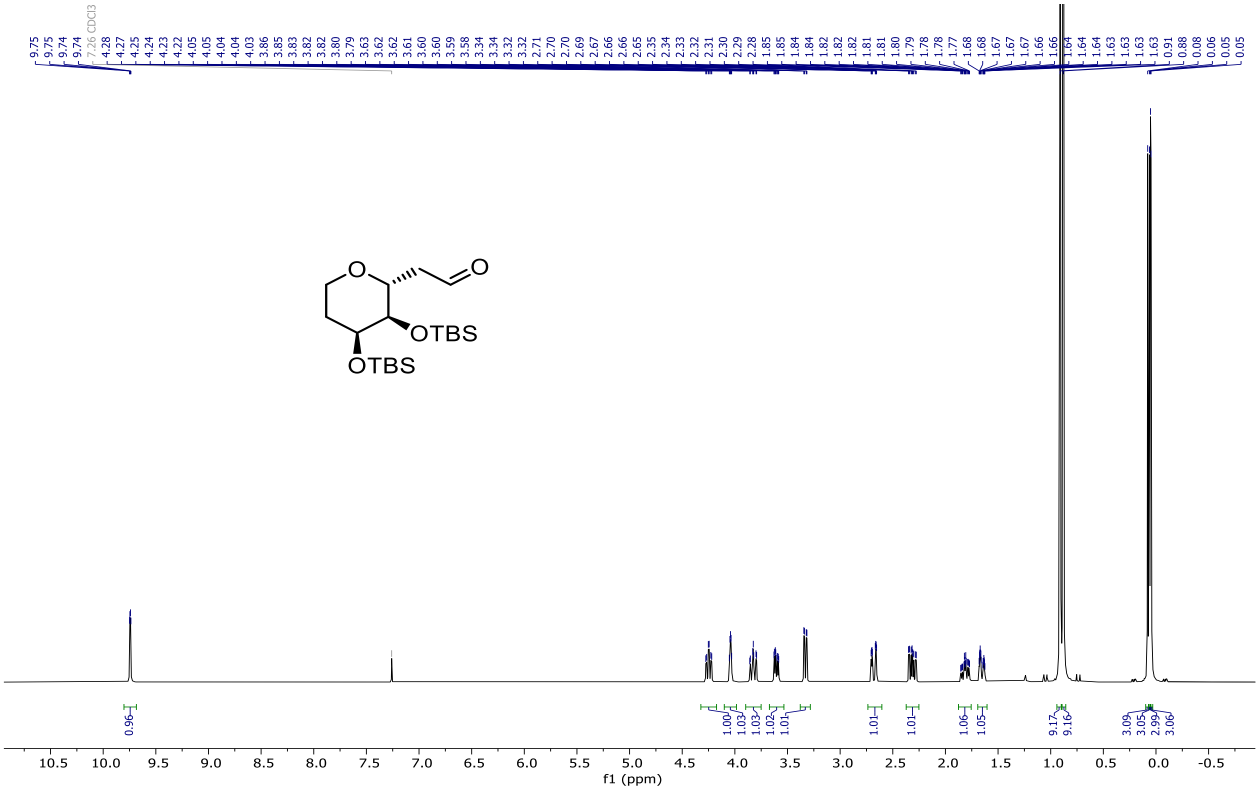

^{13}C NMR (101 MHz, $CDCl_3$)

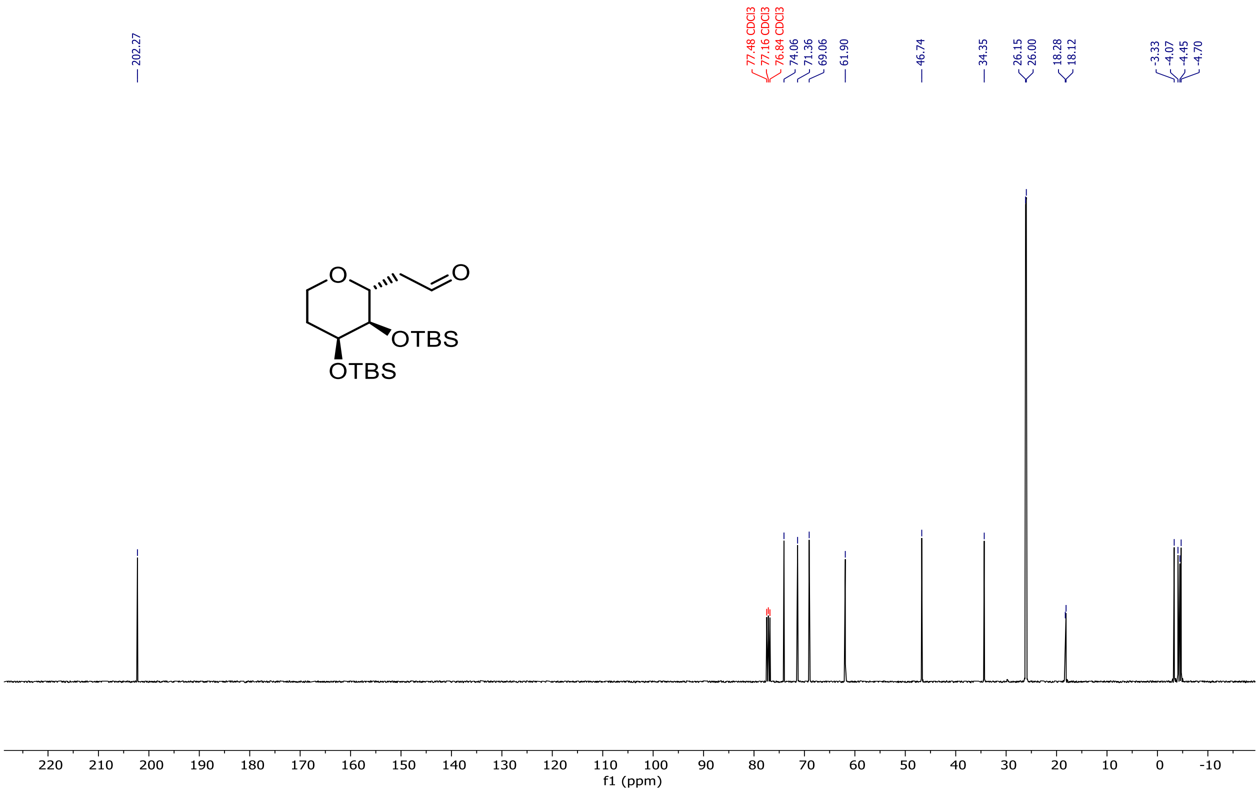

Compound 7: ^{1}H NMR (400 MHz, $CDCl_3$)

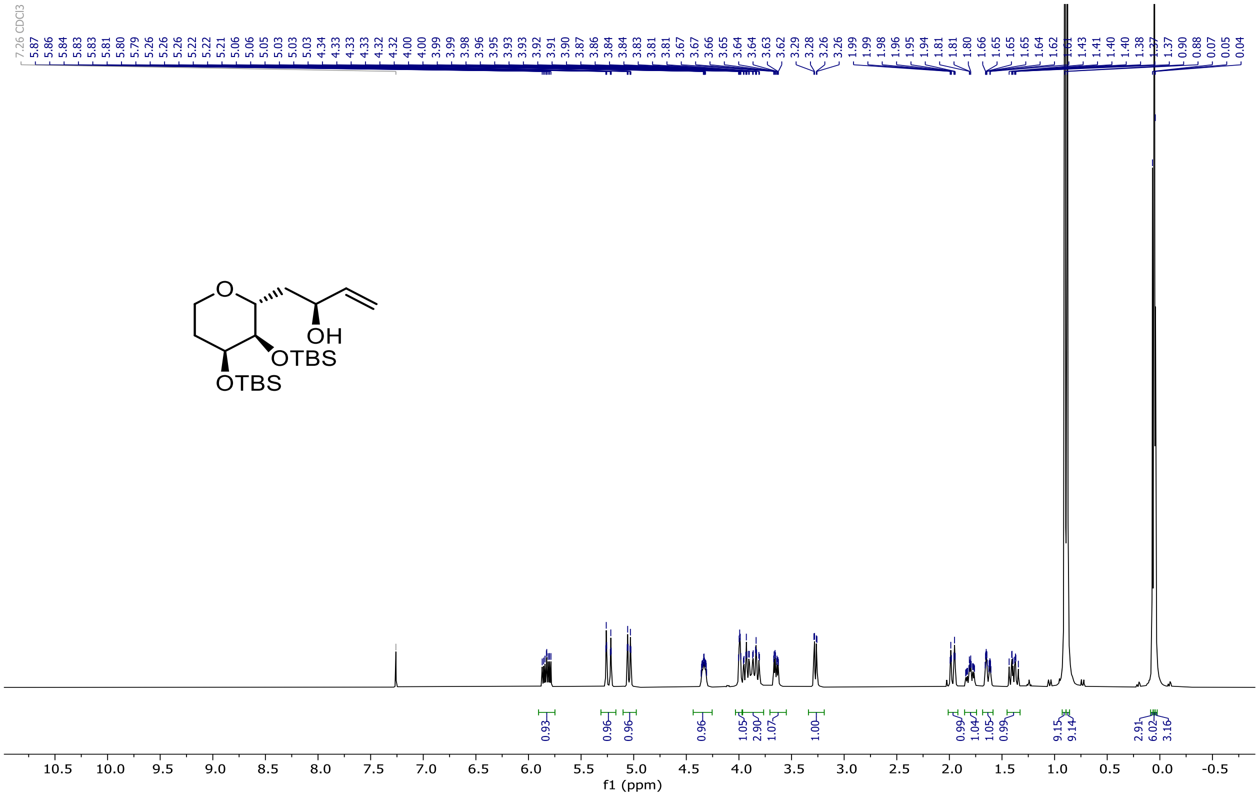

^{13}C NMR (101 MHz, $CDCl_3$)

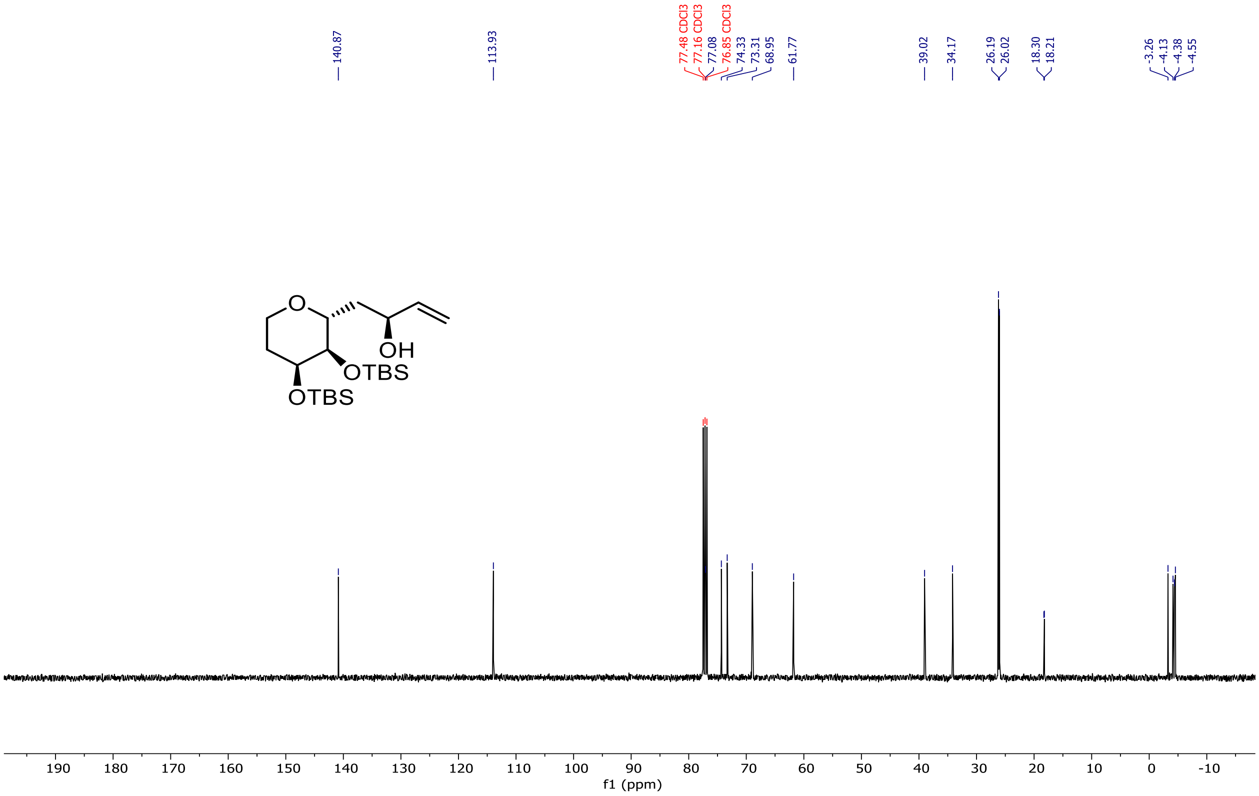

(*S*)-Mosher ester derived from compound 7: ^{1}H NMR (400 MHz, $CDCl_3$)

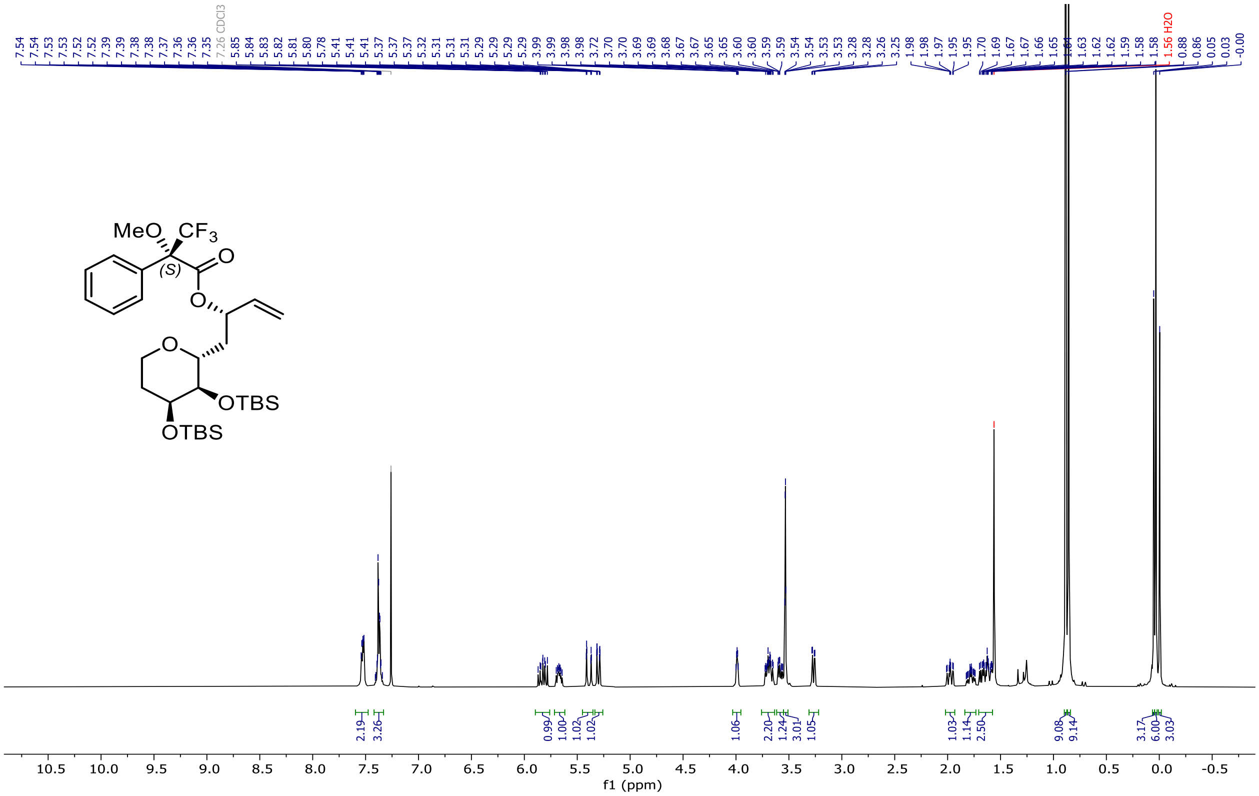

^{13}C NMR (101 MHz, $CDCl_3$)

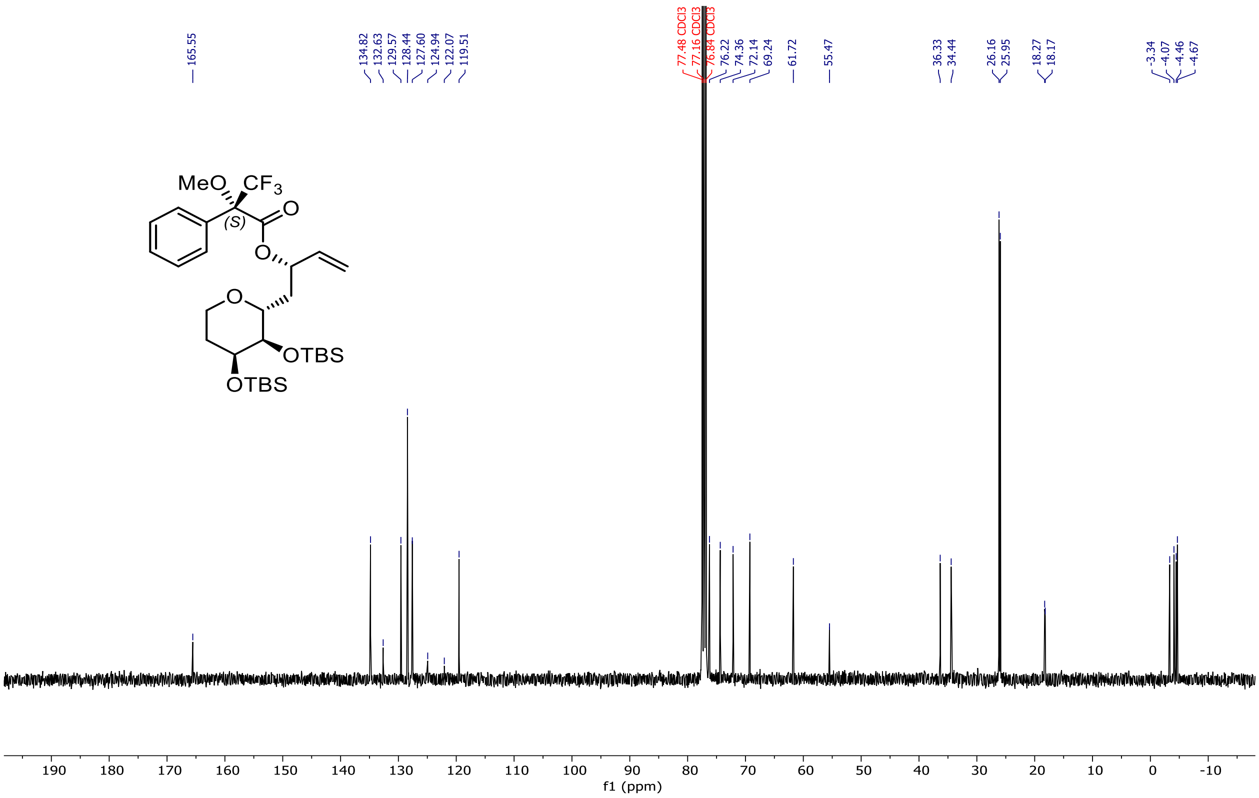

(*S*)-Mosher ester derived from compound 7: ^{1}H-^{1}H COSY ($CDCl_3$)

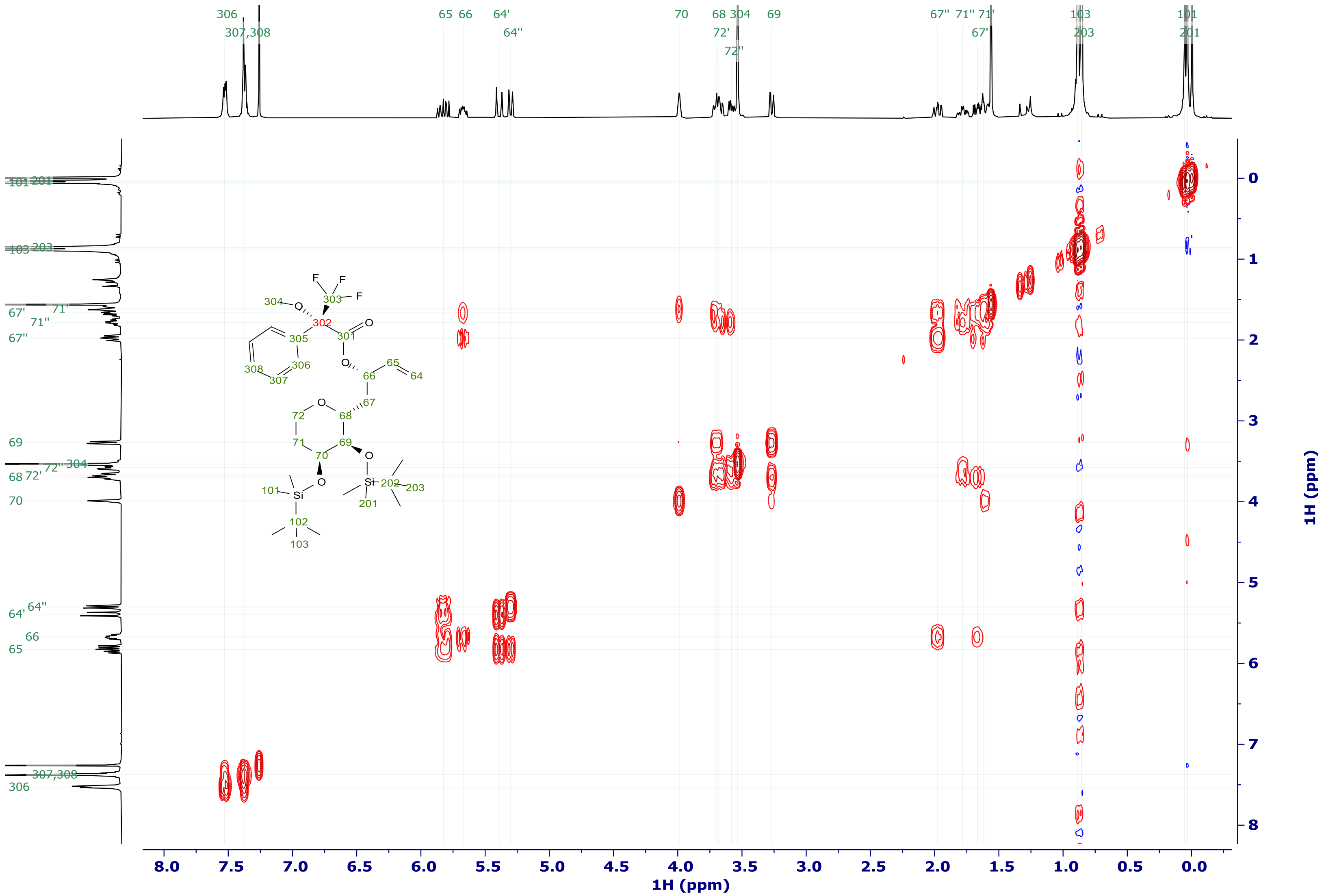

(*S*)-Mosher ester derived from compound 7: HSQC NMR ($CDCl_3$)

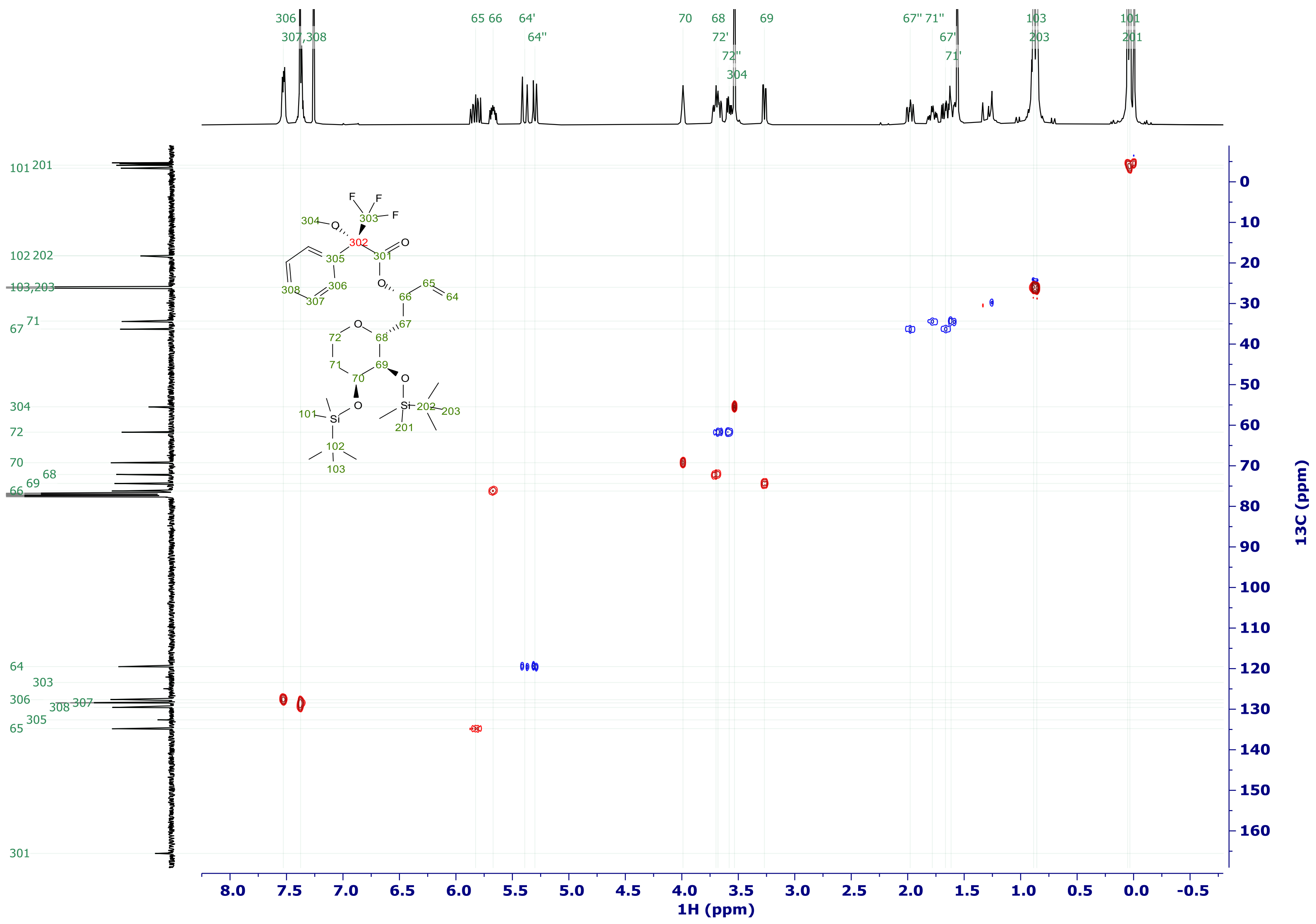

(*S*)-Mosher ester derived from compound 7: HMBC NMR ($CDCl_3$)

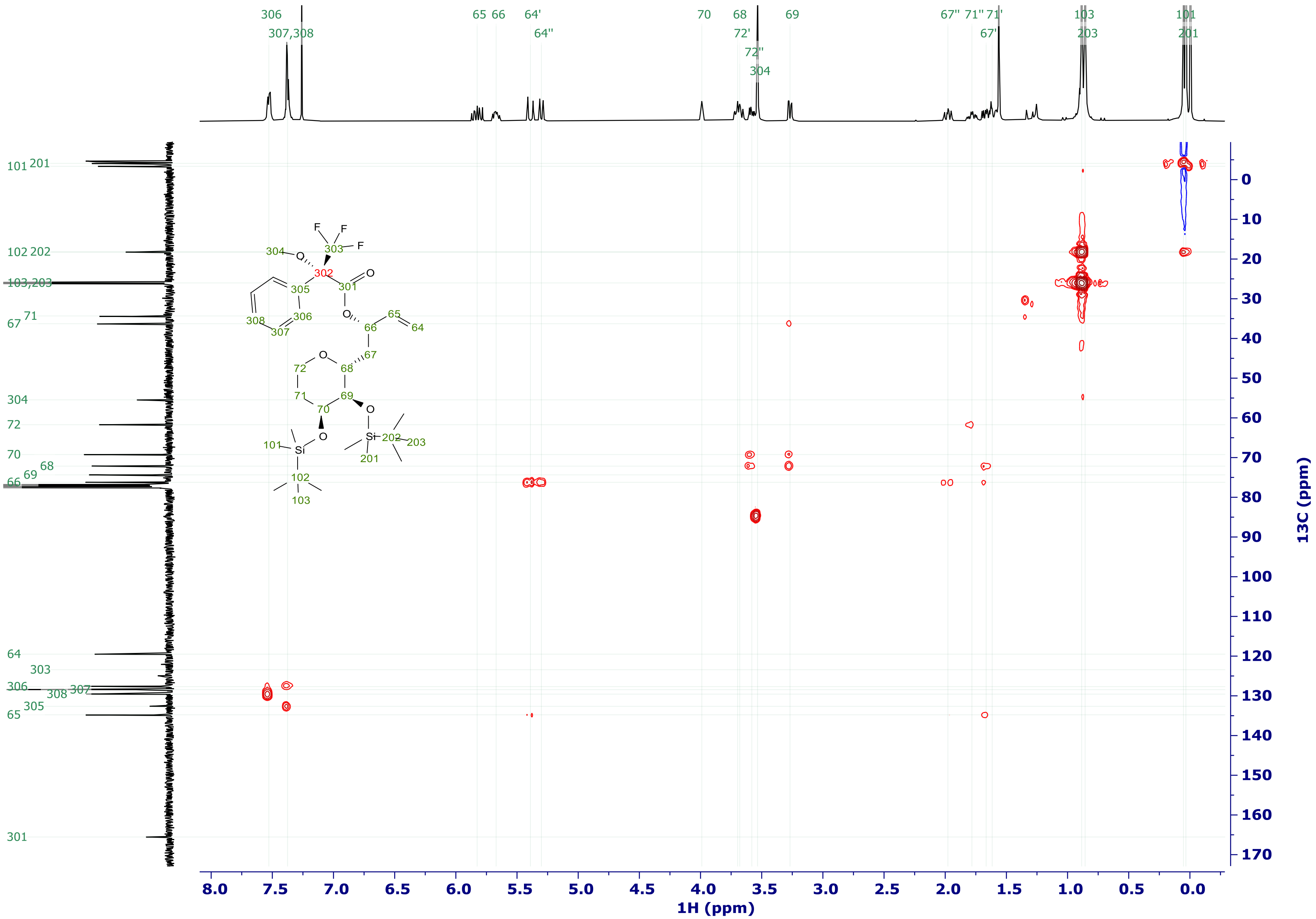

(*R*)-Mosher ester derived from compound 7: ^{1}H NMR (400 MHz, $CDCl_3$)

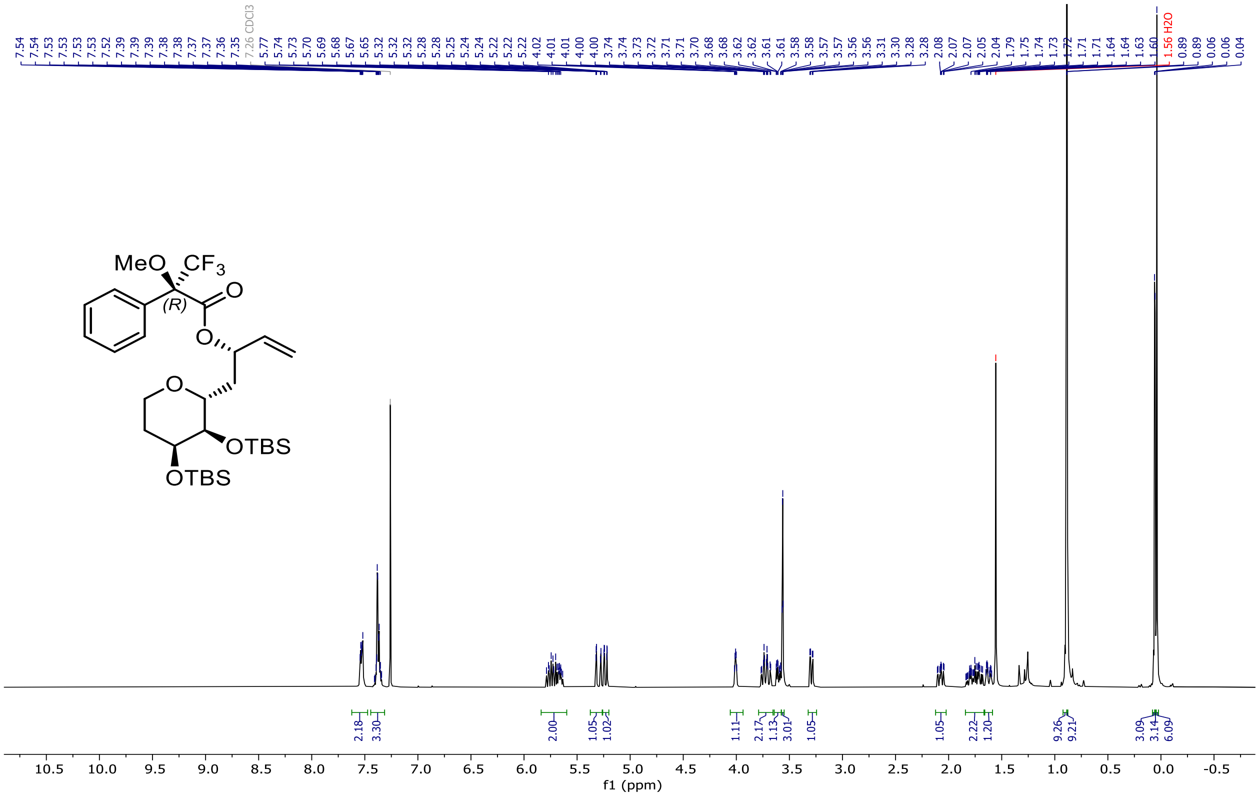

(*R*)-Mosher ester derived from compound 7: ^{13}C NMR (101 MHz, $CDCl_3$)

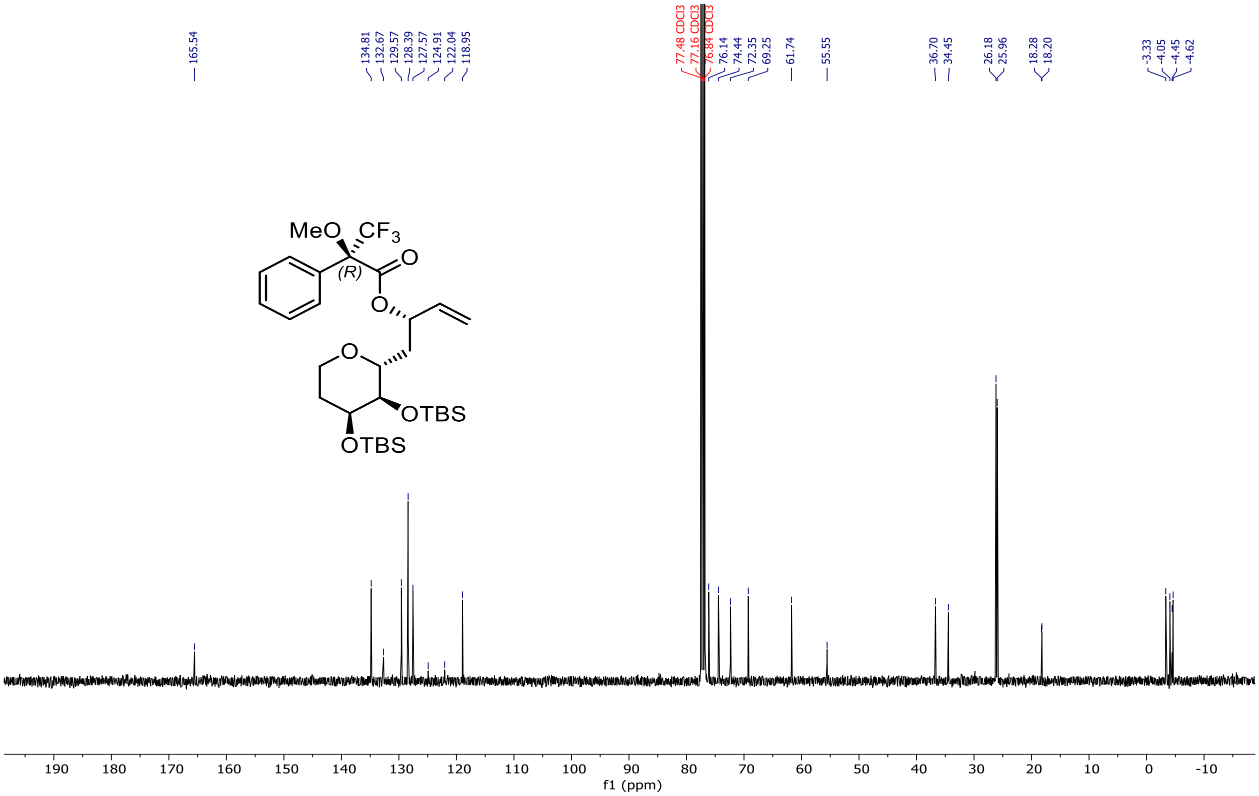

(*R*)-Mosher ester derived from compound 7: ^{1}H-^{1}H COSY ($CDCl_3$)

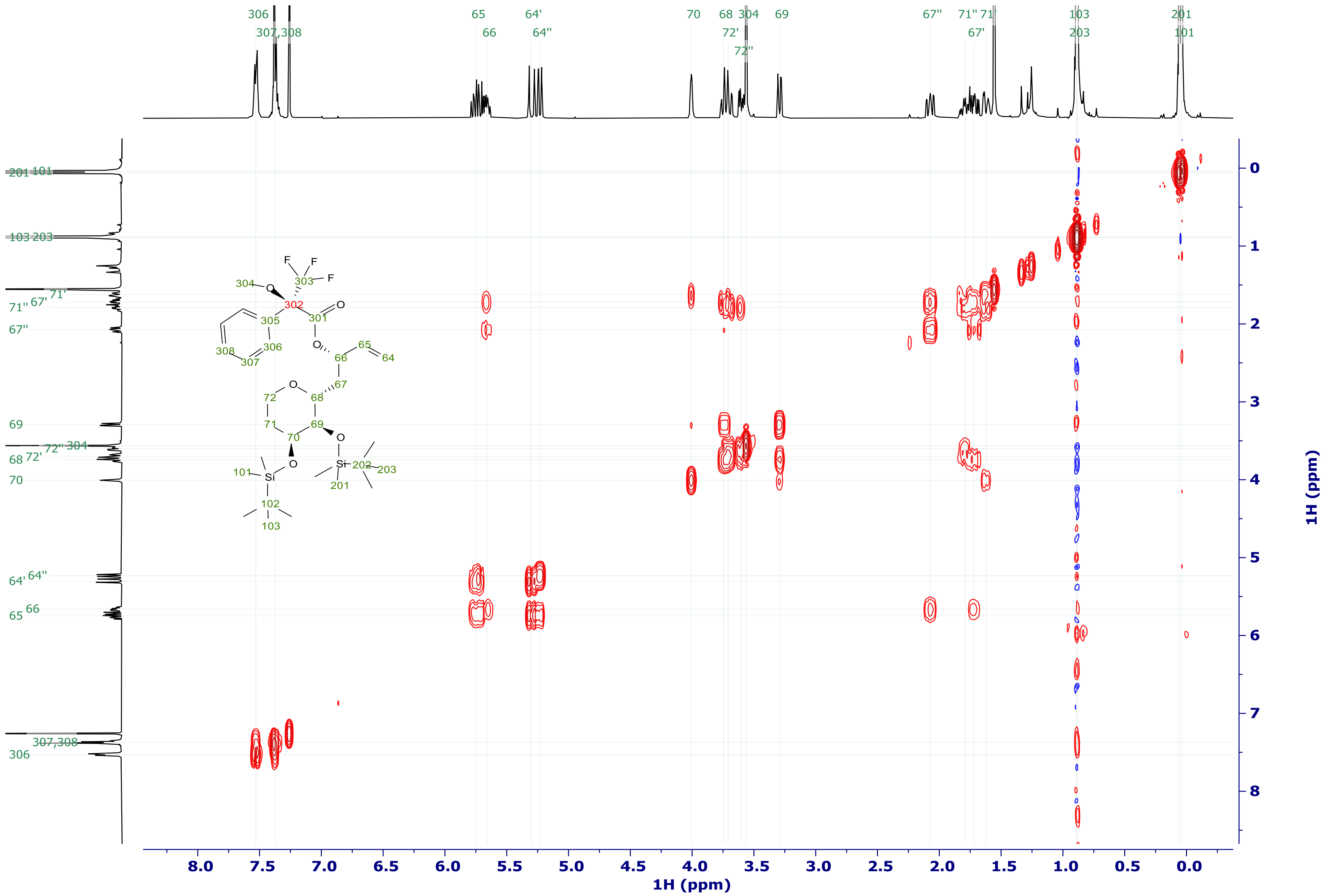

(*R*)-Mosher ester derived from compound 7: HSQC NMR ($CDCl_3$)

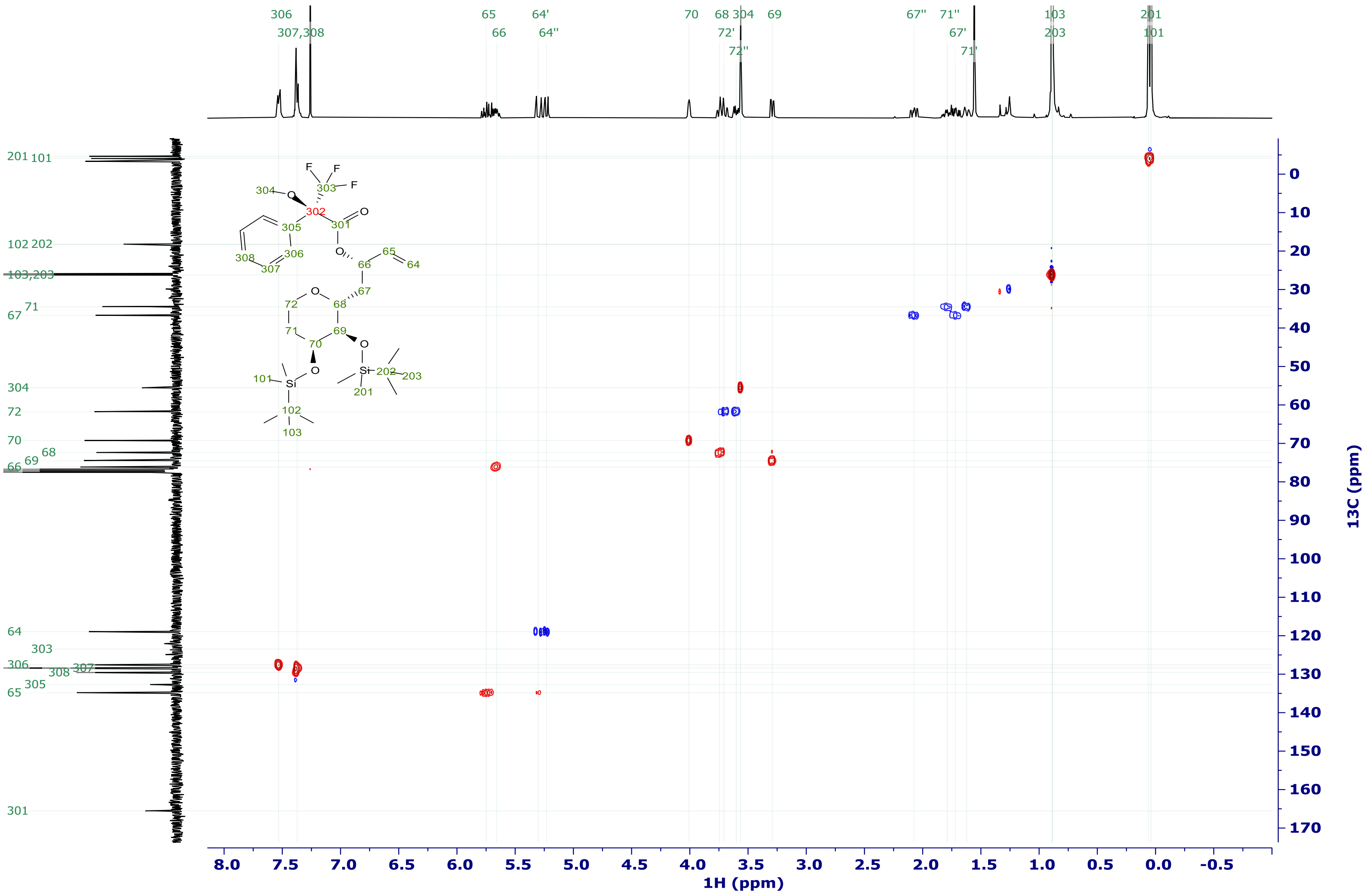

(*R*)-Mosher ester derived from compound 7: HMBC NMR ($CDCl_3$)

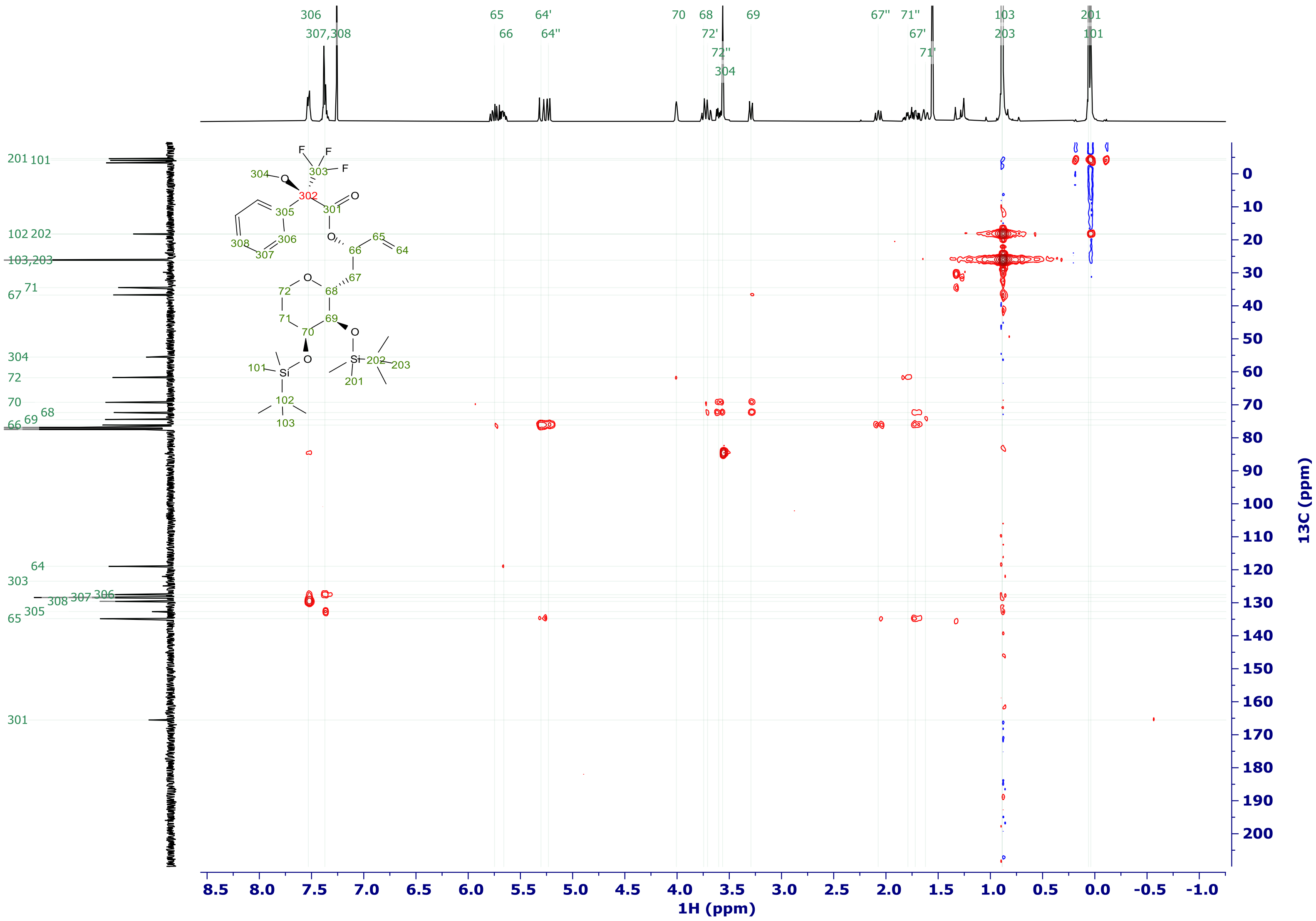

Compound 8: ^{1}H NMR (400 MHz, $CDCl_3$)

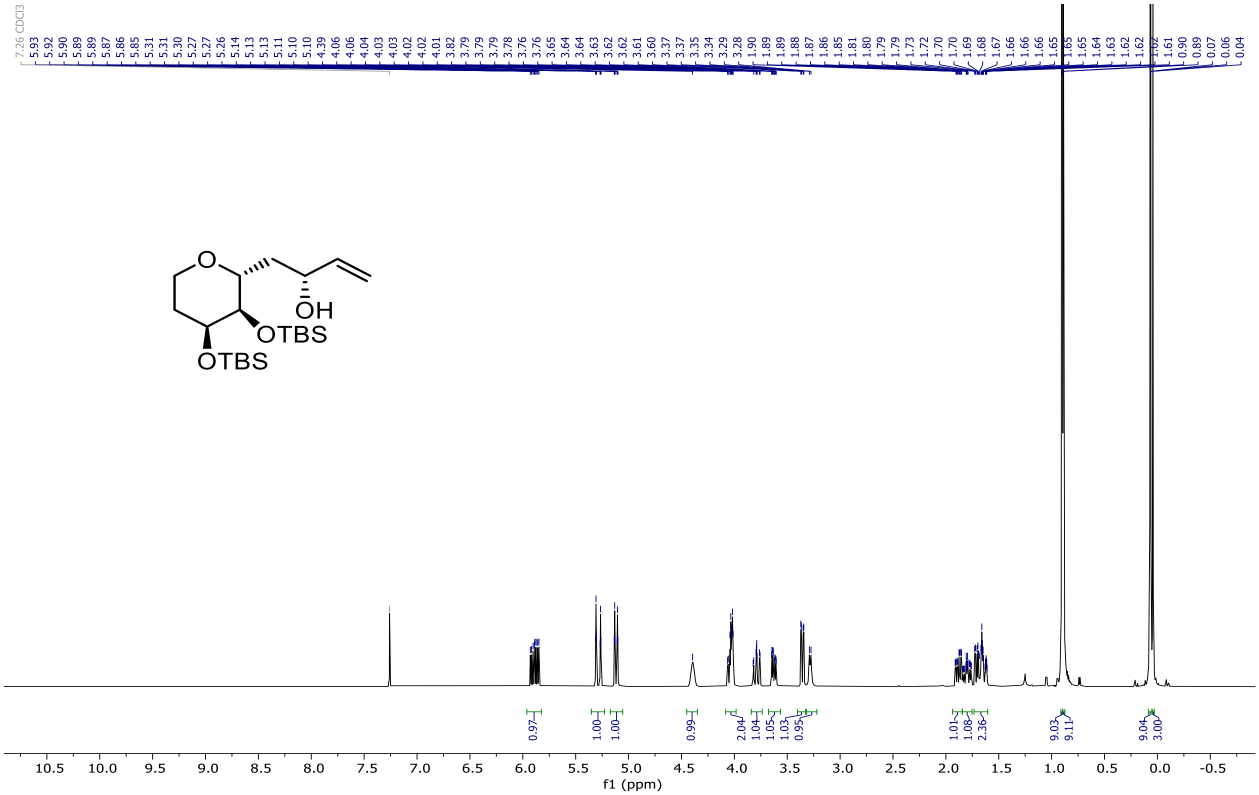

^{13}C NMR (101 MHz, $CDCl_3$)

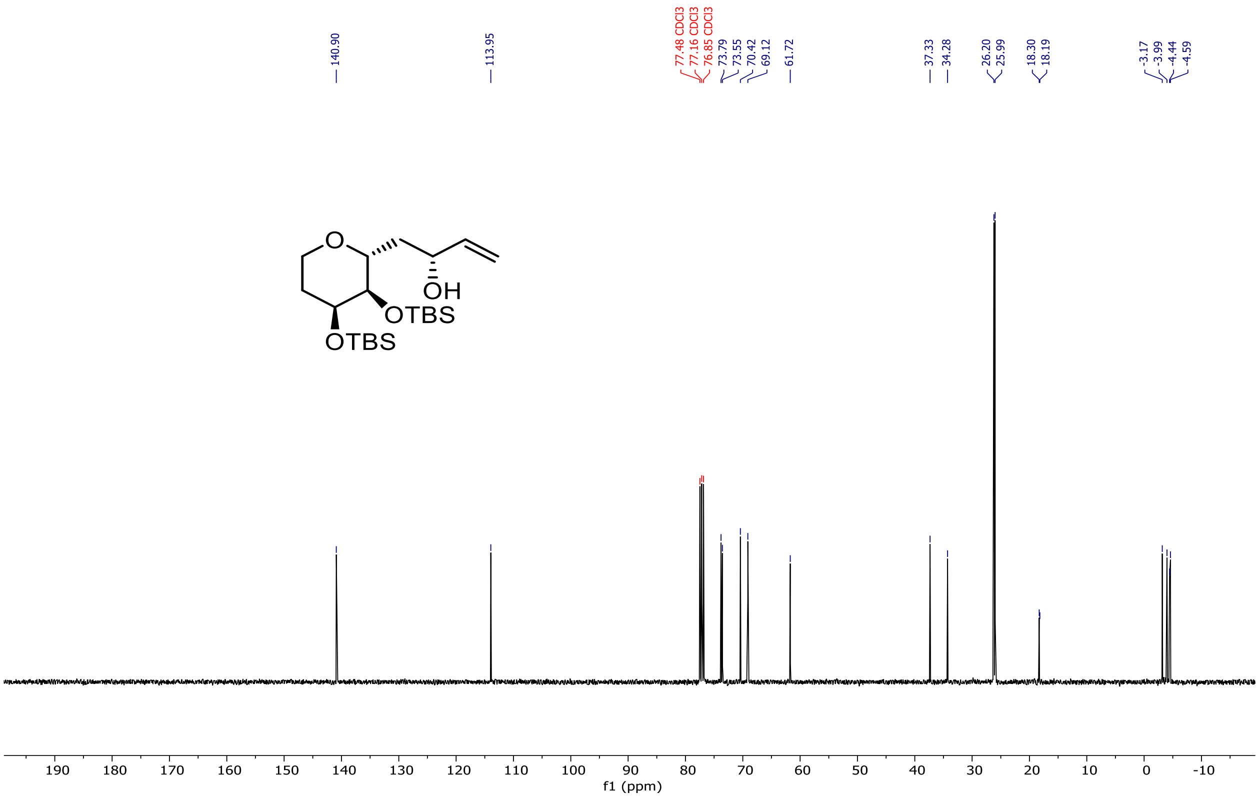

Compound 8a: ^{1}H NMR (400 MHz, $CDCl_3$)

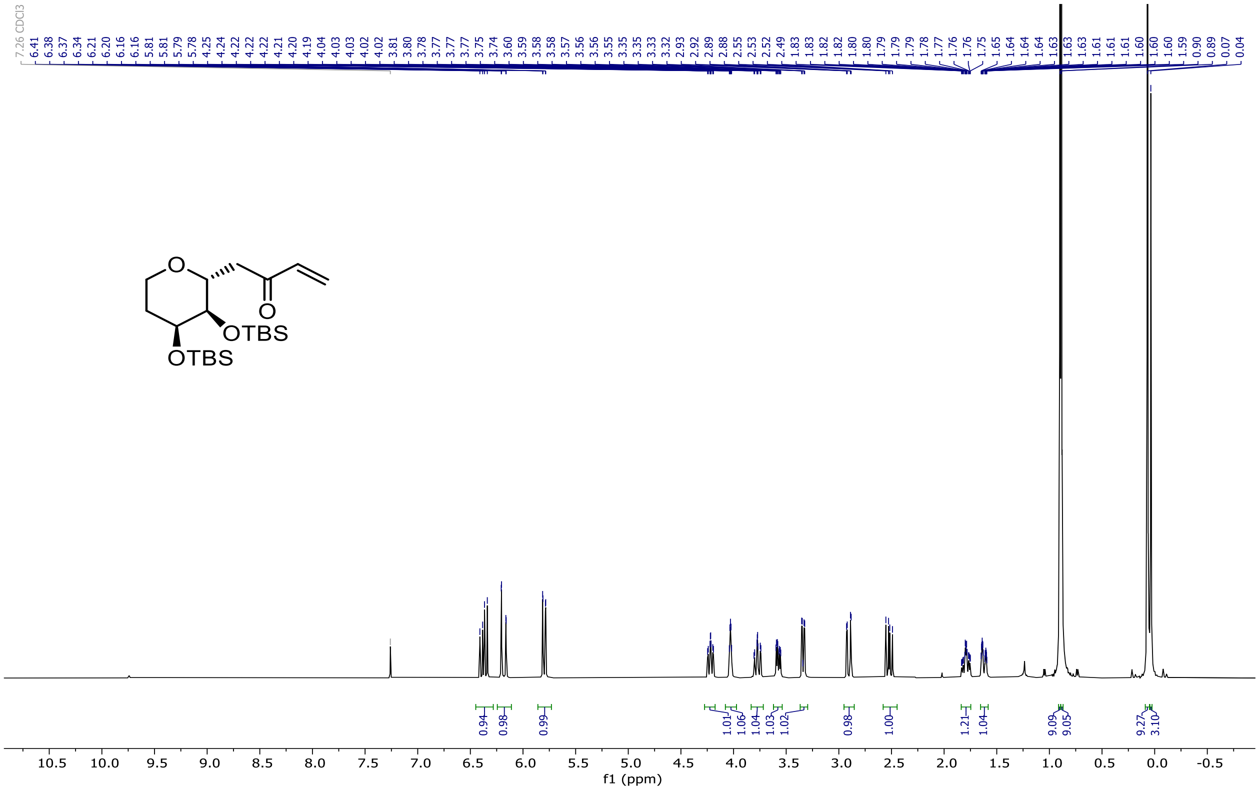

^{13}C NMR (101 MHz, $CDCl_3$)

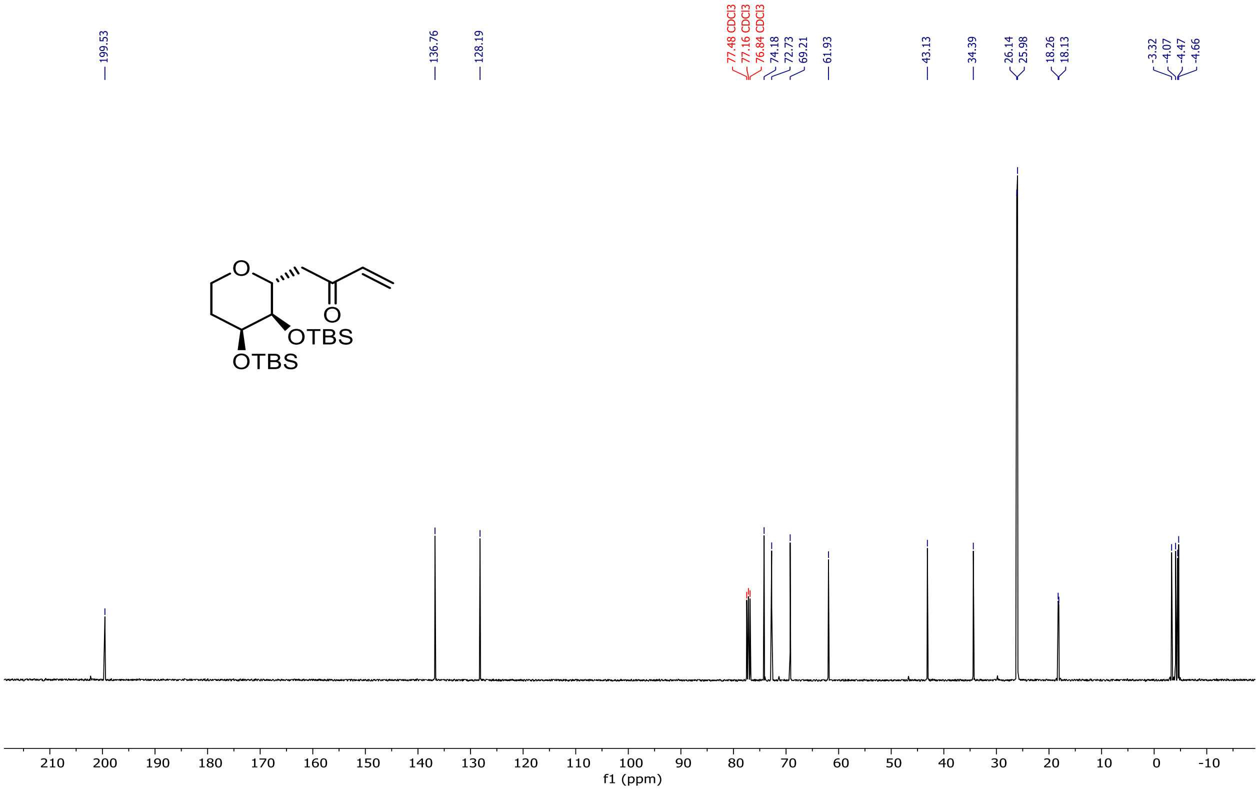

Compound 9: ^{1}H NMR ($CDCl_3$, 400 MHz)

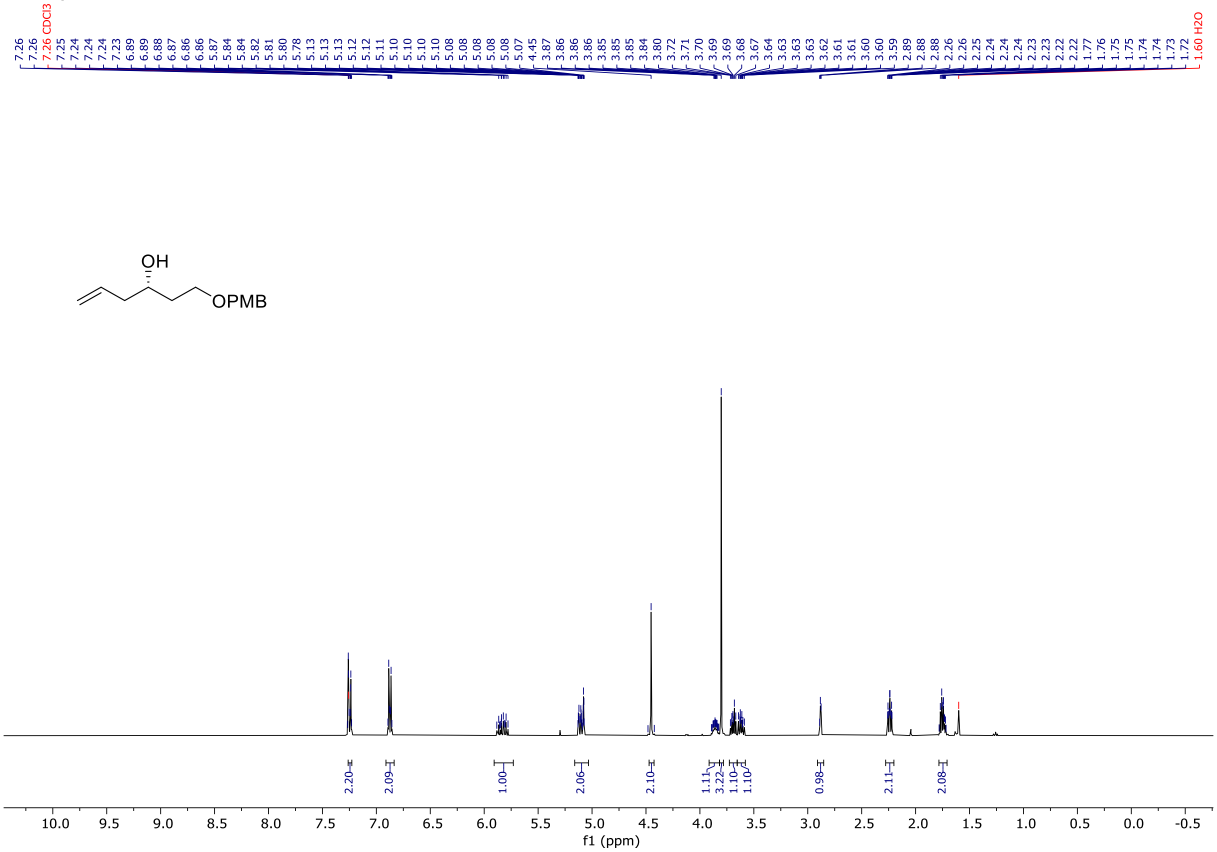

^{13}C NMR ($CDCl_3$, 101 MHz)

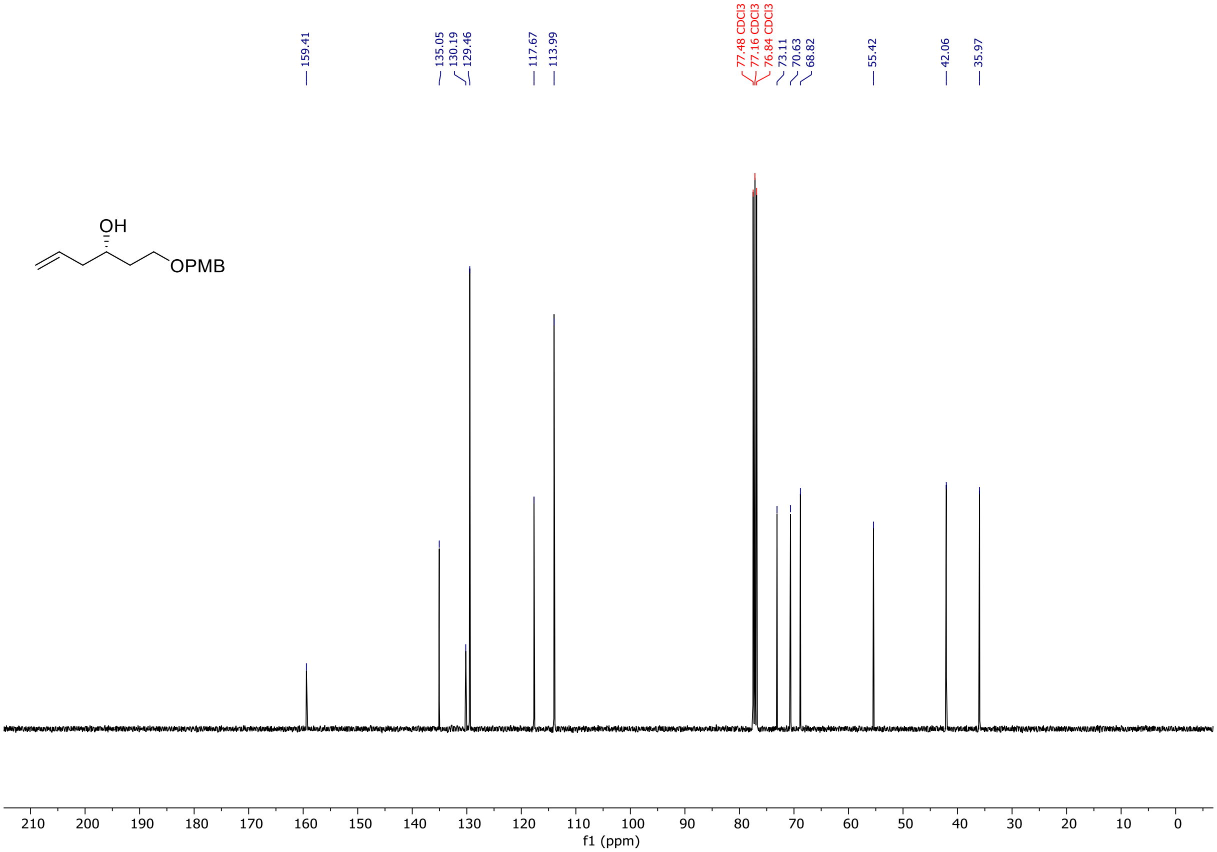

Compound 10: ^{1}H NMR ($CDCl_3$, 400 MHz)

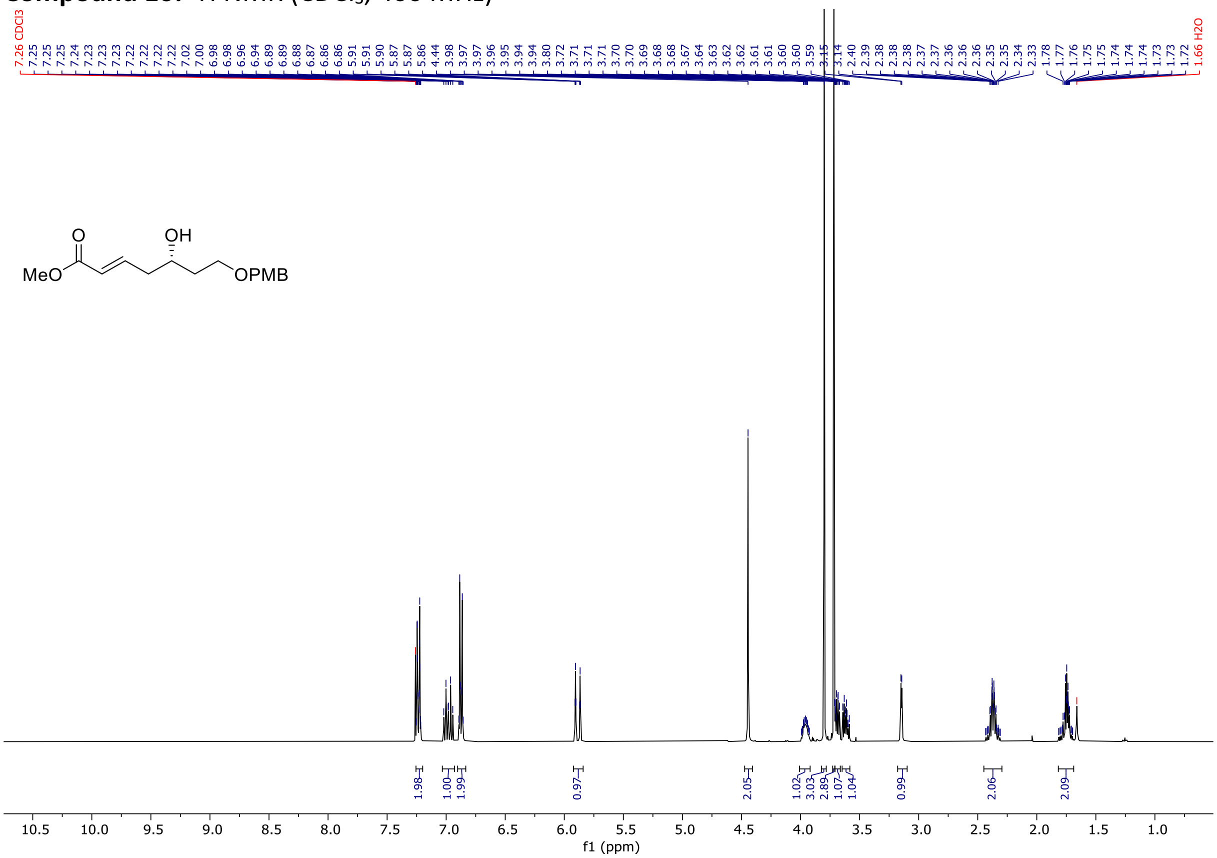

^{13}C NMR ($CDCl_3$, 101 MHz)

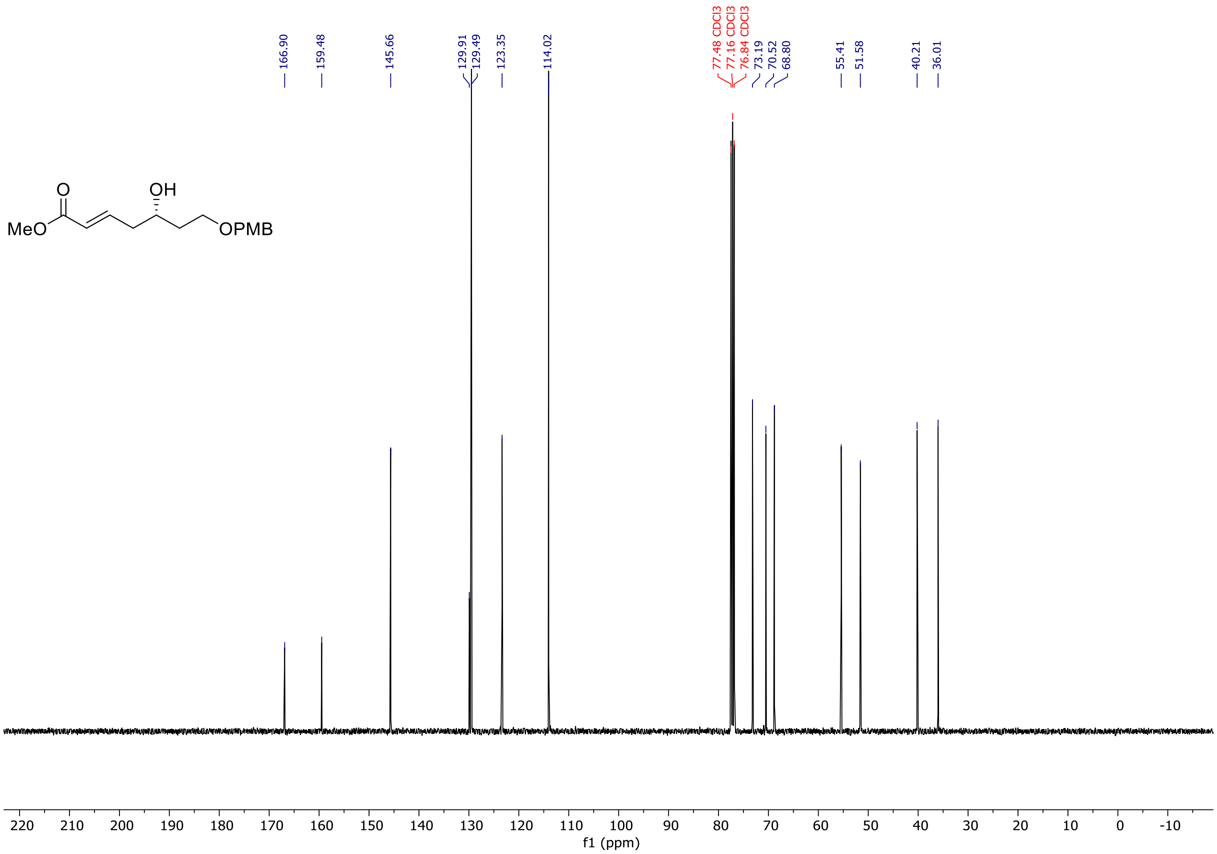

Compound 11a: ^{1}H NMR ($CDCl_3$, 400 MHz)

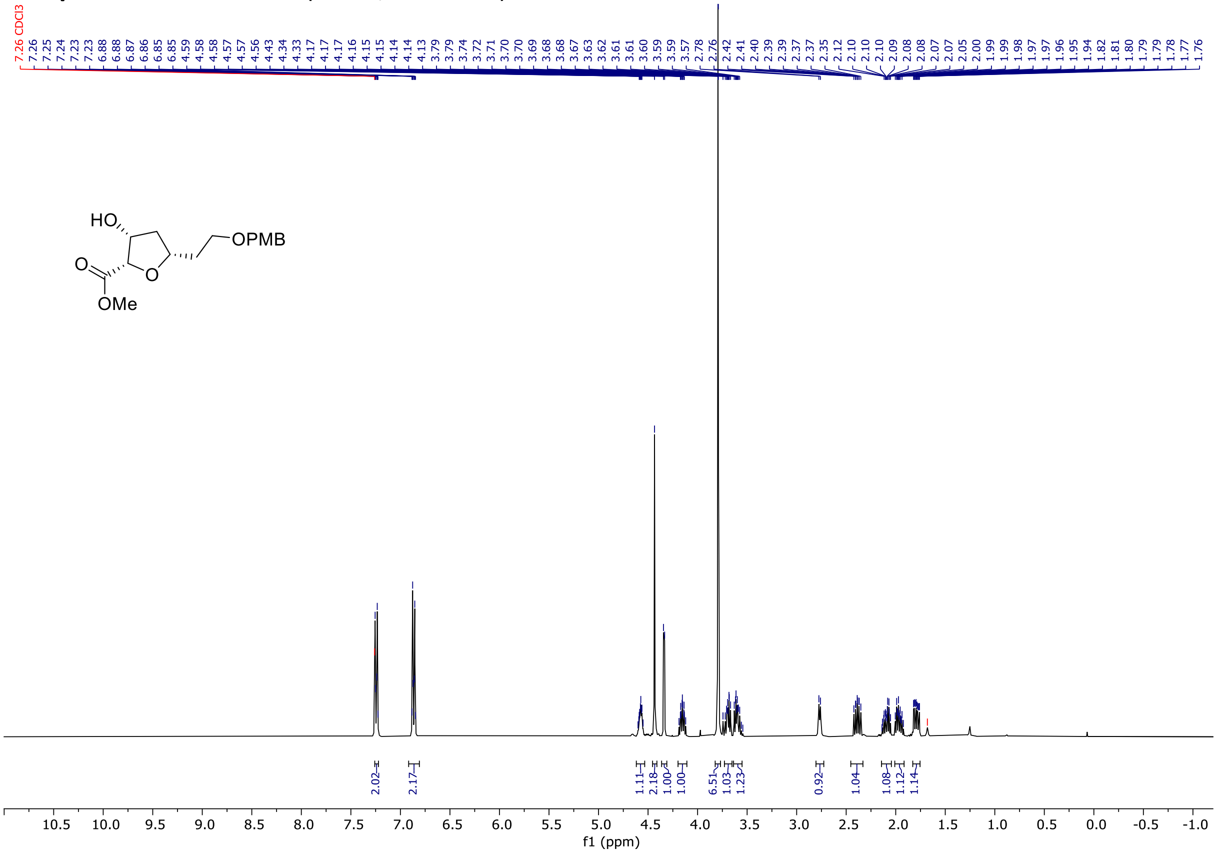

^{13}C NMR ($CDCl_3$, 101 MHz)

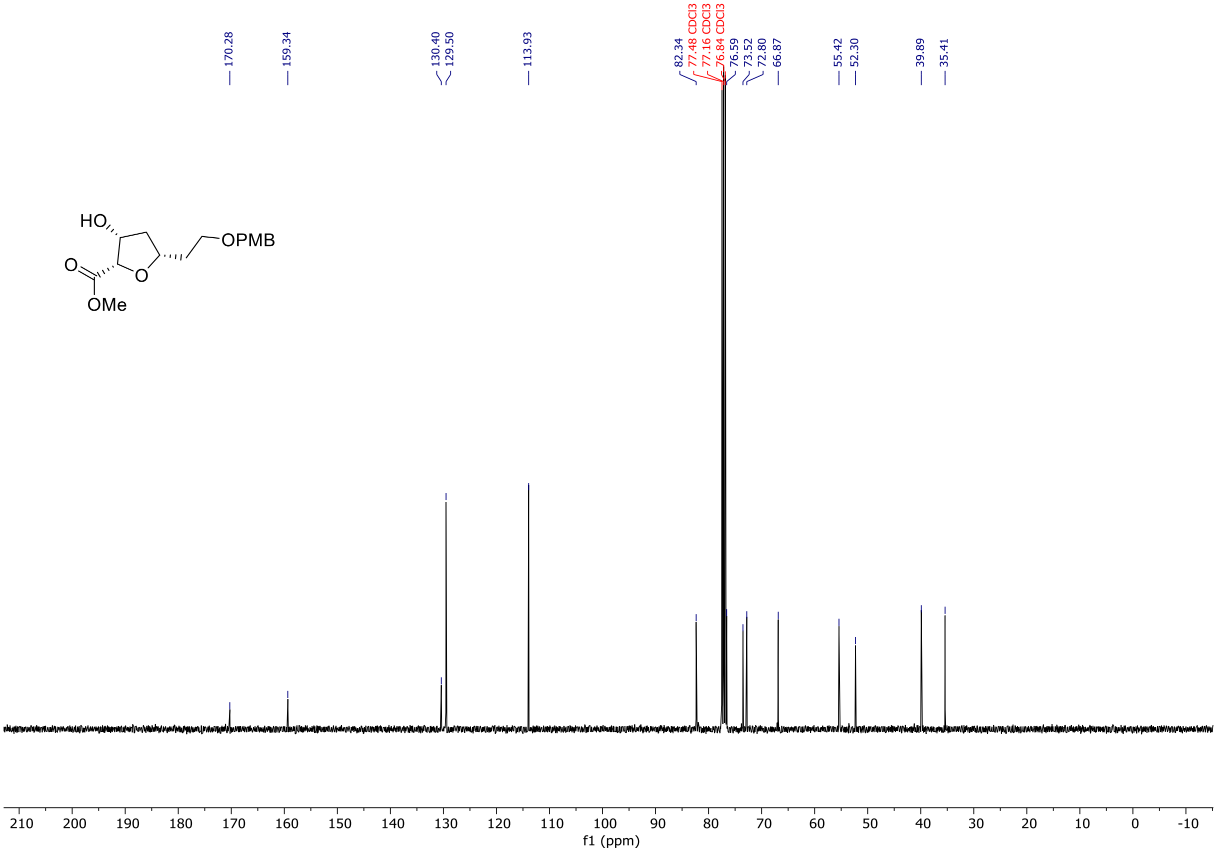

Compound 11: ^{1}H NMR ($CDCl_3$, 600 MHz)

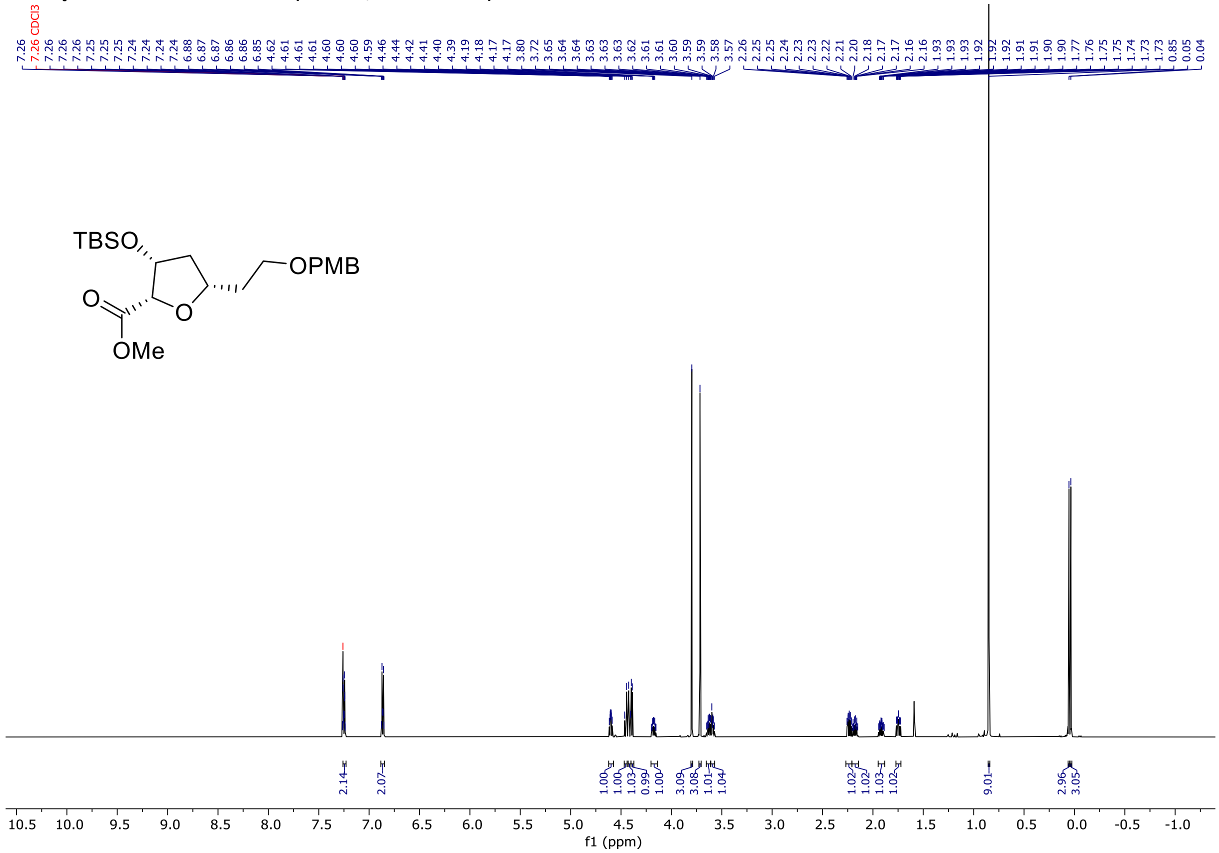

^{13}C NMR ($CDCl_3$, 151 MHz)

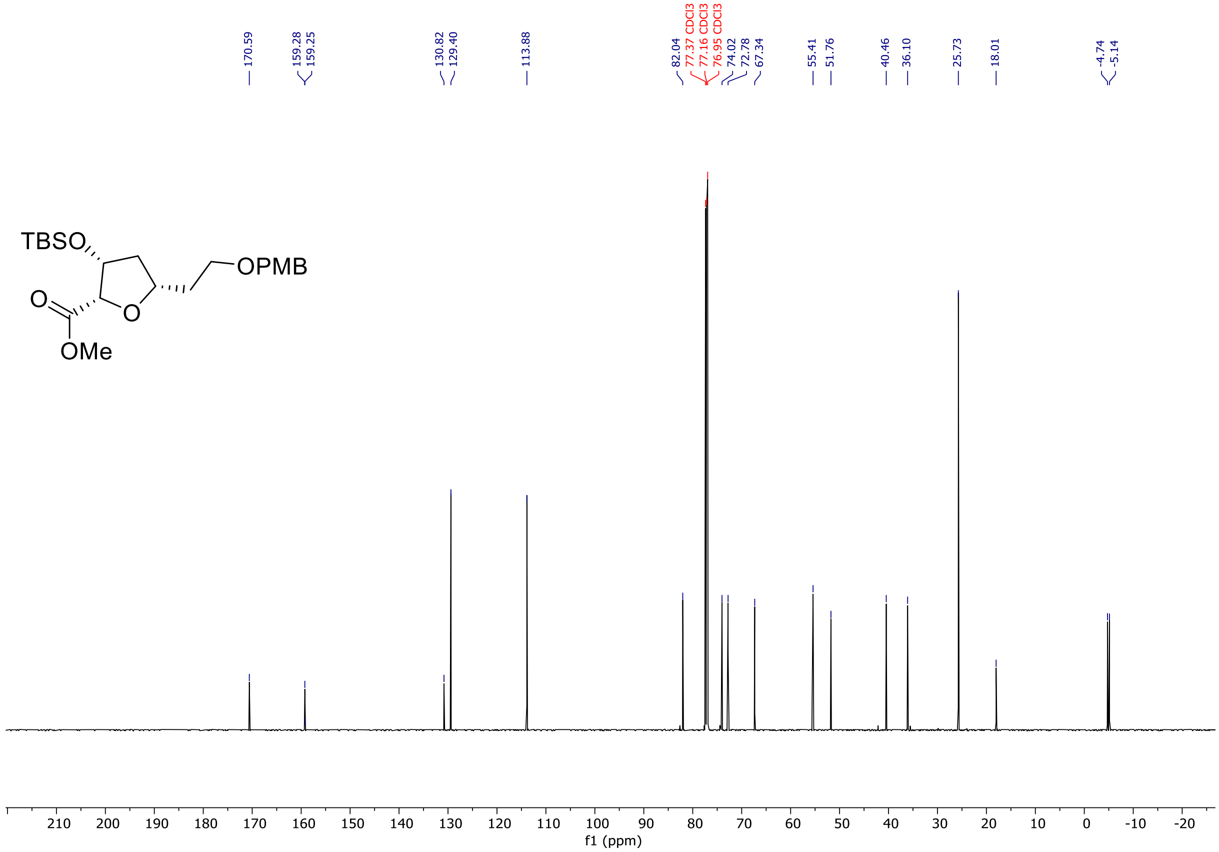

Compound 13: ^{1}H NMR ($CDCl_3$, 400 MHz)

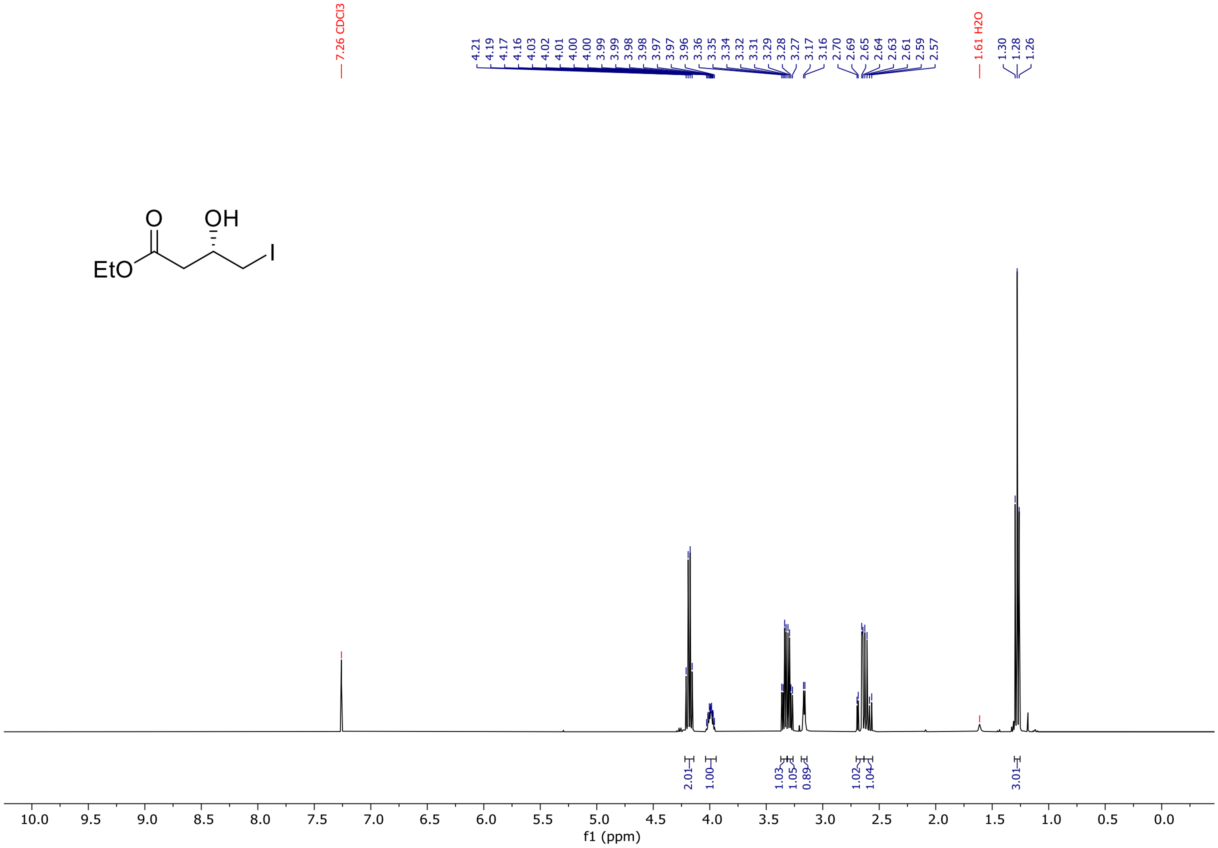

^{13}C NMR ($CDCl_3$, 101 MHz)

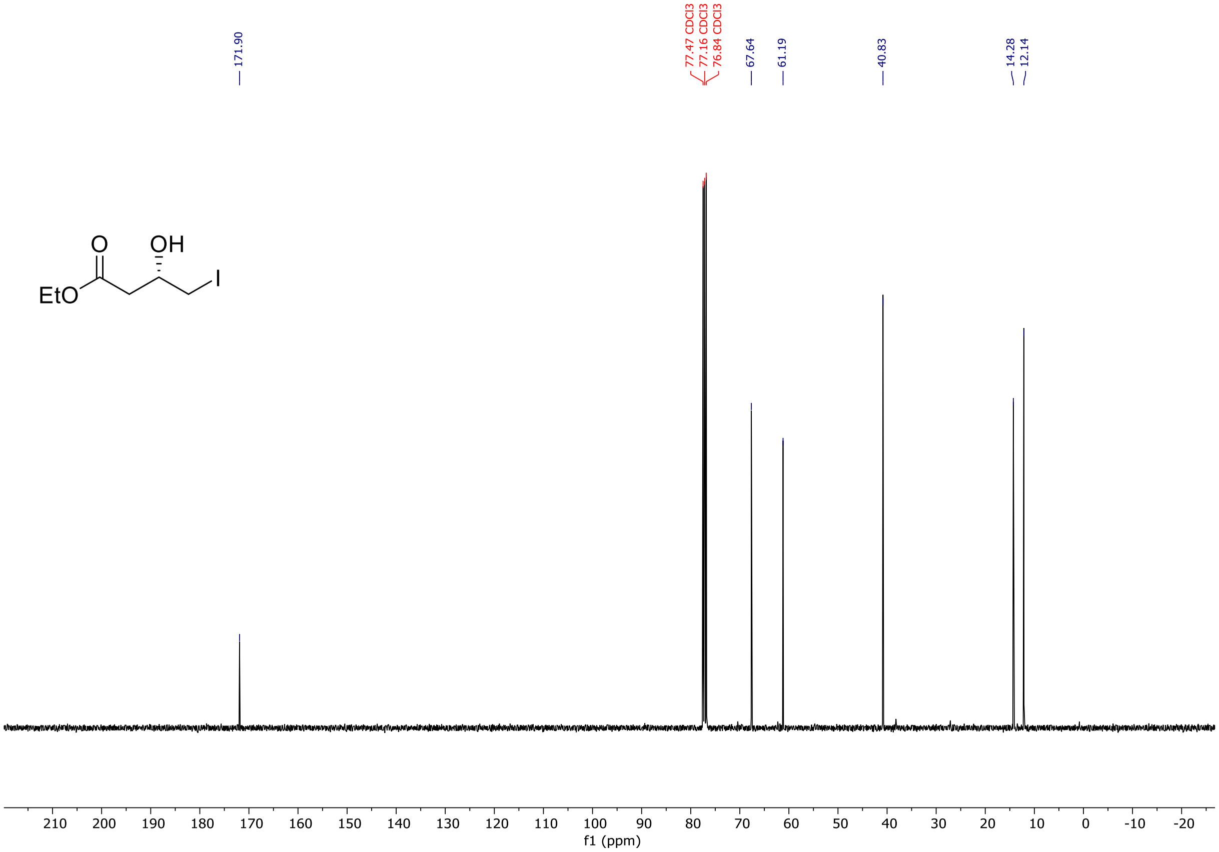

Compound 14: ^{1}H NMR ($CDCl_3$, 400 MHz)

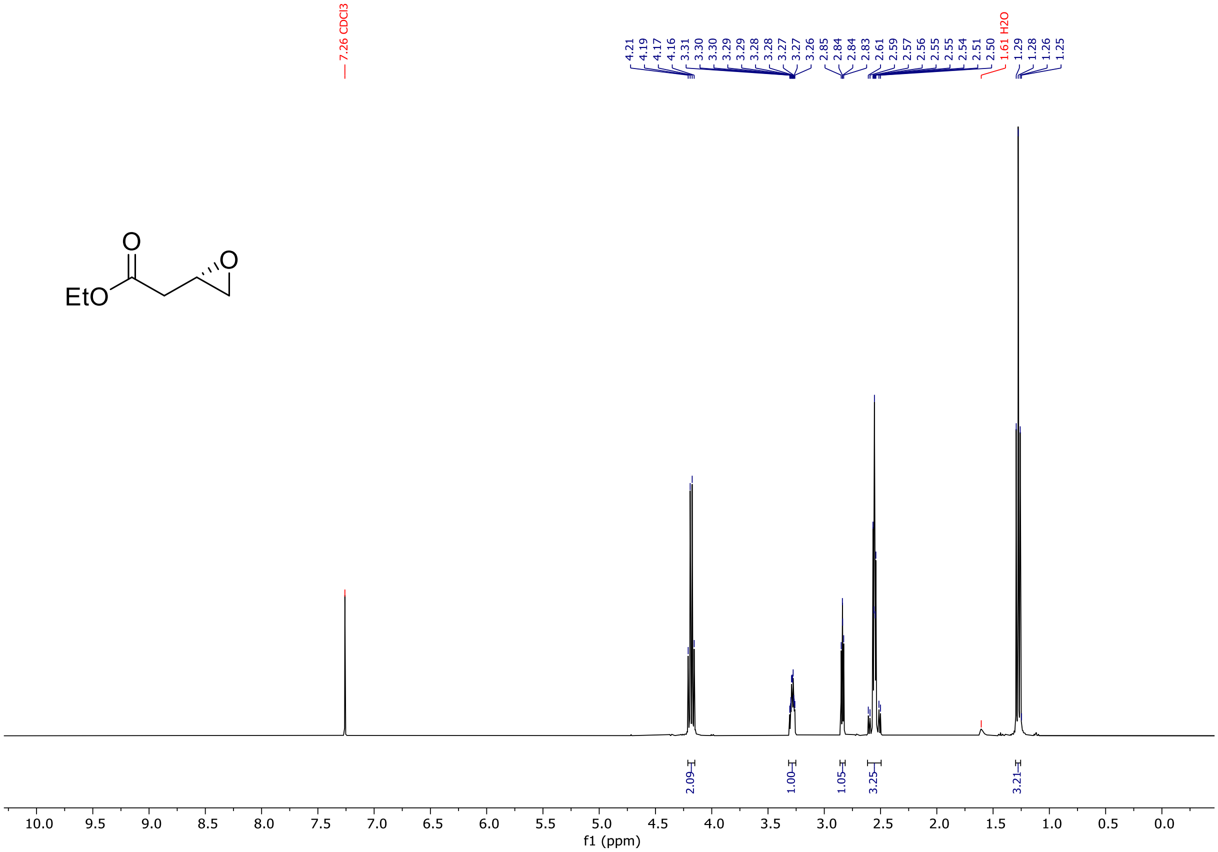

^{13}C NMR ($CDCl_3$, 101 MHz)

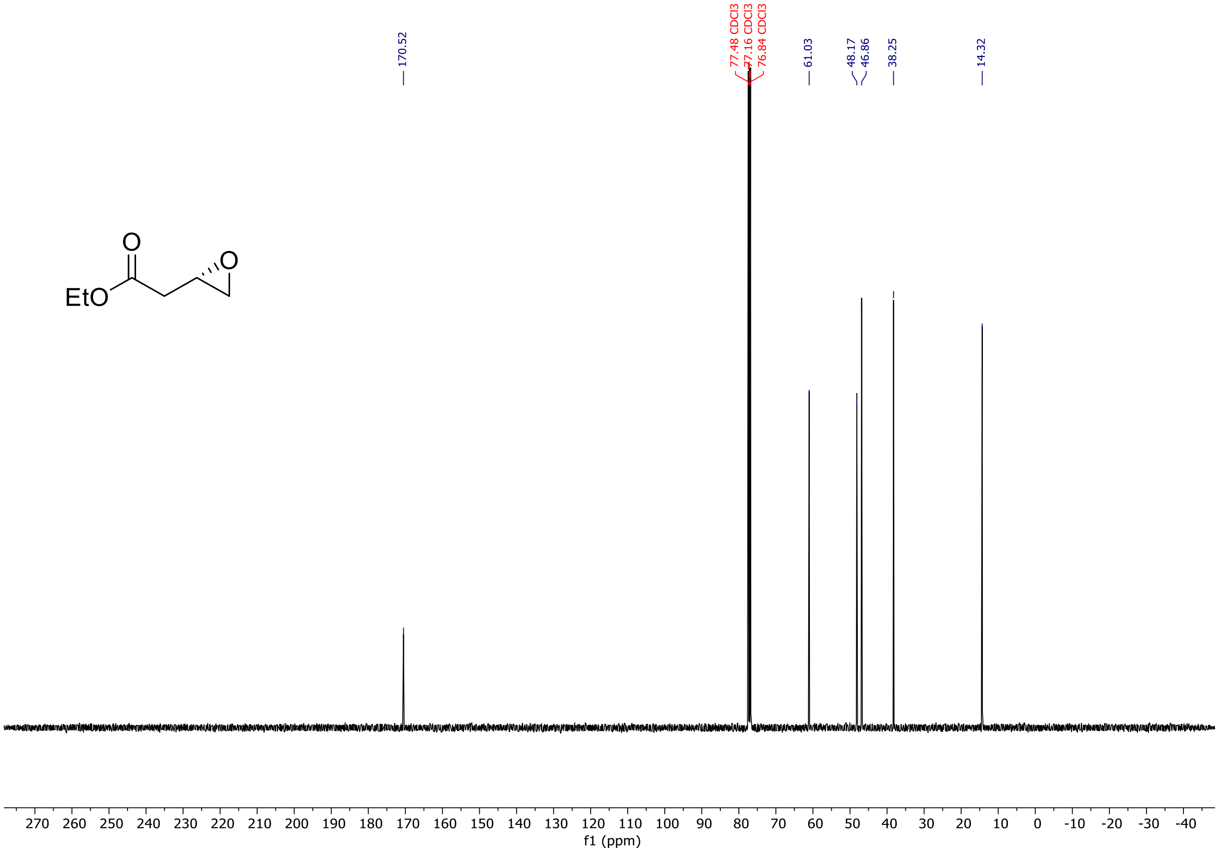

Compound 15: ^{1}H NMR ($CDCl_3$, 400 MHz)

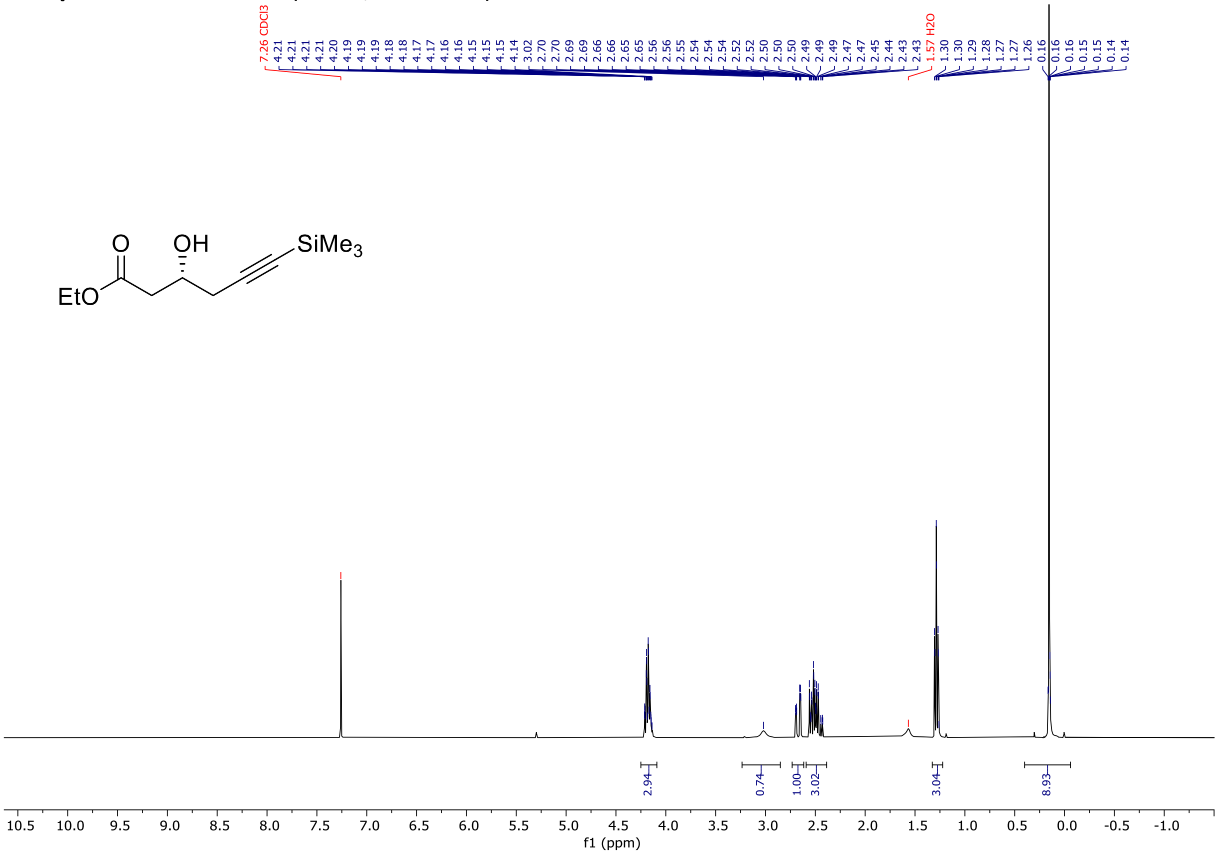

^{13}C NMR ($CDCl_3$, 101 MHz)

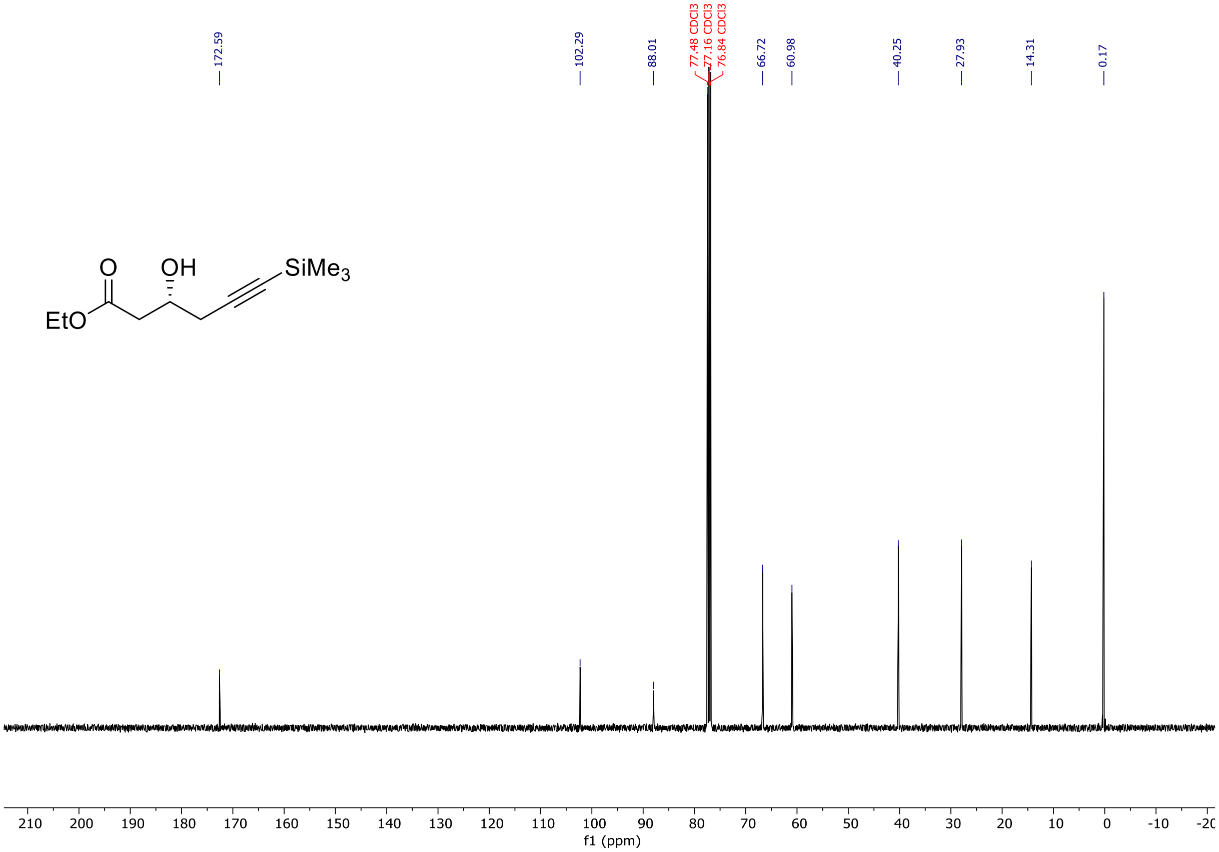

Compound S5: ^1H NMR ($CDCl_3$, 400 MHz)

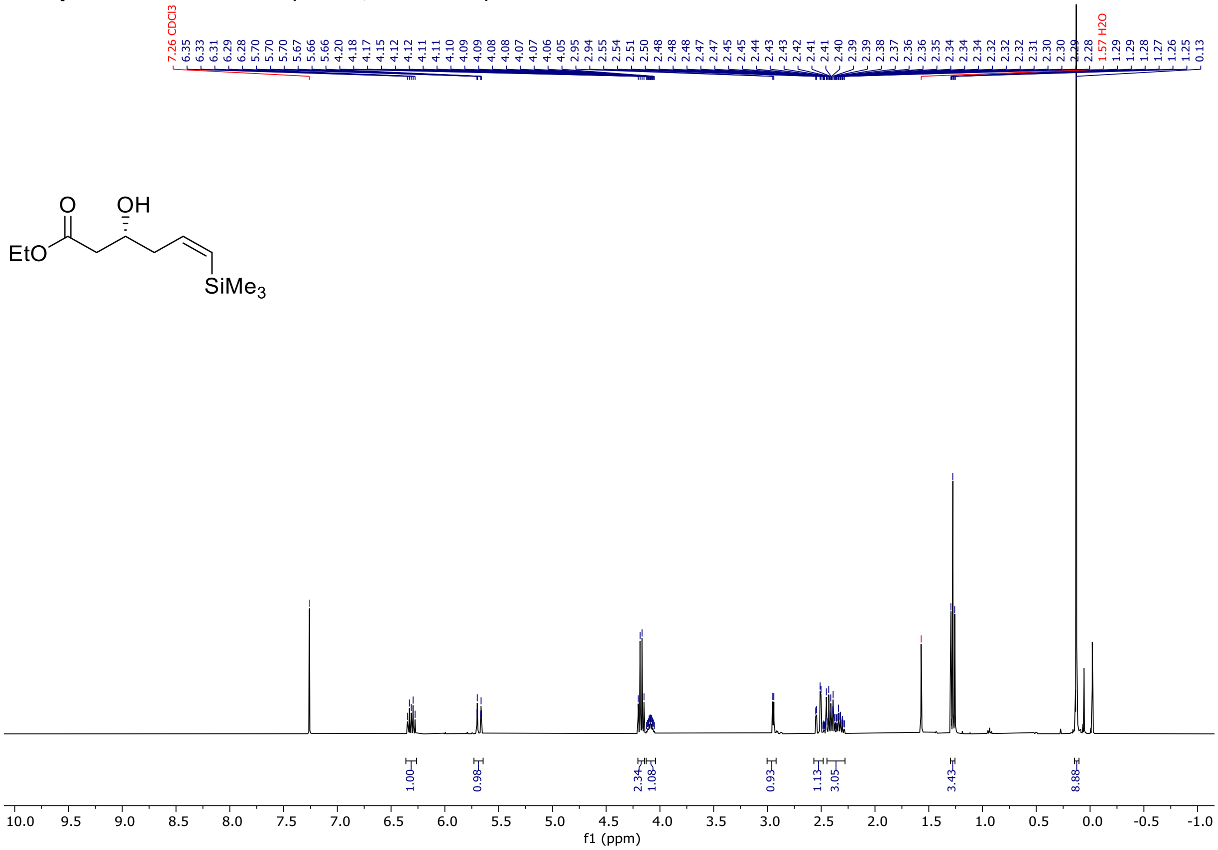

^{13}C NMR ($CDCl_3$, 101 MHz)

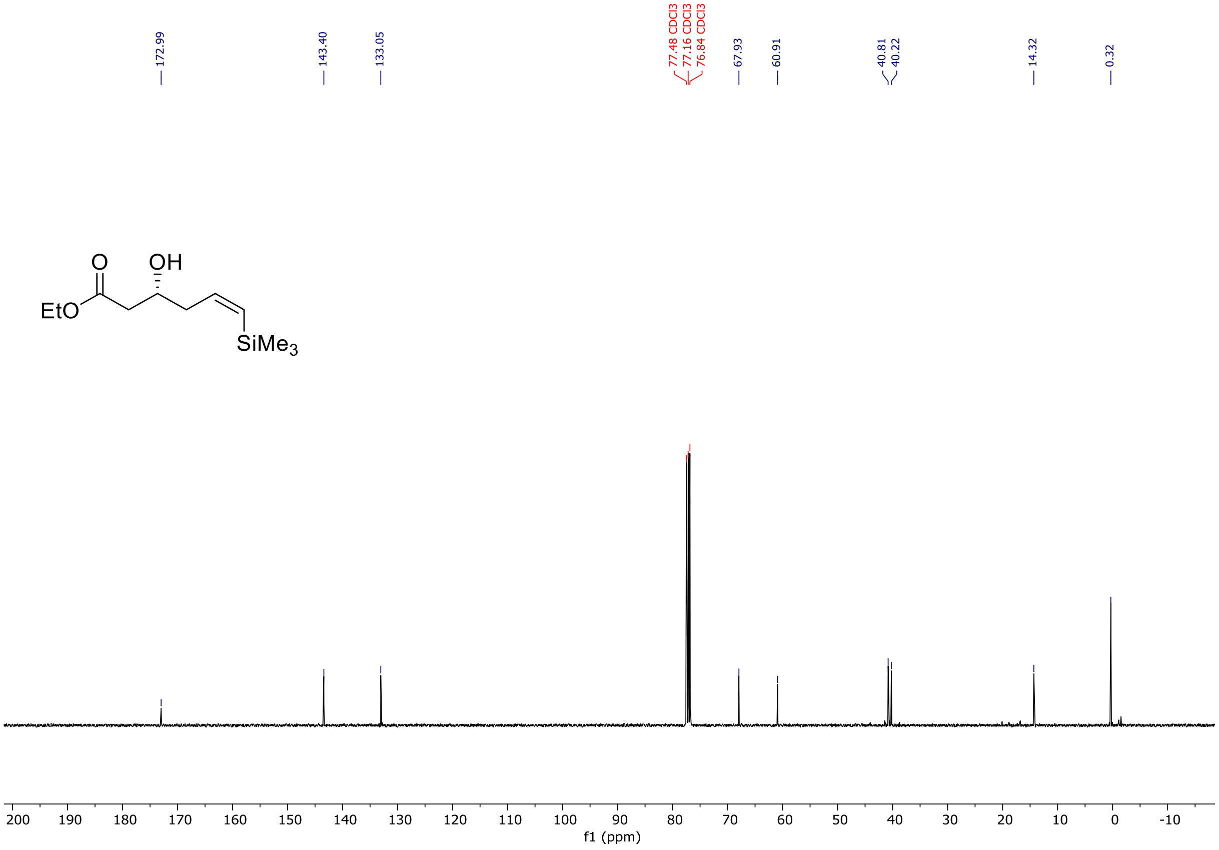

Compound 16: ^{1}H NMR (400 MHz, $CDCl_3$)

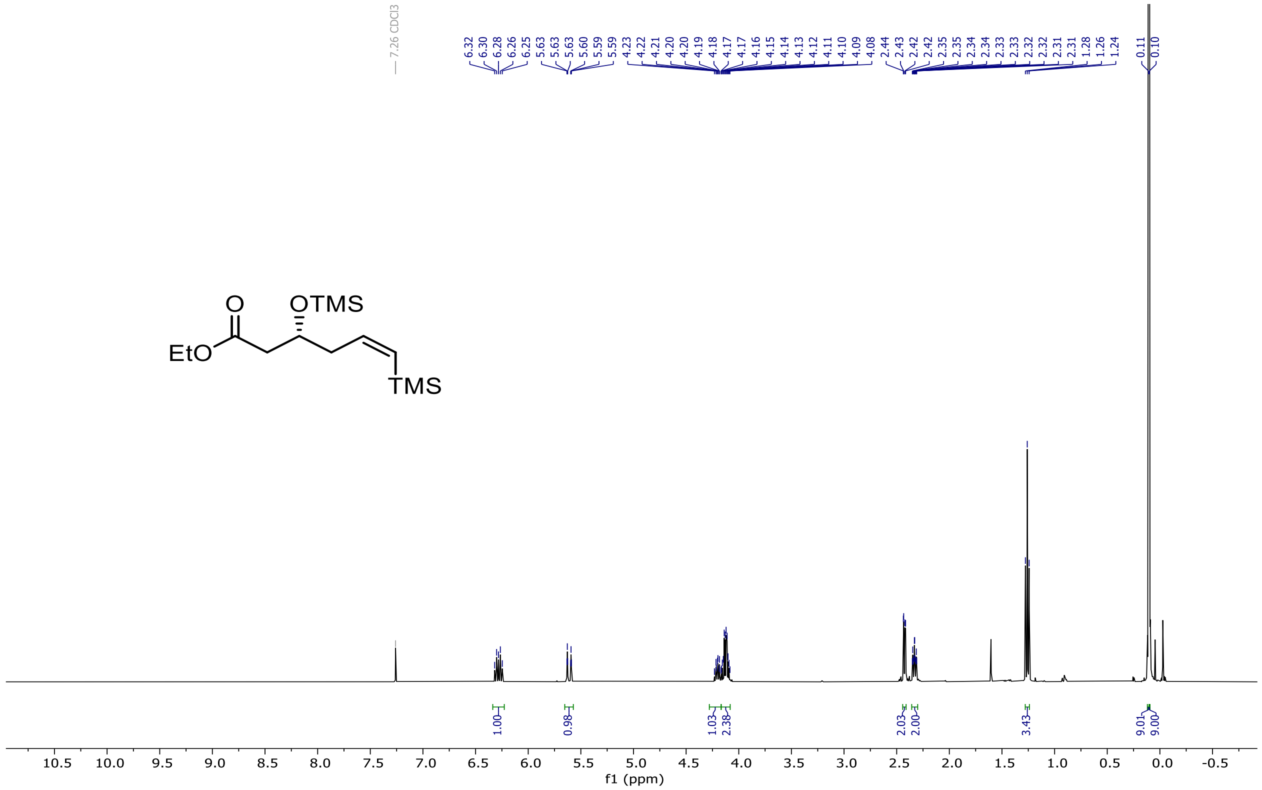

^{13}C NMR (101 MHz, $CDCl_3$)

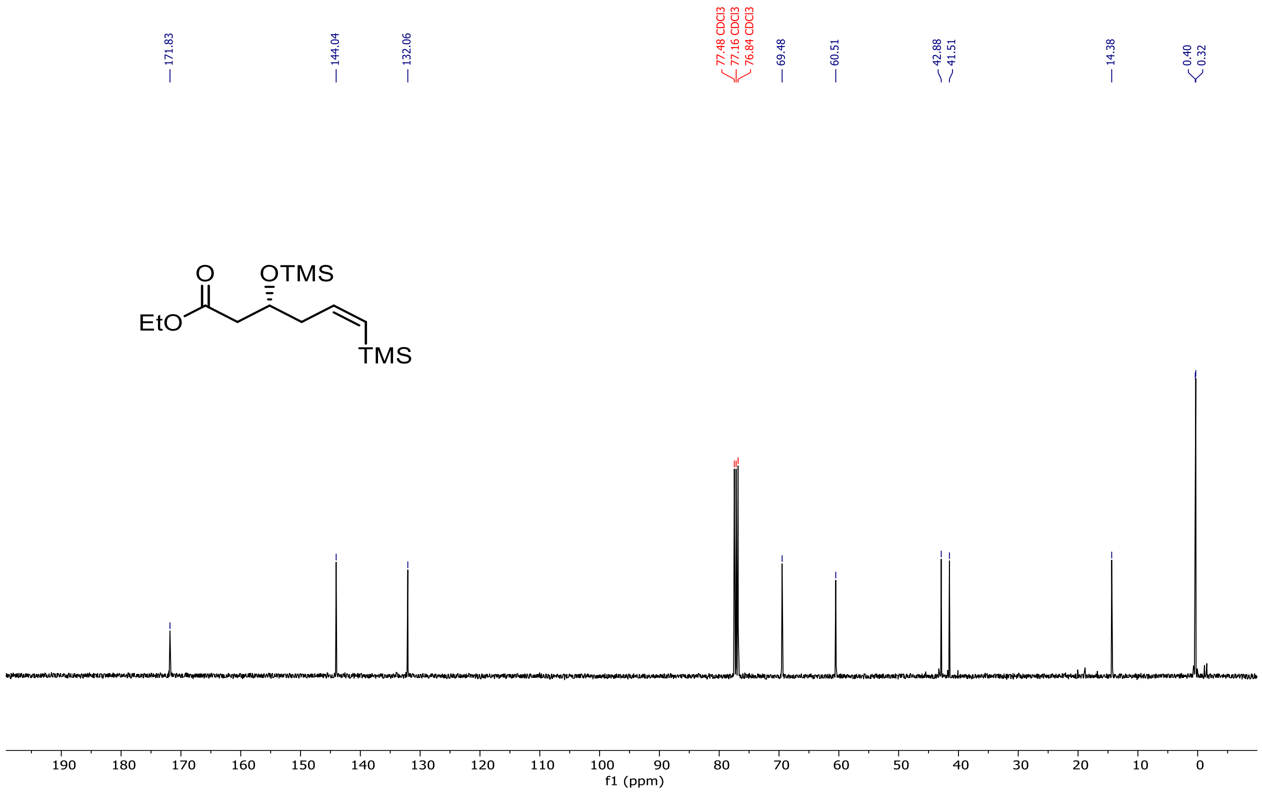

Compound 17: ^{1}H NMR (400 MHz, $CDCl_3$)

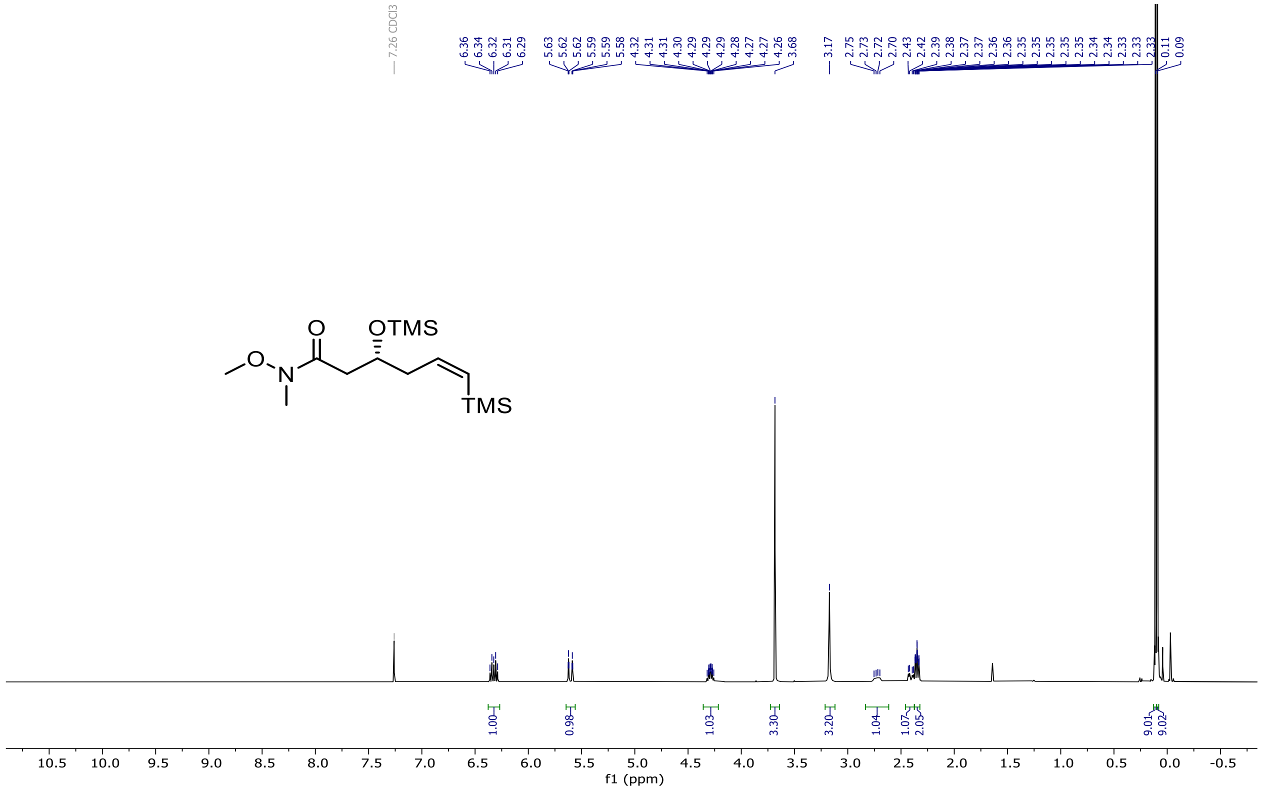

^{13}C NMR (101 MHz, $CDCl_3$)

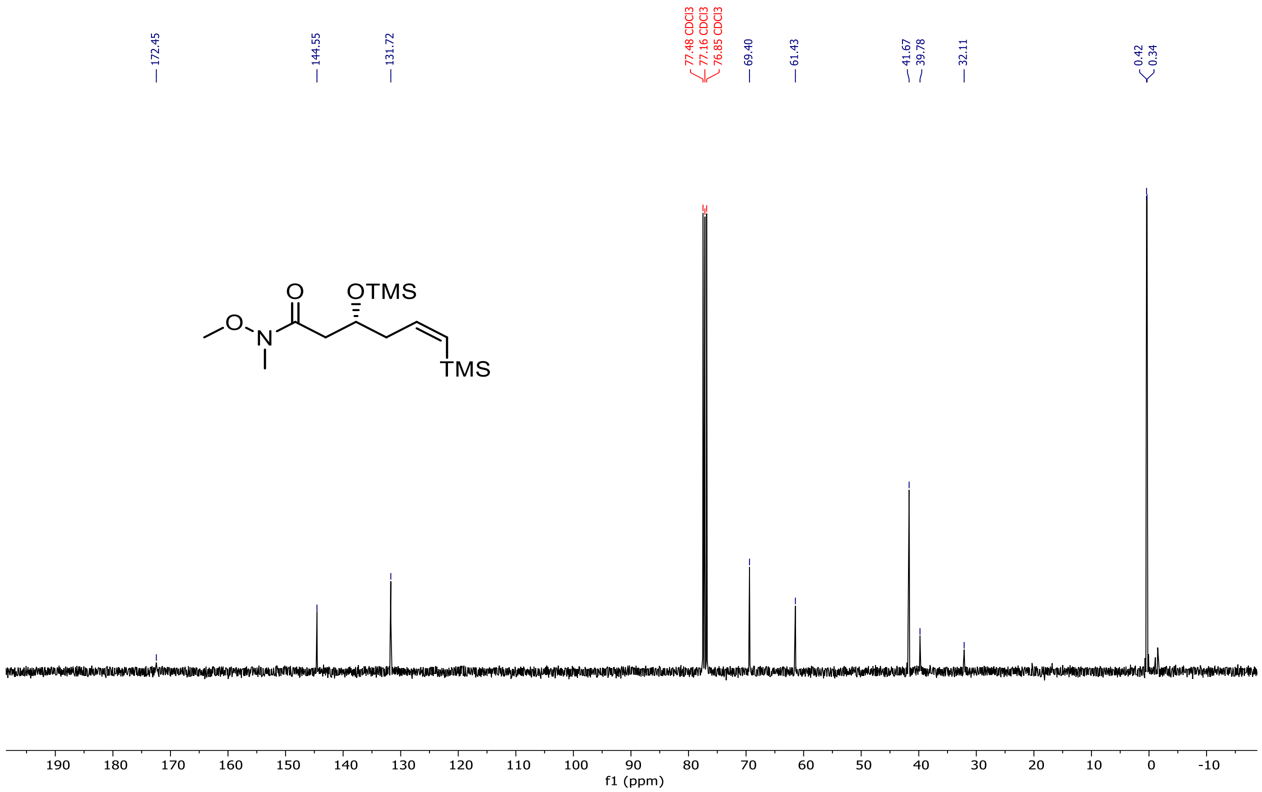

Compound 18: ^{1}H NMR (400 MHz, $CDCl_3$)

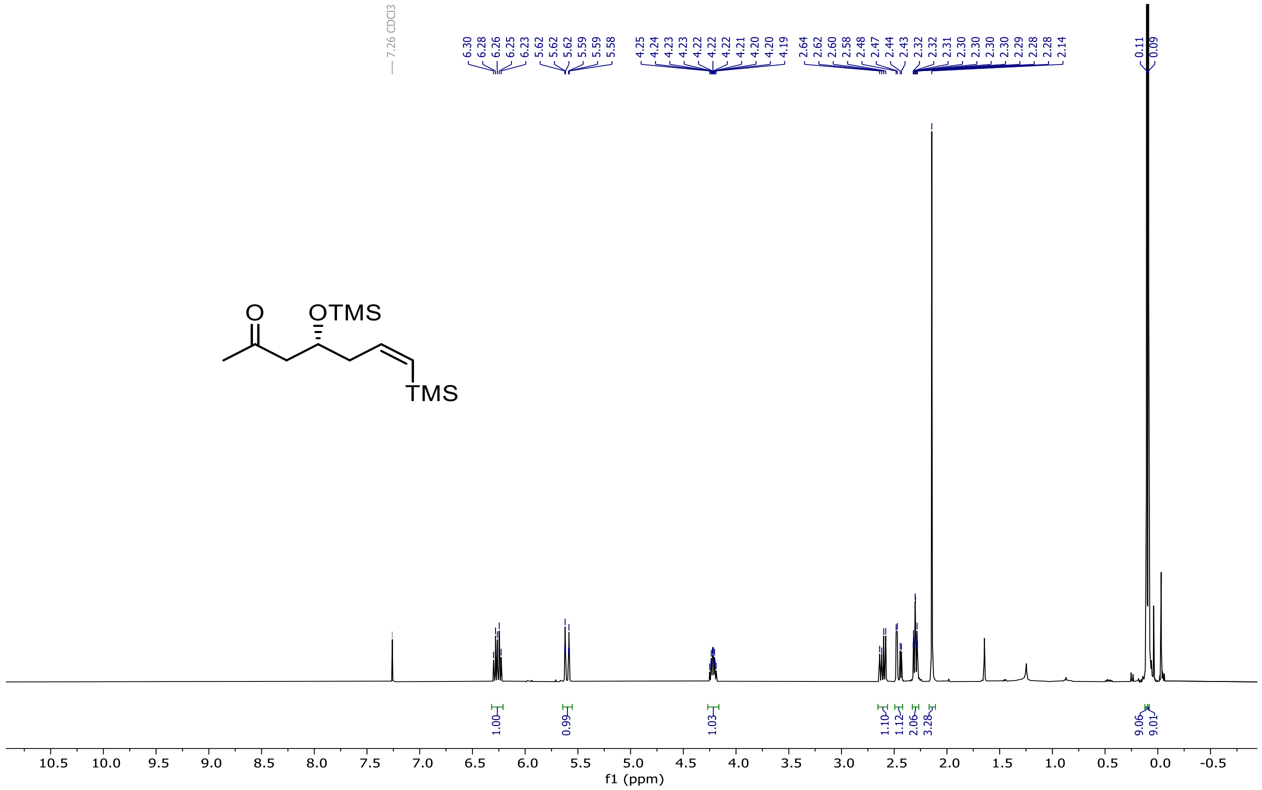

^{13}C NMR (101 MHz, $CDCl_3$)

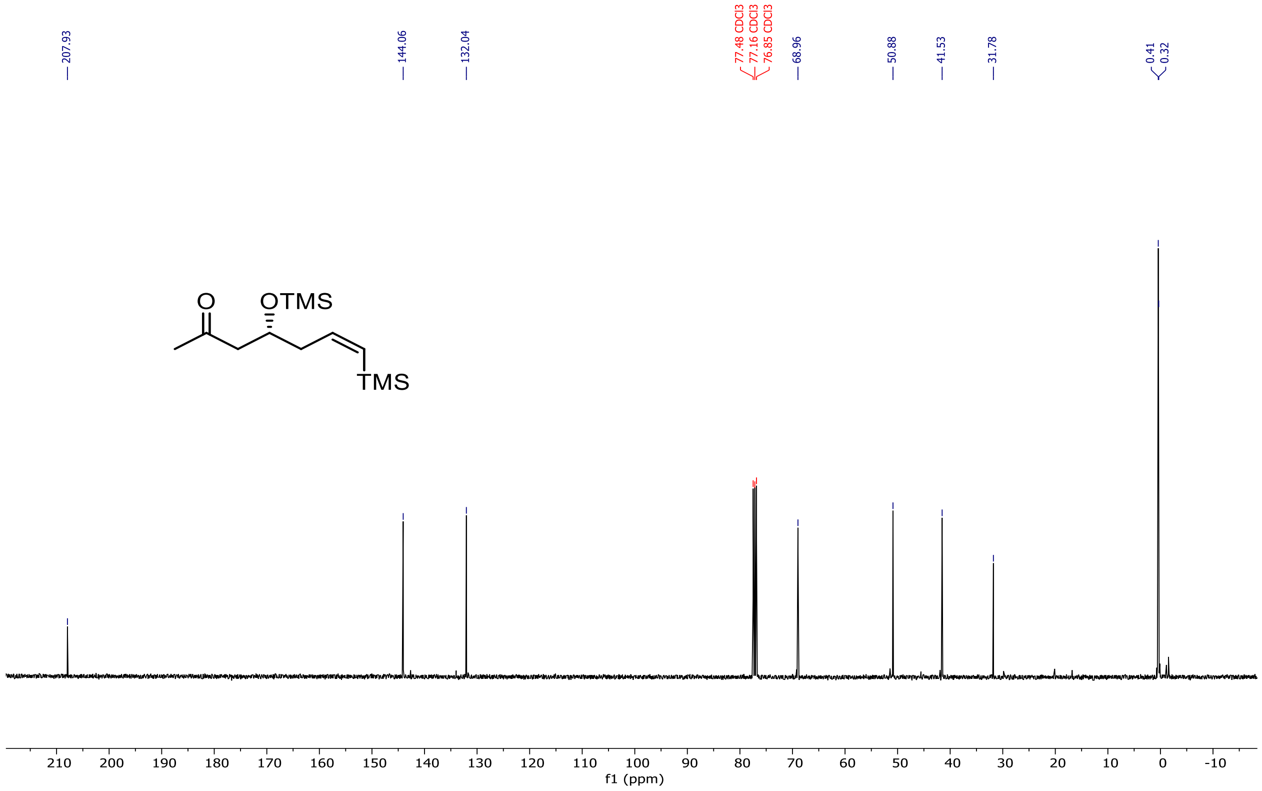

Compound S6: ^{1}H NMR ($CDCl_3$, 400 MHz)

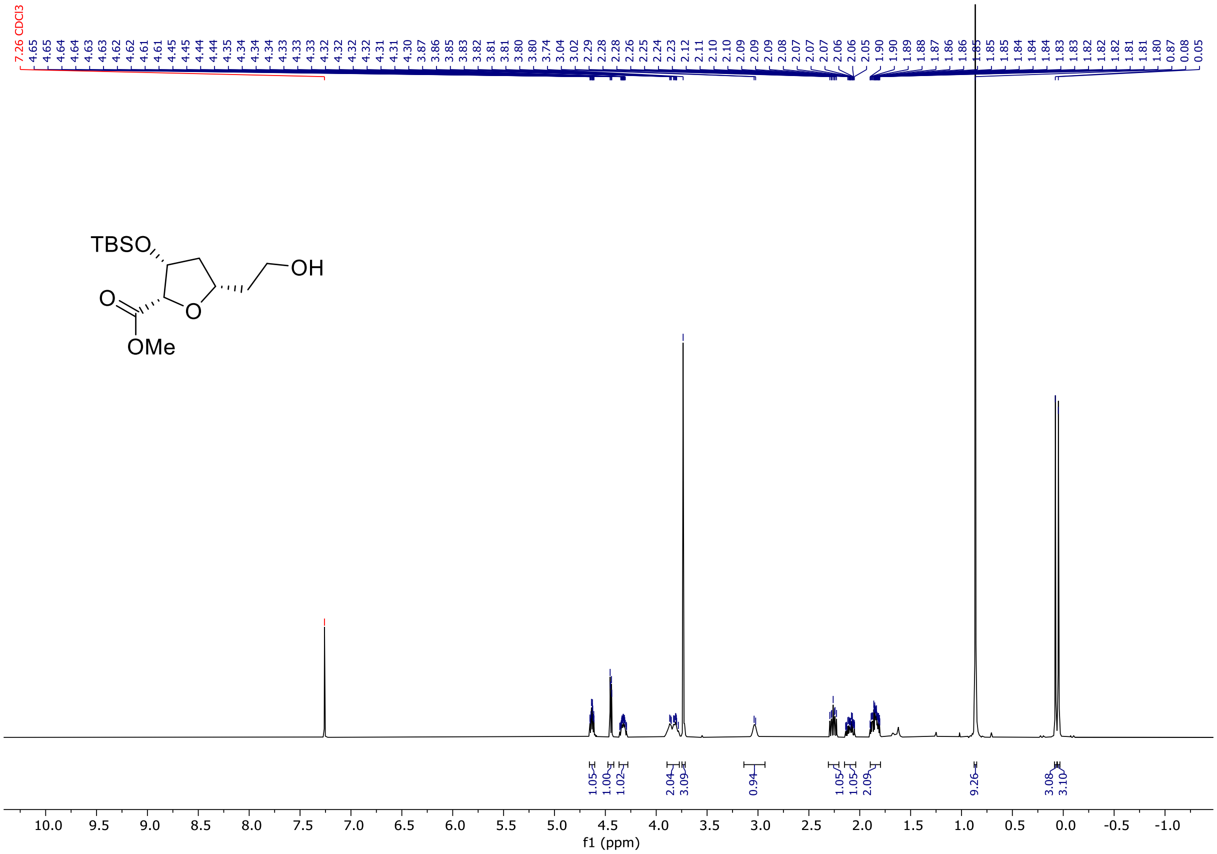

^{13}C NMR ($CDCl_3$, 101 MHz)

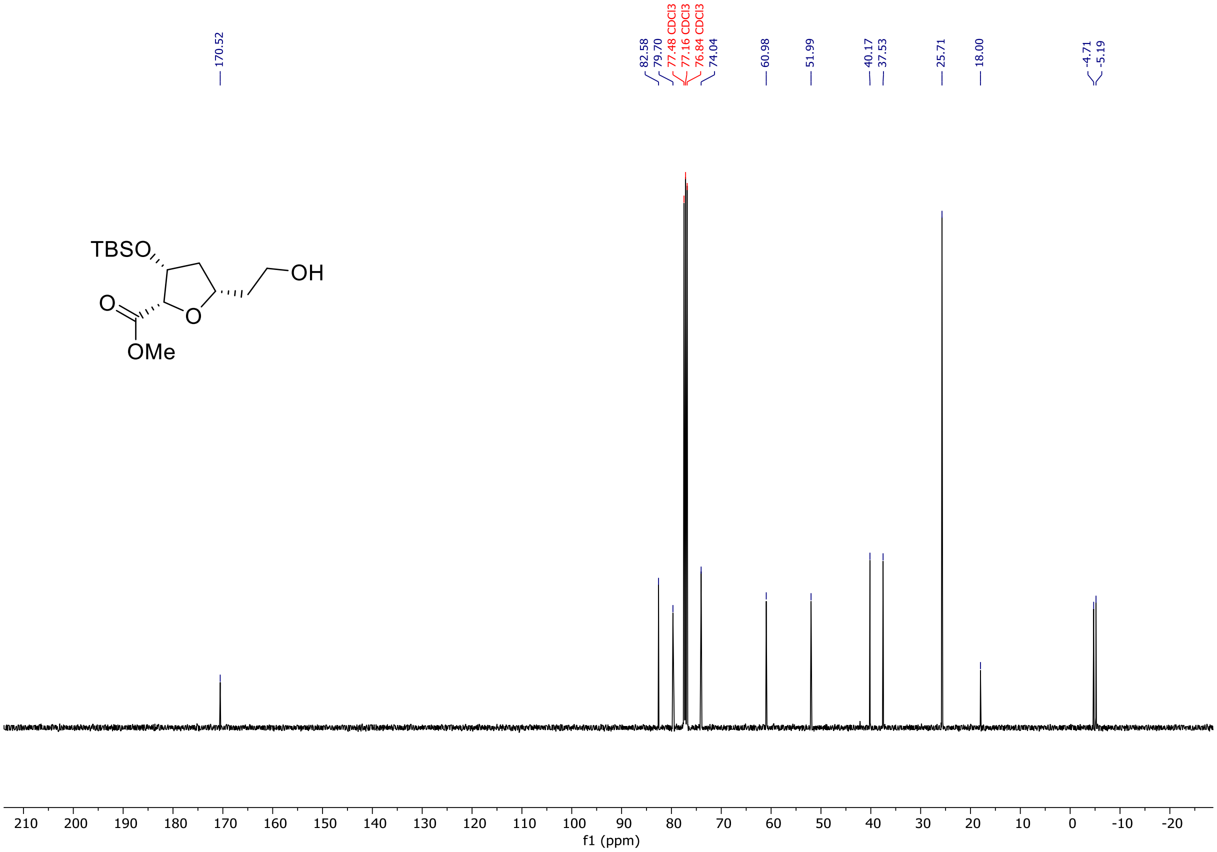

Compound 19: ^{1}H NMR (400 MHz, $CDCl_3$)

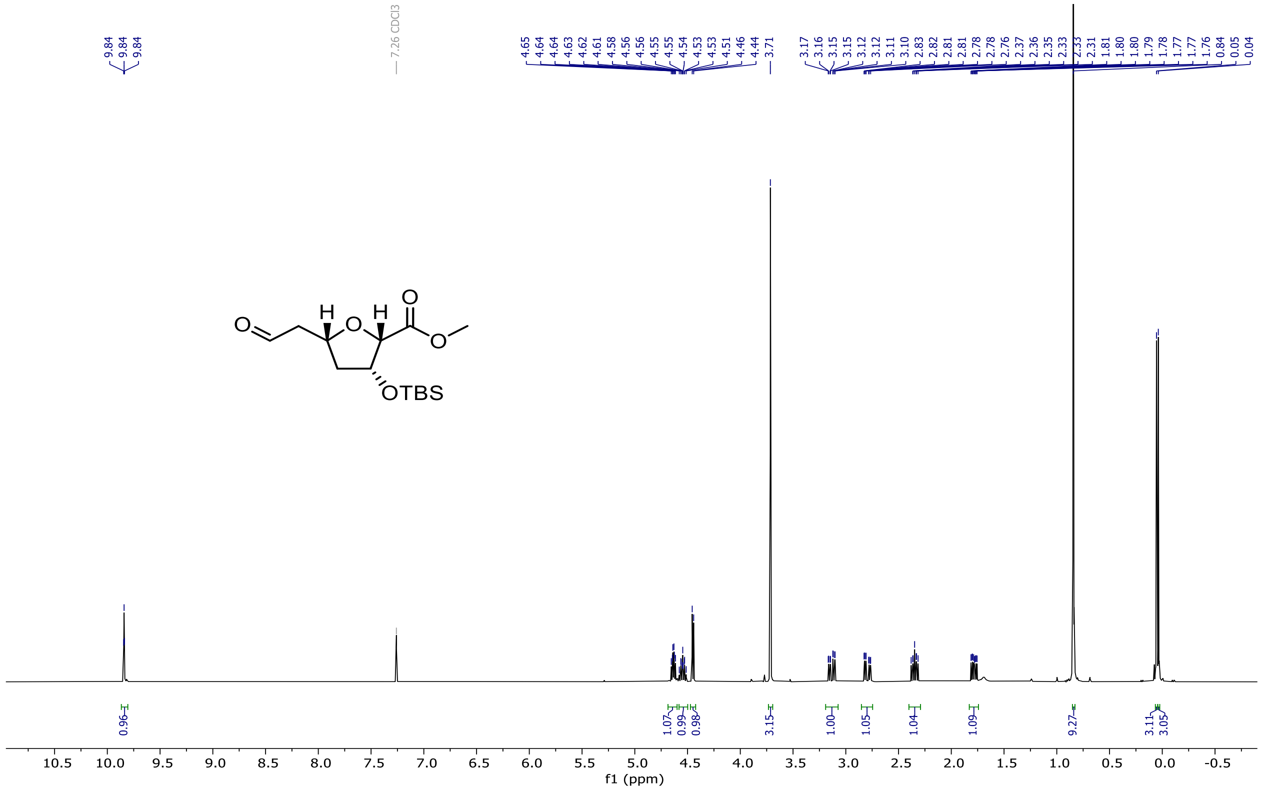

^{13}C NMR (101 MHz, $CDCl_3$)

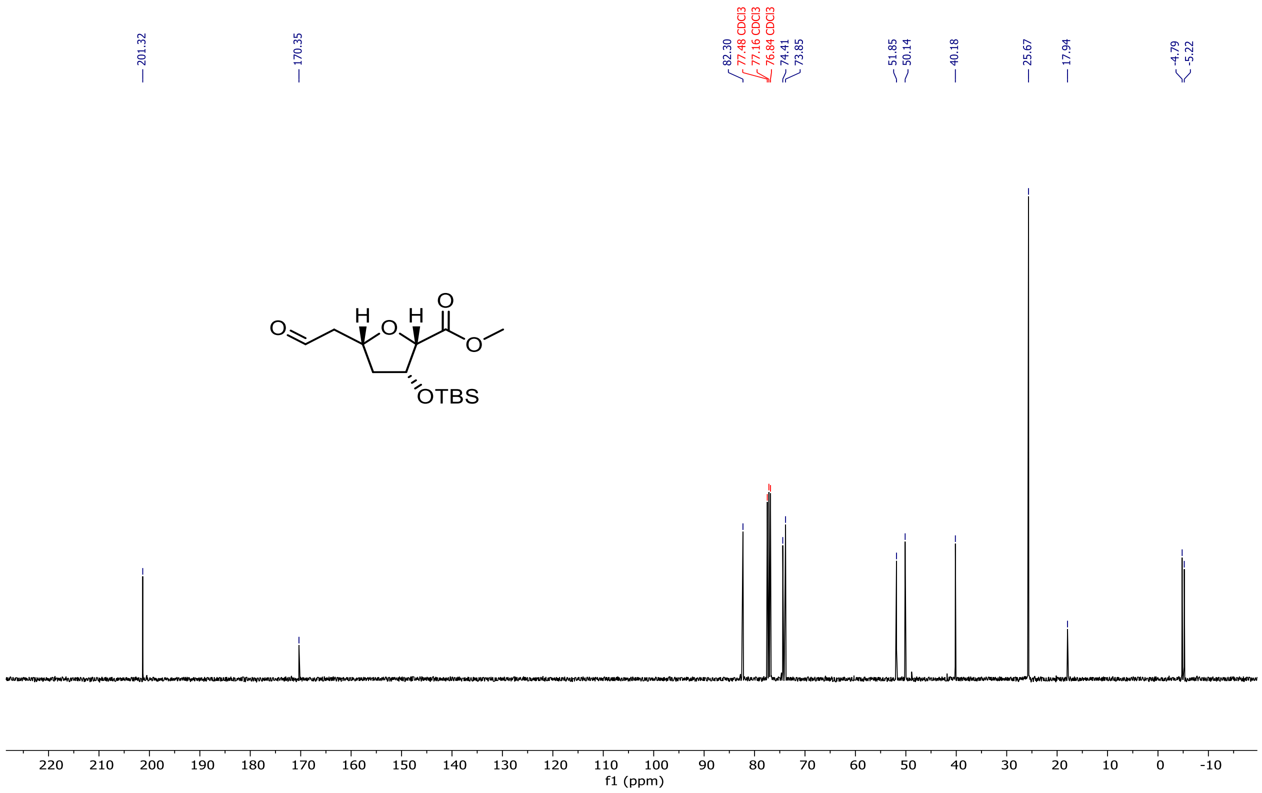

Compound 20: ^{1}H NMR (600 MHz, $CDCl_3$)

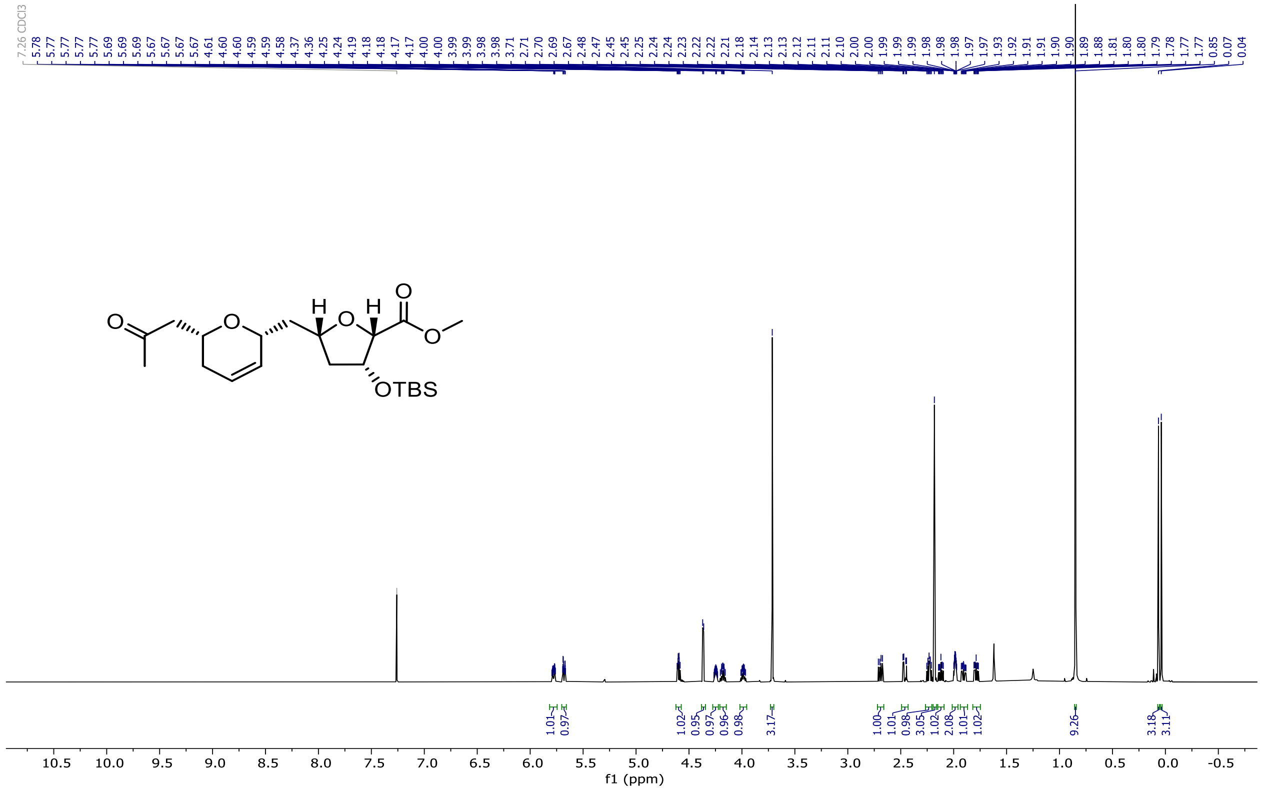

^{13}C NMR (151 MHz, $CDCl_3$)

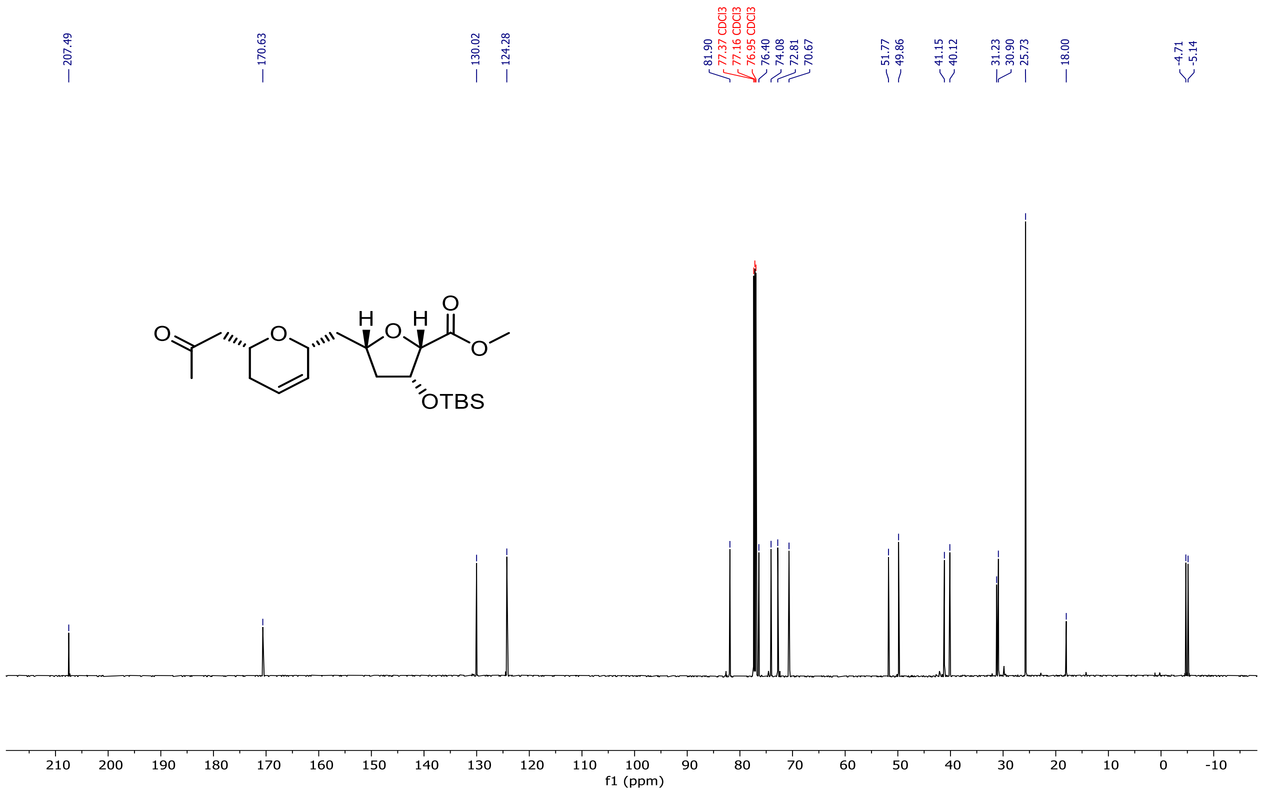

Compound 20: ^{1}H-^{1}H COSY (CDCl$_3$)

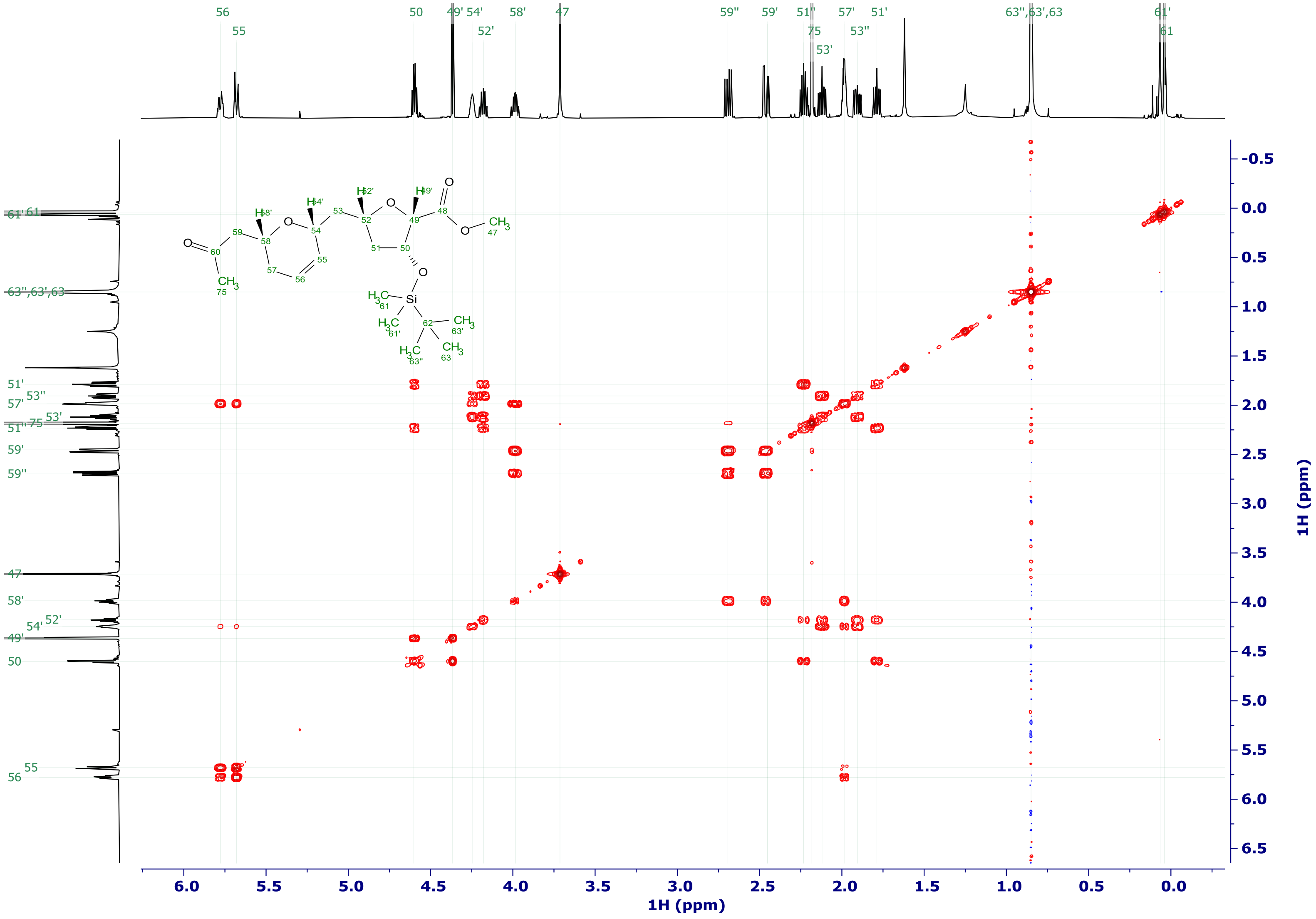

Compound 20: HSQC NMR ($CDCl_3$)

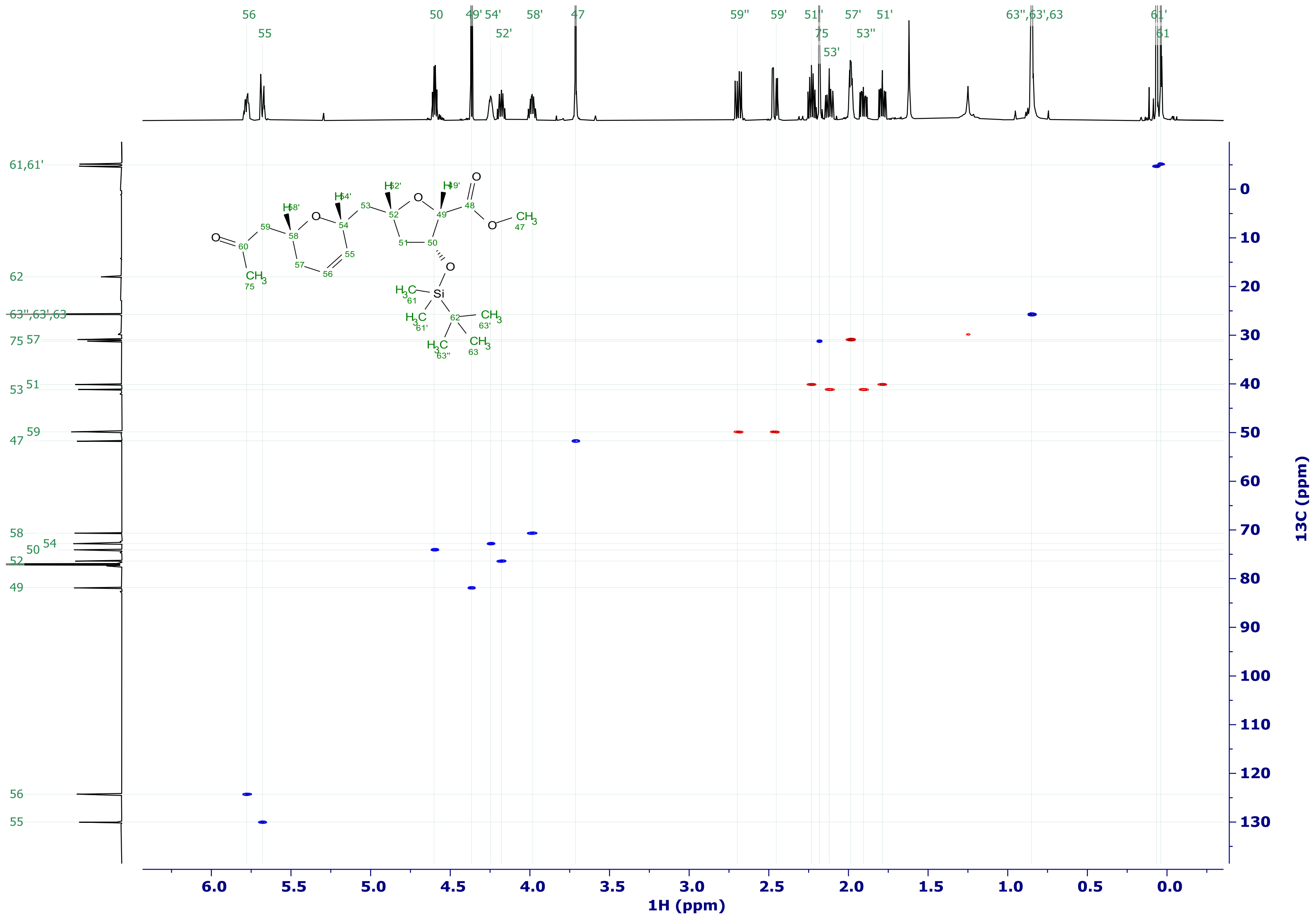

Compound 20: HMBC NMR ($CDCl_3$)

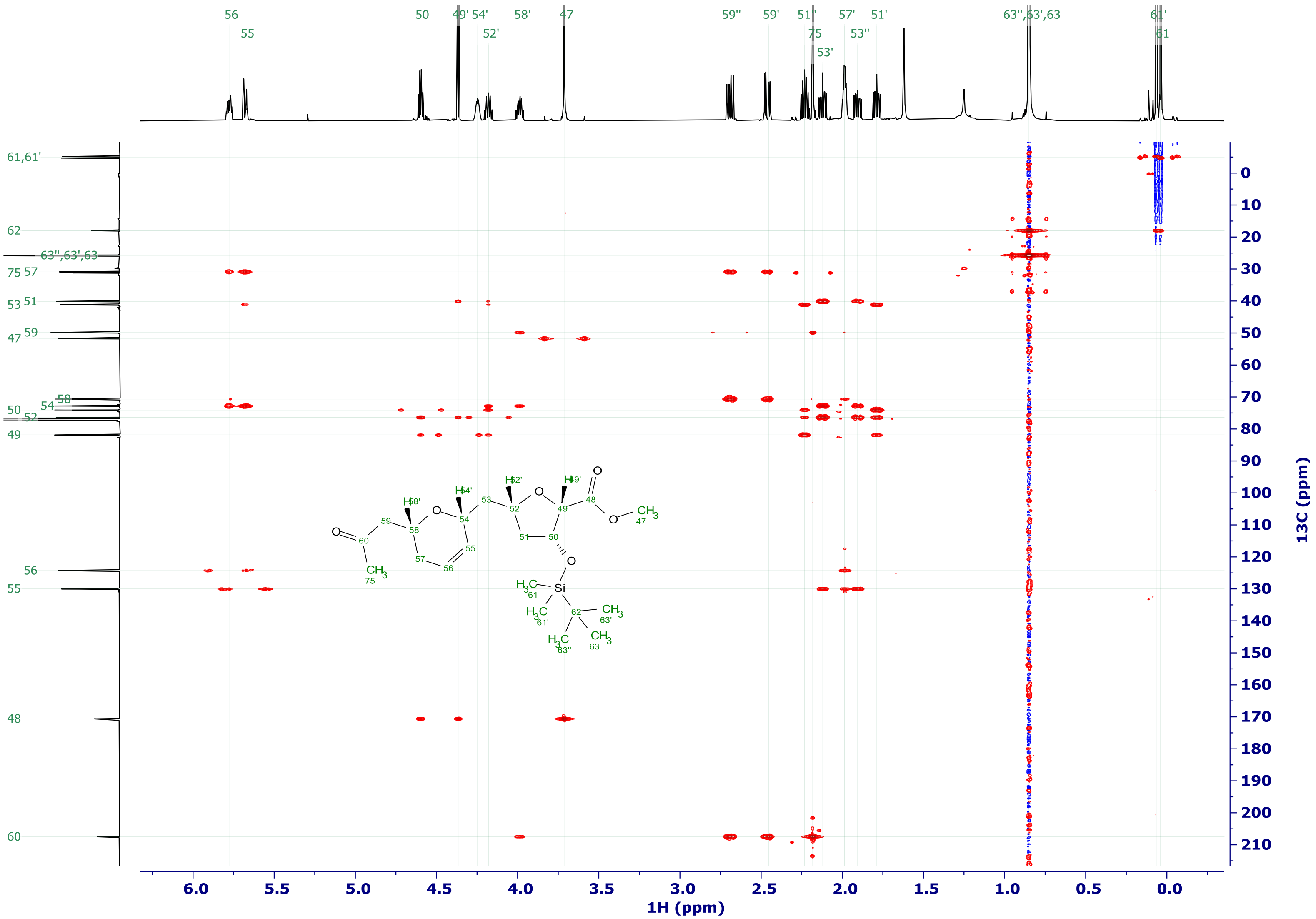

Compound 20: NOESY ($CDCl_3$)

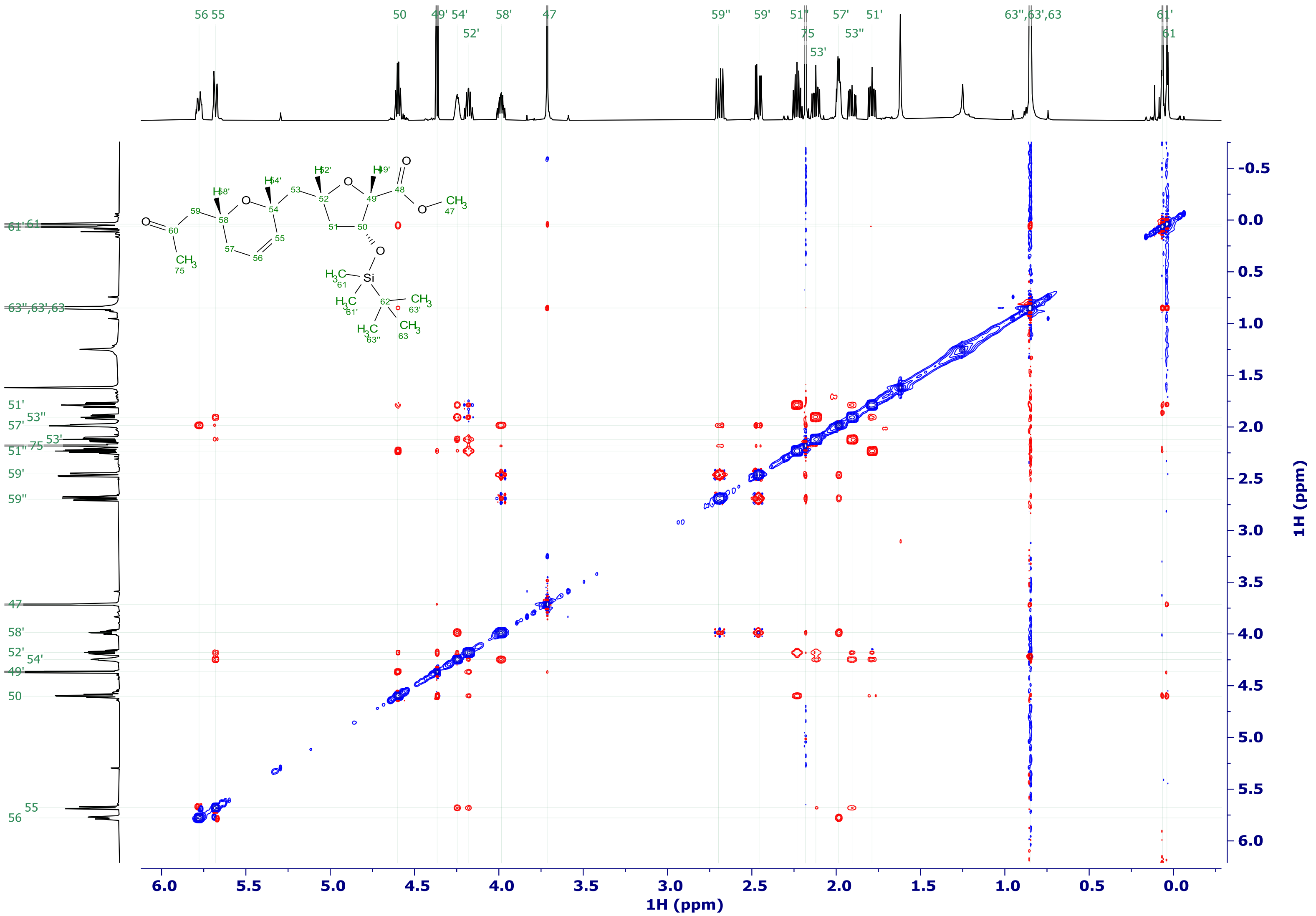

Compound 21: ^{1}H NMR (600 MHz, $CDCl_3$)

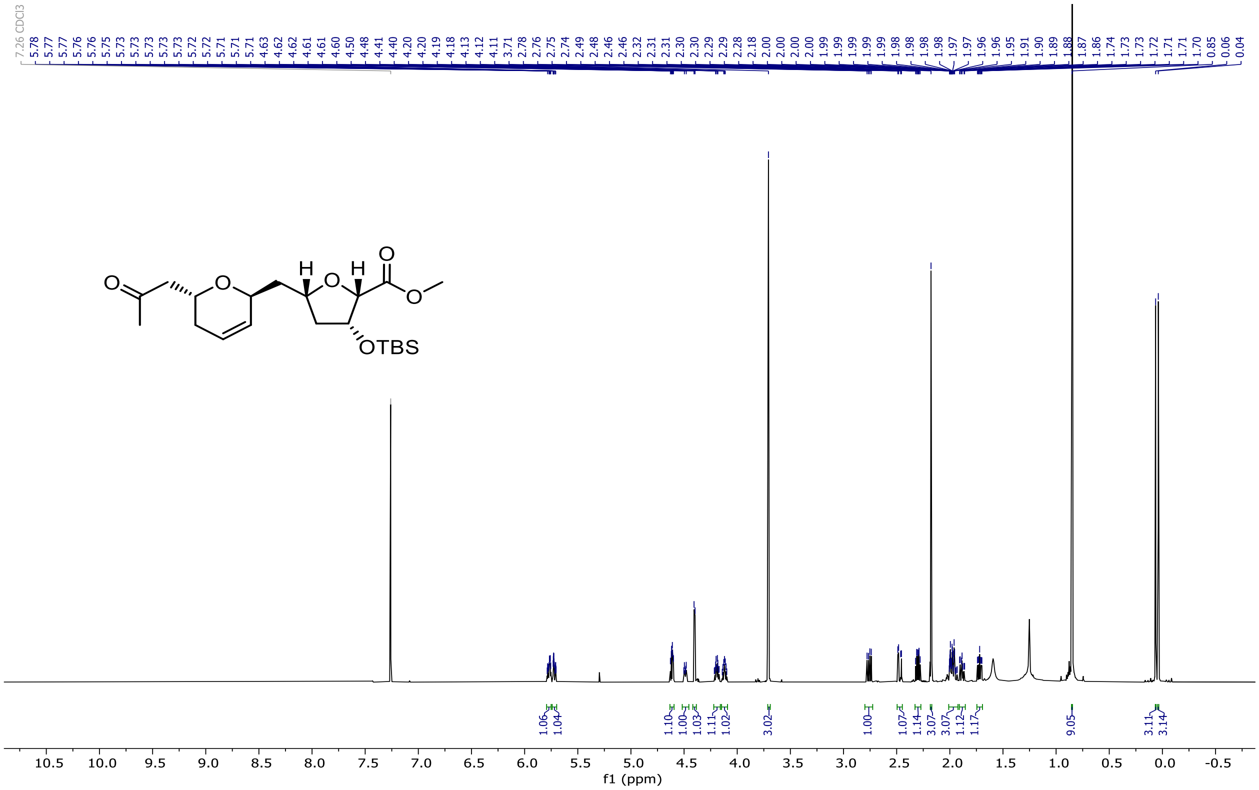

^{13}C NMR (151 MHz, $CDCl_3$)

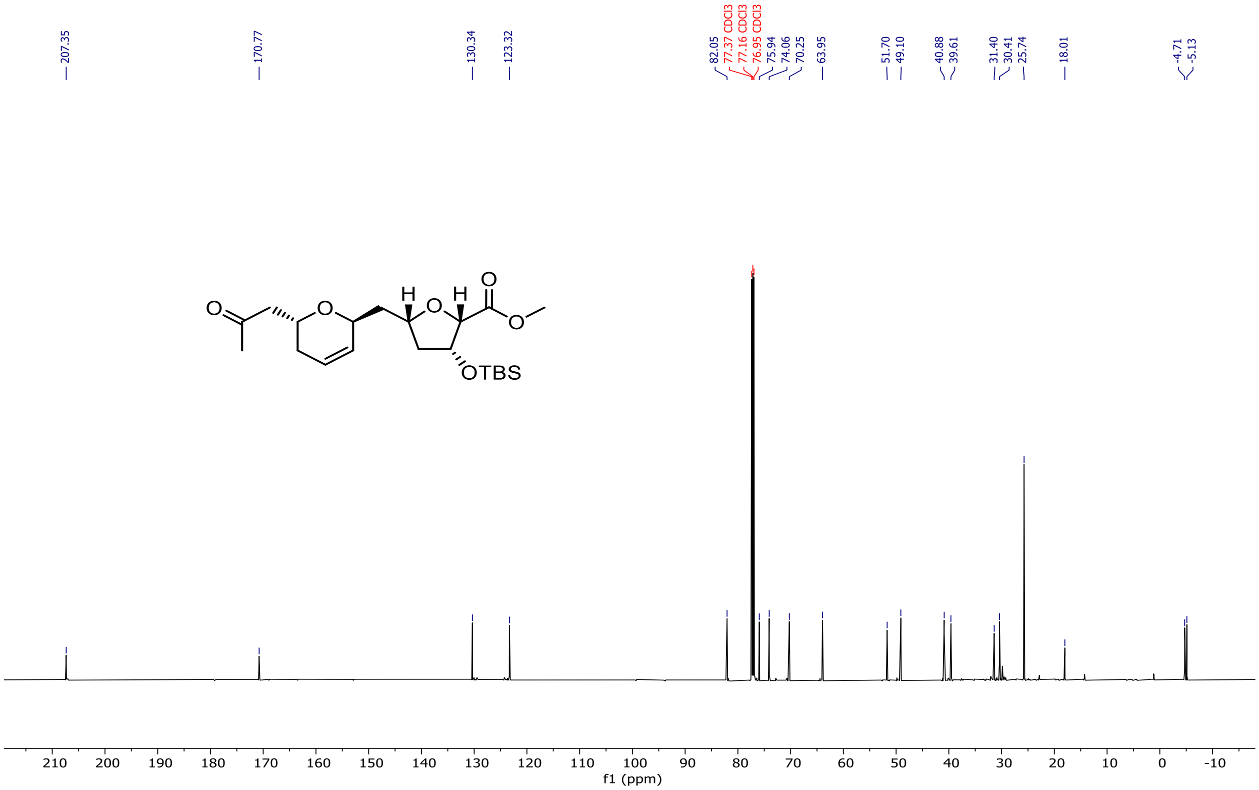

Compound 21: ^{1}H-^{1}H COSY ($CDCl_3$)

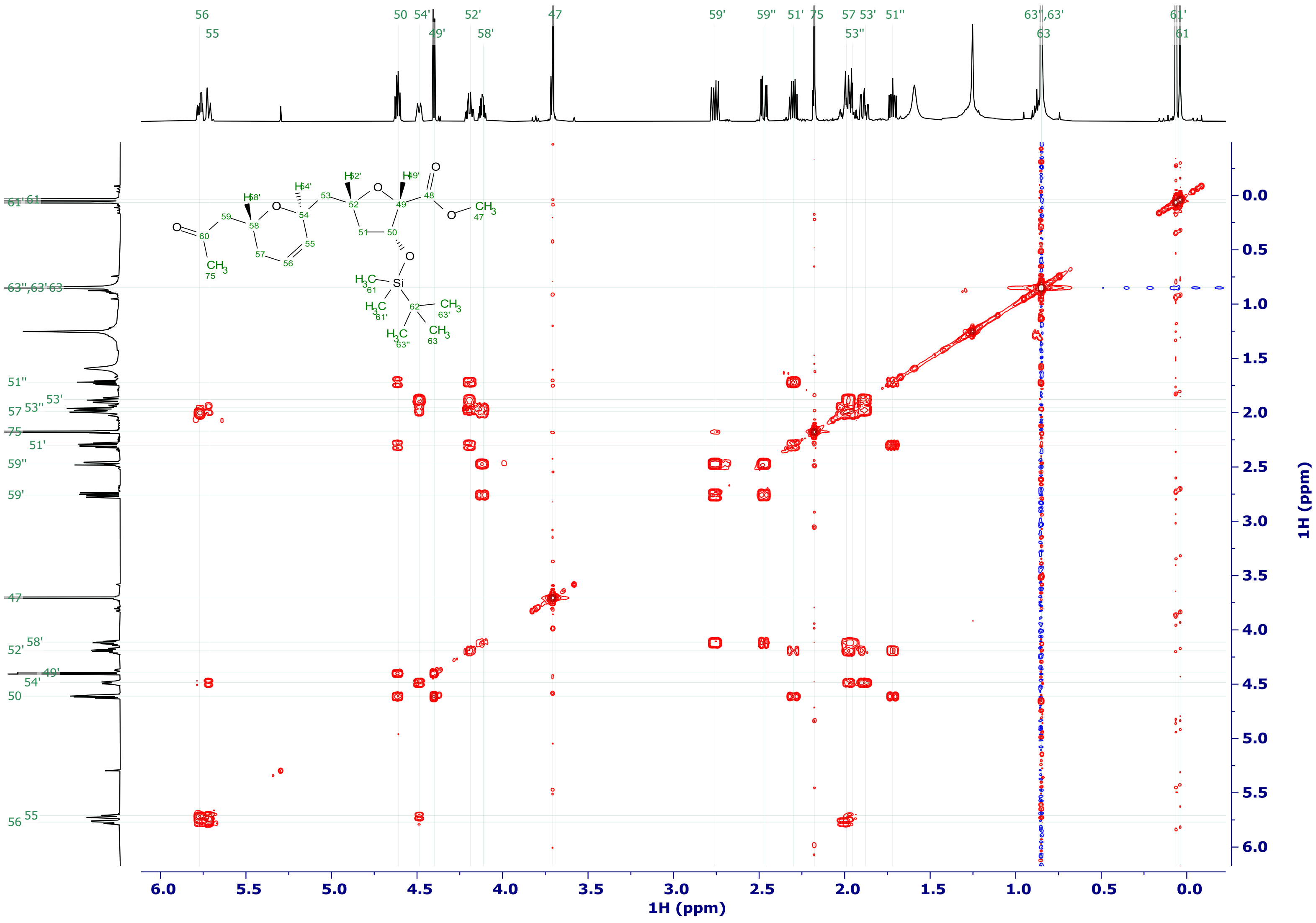

Compound 21: HSQC NMR ($CDCl_3$)

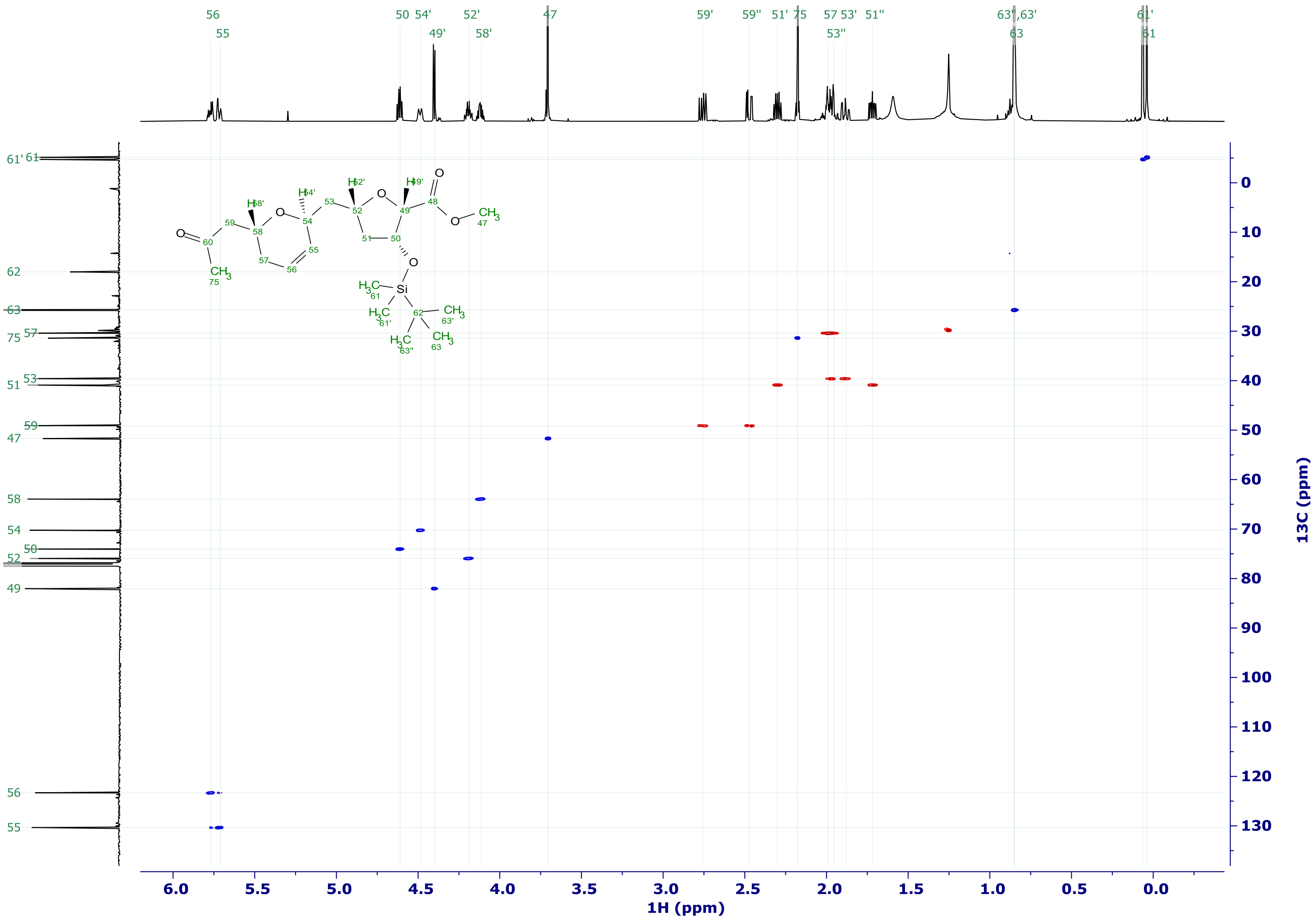

Compound 21: HMBC NMR ($CDCl_3$)

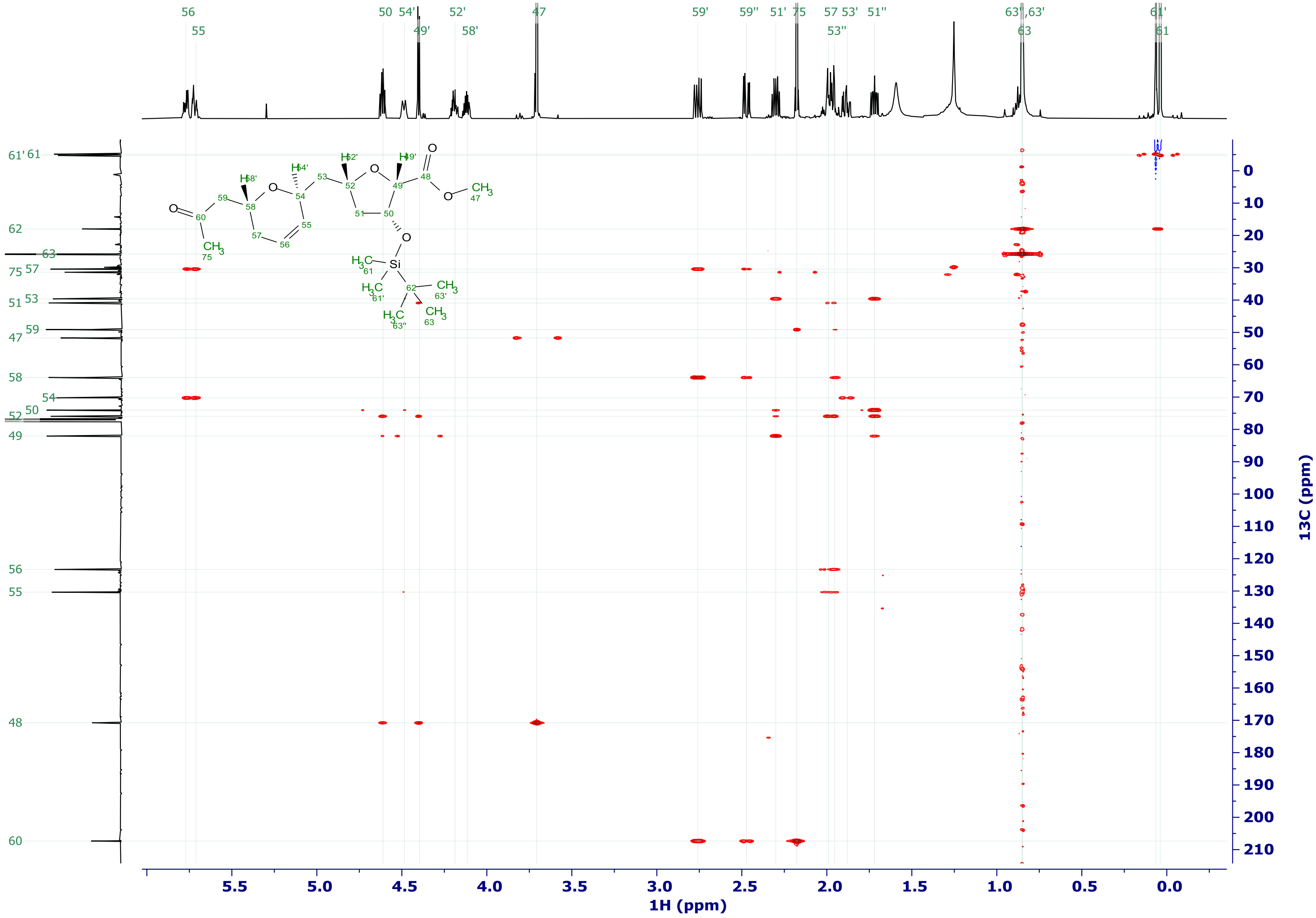

Compound 21: NOESY ($CDCl_3$)

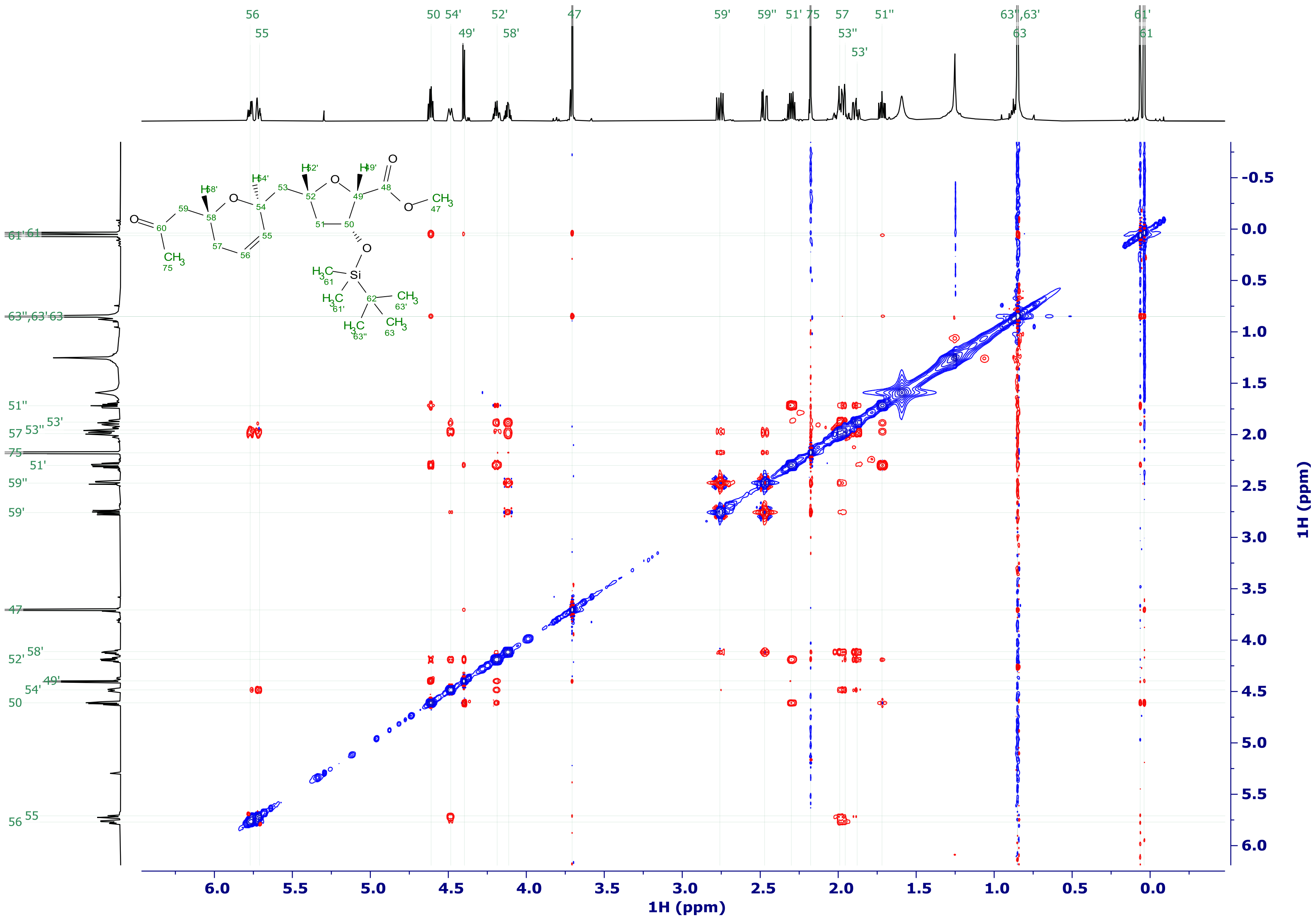

Compound S7: ^{1}H NMR (600 MHz, C_6D_6)

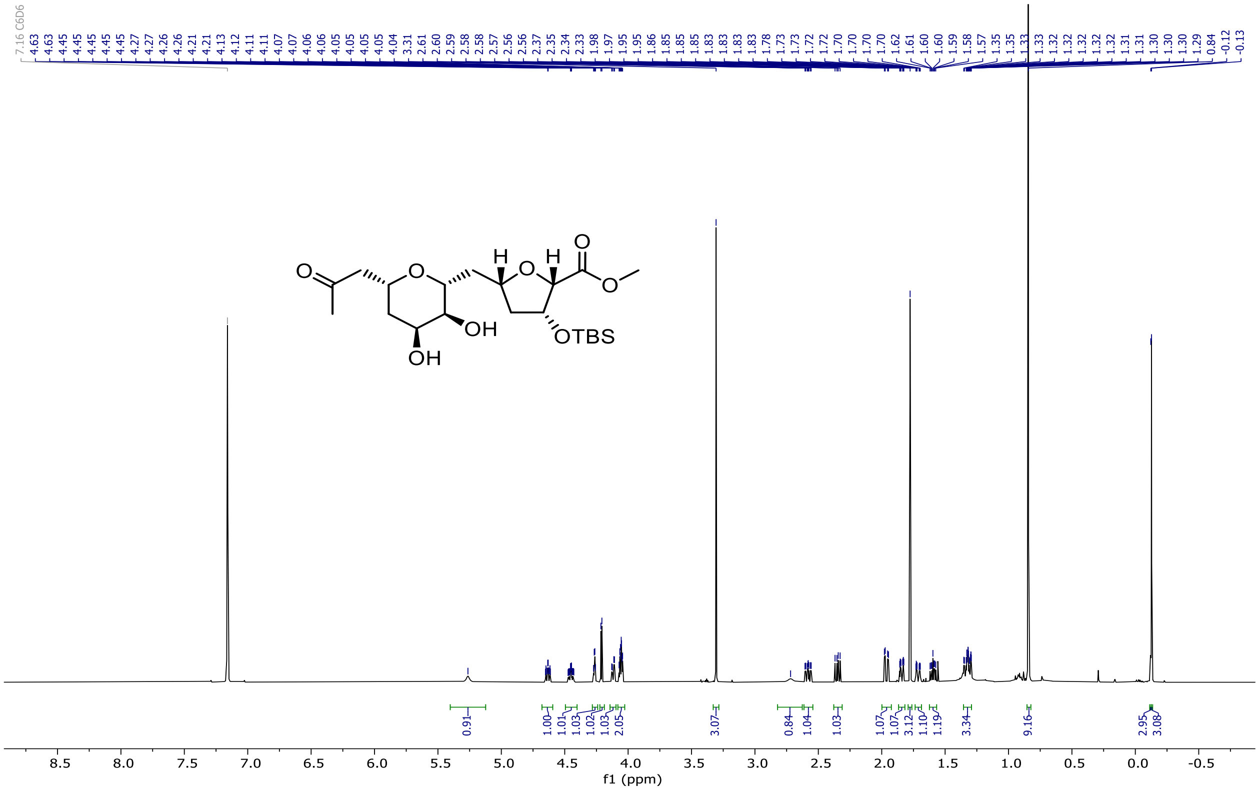

^{13}C NMR (151 MHz, C_6D_6)

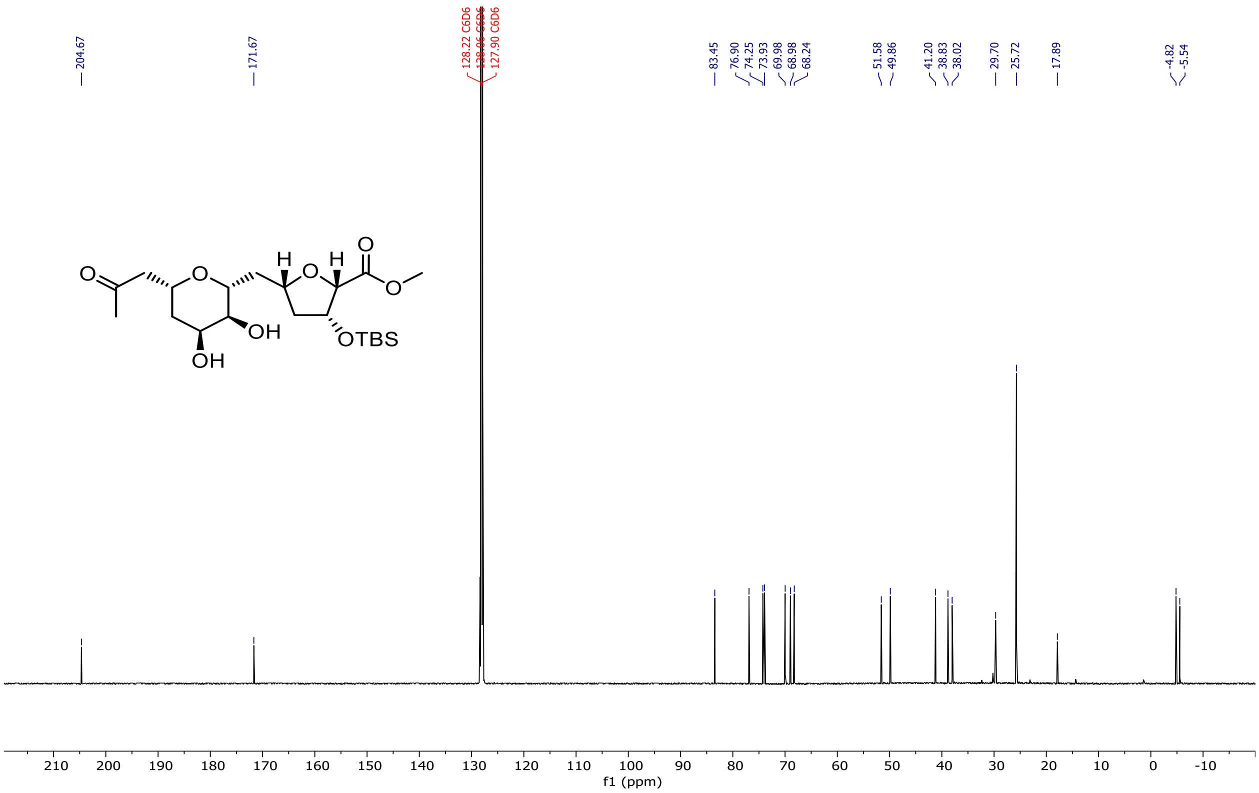

Compound S7: ^{1}H-^{1}H COSY (C_6D_6)

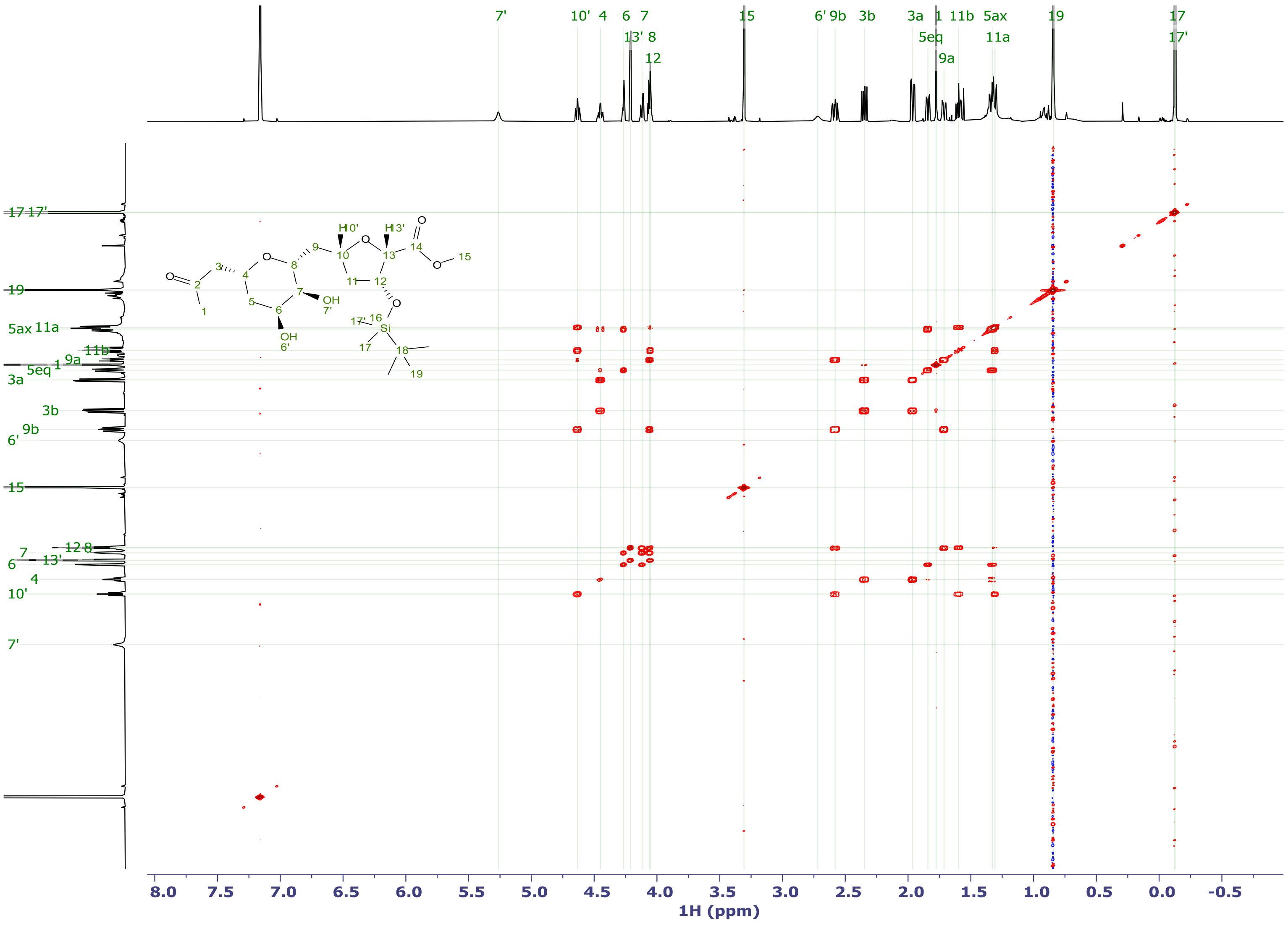

Compound S7: HSQC NMR (C_6D_6)

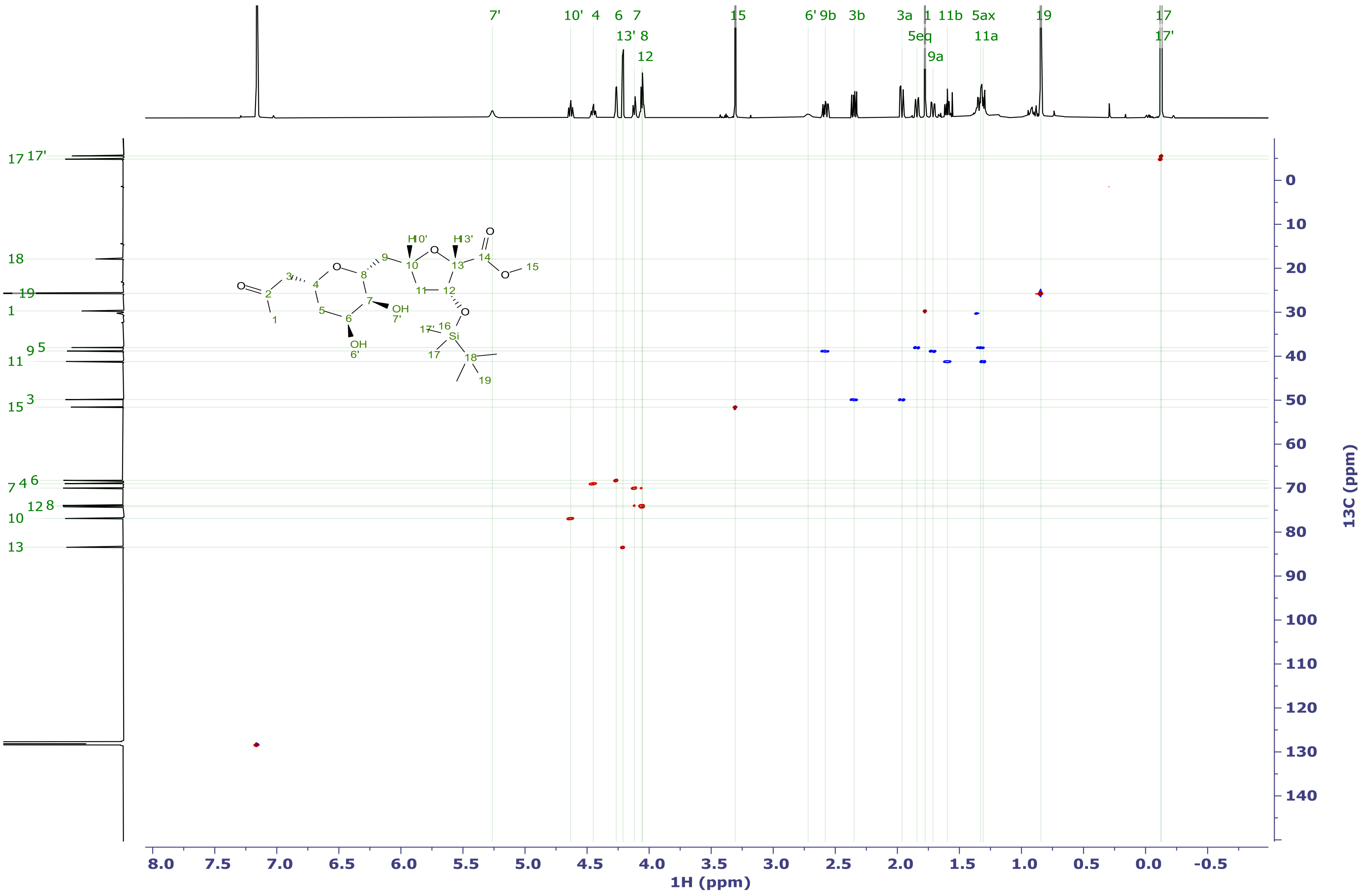

Compound S7: HMBC NMR (C_6D_6)

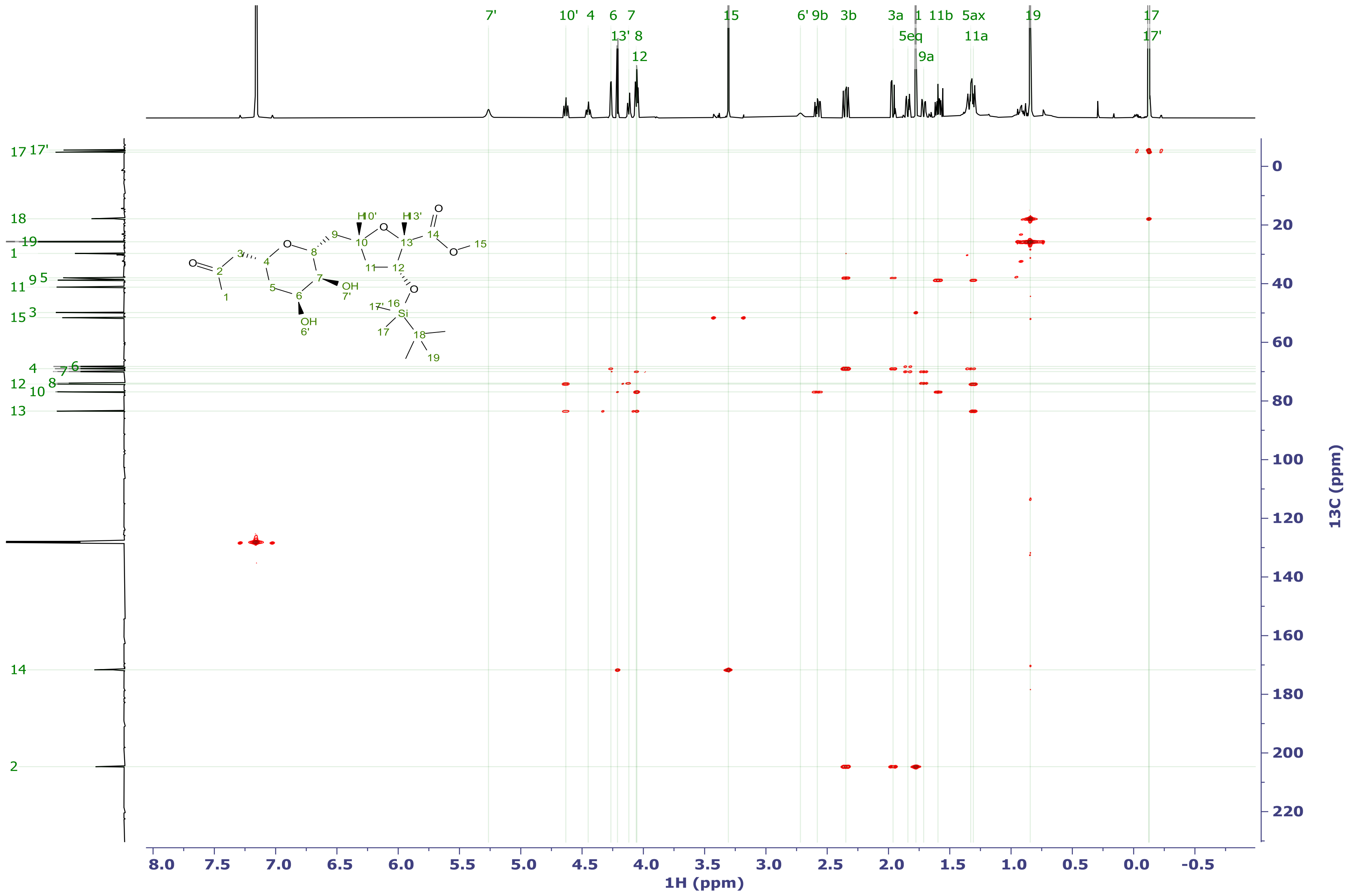

Compound S7: NOESY (C_6D_6)

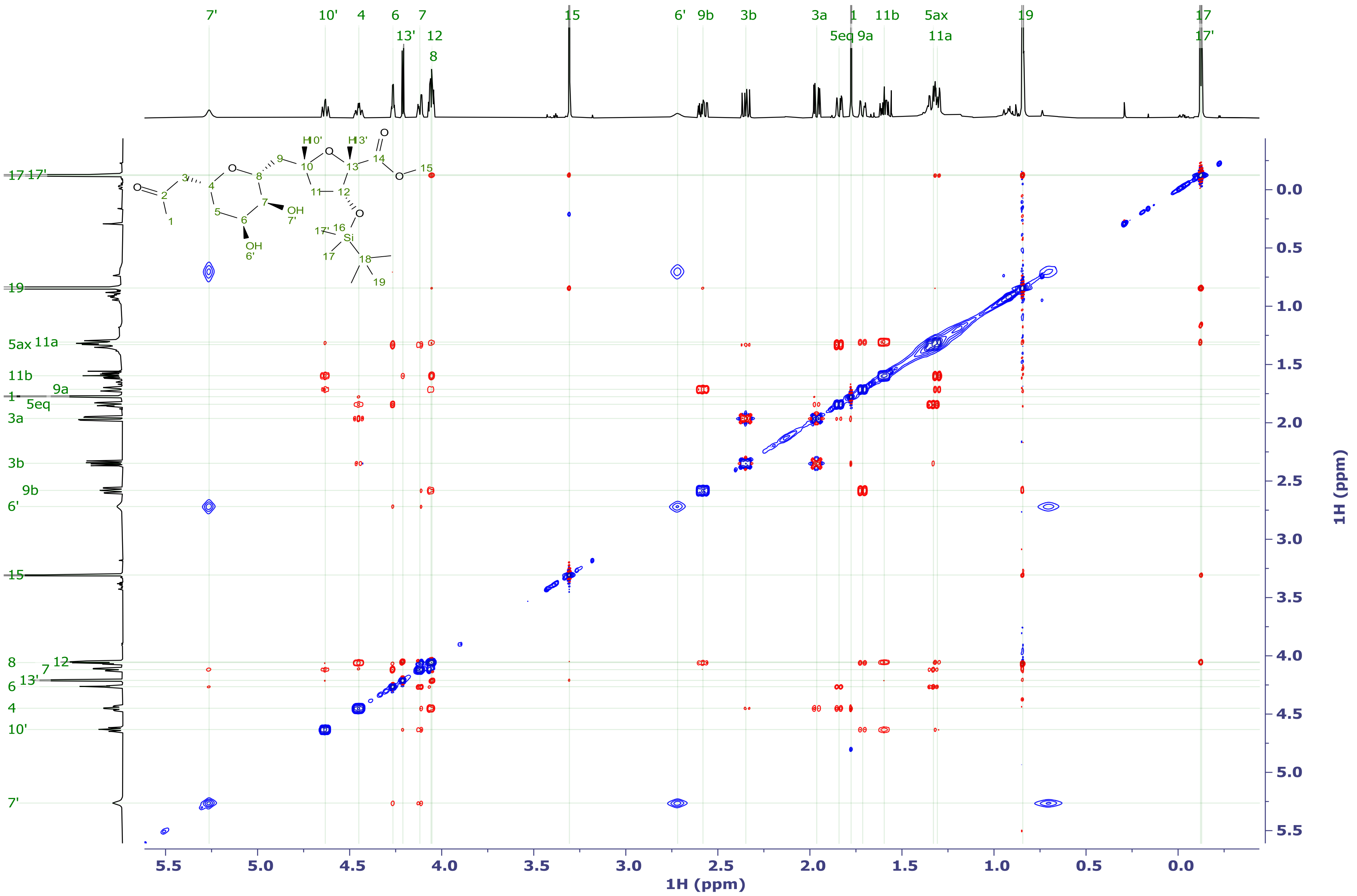

Compound 22: ^{1}H NMR (400 MHz, CDCl$_3$)

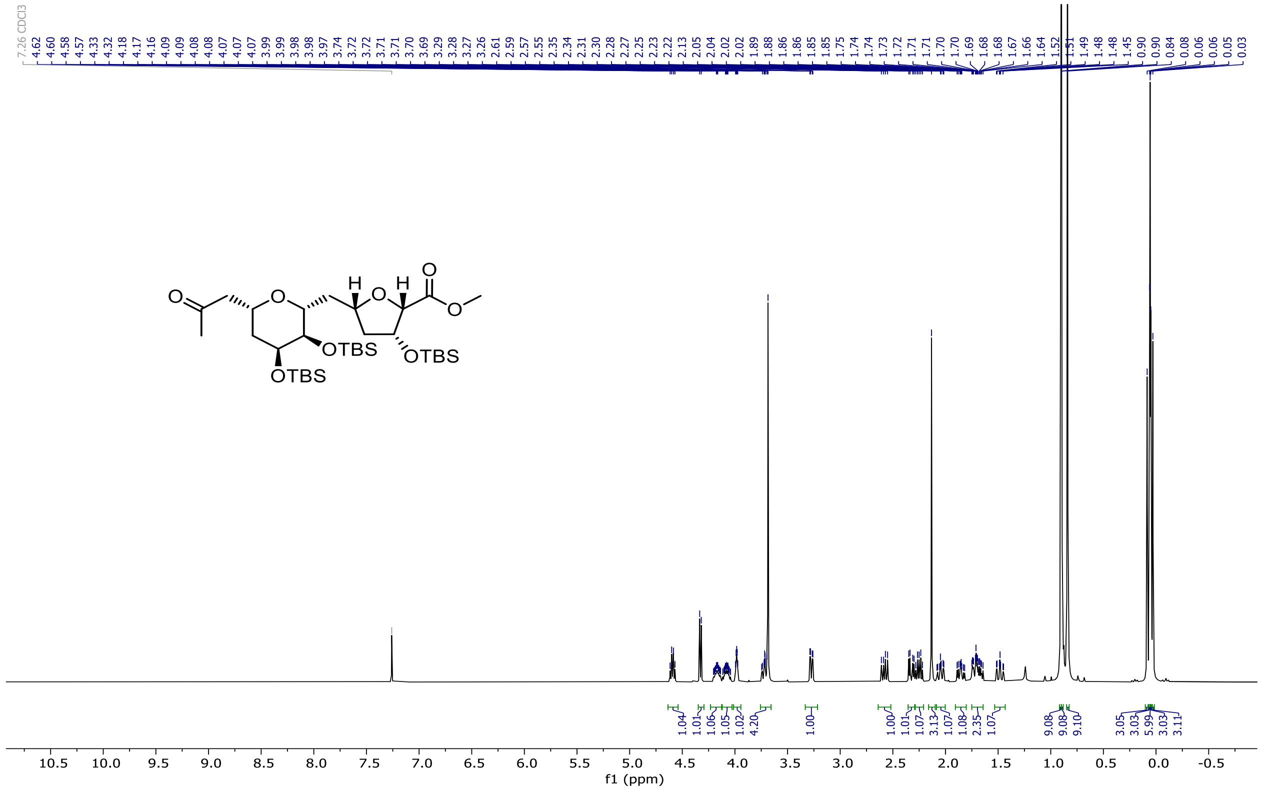

^{13}C NMR (101 MHz, CDCl$_3$)

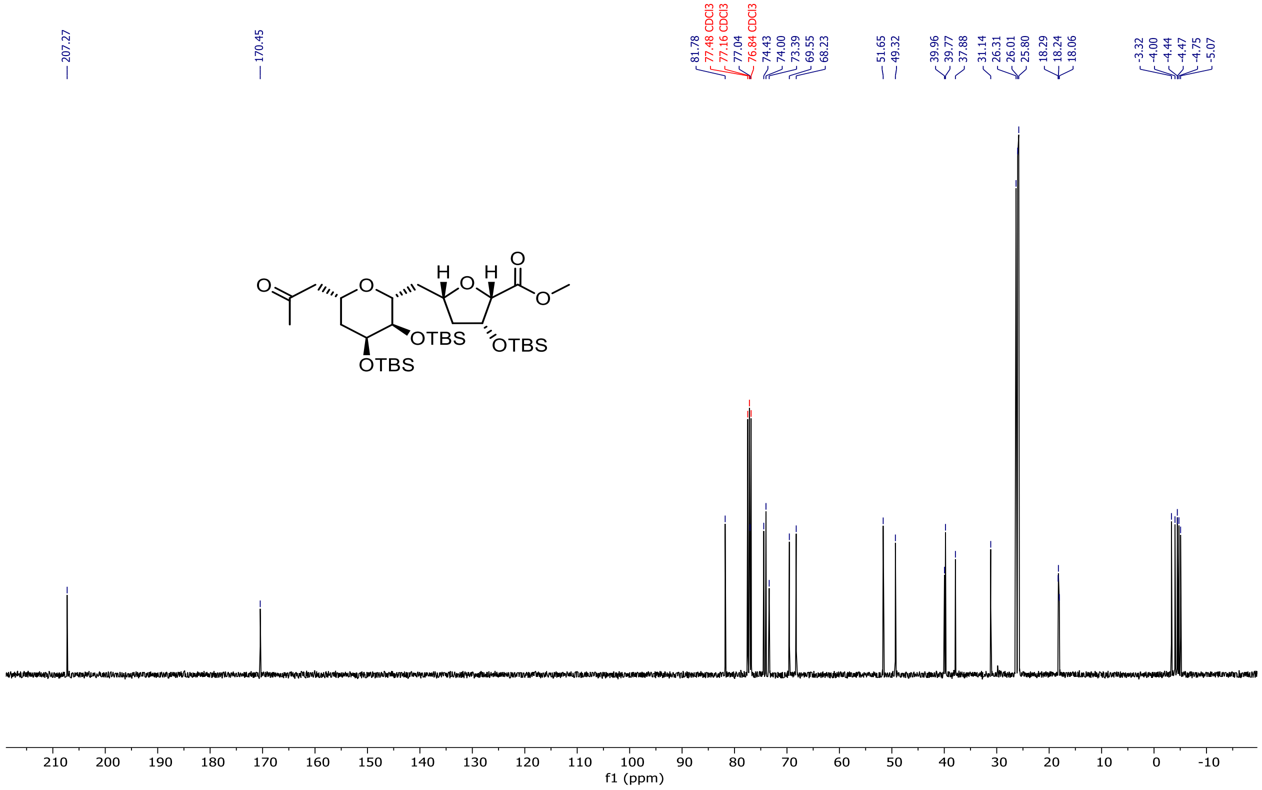

Compound *E*-S8: ^{1}H NMR (600 MHz, $CDCl_3$)

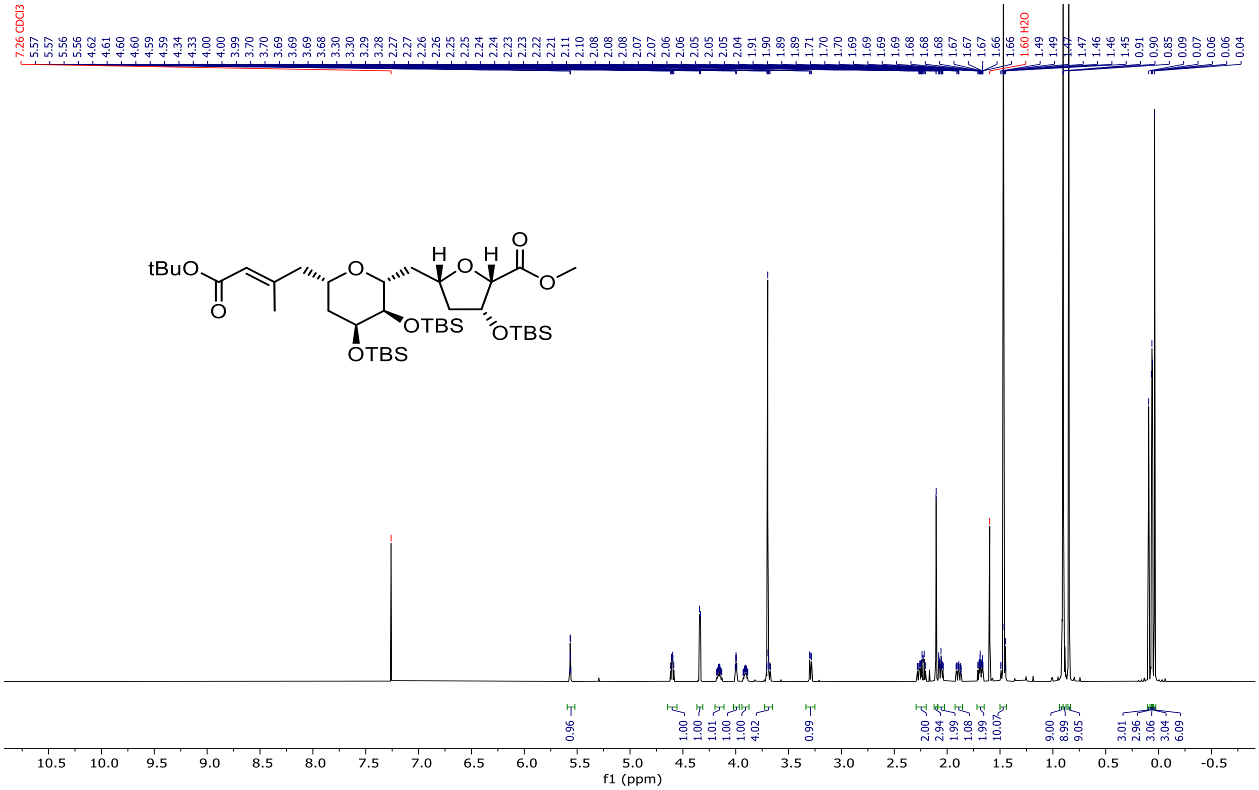

^{13}C NMR (151 MHz, $CDCl_3$)

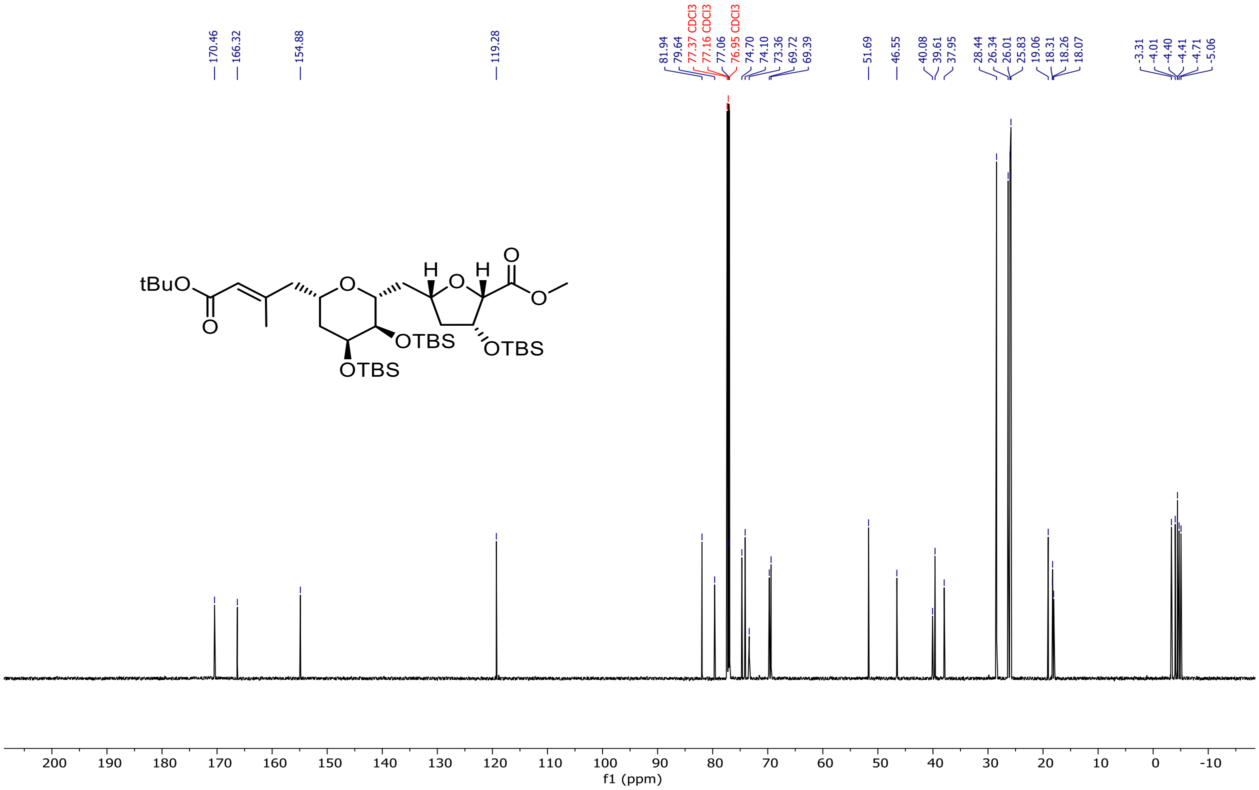

Compound *E*-S8: ^{1}H-^{1}H COSY ($CDCl_3$)

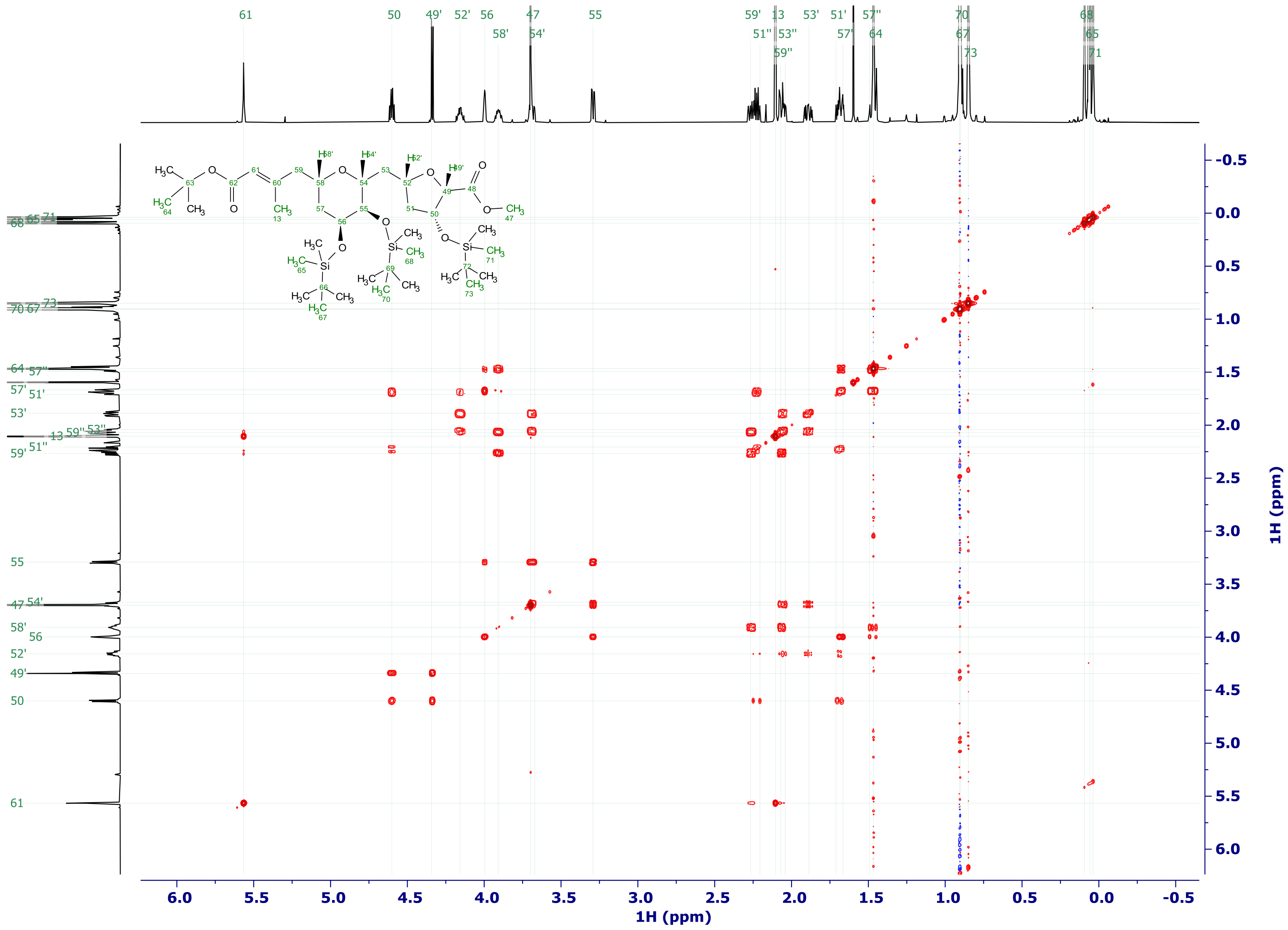

Compound *E*-S8: HSQC NMR ($CDCl_3$)

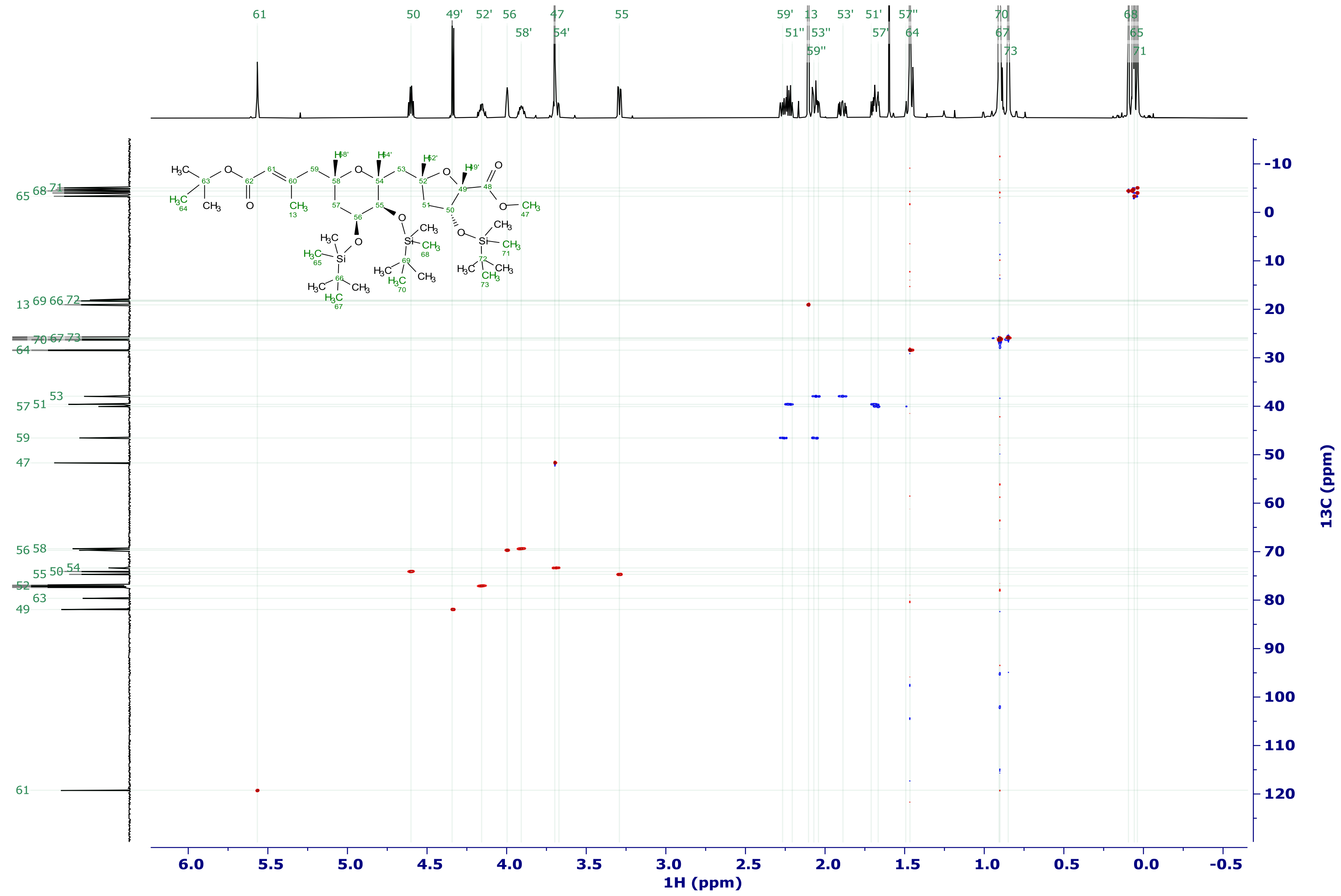

Compound *E*-S8: HMBC NMR ($CDCl_3$)

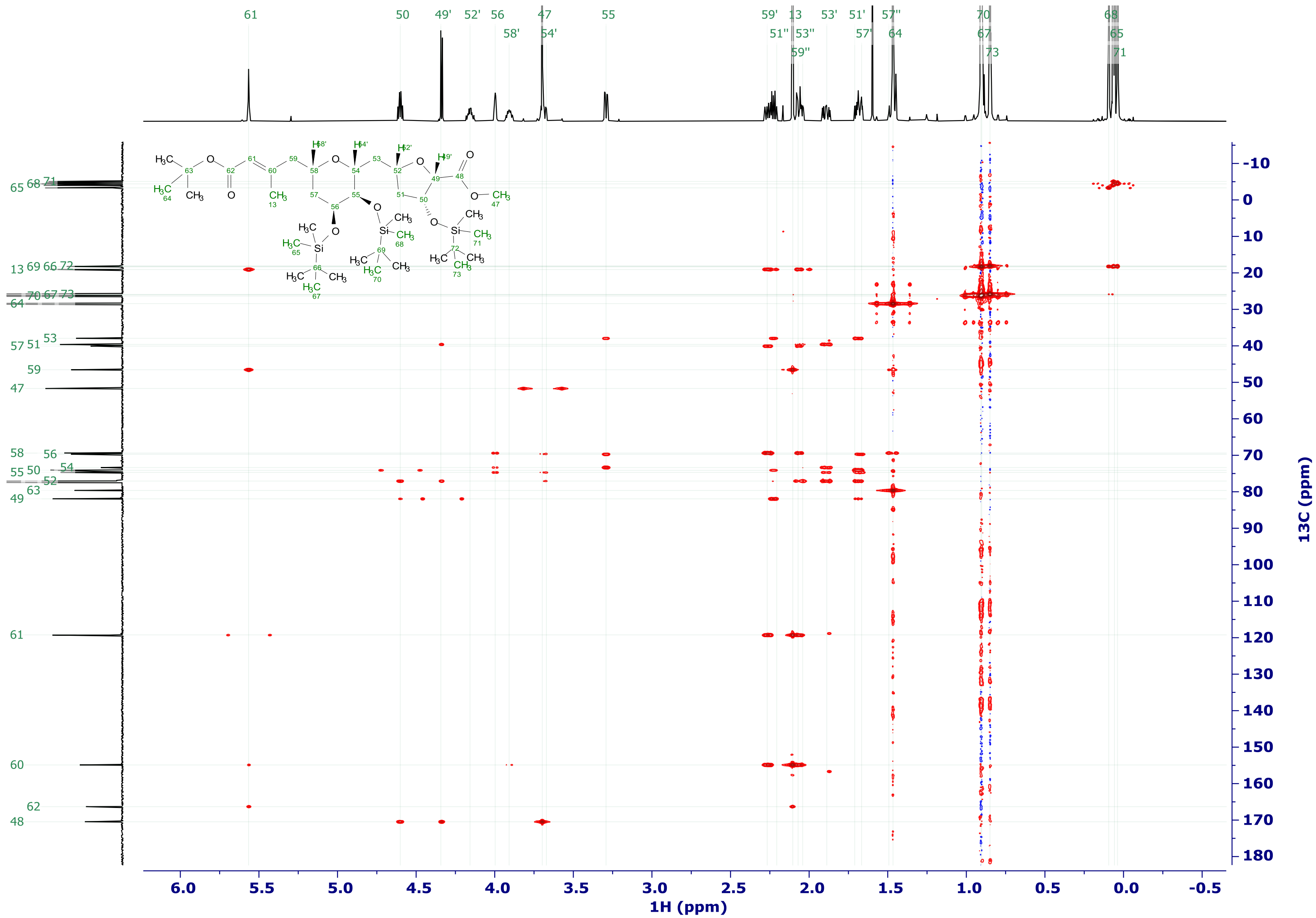

Compound *E*-S8: NOESY ($CDCl_3$)

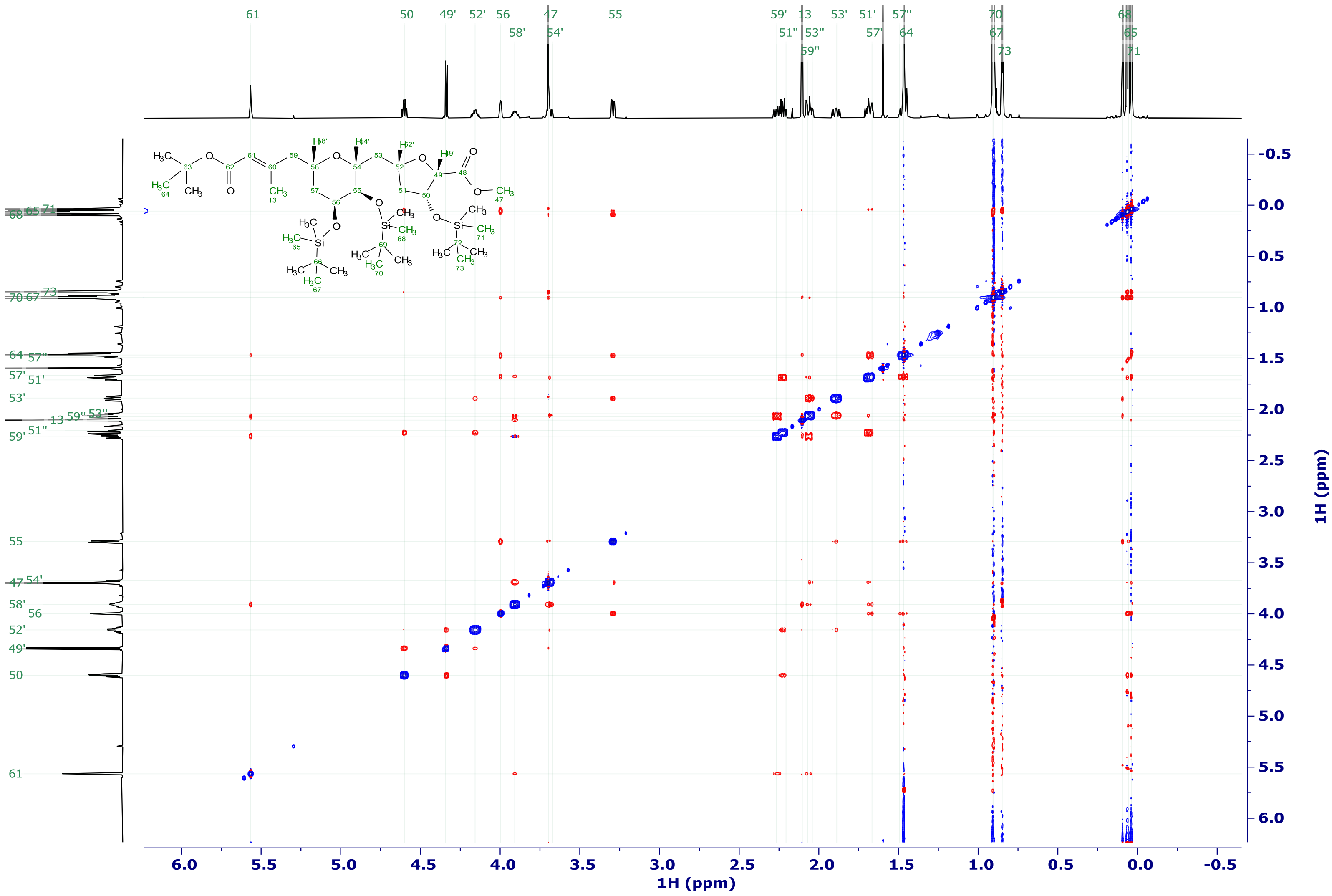

Compound *Z*-S8: ^{1}H NMR (600 MHz, $CDCl_3$)

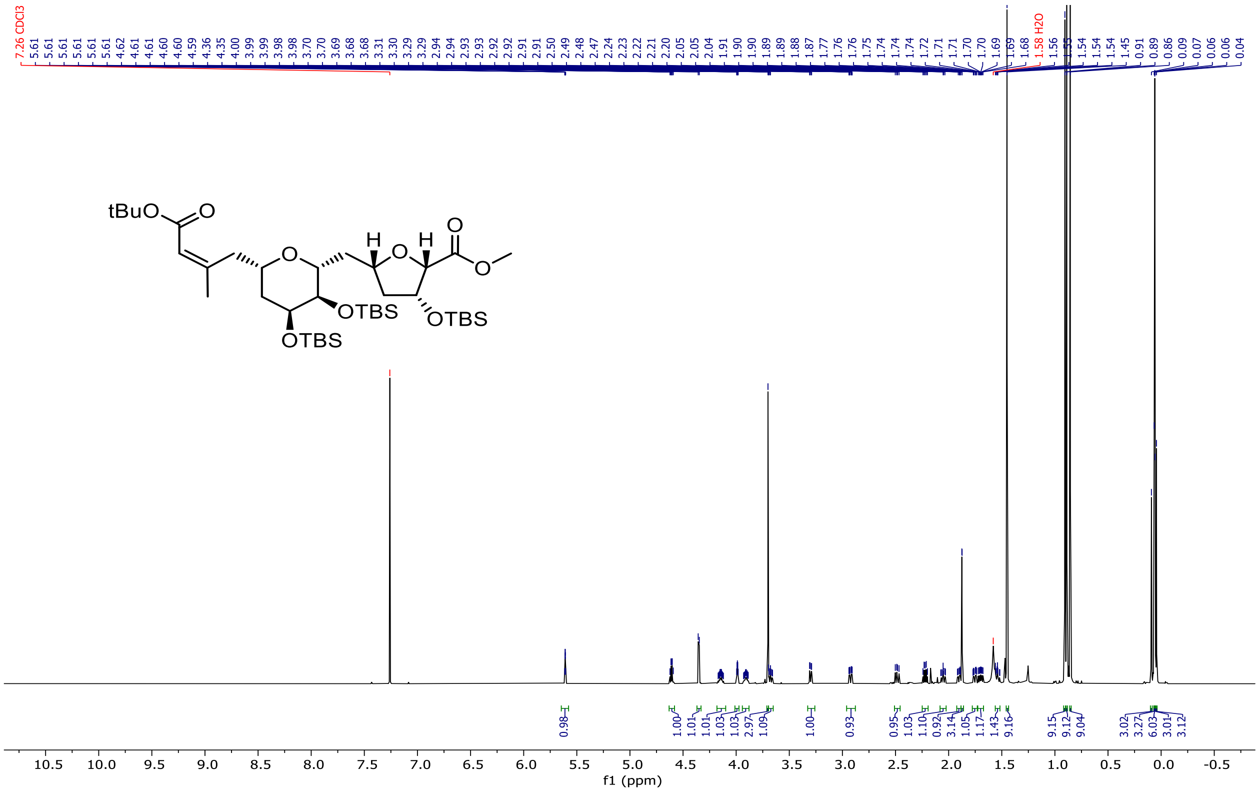

^{13}C NMR (151 MHz, $CDCl_3$)

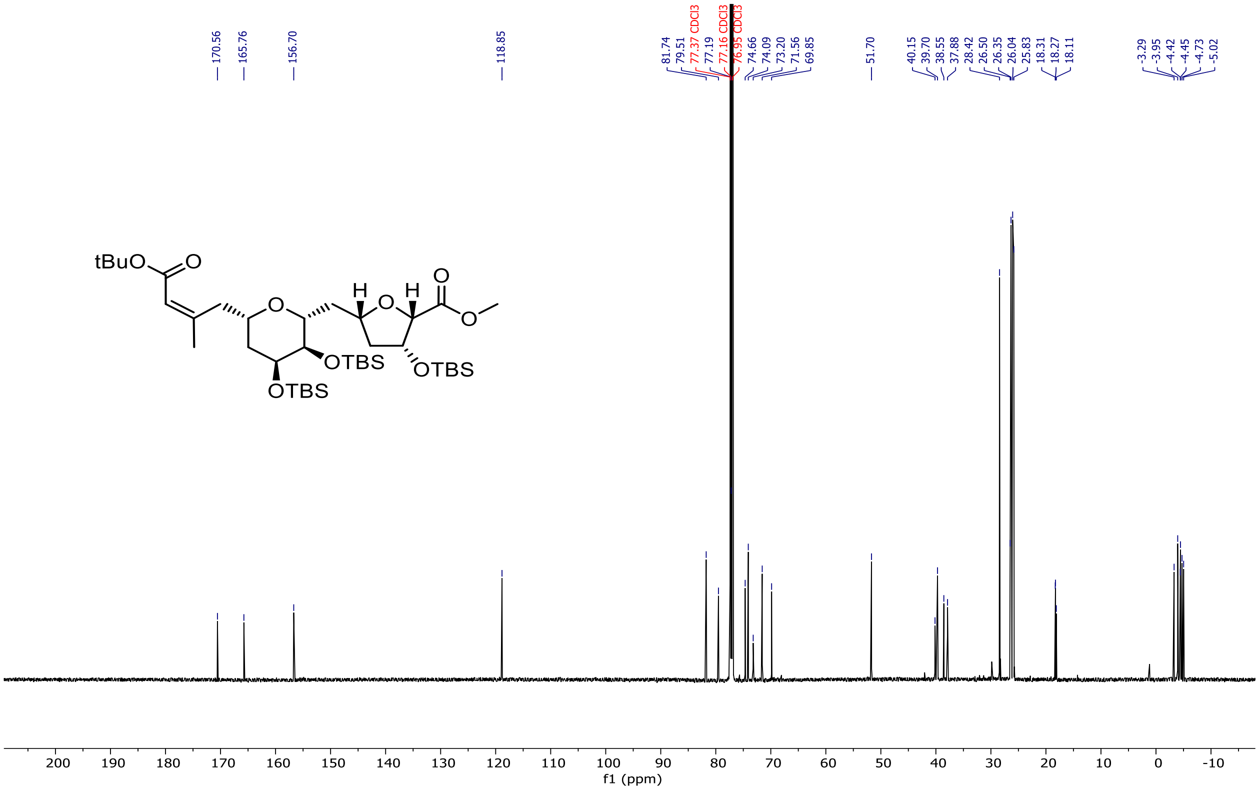

Compound *Z*-S8: ^{1}H-^{1}H COSY ($CDCl_3$)

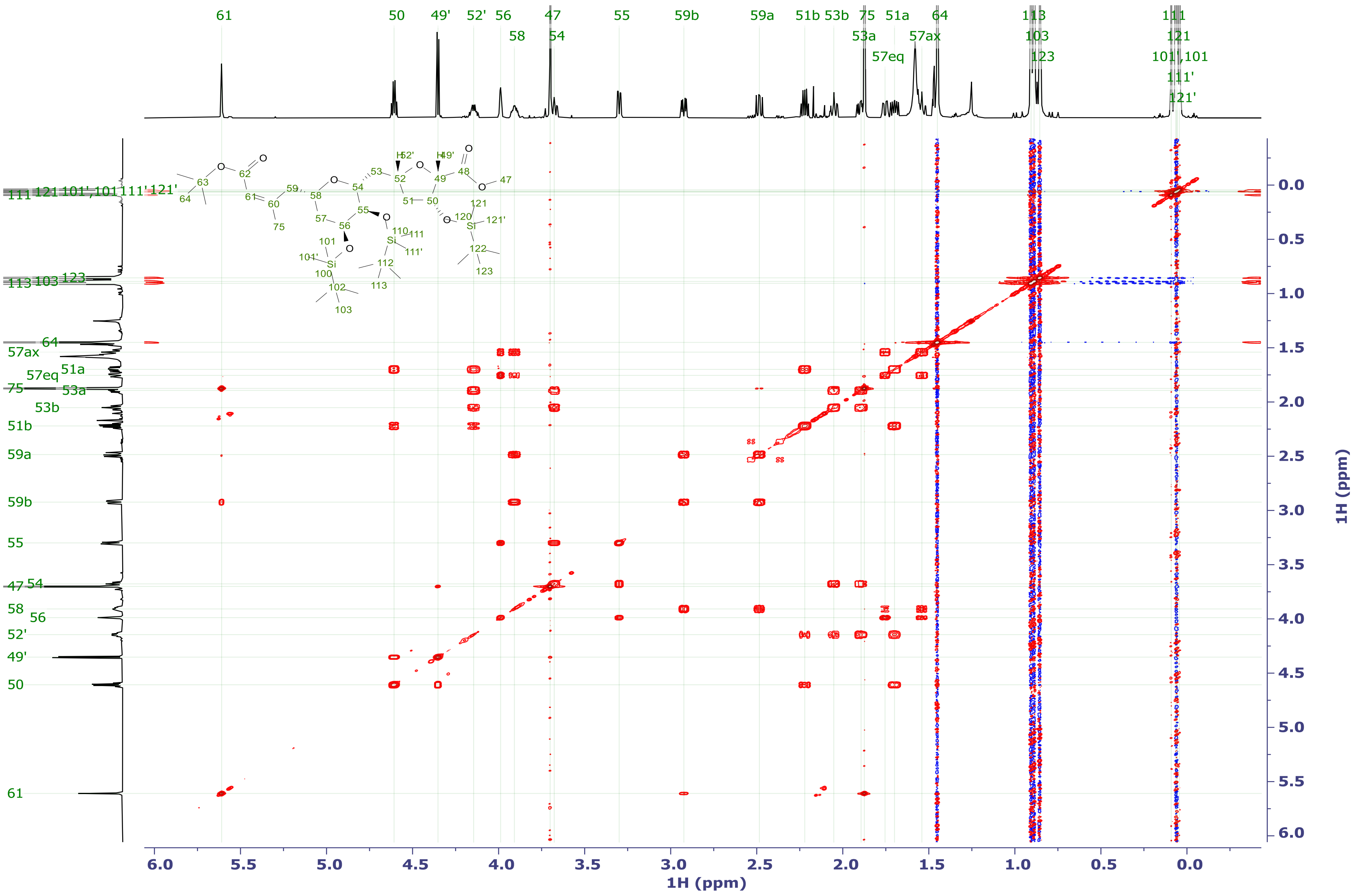

Compound *Z*-S8: HSQC NMR ($CDCl_3$)

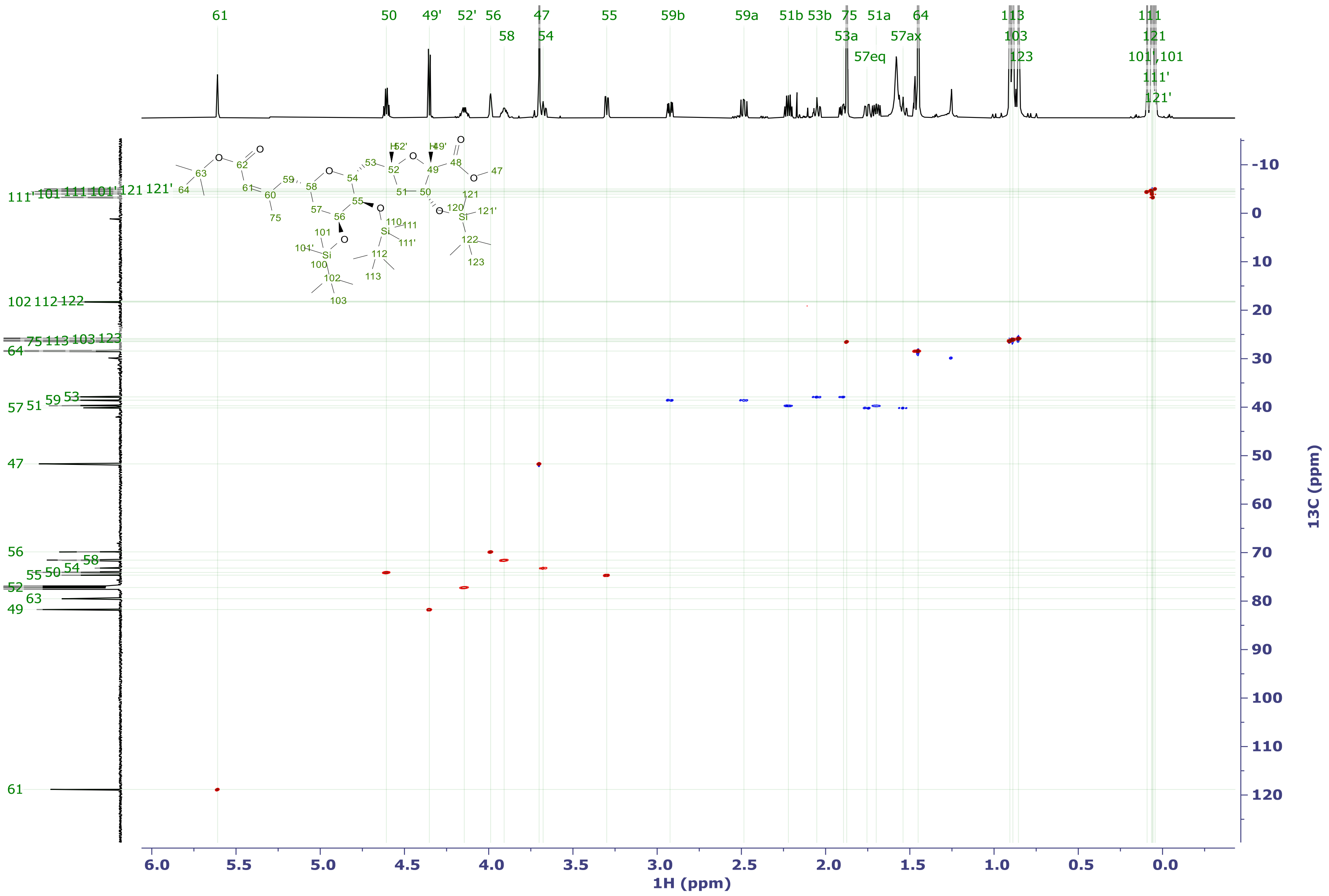

Compound *Z*-S8: HMBC NMR ($CDCl_3$)

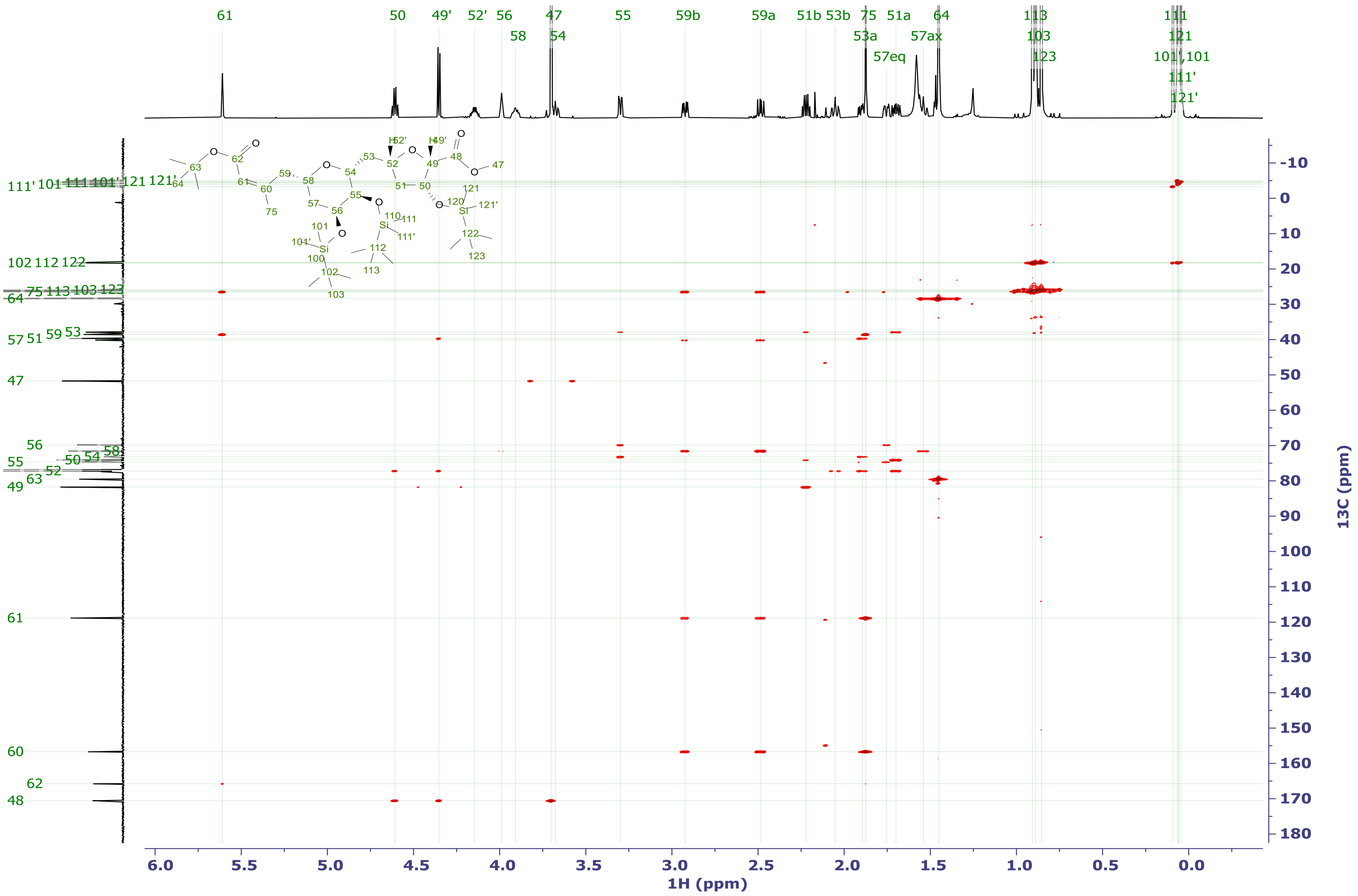

Compound *Z*-S8: NOESY ($CDCl_3$)

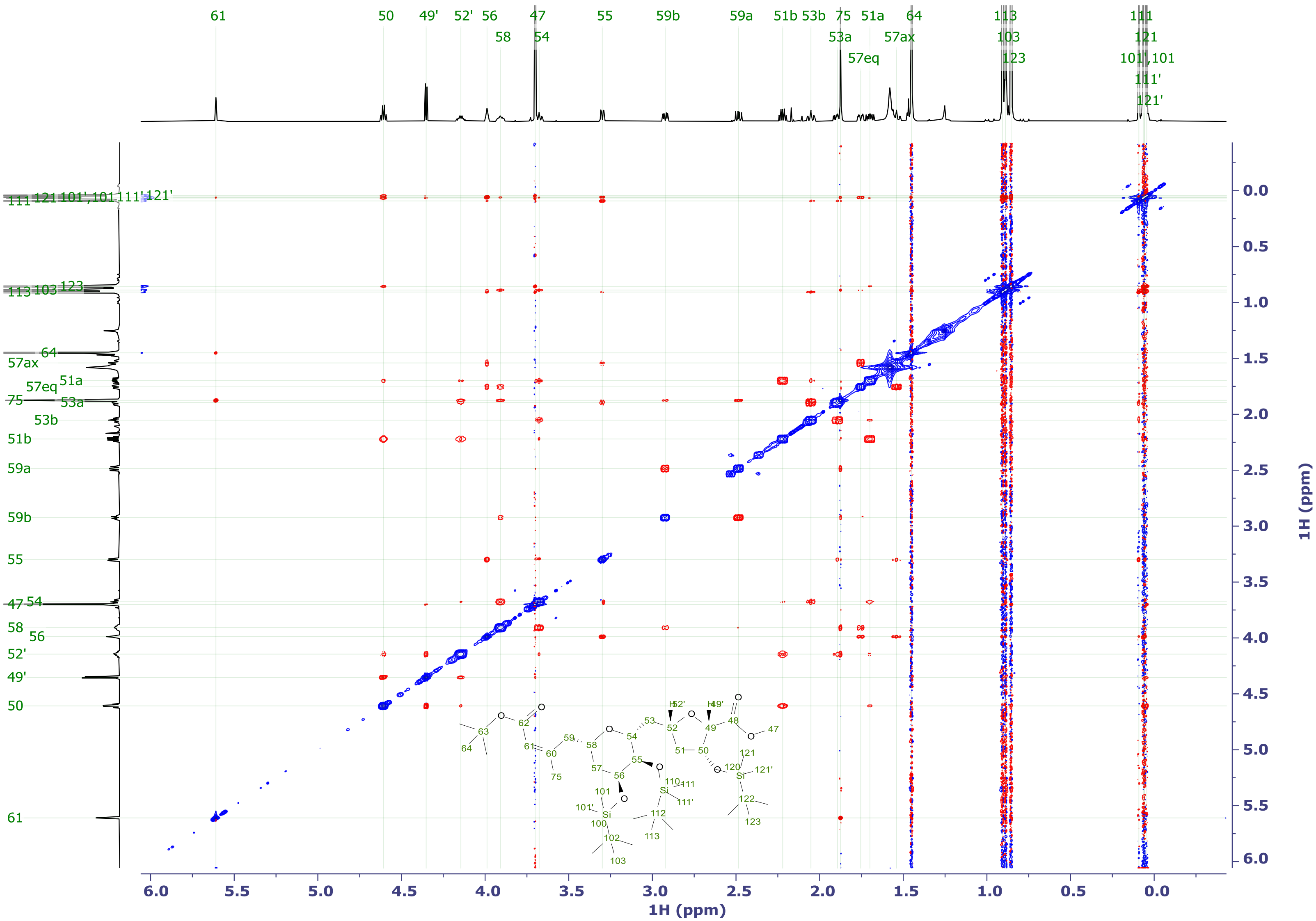

Compound 25: ^{1}H NMR (400 MHz, $CDCl_3$)

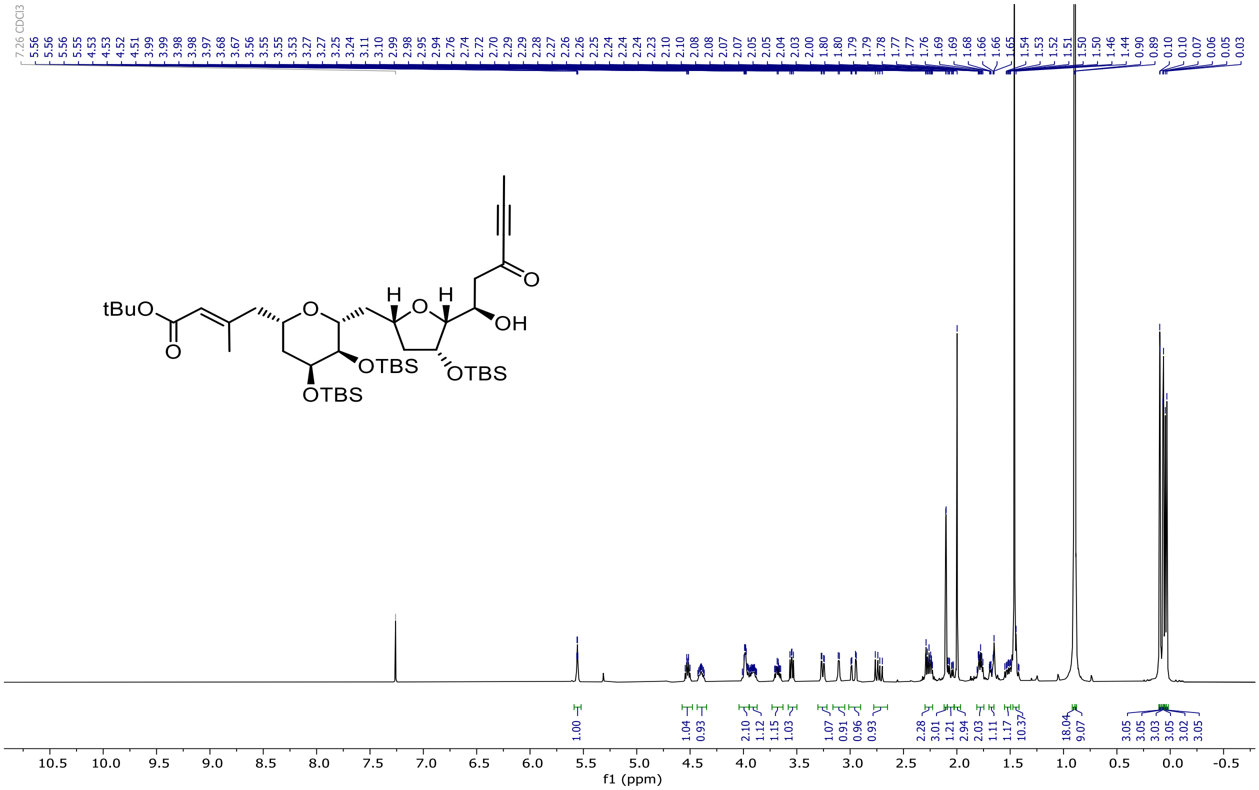

^{13}C NMR (101 MHz, $CDCl_3$)

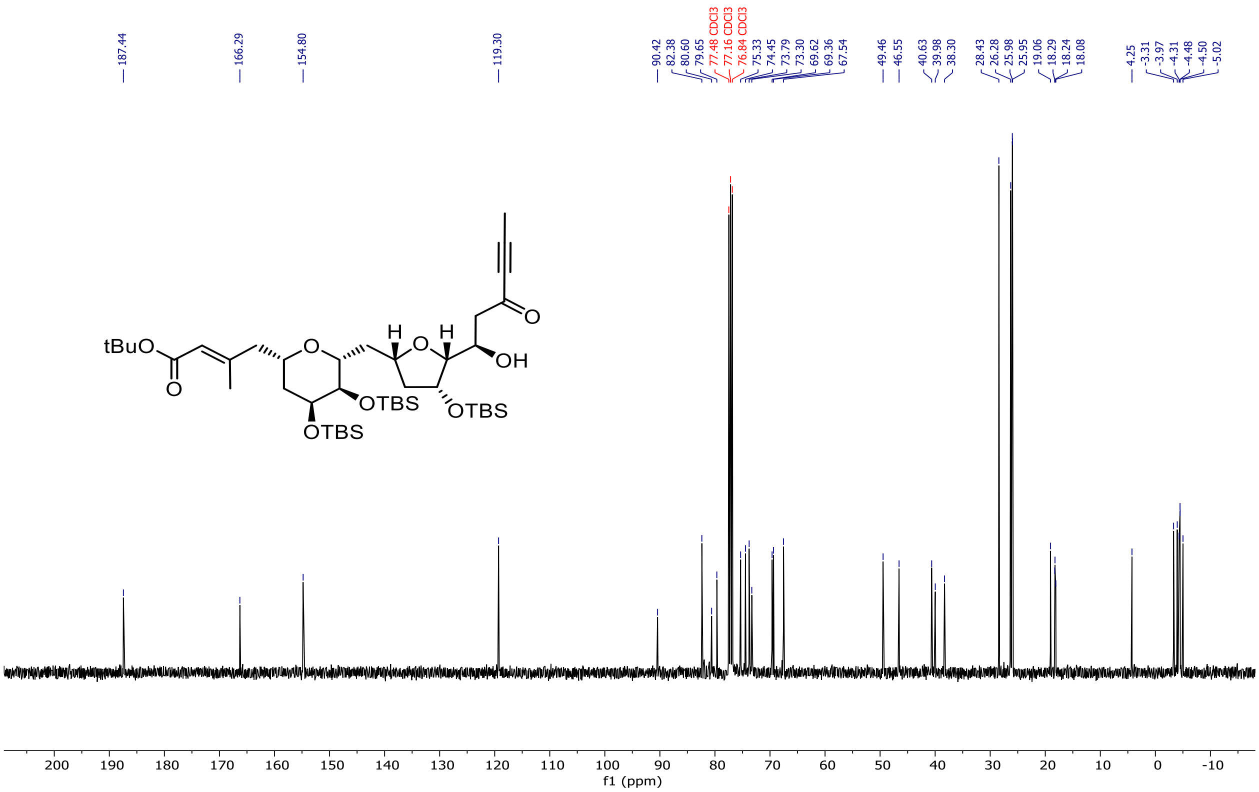

Compound S9: ^{1}H NMR (400 MHz, $CDCl_3$)

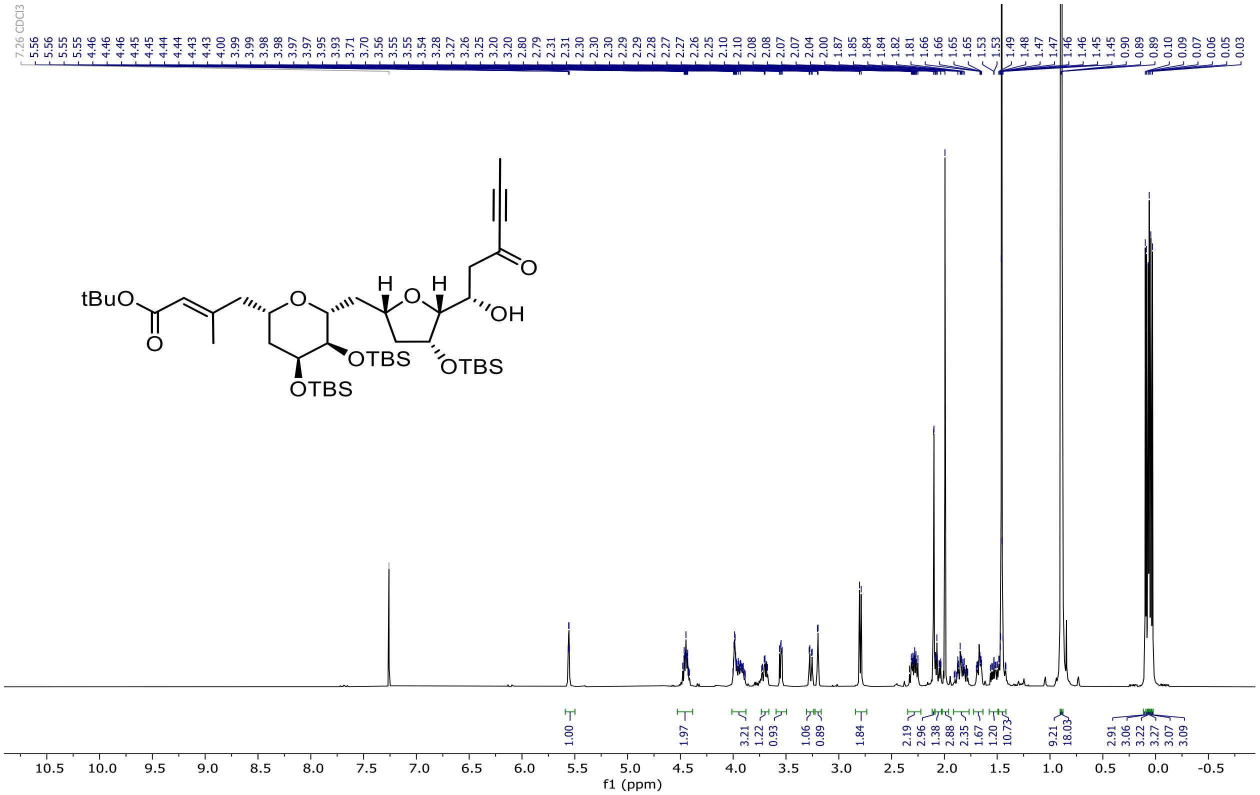

^{13}C NMR (101 MHz, $CDCl_3$)

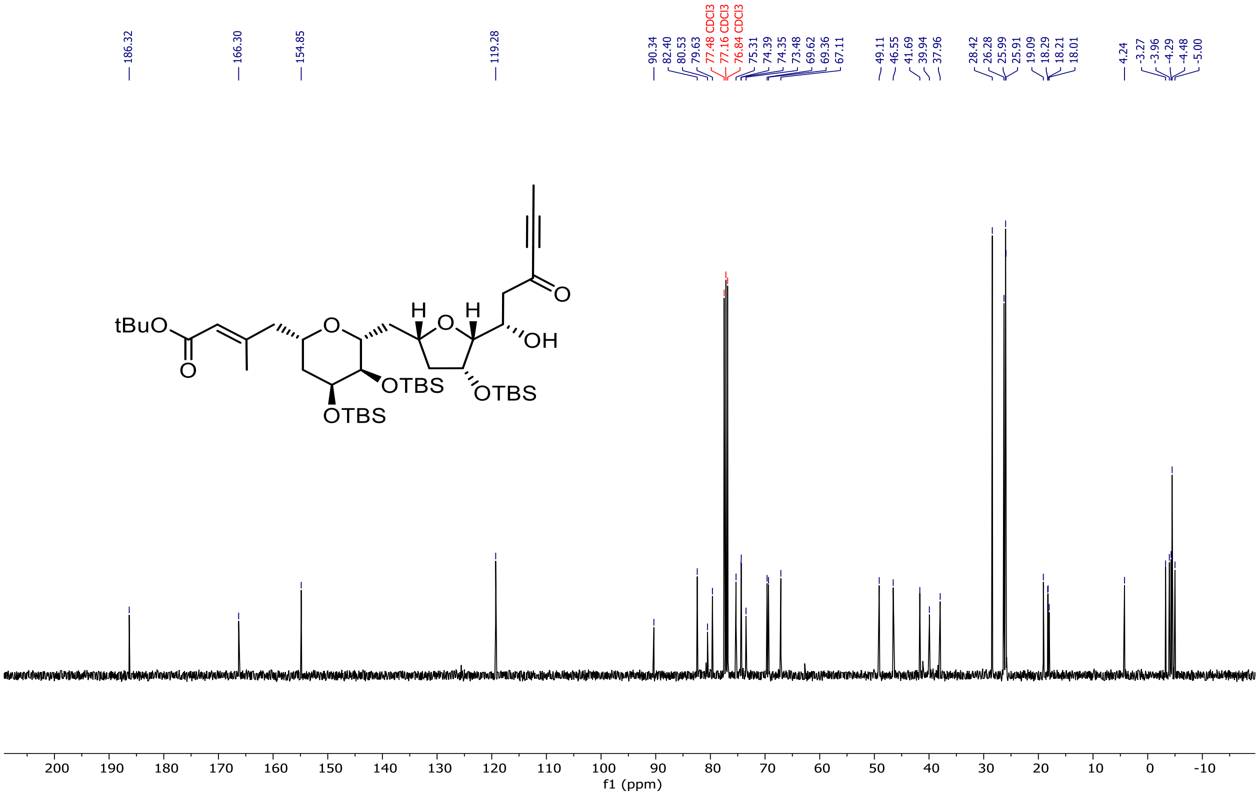

Compound 26: ^{1}H NMR (400 MHz, $CDCl_3$)

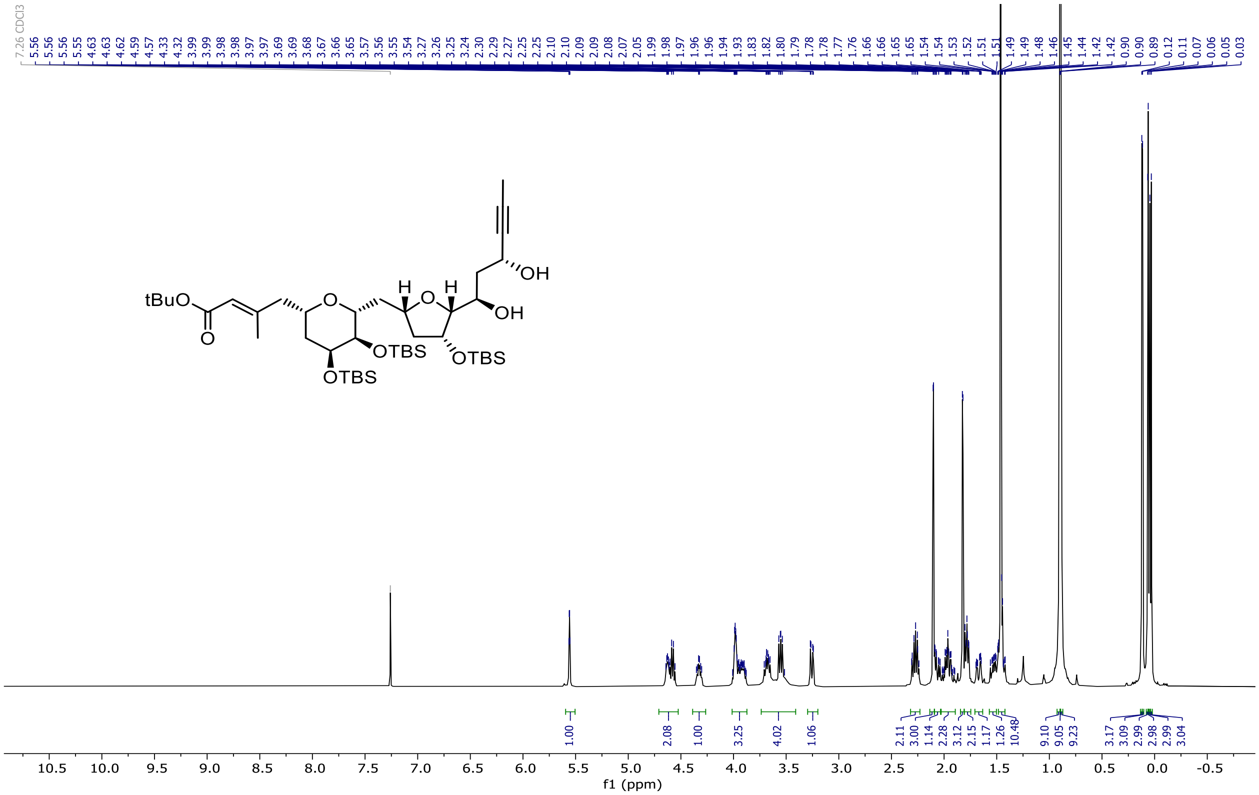

^{13}C NMR (101 MHz, $CDCl_3$)

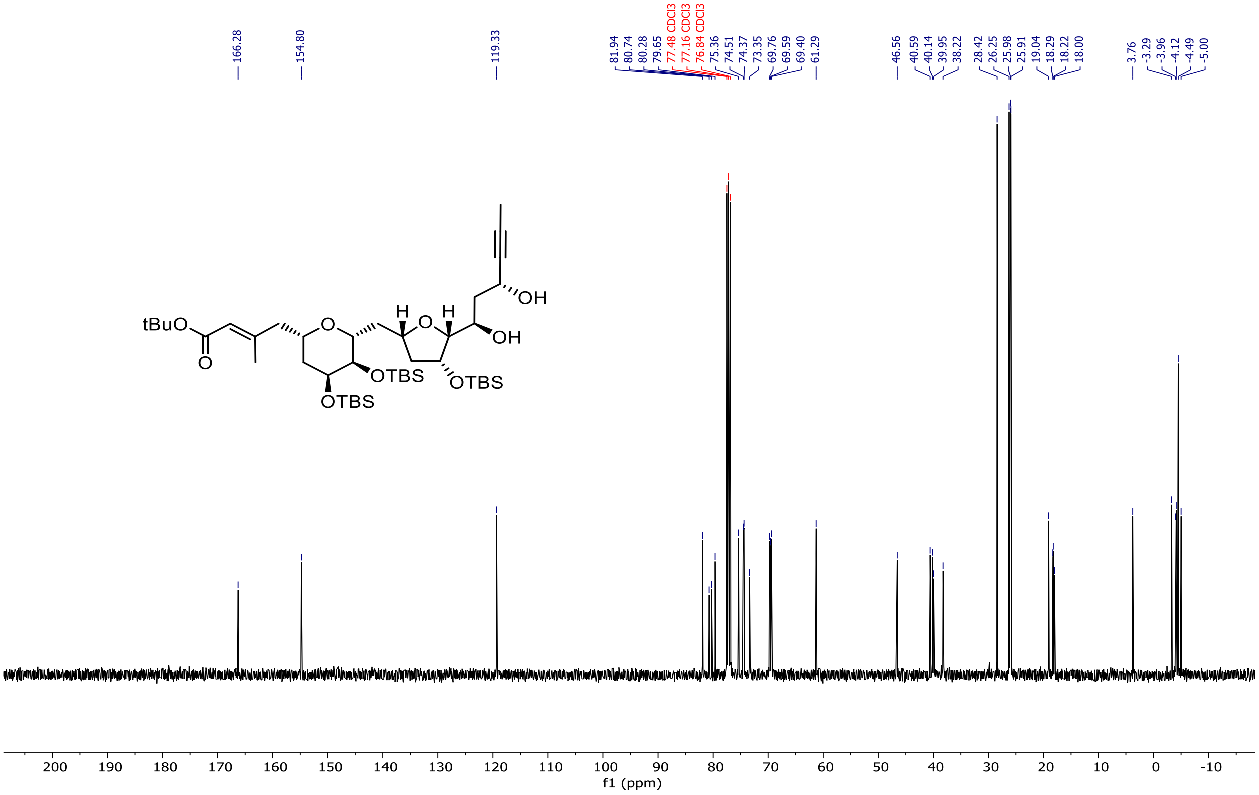

Compound S10: ^{1}H NMR (400 MHz, $CDCl_3$)

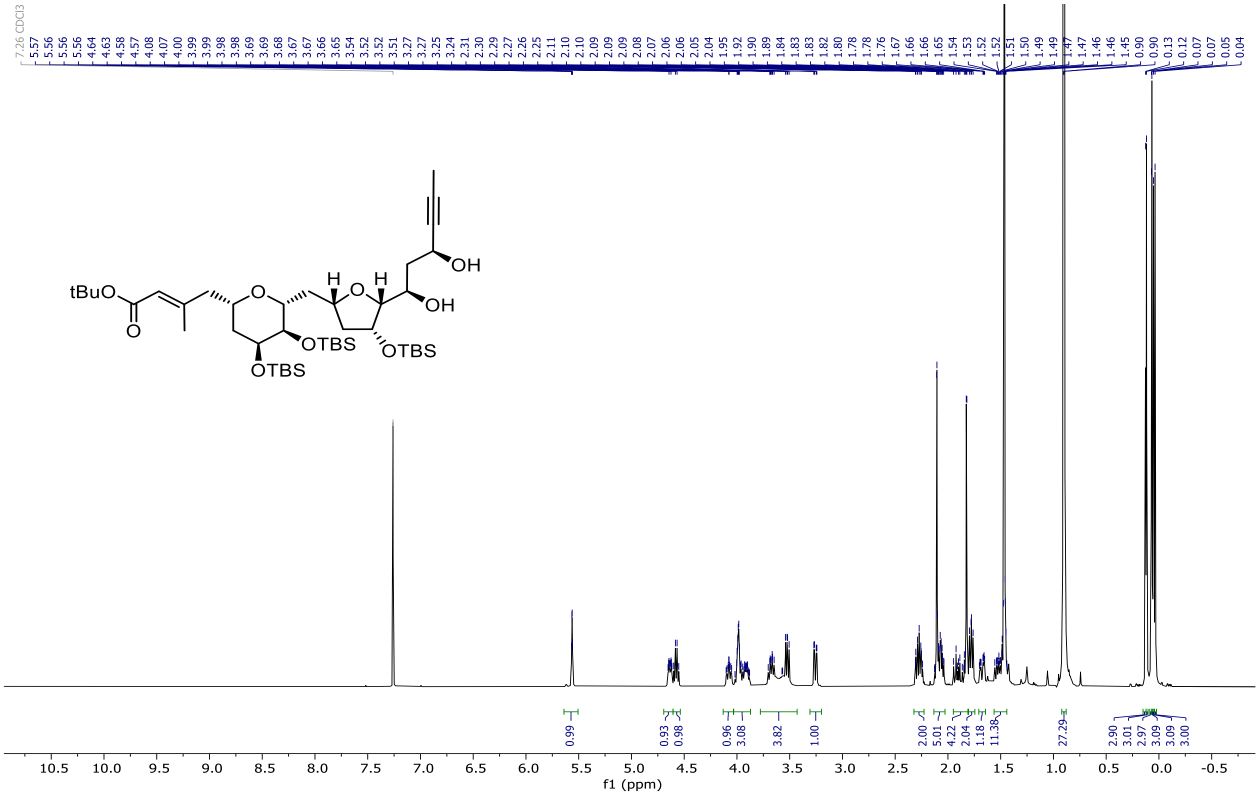

^{13}C NMR (101 MHz, $CDCl_3$)

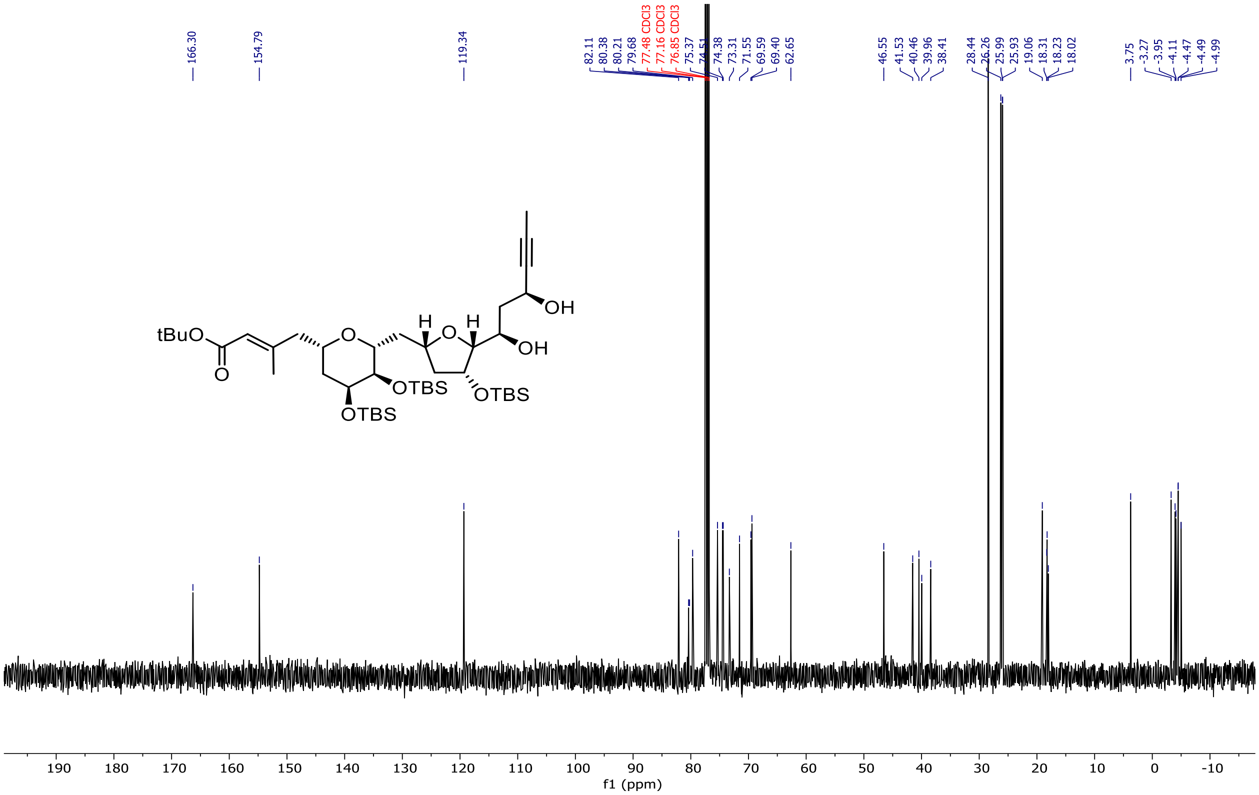

(*R*)-Mosher ester derived from compound 26: ^{1}H NMR (600 MHz, CDCl$_3$)

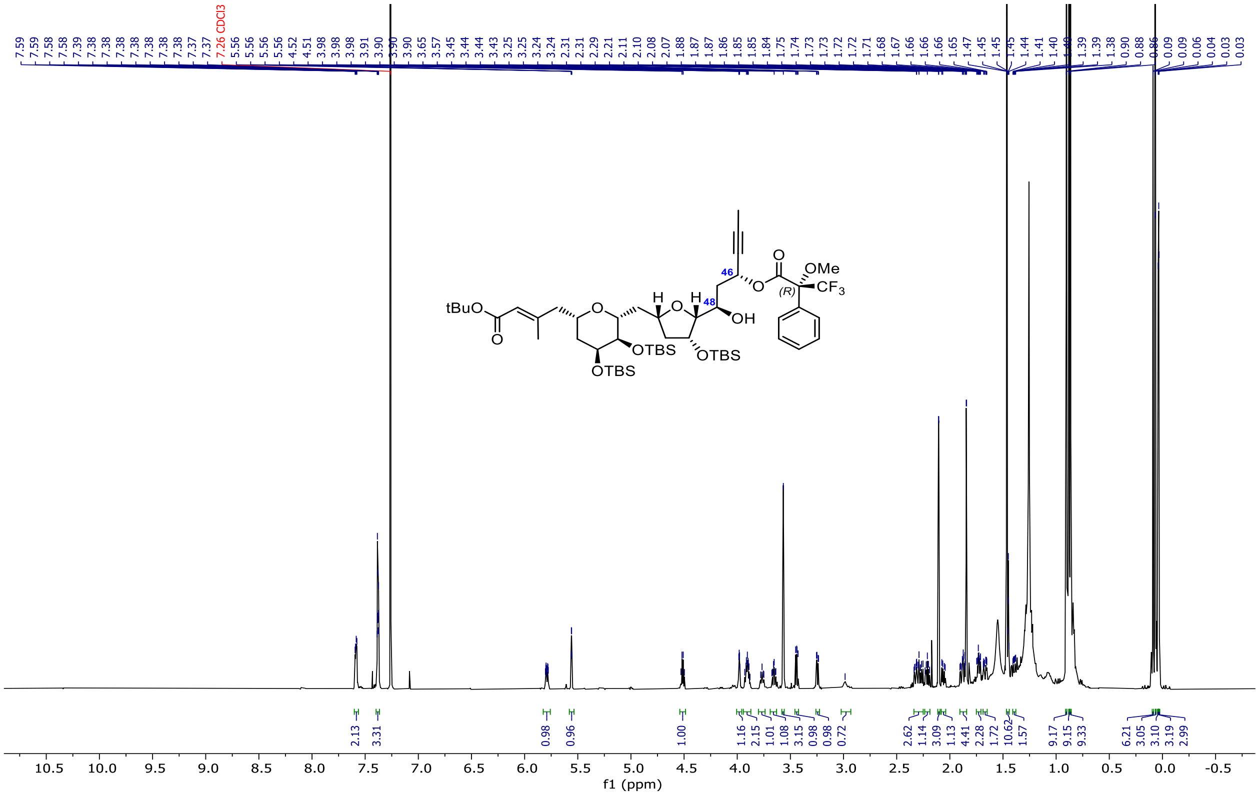

^{13}C NMR (151 MHz, CDCl$_3$)

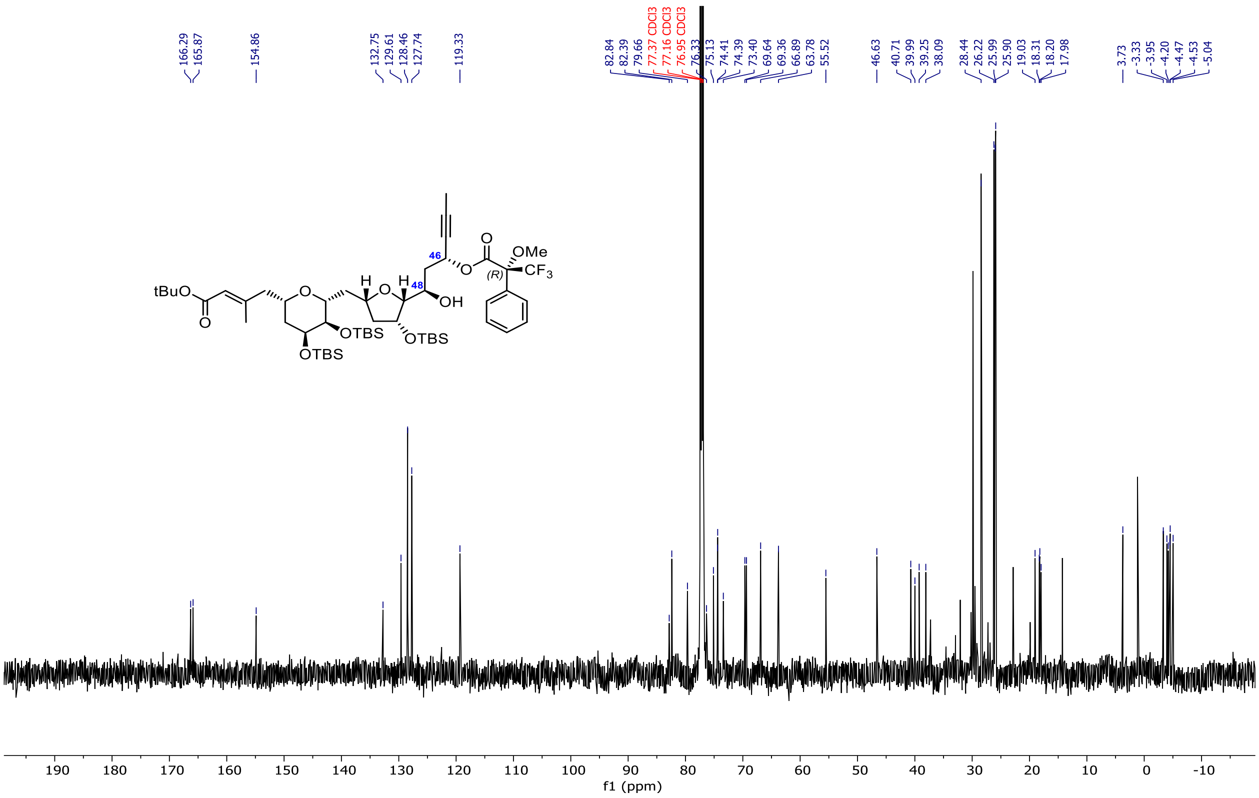

(*R*)-Mosher ester derived from compound 26: ^{1}H-^{1}H COSY ($CDCl_3$)

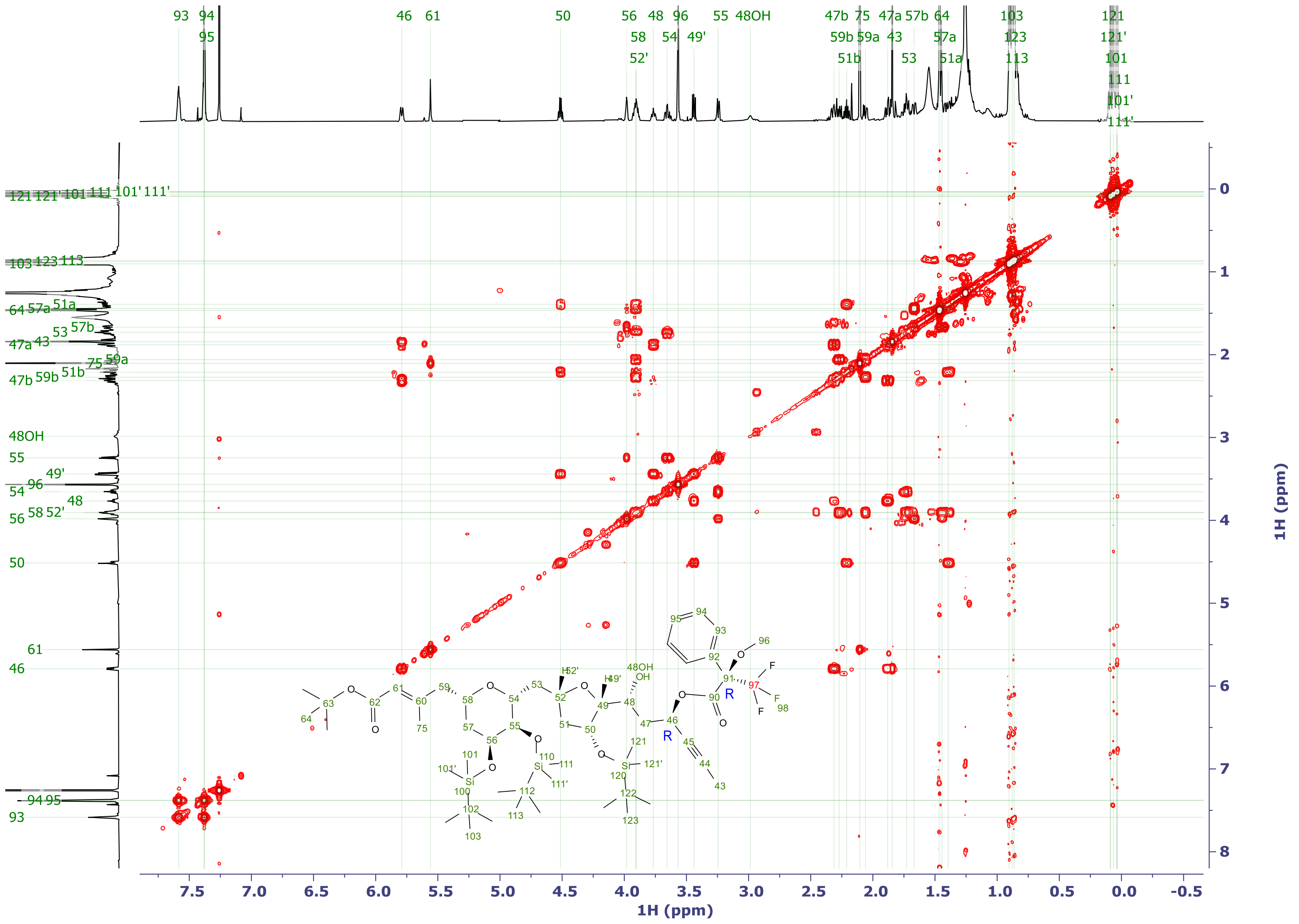

(*R*)-Mosher ester derived from compound 26: HSQC NMR ($CDCl_3$)

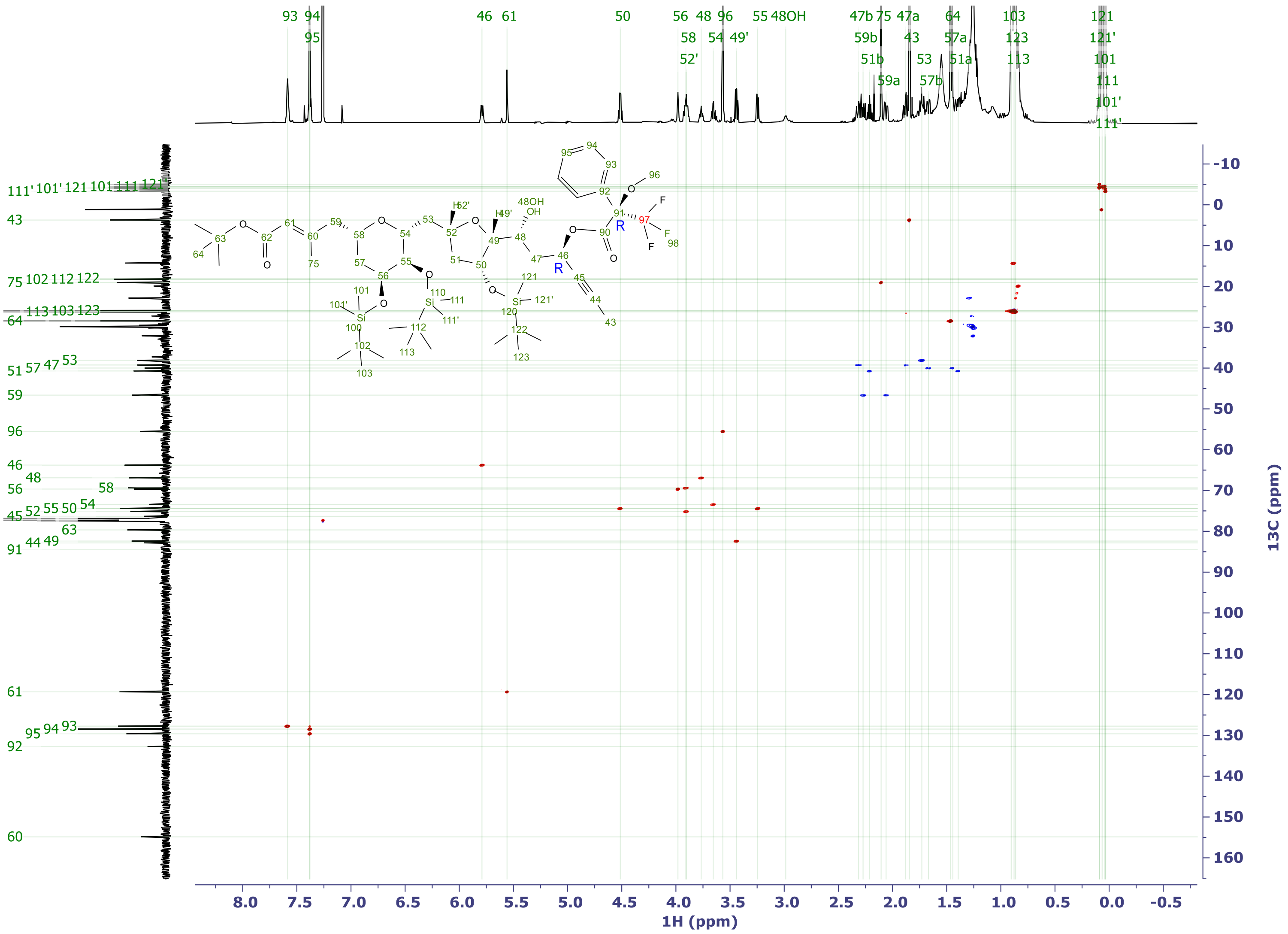

(*R*)-Mosher ester derived from compound 26: HMBC NMR ($CDCl_3$)

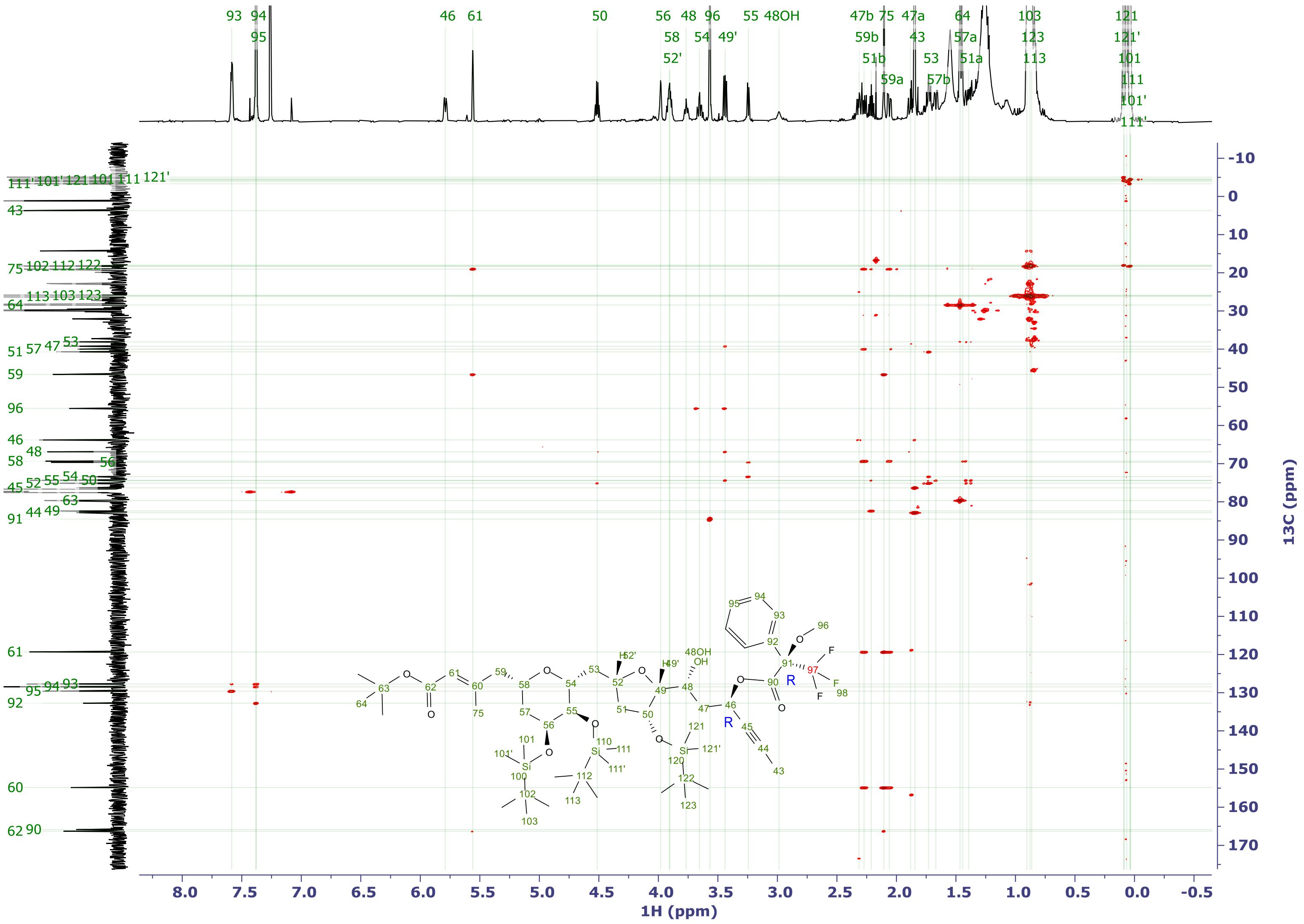

(*R*)-Mosher ester derived from compound 26: NOESY ($CDCl_3$)

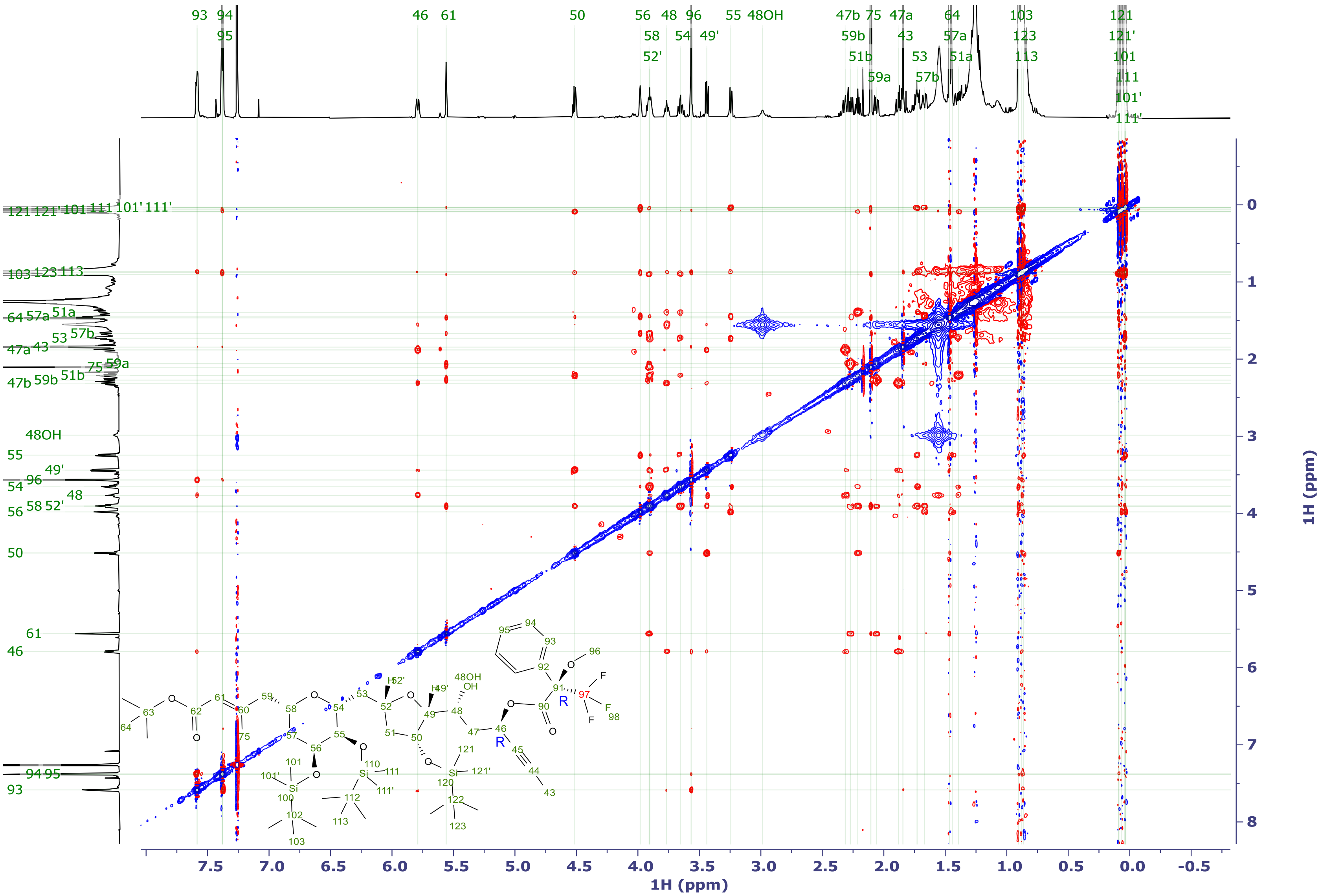

(*S*)-Mosher ester derived from compound 26: ^{1}H NMR (600 MHz, $CDCl_3$)

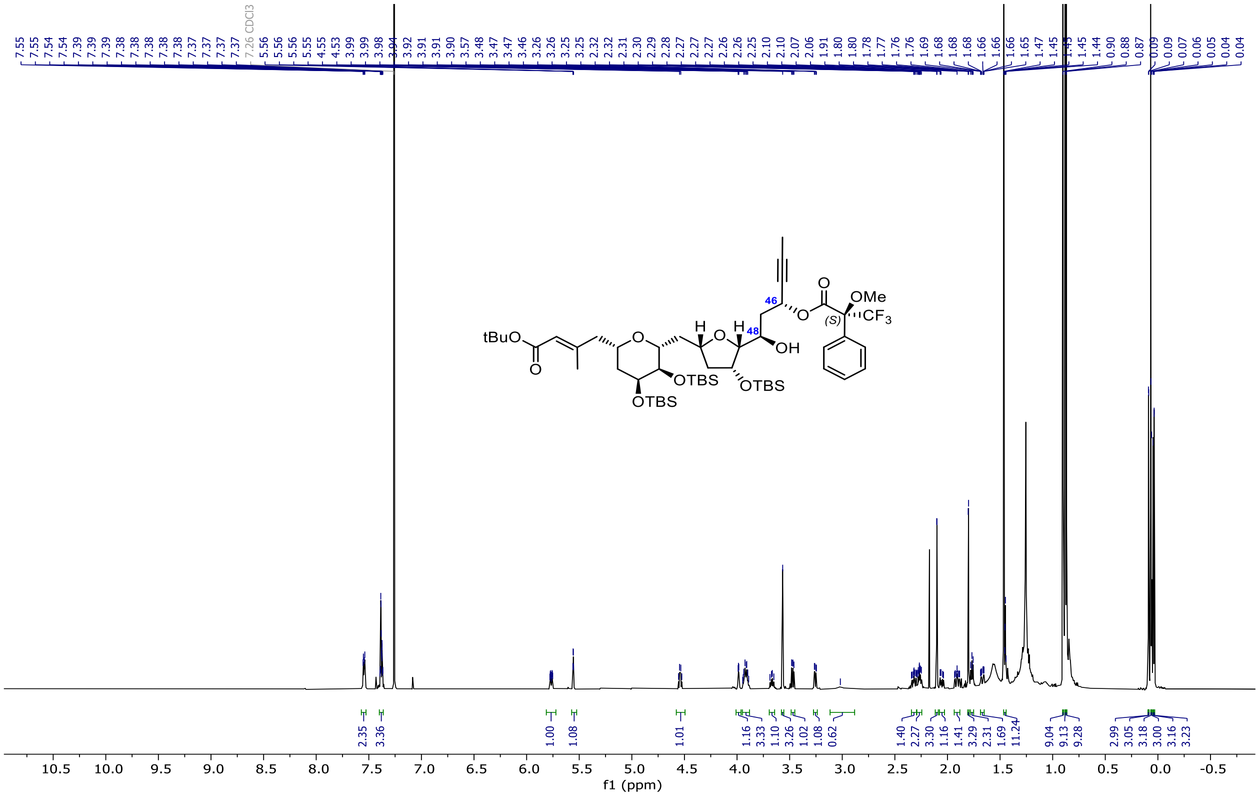

^{13}C NMR (151 MHz, $CDCl_3$)

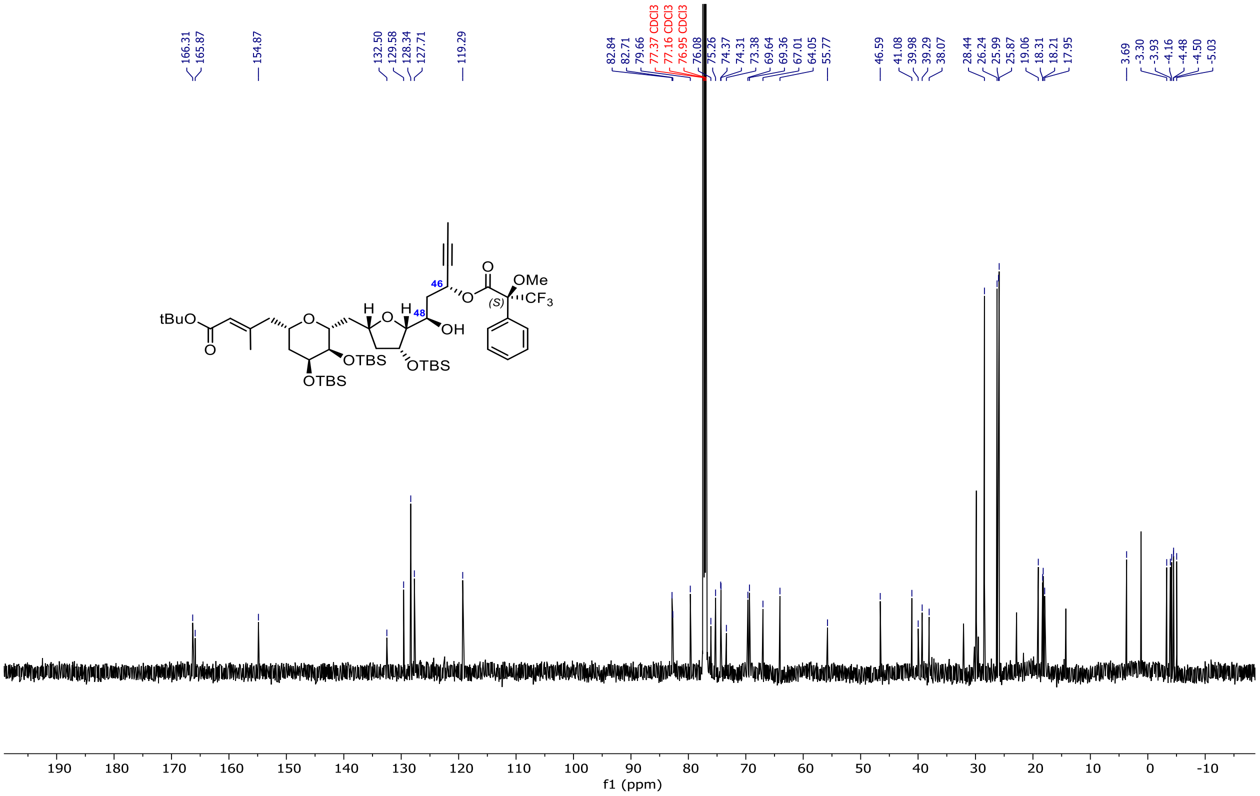

(*S*)-Mosher ester derived from compound 26: ^{1}H-^{1}H COSY ($CDCl_3$)

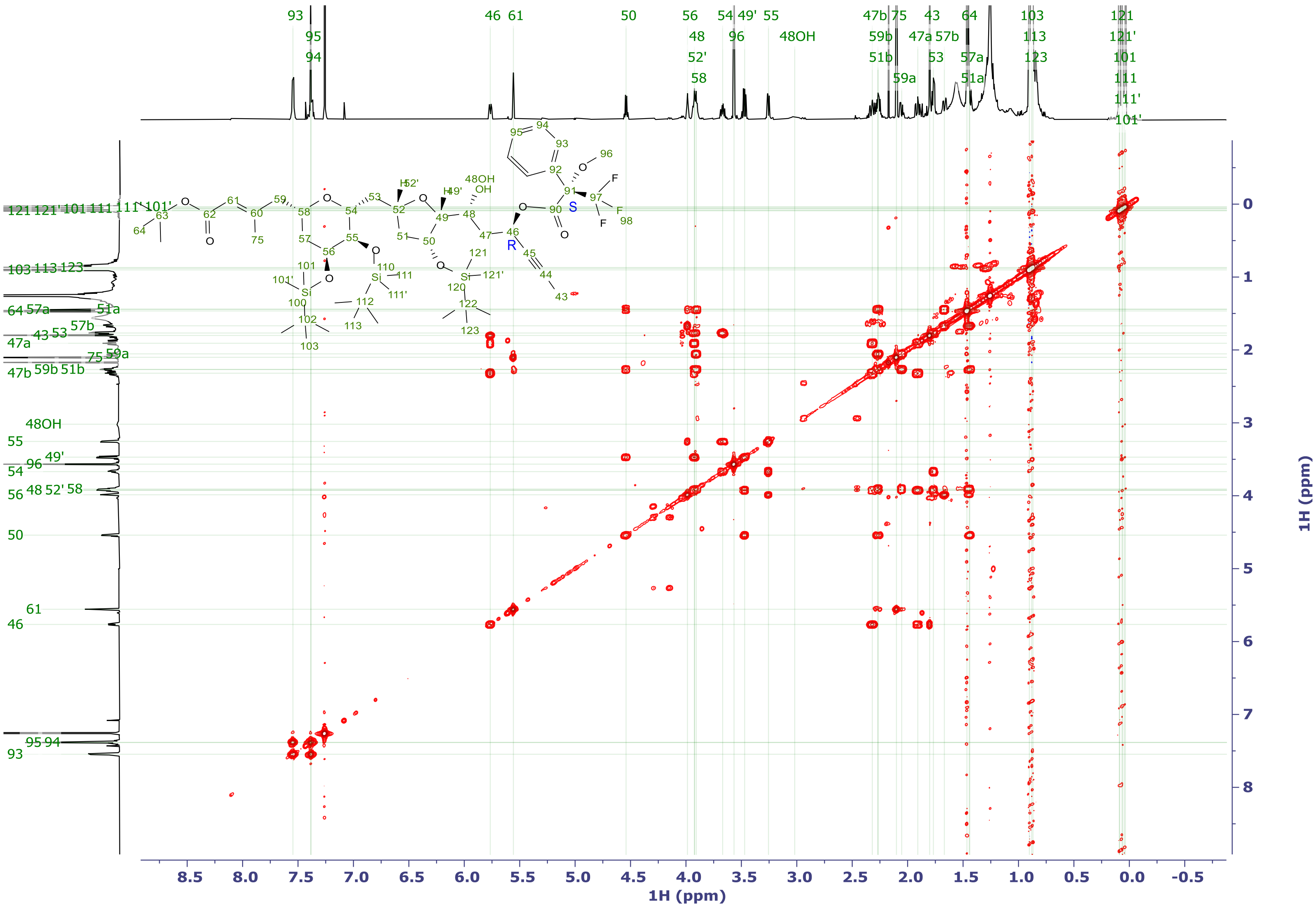

(*S*)-Mosher ester derived from compound 26: HSQC NMR ($CDCl_3$)

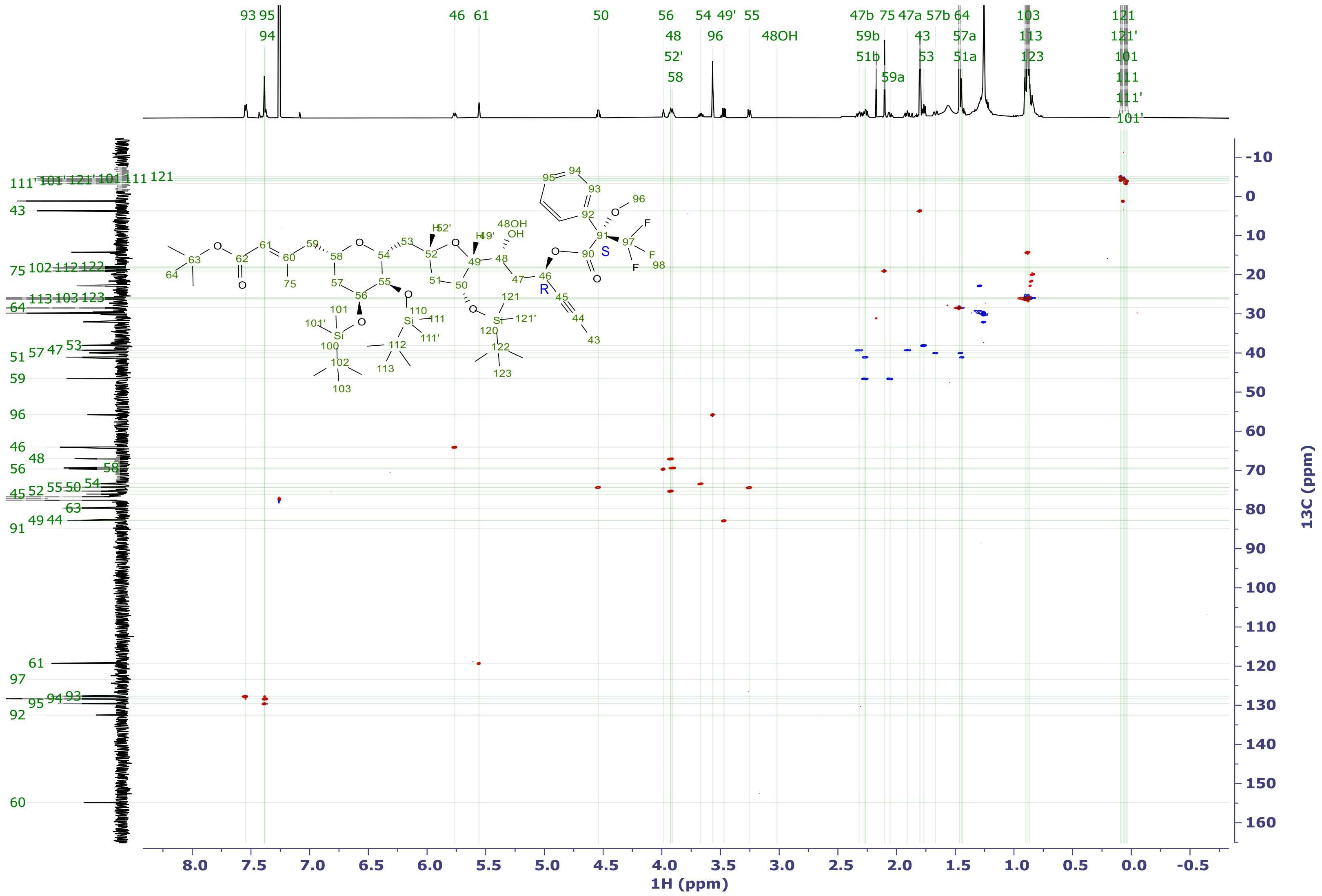

(*S*)-Mosher ester derived from compound 26: HMBC NMR ($CDCl_3$)

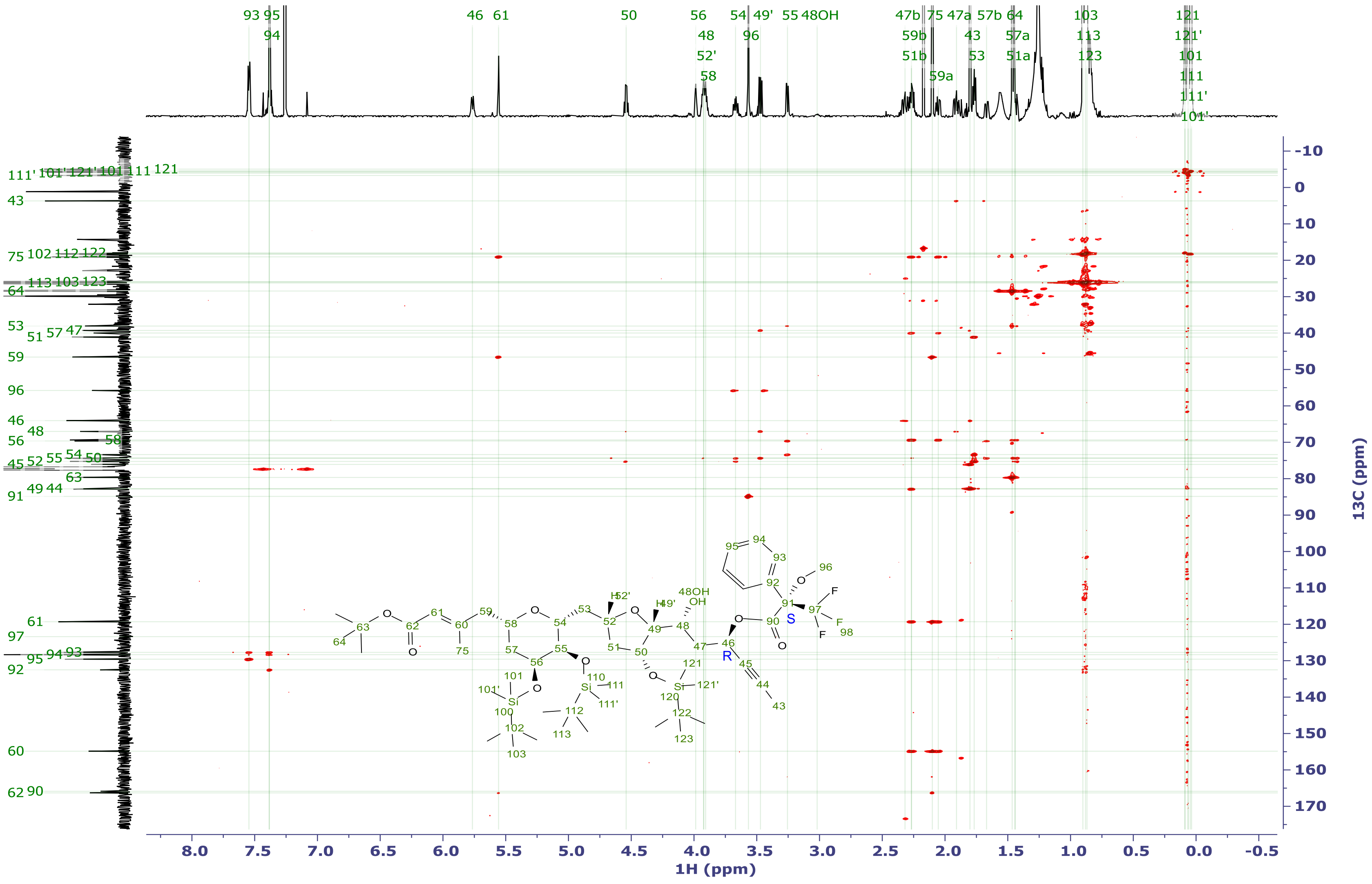

(*S*)-Mosher ester derived from compound 26: NOESY ($CDCl_3$)

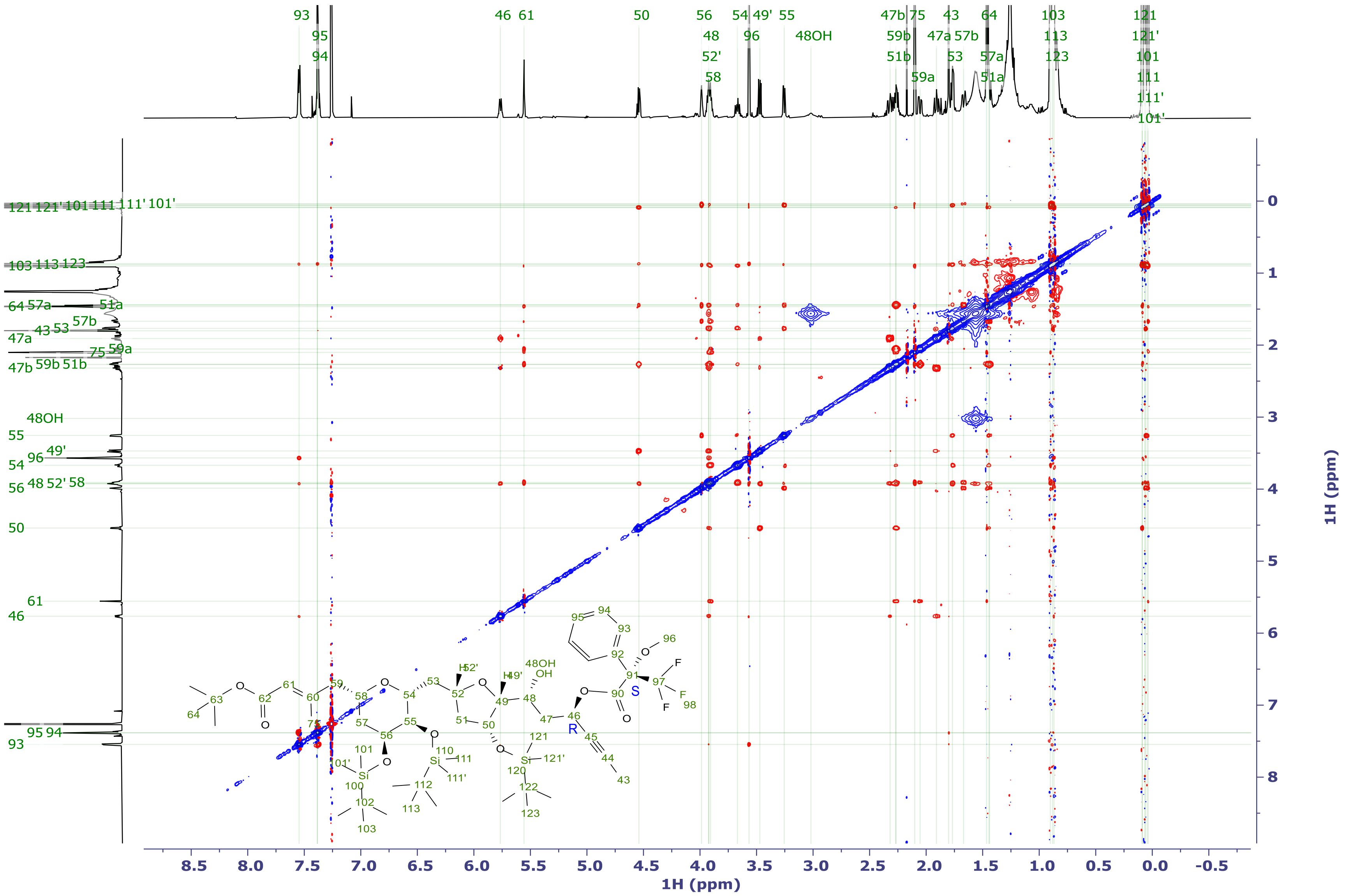

Compound S11: ^{1}H NMR (600 MHz, CD_2Cl_2)

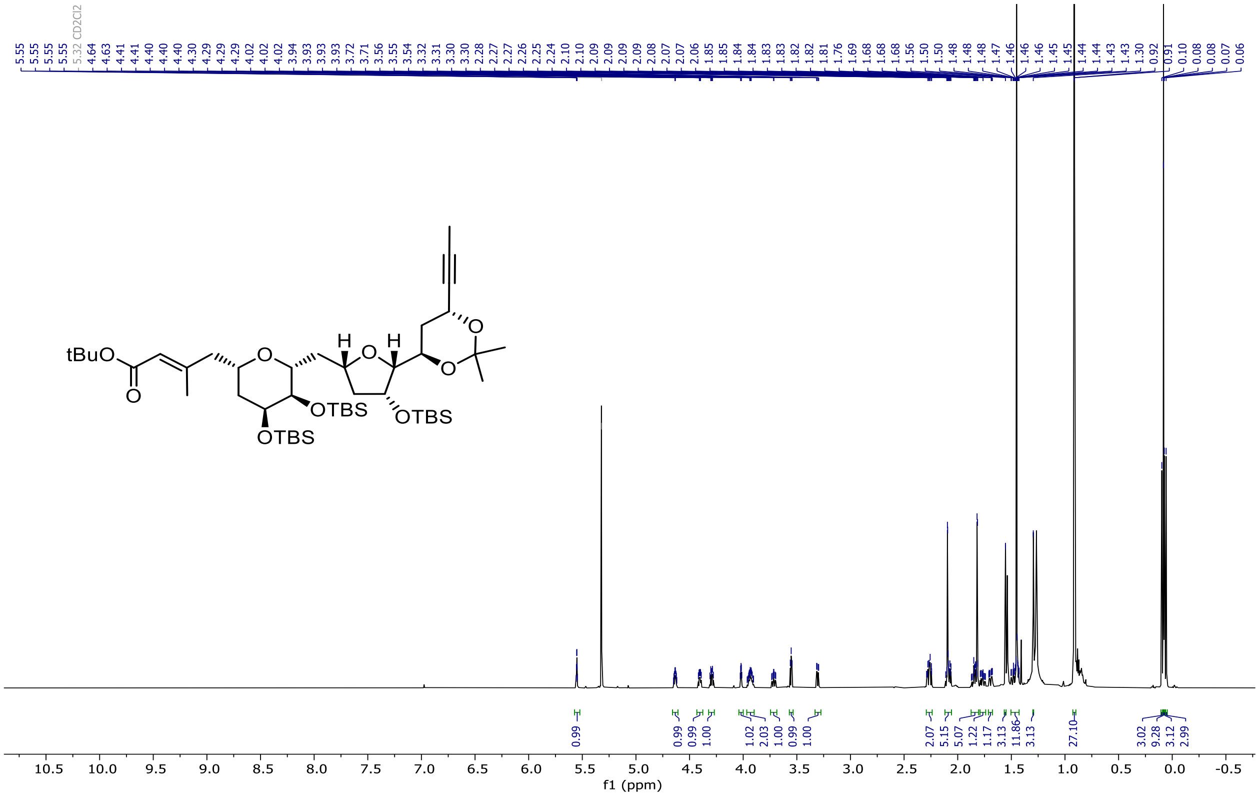

^{13}C NMR (151 MHz, CD_2Cl_2)

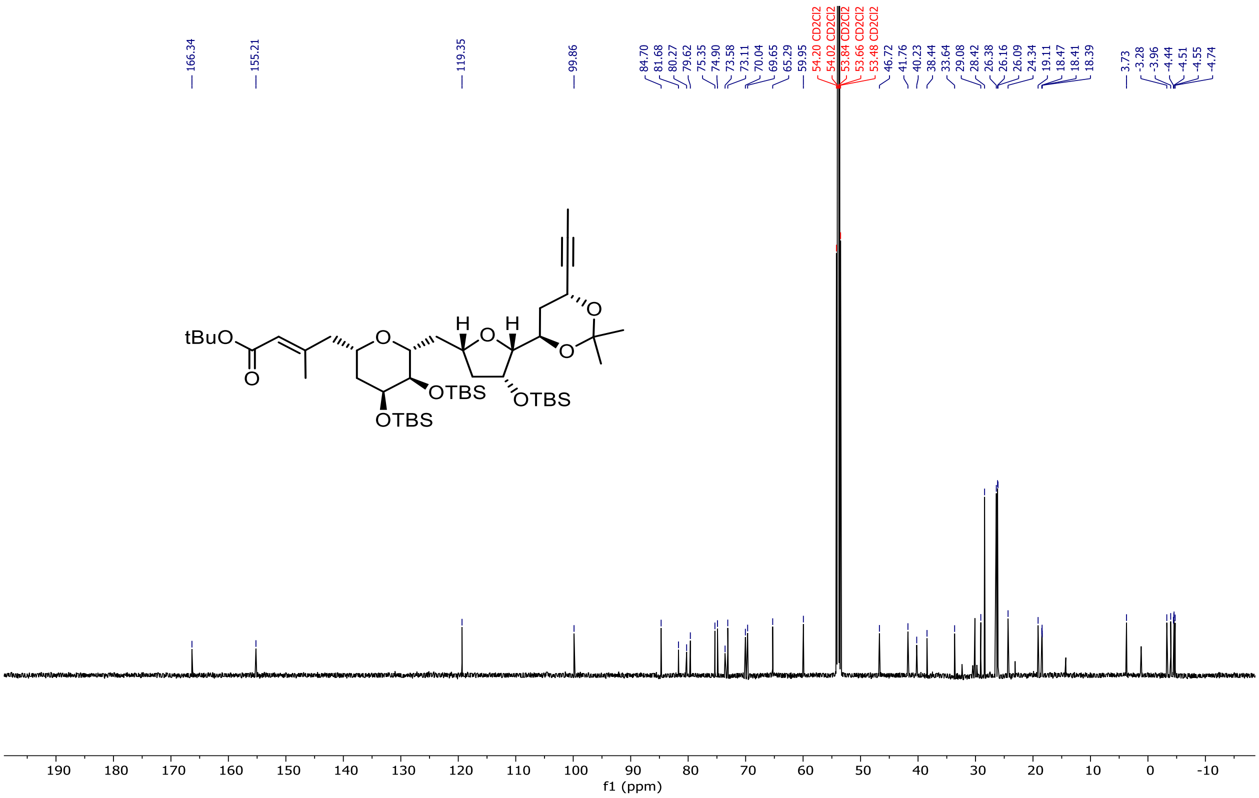

Compound S11: ^{1}H-^{1}H COSY (CD_2Cl_2)

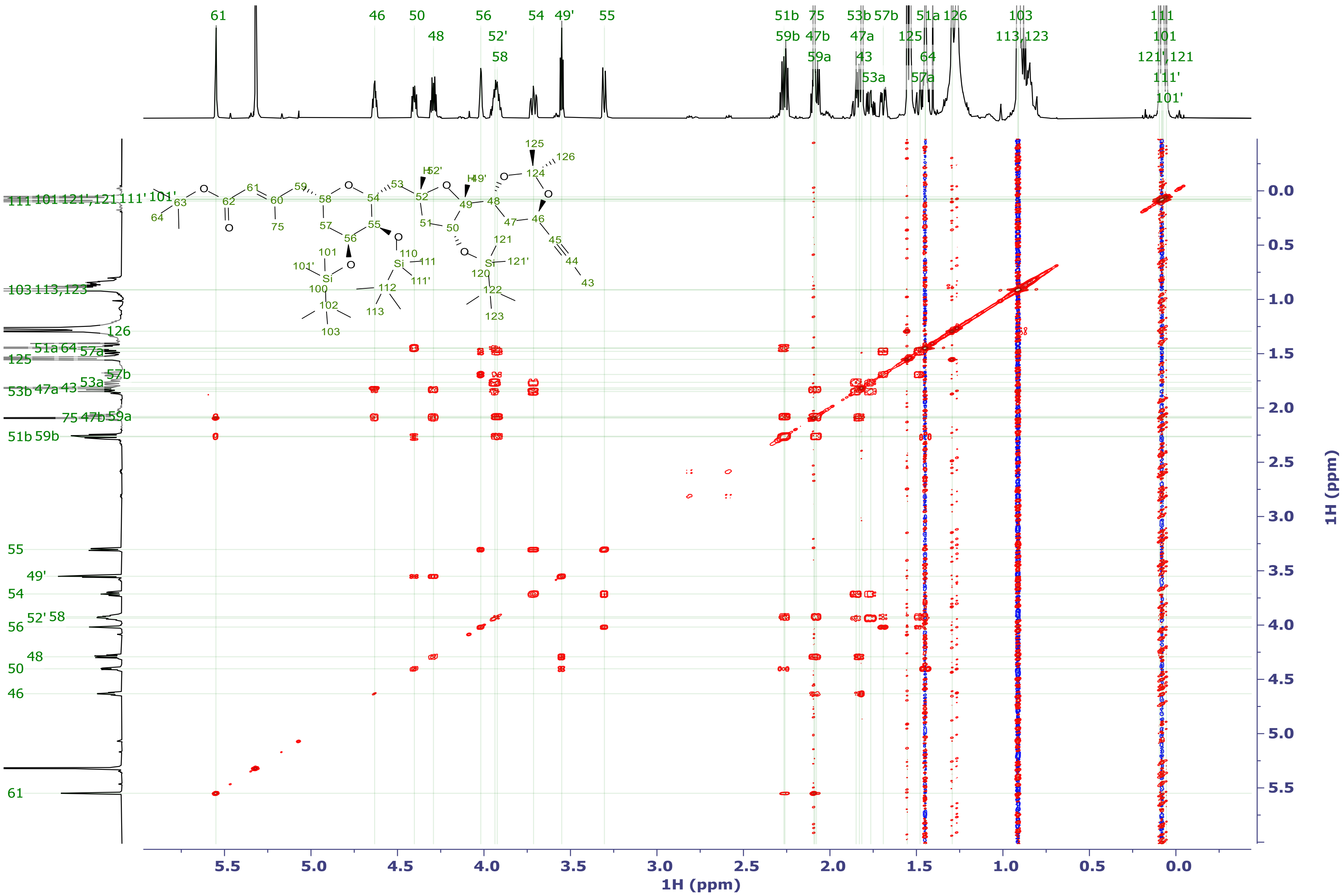

Compound S11: HSQC NMR (CD_2Cl_2)

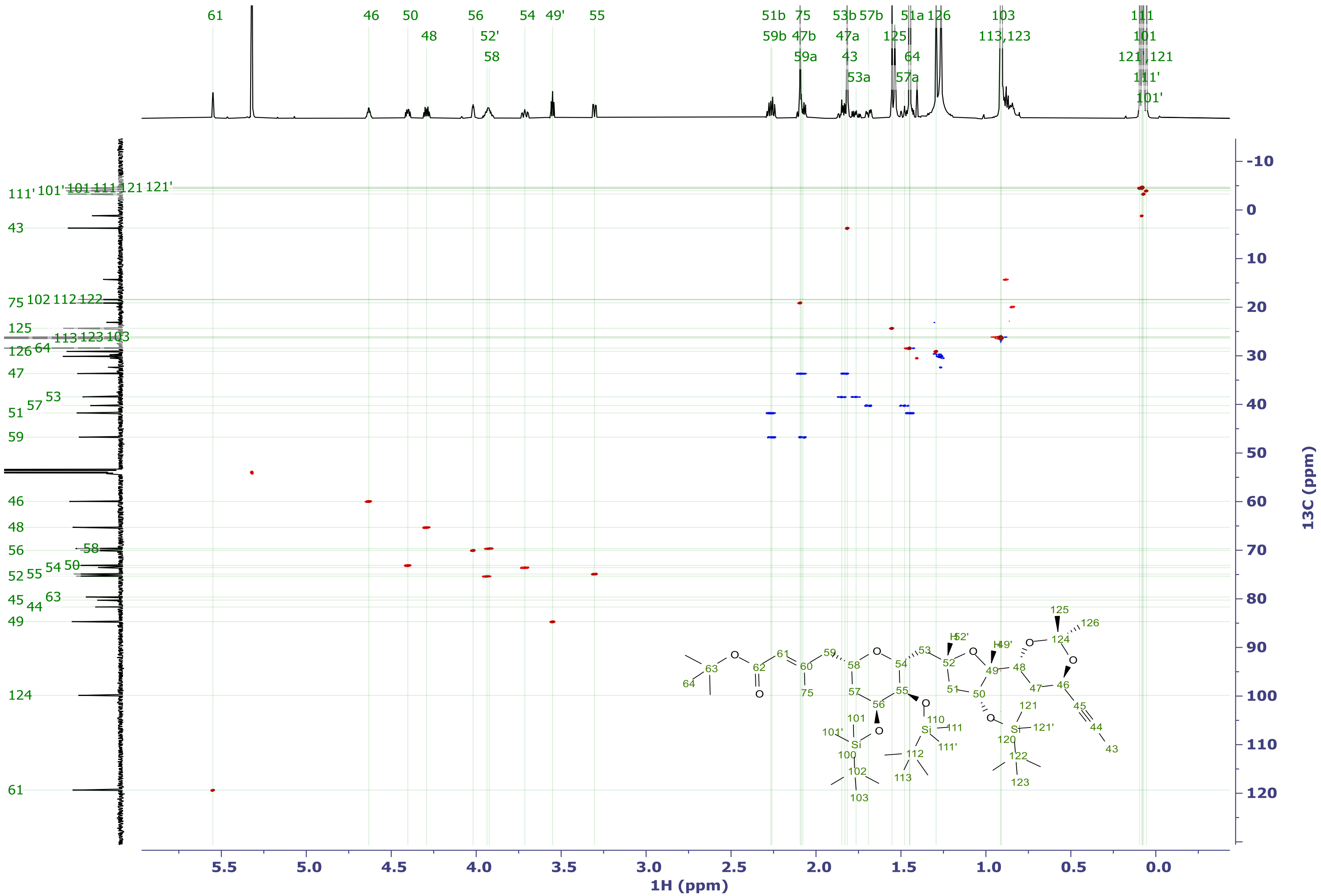

Compound S11: HMBC NMR (CD_2Cl_2)

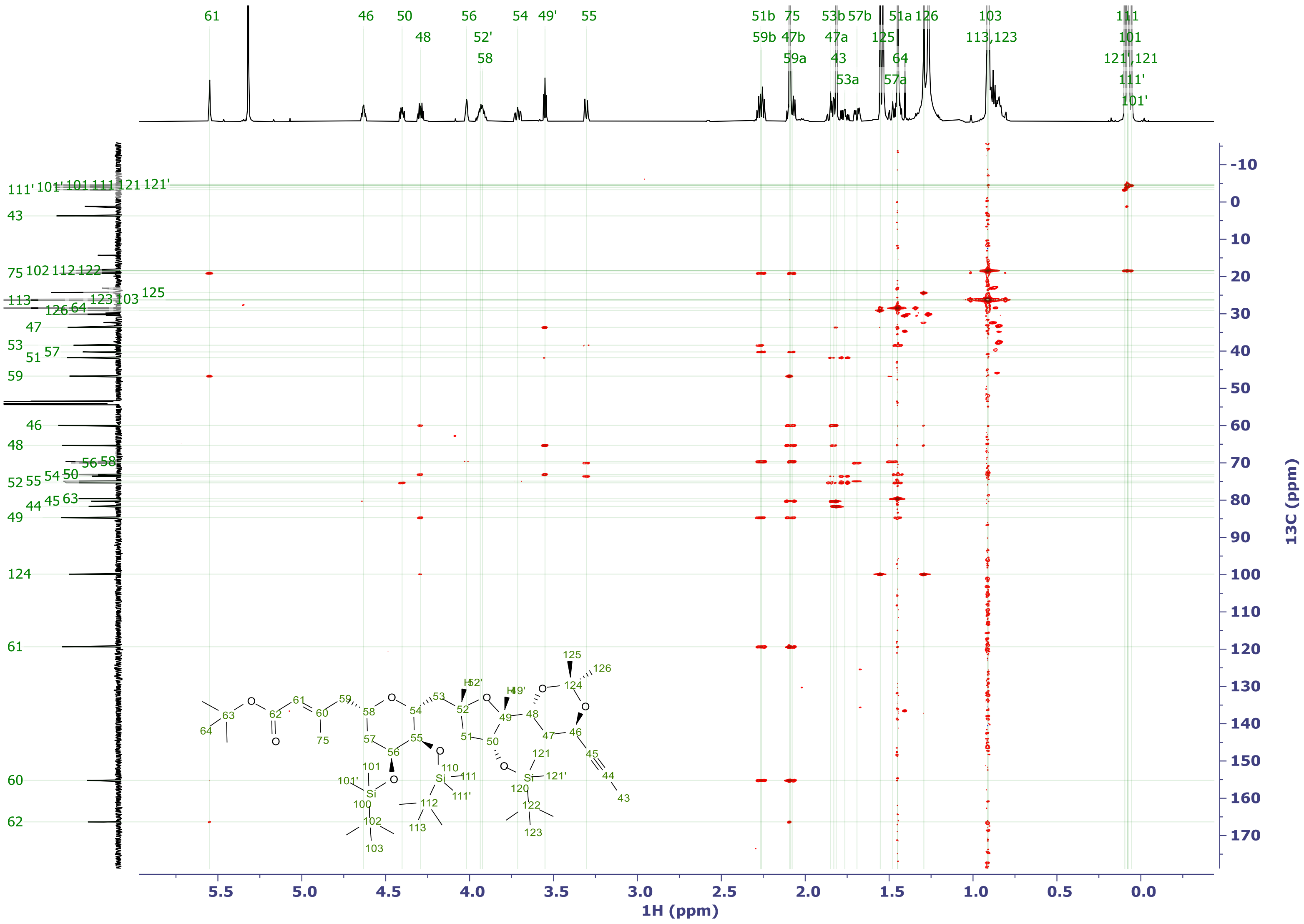

Compound S11: NOESY (CD_2Cl_2)

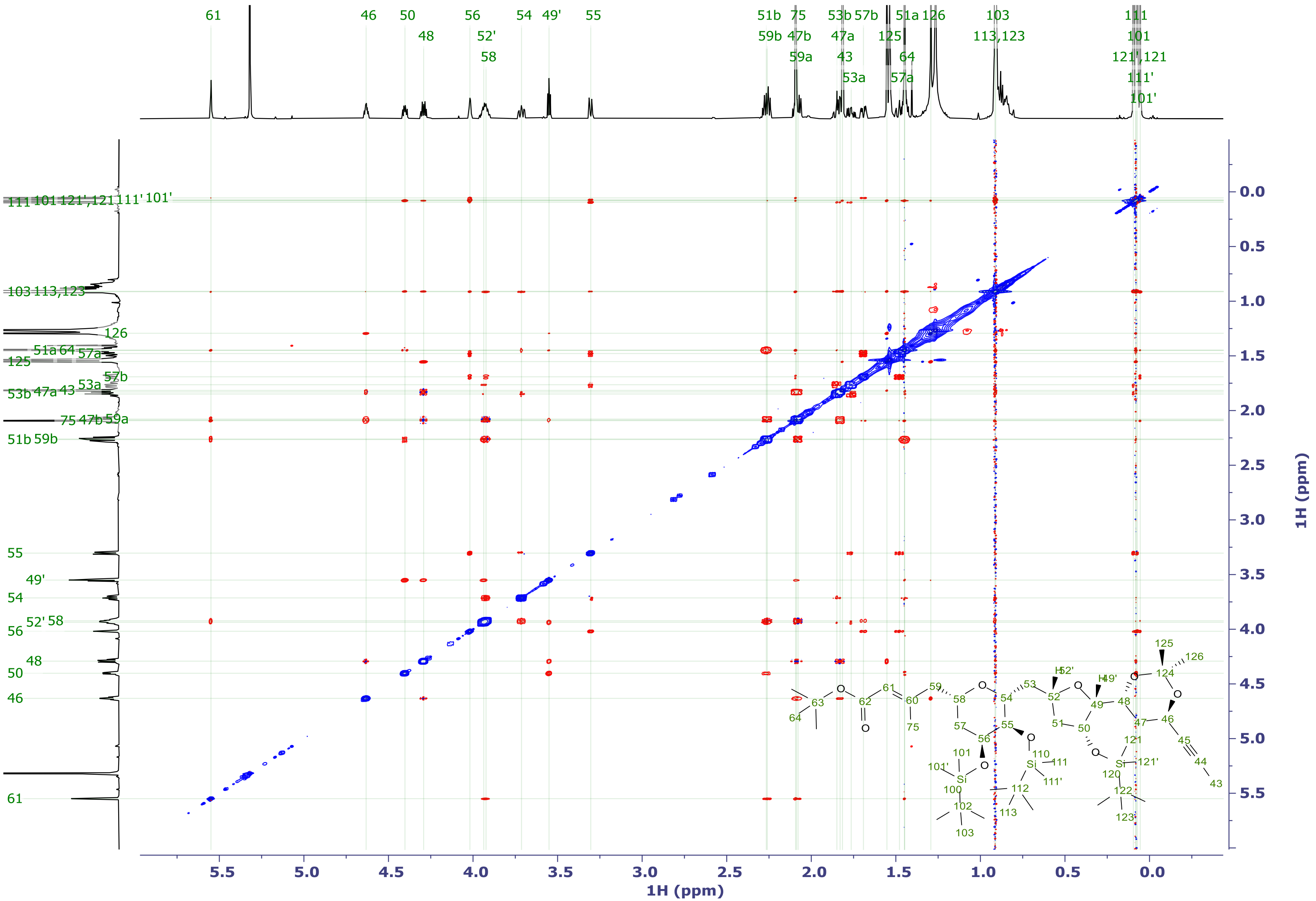

Compound S12: ^{1}H NMR (600 MHz, CD_2Cl_2)

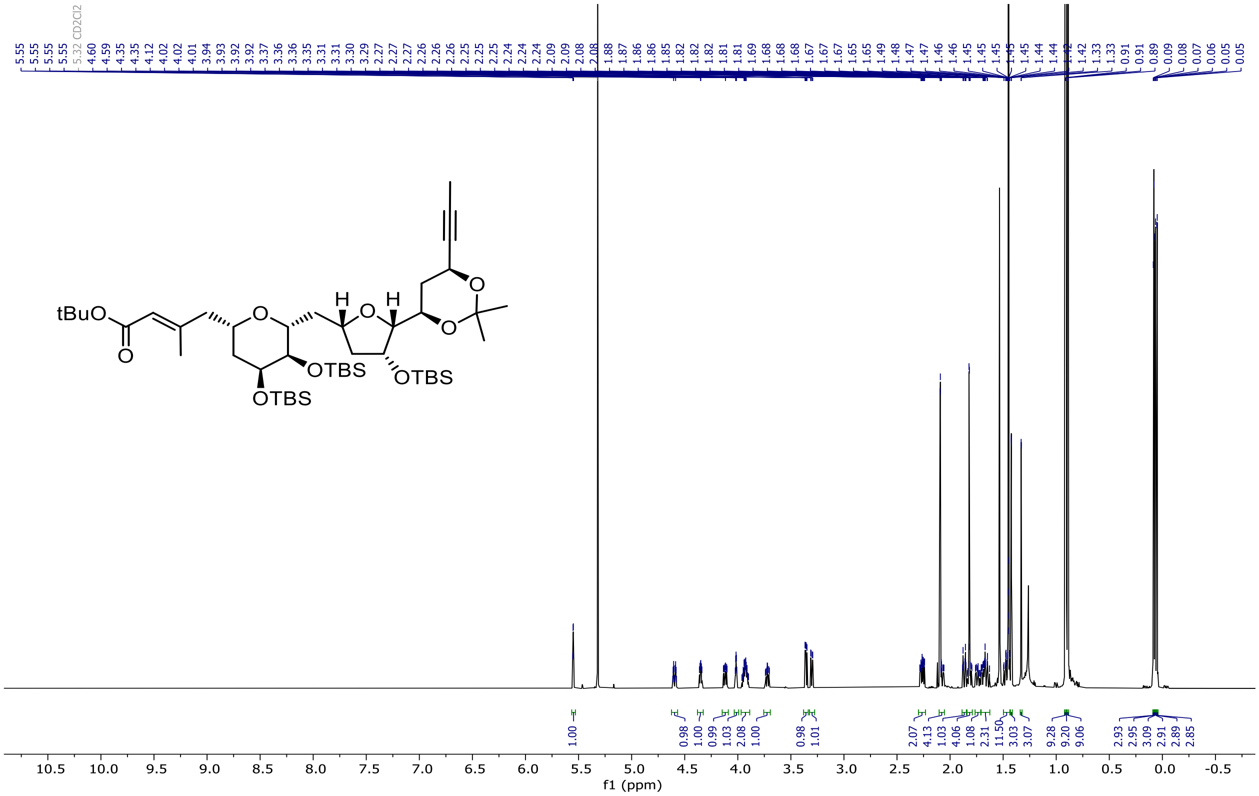

^{13}C NMR (151 MHz, CD_2Cl_2)

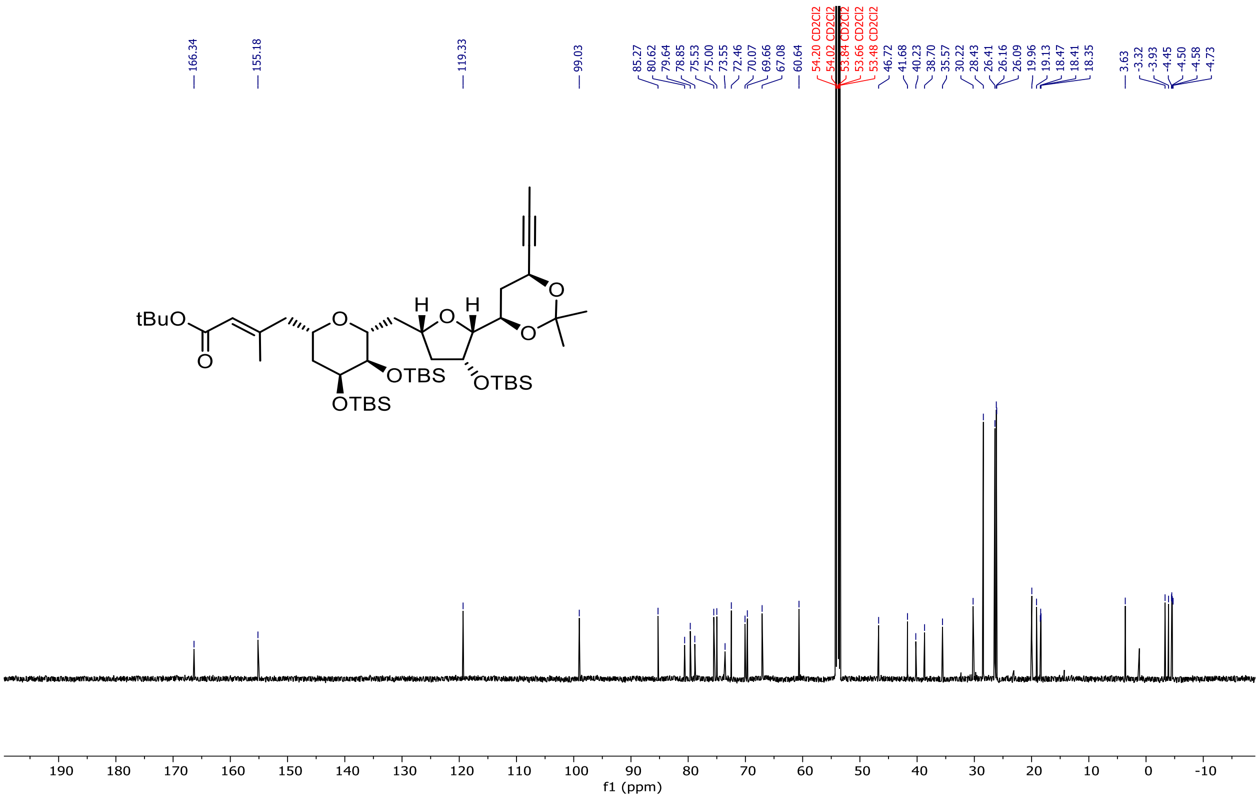

Compound S12: ^{1}H-^{1}H COSY (CD_2Cl_2)

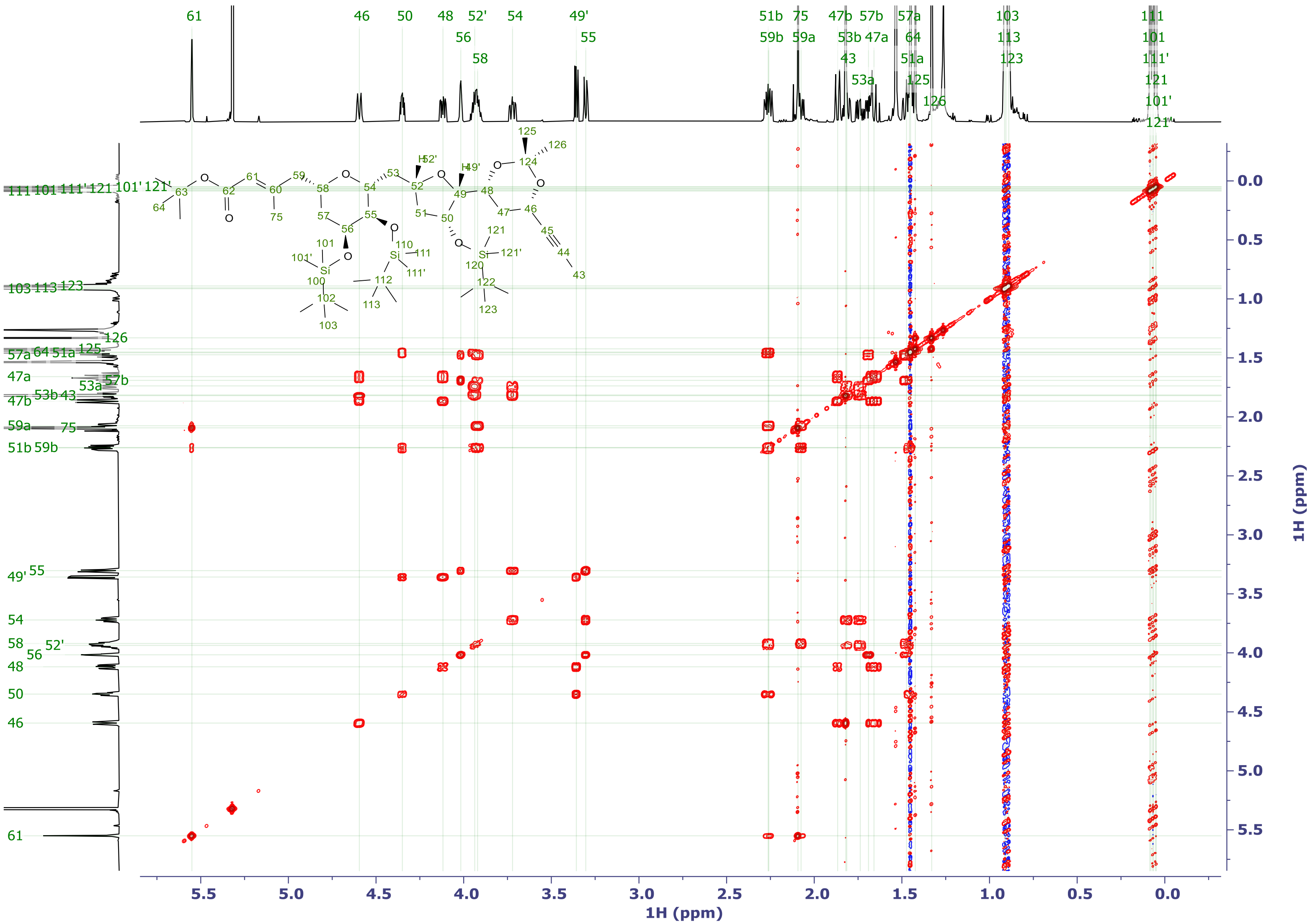

Compound S12: HSQC NMR (CD_2Cl_2)

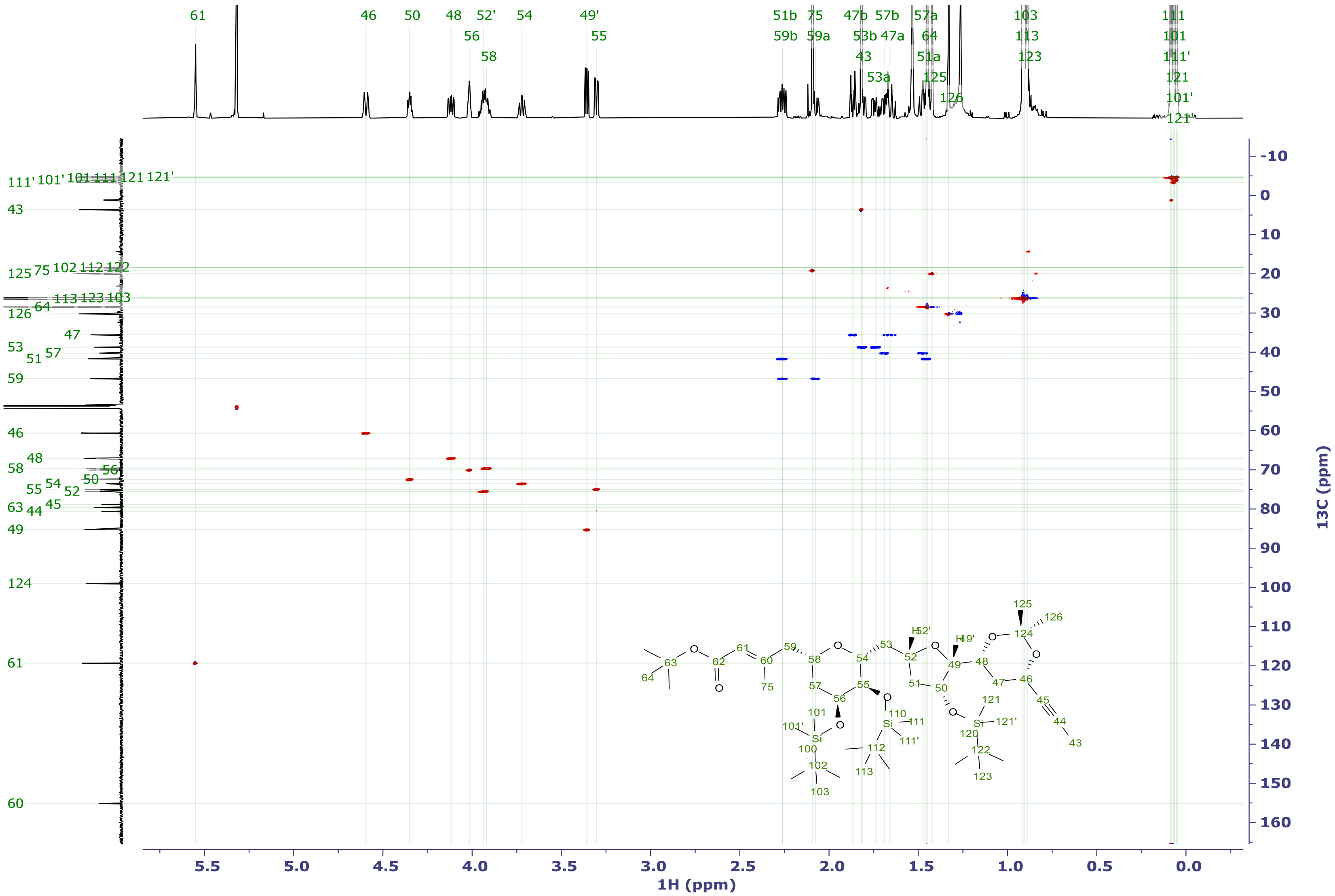

Compound S12: HMBC NMR (CD_2Cl_2)

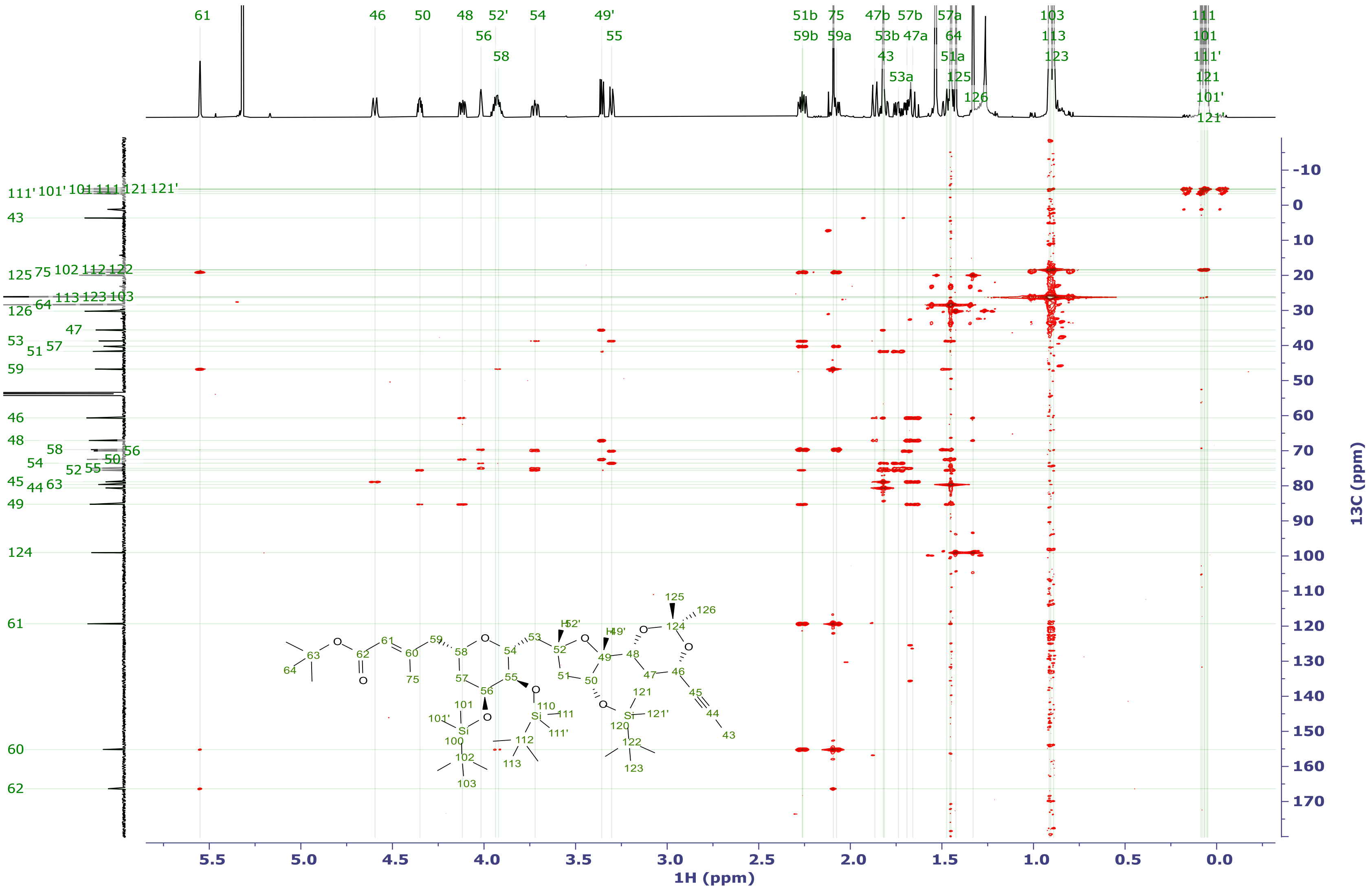

Compound S12: NOESY (CD_2Cl_2)

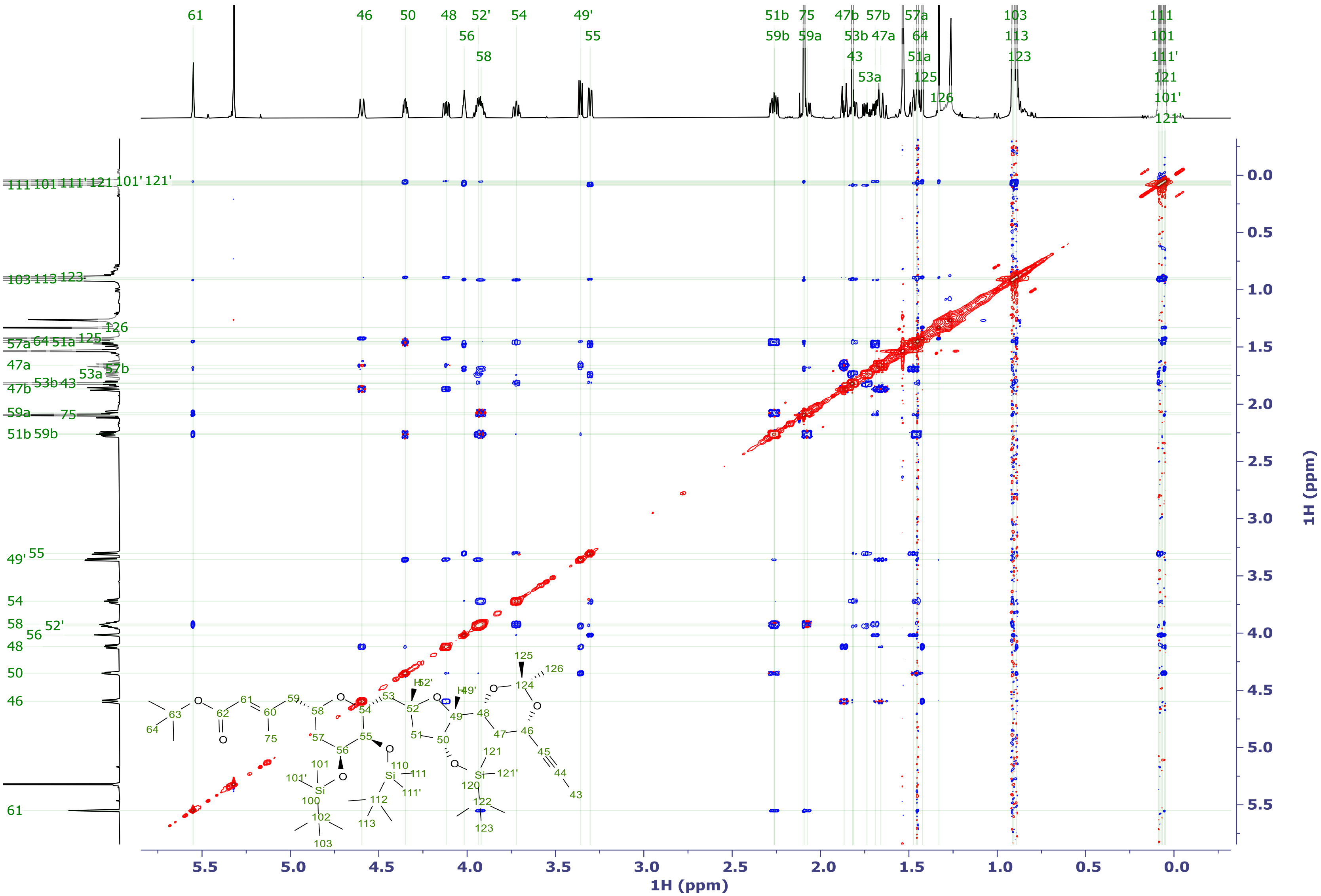

Compound S13: ^{1}H NMR (400 MHz, $CDCl_3$)

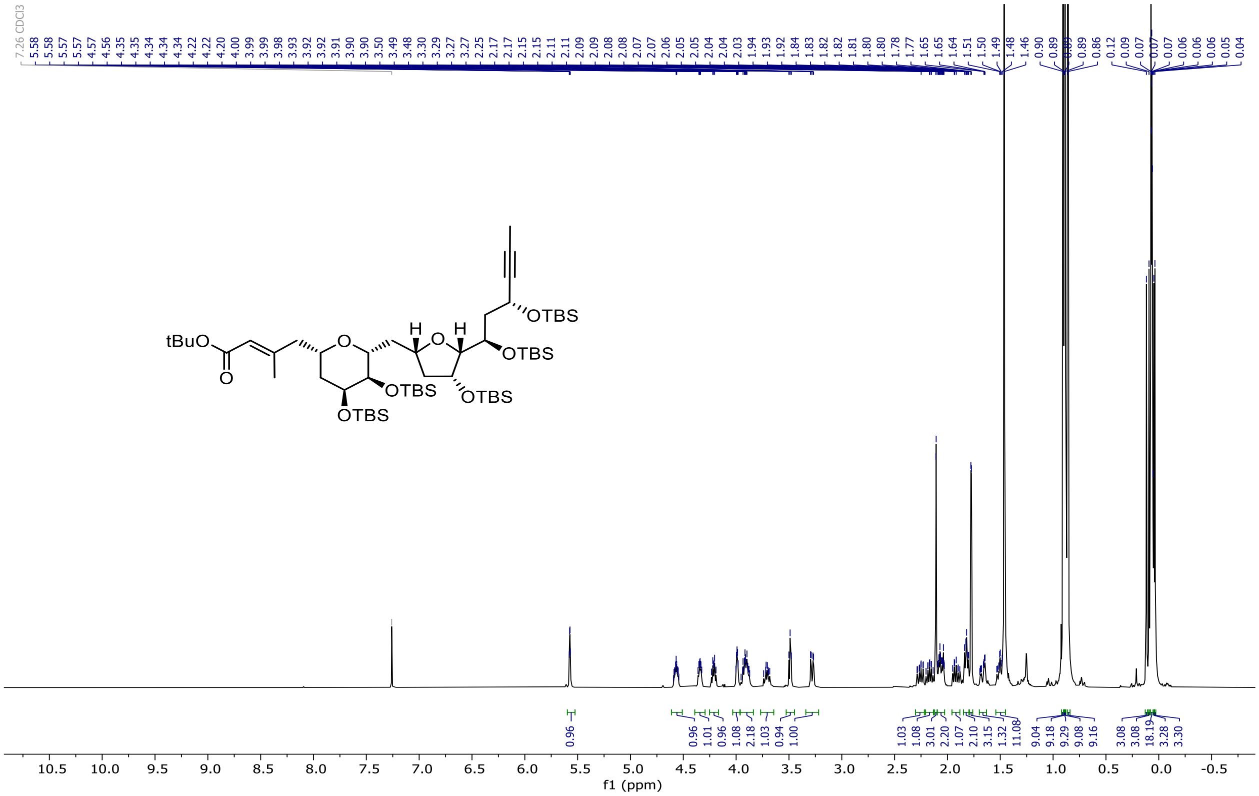

^{13}C NMR (101 MHz, $CDCl_3$)

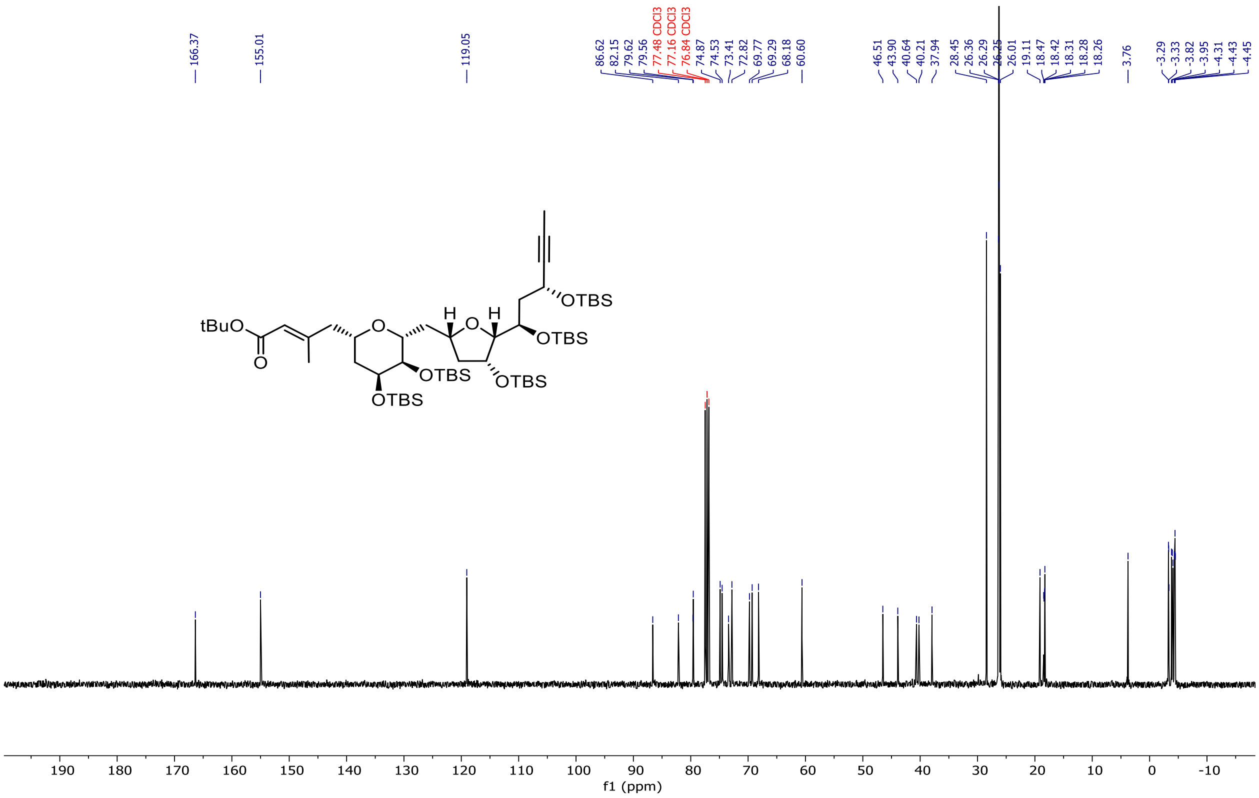

Compound 27: ^{1}H NMR (400 MHz, $CDCl_3$)

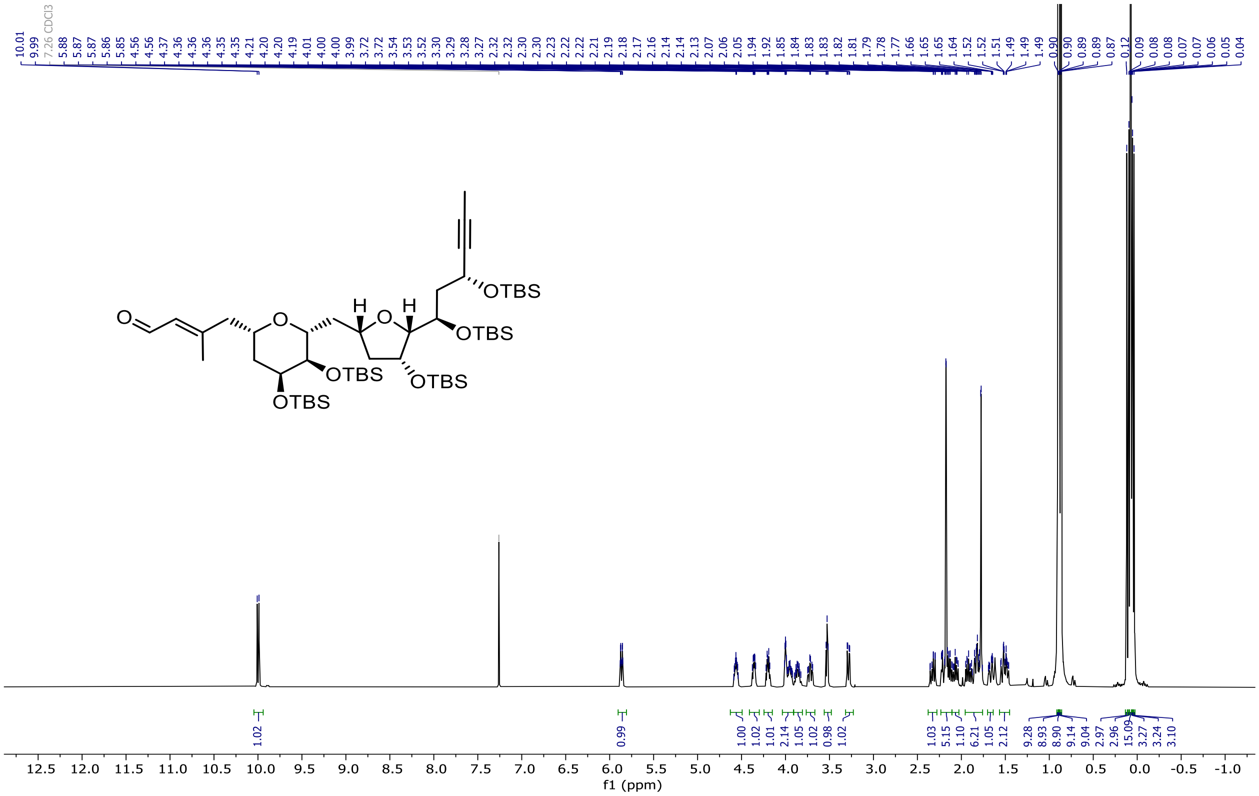

^{13}C NMR (101 MHz, $CDCl_3$)

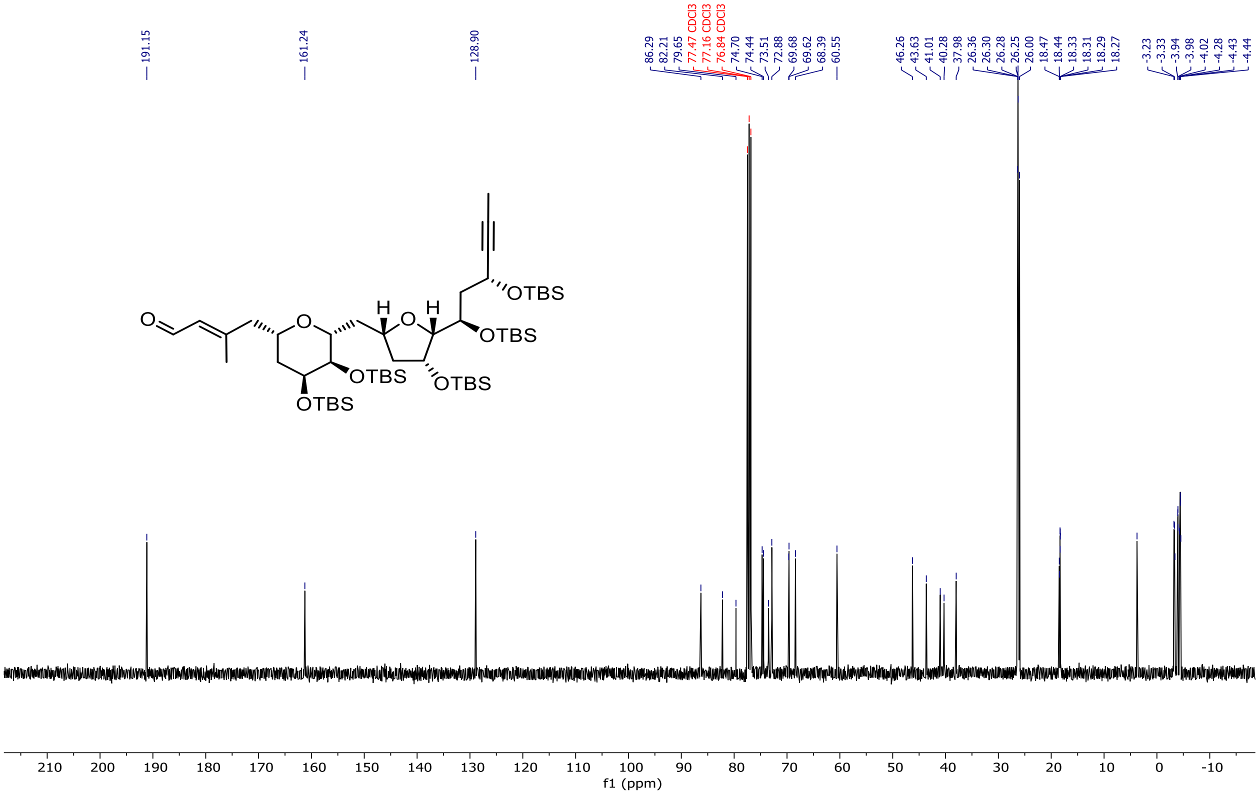

Compound 28: ^{1}H NMR (400 MHz, $CDCl_3$)

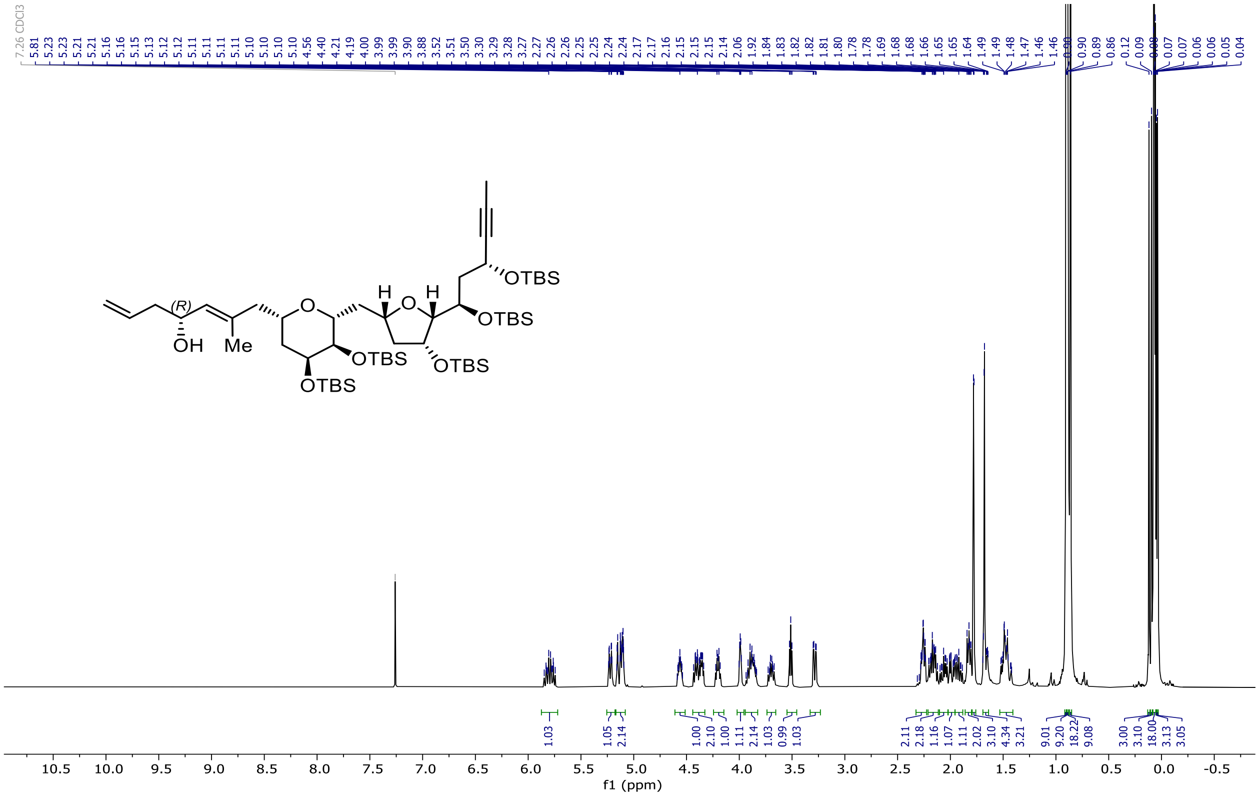

^{13}C NMR (101 MHz, $CDCl_3$)

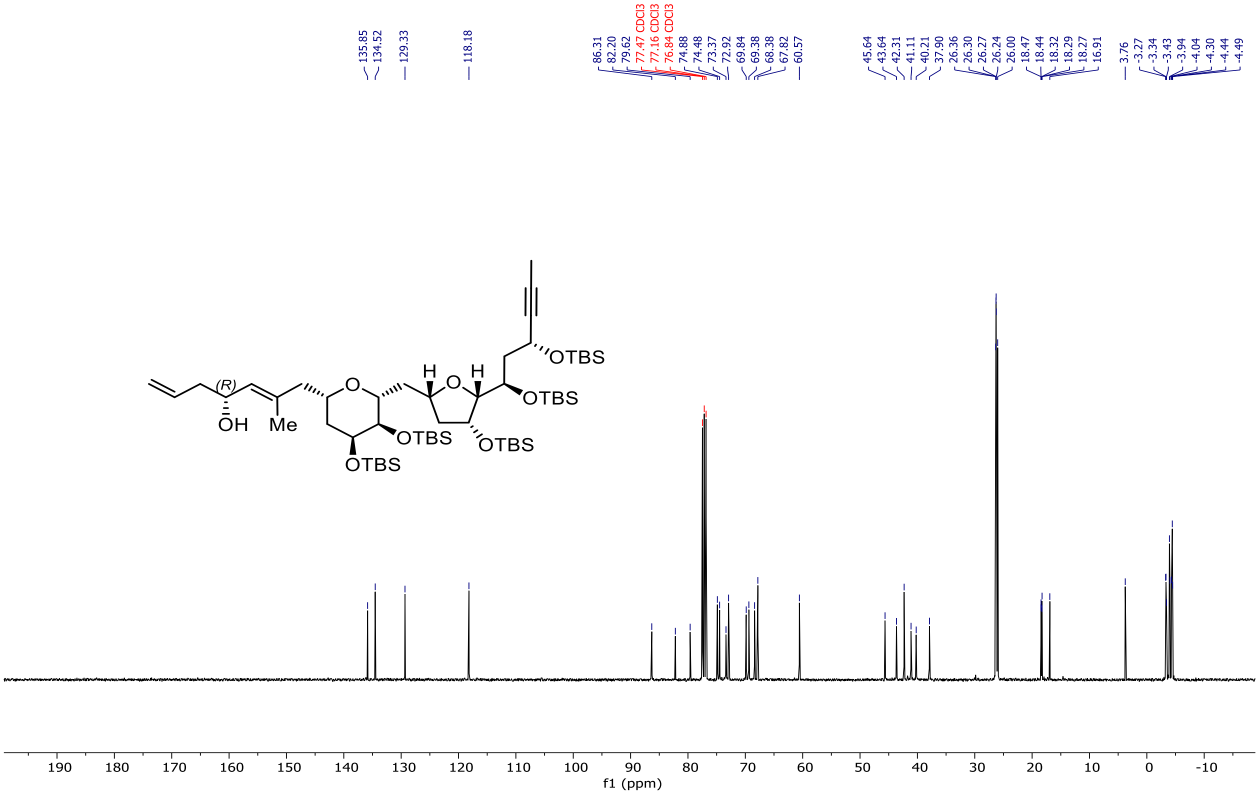

(*S*)-Mosher ester derived from compound 28: ^{1}H NMR (600 MHz, $CDCl_3$)

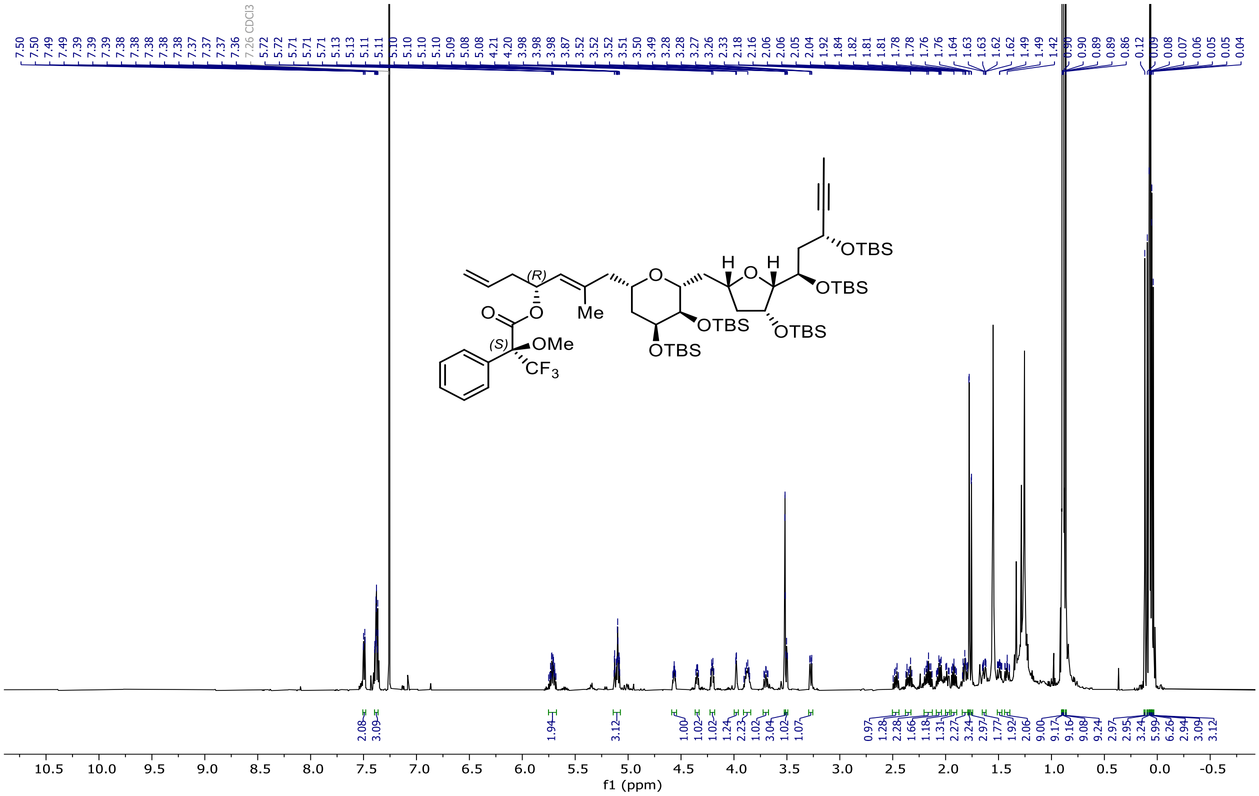

^{13}C NMR (151 MHz, $CDCl_3$)

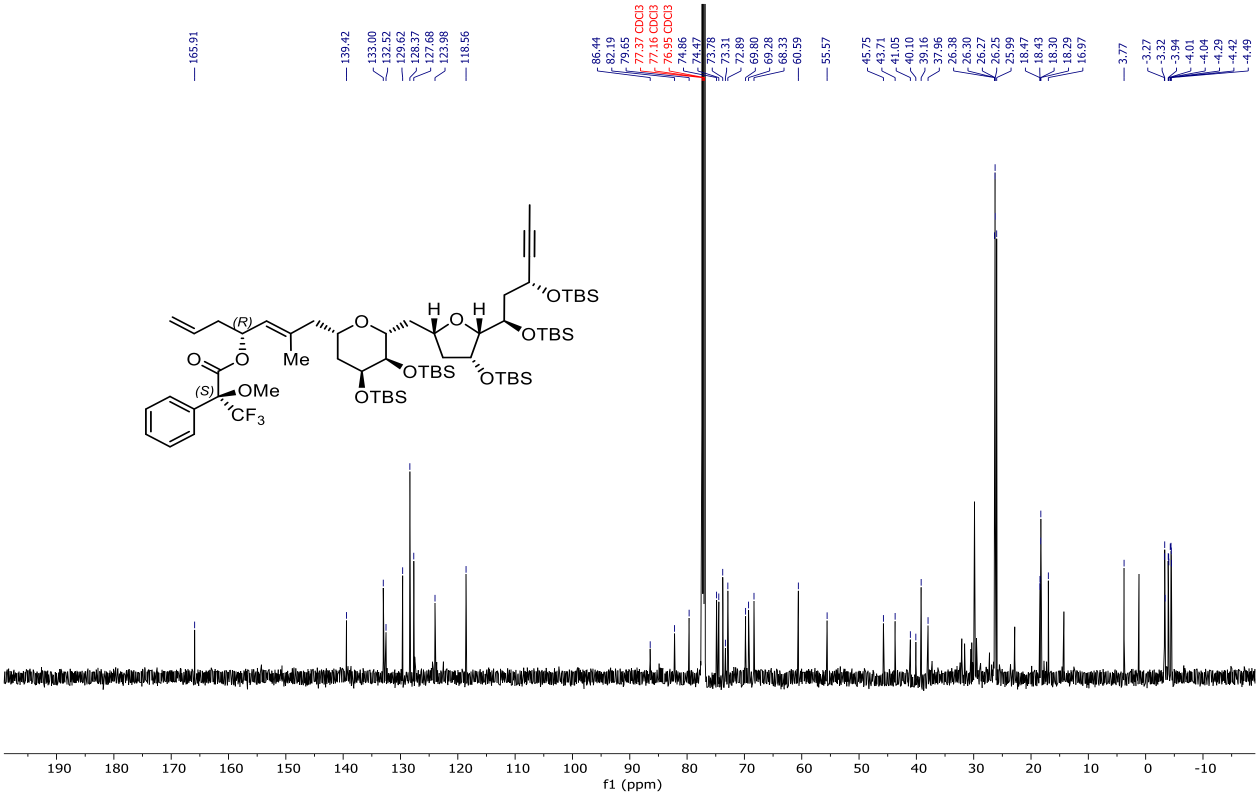

(*S*)-Mosher ester derived from compound 28: ^{1}H-^{1}H COSY ($CDCl_3$)

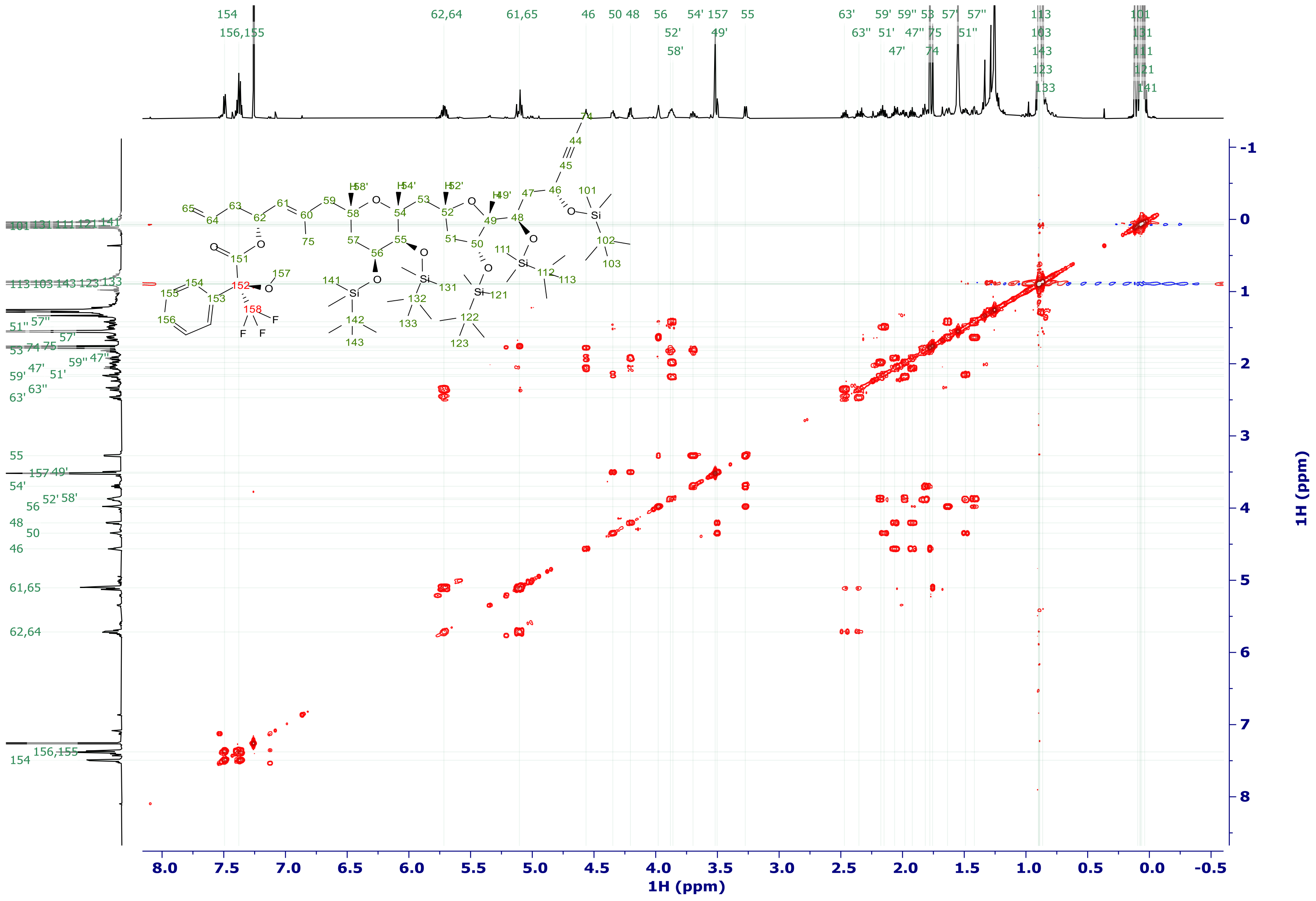

(*S*)-Mosher ester derived from compound 28: HSQC NMR ($CDCl_3$)

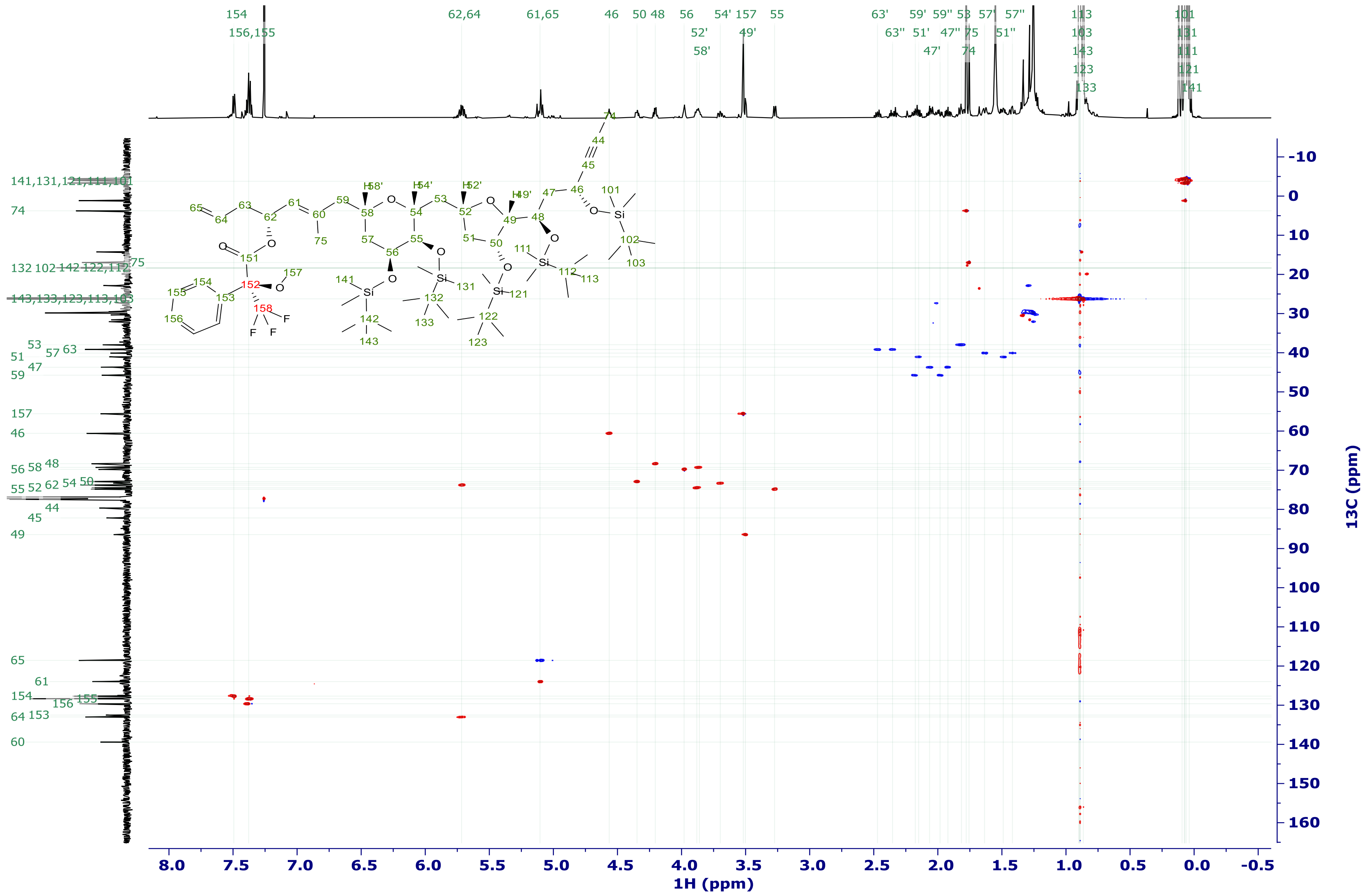

(*S*)-Mosher ester derived from compound 28: HMBC NMR ($CDCl_3$)

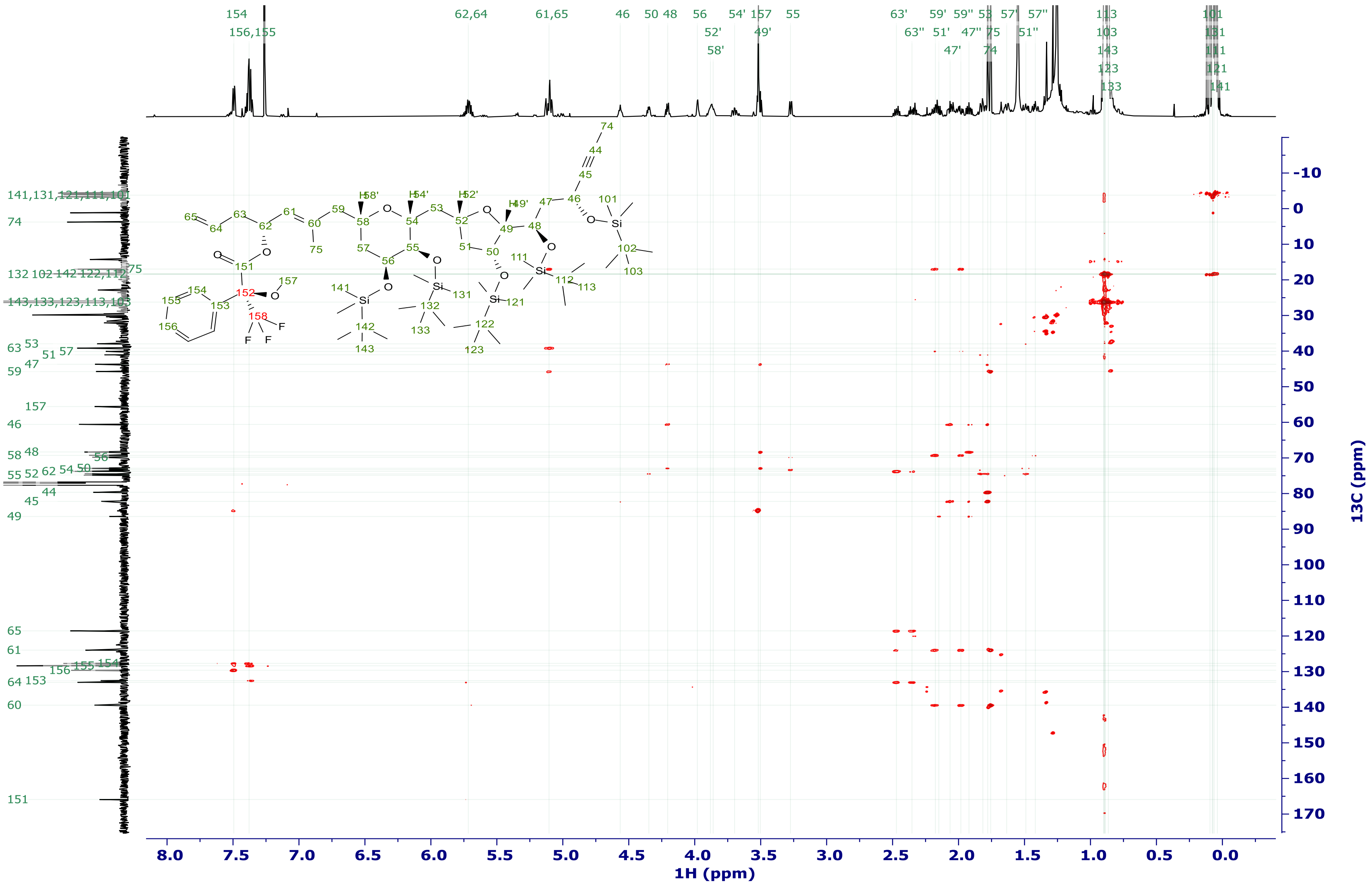

(*S*)-Mosher ester derived from compound 28: NOESY ($CDCl_3$)

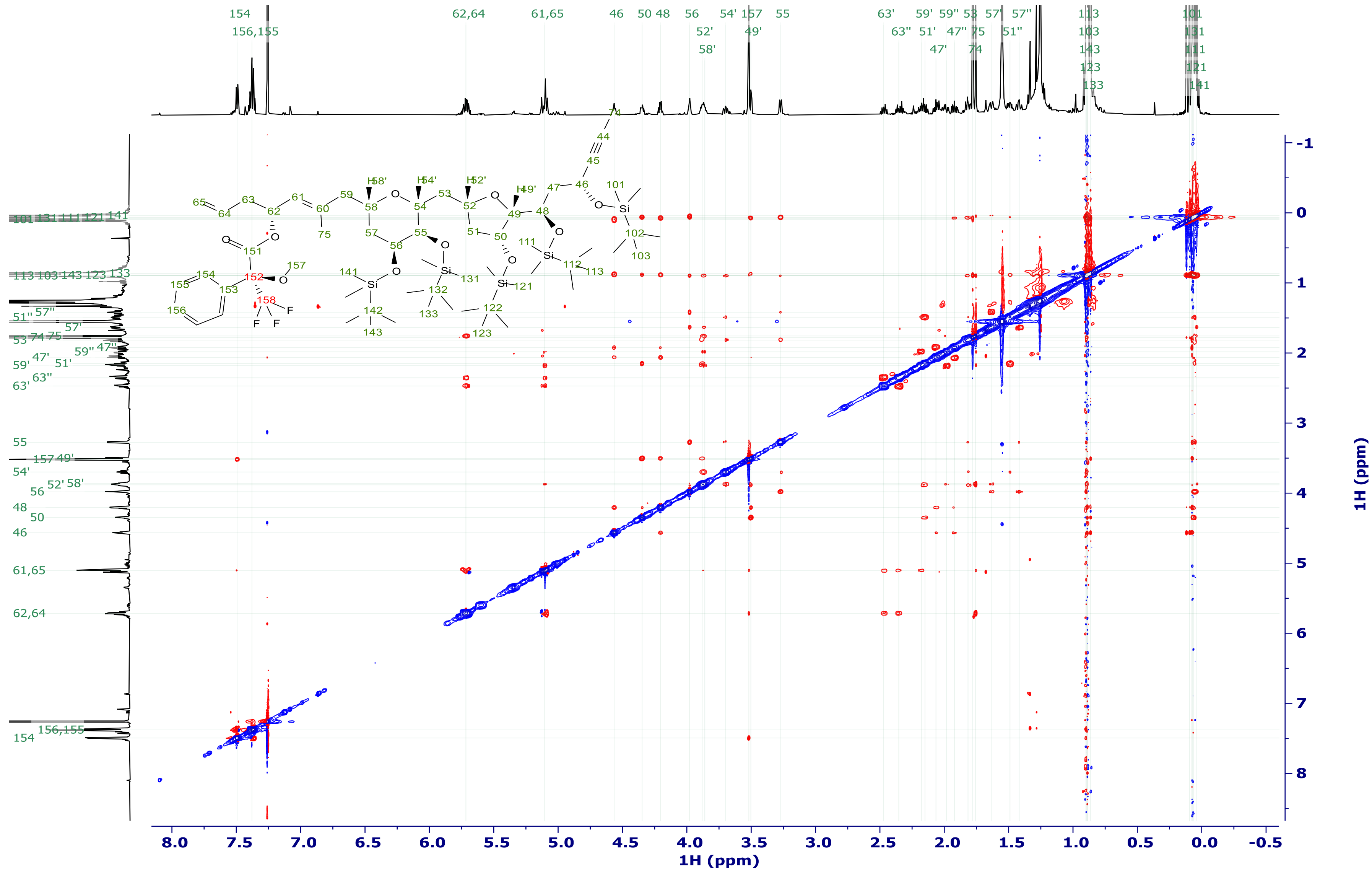

(*R*)-Mosher ester derived from compound 28: ^{1}H NMR (600 MHz, $CDCl_3$)

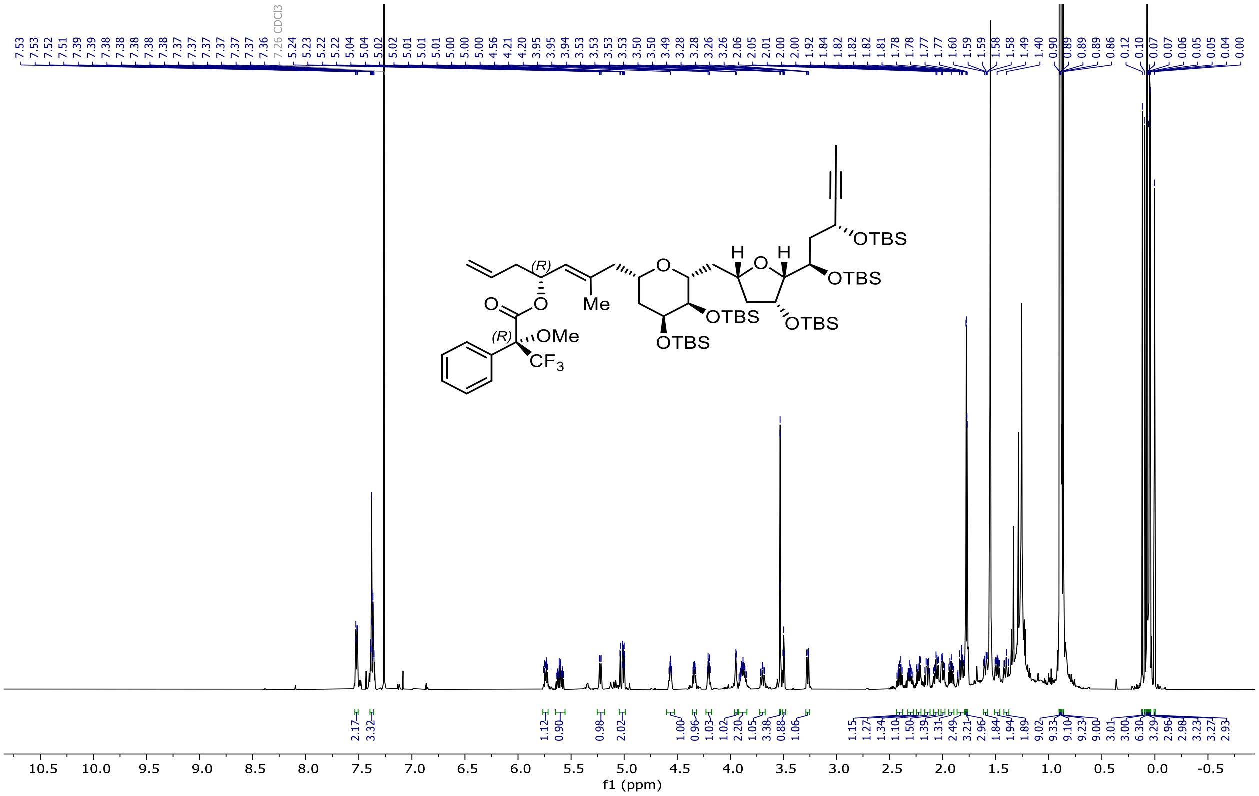

^{13}C NMR (151 MHz, $CDCl_3$)

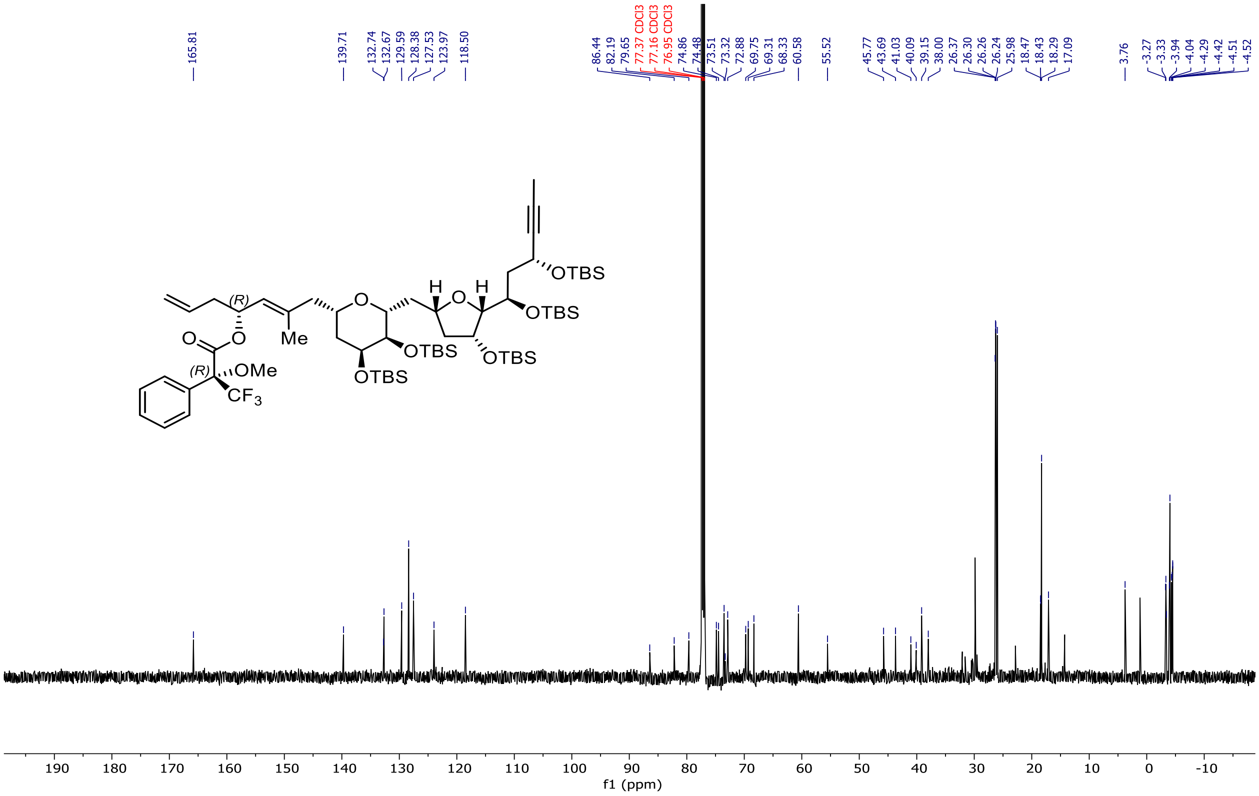

(*R*)-Mosher ester derived from compound 28: ^{1}H-^{1}H COSY ($CDCl_3$)

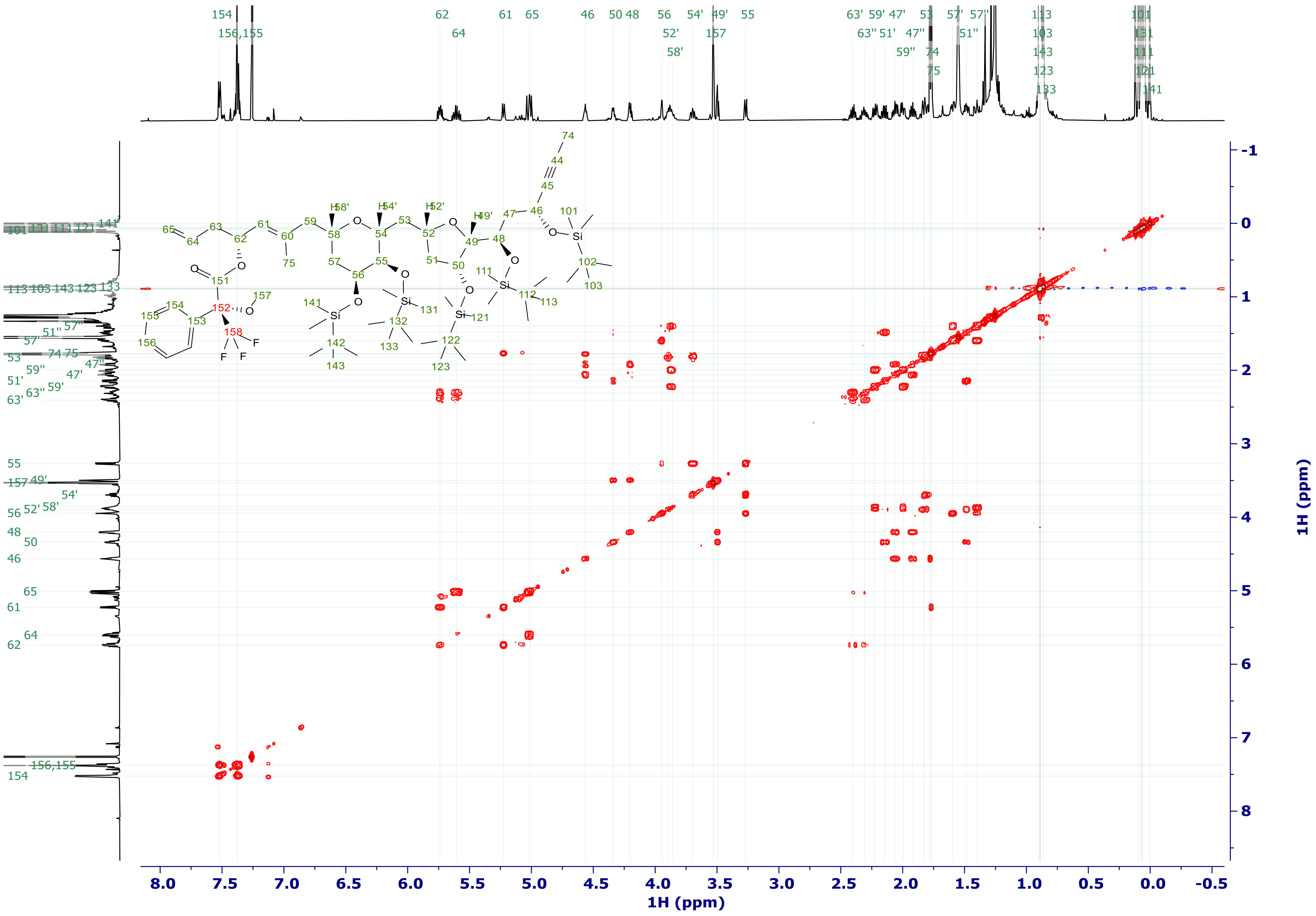

(*R*)-Mosher ester derived from compound 28: HSQC NMR ($CDCl_3$)

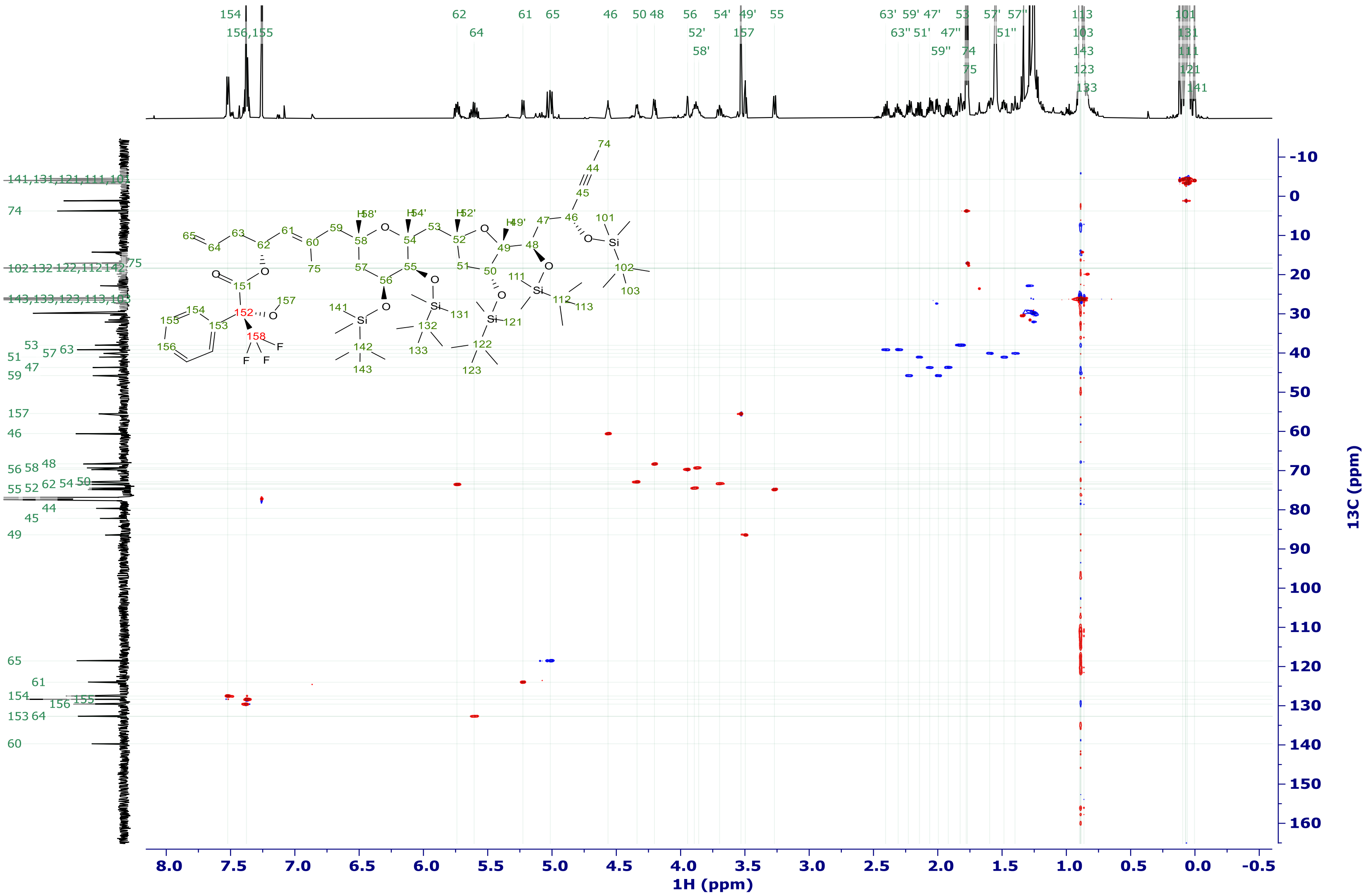

(*R*)-Mosher ester derived from compound 28: HMBC NMR ($CDCl_3$)

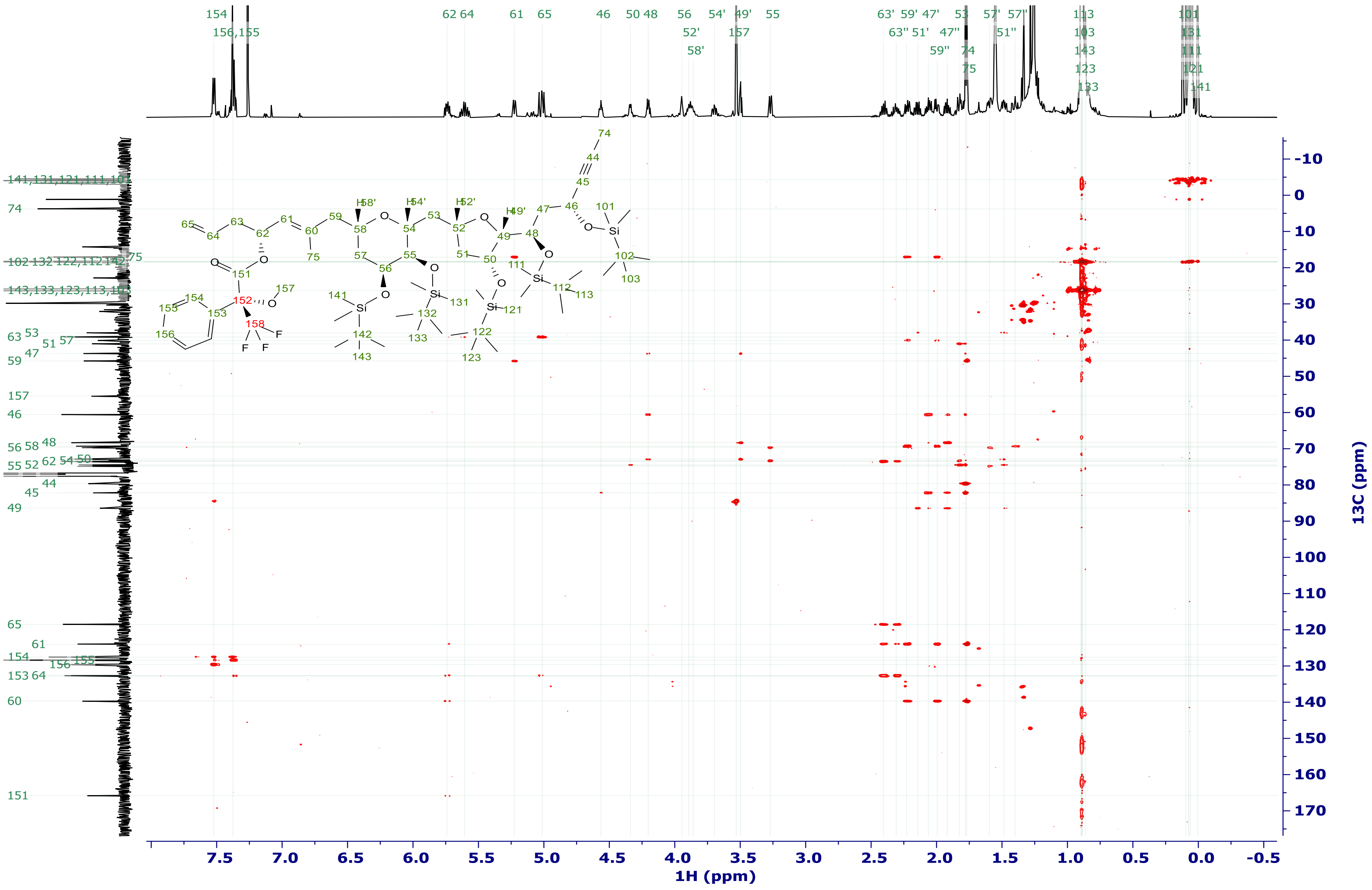

(*R*)-Mosher ester derived from compound 28: NOESY ($CDCl_3$)

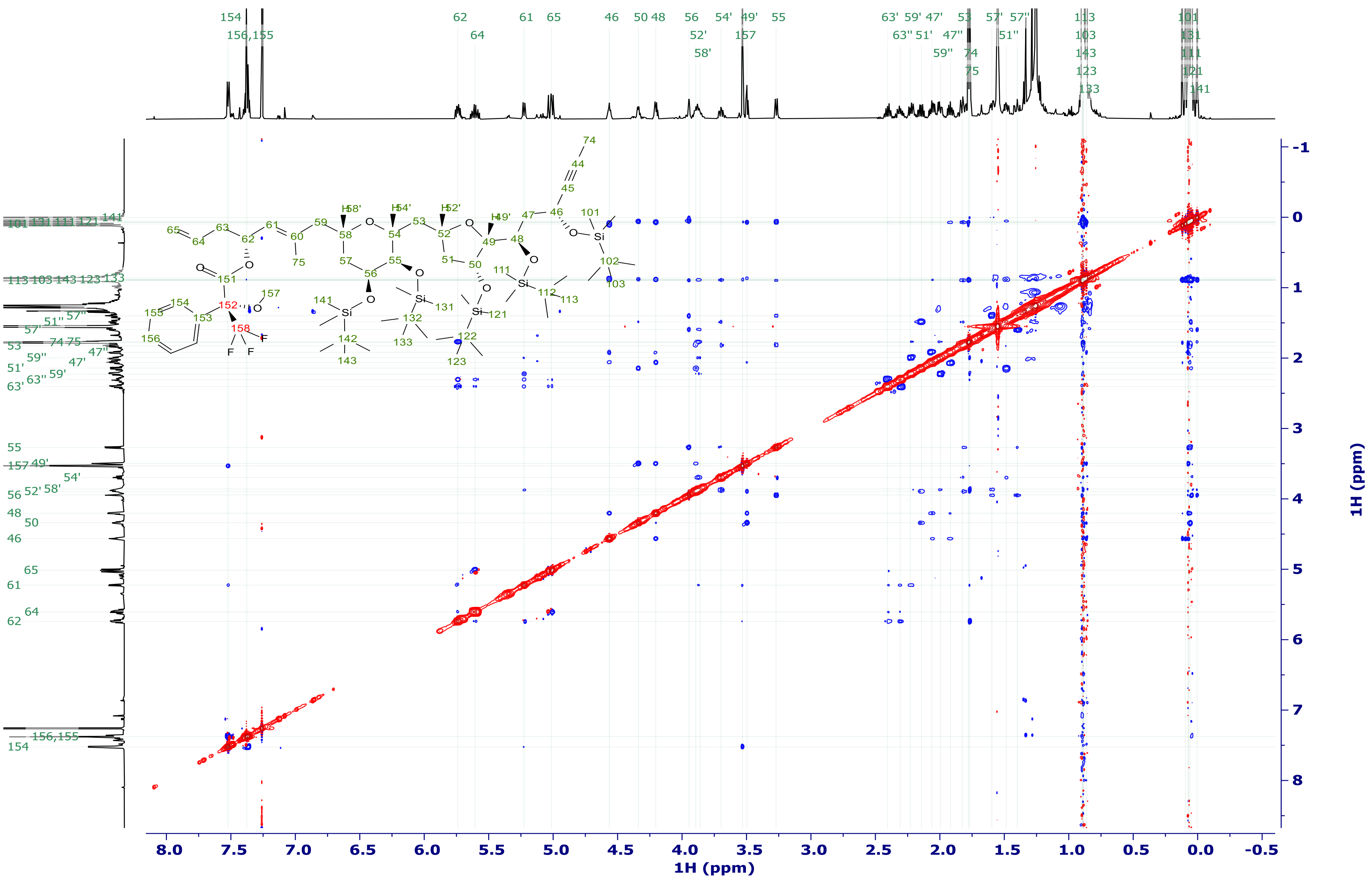

Compound 29: ^{1}H NMR (400 MHz, $CDCl_3$)

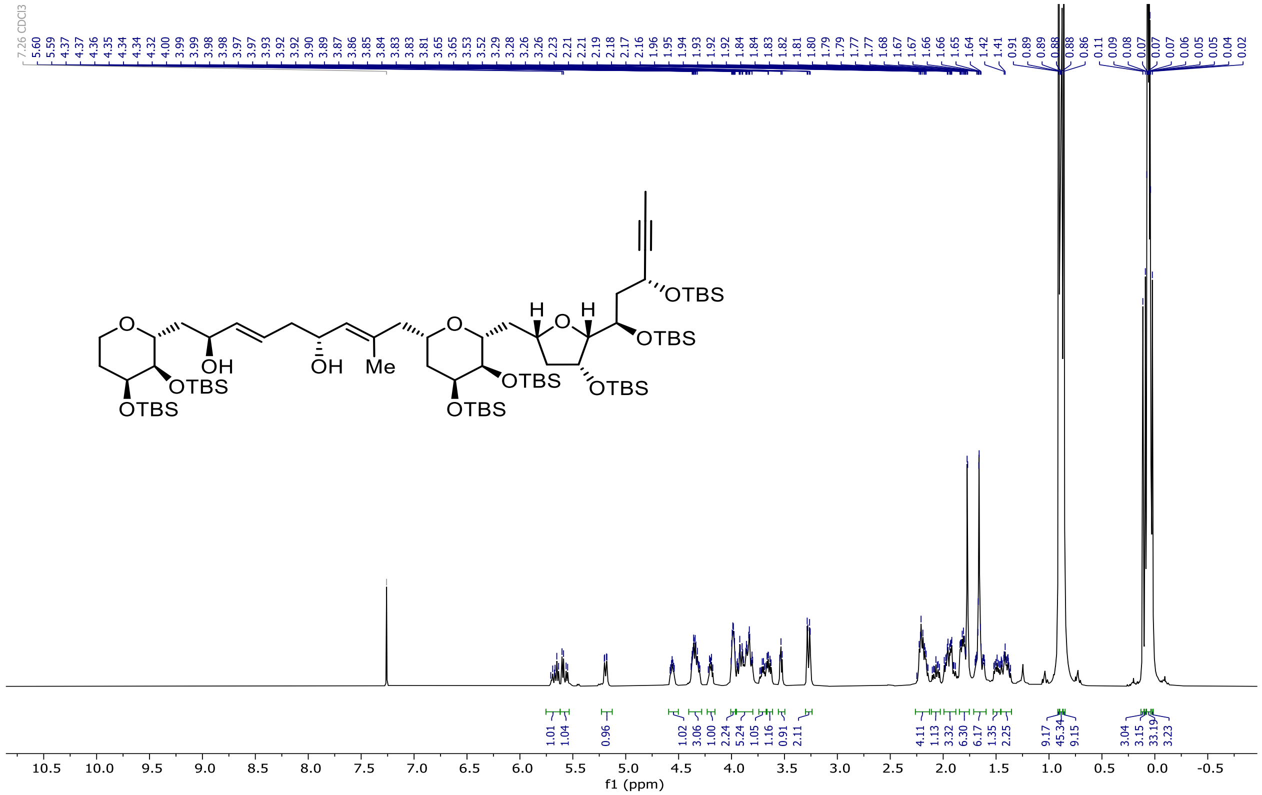

^{13}C NMR (101 MHz, $CDCl_3$)

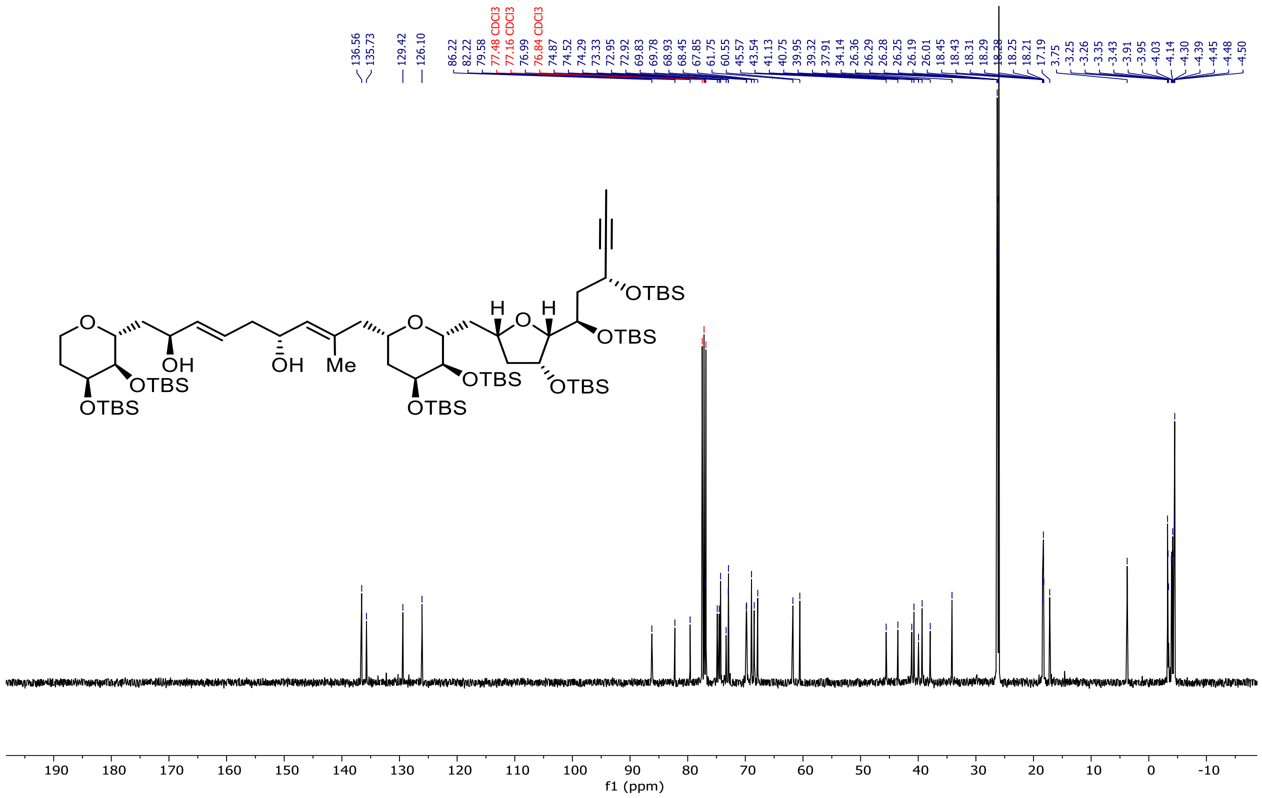

Compound S14: ^{1}H NMR (400 MHz, $CDCl_3$)

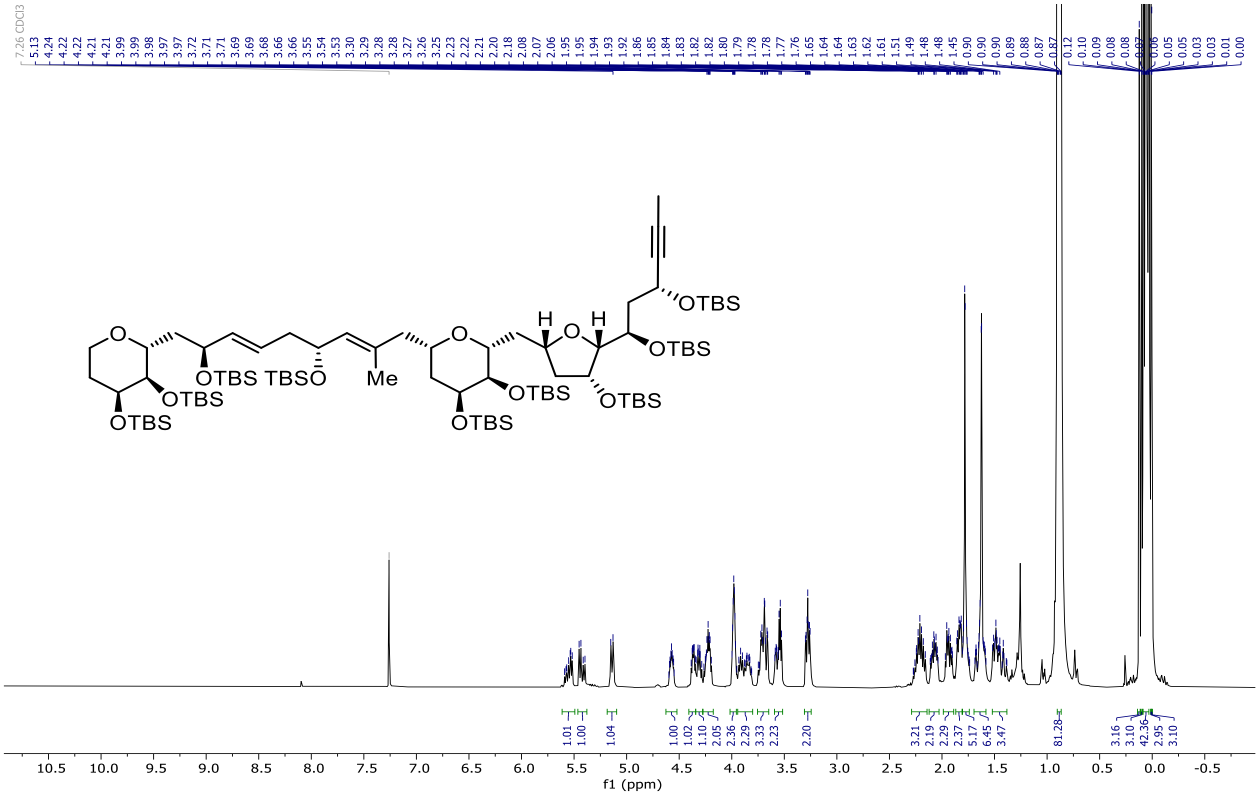

^{13}C NMR (101 MHz, $CDCl_3$)

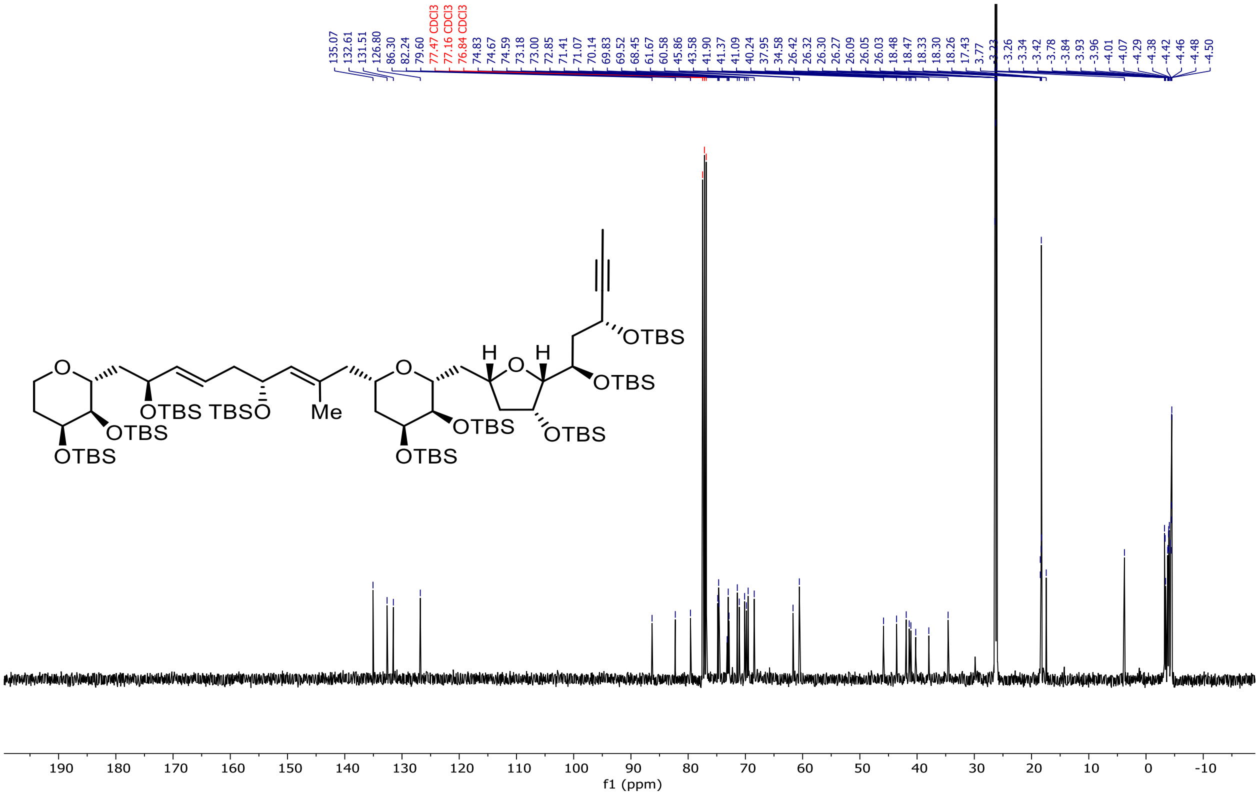

Compound 30: ^{1}H NMR (400 MHz, C_6D_6)

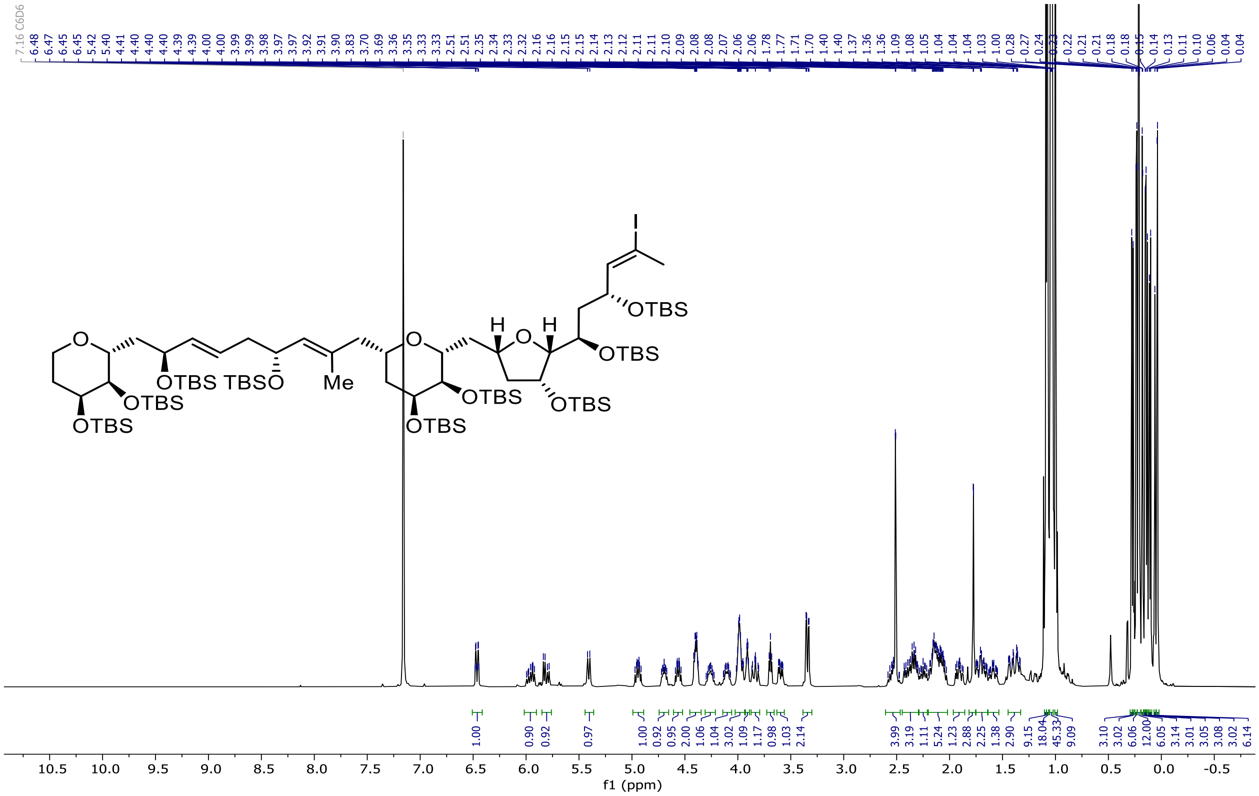

^{13}C NMR (101 MHz, C_6D_6)

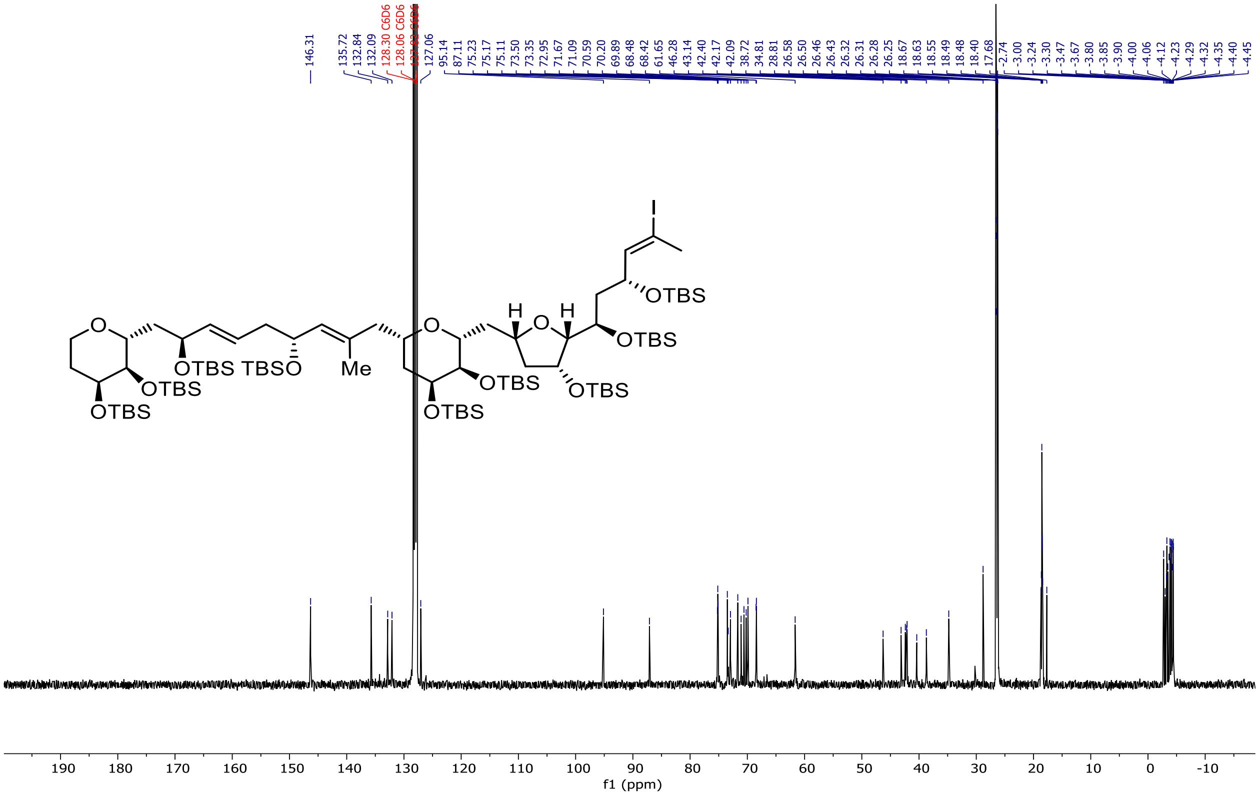

Compound 32: ^{1}H NMR (600 MHz, [D$_4$]-MeOH)

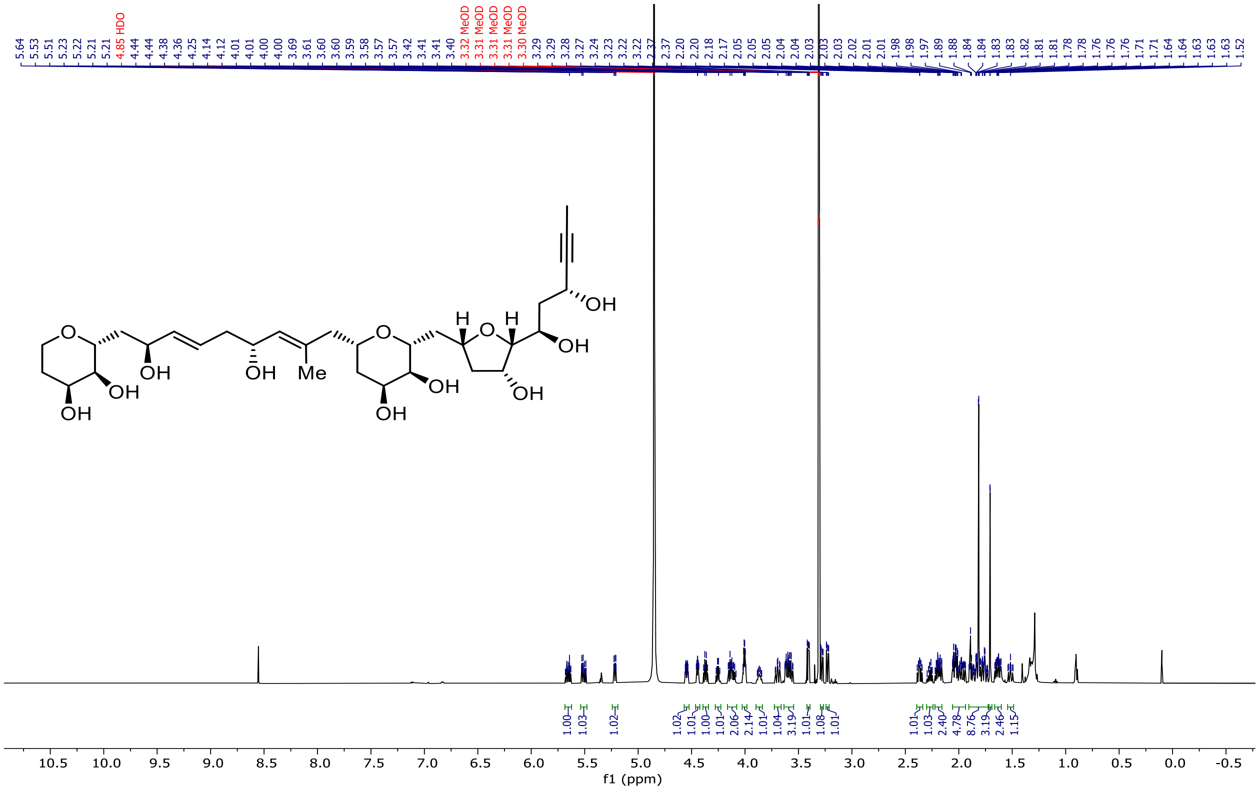

^{13}C NMR (151 MHz, [D$_4$]-MeOH)

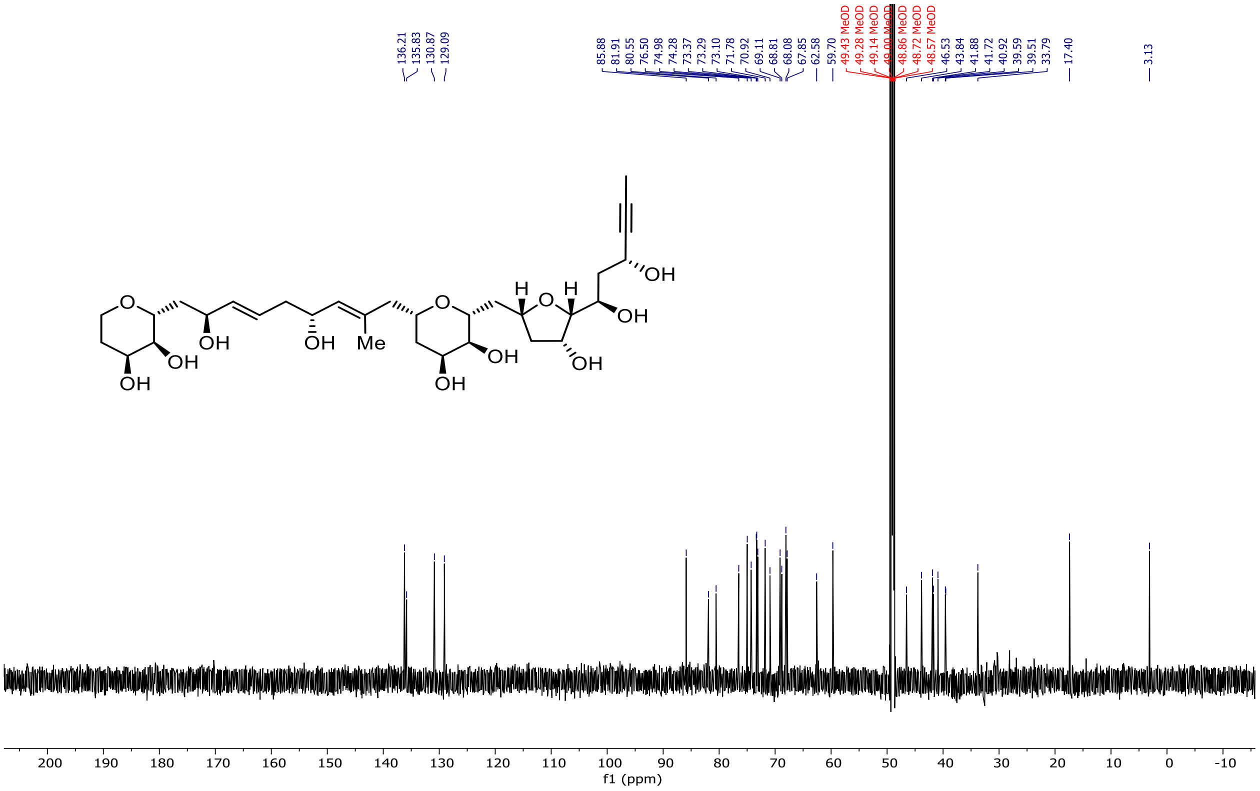

Compound 32: ^{1}H-^{1}H COSY ([D$_4$]-MeOH)

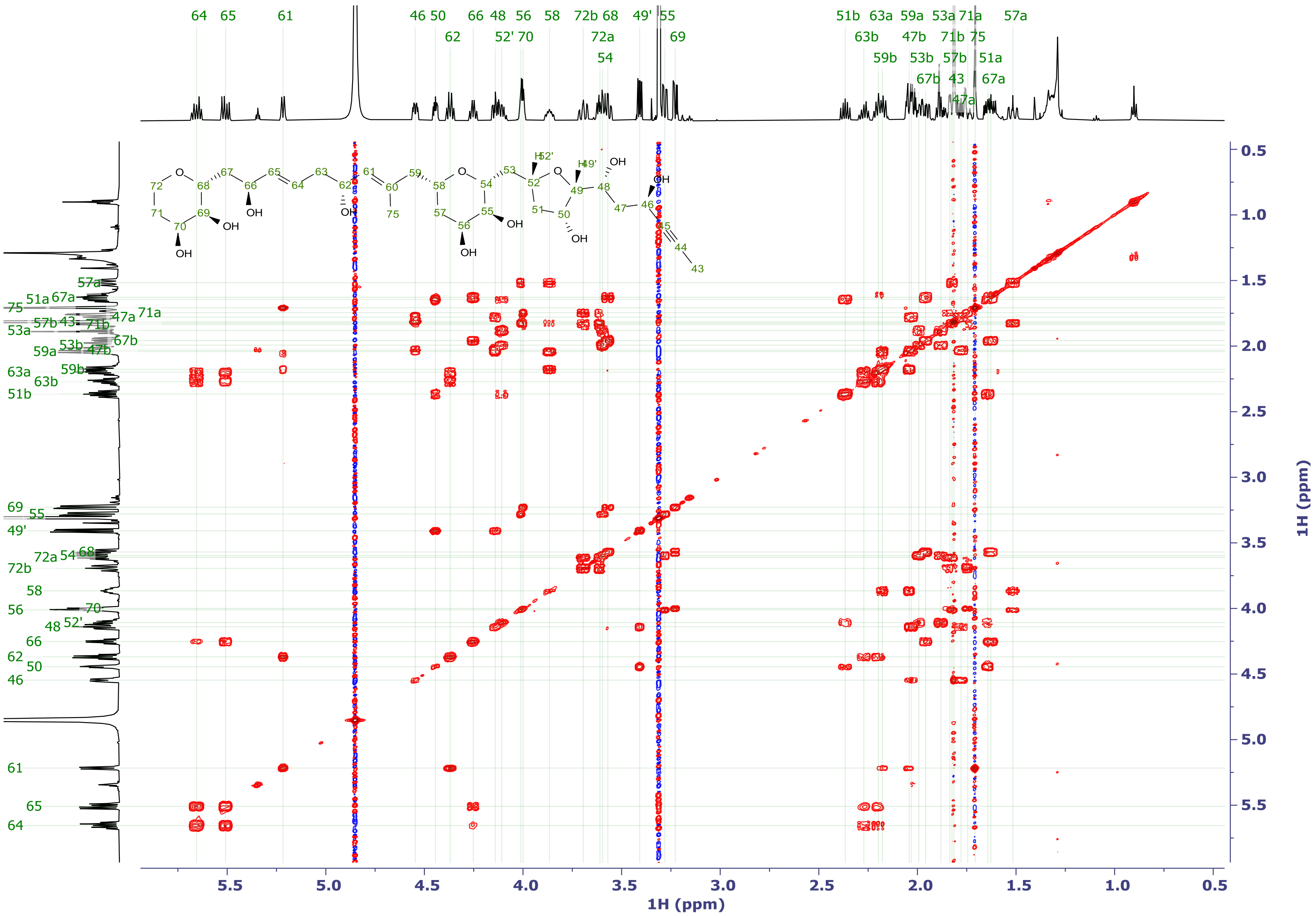

Compound 32: HSQC NMR ([D$_4$]-MeOH)

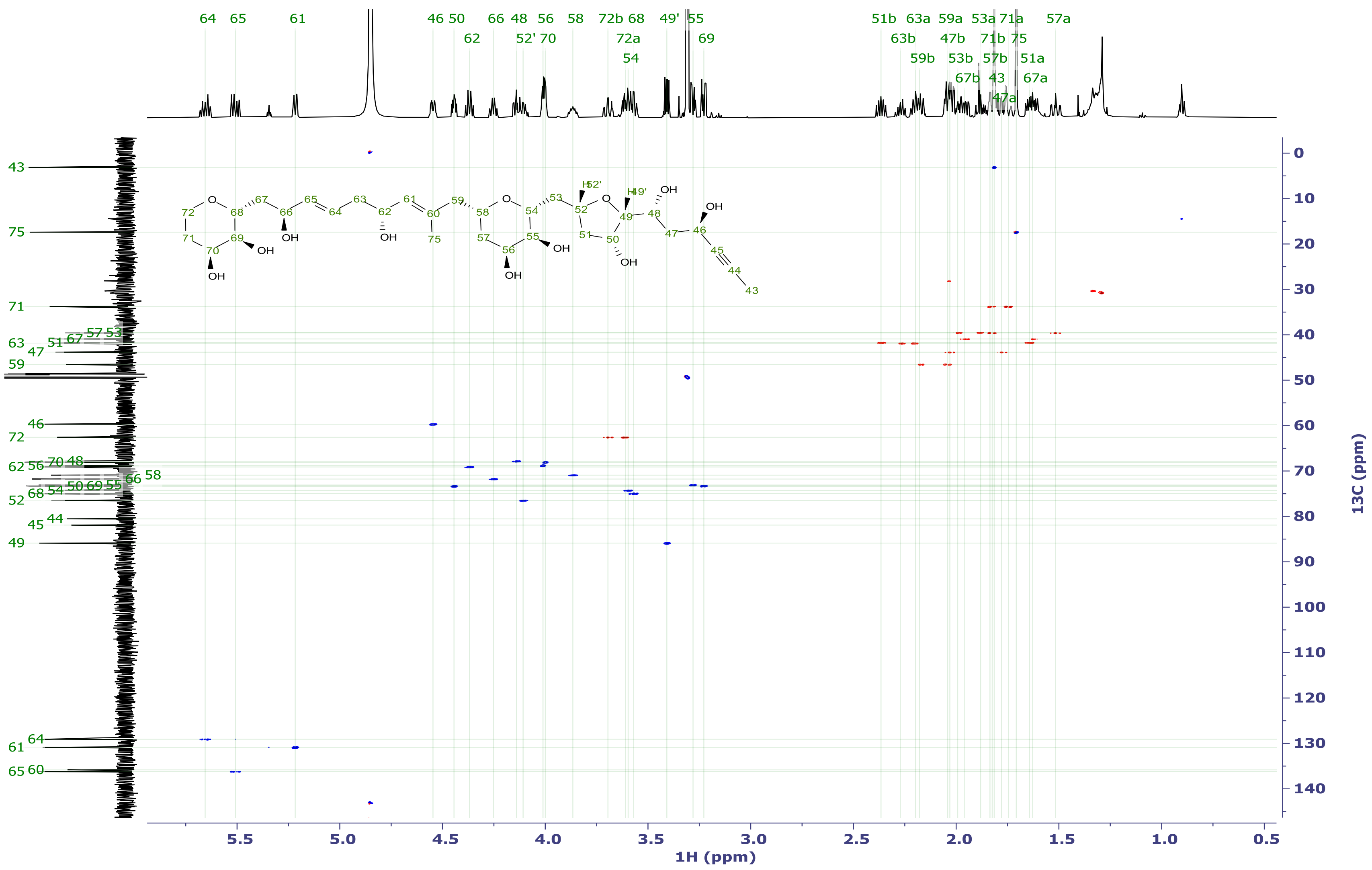

Compound 32: HMBC NMR ([D$_4$]-MeOH)

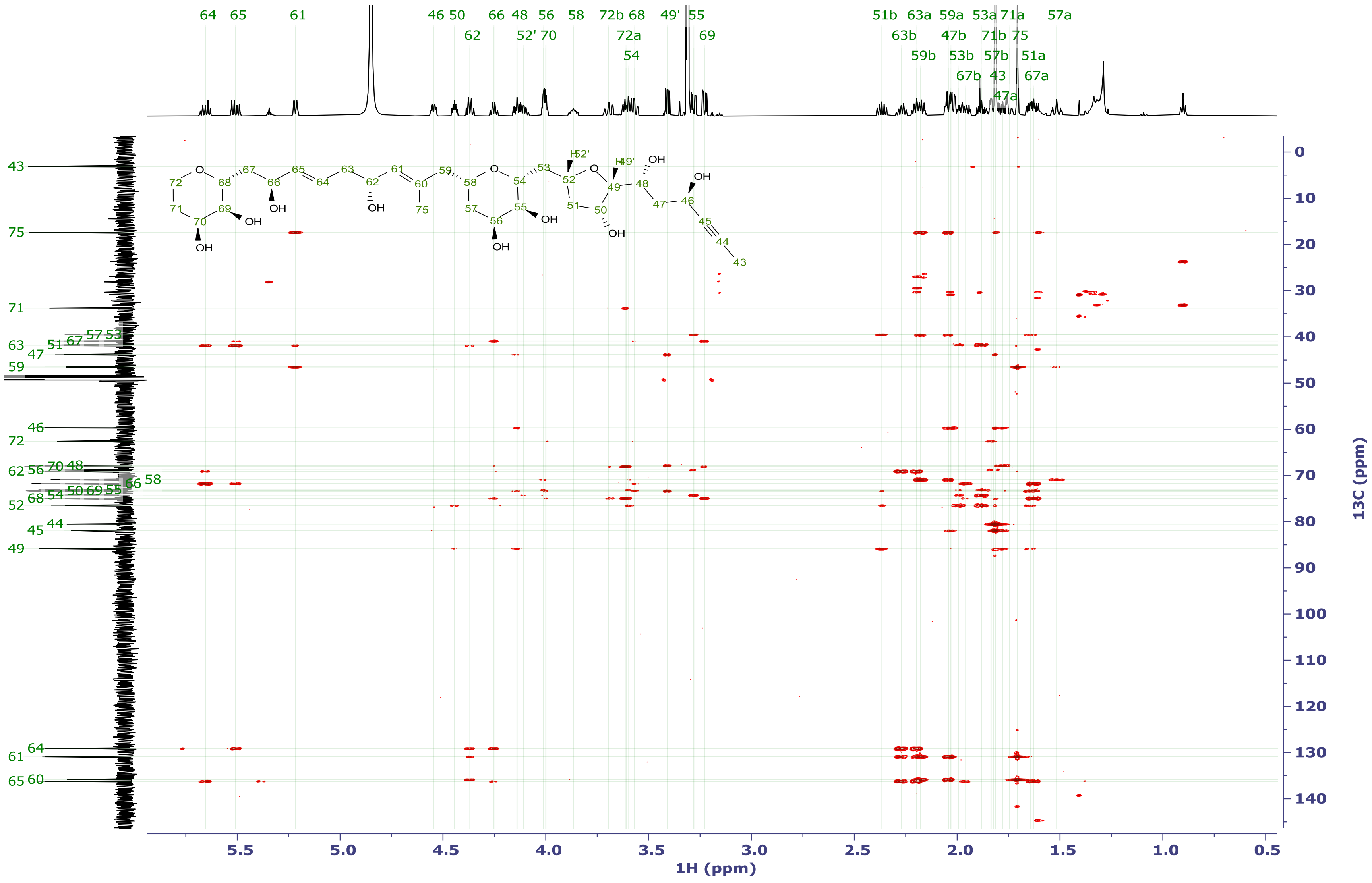

Compound 32: NOESY ([D$_4$]-MeOH)

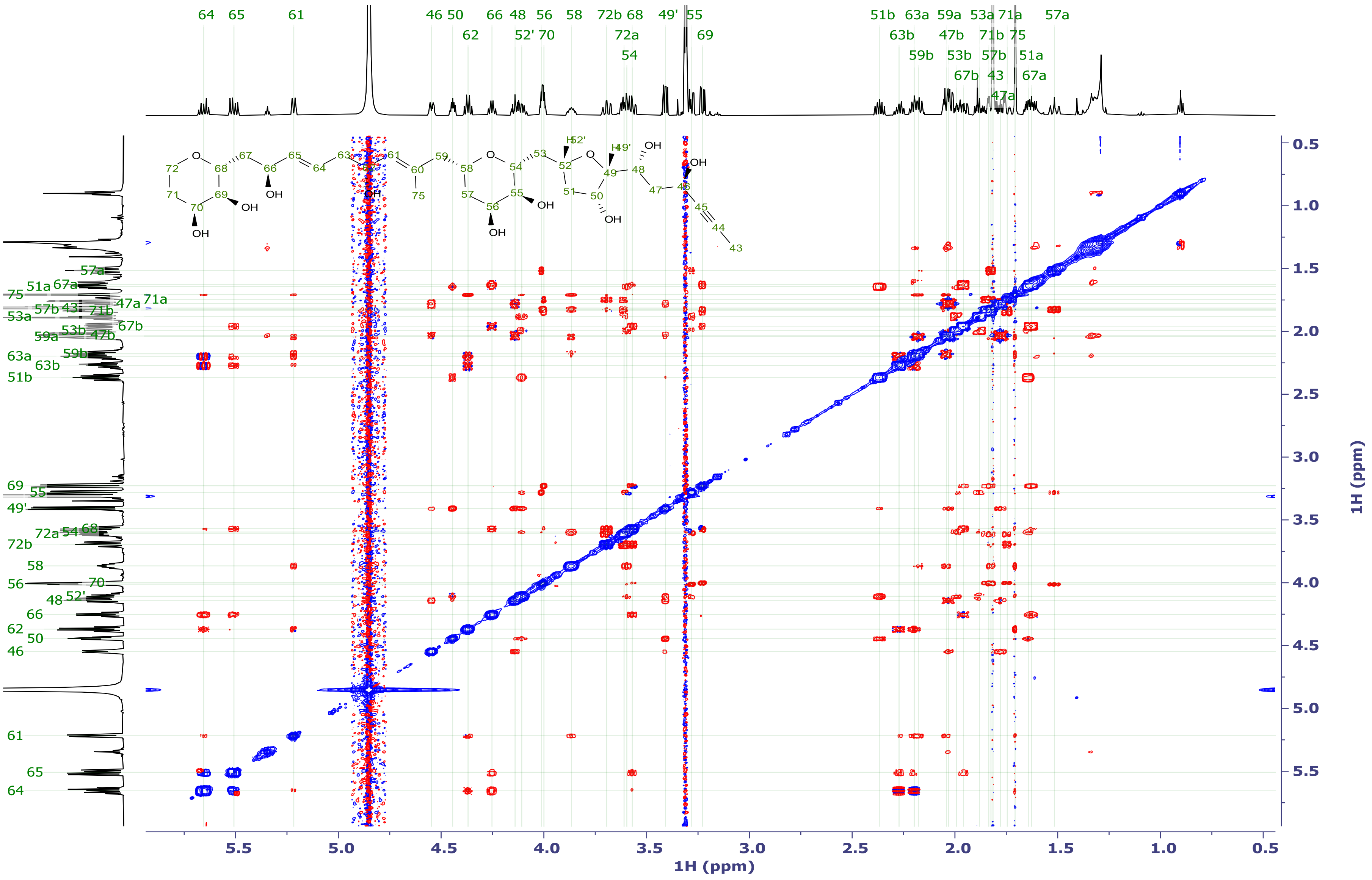

Compound S15: ^{1}H NMR ($CDCl_3$, 600 MHz)

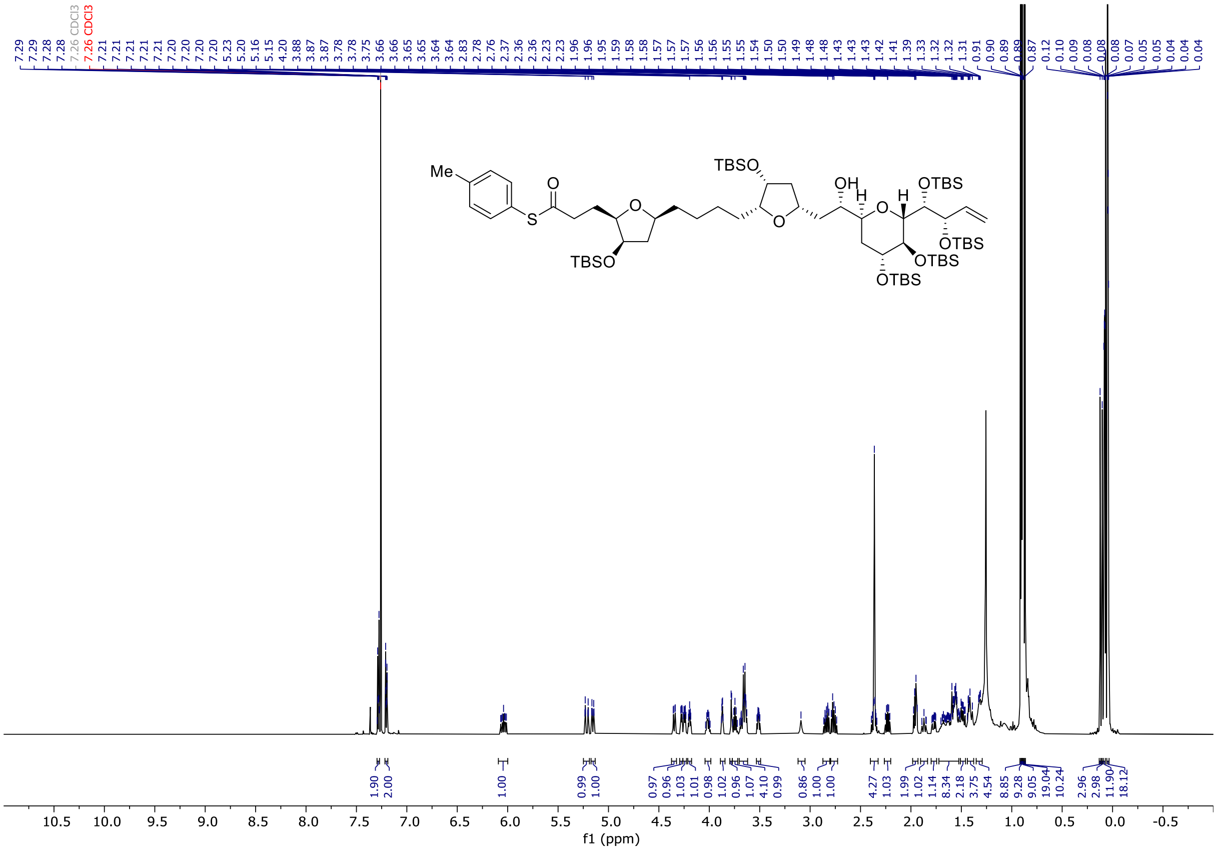

^{13}C NMR ($CDCl_3$, 151 MHz)

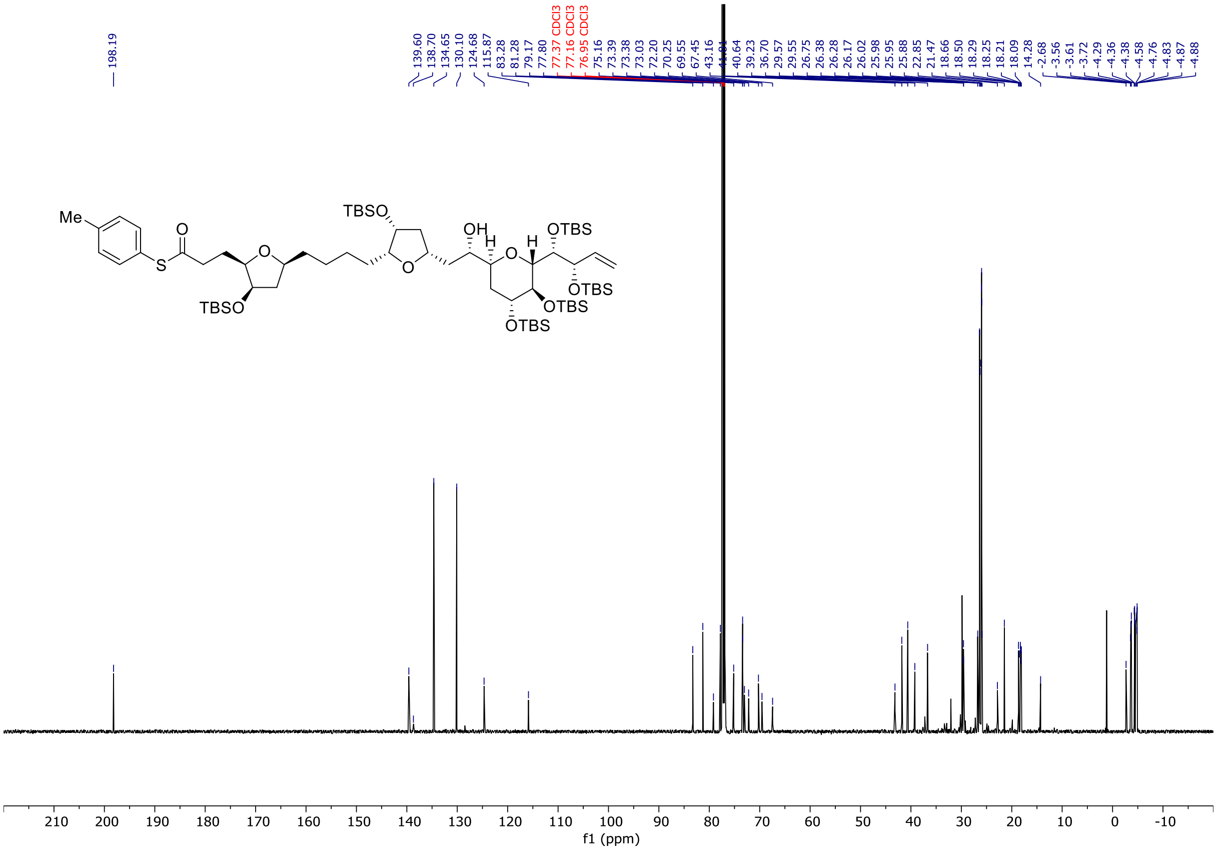

Compound S16: ^{1}H NMR ($CDCl_3$, 600 MHz)

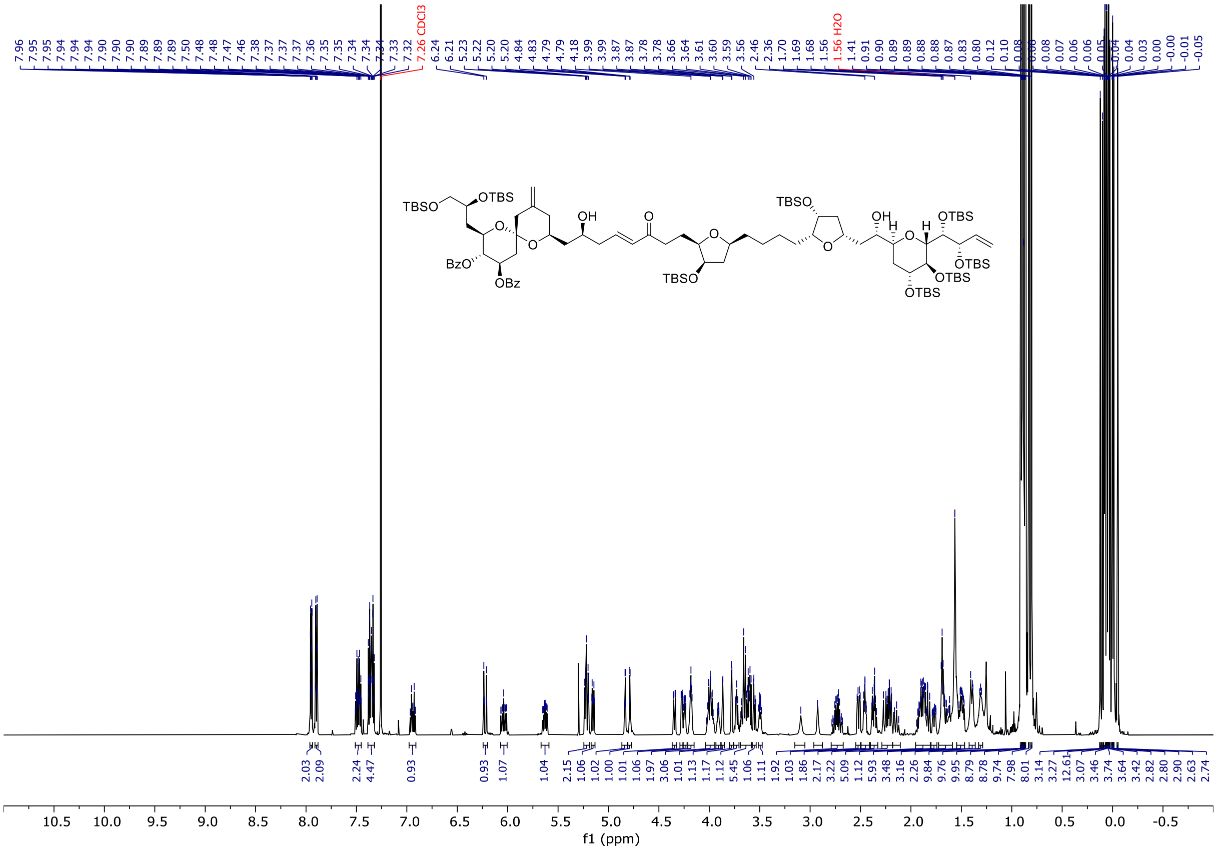

^{13}C NMR ($CDCl_3$, 151 MHz)

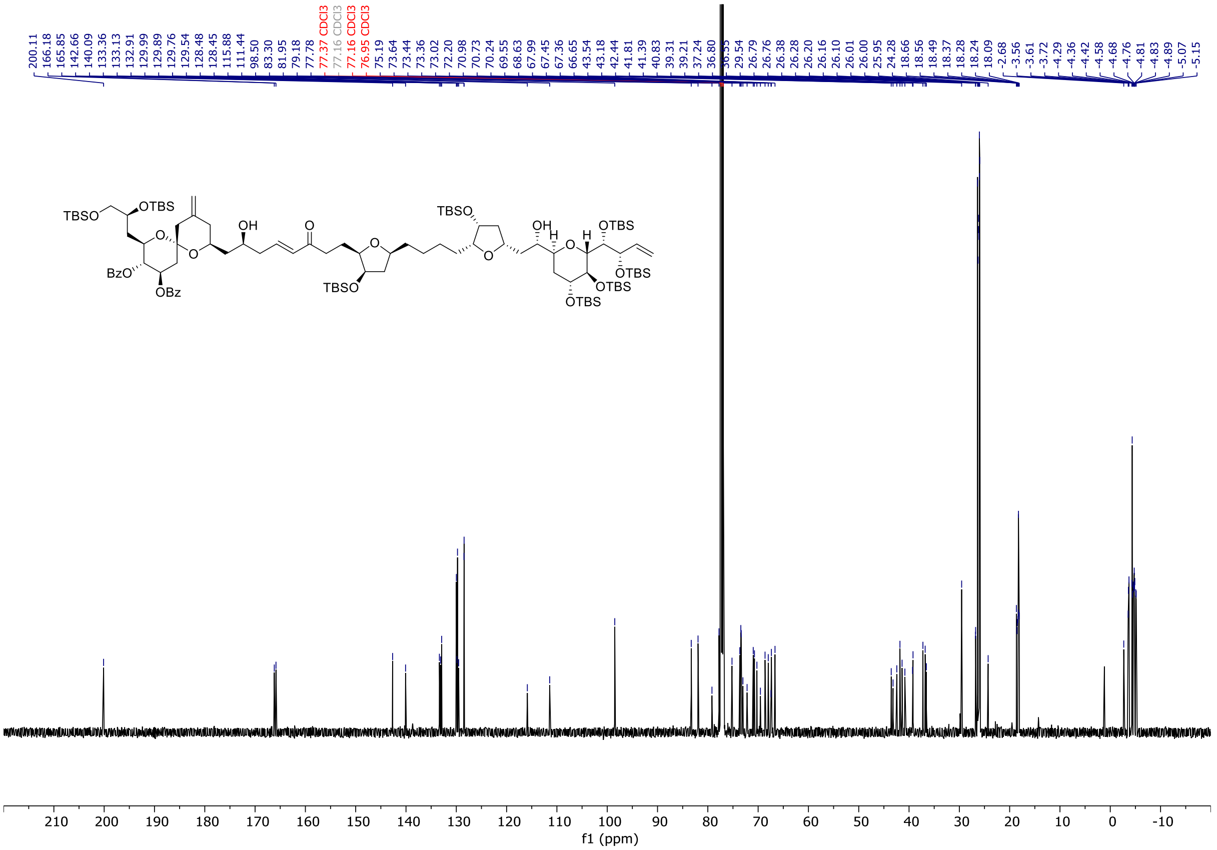

Compound 33: ^{1}H NMR ($CDCl_3$, 600 MHz)

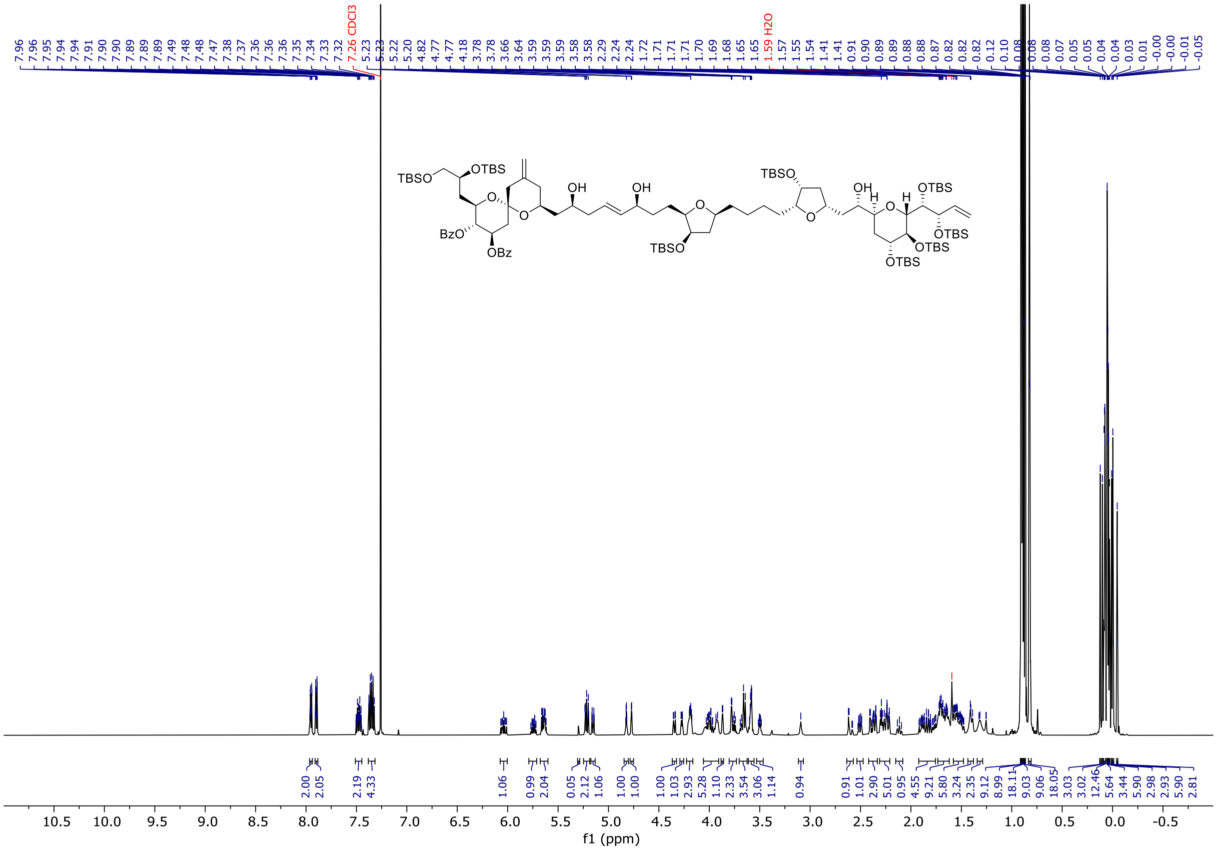

^{13}C NMR ($CDCl_3$, 151 MHz)

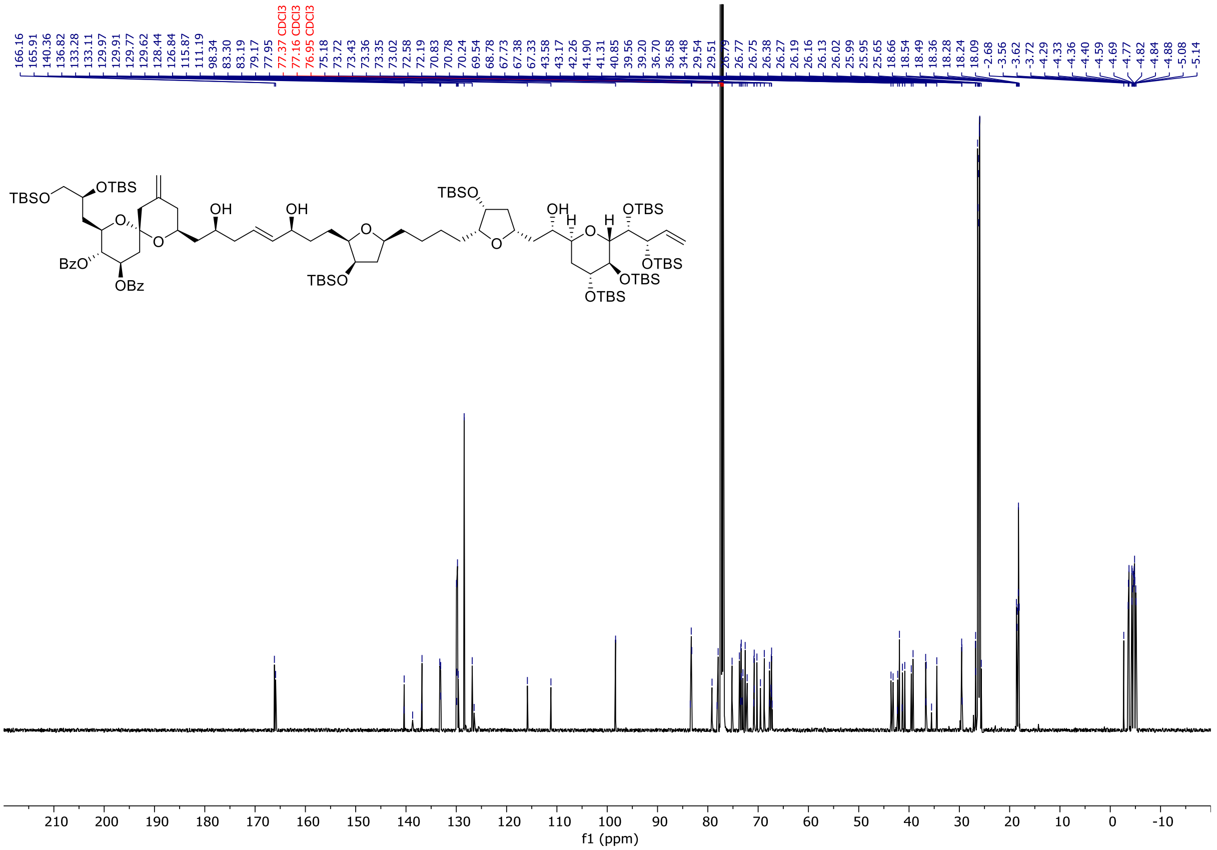

Compound 37: ^{1}H NMR (400 MHz, CD_2Cl_2)

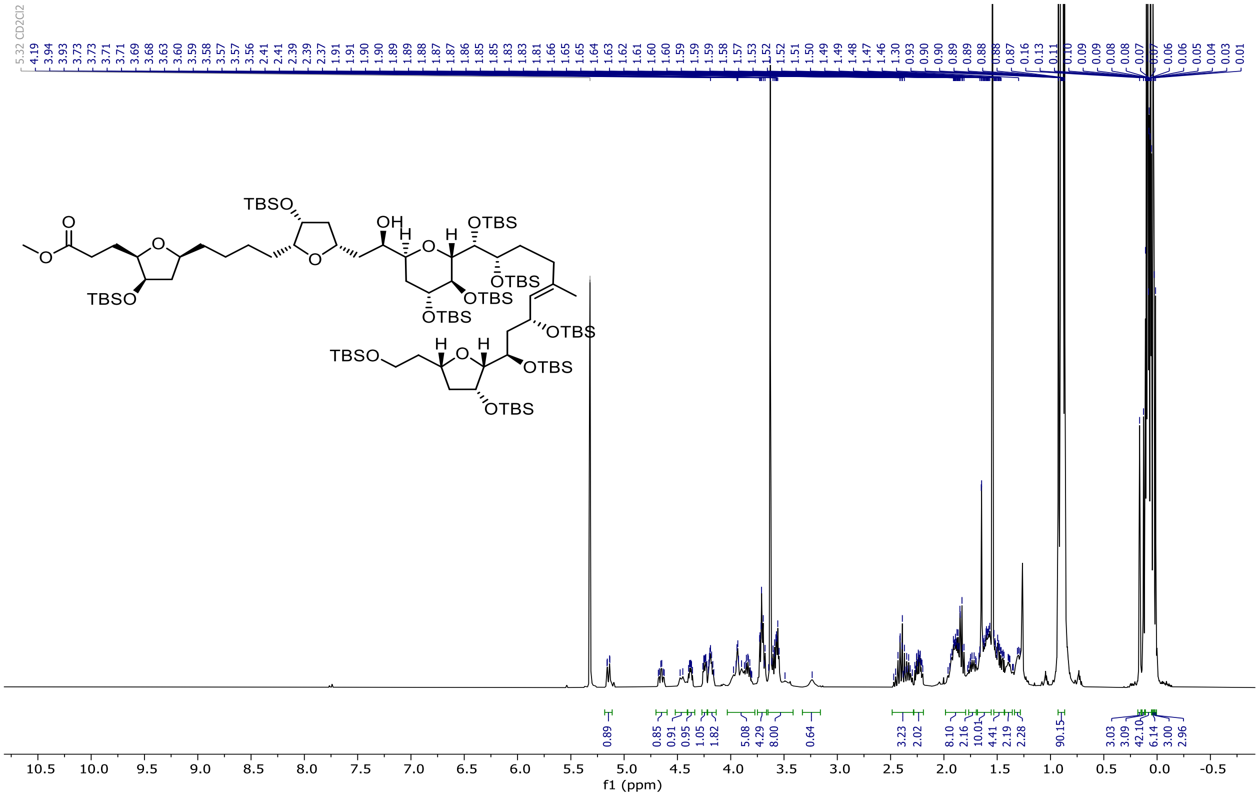

^{13}C NMR (101 MHz, CD_2Cl_2)

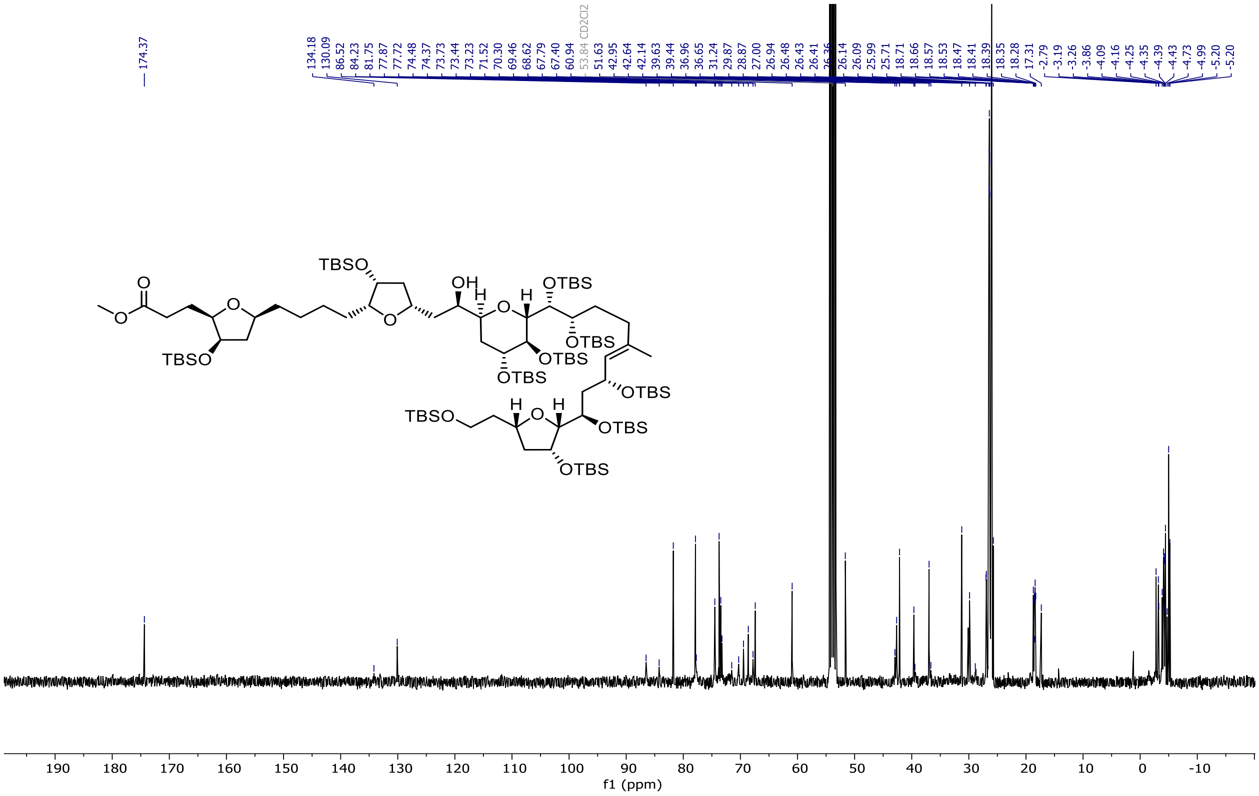

Compound 39: ^{1}H NMR (600 MHz, C_6D_6)

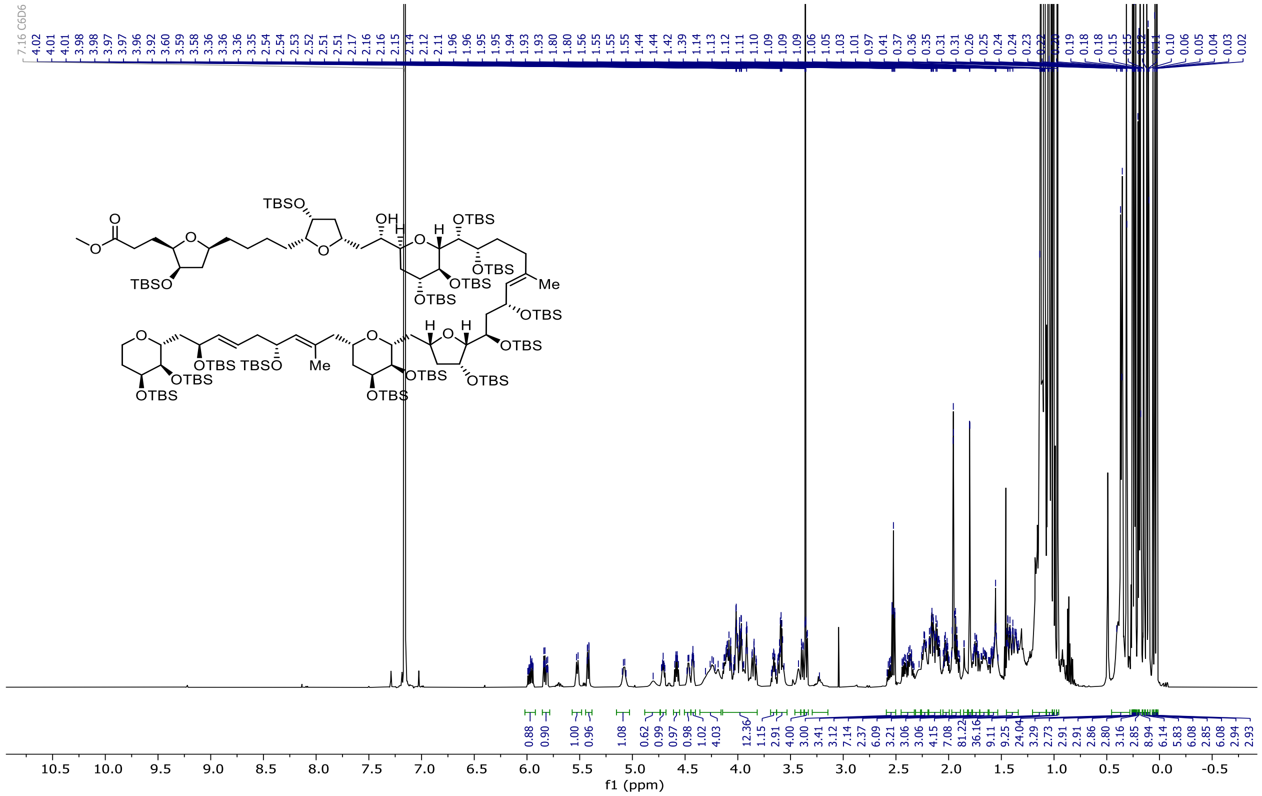

^{13}C NMR (151 MHz, C_6D_6)

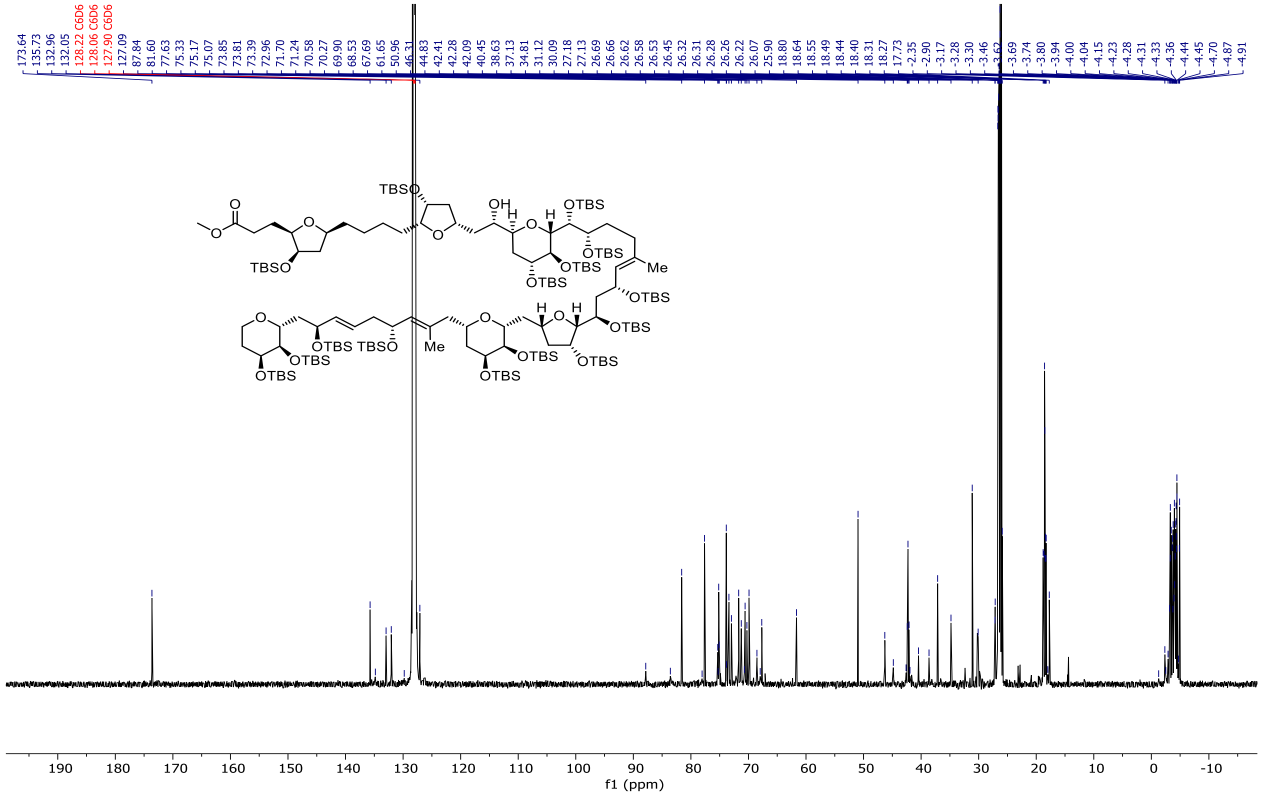

Compound 40: ^{1}H NMR (600 MHz, C_6D_6)

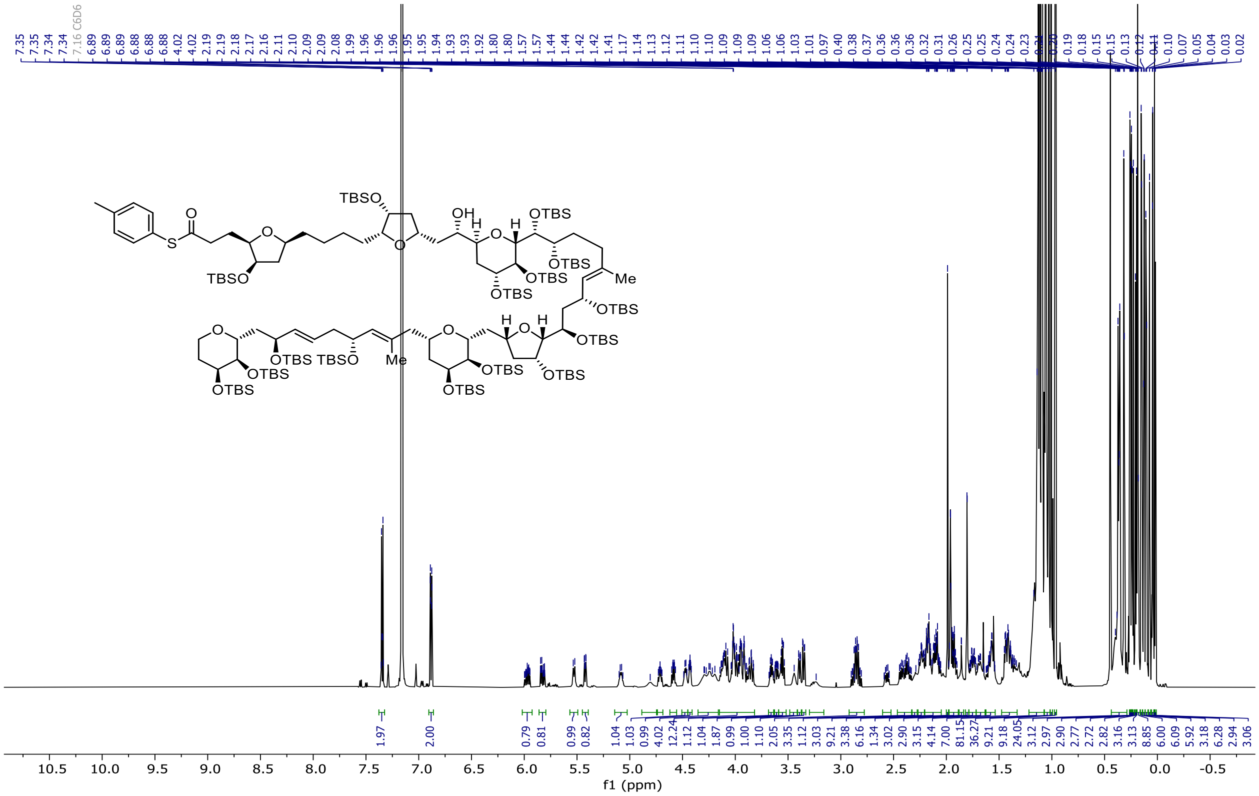

^{13}C NMR (151 MHz, C_6D_6)

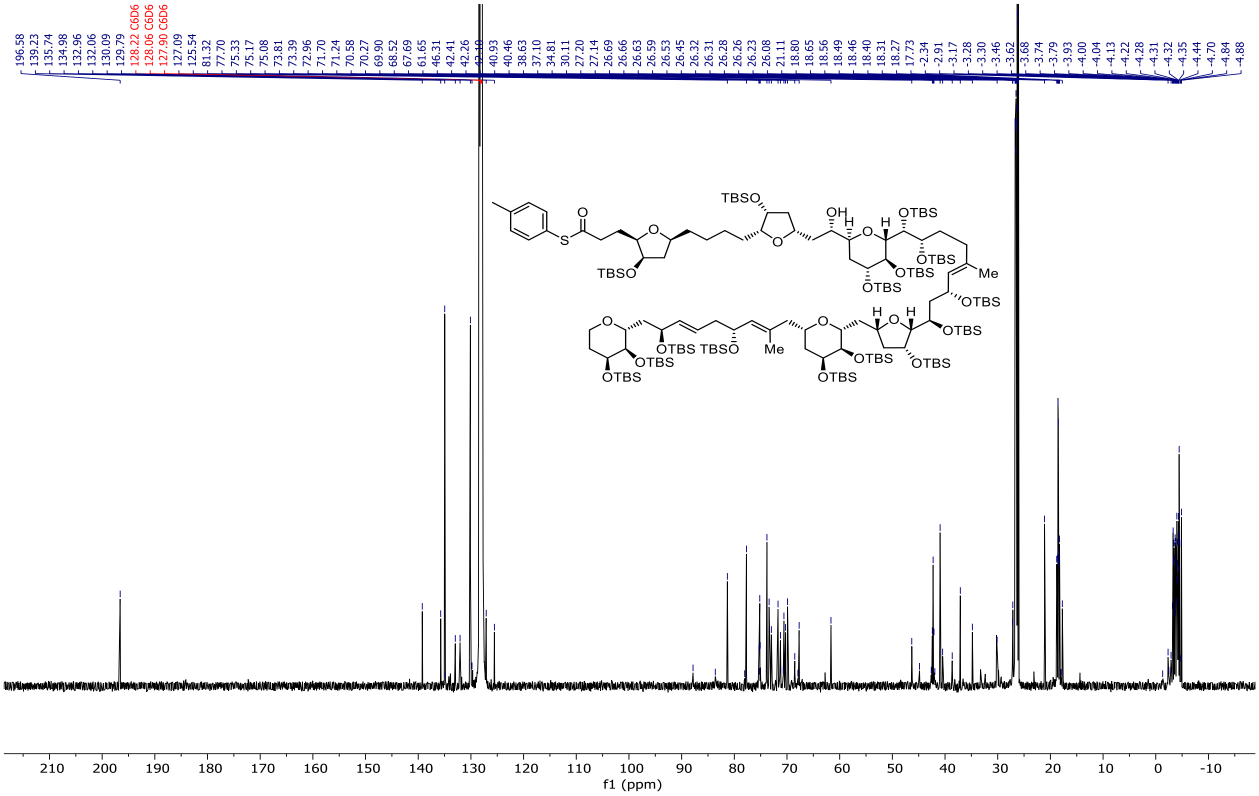

Compound 42: ^{1}H NMR (600 MHz, C_6D_6)

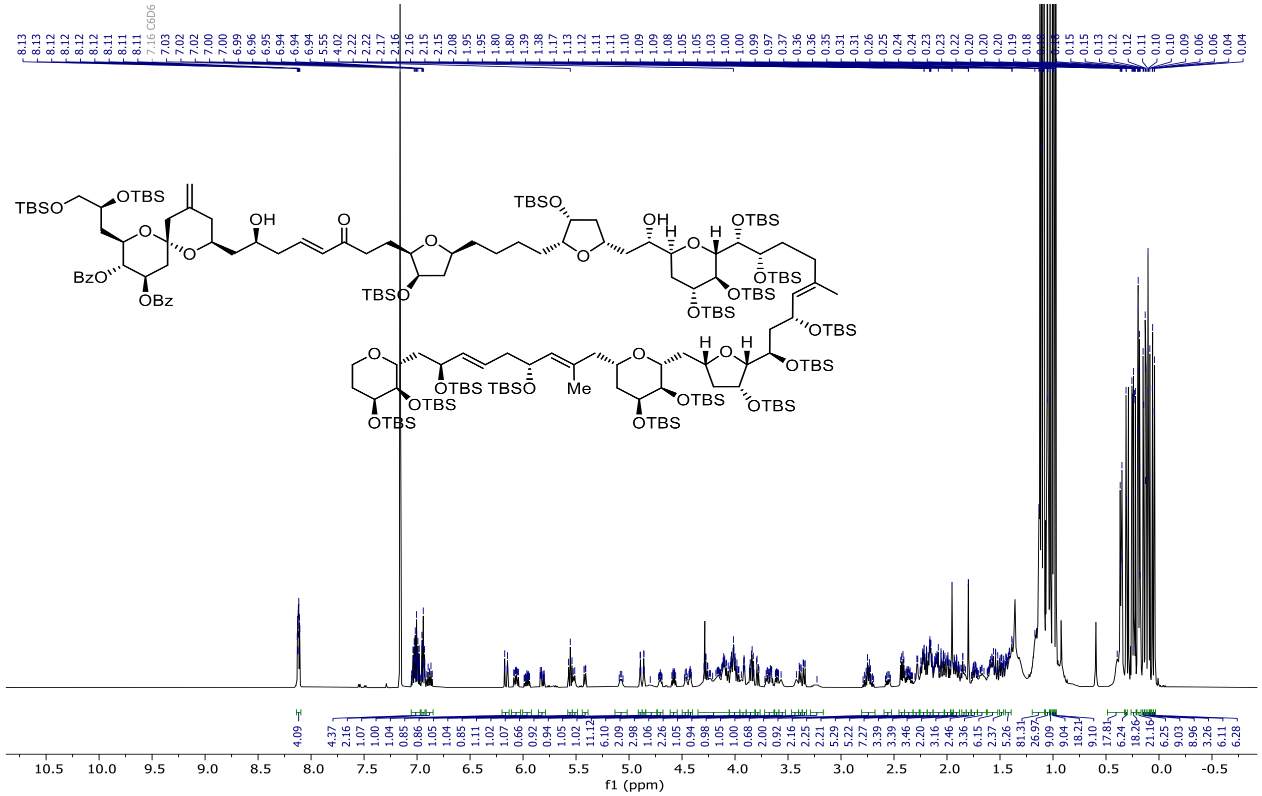

^{13}C NMR (151 MHz, C_6D_6)

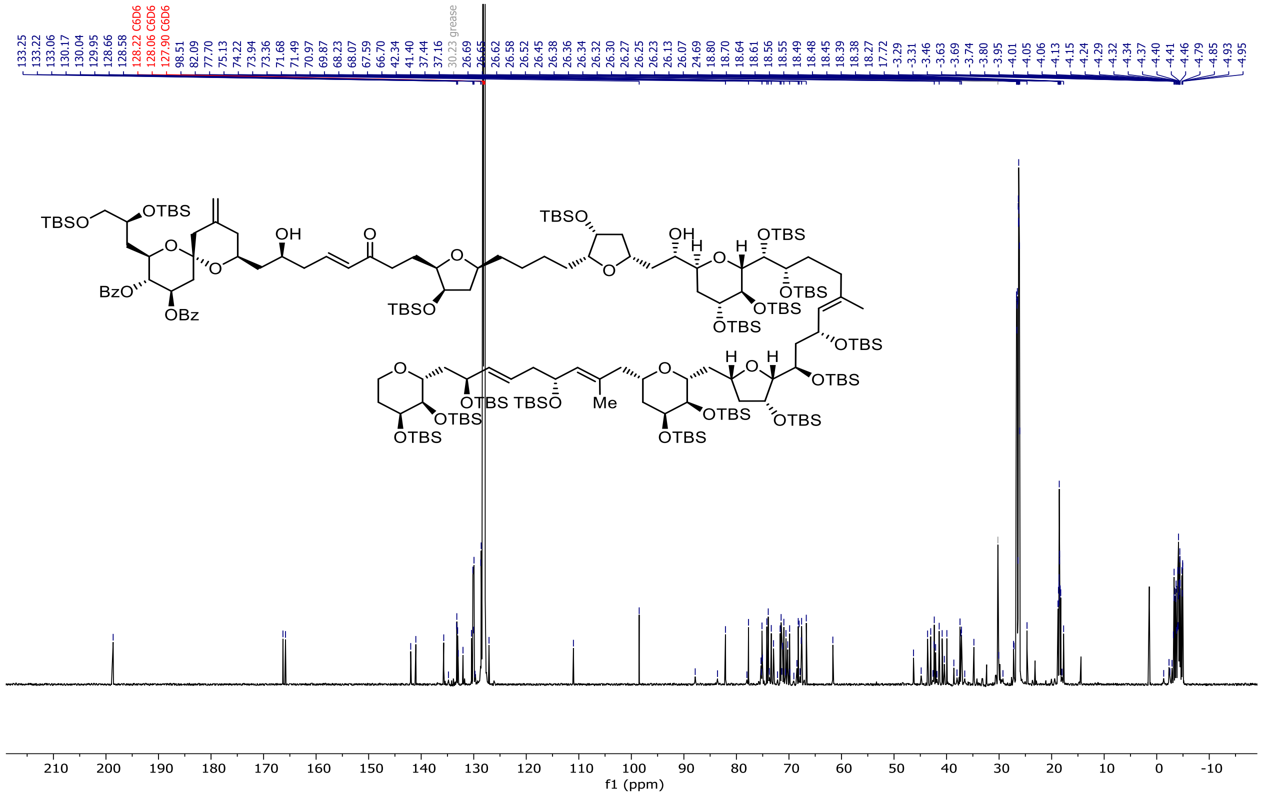

Compound 43: ^{1}H NMR (600 MHz, C_6D_6)

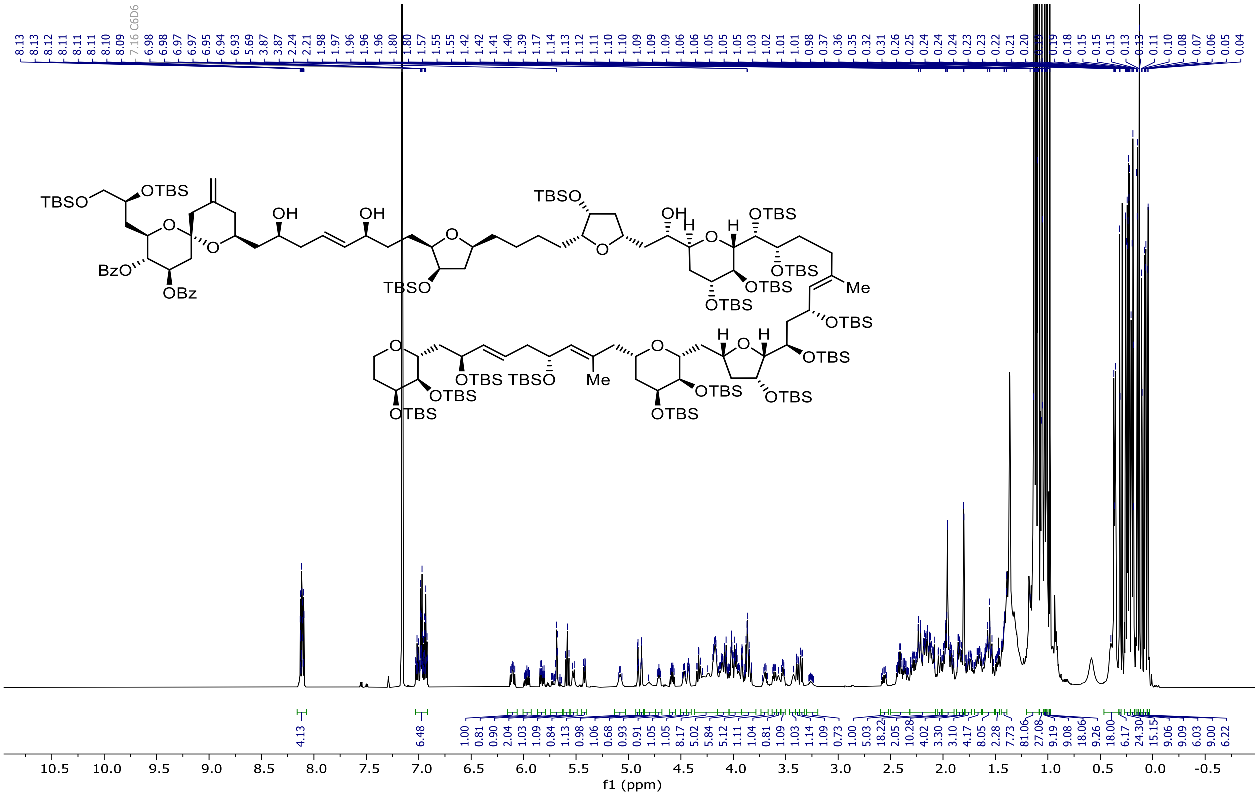

^{13}C NMR (151 MHz, C_6D_6)

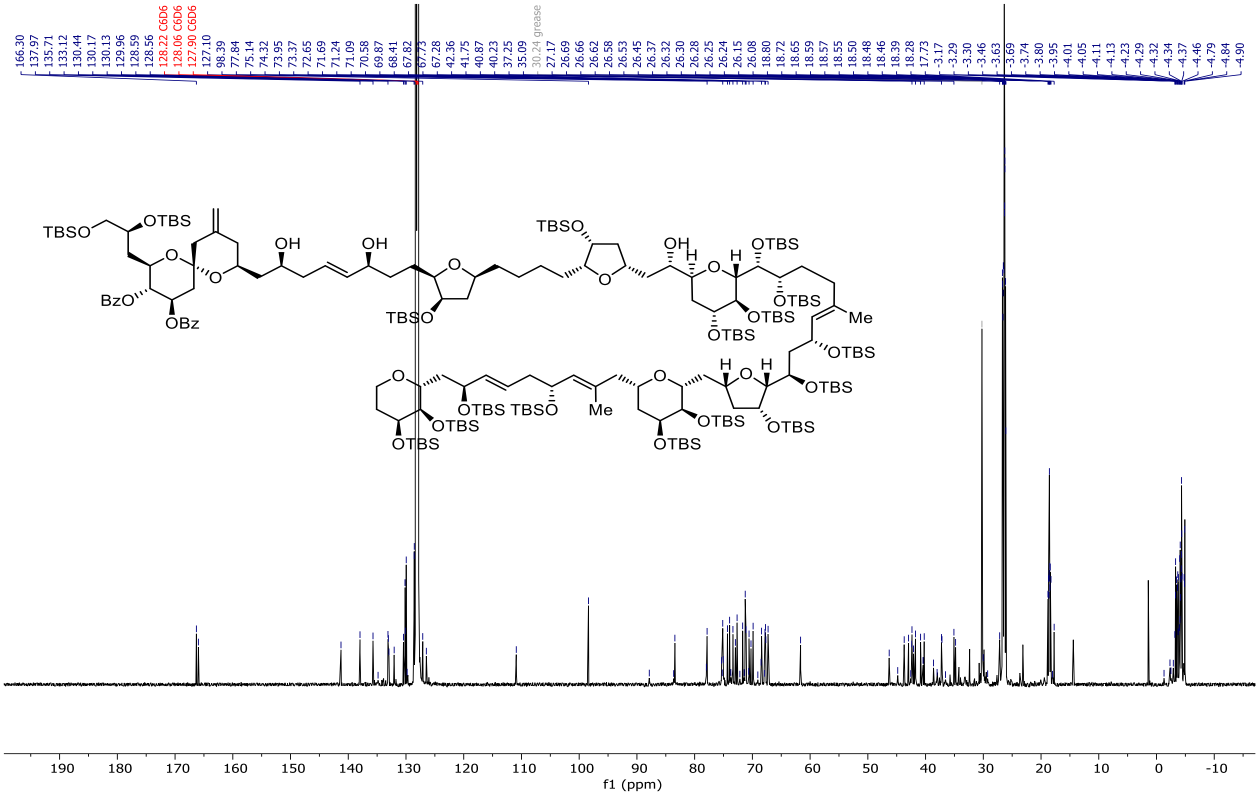

Compound 1 (nominal benthol A): ^{1}H NMR (600 MHz, [D_4]-MeOH)

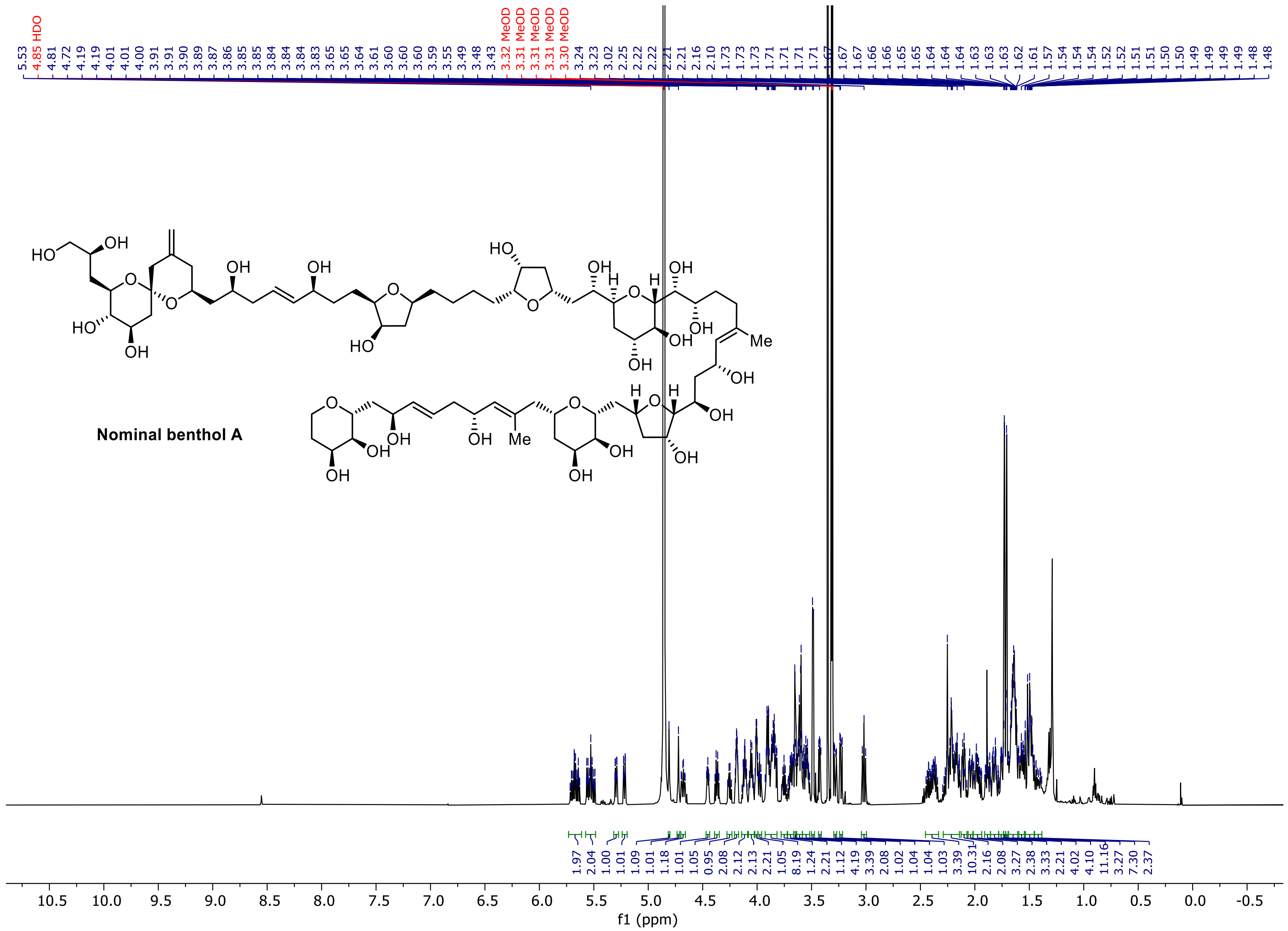

Compound 1 (nominal benthol A): ^{13}C NMR (151 MHz, [D$_4$]-MeOH)

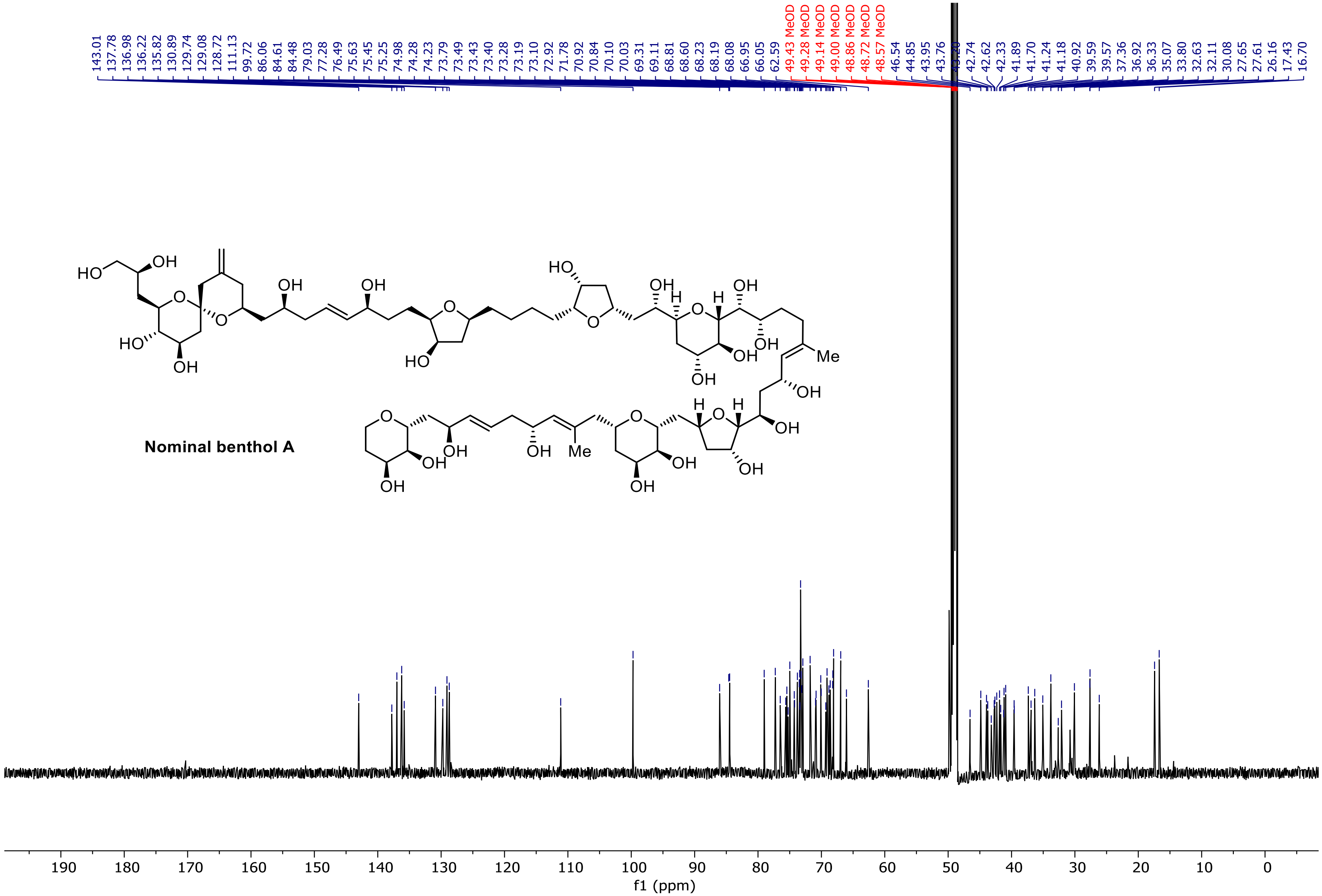

Compound 1 (nominal benthol A): ^{1}H-^{1}H COSY ([D4]-MeOH)

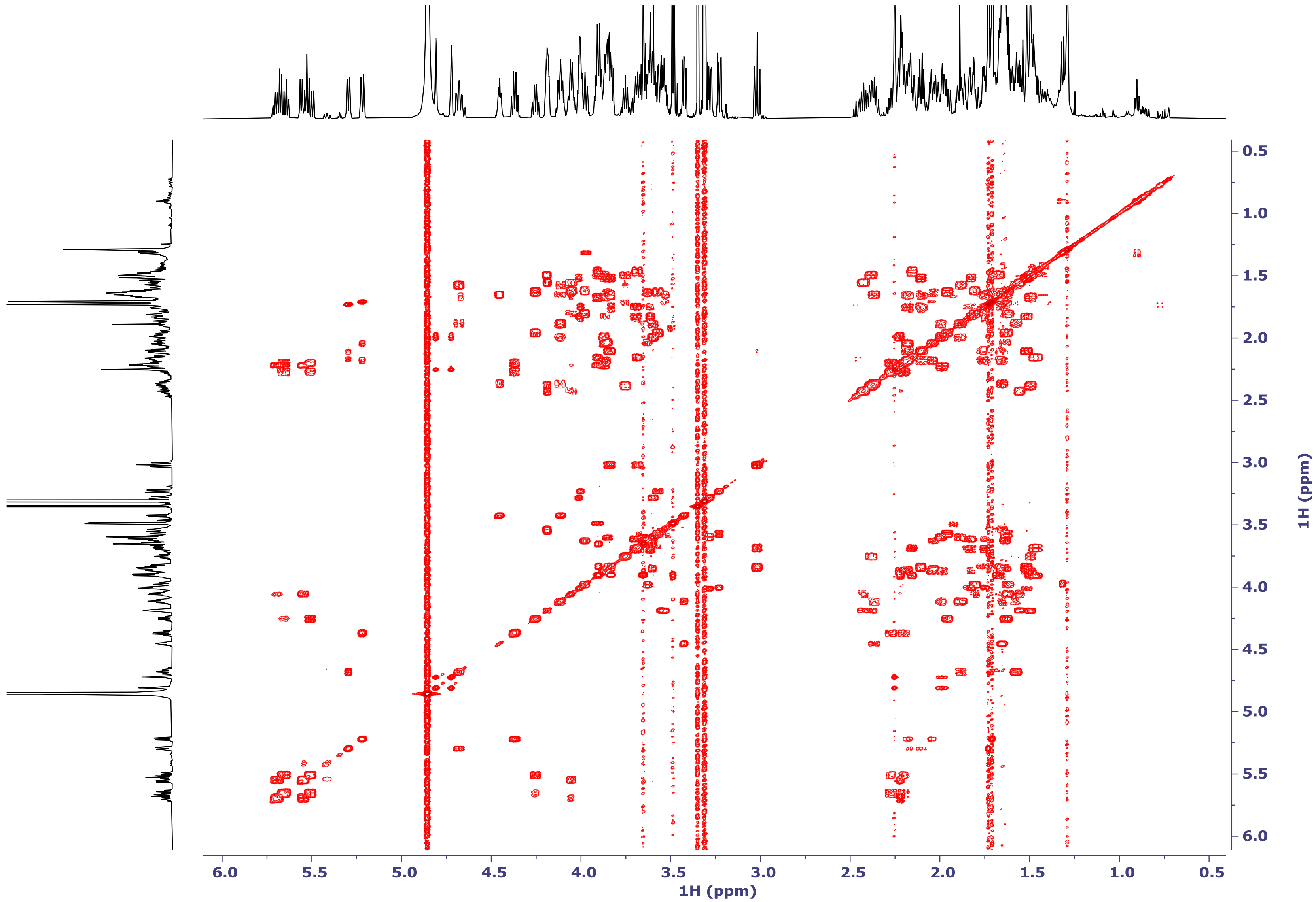

Compound 1 (nominal benthol A): HSQC NMR ([D$_4$]-MeOH)

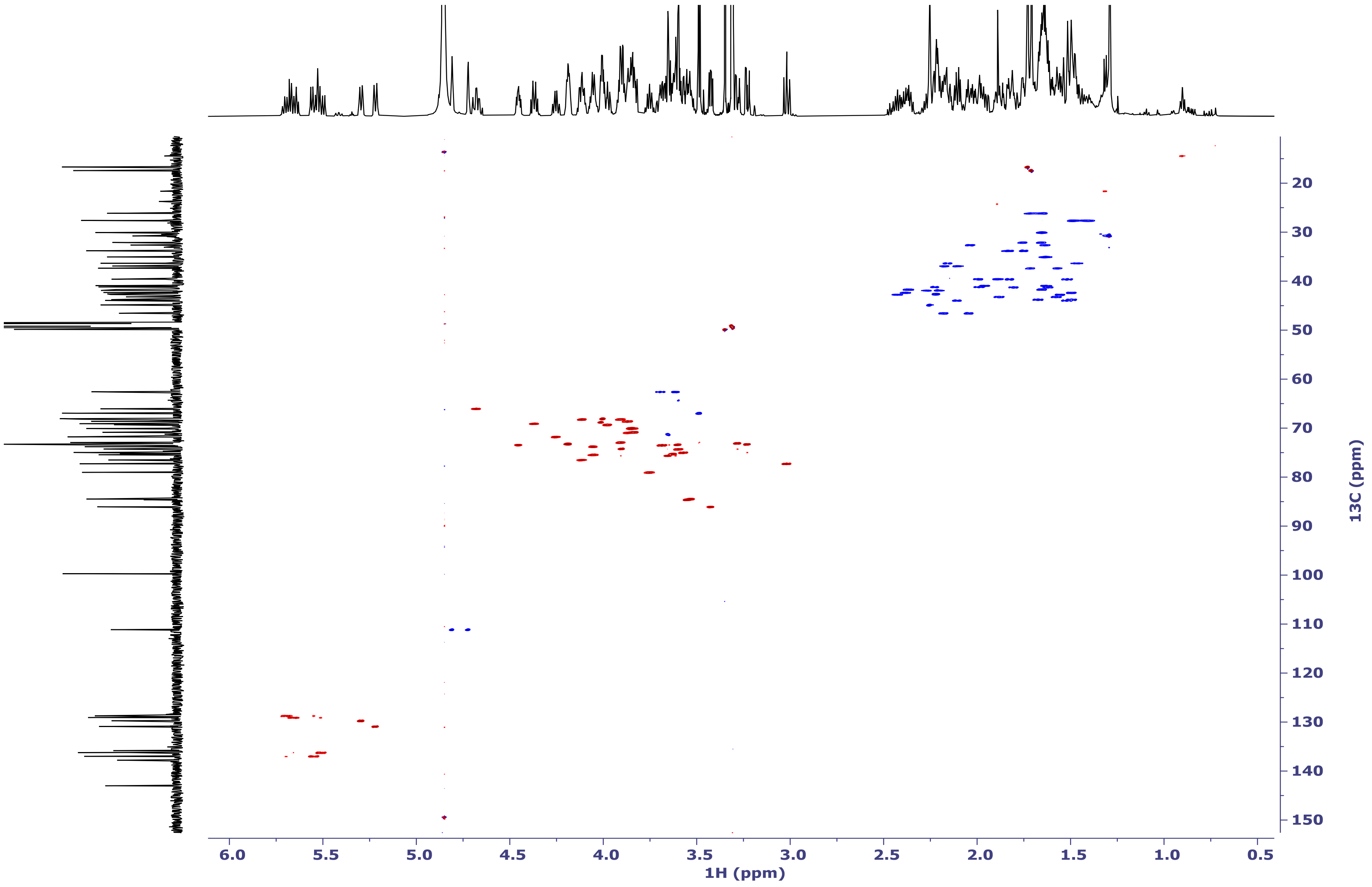

Compound 1 (nominal benthol A): HMBC NMR ([D$_4$]-MeOH)

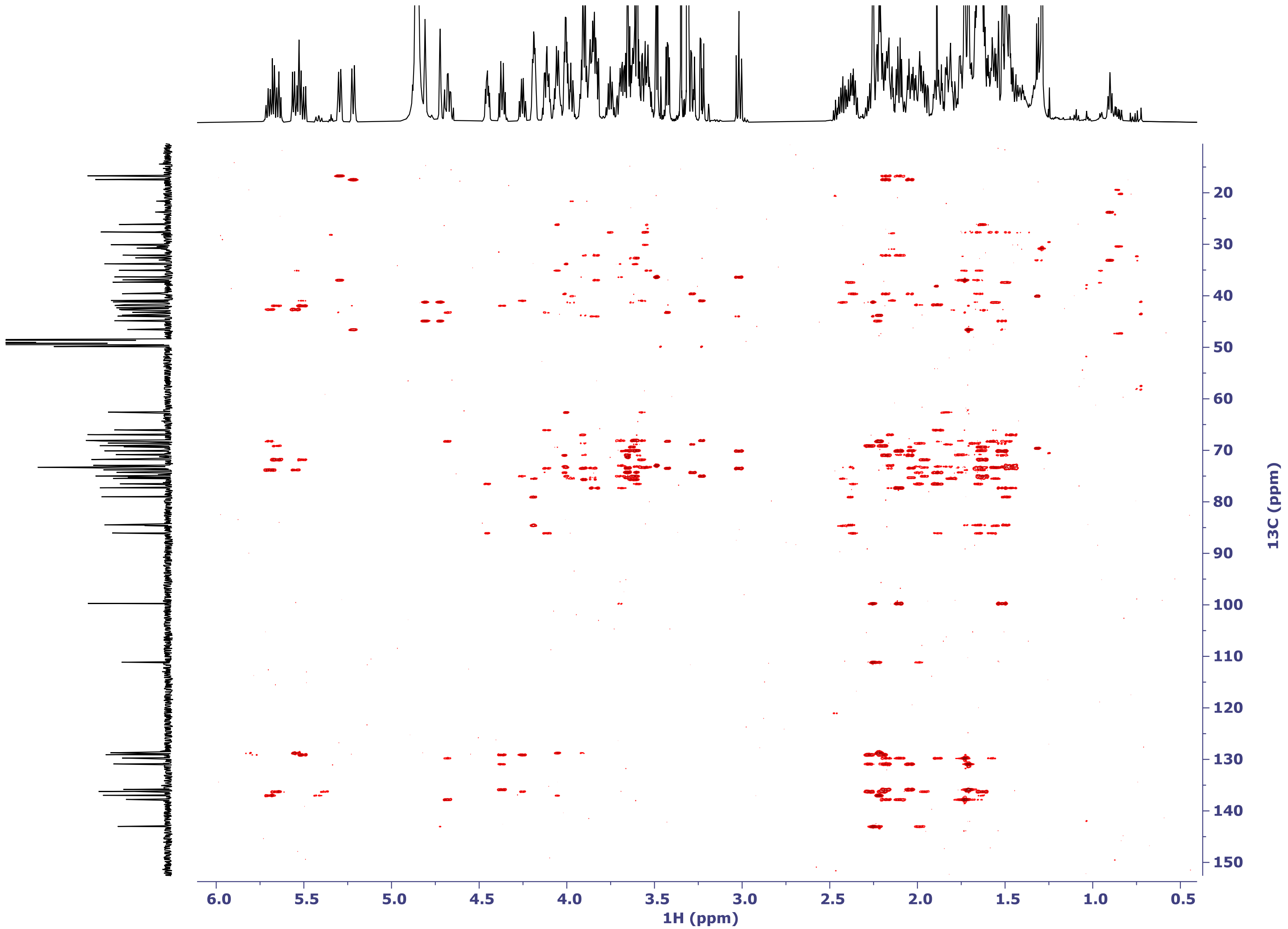

Compound 1 (nominal benthol A): ROESY ([D$_4$]-MeOH)

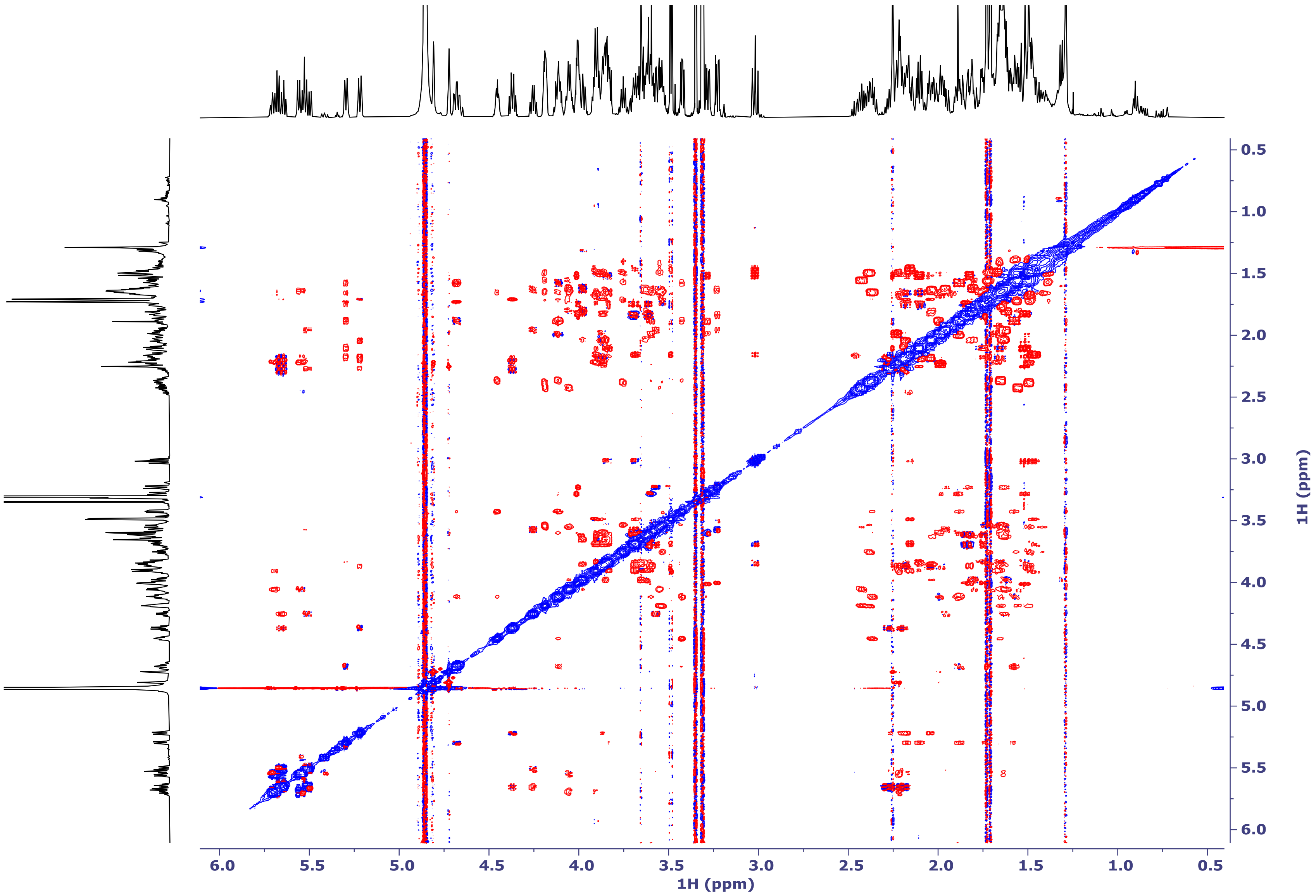

Compound 40-*epi*-39: ^{1}H NMR (600 MHz, C_6D_6)

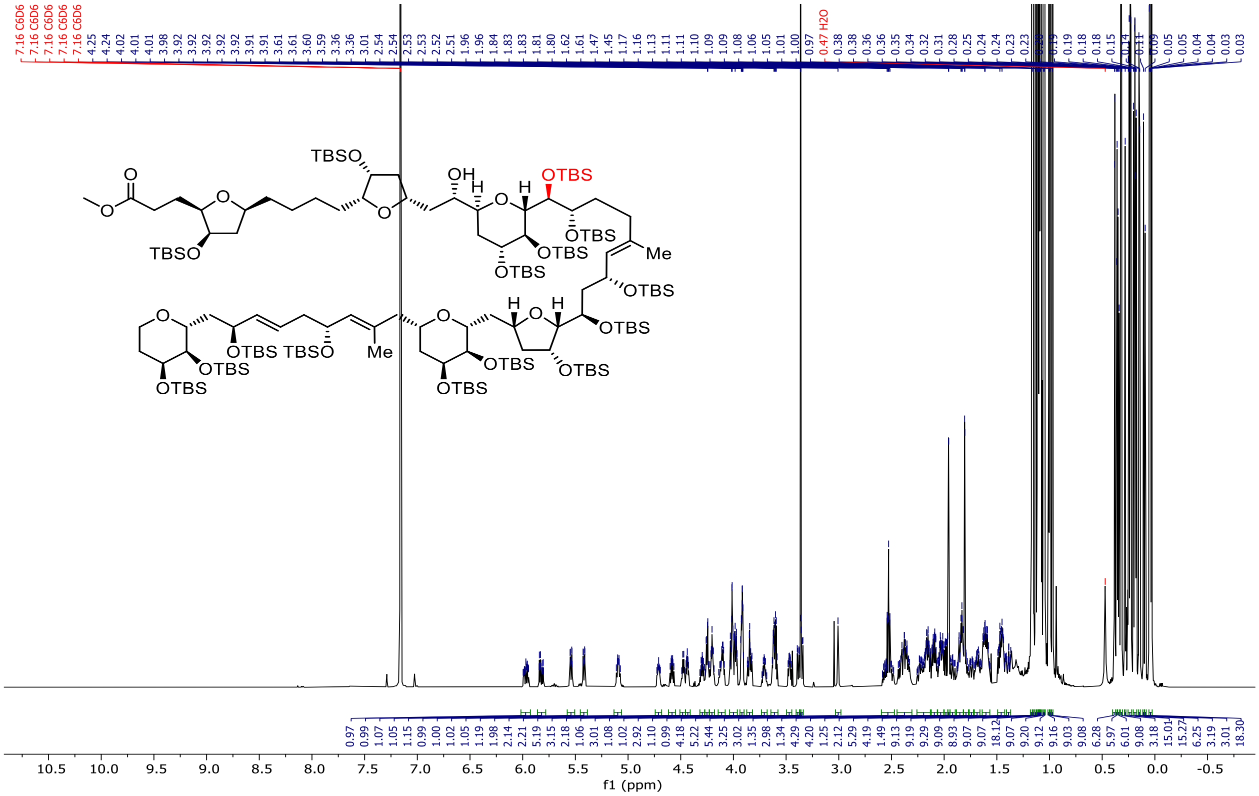

^{13}C NMR (151 MHz, C_6D_6)

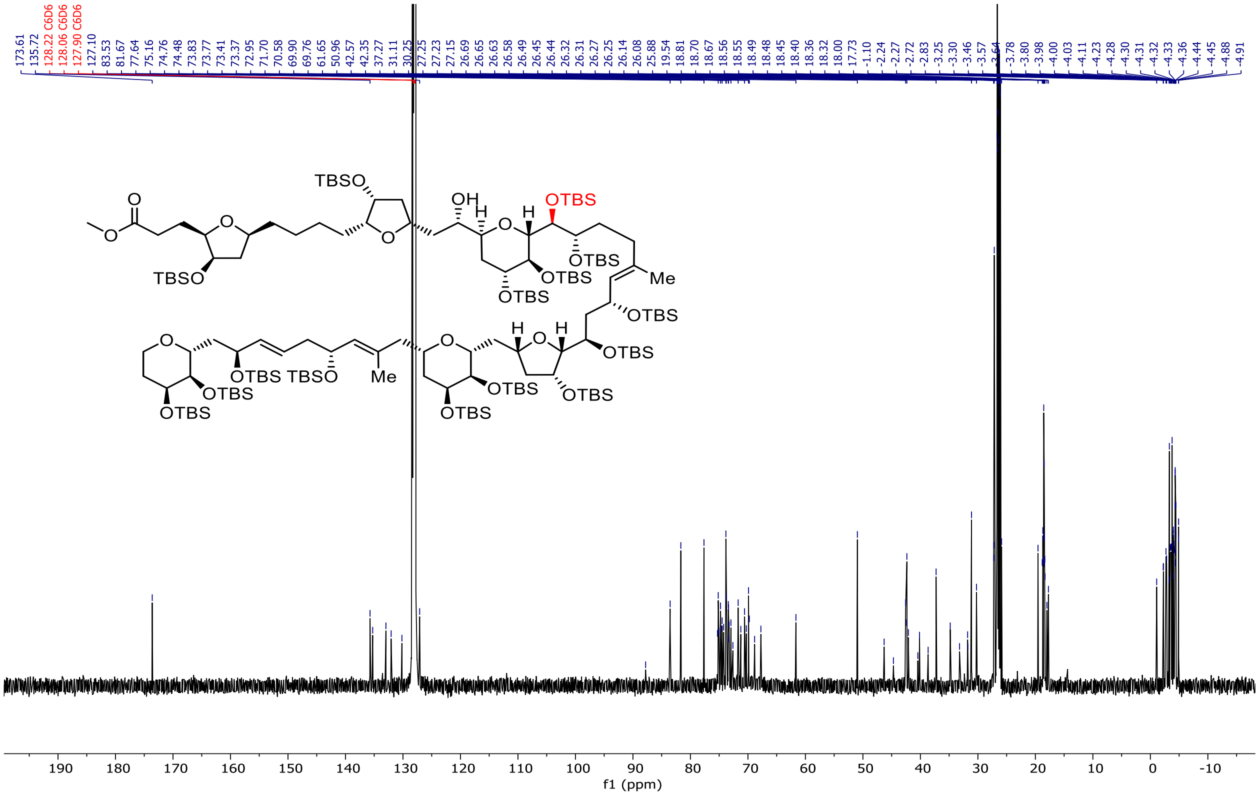

Compound 40-*epi*-40: ^{1}H NMR (600 MHz, C_6D_6)

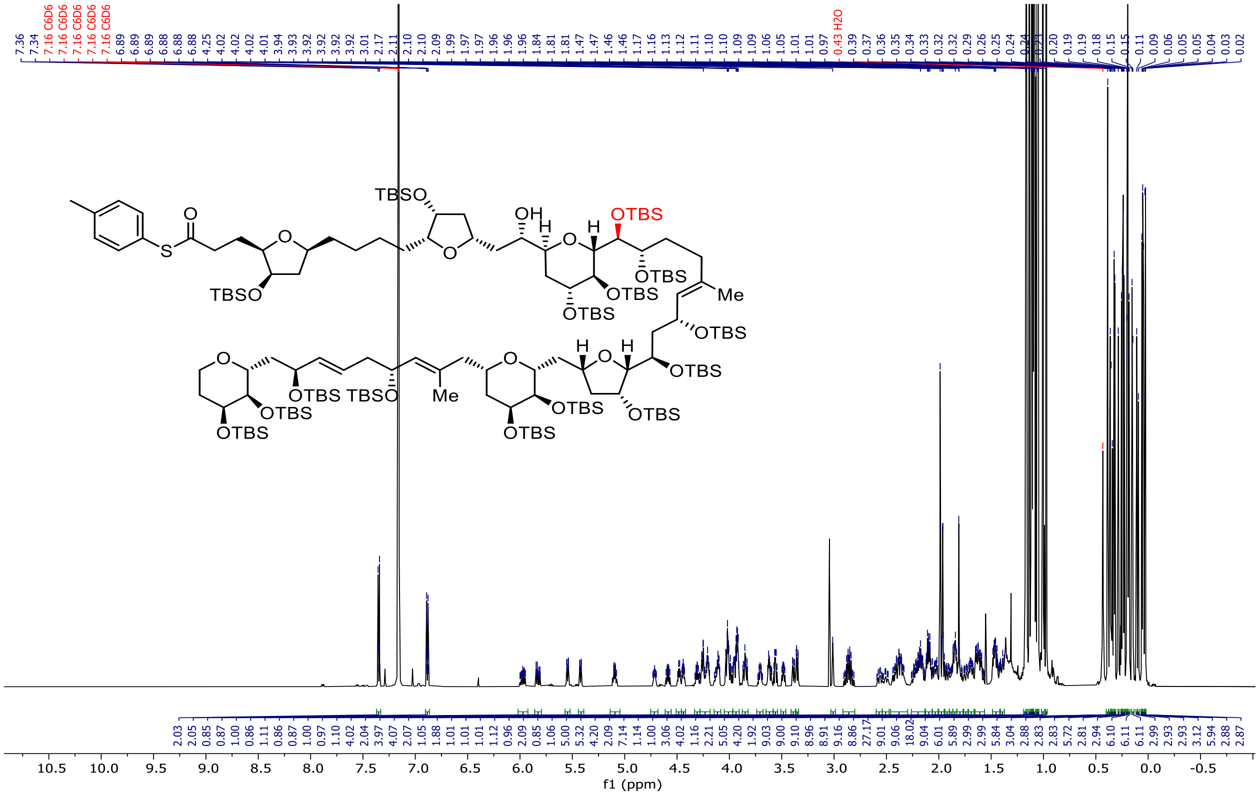

^{13}C NMR (151 MHz, C_6D_6)

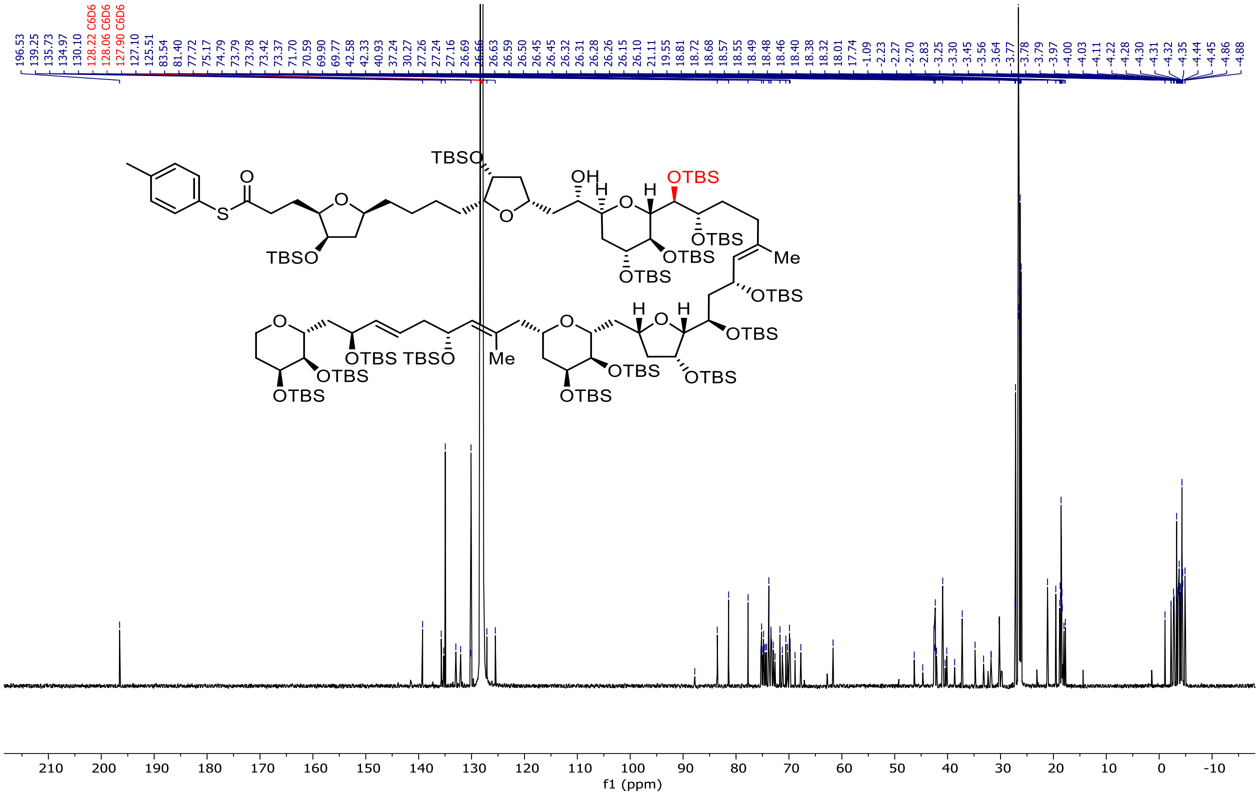

Compound 40-*epi*-42: ^{1}H NMR (600 MHz, C_6D_6)

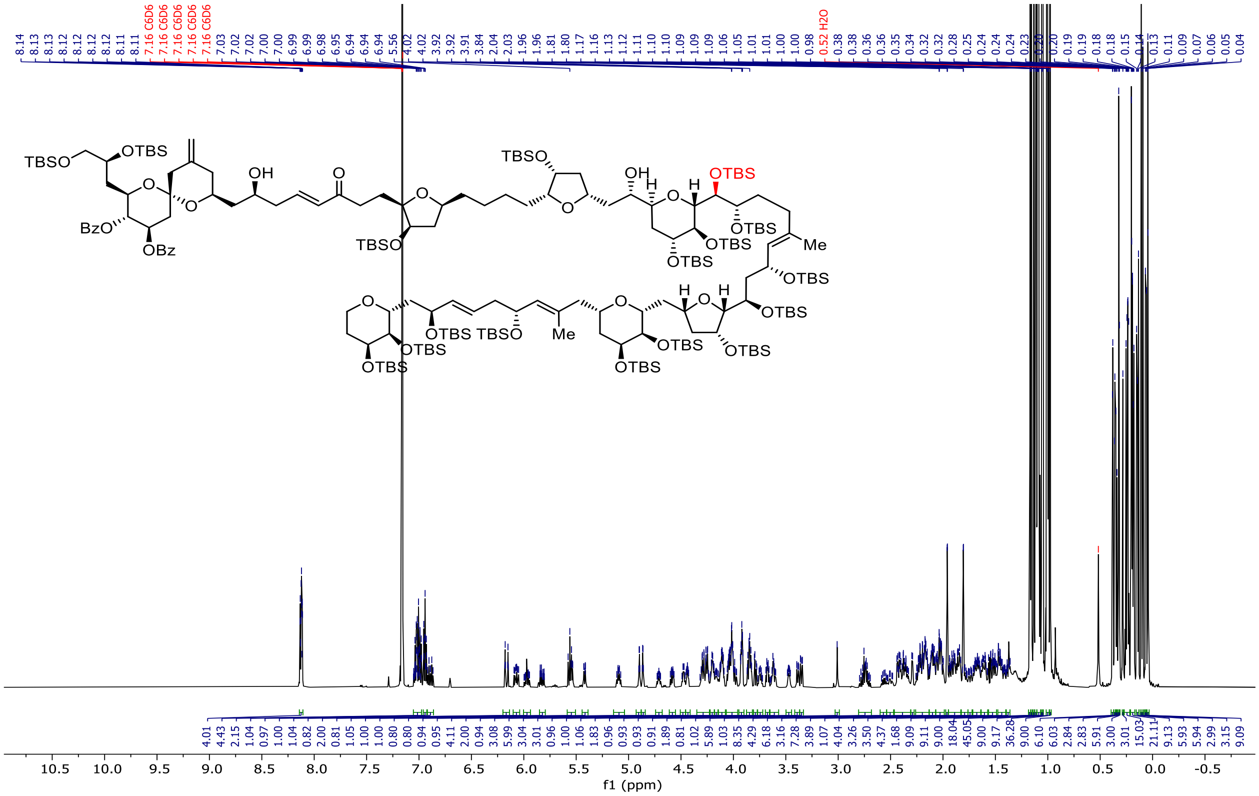

^{13}C NMR (151 MHz, C_6D_6)

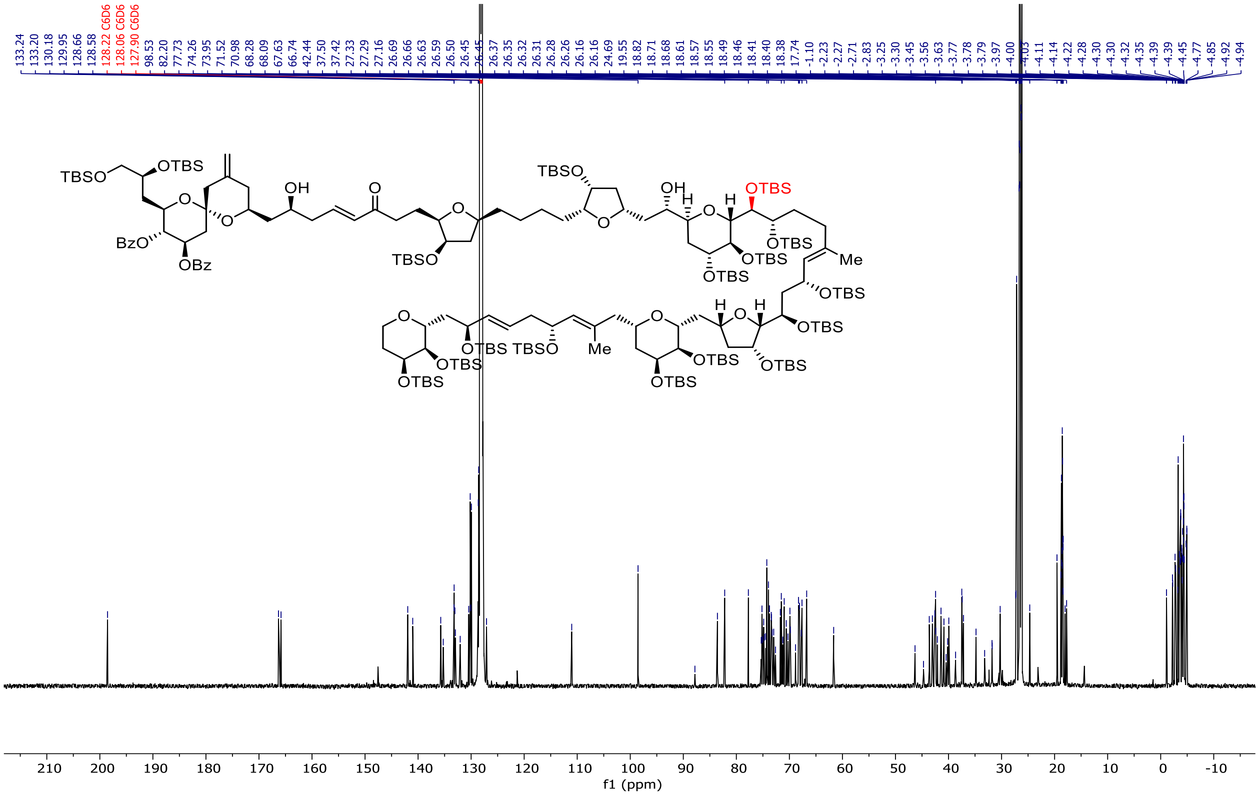

Compound 40-*epi*-43: ^{1}H NMR (600 MHz, C_6D_6)

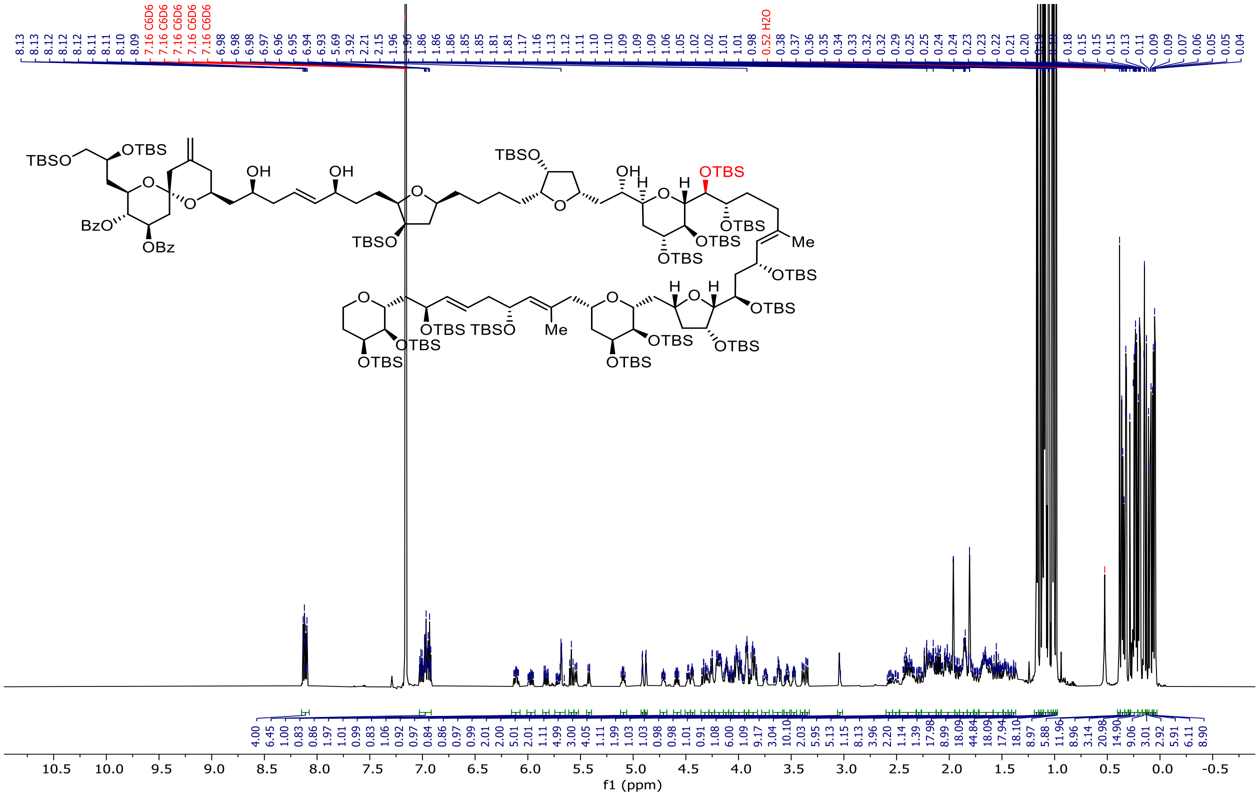

^{13}C NMR (151 MHz, C_6D_6)

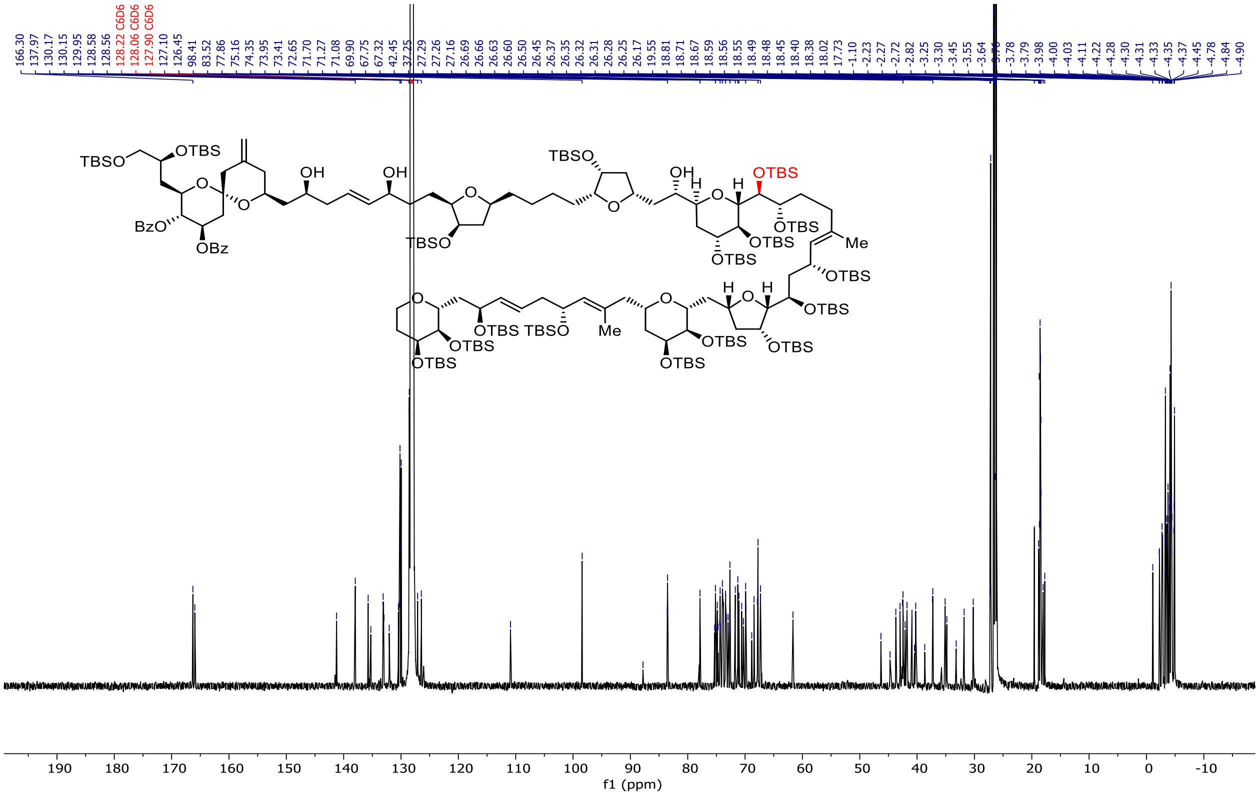

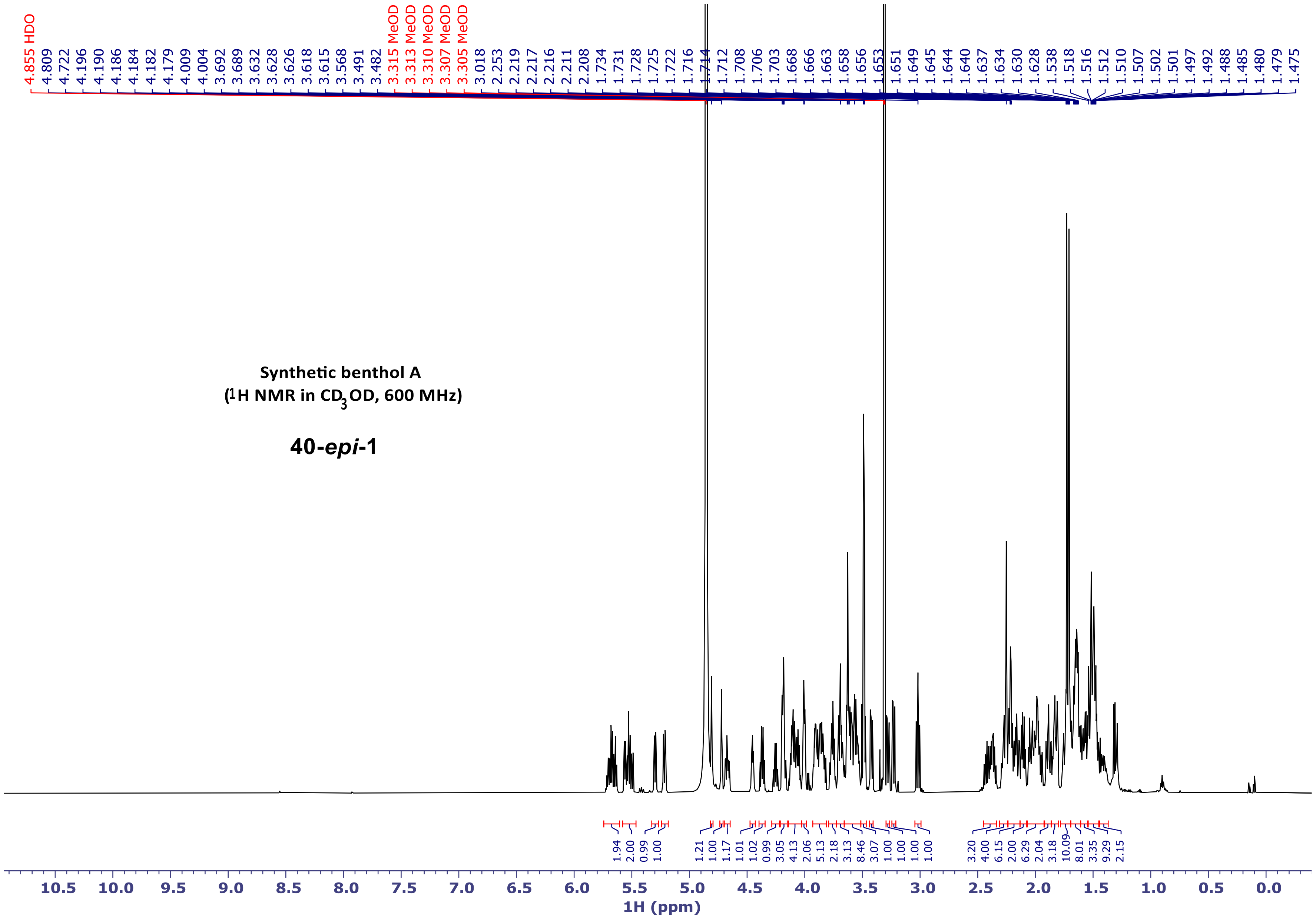
Synthetic benthol A
(^{1}H NMR in CD_3OD, 600 MHz)
40-*epi*-1
1H (ppm)

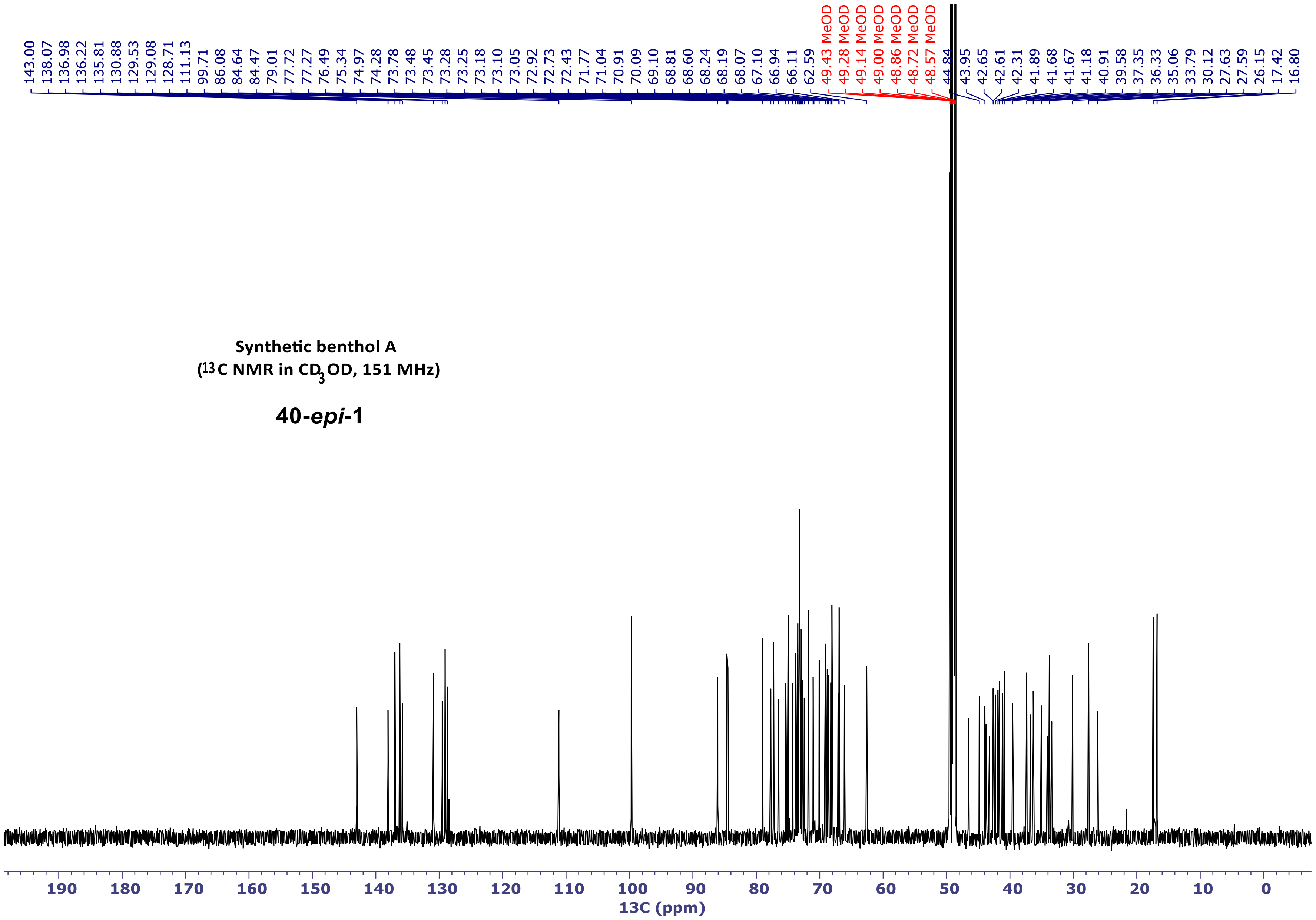
143.00
138.07
136.98
136.22
135.81
130.88
129.53
129.08
128.71
111.13
99.71
86.08
84.64
84.47
79.01
77.72
77.27
76.49
75.34
74.97
74.28
73.78
73.48
73.45
73.28
73.25
73.18
73.10
73.05
72.92
72.73
72.43
71.77
71.04
70.91
70.09
69.10
68.81
68.60
68.24
68.19
68.07
67.10
66.94
66.11
62.59
49.43 MeOD
49.28 MeOD
49.14 MeOD
49.00 MeOD
48.86 MeOD
48.72 MeOD
48.57 MeOD
44.84
43.95
42.65
42.61
42.31
41.89
41.68
41.67
41.18
40.91
39.58
37.35
36.33
35.06
33.79
30.12
27.63
27.59
26.15
17.42
16.80
Synthetic benthol A
(13C NMR in CD3OD, 151 MHz)
40-epi-1
13C (ppm)

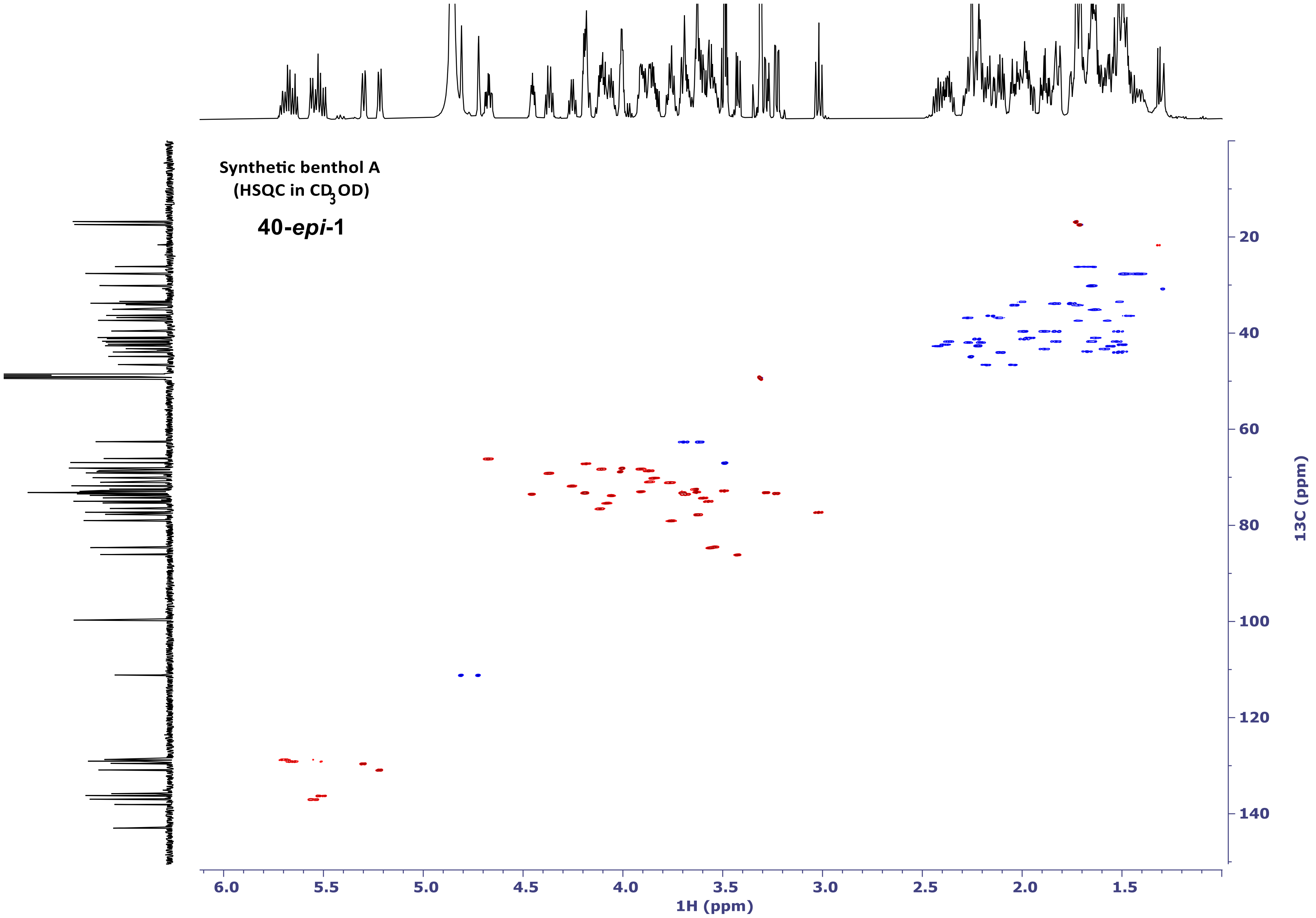
Synthetic benthol A
(HSQC in CD_3OD)
40-*epi*-1
1H (ppm)
13C (ppm)
6.0
5.5
5.0
4.5
4.0
3.5
3.0
2.5
2.0
1.5
20
40
60
80
100
120
140

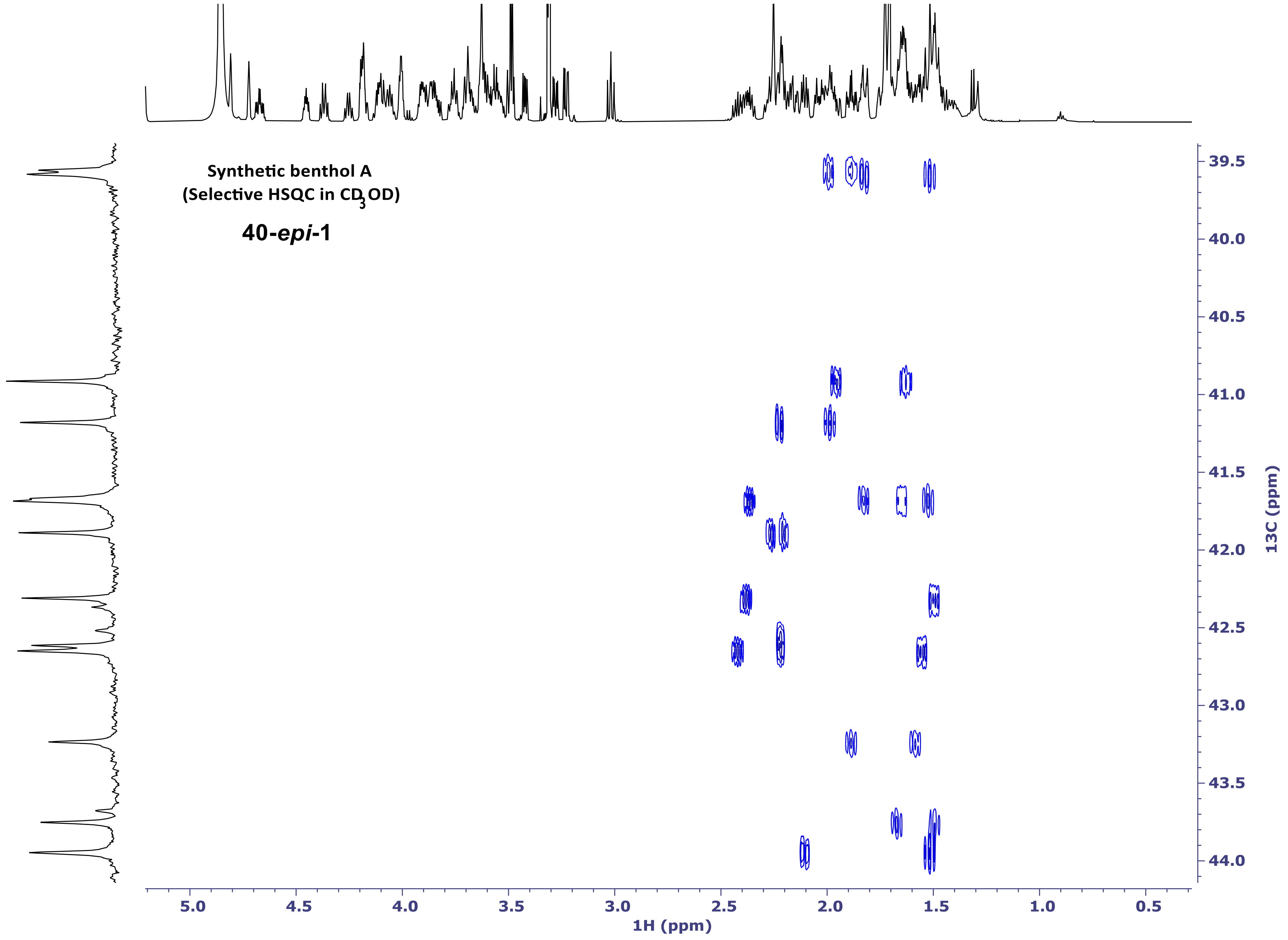
Synthetic benthol A
(Selective HSQC in CD3OD)
40-epi-1
39.5
40.0
40.5
41.0
41.5
42.0
42.5
43.0
43.5
44.0
13C (ppm)
5.0
4.5
4.0
3.5
3.0
2.5
2.0
1.5
1.0
0.5
1H (ppm)

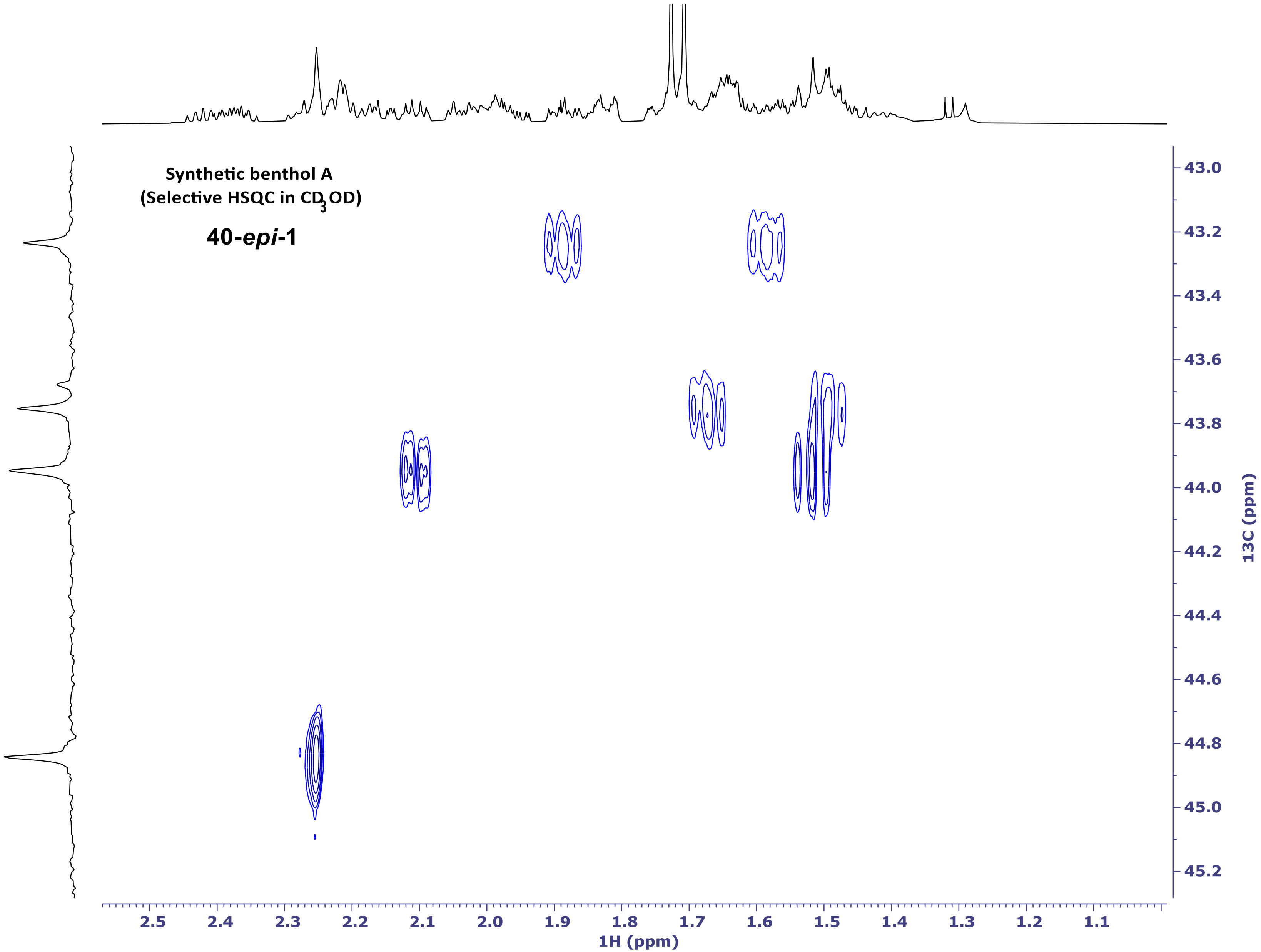
Synthetic benthol A
(Selective HSQC in CD3OD)
40-epi-1
43.0
43.2
43.4
43.6
43.8
44.0
44.2
44.4
44.6
44.8
45.0
45.2
13C (ppm)
2.5
2.4
2.3
2.2
2.1
2.0
1.9
1.8
1.7
1.6
1.5
1.4
1.3
1.2
1.1
1H (ppm)

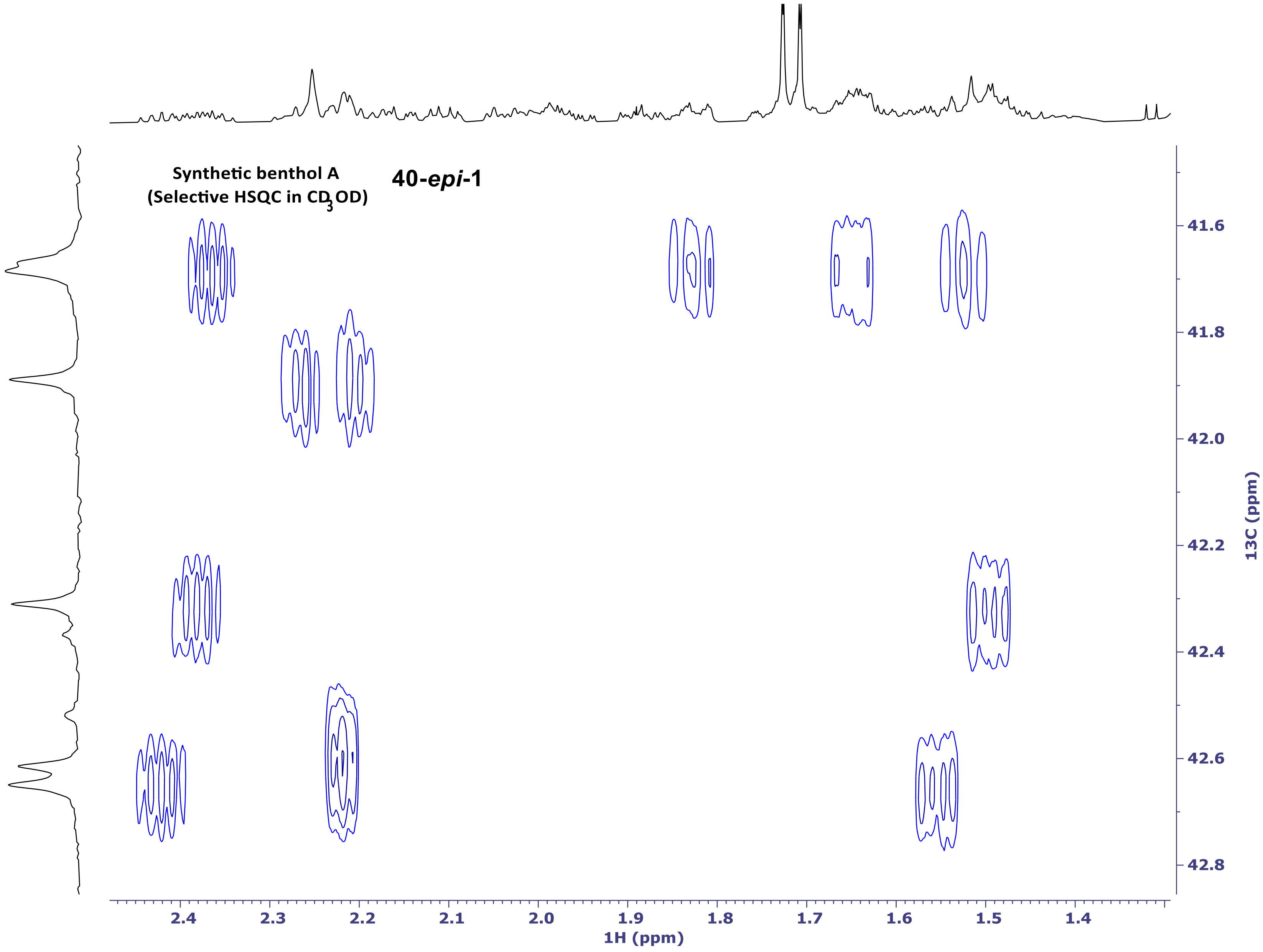
Synthetic benthol A
(Selective HSQC in CD3OD)
40-epi-1
41.6
41.8
42.0
42.2
42.4
42.6
42.8
13C (ppm)
2.4
2.3
2.2
2.1
2.0
1.9
1.8
1.7
1.6
1.5
1.4
1H (ppm)

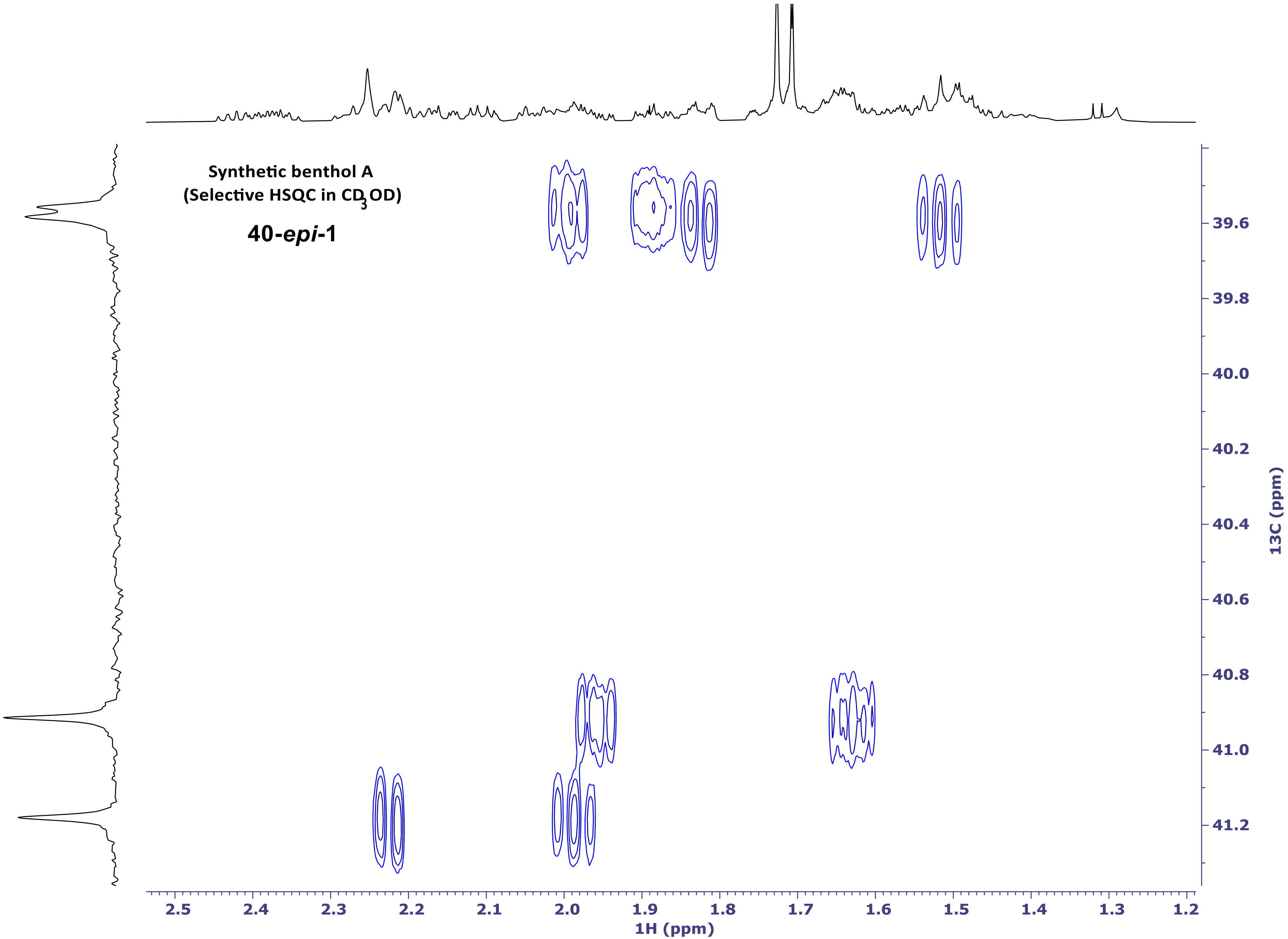
Synthetic benthol A
(Selective HSQC in CD3OD)
40-epi-1
2.5
2.4
2.3
2.2
2.1
2.0
1.9
1.8
1.7
1.6
1.5
1.4
1.3
1.2
1H (ppm)
39.6
39.8
40.0
40.2
40.4
40.6
40.8
41.0
41.2
13C (ppm)

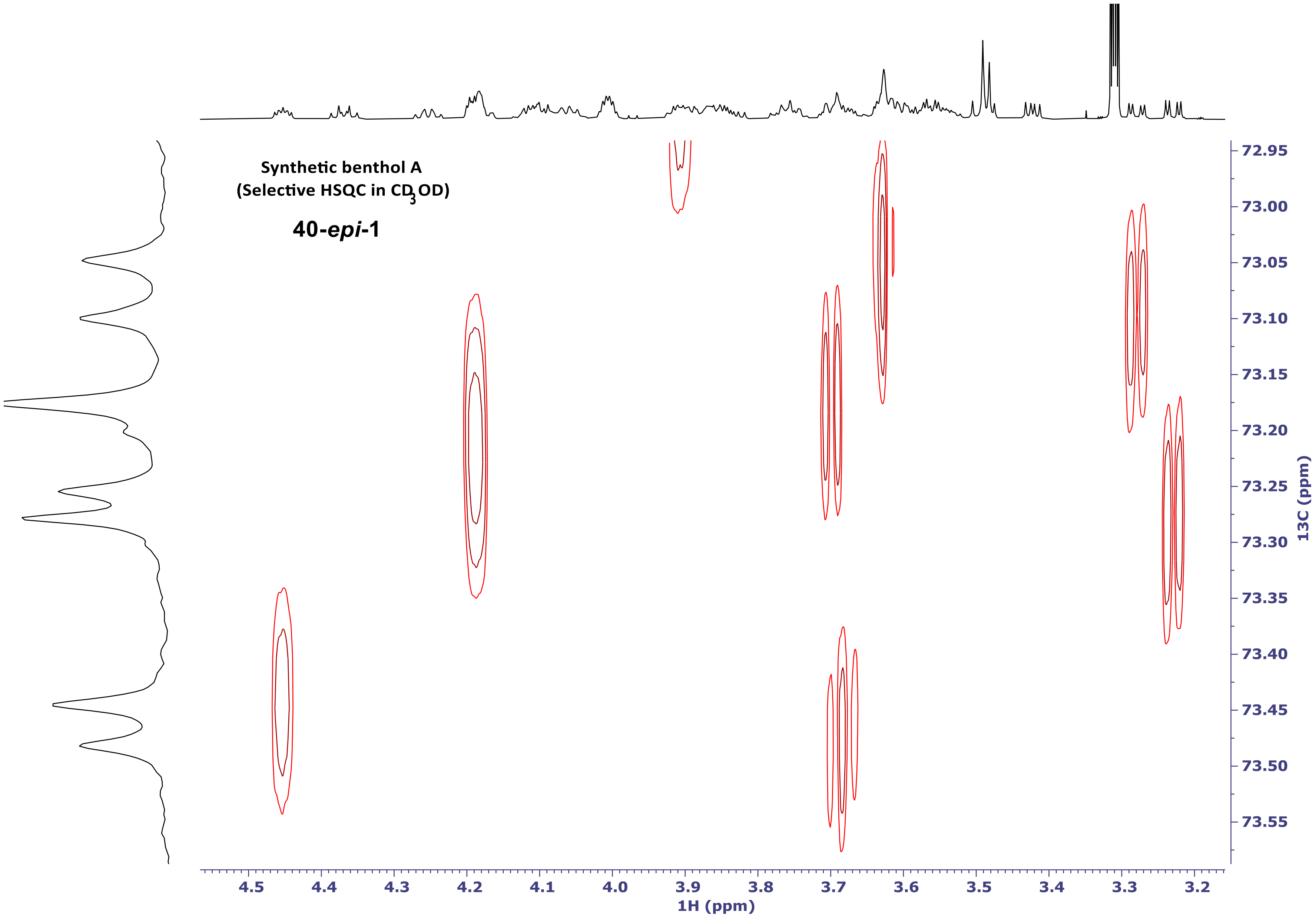

Synthetic benthol A
(Selective HSQC in CD3OD)
40-epi-1
72.95
73.00
73.05
73.10
73.15
73.20
73.25
73.30
73.35
73.40
73.45
73.50
73.55
13C (ppm)
4.5
4.4
4.3
4.2
4.1
4.0
3.9
3.8
3.7
3.6
3.5
3.4
3.3
3.2
1H (ppm)

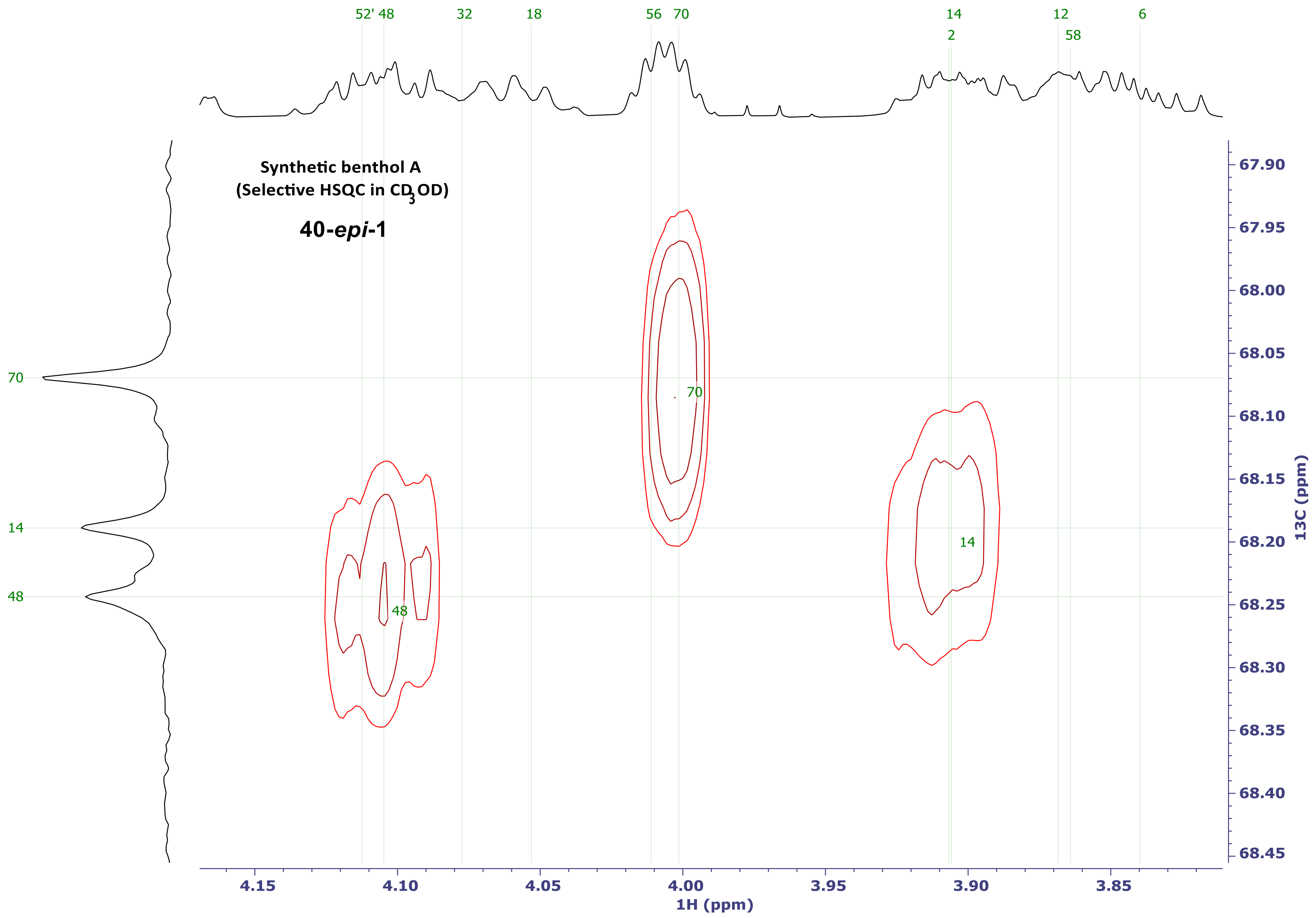
Synthetic benthol A
(Selective HSQC in CD_3OD)
40-*epi*-1
52' 48
32
18
56 70
14
2
12
58
6
70
14
48
70
14
48
67.90
67.95
68.00
68.05
68.10
68.15
68.20
68.25
68.30
68.35
68.40
68.45
13C (ppm)
4.15
4.10
4.05
4.00
3.95
3.90
3.85
1H (ppm)

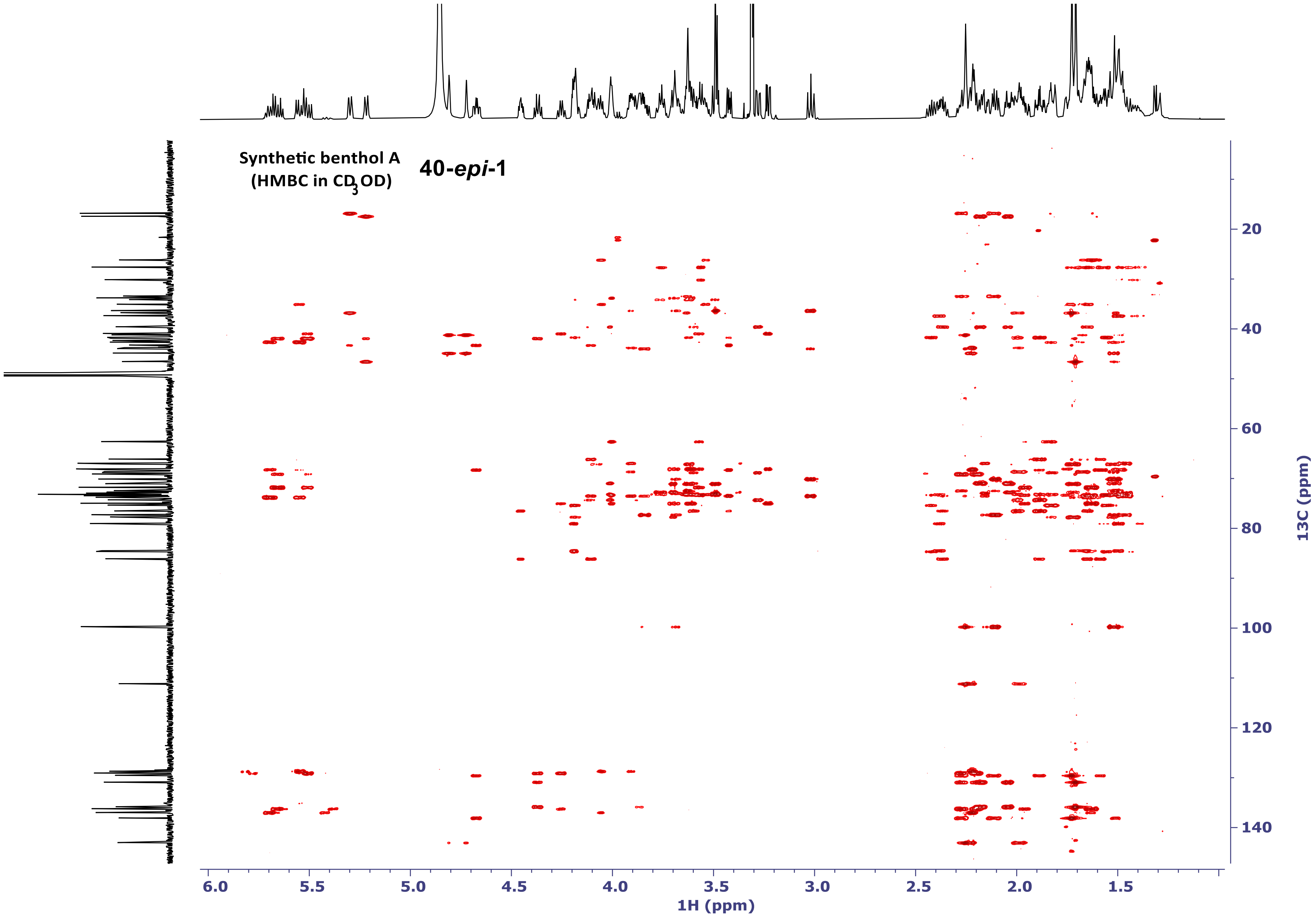
Synthetic benthol A
(HMBC in CD_3OD)
40-*epi*-1
6.0
5.5
5.0
4.5
4.0
3.5
3.0
2.5
2.0
1.5
1H (ppm)
20
40
60
80
100
120
140
13C (ppm)

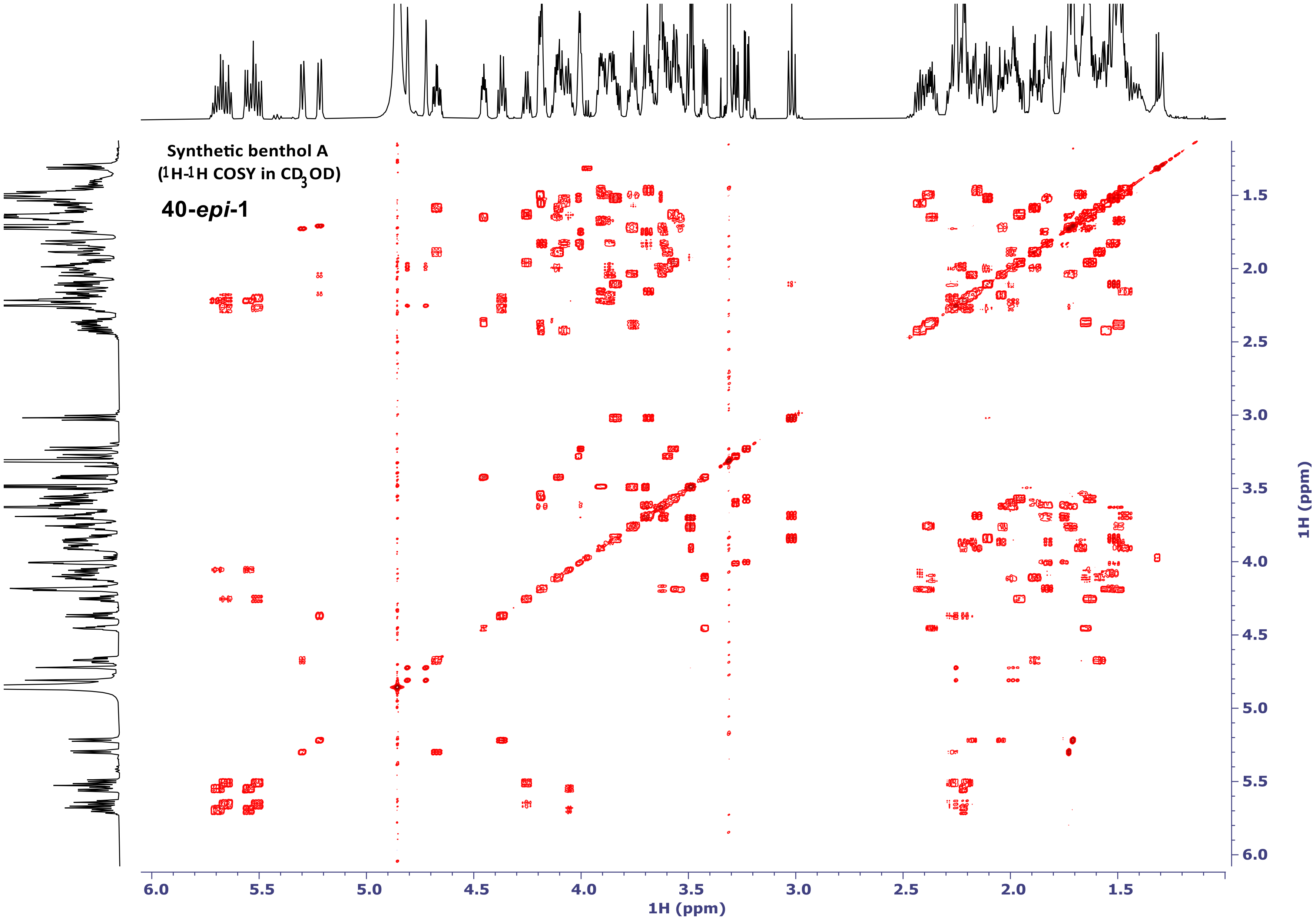

Synthetic benthol A
(1H-1H COSY in CD3OD)
40-epi-1
1H (ppm)
1H (ppm)
6.0
5.5
5.0
4.5
4.0
3.5
3.0
2.5
2.0
1.5

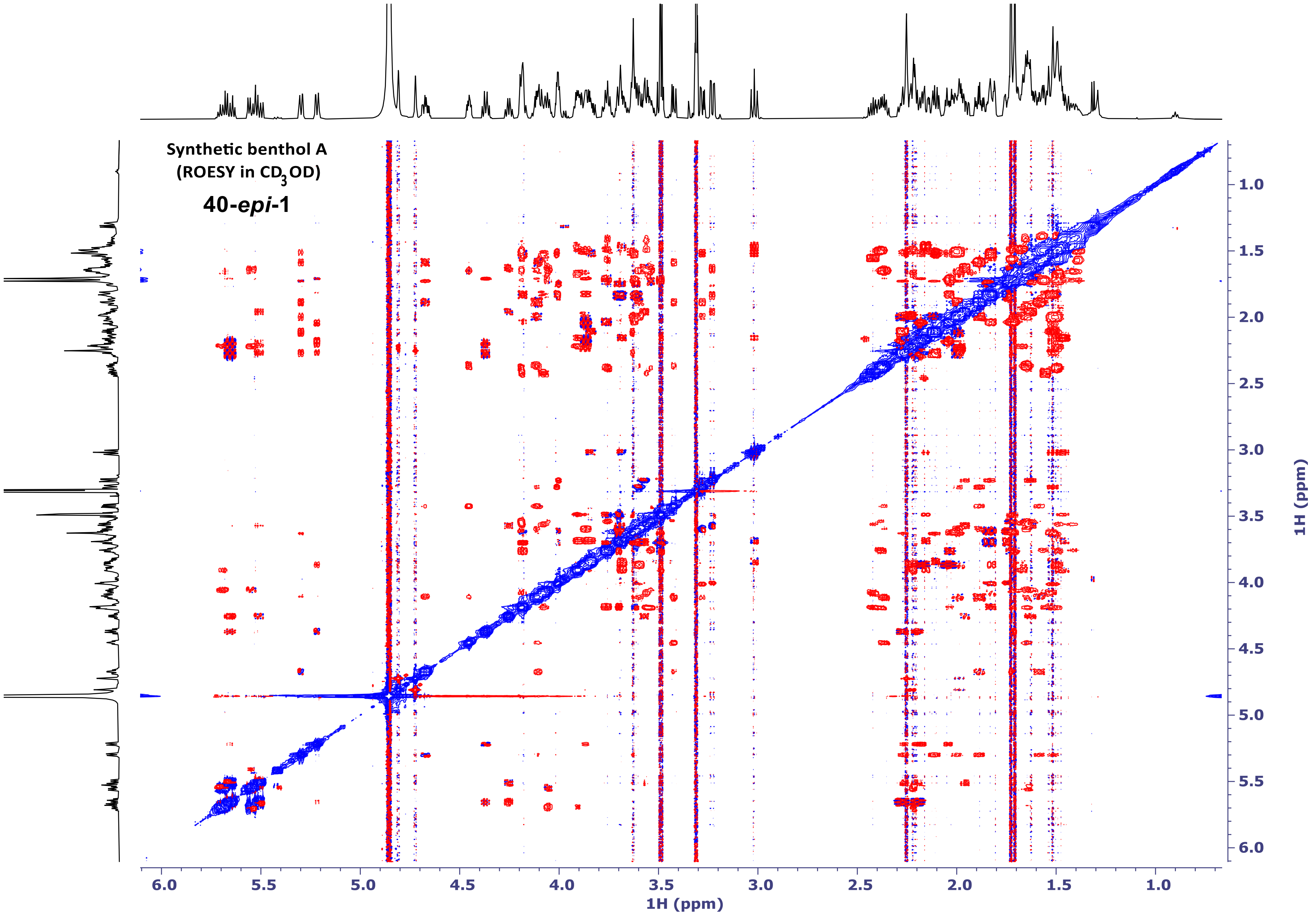

Synthetic benthol A
(ROESY in CD_3OD)
40-*epi*-1
6.0
5.5
5.0
4.5
4.0
3.5
3.0
2.5
2.0
1.5
1.0
1H (ppm)
1H (ppm)

References

1. Frick, J. A.; Klassen, J. B.; Bathe, A.; Abramson, J. M.; Rapoport, H., An Efficient Synthesis of Enantiomerically Pure (*R*)-(2-Benzyloxyethyl)oxirane from (*S*)-Aspartic Acid. *Synthesis* **1992,** 621-623.

2. Hoye, T. R.; Jeffrey, C. S.; Shao, F., Mosher ester analysis for the determination of absolute configuration of stereogenic (chiral) carbinol carbons. *Nat. Protocols* **2007,** *2* (10), 2451-2458.

3. Corey, E. J.; Helal, C. J., Reduction of Carbonyl Compounds with Chiral Oxazaborolidine Catalysts: A New Paradigm for Enantioselective Catalysis and a Powerful New Synthetic Method. *Angew. Chem. Int. Ed.* **1998,** *37* (15), 1986-2012.

4. Jia, K.; Cao, R.; Hua, D. H.; Li, P., Study of Class I and Class III Polyhydroxyalkanoate (PHA) Synthases with Substrates Containing a Modified Side Chain. *Biomacromolecules* **2016,** *17* (4), 1477-1485.

5. Martin, P.; Mueller, M.; Flubacher, D.; Boudier, A.; Blaser, H.-U.; Spielvogel, D., Total Synthesis of Hematoporphyrin and Protoporphyrin: A Conceptually New Approach. *Org. Process Res. Dev.* **2010,** *14* (4), 799-804.

6. Brown, H. C.; Singaram, B., Improved procedures for the synthesis of diisopinocampheylborane of high optical purity. *J. Org. Chem.* **1984,** *49* (5), 945-947.

7. Jiang, Z.-P.; Sun, S.-H.; Yu, Y.; Mándi, A.; Luo, J.-Y.; Yang, M.-H.; Kurtán, T.; Chen, W.-H.; Shen, L.; Wu, J., Discovery of benthol A and its challenging stereochemical assignment: opening up a new window for skeletal diversity of super-carbon-chain compounds. *Chem. Sci.* **2021,** *12* (30), 10197-10206.

8. Cohen, T.; Gapinski, R. E., The conversion of esters to phenyl thioesters by boron thiophenoxide and aluminum thiophenoxide. *Tetrahedron Lett.* **1978,** *19* (45), 4319-4322.

9. Wittenberg, R.; Srogl, J.; Egi, M.; Liebeskind, L. S., Ketone Synthesis under Neutral Conditions. Cu(I) Diphenylphosphinate-Mediated, Palladium-Catalyzed Coupling of Thiol Esters and Organostannanes. *Org. Lett.* **2003,** *5* (17), 3033-3035.
